# A Human Pharmacogenomics Approach Provides Insight Into the Pathogenesis and Pathophysiology of Steroid-Induced Ocular Hypertension

**DOI:** 10.1016/j.xops.2026.101239

**Published:** 2026-05-21

**Authors:** Zeyuan Song, Satyabrata Pany, Shengru Guo, Arpan G. Mazumder, Tatsuo Itakura, Jonathan Huang, Evan Magarychoff, Anastasia Gurinovich, Penelope H. Benchek, W. Daniel Stamer, Francis W. Price, Colin E. Willoughby, Srinivasan Senthilkumari, Ronnie J. George, Srujana Chitipothu, Jonathan H. Lass, Sudha K. Iyengar, Stephen G. Schwartz, Anthony J. Griswold, Paola Sebastiani, Marianne O. Price, M. Elizabeth Fini

**Affiliations:** 1 Institute for Clinical Research and Health Policy Studies, Tufts Medical Center, Boston, Massachusetts; 2 Tufts University Clinical and Translational Science Institute, Boston, Massachusetts; 3 New England Eye Center, Tufts Medical Center, Boston, Massachusetts; 4 The Dr. John T. Macdonald Foundation Department of Human Genetics and the John P. Hussman Institute for Human Genomics, University of Miami Miller School of Medicine, Miami, Florida; 5 Department of Ophthalmology, School of Medicine, Tufts University, Boston, Massachusetts; 6 USC Institute for Genetic Medicine, Keck School of Medicine of USC, University of Southern California, Los Angeles, California; 7 Graduate School of Biomedical Sciences, Tufts University, Boston, Massachusetts; 8 Department of Medicine, School of Medicine, Tufts University, Boston, Massachusetts; 9 Department of Population & Quantitative Health Sciences, Case Western Reserve University, Cleveland, Ohio; 10 Department of Ophthalmology, Duke University, Durham, North Carolina; 11 Price Vision Group, Indianapolis, Indiana; 12 Centre for Genomic Medicine, Biomedical Sciences Research Institute, Ulster University, Northern Ireland, UK; 13 Department of Ocular Pharmacology, Aravind Medical Research Foundation, Madurai, Tamil Nadu, India; 14 Medical Research Foundation, Smt. Jadhavbai Nathamal Singhvee Glaucoma Service, Sankara Nethralaya, Chennai, Tamil Nadu, India; 15 Central Research Instrumentation Facility, Vision Research Foundation, Chennai, Tamil Nadu, India; 16 Department of Ophthalmology and Visual Sciences, Case Western Reserve University School of Medicine and University Hospitals Eye Institute, Cleveland, Ohio; 17 Department of Epidemiology and Biostatistics, Case Western Reserve University, Cleveland, Ohio; 18 Bascom Palmer Eye Institute, University of Miami Miller School of Medicine, Naples, Florida; 19 Data Intensive Study Center, Tufts University, Boston, Massachusetts; 20 Cornea Research Foundation of America, Indianapolis, Indiana; 21 Department of Cell and Neurobiology, Keck School of Medicine of USC, University of Southern California, Los Angeles, California

**Keywords:** Pharmacogenomic, GWAS, Glucocorticoid, Steroid-induced ocular hypertension, Glaucoma

## Abstract

**Purpose:**

To identify single nucleotide polymorphisms (SNPs) and their likely target genes associated with steroid-induced ocular hypertension (SI-OHT), and to conduct functional annotation analyses.

**Design:**

Genome-wide association study (GWAS).

**Participants:**

Patients with Fuchs endothelial corneal dystrophy were enrolled after corneal transplantation at a single clinical practice (N = 439*)*. Patients self-administered 1% prednisolone acetate eyedrops after surgery. Intraocular pressure (IOP) was measured at baseline and at 1, 3, 6, and 12 months postsurgery.

**Methods:**

Saliva samples were collected, and DNA was extracted and genotyped. A GWAS was then conducted, with maximum change in IOP serving as the quantitative trait (QT). Replication analysis employed 4 independent cohorts (N = 49–103) with participants that had been previously genotyped.

**Main Outcome Measures:**

Linear regression analysis was performed to determine association between QT and genotype using the Haplotype Reference Consortium reference panel for imputation.

**Results:**

A total of 46 SNPs of genome-wide significance (*P* < 5E-08) clustered at 29 different risk loci in a total of 623 SNPs of suggestive significance (*P* < 5E-06) clustered at 323 risk loci. Most SNPs are rare or of low frequency with large effect sizes. A list of 441 prioritized target genes was validated by comparison to gene profiling study results and by annotation analyses. Of the top 29 risk loci, 31% colocalize with those for other high-tension OHT phenotypes, and 2 more are linked to OHT by biological evidence for a total of 38% overlap. Three of the discovered SNPs were independently replicated. Many of the prioritized target genes are not expressed in trabecular meshwork or juxtacanalicular tissue, contrary to the current disease paradigm. Annotation analyses suggest novel pathophysiologic mechanisms.

**Conclusions:**

Why some individuals develop SI-OHT, but others do not, has remained unknown since glucocorticoids were first used to treat eye disease in the early 1950s. Results of this study demonstrate a genetic basis for SI-OHT, support a relationship with other high-tension OHT phenotypes, and provide hypothesis-generating information for laboratory follow-up. Discovered and replicated SNPs may be valuable for SI-OHT risk prediction and prioritized target genes might be targeted for SI-OHT management.

**Financial Disclosures:**

Proprietary or commercial disclosure may be found in the Footnotes and Disclosures at the end of this article.

Glucocorticoids (GCs), commonly used to treat inflammatory diseases, alter gene expression with both beneficial and adverse consequences.^1^^,^^2^ When treated with GCs in the eye, 30%–50% of adults are “steroid responders” who develop elevated intraocular pressure (IOP), also known as ocular hypertension (OHT),^3^^,^^4^ which can lead to glaucoma. Why some individuals are steroid responders, while others are not, has remained an unanswered question since the 1950s, when GCs were first used to treat eye disease.

Like all OHT phenotypes, steroid-induced OHT (SI-OHT) is due to an increase in aqueous outflow resistance.^5^ Glucocorticoids cause mitochondrial dysfunction,^6^ leading to fibrosis in the trabecular meshwork (TM) and juxtacanalicular tissue (JCT) that abuts Schlemm's canal, with accompanying tissue stiffening.^3^^,^^4^ Aqueous outflow is thus impeded. Fibrosis is also seen in the OHT phenotype of primary open-angle glaucoma (POAG) as a primary effect^3^^,^^4^ and in pseudoexfoliation syndrome as a secondary effect,^7^ but not in the OHT phenotype of primary angle-closure glaucoma (PACG).^8^ Significantly, almost all patients with POAG are steroid responders. Moreover, steroid responders who do not have POAG are at higher risk of developing POAG compared with nonresponders, and individuals with a family history of POAG are more likely to develop SI-OHT.^3^^,^^4^ Understanding mechanistic relationships among the different conditions causing OHT could lead to better treatments.

Glucocorticoids regulate transcription by activating the GC receptor (GR), a transcription factor (TF).^1^^,^^2^ Treatment of cultured TM cells with GCs increases biosynthetic activity and expression of genes associated with fibrosis; however, this occurs in TM cells from steroid responders and nonresponders alike.^3^^,^^4^ To identify GC-induced changes in gene expression that could tip the balance to pathology, much previous work has employed a gene expression profiling approach,^3^^,^^4^ most recently with a paired eye study design that couples gene expression profiling to aqueous outflow measurement.^9^, ^10^, ^11^ However, gene expression profiling, as applied thus far, employs only a single cell type (TM cells) treated with GCs for only a few days. Moreove, it is subject to cell culturing artifacts, and the N is only 3-4 individuals.

Another possible approach is the genome-wide association study (GWAS), which has been used very successfully to discover single nucleotide polymorphisms (SNPs) and their target genes that confer risk for various OHT phenotypes.^12^ The advantage of a GWAS is that it involves a large number of individuals in an investigation of the actual disease. Typical of complex disease,^13^ SNPs associated with the common OHT phenotypes are found almost entirely in noncoding regions of the genome and likely regulate gene expression.^14^, ^15^, ^16^, ^17^, ^18^ Complex diseases exhibit relatively small effect sizes, meaning that very large cohorts must be enrolled. Single nucleotide polymorphisms discovered thus far are of mostly common frequency, accounting only for a small percentage of the estimated heritability in the case of POAG. In contrast, drug response typically manifests much larger effect sizes over a much shorter observation period,^19^ increasing the potential to reveal rare variants.^20^ Thus, an SI-OHT GWAS has the potential to uncover many more target genes than GWASs for other OHT phenotypes.

Transcriptional response to GCs in the eye has been shown to vary widely depending on the specific GC, dose, time course of treatment, and mode of delivery.^3^^,^^4^ Enrollment of large and uniform cohorts has proven elusive;^3^ however, small pilot studies conducted independently by our 2 different teams yielded variants of genome-wide significance.^21^^,^^22^ Recognizing the opportunity of the pharmacogenomics GWAS, our teams combined resources for a larger study. For the discovery cohort, we enrolled patients with Fuchs endothelial corneal dystrophy (FECD) who underwent Descemet membrane endothelial keratoplasty (DMEK) or Descemet stripping endothelial keratoplasty (DSEK) at a single clinical practice. Patients received prednisolone acetate eyedrops after surgery to suppress transplant rejection. Our objective in this study was to identify genomic variants and their likely target genes associated with SI-OHT and to conduct follow-up bioinformatics analyses to provide insight into pathogenesis and pathophysiology, and the relationship to other forms of OHT.

## Methods

### Study Approval

All cohorts were compliant with the Declaration of Helsinki and the US Health Insurance Portability and Accountability Act. Institutional review board/ethics committee approval was obtained for all cohorts (University of Southern California, Tufts University, University of Miami, Case Western Reserve University, Vision Research Foundation).

### Statistical Power for Discovery

We used the software package PASS (NCSS Statistical Software) to perform power calculations for cohort size. Figure S1 (available at www.ophthalmologyscience.org) shows the minimum detectable effect size for a quantitative trait (QT) across a range of minor allele frequencies (MAFs) and *α*_*p*_s, assuming 80% power. Our calculations indicated that a cohort of ∼400 subjects is powered for detection of (1) common SNPs (MAF >0.05) at an effect size ≥1.02–0.79 mmHg, (2) low-frequency SNPs (MAF = 0.01 to 0.05) at an effect size of 1.79 to 2.24 mmHg, and (3) rare SNPs (MAF <0.01) at an effect size of >2.24 mmHg. Detection of MAF = 0.001 would require an effect size of 5.48–7.04 mmHg. Detection of MAF = 0.0001 would require an effect size of 17.31–22.25 mmHg.

### Cohorts

#### Discovery Cohort

All participants in the Indianapolis-1 discovery cohort (N = 439) were patients with FECD enrolled into the registry of the Cornea Research Foundation of America (Indianapolis, IN) between the years 2003 and 2012 after DMEK or DSEK at the Price Vision Group (Indianapolis, IN). All received topical steroids to suppress transplant rejection. Patients were instructed to instill prednisolone acetate 1% eye drops, 4 times daily for ≥4 months, then taper by 1 drop per month to once daily (the exact timing of the taper varied).

This regimen was continued indefinitely unless the patient developed OHT, in which case the topical corticosteroid strength, potency, or dosing frequency was reduced and/or topical glaucoma medication was added as needed to maintain IOP within the target range. Of the patients who did not experience IOP elevation, 96% were on prednisolone acetate 1% once daily at the 12-month time point. The mean ( ± standard deviation) time to the start of the prednisolone taper was 132 ± 52 days.

Baseline IOP was recorded prior to surgery and at follow-up visits at ∼1, 3, 6, and 12 months after surgery. All IOP measurements were done with Goldmann applanation tonometry (Haag-Streit). Intraocular pressure measurements were not adjusted for central corneal thickness because the nomogram for IOP adjustment was developed in patients with normal corneal biomechanics and is not validated for use in patients with FECD. In fact, a study that evaluated the precision of various techniques for measuring IOP in corneas with altered biomechanical properties found that Goldmann applanation tonometry and dynamic contour tonometry both measured IOP correctly in patients with FECD without adjustment for central corneal thickness, both before and after endothelial keratoplasty.^23^

Patients were contacted for participation in the Indianapolis-1 discovery cohort if they met the following inclusion–exclusion criteria:

#### Inclusion Criteria

• Age ≥18 years
• Normal baseline IOP (between 10 and 21 mmHg)
• Had DMEK or DSEK surgery for FECD
• Had postsurgical treatment with 1% PA eye drops according to standard practice protocol
• Baseline IOP measurement and ≥2 IOP measurements in the study eye over 6–12 months by Goldman applanation tonometry

#### Allowed during Participation

• Intervention by treatment with glaucoma drops in the study eye for SI-OHT resulting from GC administration.
• Intervention by reduction in dosing, potency, or frequency of GC administration in the study eye for SI-OHT resulting from GC administration.

#### Exclusion Criteria

• A history of glaucoma, suspected glaucoma, or OHT in the study eye (because POAG is a risk factor for SI-OHT)
• Prior pars plana vitrectomy in the study eye
• Prior trabeculectomy or glaucoma tube shunt surgery in the study eye
• Anterior segment neovascularization in the study eye
• Use of any medication that could lower IOP at the time of study enrollment
• Intraocular pressure spiking immediately after surgery due to surgical complications
• Any other eye surgery performed within the first year after DMEK or DSEK for FECD

The Cornea Research Foundation of America registry was searched for qualified participants with the aim of finding as many steroid responders as possible, with at least an equal number of nonresponders. A steroid responder was defined as an individual that exhibited an IOP ≥6 mmHg above baseline. Participants were handed or mailed a saliva collection kit (OG-500, Oragene DNA Self-Collection Kit, DNA Genotek, Inc) labeled with a coded identification number for confidentiality. Participants could fill and return the kit during an office visit, or they could send it back in a prepaid mailing packet.

A total of 520 participants were consented and enrolled. Participants were selected without regard to ancestry; however, most self-identified as White, non-Hispanic, in alignment with documented FECD demographics.^24^ Those ultimately selected for the GWAS were all of European ancestry (N = 439).

#### Replication Cohorts

The Indianapolis-2 replication cohort comprises a subset of participants selected from a larger cohort enrolled into a multicenter study to map genes for FECD.^25^^,^^26^ The participants were enrolled from the Cornea Research Foundation of America registry after DMEK or DSEK at the Price Vision Group between the years 2006 and 2007. Participants were selected for the current study from this larger cohort if they met the inclusion-exclusion criteria. There is no overlap with participants in the Indianapolis-1 cohort. All the selected participants were of European ancestry (N = 104) and the steroid treatment regimen, time points used for IOP measurement, and exclusive use of Goldman applanation tonography was also the same as used for the Indianapolis-1 cohort. However, participants were enrolled into the original cohort without regard for their steroid responder status. This is reflected in the lower percentage of responders versus nonresponders, in the lower mean maximum change in IOP (deltaIOPmax) for responders, and in the lower maximum IOP.

Participants were enrolled into the Florida-1 replication cohort prior to undergoing a single intravitreal injection with triamcinolone acetate (single injection of 4 mg) for various retinal inflammatory diseases. This cohort was previously used for discovery in an SI-OHT candidate gene study^27^ and for replication in our previous Florida pilot GWAS.^21^ Participants selected for GWAS were all of European ancestry (N = 50).

The Chennai-1 replication cohort (N = 52) was enrolled as the discovery cohort in our Chennai pilot study^22^ and the Chennai-2 replication cohort (N = 50) as the discovery cohort for a second study (manuscript in preparation). Participants in both cohorts were treated for various retinal diseases by intravitreal injection with triamcinolone acetate (single injection of 2 mg; Chennai-1) or an intravitreal dexamethasone slow-releasing implant (Ozurdex intravitreal implant; Chennai-2). Participants in both cohorts were of South Asian ancestry.

### Genotyping and GWAS Quality Control for the Discovery Cohort

Saliva collection kits were returned by 480 of the 520 participants enrolled (93%). A portion of DNA was extracted from each sample using the prepIT L2P Kit (DNA Genotek, Inc). Extracted DNA was stored at –70°C and the remaining saliva was stored at ambient temperature as recommended by the kit manufacturer. Samples were transferred to the University of Miami Miller School of Medicine’s Center for Genome Technology. DNA was quantified by Nanodrop, and DNA quality was evaluated by Qubit (for concentration) and gel electrophoresis (for intactness). Any DNA samples that failed these quality control (QC) assessments were re-extracted from the banked saliva.

Final DNA samples from 471 subjects passed QC review. These samples were genotyped on the Illumina Infinium Global Screening Array-24 BeadChip (Illumina, Inc). Genotyping reproducibility was confirmed by including 5 QCs and 1 blind duplicate. Sample call rates and SNP statistics were calculated. Of the 471 samples genotyped, 460 unique samples plus the 5 QCs displayed >98% call rate.

Quality control was performed at both sample and SNP levels in accordance with Illumina protocols and published procedures.^28^ Principal components of genetic ancestry and genetic relationship matrix for cryptic relationships were inferred using PC-AiR and PC-Relate programs, respectively, both implemented in our analysis pipeline.^29^

Of the 460 well-performing genotypes, 21 samples with mismatched genders/DNA sample swap/missing age value or non-European ancestry were dropped. This left 439 samples for GWAS.

### QT for the Discovery Cohort

Maximum change in IOP was used as the QT. This was defined as the difference between the presurgical baseline IOP and the maximum IOP recorded in postsurgical follow-up. Two QTs were calculated: (1) deltaIOPmax across the entire 12 months of the study (12mQT) and (2) deltaIOPmax for the first 3 months postsurgery (3mQT). All samples that passed QC were used in the 12-month analysis (N = 439). Samples for the 3-month analysis included only those participants whose IOP was recorded for both the 1-month and 3-month visits (n = 421).

### GWAS for the Discovery Cohort

Linear regression analysis was performed to determine association between QT and genotype. Single SNP association analysis was conducted using linear mixed-effects models, treating the QT as a continuous outcome variable and using the expected dosage of each SNP as the main covariate. The models were adjusted for baseline IOP, age, sex, and the first 4 principal components of genetic ancestry. In addition, an adjustment was made if an intervention occurred over the 12 months of the study; this affected only the 12-month QT, when 37% of patients had received an intervention; no interventions occurred prior to the 3-month IOP measurement. The models included genomic relationship matrix as a random effect to account for cryptic relationships among individuals. An additive genetic effects model was assumed.

Genotype data were imputed using the Haplotype Reference Consortium reference panel on the Michigan Imputation Server.^30^ Of the 481 134 SNPs successfully genotyped, 475 122 SNPs passed quality check on the server. After imputation, approximately 9.3 million SNPs were obtained. Single nucleotide polymorphisms with an imputation R^2^ > 0.7 and a minor allele count ≥3 were retained. Duplicated and ambiguous SNPs were removed.

Manhattan plots were constructed and the genomic control parameters were calculated to confirm no inflation introduced by population stratification. Quantile–quantile Q-Q plots were generated to visualize the distribution of the test statistics. R software (R Foundation for Statistical Computing) was used for these calculations and visualizations.^31^

### Genotyping, QC, QT, and GWAS for the Replication Cohorts

#### Indianapolis-2 Cohort

All DNA samples for the larger FECD cohort were genotyped on the Illumina 2.5M genotyping array (Illumina, Inc), and QC was performed as described.^32^ Genomic data for the 104 participants selected for the Indianapolis-2 cohort were downloaded from National Center for Biotechnology Information dbGaP (accession code phs000421.v1.p1).

All participants were used in the 12-month GWAS analysis (N = 104). Samples for the 3-month analysis included only those participants whose IOP was recorded for both the 1- and 3-month visits (n = 102). Maximum change in IOP was used as the QT, calculated at 12 months and 3 months postsurgery. The same GWAS models were used as for the Indianapolis-1 cohort, including at first an adjustment for intervention in the 12-month analysis. However, the resulting Q-Q plot did not support the analysis, with a genomic inflation factor of 0.58. This may be due to the small size of the cohort, combined with the small percentage of participants—only 10% as compared with the Indianapolis-1 cohort—that received an intervention in this cohort. When the adjustment for intervention was removed, the genomic inflation factor was 0.95, indicating that population stratification was addressed properly.

#### Florida-1 Cohort

Recruitment and inclusion–exclusion criteria for participants in the Florida-1 replication cohort were similar to the Indianapolis cohorts, as previously reported.^21^ Intraocular pressure was measured in follow-up visits for up to 12 months. DNA samples were genotyped in 2007 on the Affymetrix GeneChip Human Mapping 500K Array Set.^33^ Maximum change in IOP at 12 months post-GC treatment was used as the QT for those participants surviving QC (N = 50), as previously reported.^21^ This was the first SNP microarray commercially available and imputation is not possible with the data as coded at that time.

#### Chennai-1 and Chennai-2 Cohorts

Recruitment and inclusion–exclusion for the 52 participants in the Chennai-1 cohort were previously described.^22^ Inclusion–exclusion criteria for the 50 participants of the Chennai-2 cohort were the same (manuscript in preparation). Intraocular pressure measurements were made at 1, 3, and 6 months after GC treatment was begun.

DNA samples were whole genome sequenced. Low pass 5 × Whole-Genome Sequencing (Illumina Hi Seq X10) was performed at Medgenome laboratories, Bangalore. Sequencing of the prepared DNA libraries was carried out in Hi Seq X10, which generates 2X150 bp sequence reads at 5X sequencing depths (∼15 GB). A minimum of 75% of the sequenced bases was of Q30 value. FASTQ files were generated from the sequence data and were used for further analysis.

Maximum change in IOP at 6 months post-GC treatment was used as the QT. The participants were then grouped into steroid responder (IOP ≥21 mmHg), and nonresponder (IOP ≤21 mmHg) categories for a case-control study design. Quality control steps were performed using the FastQC tool. Case-control association testing was performed using the chi-square test implemented in PLINK, as previously reported.^34^ Significant SNPs were identified using the Benjamini and Hochberg multiple corrections method with a *P* value cut off of *P* < 0.05.^22^

### Genetic Burden Analysis

Independent genome-wide significant loci were identified separately for the 3-month and 12-month GWAS using a distance-based pruning approach (±1 Mb window), retaining the most significant variant within each locus. For each set of loci identified, a genetic burden score was constructed by summing the number of risk alleles carried by each individual across the selected variants. Risk alleles were defined based on the direction of effect from the corresponding GWAS; for variants with negative effect estimates, genotype coding was reversed to ensure concordant directionality across loci. Linear regression models were used to evaluate the association between the genetic burden score and quantitative IOP change (deltaIOPmax) at 12 and 3 months. Analyses were performed separately for the 12mQT and 3mQT using their respective sets of independent loci.

### Risk Locus Overlap with Other High Tension Ocular Phenotypes

A risk locus was defined as a region of the genome that clusters a group of significant and suggestive SNPs with a single protein-coding gene as their nearest neighbor. Colocalization of SIOH risk loci with those discovered for other high-tension ocular phenotypes was investigated by cross-referencing with published reports,^14^^,^^15^^,^^17^^,^^18^^,^^35^, ^36^, ^37^ including a GWAS for IOP in rats.^38^

### Independent Replication

Single nucleotide polymorphisms in the discovery dataset with a *P* < 5.0E-06 were selected for replication testing. The association of these SNPs with the outcome was tested using the same linear mixed-effects model adjusted for baseline IOP, age, sex, the first 4 principal components of genetic ancestry and include a genomic relationship matrix as a random effect.

The 3mQT association results from the Indianapolis-1, Indianapolis-2, and Florida-1 cohorts were combined using a weighted Z-score meta-analysis (Stouffer method).^39^ Meta-analysis was not performed for the 12mQT because of the differences in the GWAS model used for the different cohorts (with and without intervention as a covariate). In addition, the Chennai cohorts were not included in the meta-analysis because their *P* values were derived from chi-square tests and were not directly comparable. Prior to meta-analysis, alleles were harmonized across cohorts using coded allele frequencies, and signs were flipped when allele orientation was inconsistent. Single nucleotide polymorphisms with missing results in all secondary cohorts were excluded. Two-sided *P* values were converted to Z-scores, with effect direction incorporated via the sign of the test statistic. Cohort-specific Z-scores were combined using weights proportional to the square root of the sample size,^40^^,^^41^ and 2-sided meta-analysis *P* values were obtained from the standard normal distribution.

### Annotation

We determined gene expression in the aqueous outflow pathways (AOPs) using data curated on the Spectacle portal.^42^ We obtained MAF ALFA values by searching the National Center for Biotechnology Information's Database of Genotypes and Phenotypes (the ALFA project provides MAF values from dbGAP).^43^ We obtained gene product information from the GeneCards Suite,^44^ examined genotype-phenotype association using the Genotype Tissue Expression (GTEx) portal^45^^,^^46^ and the National Institutes of Health (NIH) Genome-Wide Repository of Associations Between SNPs and Phenotypes portal.^47^ We performed *in silico* analyses using tools on the RegulomeDB^48^ and HaploReg v4.2^49^ servers. We extracted genomic position localization and sequence information from the University of California, Santa Cruz Genome Browser^50^ and the European Molecular Biology Laboratory Nucleotide Sequence Database.^51^ We accessed disease phenotype information sourced on MedLine Plus. We performed activated GR regulation and upstream regulator analysis using the NIH Database for Annotation, Visualization, and Integrated Discovery (DAVID) Bioinformatics functional annotation clustering tool paired with the UCSF_TF database. We performed pathway enrichment analysis using the NIH DAVID Bioinformatics functional annotation clustering tool paired with the Reactome database.^52^

## Results

### Cohorts

**Figure 1:**
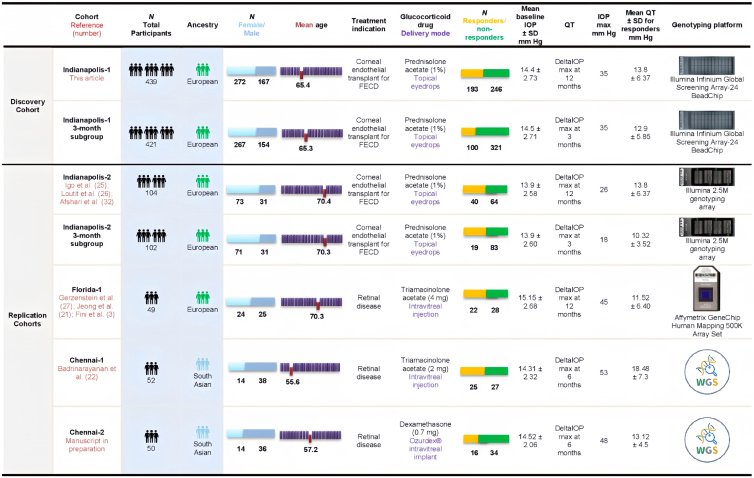
Cohorts. Demographic/clinical data and other features of the discovery and replication cohorts are shown. Steroid responders were participants who exhibited a positive change in IOP ≥6 mmHg above their baseline. deltaIOPmax = the difference between maximum intraocular pressure and baseline intraocular pressure; FECD = Fuchs endothelial corneal dystrophy; IOP = intraocular pressure; QT = quantitative trait; SD = standard deviation; WGS = whole-genome sequencing.

Clinical and demographic information about the discovery cohort and the 4 replication cohorts is summarized in Figure 1. All participants in the Indianapolis-1 discovery cohort were patients with FECD who underwent DMEK or DSEK surgery at a single clinical practice in Indianapolis, Indiana. Patients were instructed to self-administer 1% prednisolone acetate eyedrops daily after surgery to suppress transplant rejection. Baseline IOP was recorded prior to surgery and at follow-up visits at ∼1, 3, 6, and 12 months after surgery. Because POAG is a risk factor for SI-OHT, patients or suspects with glaucoma were excluded for enrollment in the study. The Indianapolis-2 replication cohort enrolled patients with FECD at the same clinical practice, and participants were treated the same way as the Indianapolis-1 cohort. The other 3 replication cohorts were enrolled at practices in Florida; the United States; and Chennai, India.

Maximum change in IOP from baseline was used as the QT for SNP association analysis for the Indianapolis cohorts and the Florida cohort. In addition to calculation of a QT that utilized the full 12 months of IOP data, the Indianapolis cohorts were stratified for calculation of a second QT that utilized IOP data collected over the first 3 months only. This provided for an analysis prior to any interventions and avoided the start of the steroid taper that began at around the 4-month time point.

Intraocular pressure data collection for the Florida-1 cohort was less organized than the Indianapolis cohorts, thus only a 12mQT could be used. Participants in the Chennai cohorts were grouped as cases and controls (steroid responders or nonresponders) based on their deltaIOPmax at the 6-month time point.

### SNP Association Analysis for the Indianapolis-1 Discovery Cohort

**Figure 2:**
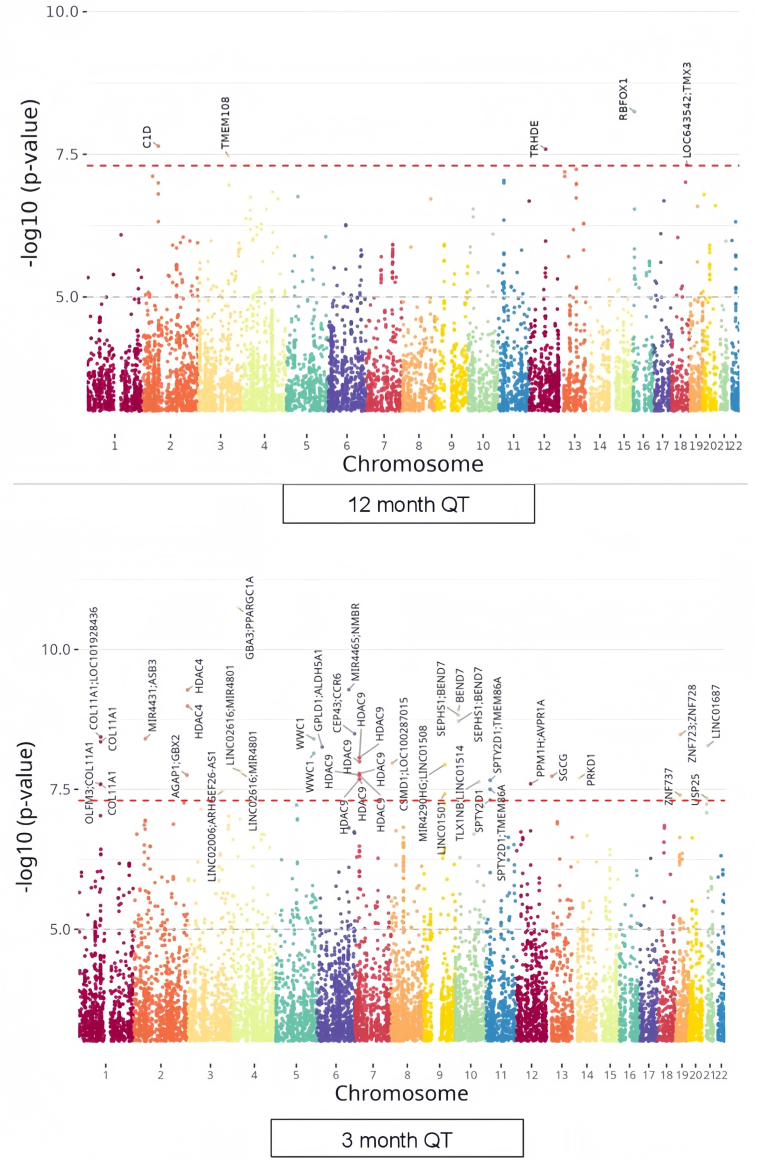
Manhattan plots for the Indianapolis-1 discovery GWASs. The x-axis is the position on each chromosome and the y-axis is the –log10 *P* value from the GWAS for each SNP. The red dashed line demarcates the threshold for genome-wide significance [-log10(5E-08); *P* = 5.0E-08]. The risk locus for each SNP of genome-wide significance is defined by the nearest genes to the SNP, as delineated on the plots. The QT used for each GWAS is indicated. GWAS = genome-wide association study; QT = quantitative trait; SNP = single nucleotide polymorphism.

Manhattan plots for SNP association analyses of the Indianapolis-1 discovery cohort using the 12-month and 3mQT are shown in Figure 2. Quantile–quantile plots with the calculated genomic control factors are shown in Figure S2 (available at www.ophthalmologyscience.org). Single nucleotide polymorphisms that attained the suggestive threshold of *P* < 5E-06 for the 12- and 3mQT analyses, ordered by *P* value, are listed in Table S1 and Table S2 (available at www.ophthalmologyscience.org). Single nucleotide polymorphism lists from the 12- and 3mQT analyses were merged in Table S3 (available at www.ophthalmologyscience.org), then sorted by chromosomal position to reveal individual risk loci that clustered multiple SNPs. Risk locus overlap between the 2 QTs is tabulated in Table S4 (available at www.ophthalmologyscience.org).

Table 1 summarizes all results. Using the 12mQT, 5 SNPs were identified clustered at 5 different risk loci that exceeded the threshold for genome-wide significance (*P* < 5E-08). Using the 3mQT, 41 SNPs were identified at 24 different risk loci that exceeded the threshold for genome-wide significance. Together, a total of 46 SNPs at 29 different risk loci attained genome-wide significance, and a total of 623 SNPs clustered at 323 risk loci attained suggestive significance (*P* < 5E-06). Most SNPs are rare or of low frequency and inversely correlated with effect size, which was overall quite large. Almost all SNPs exhibit positive effects on IOP. The exceptions are (1) the 3 suggestive SNPs associated with risk locus *GRM7;LOC101927394*, (2) 12 of the 41 suggestive SNPs associated with risk locus *RP1*, (3) 1 of the 4 suggestive SNPs associated with risk locus *LINC02303;LINC00871*, and (4) the suggestive SNP associated with risk locus *GAB4;CECR7*. These SNPs exhibit a protective effect on IOP. Only 6 SNPs are located within exonic DNA, all of suggestive significance, 4 within protein-coding genes—*LRP4, ACKR2, RP1, MUC6, ARVCF*—and 1 within a noncoding RNA gene—*FER1L4*. This is consistent with the notion that most of the discovered SNPs have regulatory effects on gene expression. Fourteen risk loci clustering SNPs overlapping between the QTs were identified. Risk locus *LINC02015;LINC01014* and risk locus *STAG1* duplicated the same suggestive SNPs, but with different *P* values. The others clustered SNPs identified in each QT that were different.

**Table 1:** Genome-Wide Association Study Summary Findings

Group/Subgroup	N SNPs *P* < 5E-08 (Genome-Wide)	N SNPs *P* < 5E-06 (Suggestive)	N risk Loci Clustering SNPs of *P* < 5E-08 (Genome-Wide)	N risk Loci Clustering SNPs *P* < 5E-06 (Suggestive)
12-month QT	5	215	5	104
3-month QT	41	412	24	232
Overlap	0	4	0	13
Merged	46	623	29	323

12-month QT				
SNP frequency	CAF	Mean effect size in mmHg	N SNPs	% Total

Common	>0.05	2.62	21	10.66%
Low frequency	0.01 to 0.05	5.45	43	21.82%
Rare	<0.01	12.18	151	67.51%
			215	

3-month QT				
SNP frequency	CAF	Mean effect size in mmHg (range)	N SNPs	% Total

Common	>0.05	2.25	47	11.44%
Low frequency	0.01 to 0.05	7.67	68	16.50%
Rare	<0.01	17.30	297	72.09%
			412	

CAF = coded allele frequency; MAF = minor allele frequency; QT = quantitative trait; SNP = single nucleotide polymorphism.

Table 2 (a stratified version of Table S3) compiles information about the top SNPs and the suggestive SNPs that clustered with them at the 29 top risk loci. All the SNPs are rare (85%) or of low frequency (15%) with large effect sizes. Two risk loci overlap between the 2 QTs. *HDAC4;HDAC4-AS1;LOC150935* clusters 1 SNP of suggestive significance identified using the 12mQT, and 2 different SNPs of genome-wide significance identified using the3mQT. *LINC00621:SGCG* clusters 2 SNPs of suggestive significance identified using the 12mQT, and 1 SNP of genome-wide significance identified using the 3mQT. In neither case are the SNPs identified in the 12 month analysis in linkage disequilibrium (LD) with the SNPs identified in the 3-month analysis.

**Table 2:** The 29 Risk Loci Clustering Single Nucleotide Polymorphisms of Genome-Wide Significance

Risk Locus Count	QT	rsID	LD with Index SNP (r^2^)	Chr	Position GRCh38	*P* Value	Ref Allele	Effect Allele	Effect Size (mmHg)	Minor Allele Count	CAF	Annotation	Risk Locus
1	3M	rs140420703	0.00	1	102337928	1.91E-06	T	G	16.49	5	0.0048	Intergenic	*OLFM3;COL11A1*
	3M	rs563167766	0.00	1	102400809	4.73E-06	G	A	19.42	3	0.0024	Intergenic	*OLFM3;COL11A1*
	3M	rs77180278	0.00	1	102496326	9.34E-08	T	C	7.34	24	0.0280	Intergenic	*OLFM3;COL11A1*
	**3M**	**rs112351653**	**0.62**	**1**	**102754804**	**2.55E-08**	**T**	**C**	**7.42**	**24**	**0.0285**	**Intergenic**	***OLFM3;COL11A1***
	3M	rs180926150	0.00	1	102760770	3.77E-06	C	T	19.64	3	0.0024	**Intergenic**	***OLFM3;COL11A1***
	**3M**	**rs114413507**	**Index**	**1**	**102953612**	**2.57E-08**	**T**	**C**	**7.42**	**24**	**0.0285**	**Intronic**	***COL11A1***
	**3M**	**rs116672066**	**0.73**	**1**	**103007360**	**4.46E-09**	**G**	**A**	**8.05**	**23**	**0.0273**	**Intronic**	***COL11A1***
	**3M**	**rs111928960**	**0.63**	**1**	**103168079**	**3.65E-09**	**G**	**A**	**8.60**	**22**	**0.0285**	**Intergenic**	***COL11A1;LOC101928436***
	3M	rs113221952	0.00	1	103288418	9.17E-07	A	G	7.92	19	0.0213	Intergenic	*COL11A1;LOC101928436*
2	3M	rs190193113	0.00	2	52858640	1.13E-07	G	A	19.95	3	0.0036	Intergenic	*MIR4431;ASB3*
	**3M**	**rs187520610**	*Index*	**2**	**53132903**	**3.93E-09**	**G**	**A**	**18.80**	**4**	**0.0048**	**Intergenic**	***MIR4431;ASB3***
	3M	rs149421869	0.00	2	53256291	2.05E-06	G	T	11.74	3	0.0083	Intergenic	*MIR4431;ASB3*
3	12M	rs191298981	0.00	2	67882914	1.57E-07	C	T	15.63	3	0.0046	Intergenic	*LINC01812;C1D*
	12M	rs113537164	0.62	2	68007798	1.00E-07	A	C	13.62	4	0.0046	Intergenic	*LINC01812;C1D*
	12M	rs191823270	0.62	2	68025878	4.79E-07	T	C	14.15	3	0.0034	Intergenic	*LINC01812;C1D*
	**12M**	**rs113154814**	**Index**	**2**	**68062365**	**2.27E-08**	**T**	**C**	**12.00**	**5**	**0.0057**	**Intronic**	***C1D***
4	**3M**	**rs147559909**	**N/A**	**2**	**236142879**	**1.75E-08**	**T**	**C**	**17.08**	**5**	**0.0086**	**Intergenic**	***AGAP1;GBX2***
5	**3M**	**rs181217257**	**Index**	**2**	**239067423**	**5.29E-10**	**C**	**T**	**21.57**	**4**	**0.0048**	**Intronic**	***HDAC4***
	**3M**	**rs188076929**	**0.0**	**2**	**239072023**	**1.03E-09**	**T**	**C**	**19.98**	**4**	**0.0048**	**Intronic**	***HDAC4***
	12M	rs115690445	0.0	2	239682619	1.13E-06	A	C	9.44	7	0.0080	Intergenic	*HDAC4-AS1;LOC150935*
6	**12M**	**rs114280794**	**N/A**	**2**	**133123359**	**3.40E-08**	**G**	**A**	**6.39**	**14**	**0.0159**	**Intronic**	***TMEM108***
7	**3M**	**rs16823323**	**N/A**	**3**	**153939413**	**3.35E-08**	**G**	**A**	**9.48**	**14**	**0.0185**	**Intergenic**	***LINC02006;ARHGEF26-AS***
8	3M	rs113751774	0.0	3	23427231	5.01E-07	C	T	14.91	7	0.0071	Intergenic	*GBA3;PPARGC1A*
	**3M**	**rs113063005**	**Index**	**4**	**23507444**	**1.79E-11**	**T**	**C**	**21.21**	**6**	**0.0039**	**Intergenic**	***GBA3;PPARGC1A***
9	**3M**	**rs77141817**	**Index**	**4**	**37052137**	**1.52E-08**	**T**	**C**	**20.61**	**3**	**0.0014**	**Intergenic**	***LINC02616; MIR4801***
	**3M**	**rs190822761**	**0.00**	**4**	**37097734**	**1.61E-08**	**G**	**T**	**20.59**	**3**	**0.0014**	**Intergenic**	***LINC02616; MIR4801***
10	**3M**	**rs545428520**	**Index**	**5**	**168394261**	**3.96E-09**	**T**	**C**	**21.57**	**4**	**0.0013**	**Intronic**	***WWC1***
	**3M**	**rs528404963**	**0.00**	**5**	**168425020**	**7.17E-09**	**T**	**C**	**21.14**	**4**	**0.0036**	**Intronic**	***WWC1***
11	3M	rs150586237	0.00	6	24491120	5.51E-09	C	T	22.05	3	0.0024	Intergenic	*GPLD1;ALDH5A1*
12	3M	rs146048121	0.00	6	141877818	3.24E-06	G	A	20.02	3	0.0024	Intergenic	*MIR4465;NMBR*
	**3M**	**rs142106992**	**Index**	**6**	**141948748**	**5.23E-10**	**C**	**A**	**22.83**	**4**	**0.0036**	**Intergenic**	***MIR4465;NMBR***
	3M	rs72983831	0.00	6	141985963	7.83E-07	T	G	15.67	6	0.0059	Intergenic	*MIR4465;NMBR*
	3M	rs72986533	0.00	6	142290121	1.54E-07	T	C	13.281	8	0.0083	Intergenic	*VTA1;ADGRG6*
	3M	rs73586304	0.00	6	142518288	4.96E-06	C	T	18.24	3	0.0036	Intergenic	*ADGRG6;LOC153910*
13	**3M**	**rs148153037**	**Index**	**6**	**167501386**	**3.20E-09**	**G**	**A**	**14.34**	**8**	**0.0140**	**Intergenic**	***CEP43;CCR6***
	3M	rs184487573	0.75	6	167099983	2.92E-07	A	G	13.50	7	0.0071	Intergenic	*CEP43;CCR6*
14	3M	rs75689761	1.00	6	18366950	9.12E-07	C	T	11.85	7	0.0083	Intronic	*HDAC9*
	3M	rs77346868	1.00	6	18366976	1.14E-06	A	G	11.02	8	0.0095	Intronic	*HDAC9*
	3M	rs78225611	1.00	6	18367841	1.13E-06	A	C	11.03	8	0.0095	Intronic	*HDAC9*
	**3M**	**rs77300464**	**1.00**	**6**	**18369138**	**2.09E-08**	**A**	**G**	**14.67**	**6**	**0.0071**	**Intronic**	***HDAC9***
	3M	rs79602997	1.00	6	18370627	9.43E-07	G	A	11.86	7	0.0083	Intronic	*HDAC9*
	3M	rs75773869	1.00	6	18371222	9.36E-07	G	T	11.86	7	0.0083	Intronic	*HDAC9*
	**3M**	**rs75606013**	**1.00**	**6**	**18374990**	**2.11E-08**	**G**	**A**	**14.66**	**6**	**0.0071**	**Intronic**	***HDAC9***
	3M	rs61434999	1.00	6	18378728	9.22E-07	A	G	11.84	7	0.0083	Intronic	*HDAC9*
	3M	rs78907958	1.00	6	18385394	4.99E-07	T	G	12.09	7	0.0083	Intronic	*HDAC9*
	**3M**	**rs76526501**	**Index**	**6**	**18391487**	**8.58E-09**	**G**	**A**	**14.84**	**6**	**0.0071**	**Intronic**	***HDAC9***
	**3M**	**rs74455595**	**1.00**	**6**	**18392161**	**8.58E-09**	**A**	**G**	**14.84**	**6**	**0.0071**	**Intronic**	***HDAC9***
	3M	rs79182806	1.00	6	18394204	4.14E-07	T	C	12.10	7	0.00831	Intronic	*HDAC9*
	**3M**	**rs10279777**	**0.57**	**6**	**18401966**	**1.65E-08**	**G**	**A**	**12.87**	**8**	**0.0095**	**Intronic**	***HDAC9***
	3M	rs77867199	0.57	6	18402652	3.93E-07	G	T	10.82	9	0.0107	Intronic	*HDAC9*
	3M	rs80156375	0.73	6	18403592	3.27E-07	A	C	11.54	8	0.0095	Intronic	*HDAC9*
	**3M**	**rs17169602**	**0.64**	**6**	**18407118**	**1.78E-08**	**G**	**A**	**12.73**	**8**	**0.0095**	**Intronic**	***HDAC9***
	**3M**	**rs10486295**	**0.64**	**6**	**18407184**	**1.75E-08**	**G**	**A**	**12.76**	**8**	**0.0095**	**Intronic**	***HDAC9***
	**3M**	**rs75090694**	**0.73**	**6**	**18407813**	**1.01E-08**	**A**	**G**	**13.83**	**7**	**0.008**	**Intronic**	***HDAC9***
15	**3M**	**rs575473987**	**Index**	**8**	**5719972**	**1.07E-08**	**C**	**T**	**22.22**	**3**	**0.0011**	**Intergenic**	***CSMD1;LOC100287015***
16	**3M**	**rs545690161**	**0.00**	**9**	**90268417**	**1.14E-08**	**G**	**A**	**17.14**	**6**	**0.0052**	**Intergenic**	***MIR4290HG;LINC01508***
	3M	rs565682685	0.00	9	90460046	3.58E-07	T	C	18.94	4	0.0048	Intergenic	*LINC01508;LINC01501*
	**3M**	**rs183737367**	**Index**	**9**	**90567765**	**3.80E-08**	**T**	**C**	**23.35**	**3**	**0.0036**	**Intronic**	***LINC01501***
	3M	rs187213609	0.00	9	90653183	5.72E-08	C	T	23.20	3	0.0036	Intergenic	*DIRAS2;SYK*
17	**3M**	**rs184425183**	**0.66**	**9**	**13415520**	**1.92E-09**	**A**	**G**	**22.67**	**3**	**0.0036**	**Intergenic**	***SEPHS1;BEND7***
	**3M**	**rs184458518**	**0.66**	**9**	**13429195**	**1.48E-09**	**T**	**G**	**22.80**	**3**	**0.0036**	**Intergenic**	***SEPHS1;BEND7***
	**3M**	**rs117998251**	**Index**	**10**	**13455976**	**1.19E-09**	**C**	**T**	**22.8**	**3**	**0.0065**	**Intronic**	***BEND7***
18	**3M**	**rs117913371**	**Index**	**10**	**101158729**	**2.31E-08**	**G**	**A**	**7.87**	**21**	**0.0189**	**Intergenic**	***TLX1NB;LINC01514***
	3M	rs75334617	0.23	10	101196395	7.30E-07	G	A	7.87	32	0.03800		*LINC01514;LBX1*
19	**3M**	**rs151115079**	**0.75**	**11**	**18634194**	**4.89E-08**	**T**	**C**	**17.83**	**5**	**0.0048**	**Intronic**	***SPTY2D1***
	**3M**	**rs138414342**	**Index**	**11**	**18657851**	**3.16E-08**	**G**	**A**	**18.16**	**5**	**0.0048**	**Intergenic**	***SPTY2D1;TMEM86A***
	**3M**	**rs541653703**	**0.00**	**11**	**18680239**	**2.18E-08**	**G**	**A**	**18.32**	**4**	**0.0048**	**Intergenic**	***SPTY2D1;TMEM86A***
	3M	rs118093638	0.00	11	18696777	9.72E-07	C	T	14.27	5	0.0059	Intergenic	*SPTY2D1;TMEM86A*
20	3M	rs137880949	0.00	12	62912517	1.75E-07	T	C	21.61	3	0.0048	Intronic	*PPM1H*
	**3M**	**rs191053292**	**Index**	**12**	**63051500**	**2.51E-08**	**T**	**C**	**22.93**	**3**	**0.0036**	**Intergenic**	***PPM1H;AVPR1A***
	3M	rs182437250	0.00	12	63214686	7.12E-07	T	C	18.81	4	0.0048	Intergenic	*AVPR1A;DPY19L2*
21	**12M**	**rs111582203**	**0.00**	**12**	**72290549**	**2.57E-08**	**A**	**G**	**10.08**	**8**	**0.0080**	**Intronic**	***TRHDE***
	12M	rs112507626	Index	12	72316321	1.05E-06	G	A	5.24	19	0.0228	Intronic	*TRHDE*
	12M	rs112796175	0.92	12	72396569	3.11E-06	C	T	4.77	21	0.02506	Intronic	*TRHDE*
	12M	rs112856203	0.73	12	72465084	4.46E-06	T	C	4.77	21	0.02506	Intronic	*TRHDE*
22	12M	rs566724618	0.00	13	22889700	6.40E-08	C	T	15.17	3	0.0034	Intronic	*LINC00621*
	12M	rs147221953	0.00	13	22911583	7.71E-08	G	A	15.58	3	0.0034	Intronic	*LINC00621*
	**3M**	**rs139598422**	**Index**	**13**	**23312875**	**1.84E-08**	**A**	**G**	**20.49**	**4**	**0.0036**	**Intronic**	***SGCG***
23	**3M**	**rs74704551**	**N/A**	**14**	**29692681**	**1.98E-08**	**C**	**T**	**24.20**	**3**	**0.0006**	**Intronic**	***PRKD1***
24	12M	rs573087160	0.00	16	5796693	4.85E-06	G	T	13.85	3	0.0023	Intergenic	*MIR8065;RBFOX1*
	12M	rs80212581	0.00	16	6362966	1.44E-06	C	T	13.25	3	0.0034	Intronic	*RBFOX1*
	12M	rs140276610	0.00	16	6383734	2.02E-06	C	T	13.53	4	0.0034	Intronic	*RBFOX1*
	12M	rs113109648	0.00	16	6574271	2.89E-07	T	C	16.03	3	0.0023	Intronic	*RBFOX1*
	12M	rs140545577	0.00	16	6805089	3.15E-06	C	T	12.37	3	0.0034	Intronic	*RBFOX1*
	12M	rs138164904	Index	16	6808238	5.67E-09	C	T	15.51	3	0.0034	Intronic	*RBFOX1*
	12M	rs533356672	0.00	16	7052822	4.28E-06	A	G	13.61	3	0.0034	Intronic	*RBFOX1*
25	**12M**	**rs559152067**	**N/A**	**18**	**68362375**	**4.86E-08**	**G**	**A**	**17.31**	**3**	**0.0023**	**Intergenic**	***LOC643542;TMX3***
26	3M	rs560206697	N/A	19	20546292	4.02E-08	C	T	25.00	3	0.0014	Intronic	*ZNF737*
27	**3M**	**rs111285015**	**N/A**	**19**	**22940396**	**3.31E-09**	**G**	**A**	**27.33**	**3**	**0.0091**	**Intergenic**	***ZNF723;ZNF728***
28	3M	rs184785969	0.00	21	15799112	8.26E-08	C	A	19.87	3	0.0036	Intronic	*USP25*
	3M	rs117280553	0.00	21	15834844	5.80E-08	T	C	20.087	3	0.0036	Intronic	*USP25*
	**3M**	**rs79486609**	**0.00**	**21**	**15872687**	**4.53E-08**	**G**	**A**	**20.66**	**3**	**0.0036**	**Intronic**	***USP25***
29	3M	rs73227413	0.21	21	21764653	2.75E-06	G	A	5.96	28	0.0344	ncRNA intronic	*LINC01425*
	3M	rs75024143	Index	21	21784226	2.10E-06	G	T	9.81	12	0.0142	ncRNA intronic	*LINC01425*
	**3M**	**rs192134381**	**0.00**	**21**	**22078395**	**5.23E-09**	**T**	**C**	**21.79**	**3**	**0.0036**	**ncRNA intronic**	***LINC01687***

Single nucleotide polymorphisms of genome-wide and suggestive significance clustered at each risk locus are listed. Single nucleotide polymorphisms of genome-wide significance are bolded. The risk locus is identified with the nearest gene(s) upstream; downstream of the hit SNP. Linkage disequilibrium was determined by querying the Haploreg v.2 portal.

3M = 3 month; 12M = 12 month; CAF = coded allele frequency; Chr = chromosome; LD = linkage disequilibrium; N/A not applicable; QT = quantitative trait; Ref = reference; rsID = single nucleotide polymorphism identification number; SNP = single nucleotide polymorphism.

**Figure 3:**
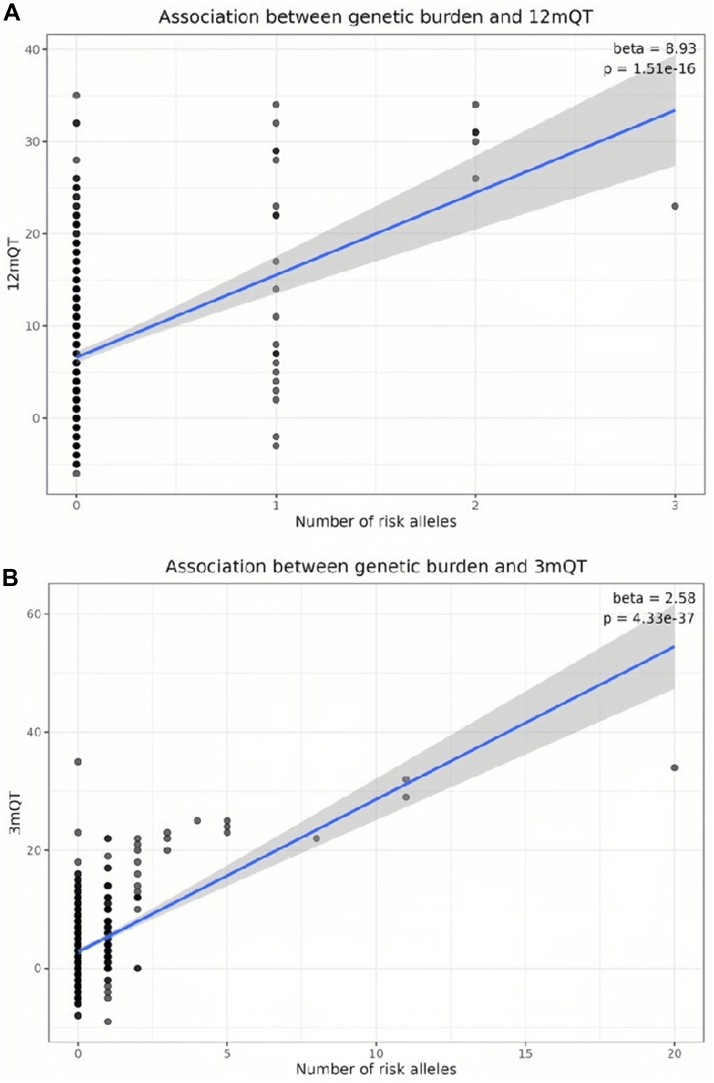
Association between genetic burden and change in intraocular pressure (IOP). Scatter plots show the relationship between the number of risk alleles (genetic burden) on the x-axis and deltaIOP in mmHg at (**A**) 3 months (3mQT) and (**B**) 12 months (12mQT) on the y-axis. The genetic burden score was calculated as the sum of risk alleles across independent genome-wide significant loci identified in the corresponding GWAS (5 loci for 12mQT and 31 loci for 3mQT; *P* < 5 × 10^–8^). Risk alleles were aligned to have consistent direction of effect. Solid lines represent fitted linear regression models with shaded areas indicating 95% confidence intervals. GWAS = genome-wide association study.

Genetic burden assessment for the SNPs of genome-wide significance is shown in Figure 3. For the 3-month outcome (3mQT), 31 independent loci with *P* < 5 × 10^–8^ were identified, and for the 12-month outcome (12mQT), 5 independent loci were identified. For the 3-month outcome (3mQT), an increasing burden of genome-wide significant risk variants was associated with a substantial increase in deltaIOPmax (β = 2.58, *P* = 4.33 × 10^–37^). A similar pattern was observed for the 12-month outcome (12mQT), where the burden of risk alleles remained significantly associated with deltaIOPmax (β = 8.93, *P*=1.51 × 10^–16^), despite the smaller number of independent loci. These results demonstrate a strong and consistent association between the cumulative number of risk alleles and IOP (deltaIOPmax) changes.

### Target Gene Prioritization

While the closest gene to a regulatory SNP is a likely target of that SNP, the closest gene might not be expressed in the relevant tissue. Moreover, regulatory SNPs can affect expression of >1 gene.^53^ To prioritize target genes, we made the assumption that a gene must be expressed in tissues of the AOPs. We searched the 2 relevant human single-cell RNA sequencing data sets on the Spectacle portal^42^ to identify the closest AOP-expressed gene(s), both upstream and downstream of hit SNPs. Results are compiled in Table S5 (available at www.ophthalmologyscience.org). Notably, of the 6 exonic SNPs discovered, only 2—*ARVCF* and *LRP2*—are located in AOP-expressed genes, suggesting the other 4 have regulatory function.

To find additional SNP target gene candidates, we searched SNPs at each risk locus on the Adult GTEx portal^46^ to determine colocalization with expression or splicing QT loci (eQTLs/sQTLs). The GTEx Project compiles QTLs from up to 54 nondiseased tissues across nearly 1000 deceased individuals; all are nonocular tissues, but many contain relevant cell types. After identifying e/sQTLs, we then searched their target genes on Spectacle to determine their AOP expression. Results are compiled in Table S6 (available at www.ophthalmologyscience.org).

Table S7 (available at www.ophthalmologyscience.org) compiles the 441 genes identified in the prioritization analyses. In some cases, eQTL/sQTL target genes were the same as genes already prioritized by expression in the AOPs (e.g., *COL11A1* and *TRDHE*), providing cross-validation.

### Validation of Prioritized Target Genes

To evaluate biological relevance of our prioritized target genes, we compared our gene list to lists derived from GC-regulated gene expression profiling studies, and we also utilized bioinformatics tools.

First, we compared our genes to a list of genes differentially regulated by GCs in 2 cultured primary human TM cell strains, as published by a member of our team.^54^ As tabulated in Table S8 (available at www.ophthalmologyscience.org), 33% (144/441) matched to prioritized target genes.

We also compared our prioritized target gene list to the lists of genes differentially-regulated by GCs in 3 different paired eye studies,^9^, ^10^, ^11^ one from members of our team.^9^ The paired eye gene lists are shown in Table S9 (available at www.ophthalmologyscience.org). We found matches to 31 of our prioritized target genes, as shown in Table S10 (available at www.ophthalmologyscience.org). Of the 31 genes, 24 were identified in steroid responders and 8 were identified in nonresponders (with 1 overlap). This includes prioritized target genes from 3 risk loci clustering SNPs of genome-wide significance: *ARHGEF26* (upregulated in nonresponders), *BEND7* (downregulated in responders), and *COL11A1* (downregulated in responders). Matching genes are tabulated with GC-regulated genes in TM cells in Table S8 and listed in Table S9 alongside the full paired eye gene lists.

To identify prioritized target genes that can be regulated directly by the activated GR, we turned to the NIH DAVID Bioinformatics functional annotation clustering tool,^55^ selecting the UCSC_TFBS (University of Santa Cruz Transcription Factor Binding Sites) option. This option identifies TF binding sites and their target genes based on data from Encyclopedia of DNA Elements (ENCODE), Blueprint, and Roadmap.^56^ The analysis was run at low stringency to reveal interactions with the GR. As summarized in Table S11 (available at www.ophthalmologyscience.org), 54% (239/441) of our prioritized target genes clustered with the GR with a high enrichment score (13.18) and high statistical significance after adjustment for multiple comparisons (1.2E-07). Matches with the prioritized target gene list are tabulated in Table S8 alongside the other GC-regulated genes.

We performed a similar DAVID analysis for the list of human paired eye study genes (Table S9) from the study published by members of our team.^9^ A much lower enrichment score (3.42) was obtained that was not significant after adjustment for multiple comparisons, as shown in Table S11. This might be explained by gene expression artifacts due to culturing of the TM cells.

Combining all results provides evidence for GC regulation of 71% (313/441) of our prioritized target genes (Table S8).

In our final analysis, we sought to identify common upstream regulators of our prioritized target genes that might validate their expression in ocular tissues. To do this, we ran the list through the DAVID plus UCSC_TF analysis as above, but this time at moderate stringency. Results are shown in Table S11. Three clusters were identified for our prioritized target gene list. The top cluster comprises 3 TFs involved in eye development: Lhx3,^57^ Hlf,^58^ and Chx10.^59^ A total of 63% (252/402 DAVID IDs) of our prioritized target genes were identified as targets of ≥1 of these 3 TFs. The cluster received a very high enrichment score (22.32) and the *P* values for each TF, after adjustment for multiple comparisons, were of high statistical significance (3.40E-26, 9.07E-22, 2.15E-17).

For comparison, we performed the same analysis using the steroid responder gene list from the human paired eye study, also shown in Table S11. This returned 3 clusters. The top cluster comprises 2 of the 3 eye development TFs identified using our prioritized target gene list: Lhx3^57^ and Chx10.^59^ A third and fourth TF involved in eye development, Sp8^60^ and Cart1,^61^ completed the cluster. Similar to the GR analysis, the enrichment score (1.46) and the adjusted *P* values (1.91E-03, 1.05E+01, 2.68E+01, 9.67E+01) were much less significant than for the GWAS prioritized target gene list. Again, this might be explained by gene expression artifacts due to culturing of the TM cells.

### Summary of Top Risk Loci Validation and Colocalization with Other OHT Phenotypes

Table 3 lists the top prioritized target genes for the 29 risk loci clustering SNPs of genome-wide significance. The genes from the 12-month analysis are (in alphabetical order): *C1D, RBFOX1, TMEM108, TMX3, TRHDE.* The genes from the 3 month analysis are: *AGAP1, ARHGEF26, ASB3, BEND7, CCR6, COL11A1, CSMD1, DIRAS2, GPLD1, HDAC4, HDAC9, KAZALD1, NCAM2, PGCKA1(C4orf19), PPARGC1A, PPM1H, PRKD1, SGCG, SPTY2D1, USP25, VTA1, WWC1, ZNF737*, and *ZNF728*. Validating evidence is summarized. Analyses described earlier provided evidence for GC regulation of 79% (23/29) of these genes. To identify any additional GC-regulated genes, we searched all SNPs clustered at the top 29 risk loci on RegulomeDB^48^ and HaploReg v4.2.^49^ Primary sources include the Encyclopedia of DNA Elements project, Roadmap Epigenomics, Gene Expression Omnibus eQTL studies, the GWAS catalog, evolutionary conservation and various published studies focused on TF binding (chromatin immunoprecipitation-sequencing), DNAse hypersensitivity, and chromatin states. The applications collect, analyze, and integrate these data sets to characterize and score the regulatory potential of noncoding variants. The results summarized in Table S12 (available at www.ophthalmologyscience.org) raised the GC-regulated gene total to 86% (25/29).

**Table 3:** Validation of the Top 29 Prioritized Target Genes

QT	Risk Locus	Closest AOP-Expressed Gene	Evidence for GC Regulation	Transcriptional Response in Paired Eye Studies	Other Validation	Genetic Association with Other OHT Phenotypes and Endophenotypes	Other Association with OHT Phenotypes
3M	*OLFM3;COL11A1;LOC101928436*	*COL11A1*	TM cells Paired eye Chromatin	Downregulated in steroid responders	sQTL Upstream TFs	POAG, PACG, PEXS, IOP, Stickler	
3M	*MIR4431; ASB3*	*ASB3*			Upstream TFs		
12M	*LINC01812;C1D*	*C1D*			Upstream TFs		
3M	*AGAP1;GBX2*	*AGAP1*	In silico Chromatin		Upstream TFs		
3M	*HDAC4*	*HDAC4*	TM cells In silico Chromatin		Upstream TFs		
12M	*TMEM108*	*TMEM108*	In silico		Upstream TFs	POAG-AF	
3M	*LINC02006;ARHGEF26-AS*	*ARHGEF26*	TM cells Paired eye In silico	Upregulated in steroid nonresponders	Upstream TFs		Knockdown prevented SI-OHT in mice
3M	*GBA3;PPARGC1A*	*PPARGC1A*	TM cells In silico		Upstream TFs		
3M	*LINC02616;MIR4801*	*PGCKA1 (C4orf19)*	In silico		Upstream TFs		
3M	*WWC1*	*WWC1*	TM cells In silico		Upstream TFs	POAG, POAG-AF	
3M	*GPLD1;ALDH5A1*	*GPLD1*	In silico Chromatin		Upstream TFs		
3M	*MIR4465;NMBR;VTA1;ADGRG6;LOC153910*	*VTA1*	TM cells		Upstream TFs		
3M	*CEP43;CCR6*	*CCR6*	Chromatin				
3M	*HDAC9*	*HDAC9*	In silico Chromatin		Upstream TFs		
3M	*CSMD1;LOC100287015*	*CSMD1*	In silico		Upstream TFs	POAG, POAG-AF IOP in rats	
3M	*MIR4290HG;LINC01508; LINC01501;DIRAS2;SYK*	*DIRAS2*			Upstream TFs		
3M	*BEND7*	*BEND7*	TM cells Paired eye In silico	Downregulated in steroid responders	Upstream TFs	POAG, POAG-AF	
3M	*TLX1NB;LINC01514; LINC01514;LBX1*	*KAZALD1*	TM cells In silico		Upstream TFs		POAG in proteome-wide association study
3M	*SPTY2D1;TMEM86A*	*SPTY2D1*	TM cells Chromatin		Upstream TFs		
3M	*PPM1H;AVPR1A*	*PPM1H*	TM cells In silico Chromatin		Upstream TFs	POAG, POAG-AF	
12M	*TRHDE*	*TRHDE*	In silico Chromatin		Upstream TFs		
3M	*LINC00621;SGCG*	*SGCG*	TM cells In silico		Upstream TFs	CCT	
3M	*PRKD1*	*PRKD1*	TM cells In silico		Upstream TFs		
12M	*MIR8065;RBFOX1*	*RBFOX1*	TM cells Paired eye In silico	Downregulated in steroid nonresponders	Upstream TFs	POAG, PEXS POAG-AF	
12M	*LOC643542;TMX3*	*TMX3*	In silico				
3M	*ZNF737*	*ZNF737*					
3M	*ZNF723;ZNF728*	*ZNF728*	TM cells				
3M	*USP25*	*USP25*	TM cells In silico		Upstream TFs		
3M	*LINC01425;LINC01687*	*NCAM2*	In silico		Upstream TFs	POAG-AF	

The closest AOP-expressed gene was determined by identifying genes located up or downstream from the hit SNP using the UCSC Genome Browser, then determining expression of each via Spectacle.

Paired eye: responder or nonresponder in 1 of the 3 paired eye studies discussed in the text. Glucocorticoid-regulated TM cells: differentially expressed in the TM cell culture study discussed in the text. Glucocorticoid-regulated in silico: identified in the DAVID Bioinformatics/UCSC_TFBS low stringency analysis discussed in the text. Glucocorticoid-regulated chromatin: identified in chromatin analysis using Regulome DB; upstream TFs: clusters with eye development TFs identified in the DAVID Bioinformatics/UCSC_TFBS moderate stringency analysis.

3M = 3 month; 12M = 12 month; AOP = aqueous outflow pathway; CCT = central corneal thickness; DAVID = Database for Annotation, Visualization, and Integrated Discovery; GC = glucocorticoid; IOP = intraocular pressure; OHT = ocular hypertension; PACG = primary angle-closure glaucoma; PEXS = pseudoexfoliation syndrome; POAG = primary open-angle glaucoma; POAG-AF = primary open-angle glaucoma-African ancestry; ; QT = quantitative trait; SI-OHT = steroid-induced ocular hypertension; SNP = single nucleotide polymorphism; sQTL = splicing quantitative trait locus; Stickler = type II Stickler syndrome; TF = transcription factor; TFBS = Transcription Factor Binding Sites; TM = trabecular meshwork; UCSC = University of Santa Cruz.

Colocalization with risk loci discovered for other OHT phenotypes and their endophenotypes was investigated by cross-referencing with published reports,^14^, ^15^, ^16^, ^17^, ^18^^,^^35^, ^36^, ^37^^,^^62^ including a GWAS for IOP in rats.^38^ Of our top 29 SI-OHT risk loci, 31% (9/29) colocalize with risk loci for ≥1 other OHT phenotypes. Particularly striking in this regard is the *OLFM3;COL11A1;LOC101928436* risk locus, which colocalizes with 5 other OHT phenotypes (endophenotypes): POAG, PACG, pseudoexfoliation syndrome, IOP, and type II Stickler syndrome. Notably, none of the associated SI-OHT SNPs at a given risk locus match with the SNPs of common frequency linked to the colocalizing phenotypes, nor are they in LD.

The prioritized target genes for the risk loci colocalizing with other OHT phenotypes (endophenotypes) are: *BEND7, COL11A1, CSMD1, NCAM2, PPM1H, RBFOX1, SGCG, TMEM108, WWC1*. Literature searching identified 2 additional prioritized target genes that associate with OHT phenotypes. *ARHGEF26* encodes a rho-guanine nucleotide exchange factor, knockdown of which ameliorated GC-induced myofibroblast transdifferentiation in TM cells and prevented the development of SI-OHT in mice.^63^ The protein encoded by *KAZALD1* was recently discovered as one of 26 plasma proteins having potential causal associations with high-tension POAG in a proteome-wide association study.^64^

### SNP Association Analysis for the Indianapolis-2 Replication Cohort

The Manhattan plots and Q-Q plots for SNP association analysis of the Indianapolis-2 discovery cohort using the 12-month and 3mQTs are show in Figure S3 (available at www.ophthalmologyscience.org).

A total of 60 SNPs attained the suggestive threshold of *P* < 5E-06 with the 12mQT and a total of 102 SNPs attained the suggestive threshold of *P* < 5E-06 with the 3mQT. The complete list of SNPs ordered by *P* value is provided in Table S13 (available at www.ophthalmologyscience.org) and Table S14 (available at www.ophthalmologyscience.org). None of the SNPs attained genome-level significance; however, in the 12 month analysis, intronic SNP rs74649788 at risk locus *L3MBTL4*, came very close (*P* = 5.5E-08).

The 12-month and 3mQT results were sorted by chromosomal position as shown in Table S15 (available at www.ophthalmologyscience.org) and Table S16 (available at www.ophthalmologyscience.org) to reveal a total of 81 individual risk loci that cluster top SNPs, with risk locus *PCBD1;UNC5B* found in both analyses. Of these 81 risk loci, 16% (13/81) colocalize with risk loci identified in the Indianapolis-1 discovery cohort. However, the SNPs associated with these loci are all new; they do not replicate SNPs found in the Indianapolis-1 discovery cohort, nor are they in LD.

### Independent Replication

Replication of individual SNPs discovered in the Indianapolis-1 cohort that exceeded a threshold of *P* = 5.0E-06 was tested using the 4 replication cohorts. Meta-analysis was performed to combine *P* values for the 3mQT of the Indianapolis-1 cohort with the 3mQT of the Indianapolis-2 cohort and the Florida-1 cohort, because these cohorts were sufficiently homogeneous.

None of the SNPs discovered in the Indianapolis-1 cohort attained a *P*-value for replicative significance in the Indianapolis-2 cohort, as shown in Table S17 (available at www.ophthalmologyscience.org). However, in the meta-analysis shown in Table S22 (available at www.ophthalmologyscience.org) *P*-values were improved for 5 SNPs, with concordant direction of effect. These SNPs are listed in Table 4. They are: rs189890455 associated with risk locus *FOXN2;PPP1R21* (*P* = 4.20E-07 to *P* = 3.61E-07), rs145439370 associated with risk locus *LIPC;ADAM10* (*P* = 9.02E-07 to *P* = 7.93E-07), rs1877768 associated with risk locus *ATXN1* (*P* = 2.78E-06 to *P* = 9.49E-07), rs139062456 associated with risk locus *DLC1* (*P* = 3.88E-06 to *P* = 1.01E-06), and rs187384541 associated with risk locus *DLGAP2* (*P* = 3.15E-06 to *P* = 1.55E-06).

**Table 4:** Replication Meta-Analysis: *P* Values of SNPs Discovered in the Indianapolis-1 Cohort Combined with *P* Values of SNPs Found in the Indianapolis-2 and Florida-1 Cohorts

QT	rsID	Chr	Position GRCh38	REF	ALT	CAF	Indianapolis-1 *P* Value	Indianapolis-1 Effect Size (mmHg)	Indianapolis-2 *P* Value	Meta-Analysis *P* Value	Risk Locus
3M	rs189890455	2	48428258	C	T	0.006	4.18E-07	15.15	2.10E-01	3.61E-07	*FOXN2;PPP1R21*
3M	rs1877768	6	16534692	C	T	0.018	2.78E-06	8.29	1.13E-01	9.49E-07	*ATXN1*
3M	rs139062456	8	13394482	C	T	0.018	3.89E-06	7.84	9.02E-02	1.01E-06	*DLC1*
3M	rs187384541	8	1684075	A	G	0.036	3.15E-06	6.89	1.56E-01	1.55E-06	*DLGAP2*
3M	rs145439370	15	58587566	T	C	0.031	9.02E-07	6.89	2.26E-01	7.93E-07	*LIPC;ADAM10*

Shown are SNPs with improved *P* values in the meta-analysis combining the 3-month QT outcomes of the Indianapolis-1 cohort with the Indianapolis-2 and the Florida-1 cohort outcomes. In these 5 cases, the Indianapolis-1 cohort SNP was found only in the Indianapolis-2 cohort; it was absent from the Florida-1 cohort. The effect sizes for the Indianapolis-1 cohort SNPs are all positive. The effect sizes for the Indianapolis-2 cohort SNPs are not listed, because they are negligible, but all are positive.

3M = 3 month; CAF = coded allele frequency; Chr = chromosome; Position GRCh38 = position of single nucleotide polymorphism on the GRCh38 reference panel; QT = quantitative trait; REF and ALT = reference allele and alternate (effect) allele; rsID = reference single nucleotide polymorphism cluster ID; SNP = single nucleotide polymorphism.

None of the SNPs discovered in the 12- or 3-month analysis of the Indianapolis-1 discovery cohort were replicated in the Chennai-1 cohort, as shown in Table S18 (available at www.ophthalmologyscience.org) and Table S19 (available at www.ophthalmologyscience.org). This was also the case for the 12-month analysis in the Chennai-2 cohort, as shown in Table S20 (available at www.ophthalmologyscience.org). However, 3 SNPs from the 3-month analysis were replicated in the Chennai-2 cohort with *P* values that exceeded the adjusted threshold for significance, and with concordant direction of effect, as shown in Table S21 (available at www.ophthalmologyscience.org). These SNPs are listed in Table 5. Replicated SNPs rs7822082 (*P* = 8.66E-06, adjusted *P* = 3.55E-04) and rs1561297 (*P* = 7.41E-05; adjusted *P* = 3.04E-03) are located in an intron of *RP1*. Both are common in frequency. Replicated SNP rs193153124 (*P* = 3.56E-04, adjusted *P* = 1.46E-02) is located intergenically, upstream of *AGTR1*. This SNP is rare. Meta-analysis to combine the *P* values from the 2 cohorts could not be performed because of the different outcomes; the Indianapolis-1 GWAS utilized a cohort study design, while the Chennai-2 cohort utilized a case-control study design.

**Table 5:** Replication Analysis: SNPs Discovered in the Indianapolis-1 Cohort Replicated in the Chennai-2 Cohort

QT	rsID	Chr	Position GRCh38	REF	ALT	CAF	Indianapolis-1 *P* Value	Indianapolis-1 Effect Size (mmHg)	Chennai-2 *P* Value	Chennai-2 Odds Ratio	Risk Locus
3M	rs193153124	3	148612923	A	G	0.005	5.30E-07	14.37	3.56E-04	N/A	*LINC02046;AGTR1*
3M	rs1561297	8	54765978	A	C	0.31	3.36E-07	2.28	7.41E-05	5.18	*RP1*
3M	rs7822082	8	54777660	T	C	0.14	1.50E-07	2.36	8.66E-06	6.11	*RP1*

Shown are SNPs from the Indianapolis-1 cohort that attained replicative significance in the Chennai-2 cohort. A cohort study design was used for analysis of the Indianapolis-1 cohort (QT, 3M) while the Chennai-2 cohort was analyzed using a case-control study design (responders vs. nonresponders). In each case there is concordance of effect direction (all positive) but the outcomes are different; thus, meta-analysis could not be performed. The lack of an odds ratio for rs193153124 is because it is too rare; there were no individuals carrying the effect allele in the control group.

3M = 3 month; CAF = coded allele frequency; Chr = chromosome; Position GRCh38 = position of single nucleotide polymorphism on the GRCh38 reference panel; N/A = not applicable; QT = quantitative trait; REF and ALT = reference allele and alternate (effect) allele; rsID = reference single nucleotide polymorphism cluster ID; SNP = single nucleotide polymorphism.

Biological information about the prioritized target genes of the 3 replicated SNPs is compiled in Table 6. The prioritized target gene of rs193153124 is *AGTR1*, encoding angiotensin II receptor type 1, a cell surface receptor that regulates blood pressure, fluid-salt balance, and cardiovascular homeostasis. A literature report documents GC stimulation of *AGTR1* expression.^65^ Activation of AGTR1 leads to vasoconstriction and increases fluid retention, both of which can raise IOP.^66^ Single nucleotide polymorphisms in the gene's coding region have been linked to high-tension POAG.^67^ Overexpression of *AGTR1* has been implicated in the development of fibrosis in various tissues, including the heart, lungs, and kidneys.^68^

**Table 6:** Validation of Prioritized Target Genes of Replicated SNPs

Risk Locus	Closest AOP-Expressed Gene	AOP-Expressed e/sQTL Target Gene(s)	GC Regulation	Genetic Association with Other OHT Phenotypes	Gene Function
*LINC02046; AGTR1*	*AGTR1*		Literature	POAG	Angiotensin II receptor type 1 (AT1 Receptor), regulates blood pressure, fluid-salt balance, and cardiovascular homeostasis
*RP1*	*SOX17*		TM cells In silico		Transcription factor that acts downstream of Wnt and TGFB pathway signaling
*RP1*	*SOX17*	*XKR4*	TM cells	OHT	Plasma membrane phospholipid scramblase, mediates exposure of phosphatidylserine on the cell surface in apoptosis

The closest AOP-expressed gene was determined on the Spectacle portal.

TM cells: GC-regulated differentially expressed genes in the TM cell culture studies discussed in the text. In silico: GC-regulated genes identified in DAVID Bioinformatics, UCSC_TFBS analysis. Chromatin: identified as GC-regulated in chromatin analysis using Regulome DB.

AOP = aqueous outflow pathway; DAVID = Database for Annotation, Visualization, and Integrated Discovery; eQTL = expression quantitative trait locus; GC = glucocorticoid; OHT = ocular hypertension; POAG = primary open-angle glaucoma; SNP = single nucleotide polymorphism; sQTL = splicing quantitative trait locus; TFBS = Transcription Factor Binding Sites; TM = trabecular meshwork; UCSC = University of Santa Cruz.

The prioritized target gene of SNPs rs7822082 and rs1561297 is *SOX17*, a TF that acts downstream of Wnt and TGFB signaling, 2 pathways linked to SI-OHT.^3^^,^^4^ In addition, rs1561297 colocalizes with an eQTL that targets *XKR4* in the tibial nerve. *XKR4* is AOP-expressed, providing a second prioritized target gene. *XKR4* encodes a plasma membrane phospholipid scramblase that mediates exposure of phosphatidylserine on the cell surface in apoptosis. The gene is regulated by GCs in TM cells and has been linked to ocular hypotension.^69^

### Cell-Type Localized Expression of Top Prioritized Target Genes and Prioritized Target Genes Associated with Replicated SNPs

Relative AOP cell type expression of the prioritized target genes for the 29 risk loci clustering SNPs of genome-wide significance are shown in Figure S4 (available at www.ophthalmologyscience.org). This figure compiles Spectacle heat maps depicting relative gene expression in the 19 cell types of the van Zyl single-cell RNA sequencing dataset. The same information for genes associated with replicated SNPs is shown in Figure S5 (available at www.ophthalmologyscience.org). Notably, TM beam cells or JCT cells that make up the tissues where fibrosis is localized in SI-OHT are not among the top expressing cell types for most of these genes. *AGTR1*, *DIRAS2, NCAM2, PPM1H,* and *RBFOX1* are not expressed in JCT cells and *CCR6*, *PGCKA1 (C4orf19)*, and *SOX17* are not expressed in JCT or TM cells. This suggests that gene dysregulation in other cell types besides those comprising the site of GC-induced fibrosis contributes to SI-OHT pathophysiology.

### Functional Annotation of SNPs Targeting COL11A1

We found the broad colocalization of risk locus *OLFM3;COL11A1;LOC101928436* with 5 other OHT phenotypes to be quite intriguing. While we did not replicate the low-frequency and rare SNPs that clustered at this locus, replication was achieved for the common SNPs associated with 4 of the other 5 OHT phenotypes.^14^, ^15^, ^16^, ^17^, ^18^ The fifth is a rare mutation causing type II Stickler Syndrome, inherited in families.^62^ Both *OLFM3* and *COL11A1* are expressed in the AOPs, but *COL11A1* is nearest to the SNPs. Significantly, 6 of the SI-OHT SNPs identified at the *OLFM3;COL11A1;LOC101928436* risk locus colocalize with sQTLs that affect *COL11A1* RNA splicing (Table S6). Moreover, in one of the paired eye studies discussed earlier,^11^ expression of *COL11A1* was downregulated by GCs in TM cells cultured from steroid responders (Table S11). Thus, multiple lines of evidence support the biological significance of SNPs affecting *COL11A1* expression across OHT phenotypes, providing the rationale for further analysis.

**Figure 4:**
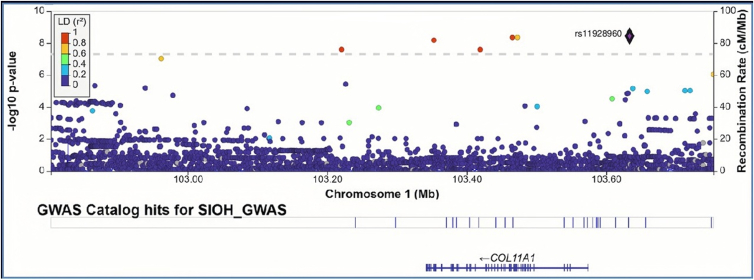
LocusZoom plot for the *COL11A1* risk locus. The plot covers the region containing the nine associated SNPs of genome-wide and suggestive significance listed in Table 5 (GRCh37). The dotted line indicates the threshold for genome-wide significance (the index SNP rs111928960 [black diamond] has the lowest *P* value [3.65E-09]). The plot shows the 4 other GWAS hit SNPs of genome-wide significance, plus 1 additional SNP (gold dot) above the threshold, but with an R^2^ below the GWAS cutoff (R^2^ > 0.7). Three GWAS hit SNPs of suggestive significance are indicated. GWAS = genome-wide association study; LD = linkage disequilibrium; SIOH = steroid-induced ocular hypertension; SNP = single nucleotide polymorphism.

The *OLFM3;COL11A1;LOC101928436* risk locus clusters a total of 9 SI-OHT SNPs identified in our GWAS, 4 of genome-wide significance and 5 of suggestive significance. Six of these SNPs (including all of genome-wide significance) are of low frequency, while 3 are rare. A LocusZoom plot covering the genomic region is shown in Figure 4. Using HaploReg v4.2,^49^ we found that index SNP rs111928960, which is the top low frequency SNP, is in LD with low-frequency SNPs rs113221952 (R^2^ = 0.77), rs116672066 (R^2^ = 0.89), and rs114413507 (R^2^ = 0.63). In addition, low-frequency SNP rs114413507 is in weak LD with low-frequency SNP rs112351653 (R^2^ = 0.36). These 5 low-frequency SNPs form an LD peak. The sixth low-frequency SNP rs77180278 is in weak LD with rare SNP rs140420703 (R^2^ = 0.36), which is in perfect LD (R^2^ = 1) with rare SNP rs563167766. These SNPs form a second LD peak. The third rare SNP, rs180926150, is not in LD with any of the others and we could find no annotation for it.

To obtain additional information about how SNPs associated with SI-OHT and the other OHT phenotypes might affect *COL11A1* expression, we returned to the GTEx portal and we also accessed additional bioinformatics platforms. Findings are compiled in Table 7. Violin plots taken from the GTEx portal that depict SNP effects on *COL11A1* RNA expression and splicing are shown in Figure S6 (available at www.ophthalmologyscience.org). It should be noted that, while colocalizing QTLs were characterized in organs and tissues other than those of the AOPs, cell types overlap. For example, testes contain contractile smooth-muscle-like cells, blood vessels, lymphatic vessels, nerve fibers, and extracellular matrix-secreting fibroblasts analogous to cell types found in the AOPs. Because cells of the AOPs express *COL11A1*, colocalization of a SNP with a QTL is evidence of the potential of that SNP to modify expression of *COL11A1* in the AOPs.

**Table 7:** SNPs within and around the *COL11A1* Gene Associated with Different OHT Phenotypes

OHT Phenotype	Genomic Position (GRCh37) GRCh38	SNP ID	MAC	Effect Size, Direction (mmHg)	MAF ALFA	*P* Value	Location in *COL11A1*	Regulatory Role	Regulatory Process Affected	Cohort or Reference
SI-OHT	(102803484) 102337928	rs140420703	5	+16.49	0.0029	1.91E-06	Downstream	Disrupts GR binding motif	Transcription	Indianapolis-1
SI-OHT	(102866365) 102400809	rs563167766	3	+19.42	0.0013	4.73E-06	Downstream	Disrupts multiple TF binding motifs	Transcription	Indianapolis-1
SI-OHT	(102961882) 102496326	rs77180278	24	+7.34	0.0095	9.34E-08	Downstream	sQTL, negative effect	Intron 41 splicing	Indianapolis-1
SI-OHT	(103220360) 102754804	rs112351653	24	+7.42	0.0172	2.55E-08	Downstream	sQTL, negative effect	Intron 41 splicing	Indianapolis-1
SI-OHT	(103226326) 102760770	rs180926150	3	+19.42	0.0006	3.77E-06	Downstream			Indianapolis-1
POAG IOP	(103341016) 102875460	rs4907985			0.4532	2.182E-09	Downstream	Disrupts TF binding motifs eQTL, neg/pos effects sQTL, positive effect	Transcription Intron 41 splicing	Han et al ^17^ Lo Faro et al ^18^
END TRANSCRIPTION, genomic position:102,876,467, exon 67 end										
PACG	(103354138) 102888582	rs1676486			0.1880	1.10E-05	Exon 60 of 67	Missense mutation	Decreased mRNA stability	Vithana et al ^15^
PACG	(103379918) 102914362	rs3753841			0.3974	9.22E-10	Exon 52 of 67	Missense mutation sQTL, positive effect	Decreased protein stability Intron 62 slicing	Vithana et al ^15^
POAG	(103385373) 102919817	rs993471			0.4575	7.76E-10	Intron 49 of 66	Disrupts multiple TF binding sites (ChIP-seq) sQTL, positive effect	Transcription Intron 62 slicing	Gharahkhani et al ^16^
Genomic position:102 946 956, intron 41 end										
SI-OHT	(103419168) 102953612	rs114413507	24	+7.42	0.017561	2.57E-08	Intron 41 of 66	sQTL, negative effect	Intron 41 splicing	Indianapolis-1
PEXS	(103422730) 102957174	rs3102053			0.283179	3.00E-04	Intron 41 of 66	sQTL, neg/pos effects	Intron 41 splicing (neg); intron 31 splicing (pos)	Krumbiegel et al ^14^
Stickler	(103427527) 102961971	None known			rare	Mendelian	Intron 41 of 66	Splice site mutation	Intron 41 splicing	Kohmoto et al ^48^
Genomic position:102 961 866, intron 41 start										
SI-OHT	(103472916) 103007360	rs116672066	23	+8.05	0.01704	4.46E-09	Intron 15 of 66	sQTL, negative effect	Intron 41 splicing	Indianapolis-1
START TRANSCRIPTION, genomic position:103,108 872, exon 1 start										
SI-OHT	(103633635) 103168079	rs111928960	22	+8.60	0.016995	3.65E-09	Upstream	sQTL, negative effect	Intron 41 splicing	Indianapolis-1
SI-OHT	(103753974) 103288418	rs113221952	19	+7.92	0.016305	9.17E-07	Upstream	sQTL, negative effect	Intron 41 splicing	Indianapolis-1

The table compiles GWAS and SNP frequency information with information obtained by searching the GTEx portal, the NIH Genome-Wide Repository of Associations Between SNPs and Phenotypes (GRASP) portal, the RegulomeDB portal, and the HaploReg v4.2 portal, by extracting genomic position localization and sequence information from the Santa Cruz Genome Browser and the European Molecular Biology Laboratory Nucleotide Sequence Database, and by accessing disease phenotype information sourced on MedLine Plus. The *COL11A1* gene is located on chromosome 1 and the major transcript has 66 introns and 67 exons.

Linkage disequilibrium (LD) relationships.

SI-OHT1. Index SNP rs111928960, which is the top low frequency SNP, is in LD with low frequency SNPs rs113221952 (R^2^ = 0.77), rs116672066 (R^2^ = 0.89) and rs114413507 (R^2^ = 0.63). In addition, low frequency SNP rs114413507 is in weak LD with low frequency SNP rs112351653 (R^2^ = 0.36).2. Low frequency SNP rs77180278 is in weak LD with rare SNP rs140420703 (R^2^ = 0.27), which is in perfect LD (R^2^ = 1) with rare SNP rs563167766.3. The third rare SNP, rs180926150, is not in LD with any of the others.

POAG/PACG.

POAG common SNP rs993471 is in perfect LD (R^2^ = 1) with PACG common SNP rs3753841.

ChIP-seq = chromatin immunoprecipitation-sequencing; eQTL = expression quantitative trait locus; GR = glucocorticoid receptor; GTEx = Genotype Tissue Expression; GWAS = GWAS = genome-wide association study; IOP = intraocular pressure; MAC = minor allele count; MAF = minor allele frequency; MAF ALFA = minor allele frequency (allele frequency aggregator dataset from the National Center for Biotechnology Information dbGAP); neg = negative; NIH = National Institutes of Health; OHT = ocular hypertension;PACG = primary angle-closure glaucoma; PEXS = pseudoexfoliation syndrome; POAG = primary open-angle glaucoma; pos = positive; SIOH = steroid-induced ocular hypertension; SI-OHT = steroid-induced ocular hypertension; SNP = single nucleotide polymorphism; sQTL = splicing quantitative trait locus; Stickler = type II Stickler syndrome; TF = transcription factor.

Each of the low frequency SNPs in the first LD peak colocalize with sQTLs identified in testes that inhibit *COL11A1* intron 41 splicing (Tables 4 and 5). Failure to remove intron 41 could affect protein function or cause nonsense-mediated decay of the mRNA.^70^ In the second LD peak, low-frequency SNP rs77180278 also colocalizes with an sQTL identified in testes tissue that inhibits *COL11A1* intron 41 splicing. Rare SNP rs140420703 colocalizes with a GR binding motif and rs563167766 colocalizes with multiple TF binding motifs. Single nucleotide polymorphisms in these binding motifs could affect gene expression. These findings align with the paired eye study observation that *COL11A1* expression is reduced by GCs in steroid responders, as noted earlier (Table S9).

Continuing with the *COL11A1* intron 41 theme, common SNP rs3102053 associated with pseudoexfoliation syndrome colocalizes with an sQTL identified in testes tissue that inhibits *COL11A1* intron 41 splicing, but also can positively affect intron 31 splicing. The rare type II Stickler syndrome mutation localizes to a splice acceptor site of intron 41 that prevents intron 41 splicing.^48^

Conversely, common SNP rs4907985, associated with POAG and IOP, colocalizes with an sQTL that positively affects splicing of intron 41 in testes. It also colocalizes with an eQTL identified in testis with a negative effect on *COL11A1* transcription, and in tibial nerve with a positive effect. It further colocalizes with multiple TF binding motifs.

Common SNP rs993471 associated with POAG and common SNP rs3753841 associated with PACG are in perfect LD (R^2^ = 1). Both colocalize with an sQTL identified in testes tissue that positively affects *COL11A1* intron 62 splicing. Single nucleotide polymorphisms rs3753841 is also a missense coding mutation (Pro1323Leu) that disrupts the Gly-Pro-Hyp triplet essential for correct folding of the collagen protomer.^71^

Common PACG SNP rs1676486 is a third a missense mutation; however, functional studies indicate that the effect of the risk allele is to reduce stability of the COL11A1 mRNA, leading to a lower amount of the protein being produced.^72^ This SNP is also associated with lumbar disk herniation.^72^

### Functional Annotation of Prioritized Target Genes

While the *P* = 5E-08 threshold for statistical significance is necessary to provide the stringency to limit false positives, a lesser threshold is acceptable for follow-ups, for which the cost of a modestly greater set of false-positives is low.^73^ Thus, we used our full prioritized target gene list to conduct pathway enrichment analysis, pairing the NIH DAVID Bioinformatics functional annotation clustering tool^55^ with the Reactome database.^52^ The results are shown in Table S23 (available at www.ophthalmologyscience.org). None of the clusters met statistical significance after adjustment for multiple comparisons. However, such clusters can still hold biological meaning, particularly if they possess high biological relevance. To provide validation for this analysis, we compared it to the same analysis performed using the human paired eye study previously published by members of our team,^9^ as well as the published bovine paired eye study.^11^

The top scored cluster (1.46) contained the Reactome event "Muscle Contraction” and sub-event “Cardiac Conduction”. All genes included in this sub-event were part of phase 1 of the 5 conduction phases that occur in the sinoatrial node or “pacemaker” of the heart. Included genes encode specific potassium channels and their modifiers: *KCND3, KCNIP1, KCNIP3, KCNIP4.* In the human paired eye study analysis, the Reactome event "Muscle Contraction" received an even stronger DAVID score (2.48) for genes regulated in steroid responders, and this event was statistically significant after adjustment for multiple comparisons (2.83E-02) in the sub-analysis of genes down-regulated in steroid responders. The top-scoring sub-event was again “Cardiac Conduction”, but this time all genes included in the subevent were part of phase 0 of the 5 cardiac conduction phases, providing an intriguing complement.

**Figure 5:**
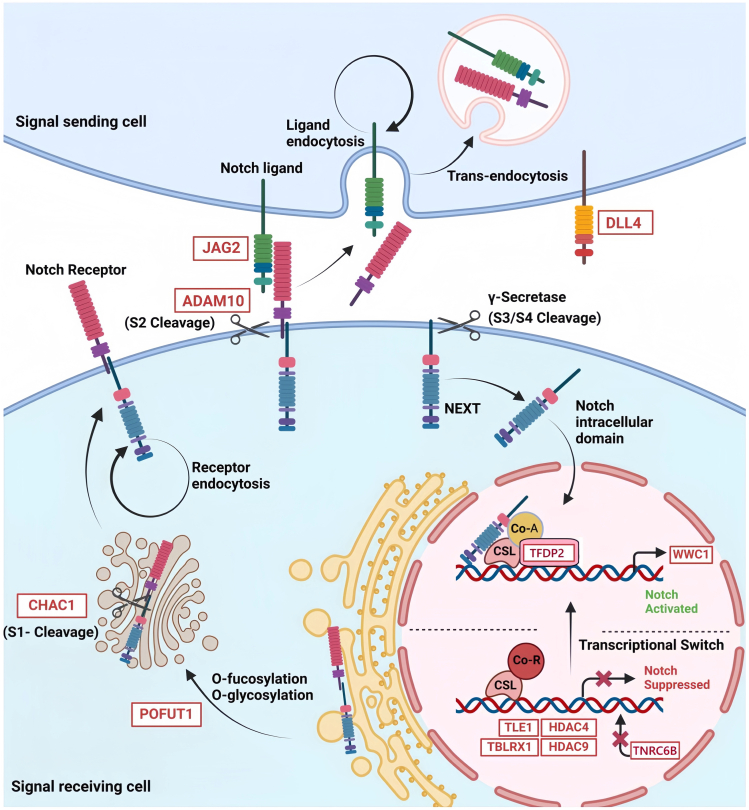
Genome-wide association study prioritized target genes that cluster with the pathway “signaling by NOTCH.” A schematic overview of the canonical Notch signaling pathway is shown, with white boxes indicating prioritized target genes of SNPs identified in this study. *TNRC6B* acts within the RISC, and studies indicate it plays a role in the pathway for pre-NOTCH transcription and translation. Notch receptors are synthesized as a single polypeptide that is O-fucosylated and glycosylated in the endoplasmic reticulum. *POFUT1* encodes a Golgi-localized fucosyltransferase that performs the first step in glycosylation of the Notch EGF-like domains. Loss of POFUT1 severely inhibits Notch signaling.^87^^,^^88^ The Notch protein then moves to the trans-Golgi network, where it is cleaved by a Furin-like convertase(s) (S1), forming a heterodimer. *CHAC1* encodes an inhibitor of this cleavage.^89^ The heterodimeric receptor is then transported to the cell membrane, where it binds with the ligand expressed in the neighboring cell. *DLL4* and *JAG2* encode members of the Notch ligand families Delta and Serrate, respectively. Receptor–ligand interaction initiates the second cleavage (S2) within the extracellular domain. *ADAM10* encodes 1 of only 2 proteinases known to effect this second cleavage step (the other is ADAM17).^89^ After S2 cleavage, the NEXT, is “transendocytosed” by the neighboring ligand-expressing cell, in a process that appears to be controlled by E3 ubiquitin ligases. This is followed by a third and fourth cleavage (S3, S4) that occurs within the transmembrane domain and is mediated by a multiprotein γ-secretase. The NICD is then released and translocates into the nucleus and binds to the transcription factor CSL. This interaction leads to transcriptional activation by displacement of Co-R, including proteins encoded by *TLE1, HDAC4, HDAC9, and TBLRX1*. Transcription factors encoded by *TFDP2* and *E2F3* bind to the promoter regions of Notch target genes, thereby enhancing their transcription.^90^ The protein encoded by *WWC1* (also known as Kibra) is a notch target gene and a key component of Hippo/YAP signaling.^91^ Co-R = co-repressors; CSL, CBF-1, Suppressor of Hairless, Lag-2; NEXT = Notch extracellular truncated fragment; NICD = intracellular domain of Notch; RISC = RNA-induced silencing complex; SNP = single nucleotide polymorphism; YAP = yes-associated transcriptional regulator.

The second scored cluster (1.42) contained the Reactome subevents “Signal Transduction: Signaling by NOTCH” and “Disease: diseases of signal transduction by growth factor receptors and second messengers”; the former subevent was also scored for the bovine paired eye study analysis (0.36) and the latter subevent was also scored for the human paired eye study analyses (0.46 and 0.36). Table S29 (available at www.ophthalmologyscience.org) lists some of the top-scoring subordinate events. As diagrammed in Figure 5, GWAS prioritized target genes that clustered with “Signal Transduction: Signaling by NOTCH” encompass the full range of Notch expression, maturation, and gene regulation and includes top prioritized target genes *WWC1*, *HDAC4*, and *HDAC9*. This finding has high biological relevance, as Notch signaling cross-talks with GR-mediated signaling in cell types examined. Notch signaling must be inhibited for GR-dependent gene activation to occur, and pathology can result if Notch signaling is too strongly inhibited in organ systems examined.^74^

The third scored cluster (1.41) contained the Reactome subevent “Metabolism: Metabolism of Carbohydrates.” This subevent (0.82), as well as the Reactome subevent: “Disease: Diseases associated with glycosaminoglycan (GAG) metabolism” (0.81) was also scored for the bovine paired eye study analysis. Table S23 lists some of the top-scoring subordinate events, all of which involve metabolism of GAGs. This finding has high biological relevance as GCs enhance the synthesis and deposition of GAGs within the JCT as part of the fibrotic response.^75^

## Discussion

The pharmacogenomics study reported here follows up on 2 pilot studies published by our teams that identified significant genomic variants associated with steroid response in the eye for the first time.^21^^,^^22^ For this new study, we built a substantially larger and more uniform discovery cohort by partnering with a single surgical practice, and we used our existing cohorts for replication. Our findings substantially expand on previous results, with 46 SNPs of genome-wide significance (*P* < 5E-08) clustered at 29 different risk loci in a total of 623 SNPs of suggestive significance (*P* < 5E-06) clustered at 323 risk loci. Similar to complex disease,^14^, ^15^, ^16^, ^17^, ^18^^,^^35^, ^36^, ^37^ essentially all the SI-OHT SNPs we discovered are located in noncoding DNA, where they are predicted to exert regulatory effects on expression of nearby genes. However, in striking contrast to the predominantly common variants of small effect size associated with complex disease,^14^, ^15^, ^16^, ^17^, ^18^^,^^35^, ^36^, ^37^ most of the SNPs that we discovered here are rare or of low frequency with large effect sizes. In disease, larger effect sizes are usually observed only when variants fall within protein-coding regions of a gene that alter the encoded protein's structure/function.^76^ These variants are typically under selective pressure of elimination; however, drug response variants may not be under such pressure.^19^ Moreover, regulatory variants affecting DNA binding of a TF like the GR, the effector of GC drug response, can mirror the effect size of coding variants affecting the GR protein itself.^77^

The data collection methodology we employed for the Indianapolis discovery cohort made it possible to use an early 3 month time point for calculating the QT. This proved much more effective than the 12-month time point for SNP discovery. Our ability to isolate a “pure” phenotype using the 3mQT may explain our greater success using this analysis. Heterogeneity increased after 3 months due to intervention and tapering of the drug dose. Moreover, SNPs that contribute to steroid response at later time points might mask SNPs contributing to early steroid response. Unfortunately, we were unable to replicate any of these SNPs; all are rare or of low frequency, requiring much larger cohorts for replication than we assembled for this study. For this reason, we cannot exclude the possibility of false-positives. That having been said, results of the genetic burden analysis conducted here support our top SNPs with high statistical significance. Moreover, validation of the prioritized target genes of these SNPs—by colocalization with eQTLs and sQTLs, comparison to gene profiling study results, and annotation analyses—provides additional support.

Importantly, we found colocalization between 31% of the top 29 SI-OHT risk loci, and risk loci for a variety of other OHT phenotypes or their endophenotypes. In addition, we found literature evidence for causal association of 2 additional risk loci. These colocalizations are not particularly surprising in the case of high-tension POAG, because its pathophysiological features are very similar to SI-OHT.^3^^,^^4^ In fact, POAG is a risk factor for SI-OHT^3^^,^^4^ and for this reason, we excluded patients with glaucoma or glaucoma suspects from our discovery cohort. Consistent with this, in no case did we find individual SI-OHT SNPs to be shared or in LD with SNPs associated with POAG. This suggests that the pathogenetic triggers for SI-OHT and POAG differ, providing evidence that they are distinct diseases, the pathophysiologies of which converge on the same genes.

More surprising was risk locus colocalization with OHT phenotypes such as PACG and type II Stickler syndrome, which do not involve fibrosis. A striking example is the risk locus that clustered SNPs located within and around *COL11A1* which was shared among 5 of the OHT phenotypes. Bioinformatics analyses predicted that these SNPs would reduce *COL11A1* expression, splicing, and/or accumulation of a functional protein product. Single nucleotide polymorphisms associated with the other OHT phenotypes were different from those identified for SI-OHT, and they were not in LD, but they converged functionally with SI-OHT SNPs.

*COL11A1* encodes one of the 3 peptide chains of type XI collagen. While it has been 15 years since SNPs associated with this gene were first linked to OHT,^15^ its role in the disease process is still unknown. Spectacle datasets analyzed here reveal expression of the *COL11A1* mRNA in the human AOPs, and a very recent report documents the presence of Col11a1 protein in the irideocorneal angle of the DBA/2J mouse.^78^ Upregulated expression of the various collagen chains is a characteristic of fibrosis and is typically reported in GC-regulated gene expression profiling studies.^3^^,^^4^ However, in addition to the reduction in COL11A1 expression predicted by bioinformatics, *COL11A1* expression was specifically downregulated in steroid responders in a paired eye study evaluated here.^11^

Overall, our analysis of cell type-specific gene expression in the AOPs suggests that the pathophysiology of SI-OHT involves AOP cell types broadly, and thus might encompass other mechanisms besides fibrosis. A similar observation was recently made for genes involved in the pathophysiology of high-tension POAG.^79^^,^^80^ Type XI collagen is a regulatory form that coassembles with types I and II collagens to control fibril diameter and organization.^81^ A reduction in the protein could result in biomechanical alterations in the irideocorneal angle that contribute to an increase in IOP. Further investigation will be necessary to address this hypothesis.

The large list of prioritized target genes identified in this study provided sufficient numbers to conduct pathway enrichment analysis, providing the opportunity to uncover gene relationships that transcend information about individual genes. The top pathway identified was “muscle contraction.” This pathway was also scored for paired eye study differentially expressed genes analyzed here. A very recently published study using the gene expression profiling approach to study SI-OHT in mice also identified muscle contraction as a top pathway.^82^ The subevent we identified, “cardiac conduction,” clusters genes encoding a related group of potassium ion channels that serve as pacemakers in the heart. Their role in the AOPs has not been previously investigated to our knowledge and follow-up is needed.

The second pathway we identified was “signaling by NOTCH.” This finding has high biological relevance, as Notch signaling has been shown to cross-talk with GR-mediated signaling.^74^ In fact, Notch signaling must be inhibited for GR-dependent gene activation to occur.^74^ Consistent with this, the same recently published gene profiling study that identified muscle contraction as a pathway also found Notch pathway inhibition in mice treated with GCs.^82^ Notch pathway genes are overexpressed in cultured TM cells from individuals with high-tension POAG.^83^ A recent conference abstract reported that activation or knockdown of Notch signaling in cultured TM cells inhibits or enhances GC-induced fibrosis, respectively.^84^

The third pathway identified was “metabolism of carbohydrates,” with top-scoring subordinate events involving metabolism of GAGs. The relevance of GAGs to OHT has much prior experimental support. These highly glycosylated molecules, including hyaluronic acid, chondroitin sulfates, keratan sulfates, and heparan sulfates, are crucial components of the TM, contributing to the filtration barrier, and affecting resistance to aqueous outflow.^85^ Glucocorticoids enhance the synthesis and deposition of GAGs within the JCT as part of the fibrotic response.^75^ Single nucleotide polymorphisms that disrupt their normal metabolism would be expected to affect aqueous outflow.

While we did not replicate SNPs of genome-wide significance, *P* values were improved for 5 SNPs of suggestive significance in our meta-analysis and 3 SNPs of suggestive significance were replicated. The prioritized target gene of replicated rare SNP rs193153124 is *AGTR1*, encoding angiotensin II receptor type 1. Activation of AGTR1 leads to vasoconstriction and increases fluid retention, both of which can raise IOP,^66^ and has been implicated in fibrosis in the heart, lungs, and kidneys.^68^ Importantly, other SNPs associated with *AGTR1* have been linked to high-tension POAG.^67^ The prioritized target gene of replicated common SNPs rs7822082 and rs1561297 is *SOX17*, a TF that acts downstream of Wnt and TGFB signaling, 2 pathways that have been linked to SI-OHT.^3^^,^^4^ In addition, rs1561297 colocalizes with an eQTL that targets *XKR4,* also an AOP-expressed gene. *XKR4* encodes a plasma membrane phospholipid scramblase that mediates exposure of phosphatidylserine on the cell surface in apoptosis. The gene is regulated by GCs in TM cells and has been linked to OHT.^69^

### Limitations of the Study

Enrollment of large cohorts for SI-OHT has proven to be elusive,^3^ and our 2 previous pilot studies were conducted with very small cohorts.^21^^,^^22^ A strength of this study is the ∼sevenfold larger size of the discovery cohort, which was sufficiently powered to discover rare SNPs; however, it is still small by GWAS standards. Moreover, the sizes of replication cohorts that we assembled were insufficient for substantial replication for the largely rare SNPs we discovered.

The uniformity of the Indianapolis discovery cohort, including common ancestry, disease indication, and steroid treatment during the first 3 months postsurgery, as well as the rigorous attention to detail in data collection by the enrolling foundation was a strength of the study. However, heterogeneity was introduced into the analysis using the 12mQT because of intervention as well as the steroid taper. While the 12mQT analysis was adjusted for intervention as a dichotomous covariate, this might not have captured all the possible variability.

Many other factors introduced heterogeneity, likely limiting our ability to replicate our discovered SNPs. Adjustment for intervention could not be made for the Indianapolis-2 replication cohort, 12mQT analysis. Participants in the other 3 replication cohorts were treated with different GCs, at different doses, delivered in different ways, for different indications. The Chennai replication cohorts were of different ancestry than the Indianapolis cohorts and were treated with different GC therapeutics for different indications. The time points used for IOP measurement of the Florida replication cohort could not be fit into the regular time points used for the Indianapolis cohorts, so a 3mQT was not possible. The Chennai cohort utilized a 6-month cohort only.

We used imputed data for rare variants; however, we were extremely conservative and only SNPs with a high quality score were retained in the analysis. We prioritized SNP target genes based on their expression in single-cell RNA sequencing data sets on Spectacle. However, the depth of single cell data is limited, so it might miss some low-expressing genes. Such genes might be substantially upregulated in response to steroids. On the positive side, very few of the genes located closest to hit SNPs were identified as not expressed; therefore, this limitation would affect only a few of our determinations.

## Conclusions

Glucocorticoid use continues to be widespread in ophthalmology and is the mainstay of treatment for inflammatory eye diseases. Glucocorticoids are frequently used postsurgery, with the requirement for prolonged treatment.^3^^,^^4^ Intravitreal implants are increasingly being used for retinal vascular disease, also meaning prolonged steroid exposure.^86^ Such indications for GC use in the eye are increasing in prevalence in our aging society. Thus, SI-OHT represents a major clinical challenge. We believe the results of this study have clear implications for addressing these challenges in the near-term future. First, the ability to predict adverse response to GCs is a broadly attractive goal for delivery of personalized medicine. Most of the SNPs discovered here present with large effect size and our genetic burden analysis supports the idea that even a single SNP would place a patient at great risk for a significant adverse response to GC treatment in the eye. Second, the SNP target genes we have identified, once further validated, could serve as drug targets for the management of SI-OHT.

## Declaration of Generative AI and AI-Assisted Technologies in the Writing Process

During the preparation of this work the authors used Google AI in order to more quickly find relevant citations. After using this tool/service, the authors reviewed and edited the content as needed and take full responsibility for the content of the publication.

## Data Availability

Genotyping data for the Indianapolis-1 cohort are available on NCBI dbGaP, accession code phs004657.v1. Genotyping data for the Indianapolis-2 cohort are available on NCBI dbGaP, accession code phs000421.v1.p1. The differentially expressed gene lists from the human paired eye study are deposited in NCBI-SRA, BioProject PRJNA729873. Any additional information required to reanalyze the data reported in this paper is available upon reasonable request to the authors.
