# Supplementary material for: A Human Pharmacogenomics Approach Provides Insight Into the Pathogenesis and Pathophysiology of Steroid-Induced Ocular Hypertension
Source: Ophthalmol Sci. 2026 May 21;6(8):101239. doi: 10.1016/j.xops.2026.101239 (PMC13355179; doi:10.1016/j.xops.2026.101239)
Supplement: Supplemental Figures S1-S6 [file mmc1.pdf]

Supplemental Figure S1. Power Calculation.

Detectable effect sizes depend on the distribution of the MAF of the disease-causing SNP. Since this is not known in advance of a scan, we performed calculations for a range of MAFs. For each outcome, we assumed a 0.05 overall significance level (family-wise error rate = 0.05) and a GWAS significance level of 5×10^{-8} (point-wise error rate, α_p). We also assumed an allele dosage model, i.e. each SNP genotype to be coded as 0, 1, or 2 for the number of minor alleles. We show the minimum detectable effect sizes conditional on all the above assumptions, and assuming 80% power. The detectable effect sizes are expressed in terms of the standard deviation (SD, s). In other words, we wrote the linear model relating the standardized trait value ($z = Y/s$) to a causative SNP (G=0, 1 or 2 minor alleles) as $z = \beta_0 + \beta_1 G$. The detectable effect size β_1 represents the increase in mean Y (expressed as fractions of its corresponding standard deviation (SD)) per increase of one minor allele.

Detectable effect size (in standard deviation units) across a range of allele frequencies

Sample size = 400<br>Power = 80%	SNP minor allele frequency						
	0.0001	0.001	0.01	0.05	0.15	0.25	0.35
Detectable effect size at α_p=5e-8	22.25	7.04	2.24	1.02	.62	.51	.47
Detectable effect size at α_p=5e-7	20.74	6.56	2.09	.95	.58	.48	.44
Detectable effect size at α_p=5e-6	19.12	6.05	1.92	.88	.53	.44	.40
Detectable effect size at α_p=5e-5	17.32	5.48	1.74	.79	.48	.40	.36

Abbreviation: α_p, point-wise error rate.

Supplemental Figure S2. Q-Q plots for the Indianapolis-1 discovery cohort.

Quantile-quantile (Q-Q) plots showing the distribution of the observed P values from the logistic regression analysis for the GWAS scan against the expected distribution under the null hypothesis. The two quantitative traits (QTs) used are indicated. The x-axis of QQ-plot reports the expected −log10 (p-value) and the y-axis reports the observed −log10 (p -value). The genomic control parameters (λ), which are equal to 1, indicate that population stratification is addressed properly.

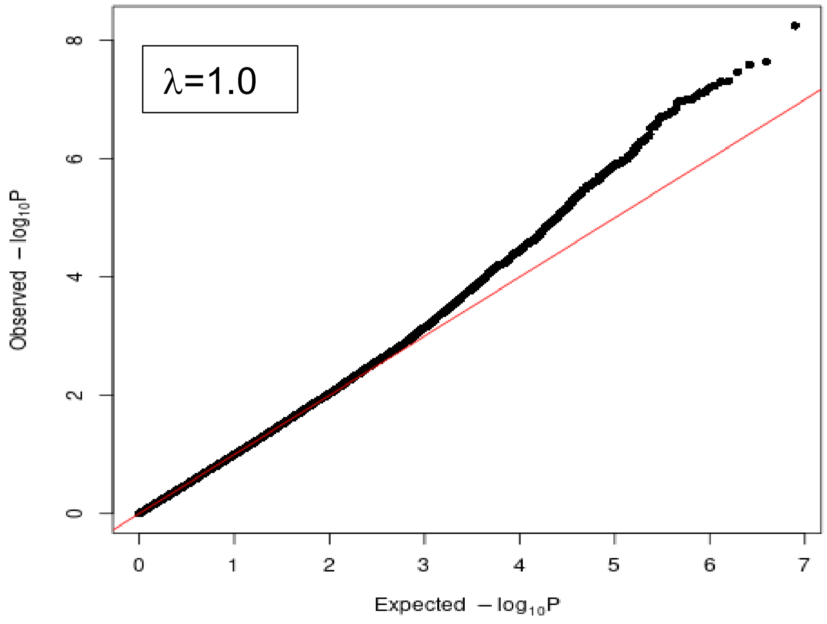

12 month QT

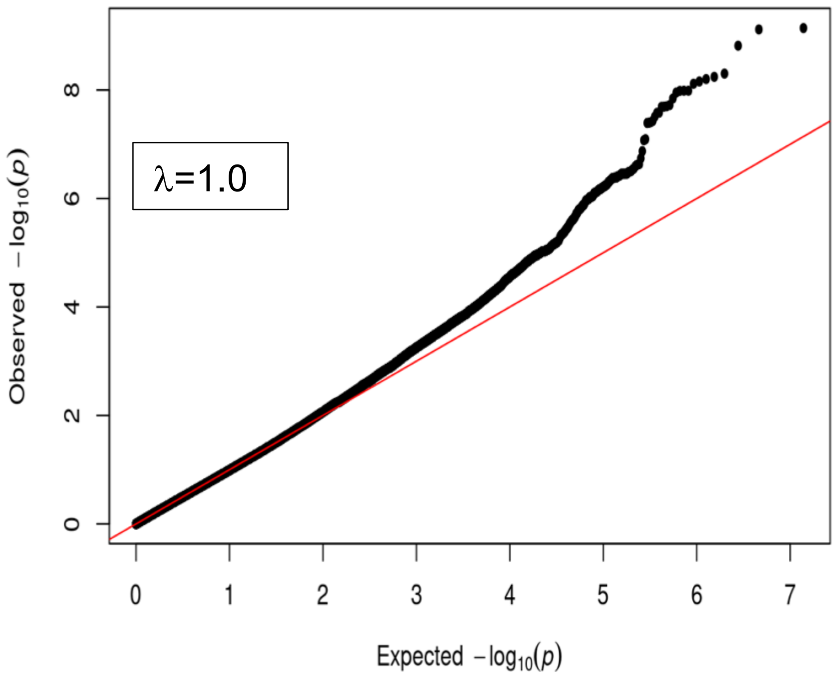

3 month QT

Supplemental Figure S3. Q-Q and Manhattan plots for the Indianapolis-2 replication cohort using the 12 month (12M) and 3 month (3M) quantitative traits. Quantile-quantile (Q-Q)-plot (left) and Manhattan plot (right) of the GWAS conducted for the Indianapolis-2 cohort with the quantitative trait (QT) as indicated. The x-axis of Q-Q-plot reports the expected −log10 (p-value) and the y-axis reports the observed −log10 (p -value). The x-axis of Manhattan plot reports chromosomes and coordinates within chromosomes. The y-axis reports the −log10 (p -value). The genomic control parameter (λ), which is close to 1 in both cases, indicates that population stratification is addressed properly.

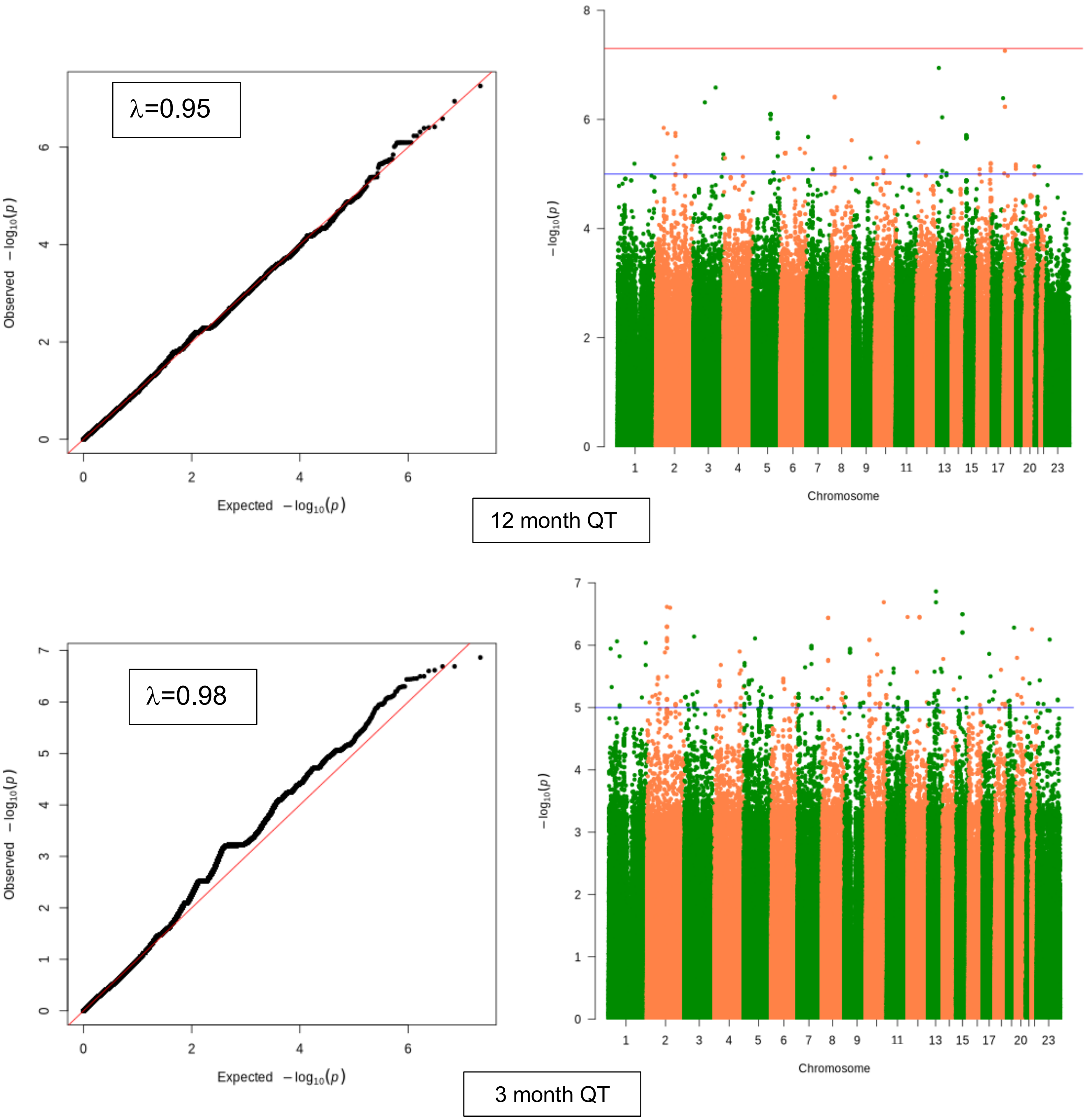

Supplemental Figure S4. Aqueous outflow pathway (AOP) cell type expression of prioritized target genes for the top 30 risk loci.

Gene expression heat map as viewed in the van Zyl human scRNA-seq dataset hosted on the Spectacle portal. Abbreviations and Acronyms: AOP: aqueous outflow pathway; JCT: juxta-canicular trabecular meshwork; MY: myelinating; NMY: non-myelinating; NKT: natural killer cell; ScEndo: Schlemm's canal endothelium.

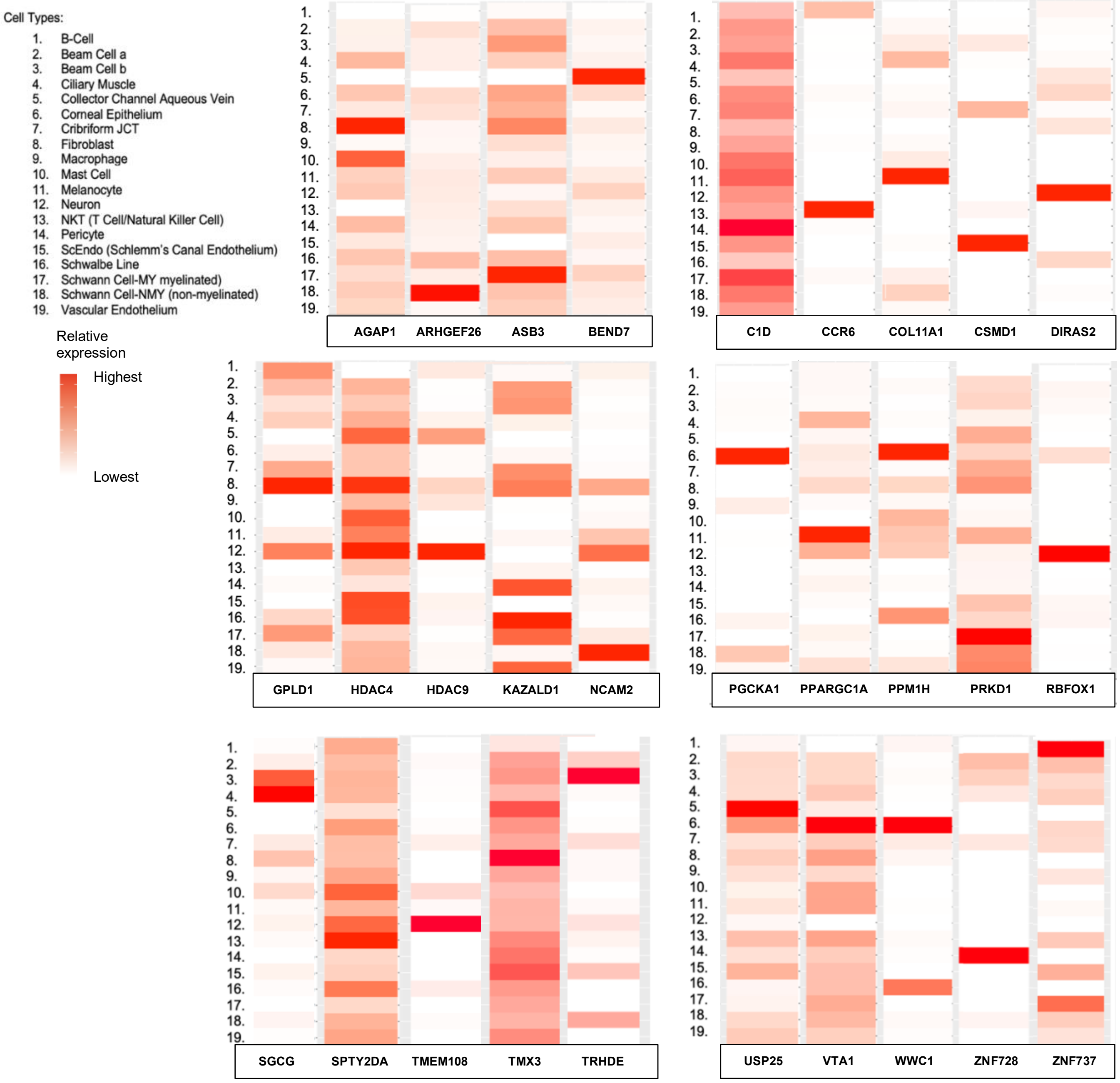

Supplemental Figure S5. Aqueous outflow pathway (AOP) cell type expression of prioritized target genes for replicated SNPs.

Gene expression heat map as viewed in the van Zyl human scRNA-seq dataset hosted on the Spectacle portal. Abbreviations and Acronyms: AOP: aqueous outflow pathway; JCT: juxta-canicular trabecular meshwork; MY: myelinating; NMY: non-myelinating; NKT: natural killer cell; ScEndo: Schlemm's canal endothelium.

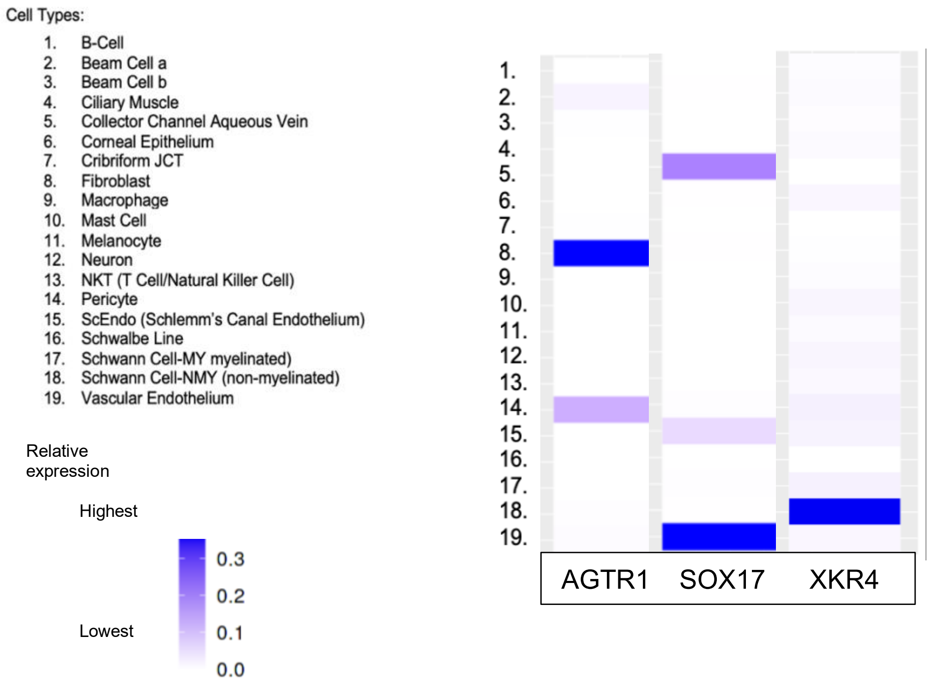

Supplemental Figure S6. Effects of sQTLs identified in the *COL11A1* gene associated with SIOH or other OHT phenotypes. Each of the indicated SNPs was searched on the GTEx portal. Shown are QTL violin plots depicting the effect of the minor allele on splicing (tissue type: testis).

COL11A1 gene: 66 introns and 67 exons

Positional Information from Genome Build GRCh38:

- Chr1:102,876,473-103,108,521 = 232,049 bp
- Chr1: 102,979,612-102,984,138 = 8,885 bp = alternative splicing, intron 31
- Chr1: 102,946,956-102,961,866 = 14,911 bp = intron 41
- Chr1: 102,888,311-102,886,807 = 3,497 bp = intron 62

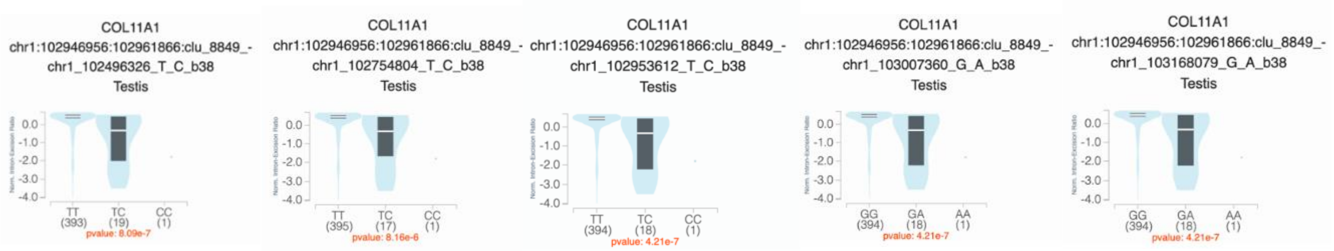

SI-OHT rs77180278<br>negative effect<br>intron 41 splicing	SI-OHT rs112351653<br>negative effect<br>intron 41 splicing	SI-OHT rs114413507<br>negative effect<br>intron 41 splicing	SI-OHT rs116672066<br>negative effect<br>intron 41 splicing	SI-OHT rs111928960<br>negative effect<br>intron 41 splicing

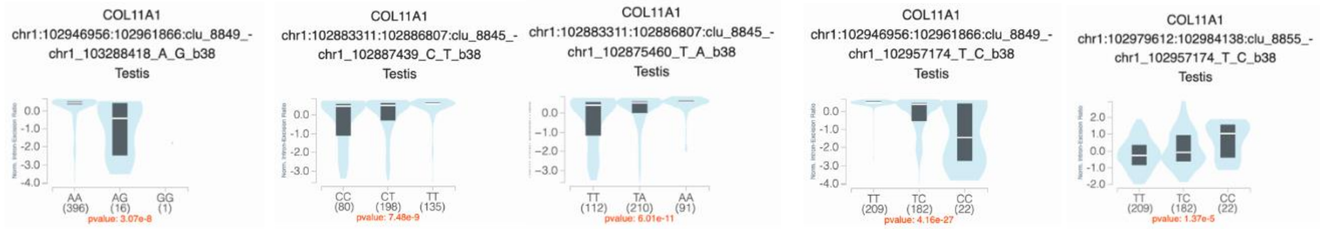

SI-OHT rs113221952<br>negative effect<br>intron 41 splicing	POAG rs993471<br>positive effect<br>intron 62 splicing	POAG rs993471<br>positive effect<br>intron 62 splicing	PEXS rs3102053<br>negative effect<br>intron 41 splicing	PEXS rs3102053<br>positive effect<br>intron 31 alternative splicing

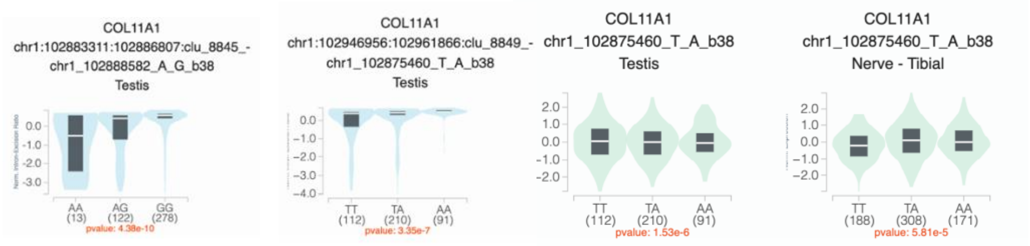

PACG rs1676486<br>positive effect<br>intron 62 splicing	POAG/IOP rs4907985<br>positive effect<br>intron 41 splicing	POAG/IOP rs4907985<br>negative effect<br>transcription	POAG/IOP rs4907985<br>positive effect<br>transcription
