# Supplementary material for: A Human Pharmacogenomics Approach Provides Insight Into the Pathogenesis and Pathophysiology of Steroid-Induced Ocular Hypertension
Source: Ophthalmol Sci. 2026 May 21;6(8):101239. doi: 10.1016/j.xops.2026.101239 (PMC13355179; doi:10.1016/j.xops.2026.101239)
Supplement: Supplemental Tables S1-S23 [file mmc2.pdf]

Supplemental Table S1. GWAS Results SNPs Indianapolis-1 Discovery Cohort
12 month (12M) quantitative trait

Notes

Ordered by SNP P-value 6 SNPs identified of genome-wide significance; 198 SNPs of suggestive significance; Total: 204 SNPs

Standard Headers

rsID: reference SNP cluster ID, chr_38: chromosome number; pos_38, position of SNP on GRCh38 reference panel; Imputation_Rsq; estimate of the squared correlation between imputed and true genotypes; REF and ALT, reference allele and alternate allele; n.obs: number of observations; caf: coding allele frequency; MAC: minor allele count; Score: p-values from Score test; Score.SE: standard error of the score statistic; Score.Stat: computed score statistic based on the derivative of the log-likelihood function; Score.pval: probability value associated with the Score.Stat; EST: estimated effect size; EST.SE: standard error of the effect size; Func.refGene: SNP location with respect to nearest gene; Gene.refGene: closet gene(s) upstream and downstream; GeneDetail.refGene: distance of SNP to nearest gene(s)

Added Headers, Abbreviations and Acronyms

QT: quantitative trait; 3M: 3 month; 12M: 12 month

QT	rsid	chr	pos_38	Imputation_Rsq	REF	ALT	n.obs	caf	MAC	Score	Score.SE	Score.Stat	Score.pval	Est	Est.SE	Func.refGene	Gene.refGene	GeneDetail.refGene	Genome-wide significance	Suggestive significance
12M	rs138164904	16	6808238	0.968829989	C	T	439	0.003416856	3	2.188468482	0.375620207	5.826279955	5.67E-09	15.51109298	2.662263589	intronic	RBFOX1	.	1	
12M	rs113154814	2	68062365	0.753430009	T	C	439	0.005694761	5	2.404201814	0.430061809	5.59036344	2.27E-08	12.99897672	2.32524716	intronic	C1D	.	1	
12M	rs111582203	12	72290549	0.795099974	A	G	439	0.007972665	8	3.07529407	0.552250588	5.568656944	2.57E-08	10.08356907	1.810772179	intronic	TRHDE	.	1	
12M	rs114280794	3	133123359	0.999639988	G	A	439	0.01594533	14	4.764666379	0.86324065	5.519511134	3.40E-08	6.393942562	1.158425521	intronic	TMEM108	.	1	
12M	rs559152067	18	68362375	0.792789996	G	A	439	0.002277904	3	1.720252761	0.315267464	5.456486816	4.86E-08	17.30748472	3.17190993	intergenic	LOC643542;TMX3	dist=462756;dist=311313	1	
12M	rs183086778	4	32609199	0.763779998	A	G	439	0.004555809	5	2.346647932	0.430540407	5.45047084	5.02E-08	12.6596035	2.322662367	intergenic	LINC02353;LOC101928622	dist=255979;dist=1287140		1
12M	rs117475675	13	73424715	0.79588002	T	C	439	0.004555809	4	2.083781896	0.384121138	5.424804025	5.80E-08	14.12263864	2.603345406	intergenic	KLF5;LINC00392	dist=347172;dist=139529		1
12M	rs566724618	13	22889700	0.869759977	C	T	439	0.003416856	3	1.92789865	0.356546436	5.407146043	6.40E-08	15.16533472	2.804683764	ncRNA_intronic	LINC00621	.		1
12M	rs186792608	2	42869559	0.931529999	A	G	439	0.003416856	3	2.044330208	0.380373238	5.374537432	7.68E-08	14.12964133	2.62899673	intergenic	HAAO;LINC01819	dist=76976;dist=158293		1
12M	rs147221953	13	22911583	0.820529997	G	A	439	0.003416856	3	1.853658494	0.344939547	5.373864819	7.71E-08	15.57914966	2.899058719	ncRNA_intronic	LINC00621	.		1
12M	rs146347965	11	24443383	0.962979972	C	A	439	0.013667426	12	3.980373837	0.744795958	5.344247369	9.08E-08	7.175451632	1.342649607	intergenic	MIR8054;LUZP2	dist=1024193;dist=53670		1
12M	rs192064655	18	63290467	0.748529971	C	T	439	0.002277904	3	1.627195325	0.305228607	5.331070832	9.76E-08	17.46582956	3.276232883	intronic	BCL2	.		1
12M	rs113537164	2	68007798	0.816209972	A	C	439	0.004555809	4	2.083286909	0.391161785	5.325895806	1.00E-07	13.61558315	2.556486955	intergenic	LINC01812;C1D	dist=182236;dist=33332		1
12M	rs186629933	11	24499372	0.972970009	A	G	439	0.013667426	12	3.971746912	0.745783477	5.325603253	1.01E-07	7.14095098	1.340871755	intronic	LUZP2	.		1
12M	rs368187808	13	73783371	0.92177999	A	C	439	0.003416856	3	1.97018281	0.370345591	5.319849503	1.04E-07	14.3645547	2.700180652	intronic	KLF12	.		1
12M	rs372194899	13	73783373	0.924839973	A	G	439	0.003416856	3	1.969004602	0.370330521	5.31688449	1.06E-07	14.35713287	2.700290535	intronic	KLF12	.		1
12M	rs148248743	3	136415753	0.708679974	C	T	439	0.002277904	3	1.586456973	0.298755282	5.310222348	1.09E-07	17.7744886	3.347221159	intronic	STAG1	.		1
12M	rs181132315	4	128726214	0.779959977	C	T	439	0.003416856	3	1.85813691	0.353270418	5.259814619	1.44E-07	14.88891893	2.830692717	intergenic	LINC02615;JADE1	dist=206818;dist=83486		1
12M	rs191298981	2	67882914	0.724789977	C	T	439	0.004555809	3	1.760281272	0.335644245	5.244485187	1.57E-07	15.62513066	2.979344989	intergenic	LINC01812;C1D	dist=57352;dist=158216		1
12M	rs547186621	20	6126094	0.76117003	A	G	439	0.003416856	4	1.874227998	0.3576548	5.240326692	1.60E-07	14.65191208	2.795992109	intergenic	FERMT1;CASC20	dist=3064;dist=300638		1
12M	rs142934021	5	53366842	0.721159995	G	A	439	0.007972665	7	2.548043395	0.487663617	5.225002041	1.74E-07	10.7143569	2.050593821	intergenic	LOC257396;FST	dist=251716;dist=113787		1
12M	rs144899985	4	32738728	0.824739993	A	G	439	0.004555809	5	2.245036023	0.430094163	5.219870935	1.79E-07	12.13657701	2.32507224	intergenic	LINC02353;LOC101928622	dist=385508;dist=1157611		1
12M	rs142497891	13	73462846	0.852349997	G	A	439	0.004555809	4	2.241850629	0.429935434	5.214389078	1.84E-07	12.12830735	2.325930644	intergenic	KLF5;LINC00392	dist=385303;dist=101398		1
12M	rs139457011	8	124378581	0.812430024	C	T	439	0.003416856	3	1.698245565	0.326123918	5.207362812	1.92E-07	15.96743605	3.0663191	intergenic	TMEM65;TRMT12	dist=5882;dist=72239		1
12M	rs144967461	4	152847797	0.855189979	T	G	439	0.018223235	16	4.171868899	0.801202658	5.207008309	1.92E-07	6.498990306	1.248123667	intronic	ARFIP1	.		1
12M	rs188353596	17	48001236	0.945710003	C	T	439	0.003416856	3	1.927521209	0.371158042	5.193262681	2.07E-07	13.99205215	2.694270059	intergenic	CDK5RAP3;COPZ2	dist=19450;dist=24931		1
12M	rs117699122	12	451096	0.758159995	T	C	439	0.003416856	4	1.919399067	0.369742041	5.191184271	2.09E-07	14.04001627	2.704588306	intergenic	CCDC77;B4GALNT3	dist=8456;dist=8843		1
12M	rs3795102	20	57683075	0.988770008	C	T	439	0.004555809	4	2.19105865	0.424843705	5.157328741	2.50E-07	12.13935544	2.353806795	intronic	PMEPA1	.		1
12M	rs367732718	19	35427102	0.764100015	G	A	439	0.002277904	3	1.635791474	0.317523551	5.151717003	2.58E-07	16.22467686	3.149372695	intergenic	LINC01531;FFAR2	dist=10262;dist=21155		1
12M	rs142956968	10	23201236	0.895780027	C	T	439	0.01594533	14	3.953608313	0.770445563	5.131586839	2.87E-07	6.660544343	1.297950235	downstream	C10orf67	dist=680		1
12M	rs113109648	16	6574271	0.706610024	T	C	439	0.002277904	3	1.641370009	0.319940791	5.130230515	2.89E-07	16.03493727	3.125578319	intronic	RBFOX1	.		1
12M	rs979665	4	100960199	0.84276998	A	G	439	0.644646925	320	14.57615127	2.841511195	5.129718053	2.90E-07	1.805278143	0.351925413	intergenic	LINC01216;PPP3CA	dist=285086;dist=63219		1
12M	rs146847867	4	32390284	0.886510015	G	T	439	0.004555809	4	2.185302341	0.426711618	5.121262811	3.03E-07	12.00169528	2.3435031	intergenic	LINC02353;LOC101928622	dist=37064;dist=1506055		1
12M	rs145764464	10	23312918	0.995850027	G	A	439	0.017084282	15	4.204896484	0.829087356	5.071717058	3.94E-07	6.11722881	1.20614552	intronic	C10orf67	.		1
12M	rs189094663	4	11621826	0.791639984	G	A	439	0.002277904	3	1.615404085	0.319409161	5.057475753	4.25E-07	15.83384692	3.13078059	intergenic	HS3ST1;LINC02360	dist=192932;dist=119125		1
12M	rs192772770	4	32808172	0.889519989	T	G	439	0.005694761	5	2.334772732	0.461927473	5.054414098	4.32E-07	10.94200798	2.164842011	intergenic	LINC02353;LOC101928622	dist=454952;dist=1088167		1
12M	rs183894319	11	24243274	0.897210002	T	C	439	0.012528474	11	3.441856532	0.682038209	5.046427729	4.50E-07	7.399039613	1.466193516	intergenic	MIR8054;LUZP2	dist=824084;dist=253779		1
12M	rs191823270	2	68025878	0.808019996	T	C	439	0.003416856	3	1.79080594	0.35569611	5.034651464	4.79E-07	14.1543619	2.811388633	intergenic	LINC01812;C1D	dist=200316;dist=15252		1
12M	rs117360642	22	33435071	0.772360027	G	A	439	0.013667426	13	3.472105912	0.689882552	5.032894224	4.83E-07	7.295291363	1.449522092	intronic	LARGE1	.		1
12M	rs561254473	13	105826900	0.828740001	A	G	439	0.003416856	3	1.848475898	0.368215591	5.020091341	5.16E-07	13.63356541	2.715800268	intergenic	LINC00343;LINC00460	dist=65104;dist=549663		1
12M	rs139206944	13	105814677	0.821319997	C	T	439	0.003416856	3	1.855091687	0.369681378	5.018082589	5.22E-07	13.57407456	2.705032115	intergenic	LINC00343;LINC00460	dist=52881;dist=561886		1
12M	rs564991659	4	79495431	0.717190027	A	G	439	0.002277904	3	1.478600686	0.29470879	5.017158418	5.24E-07	17.02412208	3.393180096	ncRNA_intronic	LINC00989	.		1
12M	rs12212412	6	81267112	0.907299995	C	T	439	0.009111617	8	2.865042865	0.571860889	5.010034645	5.44E-07	8.760932498	1.748677029	intergenic	BCKDHB;TENT5A	dist=920842;dist=478618		1
12M	rs71568803	6	81284001	0.912039995	A	G	439	0.009111617	8	2.871784743	0.573932376	5.003698802	5.62E-07	8.718272413	1.74236555	intergenic	BCKDHB;TENT5A	dist=937731;dist=461729		1
12M	rs181164671	4	60202387	0.850399971	G	T	439	0.006833713	6	2.365357436	0.473829215	4.992004204	5.98E-07	10.53545041	2.110465052	intergenic	LINC02429;MIR548AG1	dist=1155428;dist=720232		1
12M	rs183601329	4	79385832	0.769129992	C	T	439	0.003416856	3	1.528621191	0.307223916	4.97559308	6.50E-07	16.19533121	3.254954928	intergenic	NAA11;GK2	dist=59771;dist=20529		1
12M	rs76887296	4	32865383	0.91079998	C	T	439	0.005694761	5	2.374205059	0.477289182	4.974353383	6.55E-07	10.42209538	2.09516586	intergenic	LINC02353;LOC101928622	dist=512163;dist=1030956		1
12M	rs113791989	13	61344755	0.787899971	G	A	439	0.002277904	3	1.593884468	0.320567115	4.972077284	6.62E-07	15.51025373	3.119471569	intergenic	MIR3169;PCDH20	dist=144874;dist=64931		1
12M	rs181669665	4	128644045	0.701950014	G	A	439	0.005694761	4	1.881997947	0.380114274	4.951137266	7.38E-07	13.02539159	2.630787815	intergenic	LINC02615;JADE1	dist=124649;dist=165655		1
12M	rs72825069	17	34273909	0.886940002	C	T	439	0.021640091	20	4.470064227	0.904834992	4.940198229	7.80E-07	5.459778049	1.105173881	intergenic	CCL7;CCL11	dist=1667;dist=11833		1
12M	rs147944608	10	108663951	0.972880006	C	T	439	0.012528474	11	3.51284077	0.711541711	4.936942858	7.94E-07	6.938374489	1.405398986	intergenic	LINC01435;XPNPEP1	dist=594658;dist=1200815		1
12M	rs191122698	4	60001148	0.901989996	T	C	439	0.006833713	6	2.484118908	0.503688379	4.931856703	8.15E-07	9.791483996	1.98535452	intergenic	LINC02429;MIR548AG1	dist=954189;dist=921471		1
12M	rs146600651	1	152295232	0.872979999	C	T	439	0.014806378	14	3.66583614	0.743346782	4.931528902	8.16E-07	6.634223785	1.345267141	intergenic	HRNR;FLG	dist=71039;dist=6933		1
12M	rs141926026	5	175205538	0.808709979	C	T	439	0.002277904	3	1.568445555	0.318998882	4.916774461	8.80E-07	15.41314011	3.134807227	intergenic	LINC01951;DRD1	dist=209807;dist=234499		1
12M	rs192500829	2	176385923	0.71104002	A	G	439	0.004555809	3	1.674098367	0.340713541	4.913506992	8.95E-07	14.42122604	2.935016895	intergenic	MTX2;MIR1246	dist=47898;dist=215057		1
12M	rs147669485	18	29023686	0.80734998	G	T	439	0.003416856	4	1.924640141	0.391979738	4.910050065	9.11E-07	12.52628537	2.551152271	intergenic	CDH2;MIR302F	dist=846556;dist=1275226		1
12M	rs532464521	4	43569022	0.992690027	T	C	439	0.004555809	4	2.094609056	0.427328656	4.901634909	9.50E-07	11.47041005	2.340119219	intergenic	LINC02383;LINC02475	dist=76479;dist=447839		1
12M	rs556435089	4	43549016	0.982659996	G	A	439	0.004555809	4	2.093380466	0.427400072	4.897941303	9.68E-07	11.45985137	2.339728197	intergenic	LINC02383;LINC02475	dist=56473;dist=467845		1
12M	rs147267707	4	43024541	0.989459991	G	A	439	0.004555809	4	2.094031845	0.428388026	4.888166139	1.02E-06	11.41060403	2.334332284	intronic	GRXCR1	.		1

12M	rs77618729	4	43048951	0.989409983	T	C	439	0.004555809	4	2.093237083	0.42828012	4.887542018	1.02E-06	11.41202168	2.334920424	intergenic	GRXCR1;LINC02383	dist=18293;dist=408583		1
12M	rs5994128	22	17010042	0.711239994	A	G	439	0.774487472	220	-11.14584694	2.280785591	-4.886845561	1.02E-06	-2.142615062	0.438445422	intergenic	GAB4;CECR7	dist=1820;dist=26528		1
12M	rs148953849	3	56866690	0.716539979	G	A	439	0.004555809	4	1.732750622	0.35482831	4.883349426	1.04E-06	13.76256993	2.818264419	intronic	ARHGEF3	.		1
12M	rs140561530	2	205755707	0.75770998	G	T	439	0.002277904	3	1.412604553	0.289304563	4.882759325	1.05E-06	16.87757453	3.456564906	intronic	NRP2	.		1
12M	rs117262205	21	37733520	0.994069993	C	T	439	0.006833713	6	2.582956261	0.529108171	4.881716823	1.05E-06	9.226311531	1.889972701	intronic	KCNJ6	.		1
12M	rs112507626	12	72316321	0.957830012	G	A	439	0.022779043	19	4.543772122	0.930816356	4.881491491	1.05E-06	5.244312112	1.074325771	intronic	TRHDE	.		1
12M	rs146300183	2	164138767	0.828170002	T	C	439	0.004555809	4	1.984193903	0.407444048	4.869856149	1.12E-06	11.95220834	2.454324722	intergenic	FIGN;GRB14	dist=402759;dist=353650		1
12M	rs115690445	2	239682619	0.762839973	A	C	439	0.007972665	7	2.51110667	0.515854898	4.867854661	1.13E-06	9.436480453	1.938529621	intergenic	HDAC4-AS1;LOC150935	dist=280255;dist=80241		1
12M	rs76356799	3	179875980	0.997150004	G	A	439	0.004555809	4	2.106771109	0.432864845	4.867041374	1.13E-06	11.24378991	2.310189917	intronic	PEX5L	.		1
12M	rs142721557	7	117578274	0.997680008	A	C	439	0.003416856	3	1.830638975	0.377148582	4.853893297	1.21E-06	12.86997625	2.651474902	intronic	CFTR	.		1
12M	rs60006744	9	37672694	0.97507	G	T	439	0.051252847	45	7.444177374	1.53385479	4.853247792	1.21E-06	3.164085559	0.651952197	intronic	FRMPD1	.		1
12M	rs2148140	9	37673842	0.975399971	C	T	439	0.051252847	45	7.444177374	1.53385479	4.853247792	1.21E-06	3.164085559	0.651952197	intronic	FRMPD1	.		1
12M	rs113399724	9	37674360	0.97507	C	T	439	0.051252847	45	7.444177374	1.53385479	4.853247792	1.21E-06	3.164085559	0.651952197	intronic	FRMPD1	.		1
12M	rs142215699	7	117559820	0.997420013	T	C	439	0.003416856	3	1.830346943	0.377150347	4.853096273	1.22E-06	12.86780275	2.651462495	ncRNA_intronic	CFTR-AS1	.		1
12M	rs201355675	7	117585727	0.99436003	G	A	439	0.003416856	3	1.830359186	0.377152899	4.853095897	1.22E-06	12.86771469	2.651444555	intronic	CFTR	.		1
12M	rs10973466	9	37661113	0.992219985	G	A	439	0.051252847	45	7.461346315	1.537487847	4.852946531	1.22E-06	3.156412936	0.650411645	intronic	FRMPD1	.		1
12M	rs10973475	9	37666364	0.986980021	C	T	439	0.051252847	45	7.456175961	1.536478706	4.852768822	1.22E-06	3.158370371	0.650838828	intronic	FRMPD1	.		1
12M	rs144801217	2	129943819	0.77737999	T	C	439	0.012528474	11	3.020258487	0.622809182	4.849412265	1.24E-06	7.786353193	1.605628222	ncRNA_intronic	LINC01856	.		1
12M	rs148157126	20	32361036	0.807780027	C	T	439	0.004555809	5	2.092134134	0.43147068	4.848844269	1.24E-06	11.23794616	2.317654586	intronic	ASXL1	.		1
12M	rs79914278	9	37658199	0.999939978	C	T	439	0.051252847	45	7.463015515	1.54104852	4.842816705	1.28E-06	3.142546548	0.648908835	intronic	FRMPD1	.		1
12M	rs117285875	10	10833324	0.944440007	G	T	439	0.004555809	4	2.102691811	0.434748428	4.836571393	1.32E-06	11.1249888	2.30018083	intronic	CELF2	.		1
12M	rs111896192	8	37846591	0.778270006	C	T	439	0.003416856	4	1.834661883	0.379554059	4.833730106	1.34E-06	12.7352876	2.634670807	intronic	BRF2	.		1
12M	rs184618624	4	150066449	0.923269999	T	C	439	0.002277904	3	1.489513245	0.308282145	4.831655903	1.35E-06	15.67283732	3.243781766	intergenic	IQCM;DCLK2	dist=250606;dist=11996		1
12M	rs76487561	2	147457410	0.994270027	C	T	439	0.028473804	25	5.507313038	1.141042849	4.826561108	1.39E-06	4.229956054	0.876391277	intergenic	PABPC1P2;ACVR2A	dist=866420;dist=387108		1
12M	rs193041547	20	32184077	0.881550014	T	C	439	0.004555809	4	2.07477076	0.430413643	4.820411237	1.43E-06	11.19948524	2.32334643	intergenic	TM9SF4;TSPY26P	dist=16819;dist=5069		1
12M	rs571346923	3	184247590	0.805440009	G	A	439	0.003416856	3	1.651446737	0.342609142	4.820206279	1.43E-06	14.06911168	2.918777925	intronic	ALG3	.		1
12M	rs80212581	16	6362966	0.827440023	C	T	439	0.003416856	3	1.753654591	0.363868409	4.819474705	1.44E-06	13.24510343	2.74824628	intronic	RBFOX1	.		1
12M	rs576047962	7	117468967	0.980849981	A	G	439	0.003416856	3	1.817321889	0.377176493	4.818226812	1.45E-06	12.7744621	2.651278697	intergenic	ASZ1;CFTR	dist=41474;dist=11058		1
12M	rs189709453	3	177856520	0.849210024	G	A	439	0.003416856	3	1.623833795	0.337340968	4.813627607	1.48E-06	14.26932412	2.964359789	ncRNA_intronic	LINC02015	.		1
12M	rs56821264	6	148382765	0.986599982	C	T	439	0.006833713	6	2.51782977	0.523428582	4.810264197	1.51E-06	9.18991503	1.910480309	intronic	SASH1	.		1
12M	rs117245766	13	105804682	0.738900006	A	G	439	0.003416856	3	1.590303394	0.330622791	4.8100235	1.51E-06	14.54837244	3.024594879	intergenic	LINC00343;LINC00460	dist=42886;dist=571881		1
12M	rs183749982	11	102098824	0.793789983	G	A	439	0.004555809	4	1.971855551	0.409991583	4.80950252	1.51E-06	11.73073478	2.439074464	intergenic	CFAP300;YAP1	dist=14264;dist=11623		1
12M	rs188993522	7	117634677	0.964349985	T	C	439	0.003416856	3	1.746488875	0.364012714	4.797878776	1.60E-06	13.18052527	2.747156793	intronic	CFTR	.		1
12M	rs182868205	3	177994660	0.920109987	C	T	439	0.003416856	3	1.649581368	0.344172256	4.792894657	1.64E-06	13.92586001	2.90552182	intergenic	LINC02015;LINC01014	dist=95436;dist=424541		1
12M	rs146249289	20	32094428	0.899169981	C	T	439	0.004555809	4	2.061071174	0.430206938	4.790883158	1.66E-06	11.13622941	2.324462744	intronic	HCK	.		1
12M	rs187982129	10	10793335	0.934319973	G	T	439	0.004555809	4	2.104360473	0.43943772	4.788757028	1.68E-06	10.89746466	2.275635325	ncRNA_intronic	SFTA1P	.		1
12M	rs13103178	4	130157785	0.71529001	T	C	439	0.019362187	21	4.095545102	0.855702041	4.786181292	1.70E-06	5.593280207	1.168631079	intergenic	LINC02465;LINC02479	dist=202417;dist=218576		1
12M	rs141411318	11	24584003	0.980279982	C	T	439	0.022779043	20	4.434973388	0.926703454	4.78575252	1.70E-06	5.164276125	1.079093853	intronic	LUZP2	.		1
12M	rs558784715	7	117403240	0.962360024	A	G	439	0.003416856	3	1.805721393	0.377457708	4.783903883	1.72E-06	12.67401298	2.649303432	intronic	ASZ1	.		1
12M	rs72874841	11	24549456	0.97706002	C	T	439	0.022779043	20	4.439777519	0.9297034	4.775477341	1.79E-06	5.136560051	1.075611857	intronic	LUZP2	.		1
12M	rs529110230	7	117535853	0.949980021	T	C	439	0.003416856	3	1.806947008	0.378567349	4.773119008	1.81E-06	12.60837477	2.641537902	intronic	CFTR	.		1
12M	rs181832855	6	151727964	0.80726999	C	T	439	0.003416856	3	1.6160007	0.338696272	4.771238528	1.83E-06	14.0870713	2.95249781	intronic	ESR1	.		1
12M	rs61546457	22	34073343	0.989449978	T	C	439	0.050113895	44	6.361384106	1.334216593	4.767879623	1.86E-06	3.573542443	0.749503495	intergenic	LARGE1;LINC02885	dist=150520;dist=683324		1
12M	rs35373244	22	34073359	0.989570022	G	A	439	0.050113895	44	6.361383523	1.334255598	4.767739805	1.86E-06	3.573333185	0.749481585	intergenic	LARGE1;LINC02885	dist=150536;dist=683308		1
12M	rs10038482	5	37593566	0.945800006	C	T	439	0.004555809	4	1.945686385	0.408444718	4.763646826	1.90E-06	11.66289247	2.448311744	intronic	WDR70	.		1
12M	rs4896997	6	148362788	0.973269999	C	T	439	0.006833713	6	2.511111457	0.527345105	4.761799164	1.92E-06	9.029758847	1.896291425	intronic	SASH1	.		1
12M	rs186767531	3	177921989	0.890630007	T	C	439	0.003416856	3	1.610325603	0.338183106	4.761697361	1.92E-06	14.08023428	2.956977988	intergenic	LINC02015;LINC01014	dist=22765;dist=497212		1
12M	rs17078283	6	148375784	0.969319999	C	T	439	0.006833713	6	2.511039365	0.527359738	4.761530287	1.92E-06	9.028998503	1.896238805	intronic	SASH1	.		1
12M	rs4131286	6	148367827	0.969200015	G	T	439	0.006833713	6	2.510967274	0.527374461	4.761260659	1.92E-06	9.028235175	1.896185868	intronic	SASH1	.		1
12M	rs567095960	13	39877533	0.83209002	T	G	439	0.003416856	3	1.525495529	0.320695601	4.75683334	1.97E-06	14.83286119	3.118221752	intergenic	COG6;LINC00332	dist=85867;dist=304276		1
12M	rs182211730	7	116363561	0.920289993	G	A	439	0.004555809	4	1.988619676	0.418066319	4.756708646	1.97E-06	11.37788056	2.391964991	intergenic	LOC102724434;CAV2	dist=76834;dist=136177		1
12M	rs548247109	7	67561468	0.921350002	G	A	439	0.006833713	6	2.378475952	0.500107071	4.755933457	1.98E-06	9.509830457	1.999571807	intergenic	LINC01372;LOC102723427	dist=221443;dist=458785		1
12M	rs35482368	22	34073458	0.999559998	T	C	439	0.050113895	44	6.407425495	1.347517024	4.754986676	1.98E-06	3.528702488	0.742105652	intergenic	LARGE1;LINC02885	dist=150635;dist=683209		1
12M	rs140276610	16	6383734	0.729809999	C	T	439	0.003416856	4	1.668191344	0.35107035	4.751729512	2.02E-06	13.53497813	2.848431943	intronic	RBFOX1	.		1
12M	rs569429270	5	116060403	0.922169983	C	T	439	0.003416856	3	1.696972577	0.357132375	4.751662676	2.02E-06	13.30504599	2.800082182	downstream	ARL14EPL	dist=914		1
12M	rs1442156642	9	37664627	0.928049982	G	A	439	0.15261959	134	10.58008025	2.239682708	4.723919246	2.31E-06	2.109191284	0.446491816	intronic	FRMPD1	.		1
12M	rs10814594	9	37666670	0.925939977	T	C	439	0.15261959	134	10.57879091	2.239737923	4.723227126	2.32E-06	2.108830269	0.446480809	intronic	FRMPD1	.		1
12M	rs2057644	9	37665983	0.928040028	G	A	439	0.15261959	134	10.57853162	2.239752645	4.723080313	2.32E-06	2.108750858	0.446477874	intronic	FRMPD1	.		1
12M	rs2057643	9	37666013	0.928040028	T	C	439	0.15261959	134	10.57853162	2.239752645	4.723080313	2.32E-06	2.108750858	0.446477874	intronic	FRMPD1	.		1
12M	rs1125576	9	37667822	0.927389979	T	C	439	0.15261959	134	10.57668904	2.239823833	4.722107554	2.33E-06	2.108249535	0.446463684	intronic	FRMPD1	.		1
12M	rs572469018	16	72297050	0.963840008	C	T	439	0.003416856	3	1.768848944	0.374808516	4.719340325	2.37E-06	12.59133697	2.668029026	ncRNA_intronic	LINC01572	.		1
12M	rs2229593	3	42864724	0.999499977	C	T	439	0.027334852	24	4.870496863	1.032388754	4.717696551	2.39E-06	4.569689987	0.968627367	exonic	ACKR2	.		1
12M	rs537465910	16	72326220	0.957490027	G	A	439	0.003416856	3	1.764616188	0.374136714	4.716501002	2.40E-06	12.60635704	2.672819752	ncRNA_intronic	LINC01572	.		1
12M	rs115901263	4	86154667	0.712899983	A	G	439	0.005694761	6	2.238202026	0.474593389	4.716041306	2.40E-06	9.93701433	2.107066856	ncRNA_intronic	MAPK10-AS1	.		1
12M	rs550597094	16	72420594	0.951659977	G	A	439	0.003416856	3	1.75871144	0.372991675	4.715149313	2.42E-06	12.64143312	2.681024986	ncRNA_intronic	LINC01572	.		1
12M	rs181104894	19	34621605	0.903439999	A	C	439	0.003416856	3	1.675710965	0.35551557	4.713467166	2.44E-06	13.25811742	2.81281633	ncRNA_intronic	SCGB1B2P	.		1
12M	rs543741501	16	72351658	0.946910024	T	C	439	0.003416856	3	1.762934021	0.374136356	4.712009391	2.45E-06	12.59436385	2.672822315	ncRNA_intronic	LINC01572	.		1
12M	rs77846488	7	117571897	0.990610003	T	C	439	0.004555809	4	2.054832011	0.436103169	4.71180252	2.46E-06	10.80432993	2.293035389	intronic	CFTR	.		1
12M	rs77158902	17	34365017	0.998969972	C	T	439	0.022779043	20	4.491884806	0.953623181	4.710335166	2.47E-06	4.939409255	1.048632227	intergenic	CCL1;C17orf102	dist=1784;dist=209106		1
12M	rs75862363	7	117570346	0.991659999	G	A	439	0.004555809	4	2.053944777	0.436108133	4.709714457	2.48E-06	10.79941901	2.293009291	intronic	CFTR	.		1
12M	rs78282451	7	117553939	0.988619983	C	A	439	0.004555809	4	2.053353534	0.436106063	4.708381076	2.50E-06	10.79641279	2.293020173	ncRNA_intronic	CFTR-AS1	.		1
12M	rs149214138	16	72417776	0.94986999	T	C	439	0.003416856	3	1.753884158	0.372559459	4.707662358	2.51E-06	12.63600287	2.68413531	ncRNA_intronic	LINC01572	.		1
12M	rs55939894	3	7921601	0.98773998	A	G	439	0.187927107	165	-12.24219411	2.600577928	-4.707489817	2.51E-06	-1.810170642	0.384529911	intergenic	GRM7;LOC101927394	dist=180068;dist=31204		1
12M	rs143281973	7	116367942	0.928539991	C	T	439	0.004555809	4	2.006625392	0.427483854	4.694037855	2.68E-06	10.98062023	2.339269638	intergenic	LOC102724434;CAV2	dist=81215;dist=131796		1
12M	rs149859280	20	32090367	0.777029991	C	T	439	0.004555809	5	2.082920752	0.443760413	4.693795782	2.68E-06	10.57731976	2.25346825	intronic	HCK	.		1
12M	rs72878847	11	24599192	0.947830021	A	G	439	0.023917995	20	4.373449314	0.932123939	4.691918242	2.71E-06	5.033577665	1.072818708	intronic	LUZP2	.		1

12M	rs75343152	2	147447179	0.984929979	T	C	439	0.029612756	25	5.373706425	1.145505076	4.691124061	2.72E-06	4.095245108	0.872977362	intergenic	PABPC1P2;ACVR2A	dist=856189;dist=397339		1
12M	rs75330406	2	147481113	0.990610003	G	A	439	0.027334852	24	5.0599299	1.078749077	4.690553168	2.72E-06	4.348141072	0.926999635	intergenic	PABPC1P2;ACVR2A	dist=890123;dist=363405		1
12M	rs78849093	2	147478023	0.988759995	C	A	439	0.027334852	24	5.057603105	1.078451085	4.689691702	2.74E-06	4.348543728	0.927255778	intergenic	PABPC1P2;ACVR2A	dist=887033;dist=366495		1
12M	rs138733283	20	32087884	0.776130021	C	T	439	0.004555809	5	2.081660055	0.444061489	4.687774347	2.76E-06	10.55658837	2.251940385	intronic	HCK	.		1
12M	rs192072893	11	24171605	0.851140022	G	A	439	0.011389522	10	2.98365585	0.63649116	4.687662667	2.76E-06	7.364851171	1.571113729	intergenic	MIR8054;LUZP2	dist=752415;dist=325448		1
12M	rs139816293	20	32333540	0.77214998	C	T	439	0.005694761	6	2.161099014	0.461179259	4.6860282	2.79E-06	10.16096909	2.168354235	UTR3	KIF3B	NM_004798:c.*22210>0		1
12M	rs148996503	16	72564549	0.936999977	G	A	439	0.003416856	3	1.724389568	0.368079708	4.684826489	2.80E-06	12.7774994	2.716802848	ncRNA_intronic	LINC01572	.		1
12M	rs537719803	16	72564328	0.937420011	C	A	439	0.003416856	3	1.724217925	0.368081111	4.68434232	2.81E-06	12.72638607	2.716792496	ncRNA_intronic	LINC01572	.		1
12M	rs6724770	2	147469854	0.987339973	A	G	439	0.027334852	24	5.05104599	1.078982106	4.68130654	2.85E-06	4.338632229	0.926799429	intergenic	PABPC1P2;ACVR2A	dist=878864;dist=374664		1
12M	rs80339745	2	147470110	0.994419992	G	A	439	0.027334852	24	5.050651164	1.078960151	4.681035866	2.85E-06	4.338469649	0.926818288	intergenic	PABPC1P2;ACVR2A	dist=879120;dist=374408		1
12M	rs75304512	2	147460438	0.994880021	A	G	439	0.027334852	24	5.052915513	1.079656239	4.680115142	2.87E-06	4.334819707	0.926220739	intergenic	PABPC1P2;ACVR2A	dist=869448;dist=384080		1
12M	rs77592434	2	147469077	0.994220018	A	G	439	0.027334852	24	5.051588616	1.07940957	4.679955372	2.87E-06	4.335662295	0.926432401	intergenic	PABPC1P2;ACVR2A	dist=878087;dist=375441		1
12M	rs77928469	2	147466550	0.994260013	C	T	439	0.027334852	24	5.051484579	1.079409282	4.679857902	2.87E-06	4.335570987	0.926432186	intergenic	PABPC1P2;ACVR2A	dist=875560;dist=377968		1
12M	rs10210722	2	147465215	0.987999976	T	C	439	0.027334852	24	5.051665699	1.07945012	4.679850978	2.87E-06	4.335402712	0.9263976	intergenic	PABPC1P2;ACVR2A	dist=874225;dist=379303		1
12M	rs1258074269	2	147466224	0.987990022	A	C	439	0.027334852	24	5.051665699	1.07945012	4.679850978	2.87E-06	4.335402712	0.9263976	intergenic	PABPC1P2;ACVR2A	dist=875234;dist=378294		1
12M	rs74380227	2	147462714	0.995109975	C	A	439	0.027334852	24	5.051193478	1.079667701	4.678470397	2.89E-06	4.333250397	0.926210907	intergenic	PABPC1P2;ACVR2A	dist=871724;dist=381804		1
12M	rs62582460	9	138095976	0.890290022	T	C	439	0.05808656	51	6.819364418	1.458397024	4.675931387	2.93E-06	3.206212925	0.68568434	intronic	CACNA1B	.		1
12M	rs118171627	6	143294753	0.810609996	C	A	439	0.003416856	3	1.522870764	0.325892127	4.672928974	2.97E-06	14.33888267	3.068500023	intronic	AIG1	.		1
12M	rs10175536	2	147460050	0.999530017	A	G	439	0.027334852	24	5.059239074	1.084265764	4.666050745	3.07E-06	4.30341979	0.92228311	intergenic	PABPC1P2;ACVR2A	dist=869060;dist=384468		1
12M	rs17875371	6	30492455	0.846430004	C	T	439	0.018223235	16	3.620688814	0.776176074	4.664777664	3.09E-06	6.009947767	1.288367464	intronic	HLA-E	.		1
12M	rs112796175	12	72396569	0.974420011	C	T	439	0.025056948	21	4.562372955	0.978318805	4.663482835	3.11E-06	4.766833481	1.022161687	intronic	TRHDE	.		1
12M	rs140545577	16	6805089	0.953159988	C	T	439	0.003416856	3	1.756164801	0.376771481	4.6610874	3.15E-06	12.37112584	2.6541287	intronic	RBFOX1	.		1
12M	rs138055631	20	32192841	0.808770001	G	A	439	0.005694761	5	2.092550815	0.449042548	4.660027927	3.16E-06	10.37769794	2.226960461	UTR3	PLAGL2	NM_002657:c.*36110>0		1
12M	rs74517425	2	147435566	0.967649996	A	C	439	0.029612756	25	5.255817679	1.12800262	4.659402016	3.17E-06	4.130665953	0.886522764	intergenic	PABPC1P2;ACVR2A	dist=844576;dist=408952		1
12M	rs5994839	22	34088192	0.999319971	C	A	439	0.039863326	35	5.549459281	1.192274826	4.654513506	3.25E-06	3.903893133	0.838732797	intergenic	LARGE1;LINC02885	dist=165369;dist=668475		1
12M	rs12193585	6	81292327	0.816420019	C	T	439	0.010250569	9	2.759336125	0.592983165	4.653312755	3.27E-06	7.847293194	1.686388717	intergenic	BCKDHB;TENT5A	dist=946057;dist=453403		1
12M	rs11920261	3	7935524	0.993160009	G	T	439	0.185649203	162	-12.09011362	2.598457274	-4.65280447	3.27E-06	-1.790602646	0.384843734	intergenic	GRM7;LOC101927394	dist=193991;dist=17281		1
12M	rs2171551	3	7936391	0.993030012	C	A	439	0.185649203	162	-12.08866418	2.598460193	-4.652241434	3.28E-06	-1.790383954	0.384843302	intergenic	GRM7;LOC101927394	dist=194858;dist=16414		1
12M	rs74492945	2	147426871	0.976989985	G	A	439	0.029612756	25	5.284785249	1.136173009	4.651391299	3.30E-06	4.093911105	0.880147647	intergenic	PABPC1P2;ACVR2A	dist=835881;dist=417647		1
12M	rs75380640	2	147428229	0.976960003	A	G	439	0.029612756	25	5.284373278	1.136163974	4.65106569	3.30E-06	4.093657074	0.880154646	intergenic	PABPC1P2;ACVR2A	dist=837239;dist=416289		1
12M	rs577438374	7	67418626	0.944700003	C	T	439	0.006833713	6	2.39527184	0.515335444	4.647985827	3.35E-06	9.019340475	1.940483644	intergenic	LINC01372;LOC102723427	dist=78601;dist=601627		1
12M	rs185899532	1	227321571	0.810259998	T	C	439	0.005694761	4	1.908112393	0.410717289	4.645804902	3.39E-06	11.31144228	2.434764807	intergenic	CDC42BPA;ZNF678	dist=3079;dist=241985		1
12M	rs140512918	5	116111989	0.876070023	T	C	439	0.003416856	3	1.688393978	0.363434391	4.645663762	3.39E-06	12.78267517	2.75152827	intronic	COMMD10	.		1
12M	rs564332768	7	64988831	0.864449978	A	C	439	0.007972665	6	2.356215761	0.507434013	4.643393431	3.43E-06	9.150733523	1.970699589	intronic	ERV3-1-ZNF117;ZNF117	.		1
12M	rs148815783	11	1013992	0.905279994	C	T	439	0.007972665	8	2.639026369	0.568648462	4.640874893	3.47E-06	8.161237049	1.758555711	exonic	MUC6	.		1
12M	rs185242027	5	29071862	0.763419986	G	A	439	0.004555809	3	1.595602789	0.344148816	4.636374483	3.55E-06	13.47200475	2.905719716	ncRNA_intronic	LOC101929645	.		1
12M	rs139635002	3	164614597	0.849020004	T	C	439	0.007972665	8	2.540439901	0.548123379	4.63479574	3.57E-06	8.4557527	1.824406765	intergenic	MIR1263;LINC01324	dist=443041;dist=99498		1
12M	rs530059247	16	72820010	0.909240007	C	T	439	0.003416856	3	1.656813634	0.357508509	4.634333424	3.58E-06	12.96286187	2.797136219	intronic	ZFHX3	.		1
12M	rs190354334	7	116435792	0.86668998	G	A	439	0.004555809	5	2.00243762	0.432429385	4.630669627	3.64E-06	10.70849897	2.312516295	intergenic	LOC102724434;CAV2	dist=149065;dist=63946		1
12M	rs186893139	7	116456916	0.854889989	C	T	439	0.004555809	4	1.880403456	0.406387212	4.627122608	3.71E-06	11.38599462	2.460707351	intergenic	LOC102724434;CAV2	dist=170189;dist=42822		1
12M	rs139548692	11	82893220	0.700569987	C	T	439	0.004555809	6	1.861738012	0.402460972	4.625884601	3.73E-06	11.49399551	2.484712979	intronic	PRCP	.		1
12M	rs74384278	10	44647124	0.802160025	T	C	439	0.009111617	8	2.588932233	0.559803403	4.624716857	3.75E-06	8.261323236	1.786341411	intergenic	CXCL12;TMEM72-AS1	dist=262027;dist=163900		1
12M	rs180797135	16	58970453	0.854189992	G	A	439	0.003416856	3	1.571216829	0.339826153	4.623590074	3.77E-06	13.60575114	2.942681104	intergenic	GOT2;APOOP5	dist=236196;dist=783629		1
12M	rs138873576	7	116385305	0.899990022	G	T	439	0.004555809	4	1.991831401	0.430896074	4.622533181	3.79E-06	10.72772174	2.320745209	intergenic	LOC102724434;CAV2	dist=98578;dist=114433		1
12M	rs150788978	11	18175633	0.804279983	G	A	439	0.007972665	8	2.532677712	0.547949348	4.622101879	3.80E-06	8.435272154	1.824986202	intergenic	MRGPRX4;SLC25A51P4	dist=1353;dist=33505		1
12M	rs185948380	12	70219358	0.802529991	T	C	439	0.005694761	5	2.022172064	0.437534471	4.621743424	3.81E-06	10.56315269	2.285534207	intergenic	MYRFL;PRANCR	dist=260261;dist=2832		1
12M	rs138138661	3	147940027	0.736020029	C	T	439	0.004555809	4	1.755727133	0.380325922	4.616375137	3.91E-06	12.13794502	2.629323801	intergenic	LOC440982;LINC02032	dist=430117;dist=138132		1
12M	rs76294395	9	135780517	0.958130002	G	A	439	0.005694761	5	2.250462353	0.487505863	4.616277515	3.91E-06	9.469173331	2.051257382	intronic	KCNT1	.		1
12M	rs138454387	2	163831400	0.865909994	G	A	439	0.005694761	5	2.115842897	0.458436819	4.615342424	3.92E-06	10.0675649	2.181325669	intergenic	FIGN;GRB14	dist=95392;dist=661017		1
12M	rs12350891	9	79989706	0.914240003	G	A	439	0.004555809	4	1.919901525	0.416077273	4.614290779	3.94E-06	11.08998515	2.403399716	ncRNA_intronic	LINC01507	.		1
12M	rs114259958	4	162398591	0.779690027	A	G	439	0.003416856	3	1.650939137	0.357816014	4.613933065	3.95E-06	12.89470814	2.794732381	intergenic	FSTL5;MIR4454	dist=234557;dist=694983		1
12M	rs148997617	3	148037789	0.874289989	C	T	439	0.004555809	4	1.904562472	0.412852965	4.613173833	3.97E-06	11.17389037	2.422169838	intergenic	LOC440982;LINC02032	dist=527879;dist=40370		1
12M	rs145791959	20	32134626	0.783770025	G	A	439	0.005694761	5	2.057551242	0.446297665	4.61026665	4.02E-06	10.33002637	2.240657027	intronic	TM9SF4	.		1
12M	rs181375873	1	118341865	0.994809985	G	T	439	0.010250569	9	2.965562087	0.643467132	4.608723494	4.05E-06	7.162329305	1.554080932	intergenic	SPAG17;TBX15	dist=156637;dist=541182		1
12M	rs188069356	1	118327095	0.994989991	A	G	439	0.010250569	9	2.965705392	0.643538514	4.60843497	4.06E-06	7.16108651	1.553908552	intergenic	SPAG17;TBX15	dist=141867;dist=555952		1
12M	rs566394355	7	67352900	0.961000025	T	G	439	0.006833713	6	2.423461328	0.526255376	4.605105094	4.12E-06	8.750704076	1.900218105	intergenic	LINC01372;LOC102723427	dist=12875;dist=667353		1
12M	rs145421321	20	32274323	0.810339987	C	T	439	0.005694761	6	2.10438963	0.457025953	4.604529826	4.13E-06	10.07498546	2.18805955	intergenic	POFUT1;KIF3B	dist=35665;dist=3328		1
12M	rs182996422	2	111163073	0.913890004	G	A	439	0.004555809	4	1.96047942	0.426062862	4.601385365	4.20E-06	10.79978043	2.347071496	intronic	BCL2L11	.		1
12M	rs139174841	16	59022371	0.866789997	G	A	439	0.009111617	8	2.600047823	0.565206697	4.600171647	4.22E-06	8.138919215	1.769264245	intergenic	GOT2;APOOP5	dist=288055;dist=731770		1
12M	rs994788245	3	165182662	0.830219984	G	T	439	0.003416856	3	1.457659335	0.317040616	4.597705341	4.27E-06	14.50194425	3.154169999	intergenic	SI;SLITRK3	dist=104166;dist=4058		1
12M	rs533356672	16	7052822	0.888069987	A	G	439	0.003416856	3	1.552617484	0.337713192	4.597443984	4.28E-06	13.61345689	2.961092499	intronic	RBFOX1	.		1
12M	rs146919974	2	114495286	0.777989984	T	C	439	0.004555809	5	1.870582973	0.407116317	4.594713833	4.33E-06	11.28599774	2.456300469	intronic	DPP10	.		1
12M	rs112856203	12	72465084	0.950670004	T	C	439	0.025056948	21	4.413662148	0.961866897	4.588641281	4.46E-06	4.770557441	1.039644886	intronic	TRHDE	.		1
12M	rs190601781	7	132751214	0.745159984	G	A	439	0.003416856	3	1.55039666	0.338020811	4.586689956	4.50E-06	13.56925315	2.958397728	intergenic	FLJ40288;LOC100506937	dist=22446;dist=7756		1
12M	rs181182636	7	116014504	0.859690011	A	G	439	0.003416856	3	1.592950272	0.347387686	4.585511625	4.53E-06	13.19998323	2.878628233	intronic	TFEC	.		1
12M	rs75626507	1	241251961	0.828010023	G	A	439	0.003416856	4	1.774925973	0.38713626	4.584757758	4.55E-06	11.84274951	2.583069844	intronic	RGS7	.		1
12M	rs115658028	1	7627617	0.828270018	G	A	439	0.006833713	7	2.313647214	0.504805248	4.583247148	4.58E-06	9.07923831	1.980961972	intronic	CAMTA1	.		1
12M	rs146298016	2	237657156	0.824119985	G	A	439	0.007972665	7	2.369872335	0.517388552	4.580449887	4.64E-06	8.85301746	1.93278339	intronic	LRRFIP1	.		1
12M	rs533568586	7	66745708	0.912609994	G	A	439	0.007972665	7	2.458442137	0.536776262	4.580012523	4.65E-06	8.532441903	1.862973488	intronic	RABGEF1	.		1
12M	rs754653672	3	165112751	0.968649983	A	G	439	0.003416856	3	1.695149727	0.37024662	4.578434038	4.68E-06	12.36590367	2.700902442	intergenic	SI;SLITRK3	dist=34255;dist=73969		1
12M	rs191903483	7	116474429	0.840730011	A	G	439	0.004555809	5	1.937757245	0.423414829	4.576498295	4.73E-06	10.80854514	2.361750063	intergenic	LOC102724434;CAV2	dist=187702;dist=25309		1
12M	rs139496704	12	124154097	0.772180021	A	G	439	0.007972665	7	2.256578283	0.493630452	4.571391967	4.84E-06	9.260757615	2.025806949	intronic	ZNF664-RFLNA	.		1
12M	rs573087160	16	5796693	0.802129984	G	T	439	0.002277904	3	1.508610705	0.330012697	4.571371704	4.85E-06	13.85210855	3.030186441	intergenic	MIR8065;RBFOX1	dist=164127;dist=222331		1
12M	rs139029292	3	44072032	0.860300004	G	A	439	0.005694761	6	2.199467961	0.481405962	4.568842376	4.90E-06	9.490622744	2.077248888	intergenic	ABHD5;MIR138-1	dist=337661;dist=42180		1
12M	rs72779724	16	10323552	0.977559984	C	T	439	0.085421412	74	8.555089753	1.872733081	4.568237642	4.92E-06	2.439342632	0.533978927	intergenic	GRIN2A;ATF7IP2	dist=140644;dist=62509		1
12M	rs185275344	5	120442008	0.845849991	C	T	439	0.004555809	5	1.963836945	0.429890979	4.56822088	4.92E-06	10.6264637	2.326171168	intergenic	FAM170A;PRR16	dist=806186;dist=22270		1

12M	rs189433222	7	67186631	0.864870012	C	T	439	0.006833713	7	2.388728197	0.523042224	4.566989217	4.95E-06	8.73158802	1.911891534	intronic	TYW1	.		1
12M	rs143432612	20	32304972	0.798579991	C	T	439	0.005694761	6	2.095354506	0.458885267	4.566183876	4.97E-06	9.950600304	2.179193956	intronic	KIF3B	.		1
12M	rs192078346	14	103732352	0.759670019	T	C	439	0.004555809	4	1.644493869	0.360149192	4.566146218	4.97E-06	12.67848527	2.776627086	intronic	ZFYVE21	.		1
12M	rs200198574	20	32358851	0.817799985	G	A	439	0.005694761	6	2.117861385	0.463869302	4.565642465	4.98E-06	9.842519093	2.155779645	intronic	ASXL1	.		1
																		Subtotals:	5	192
																		Total		197

Supplemental Table S2. GWAS Results SNPs Indianapolis-1 Discovery Cohort
3 month (3M) quantitative trait

Notes
Ordered by SNP P-value 42 SNPs identified of genome-wide significance; 371 SNPs of suggestive significance; Total: 413
Imputation Rsq listed only if less than 0.8
Standard Headers
rsID: reference SNP cluster ID, chr_38: chromosome number; pos_38, position of SNP on GRCh38 reference panel; Imputation_Rsq; estimate of the squared correlation between imputed and true genotypes; REF and ALT, reference allele and alternate allele; n.obs: number of observations;
caf: coding allele frequency; MAC: minor allele count; Score: p-values from Score test; Score.SE: standard error of the score statistic; Score.Stat: computed score statistic based on the derivative of the log-likelihood function; Score.pval: probability value associated with the Score.Stat;
EST: estimated effect size; EST.SE: standard error of the effect size; Func.refGene: SNP location with respect to nearest gene; Gene.refGene: closet gene(s) upstream and downstream; GeneDetail.refGene: distance of SNP to nearest gene(s)
Added Headers, Abbreviations and Acronyms
QT: quantitative trait; 3M: 3 month; 12M: 12 month

QT	rsID	chr_38	pos_38	Imputation_Rsq	REF	ALT	n.obs	caf	MAC	Score	Score.SE	Score.Stat	Score.pval	Est	Est.SE	Func.refGene	Gene.refGene	GeneDetail.refGene	Genome-wide significance	Suggestive significance
3M	rs113063005	4	23507444	0.763409972	T	C	421	0.005938	6	2.130262689	0.316937024	6.721406875	1.80E-11	21.2073894	3.155200957	intergenic	GBA3;PPARGC1A	dist=687872;dist=284577	1	
3M	rs142106992	6	141948748		C	A	421	0.003563	4	1.689946394	0.272044449	6.212023076	5.23E-10	22.83458862	3.675869896	intergenic	MIR4465;NMBR	dist=1264865;dist=125736	1	
3M	rs181217257	2	239067423		C	T	421	0.004751	4	1.788144205	0.287937999	6.210170973	5.29E-10	21.56773677	3.472969885	intronic	HDAC4	.	1	
3M	rs188076929	2	239072023		T	C	421	0.004751	4	1.865485168	0.305579814	6.104739516	1.03E-09	19.97756143	3.272467463	intronic	HDAC4	.	1	
3M	rs117998251	10	13455976		C	T	421	0.003563	3	1.610926128	0.264914355	6.080931816	1.19E-09	22.95433112	3.774804885	intronic	BEND7	.	1	
3M	rs184458518	10	13429195		T	G	421	0.003563	3	1.603233248	0.265164814	6.046176429	1.48E-09	22.80157891	3.771239424	intergenic	SEPHS1;BEND7	dist=80897;dist=9286	1	
3M	rs184425183	10	13415520		A	G	421	0.003563	3	1.590271029	0.264865828	6.004062666	1.92E-09	22.66831744	3.775496477	intergenic	SEPHS1;BEND7	dist=67222;dist=22961	1	
3M	rs148153037	6	167087898		G	A	421	0.008314	8	2.445317826	0.413004518	5.920801636	3.20E-09	14.33592462	2.421281019	intergenic	CEP43;CCR6	dist=35180;dist=23909	1	
3M	rs111285015	19	22940396	0.738849998	G	A	421	0.003563	3	1.280187265	0.216410877	5.915540303	3.31E-09	27.33476425	4.62083983	intergenic	ZNF723;ZNF728	dist=81729;dist=34487	1	
3M	rs111928960	1	103168079		G	A	421	0.028504	22	4.045287237	0.68574164	5.899141891	3.65E-09	8.602572083	1.45827516	intergenic	COL11A1;LOC101928436	dist=59557;dist=325967	1	
3M	rs187520610	2	53132903		G	A	421	0.004751	4	1.843352227	0.313121747	5.887014379	3.93E-09	18.80103964	3.193645952	intergenic	MIR4431;ASB3	dist=430288;dist=537076	1	
3M	rs545428520	5	168394261		T	C	421	0.003563	4	1.606350972	0.272919814	5.885798274	3.96E-09	21.56603503	3.664079879	intronic	WWC1	.	1	
3M	rs116672066	1	103007360		G	A	421	0.027316	23	4.276024986	0.728927557	5.866186486	4.46E-09	8.047694764	1.37187844	intronic	COL11A1	.	1	
3M	rs192134381	21	22078395		T	C	421	0.003563	3	1.565034889	0.268006444	5.83954202	5.23E-09	21.78881202	3.731253572	ncRNA_intronic	LINC01687	.	1	
3M	rs150586237	6	24491120		C	T	421	0.003563	3	1.542166452	0.264483938	5.830851057	5.51E-09	22.04614425	3.780947933	intergenic	GPLD1;ALDH5A1	dist=1542;dist=3849	1	
3M	rs528404963	5	168425020		T	C	421	0.003563	4	1.58410842	0.273744969	5.786803767	7.17E-09	21.13939767	3.653035168	intronic	WWC1	.	1	
3M	rs76526501	7	18391487		G	A	421	0.007126	6	2.233180137	0.387925975	5.75671721	8.58E-09	14.83973124	2.577811398	intronic	HDAC9	.	1	
3M	rs74455595	7	18392161		A	G	421	0.007126	6	2.233180137	0.387925975	5.75671721	8.58E-09	14.83973124	2.577811398	intronic	HDAC9	.	1	
3M	rs75090694	7	18407813		A	G	421	0.008314	7	2.37298061	0.41420374	5.729017827	1.01E-08	13.83140053	2.414270813	intronic	HDAC9	.	1	
3M	rs575473987	8	5719972		C	T	421	0.003563	3	1.471553256	0.257316966	5.718834929	1.07E-08	22.22486534	3.886257536	intergenic	CSMD1;LOC100287015	dist=725058;dist=683583	1	
3M	rs545690161	9	90268417	0.734099984	G	A	421	0.005938	6	1.900812423	0.332979511	5.708496652	1.14E-08	17.14368742	3.003187786	intergenic	MIR4290HG;LINC01508	dist=226918;dist=32479	1	
3M	rs77141817	4	37052137		T	C	421	0.003563	3	1.553761456	0.274563203	5.659030198	1.52E-08	20.61102923	3.642148656	intergenic	LINC02616;MIR4801	dist=31431;dist=189773	1	
3M	rs190822761	4	37097734		G	T	421	0.003563	3	1.550650175	0.274463961	5.649740562	1.61E-08	20.58463536	3.643465595	intergenic	LINC02616;MIR4801	dist=77028;dist=144176	1	
3M	rs10279777	7	18401966		G	A	421	0.009501	8	2.476234721	0.438670193	5.644866606	1.65E-08	12.86813351	2.279616935	intronic	HDAC9	.	1	
3M	rs10486295	7	18407184		G	A	421	0.009501	8	2.488096085	0.441539202	5.635051363	1.75E-08	12.76229003	2.264804562	intronic	HDAC9	.	1	
3M	rs147559909	2	236142879		T	C	421	0.005938	5	1.858263542	0.329805301	5.634425932	1.76E-08	17.08409752	3.032091952	intergenic	AGAP1;GBX2	dist=11086;dist=22356	1	
3M	rs17169602	7	18407118		G	A	421	0.009501	8	2.49198241	0.442489656	5.631730316	1.78E-08	12.72737168	2.259939835	intronic	HDAC9	.	1	
3M	rs139598422	13	23312875	0.783299983	A	G	421	0.003563	4	1.544940466	0.274599144	5.626166351	1.84E-08	20.48865215	3.641671944	intronic	SGCG	.	1	
3M	rs74704551	14	29692681		C	T	421	0.003563	3	1.302308212	0.231983741	5.613790894	1.98E-08	24.19907048	4.31064693	intronic	PRKD1	.	1	
3M	rs77300464	7	18369138		A	G	421	0.007126	6	2.141507953	0.382123995	5.604222657	2.09E-08	14.66597943	2.616951597	intronic	HDAC9	.	1	
3M	rs75606013	7	18374990		G	A	421	0.007126	6	2.140929626	0.382127995	5.602650559	2.11E-08	14.66171186	2.616924205	intronic	HDAC9	.	1	
3M	rs541653703	11	18680239		G	A	421	0.004751	4	1.710453669	0.305582922	5.597347058	2.18E-08	18.31694986	3.272434185	intergenic	SPTY2D1;TMEM86A	dist=45897;dist=18540	1	
3M	rs117913371	10	101158729		G	A	421	0.024941	21	3.968654489	0.710300363	5.587290525	2.31E-08	7.866095548	1.407855116	intergenic	TLX1NB;LINC01514	dist=17463;dist=17593	1	
3M	rs191053292	12	63051500	0.719120026	T	C	421	0.003563	3	1.354341299	0.243048963	5.572298207	2.51E-08	22.9266488	4.114397317	intergenic	PPM1H;AVPR1A	dist=116350;dist=91259	1	
3M	rs112351653	1	102754804		T	C	421	0.028504	24	4.178844629	0.750261996	5.569847131	2.55E-08	7.423869471	1.332867724	intergenic	OLFM3;COL11A1	dist=757570;dist=121663	1	
3M	rs114413507	1	102953612		T	C	421	0.028504	24	4.177369718	0.750215949	5.568223023	2.57E-08	7.42216029	1.332949535	intronic	COL11A1	.	1	
3M	rs138414342	11	18657851		G	A	421	0.004751	5	1.685610798	0.304678389	5.532426519	3.16E-08	18.15825054	3.282149429	intergenic	SPTY2D1;TMEM86A	dist=23509;dist=40928	1	
3M	rs16823323	3	153939413		G	A	421	0.016627	14	3.214602568	0.582174532	5.521716236	3.36E-08	9.484640653	1.717697949	intergenic	LINC02006;ARHGEF26-AS	dist=176887;dist=84988	1	
3M	rs183737367	9	90567765		T	C	421	0.003563	3	1.295690386	0.235587115	5.499835526	3.80E-08	23.34523057	4.244714311	ncRNA_intronic	LINC01501	.	1	
3M	rs560206697	19	20546292	0.700380027	C	T	421	0.002375	3	1.205675879	0.219610964	5.490053226	4.02E-08	24.99899431	4.553506728	intronic	ZNF737	.	1	
3M	rs79486609	21	15872687		G	A	421	0.003563	3	1.447442576	0.264672828	5.468799292	4.53E-08	20.66248857	3.77824957	intronic	USP25	.	1	
3M	rs151115079	11	18634194	0.792460024	T	C	421	0.004751	5	1.669234911	0.305995633	5.455093905	4.89E-08	17.8273587	3.268020498	intronic	SPTY2D1	.	1	
3M	rs185510569	2	222950143		G	A	421	0.003563	3	1.507358682	0.277399453	5.433892049	5.51E-08	19.58869056	3.604909775	intergenic	ACSL3;KCNE4	dist=5505;dist=102047		1
3M	rs187213609	9	90653183		C	T	421	0.003563	3	1.269834136	0.233965931	5.427431802	5.72E-08	23.19753039	4.274126556	intergenic	DIRAS2;SYK	dist=10359;dist=148417		1
3M	rs117280553	21	15834844		T	C	421	0.003563	3	1.465128514	0.27006967	5.425002059	5.80E-08	20.08741695	3.702748263	intronic	USP25	.		1
3M	rs183816745	5	92336071		A	G	421	0.005938	5	1.692149037	0.312309144	5.418186024	6.02E-08	17.34879088	3.201955563	intergenic	ARRDC3-AS1;NR2F1-AS1	dist=915356;dist=1113174		1
3M	rs180828621	10	122773893		G	A	421	0.007126	6	1.931459198	0.357196912	5.407267342	6.40E-08	15.13805735	2.799576273	ncRNA_intronic	DMBT1L1	.		1
3M	rs142684595	3	55285273	0.77293998	T	C	421	0.005938	5	1.730096674	0.321166729	5.386911268	7.17E-08	16.77294307	3.113647549	intergenic	LINC02017;WNT5A	dist=96059;dist=180442		1
3M	rs184785969	21	15799112		C	A	421	0.003563	3	1.446338922	0.269771603	5.361346058	8.26E-08	19.87364864	3.706839369	intronic	USP25	.		1
3M	rs77180278	1	102496326		T	C	421	0.028504	24	3.885721783	0.727780259	5.339141503	9.34E-08	7.336199959	1.374041118	intergenic	OLFM3;COL11A1	dist=499092;dist=380141		1
3M	rs189709453	3	177856520		G	A	421	0.003563	3	1.314463975	0.246356303	5.335621454	9.52E-08	21.65814876	4.05916142	ncRNA_intronic	LINC02015	.		1
3M	rs111846247	5	95775939		T	C	421	0.003563	3	1.3787786	0.259425442	5.314739335	1.07E-07	20.48657718	3.854672052	intronic	RHOBTB3	.		1
3M	rs145875128	4	32071514	0.719099998	G	A	421	0.003563	3	1.153690598	0.217089638	5.314351295	1.07E-07	24.47998599	4.606392132	ncRNA_intronic	LINC02506	.		1
3M	rs75848314	5	95762636		T	C	421	0.003563	3	1.401525911	0.264060948	5.307584942	1.11E-07	20.0998481	3.787004507	intronic	RHOBTB3	.		1
3M	rs190193113	2	52858640		G	A	421	0.003563	3	1.409901074	0.265819171	5.303985667	1.13E-07	19.95336019	3.761955902	intergenic	MIR4431;ASB3	dist=156025;dist=811339		1
3M	rs183378658	2	52336233		C	T	421	0.003563	3	1.421941101	0.268359175	5.298649105	1.17E-07	19.74461693	3.726349214	ncRNA_intronic	LOC730100	.		1
3M	rs143371352	13	46809699		C	T	421	0.003563	3	1.364496992	0.257779154	5.29327904	1.20E-07	20.53416252	3.879289636	intergenic	ESD;HTR2A	dist=12538;dist=21847		1
3M	rs75186966	13	46821623		A	C	421	0.003563	3	1.377358459	0.260942954	5.278389159	1.30E-07	20.22813447	3.832255231	intergenic	ESD;HTR2A	dist=24462;dist=9923		1
3M	rs532416695	2	176622062		G	A	421	0.003563	3	1.400161077	0.265436681	5.274934396	1.33E-07	19.87266561	3.767376828	intergenic	MIR1246;LINC01116	dist=21010;dist=7519		1
3M	rs145080832	2	48246004		G	A	421	0.004751	4	1.617719031	0.30702202	5.269065159	1.37E-07	17.16184772	3.257095367	intergenic	FBXO11;FOXN2	dist=339506;dist=68295		1
3M	rs182868205	3	177994660		C	T	421	0.003563	3	1.323927501	0.251356167	5.267137522	1.39E-07	20.95487681	3.978418395	intergenic	LINC02015;LINC01014	dist=95436;dist=424541		1
3M	rs143538552	18	31470299		A	G	421	0.003563	3	1.44755014	0.274967228	5.264446053	1.41E-07	19.14572179	3.636797033	intronic	DSG3	.		1
3M	rs7822082	8	54777660		T	C	421	0.330166	277	11.70263936	2.228199285	5.252061357	1.50E-07	2.357087802	0.448792891	intronic	RP1	.		1
3M	rs72986533	6	142290121	0.786499977	T	C	421	0.008314	8	2.07386378	0.395157536	5.248194939	1.54E-07	13.28127256	2.530636288	intergenic	VTA1;ADGRG6	dist=65437;dist=11798		1
3M	rs373746073	18	31478421		C	A	421	0.003563	3	1.441999195	0.274877768	5.245965155	1.55E-07	19.08471968	3.637980642	UTR3	DSG3	NM_001944:c.*21610>0		1
3M	rs181193202	2	52300216		T	C	421	0.003563	3	1.397666205	0.26682604	5.238117712	1.62E-07	19.63120884	3.747760153	ncRNA_intronic	LOC730100	.		1
3M	rs186767531	3	177921989		T	C	421	0.003563	3	1.293967205	0.247047316	5.237730262	1.63E-07	21.20132426	4.047807581	intergenic	LINC02015;LINC01014	dist=22765;dist=497212		1
3M	rs140797780	8	22230279		C	T	421	0.003563	3	1.415632732	0.270894217	5.225776871	1.73E-07	19.29083956	3.691477848	intronic	PHYHIP	.		1
3M	rs137880949	12	62912517	0.737010002	T	C	421	0.004751	3	1.263235635	0.24178583	5.224605748	1.75E-07	21.60840343	4.135891677	intronic	PPM1H	.		1
3M	rs148532212	6	165086368		T	C	421	0.003563	3	1.406500453	0.26958124	5.217352852	1.81E-07	19.35354569	3.709456929	intergenic	MEAT6;C6orf118	dist=264305;dist=193296		1
3M	rs141754456	12	19998198	0.798650026	T	C	421	0.007126	7	2.240311418	0.429474084	5.21640654	1.82E-07	12.14603335	2.328429207	intergenic	AEBP2;LINC02398	dist=475971;dist=16487		1
3M	rs117498042	6	165100792		C	T	421	0.003563	3	1.405430664	0.269604623	5.212932361	1.86E-07	19.33547097	3.70913521	intergenic	MEAT6;C6orf118	dist=278729;dist=178872		1
3M	rs184487573	6	167099983		A	G	421	0.007126	7	2.008477086	0.385701261	5.207338654	1.92E-07	13.50096351	2.592680141	intergenic	CEP43;CCR6	dist=47265;dist=11824		1

3M	rs149298750	4	126237546		A	C	421	0.003563	3	1.219140188	0.234426329	5.200525875	1.99E-07	22.18405204	4.265732461	intergenic	MIR2054;INTU	dist=730239;dist=1395411		1
3M	rs140277951	10	80599344		G	A	421	0.008314	6	1.967344821	0.378309358	5.200359916	1.99E-07	13.74631584	2.643339318	intronic	SH2D4B	.		1
3M	rs558553658	2	15542600	0.761030018	C	T	421	0.004751	3	1.579685756	0.303851567	5.198873167	2.01E-07	17.10991069	3.291080613	intronic	NBAS	.		1
3M	rs112679237	4	138219706	0.778339982	T	C	421	0.017815	18	3.098512369	0.596371661	5.195606321	2.04E-07	8.712027524	1.676806707	intronic	SLC7A11	.		1
3M	rs116862847	14	63674959	0.740740001	C	T	421	0.007126	7	1.757642639	0.338759972	5.188460225	2.12E-07	15.3160369	2.951942625	intergenic	WDR89;SGPP1	dist=33088;dist=9258		1
3M	rs528809914	13	112386942		G	A	421	0.003563	3	1.373302637	0.265345521	5.17552597	2.27E-07	19.50485519	3.768671108	intronic	SPACA7	.		1
3M	rs112007361	11	99317649		A	C	421	0.030879	26	4.213518738	0.814161472	5.175286336	2.28E-07	6.356584678	1.228257582	intronic	CNTN5	.		1
3M	rs546144116	19	19452530	0.741270006	C	T	421	0.002375	3	1.157037115	0.22363222	5.173839072	2.29E-07	23.13548147	4.471627577	intronic	GATAD2A	.		1
3M	rs9643828	8	54616513		C	T	421	0.67696	279	-11.04343772	2.134612444	-5.173509483	2.30E-07	-2.423629403	0.468469114	intronic	RP1	.		1
3M	rs17510814	12	28316036		A	C	421	0.007126	6	1.905807603	0.368446684	5.17254649	2.31E-07	14.03879235	2.714096892	intronic	CCDC91	.		1
3M	rs113167689	12	28283029		C	T	421	0.007126	6	1.892111617	0.365850586	5.171815182	2.32E-07	14.13641354	2.733356286	intronic	CCDC91	.		1
3M	rs140788628	20	15877856		C	A	421	0.010689	8	2.265436379	0.438089306	5.171174799	2.33E-07	11.80392839	2.282639604	intronic	MACROD2	.		1
3M	rs141756120	12	28358163		A	C	421	0.008314	6	1.868183897	0.361717894	5.164753879	2.41E-07	14.27840304	2.764585375	intronic	CCDC91	.		1
3M	rs117991215	12	28358540		T	C	421	0.008314	6	1.855759228	0.359817411	5.15750259	2.50E-07	14.33366601	2.77918736	intronic	CCDC91	.		1
3M	rs191423619	4	125595472	0.741770029	G	T	421	0.002375	3	1.225954306	0.23771753	5.157189324	2.51E-07	21.69461097	4.206673365	intergenic	MIR2054;INTU	dist=88165;dist=2037485		1
3M	rs113651406	4	997179		C	T	421	0.003563	3	1.369997433	0.265704278	5.156098506	2.52E-07	19.40540264	3.763582603	intronic	IDUA	.		1
3M	rs187942235	18	29450465		C	T	421	0.003563	3	1.294384565	0.251912631	5.138228126	2.77E-07	20.39686581	3.969630251	intergenic	CDH2;MIR302F	dist=1273336;dist=848446		1
3M	rs16920698	8	54765874		G	A	421	0.330166	277	11.42894943	2.226765953	5.132532863	2.86E-07	2.304926953	0.449081772	intronic	RP1	.		1
3M	rs4737676	8	54766986		G	A	421	0.330166	277	11.42891023	2.226765217	5.132516953	2.86E-07	2.304920569	0.44908192	intronic	RP1	.		1
3M	rs4737674	8	54749094		C	A	421	0.330166	277	11.42523599	2.226081333	5.132443196	2.86E-07	2.305595541	0.449219885	intronic	RP1	.		1
3M	rs13277510	8	54761589		G	A	421	0.330166	277	11.42815338	2.22667847	5.132377007	2.86E-07	2.304947516	0.449099416	intronic	RP1	.		1
3M	rs983248	8	54768232		C	T	421	0.330166	277	11.43612477	2.228588003	5.131556283	2.87E-07	2.302604284	0.448714611	intronic	RP1	.		1
3M	rs1391463	8	54769316		T	G	421	0.330166	277	11.43612548	2.228595636	5.131539026	2.87E-07	2.302588654	0.448713075	intronic	RP1	.		1
3M	rs4737201	8	54778898		C	T	421	0.330166	277	11.43417402	2.228701352	5.130420012	2.89E-07	2.301977341	0.44869179	intronic	RP1	.		1
3M	rs549931083	12	20363352		A	C	421	0.003563	3	1.829003959	0.357281438	5.119224688	3.07E-07	14.32826937	2.798913946	intergenic	LINC02468;PDE3A	dist=235451;dist=5185		1
3M	rs141281289	11	123822316	0.733070016	A	G	421	0.005938	6	1.657109443	0.323734372	5.118731856	3.08E-07	15.81151802	3.088952198	intergenic	OR6M1;TMEM225	dist=15967;dist=60603		1
3M	rs184220112	2	48404604		C	A	421	0.004751	4	1.587567329	0.310301123	5.116215225	3.12E-07	16.48790432	3.22267606	intergenic	FOXN2;PPP1R21	dist=25309;dist=36162		1
3M	rs11987234	8	54757269		A	G	421	0.328979	276	11.39664661	2.227939022	5.115331478	3.13E-07	2.295992587	0.448845319	intronic	RP1	.		1
3M	rs13276543	8	54775614		G	T	421	0.328979	276	11.41118163	2.230992831	5.114844597	3.14E-07	2.29263157	0.448230934	intronic	RP1	.		1
3M	rs111838310	2	74446364	0.780420005	C	A	421	0.004751	5	1.465896314	0.287002463	5.107608836	3.26E-07	17.79639376	3.48429066	intergenic	RTKN;INO80B-WBP1	dist=4427;dist=8659		1
3M	rs80156375	7	18403592		A	C	421	0.009501	8	2.260074467	0.442534058	5.107119835	3.27E-07	11.5406255	2.25971308	intronic	HDAC9	.		1
3M	rs1561297	8	54765978		A	C	421	0.332542	279	11.40464743	2.234979191	5.102798037	3.35E-07	2.28315237	0.447431459	intronic	RP1	.		1
3M	rs112983626	2	74470023	0.782959998	G	A	421	0.004751	4	1.464072657	0.286917293	5.102768952	3.35E-07	17.78480792	3.485324945	intergenic	MOGS;MRPL53	dist=4641;dist=1959		1
3M	rs12548593	8	54762057		G	T	421	0.332542	279	11.39766972	2.234516943	5.100730946	3.38E-07	2.282699606	0.447524018	intronic	RP1	.		1
3M	rs10105693	8	54727912		C	T	421	0.328979	276	11.34015363	2.223938019	5.099132049	3.41E-07	2.292839101	0.449652819	intronic	RP1	.		1
3M	rs13278605	8	54775611		C	T	421	0.328979	277	11.43432709	2.242496155	5.09892829	3.42E-07	2.273773481	0.445931645	intronic	RP1	.		1
3M	rs2083123	8	54767758		C	T	421	0.332542	279	11.40261082	2.236761629	5.097821184	3.44E-07	2.279107938	0.447074908	intronic	RP1	.		1
3M	rs17017794	4	90904734		T	C	421	0.039192	33	4.452559757	0.873737599	5.095991934	3.47E-07	5.83240545	1.144508375	intronic	CCSER1	.		1
3M	rs74521112	11	99218416		G	T	421	0.032067	27	4.149638813	0.814414575	5.095241344	3.48E-07	6.256323868	1.227875864	intronic	CNTN5	.		1
3M	rs116651654	4	162317591	0.708450019	C	T	421	0.007126	6	1.661626207	0.326131851	5.094952243	3.49E-07	15.62236937	3.066244515	intergenic	FSTL5;MIR4454	dist=153557;dist=775983		1
3M	rs565682685	9	90460046		T	C	421	0.004751	4	1.368085345	0.268772597	5.090122138	3.58E-07	18.93839699	3.720617399	intergenic	LINC01508;LINC01501	dist=26557;dist=2386		1
3M	rs144541665	1	103768107		G	A	421	0.002375	3	1.181121027	0.232374488	5.082834329	3.72E-07	21.87346108	4.303398392	intergenic	AMY1C;LOC100129138	dist=9415;dist=304916		1
3M	rs193253461	15	58937154	0.781300008	A	G	421	0.013064	12	2.449615619	0.482149005	5.080619466	3.76E-07	10.53744676	2.074047629	intergenic	SLTM;RNF111	dist=3475;dist=50509		1
3M	rs77867199	7	18402652		G	T	421	0.010689	9	2.377239679	0.468684022	5.072158572	3.93E-07	10.82212821	2.13363365	intronic	HDAC9	.		1
3M	rs189360484	12	1761344		A	G	421	0.004751	4	1.530180886	0.301727521	5.071399785	3.95E-07	16.8078794	3.314248553	intronic	ADIPOR2	.		1
3M	rs79182806	7	18394204		T	C	421	0.008314	7	2.11859485	0.418485528	5.062528352	4.14E-07	12.09726027	2.389568893	intronic	HDAC9	.		1
3M	rs189890455	2	48428258		C	T	421	0.005938	5	1.690230255	0.334051451	5.059790188	4.20E-07	15.14673914	2.993550834	intergenic	FOXN2;PPP1R21	dist=48963;dist=12508		1
3M	rs190294315	9	83330550		C	T	421	0.005938	5	1.754632626	0.347069598	5.055564181	4.29E-07	14.56642764	2.881266486	intronic	FRMD3	.		1
3M	rs185819304	18	29421615		G	A	421	0.003563	3	1.281236388	0.25348417	5.05450257	4.32E-07	19.94011134	3.945019527	intergenic	CDH2;MIR302F	dist=1244486;dist=877296		1
3M	rs148433854	19	30605571		G	A	421	0.003563	3	1.369429098	0.271144709	5.050547011	4.41E-07	18.62675851	3.688067545	intronic	ZNF536	.		1
3M	rs576124203	3	142122354		T	G	421	0.003563	3	1.313230925	0.260150702	5.047962263	4.47E-07	19.4039925	3.843925823	intronic	TFDP2	.		1
3M	rs143597860	1	103614521		A	G	421	0.002375	3	1.179951068	0.233861351	5.045515482	4.52E-07	21.57481541	4.276037896	intergenic	AMY2B;AMY2A	dist=34987;dist=2811		1
3M	rs140782222	9	81413979	0.778829992	T	C	421	0.002375	3	1.1680569	0.231519821	5.045170192	4.53E-07	21.79152597	4.31928461	intergenic	LINC01507;TLE1	dist=1379424;dist=169704		1
3M	rs1856085	1	103571923		G	A	421	0.002375	3	1.181208876	0.234330387	5.040784048	4.64E-07	21.51143997	4.267478979	intronic	AMY2B	.		1
3M	rs186768950	19	18695314		C	A	421	0.002375	3	1.117877431	0.221839436	5.039128522	4.68E-07	22.71520611	4.507764788	intronic	CRTC1	.		1
3M	rs182303755	15	59342593		A	C	421	0.013064	12	2.406496876	0.477647778	5.038224785	4.70E-07	10.54799166	2.093592905	intronic	MYO1E	.		1
3M	rs545552231	3	142145508		C	T	421	0.003563	3	1.275833022	0.253432385	5.034214646	4.80E-07	19.8641332	3.945825635	intronic	TFDP2	.		1
3M	rs78547898	22	32428291	0.730069995	G	A	421	0.003563	3	1.210956763	0.240651719	5.03198883	4.85E-07	20.90983951	4.155382737	intronic	BPIFC	.		1
3M	rs78907958	7	18385394		T	G	421	0.008314	7	2.090002735	0.415774551	5.026769271	4.99E-07	12.09013217	2.405149614	intronic	HDAC9	.		1
3M	rs113751774	4	23427231	0.70836997	C	T	421	0.007126	7	1.694246649	0.337089114	5.026109061	5.01E-07	14.91032743	2.966574592	intergenic	GBA3;PPARGC1A	dist=607659;dist=364790		1
3M	rs10958428	8	54773081		A	G	421	0.333729	280	11.27671394	2.243728439	5.025881807	5.01E-07	2.239968848	0.445686734	intronic	RP1	.		1
3M	rs17776100	7	6386848		G	A	421	0.029691	25	3.798893519	0.756560221	5.021270496	5.13E-07	6.63697397	1.321771845	intronic	RAC1	.		1
3M	rs185464792	19	18686561		C	T	421	0.002375	3	1.114587174	0.222127708	5.017776414	5.23E-07	22.58960154	4.501914727	intronic	CRTC1	.		1
3M	rs117025967	10	19918123		C	A	421	0.013064	11	2.602875018	0.518823163	5.016882829	5.25E-07	9.669735635	1.927439002	intronic	PLXDC2	.		1
3M	rs188028357	7	101025144		C	T	421	0.002375	3	1.150125852	0.229467369	5.012154263	5.38E-07	21.84255775	4.357918094	intronic	MUC17	.		1
3M	rs148556485	9	81408911	0.779910028	A	C	421	0.002375	3	1.17470554	0.234401563	5.011508991	5.40E-07	21.38001525	4.266183157	intergenic	LINC01507;TLE1	dist=1374356;dist=174772		1
3M	rs184613584	17	50430860		A	C	421	0.007126	6	1.77626107	0.354456155	5.011229298	5.41E-07	14.13779737	2.821223402	intronic	ACSF2	.		1
3M	rs528288879	2	65648796	0.785229981	C	T	421	0.005938	6	1.697856008	0.338843001	5.010745399	5.42E-07	14.78780847	2.951219288	intergenic	SPRED2;MIR4778	dist=216197;dist=709451		1
3M	rs1437782	8	54720202		C	T	421	0.330166	278	11.10161994	2.217354849	5.006695229	5.54E-07	2.2579585	0.450987807	exonic	RP1	.		1
3M	rs534845494	13	57639730		A	G	421	0.004751	5	1.571808362	0.313987509	5.005958262	5.56E-07	15.94317648	3.184840074	intronic	PCDH17	.		1
3M	rs1008091735	19	30599192		T	C	421	0.003563	3	1.355816116	0.270944036	5.004044884	5.61E-07	18.46892428	3.690799086	intronic	ZNF536	.		1
3M	rs567982164	5	25631188	0.711179972	G	A	421	0.002375	3	1.14494858	0.228986639	5.000067194	5.73E-07	21.8356286	4.367067031	intergenic	LINC02211;CDH9	dist=328908;dist=1249409		1
3M	rs79213709	11	99222724		G	A	421	0.032067	26	4.048879944	0.810020577	4.998490236	5.78E-07	6.17081884	1.23453654	intronic	CNTN5	.		1
3M	rs559008174	19	18765249	0.769429982	C	T	421	0.004751	5	1.594517191	0.319680825	4.987841211	6.11E-07	15.60256615	3.12812006	intronic	CRTC1	.		1
3M	rs541288561	19	18758635	0.77651	T	G	421	0.004751	5	1.591688559	0.31930164	4.984905675	6.20E-07	15.61190124	3.131834835	intronic	CRTC1	.		1
3M	rs190251199	14	105124240	0.736329973	T	C	421	0.003563	3	1.240668633	0.248987137	4.982862353	6.27E-07	20.01252919	4.016271726	intergenic	LINC02298;JAG2	dist=24744;dist=16758		1
3M	rs138215817	14	22173619		A	G	421	0.004751	4	1.521988034	0.305467544	4.982486896	6.28E-07	16.31101896	3.273670217	intergenic	OR4E1;LOC105370401	dist=502284;dist=206292		1
3M	rs111407636	5	95744325		C	T	421	0.003563	3	1.331610247	0.26725959	4.98246012	6.28E-07	18.64277396	3.741680518	intronic	RHOBTB3	.		1
3M	rs73085348	3	42669729		A	G	421	0.011876	11	2.448275953	0.491389455	4.982353469	6.28E-07	10.13931704	2.035045707	intergenic	ZBTB47;KLHL40	dist=2149;dist=15808		1
3M	rs2375537	8	54706948		C	T	421	0.332542	280	11.08575521	2.225246111	4.981810848	6.30E-07	2.238768477	0.449388495	intronic	RP1	.		1
3M	rs1437781	8	54717292		T	C	421	0.332542	280	11.08371192	2.225151228	4.981105007	6.32E-07	2.23854673	0.449407657	intronic	RP1	.		1
3M	rs2274997	1	229668899		A	G	421	0.041568	35	4.42362483	0.889790308	4.971536315	6.64E-07	5.587312281	1.123860297	intergenic	URB2;LINC01682	dist=8699;dist=206651		1
3M	rs147032554	1	186179732		T	G	421	0.003563	3	1.360299196	0.273670575	4.970571635	6.68E-07	18.16260897	3.654028209	intronic	HMCN1	.		1
3M	rs111676272	5	95758594		C	A	421	0.003563	3	1.327188035	0.267072797	4.969386812	6.72E-07	18.6068625	3.744297477	intronic	RHOBTB3	.		1
3M	rs2274996	1	229668791		C	T	421	0.041568	35	4.420411647	0.88962935	4.968823976	6.74E-07	5.585274332	1.124063633	intergenic	URB2;LINC01682	dist=8591;dist=206759		1
3M	rs2891865	1	229670621		A	G	421	0.041568	35	4.419830983	0.88984663	4.966958165	6.80E-07	5.581813763	1.123789164	intergenic	URB2;LINC01682	dist=10421;dist=204929		1
3M	rs2385790	1	229671745		C	T	421	0.041568	35	4.418931852	0.88972149	4.966646197	6.81E-07	5.582248215	1.123947226	intergenic	URB2;LINC01682	dist=11545;dist=203805		1
3M	rs12024557	1	229676610		A	C	421	0.042755	35	4.42434228	0.891299524	4.963923079	6.91E-07	5.569309691	1.121957291	intergenic	URB2;LINC01682	dist=16410;dist=198940		1
3M	rs78296164	9	132391328		C	T	421	0.008314	7	2.121725249	0.427501046	4.963087859	6.94E-07	11.60953385	2.339175566	intronic	TTF1	.		1
3M	rs570407448	19	18769220	0.776769996	G	A	421	0.004751	5	1.587863075	0.320089569	4.960683605	7.02E-07	15.4977984	3.124125552	intronic	CRTC1	.		1
3M	rs76777840	2	48085811		G	A	421	0.005938	5	1.714828291	0.345687108	4.960637102	7.03E-07	14.35007842	2.892789399	intergenic	FBXO11;FOXN2	dist=179313;dist=228488		1

3M	rs528609331	11	125972300		C	T	421	0.003563	3	1.256070429	0.253271825	4.959376858	7.07E-07	19.58124182	3.948327054	intronic	CDON	.		1
3M	rs182437250	12	63214686	0.713500023	T	C	421	0.004751	4	1.307028829	0.263613284	4.958129612	7.12E-07	18.80834508	3.793435539	intergenic	AVPR1A;DPY19L2	dist=63485;dist=344227		1
3M	rs147601511	8	22464806		A	G	421	0.004751	4	1.494160188	0.301407236	4.957280417	7.15E-07	16.44711814	3.317770381	intronic	PPP3CC	.		1
3M	rs858397	8	54702130		A	G	421	0.331354	278	11.01774054	2.2226993	4.956919066	7.16E-07	2.230134803	0.449903412	intronic	RP1	.		1
3M	rs75334617	10	101196395		G	A	421	0.038005	32	4.196585386	0.847261284	4.953118316	7.30E-07	5.846034048	1.180273451	intergenic	LINC01514;LBX1	dist=2248;dist=30581		1
3M	rs4562666	1	229689023		T	C	421	0.042755	36	4.44283225	0.897203314	4.951867853	7.35E-07	5.519225993	1.114574572	intergenic	URB2;LINC01682	dist=28823;dist=186527		1
3M	rs382476	8	54678415		G	A	421	0.666271	288	-10.90475865	2.202374434	-4.951364528	7.37E-07	-2.24819379	0.454055398	intronic	RP1	.		1
3M	rs384543	8	54679049		G	A	421	0.666271	288	-10.90475865	2.202374434	-4.951364528	7.37E-07	-2.24819379	0.454055398	intronic	RP1	.		1
3M	rs446222	8	54662400		G	A	421	0.666271	288	-10.90656484	2.202740432	-4.951361803	7.37E-07	-2.247819003	0.453979954	intronic	RP1	.		1
3M	rs384127	8	54684929		G	A	421	0.666271	288	-10.90492469	2.202409773	-4.951360471	7.37E-07	-2.248155875	0.454048112	intronic	RP1	.		1
3M	rs12045643	1	229698303		C	T	421	0.042755	36	4.472324416	0.903269355	4.951263308	7.37E-07	5.481491517	1.107089479	intergenic	URB2;LINC01682	dist=38103;dist=177247		1
3M	rs147630370	4	86529522		T	C	421	0.004751	4	1.530518883	0.309272178	4.948776482	7.47E-07	16.00136328	3.23339786	intergenic	MAPK10;MIR4452	dist=76327;dist=12960		1
3M	rs532513136	8	134801505		C	A	421	0.003563	3	1.227599638	0.248065954	4.948682463	7.47E-07	19.94905941	4.031185989	upstream	MIR30B	dist=898		1
3M	rs184265355	13	107359004		A	C	421	0.005938	6	1.694033349	0.342665495	4.94369399	7.67E-07	14.42717187	2.918297916	intronic	FAM155A	.		1
3M	rs148248743	3	136415753	0.708679974	C	T	421	0.002375	3	1.069833829	0.216429281	4.943110391	7.69E-07	22.83937904	4.620446891	intronic	STAG1	.		1
3M	rs72983831	6	141985963	0.75375998	T	G	421	0.005938	6	1.556807425	0.31516421	4.939670734	7.83E-07	15.67332386	3.17294911	intergenic	MIR4465;NMBR	dist=1302080;dist=88521		1
3M	rs80292573	15	59142887		T	G	421	0.034442	30	3.721656224	0.753598905	4.938510655	7.87E-07	6.553234912	1.326965834	intronic	MYO1E	.		1
3M	rs111927235	2	74256827		A	G	421	0.004751	4	1.423024126	0.288923909	4.925255689	8.43E-07	17.04689553	3.461118894	intronic	SLC4A5	.		1
3M	rs369623	8	54659380		A	C	421	0.666271	287	-10.8407396	2.201888659	-4.923382276	8.51E-07	-2.235981486	0.45415557	intronic	RP1	.		1
3M	rs188720948	3	150352093		T	C	421	0.003563	3	1.368098189	0.277991774	4.921362129	8.59E-07	17.7032653	3.597228742	intergenic	LINC01214;TSC22D2	dist=28346;dist=56205		1
3M	rs148998974	22	40224526		A	G	421	0.005938	5	1.667947442	0.339010604	4.920045046	8.65E-07	14.51295325	2.949760239	intronic	TNRC6B	.		1
3M	rs144954214	16	76145464		A	G	421	0.002375	3	1.106696587	0.225254382	4.913096823	8.96E-07	21.81132631	4.439425294	intergenic	CPHXL;CNTNAP4	dist=418974;dist=131937		1
3M	rs145439370	15	58587566		T	C	421	0.030879	26	3.500007735	0.712559708	4.911879937	9.02E-07	6.893288912	1.403391166	intergenic	LIPC;ADAM10	dist=17722;dist=1243		1
3M	rs75689761	7	18366950		C	T	421	0.008314	7	2.0345491	0.414387092	4.909779146	9.12E-07	11.84829172	2.413202584	intronic	HDAC9	.		1
3M	rs113221952	1	103288418		A	G	421	0.021378	19	3.040841012	0.619495467	4.908576695	9.17E-07	7.923507043	1.614216816	intergenic	COL11A1;LOC101928436	dist=179896;dist=205628		1
3M	rs61434999	7	18378728		A	G	421	0.008314	7	2.033933624	0.414449316	4.907556958	9.22E-07	11.84115106	2.412840272	intronic	HDAC9	.		1
3M	rs433324	8	54652049		A	G	421	0.666271	287	-10.71178821	2.182726874	-4.907525693	9.22E-07	-2.248346209	0.458142524	intronic	RP1	.		1
3M	rs528140343	11	125849332		A	C	421	0.003563	3	1.315991741	0.268284941	4.905201681	9.33E-07	18.28355207	3.727380292	intergenic	PATE4;HYLS1	dist=9260;dist=34282		1
3M	rs34270375	1	88905019		G	A	421	0.026128	21	3.119890277	0.63606437	4.904991419	9.34E-07	7.711470175	1.572167924	intergenic	GTF2B;KYAT3	dist=13452;dist=30754		1
3M	rs75773869	7	18371222		G	T	421	0.008314	7	2.02750066	0.413389996	4.904571175	9.36E-07	11.86427156	2.41902322	intronic	HDAC9	.		1
3M	rs79602997	7	18370627		G	A	421	0.008314	7	2.026922666	0.413391185	4.903158896	9.43E-07	11.86082112	2.419016265	intronic	HDAC9	.		1
3M	rs56224400	6	97644799		T	C	421	0.016627	14	2.845683986	0.580546566	4.901732531	9.50E-07	8.443306398	1.722514712	ncRNA_intronic	LOC101927314	.		1
3M	rs186532456	1	18621834		C	T	421	0.003563	3	1.189953152	0.242873045	4.899486277	9.61E-07	20.17303433	4.117377453	intergenic	KLHDC7A;PAX7	dist=135848;dist=9012		1
3M	rs118093638	11	18696777		C	T	421	0.005938	5	1.681215257	0.343292823	4.897321308	9.72E-07	14.26572587	2.912965064	intergenic	SPTY2D1;TMEM86A	dist=62435;dist=2002		1
3M	rs77353774	12	28095919		G	A	421	0.007126	6	1.82528489	0.373198242	4.890925749	1.00E-06	13.1054362	2.679541026	intergenic	PTHLH;LOC729291	dist=123186;dist=89706		1
3M	rs532730683	9	1784492		G	T	421	0.003563	3	1.275657724	0.260996603	4.887641107	1.02E-06	18.72683804	3.831467497	intergenic	DMRT2;SMARCA2	dist=726938;dist=230855		1
3M	rs185139807	22	40198777		G	A	421	0.005938	5	1.65498472	0.338624126	4.887379825	1.02E-06	14.43305263	2.953126858	intronic	TNRC6B	.		1
3M	rs146333745	18	57830225	0.779890001	C	T	421	0.003563	4	1.368613476	0.280282935	4.882971117	1.04E-06	17.42157837	3.567823349	intergenic	ATP8B1;NEDD4L	dist=26910;dist=214001		1
3M	rs541680196	22	40132086		G	A	421	0.005938	5	1.654258232	0.339093792	4.878468054	1.07E-06	14.38678079	2.949036589	intronic	TNRC6B	.		1
3M	rs12036586	1	229690631		G	A	421	0.047506	40	4.551491816	0.933583971	4.875289164	1.09E-06	5.222121756	1.071140927	intergenic	URB2;LINC01682	dist=30431;dist=184919		1
3M	rs529523094	16	77681654		A	G	421	0.003563	3	1.307832421	0.26837178	4.873211417	1.10E-06	18.15843462	3.726174193	intergenic	ADAMTS18;NUDT7	dist=246620;dist=40838		1
3M	rs562032622	1	18632839		A	C	421	0.003563	3	1.126818759	0.231494862	4.867575676	1.13E-06	21.0267115	4.319750303	intronic	PAX7	.		1
3M	rs78225611	7	18367841		A	C	421	0.009501	8	2.147570798	0.441262518	4.866877908	1.13E-06	11.02943875	2.266224663	intronic	HDAC9	.		1
3M	rs77346868	7	18366976		A	G	421	0.009501	8	2.148608551	0.441537806	4.866193838	1.14E-06	11.02101286	2.264811725	intronic	HDAC9	.		1
3M	rs184098071	2	176251692		G	A	421	0.003563	3	1.695892029	0.34869637	4.863520744	1.15E-06	13.94772404	2.86782452	intergenic	HOXD1;MTX2	dist=60785;dist=17750		1
3M	rs118183140	21	34105187		C	T	421	0.02019	17	3.113677552	0.640523765	4.861142898	1.17E-06	7.589324801	1.561222322	UTR3	SLC5A3	NM_006933:c.*78320>0		1
3M	rs113625788	22	19981659		C	T	421	0.008314	7	2.000739147	0.411621625	4.860626906	1.17E-06	11.80848286	2.429415606	exonic	ARVCF	.		1
3M	rs116189766	2	125636287	0.723290026	T	C	421	0.002375	3	1.114549479	0.229338184	4.85985133	1.17E-06	21.19076397	4.360372887	intergenic	CNTNAP5;LINC01941	dist=715069;dist=473813		1
3M	rs76098744	1	111806750		C	T	421	0.016627	14	2.811615503	0.579024995	4.855775707	1.20E-06	8.386124522	1.727041163	intronic	KCND3	.		1
3M	rs74683551	1	111811796		G	A	421	0.016627	14	2.810351968	0.579019309	4.853641189	1.21E-06	8.38252043	1.727058121	intronic	KCND3	.		1
3M	rs185855183	12	101111348		C	T	421	0.003563	3	1.256842073	0.258975211	4.853136594	1.22E-06	18.73977272	3.861373435	intronic	ANO4	.		1
3M	rs405226	8	54679776		A	G	421	0.662708	291	-10.75682372	2.217202771	-4.851529081	1.23E-06	-2.188130533	0.45101874	intronic	RP1	.		1
3M	rs3098298	8	54670278		C	T	421	0.662708	291	-10.75741494	2.217551047	-4.851033737	1.23E-06	-2.187563503	0.450947906	intronic	RP1	.		1
3M	rs367179	8	54675056		T	C	421	0.662708	291	-10.75741494	2.217551047	-4.851033737	1.23E-06	-2.187563503	0.450947906	intronic	RP1	.		1
3M	rs432393	8	54667738		C	T	421	0.662708	291	-10.75654812	2.217474543	-4.850810194	1.23E-06	-2.187538166	0.450963464	intronic	RP1	.		1
3M	rs117185941	21	37394182		G	A	421	0.005938	5	1.529223098	0.31531516	4.849824212	1.24E-06	15.38087864	3.171430132	intronic	DYRK1A	.		1
3M	rs139360368	5	74076284		A	C	421	0.003563	4	1.280137576	0.264060375	4.847897286	1.25E-06	18.35904869	3.78701272	intergenic	ARHGEF28;LINC01335	dist=134291;dist=230126		1
3M	rs184117160	15	59112107	0.770810008	C	T	421	0.014252	11	2.271648443	0.468677492	4.846933088	1.25E-06	10.34172362	2.133663377	intronic	CCNB2	.		1
3M	rs145676540	3	2002951		C	T	421	0.005938	5	1.68032939	0.346683687	4.846866048	1.25E-06	13.98065798	2.884473769	intergenic	CNTN6;CNTN4	dist=598734;dist=95852		1
3M	rs556293455	2	176085692		G	A	421	0.003563	3	1.630922146	0.336702149	4.843812709	1.27E-06	14.38604633	2.969984018	intergenic	EVX2;HOXD13	dist=1730;dist=7029		1
3M	rs150027952	9	100623134	0.704400003	A	G	421	0.002375	3	1.072594942	0.221501288	4.842386925	1.28E-06	21.86166485	4.514646431	intergenic	CAVIN4;PLPPR1	dist=34747;dist=405593		1
3M	rs138217865	15	93849653		C	T	421	0.004751	4	1.402299902	0.28969226	4.840653664	1.29E-06	16.70964097	3.451938959	intergenic	LOC105370980;LINC02207	dist=641605;dist=6907		1
3M	rs112475378	3	1584977	0.749329984	T	C	421	0.016627	14	2.445371748	0.506000821	4.832742644	1.35E-06	9.550859286	1.976281377	intergenic	CNTN6;CNTN4	dist=180760;dist=513826		1
3M	rs146007933	3	28185932		T	C	421	0.021378	18	3.108745063	0.643323442	4.832320511	1.35E-06	7.511494525	1.554428045	intergenic	LINC01980;CMC1	dist=325607;dist=55687		1
3M	rs192443987	3	135813503		G	A	421	0.004751	4	1.31410558	0.27206124	4.830183004	1.36E-06	17.75402845	3.675643022	intergenic	EPHB1;PPP2R3A	dist=553038;dist=152225		1
3M	rs149493615	1	79414404		G	A	421	0.008314	8	1.98260972	0.410759865	4.826688019	1.39E-06	11.750632	2.434512435	intergenic	ADGRL4;LINC01781	dist=407674;dist=1121351		1
3M	rs140706881	10	94457778		G	A	421	0.004751	4	1.226743807	0.254265992	4.824647601	1.40E-06	18.97480496	3.932889306	intronic	TBC1D12	.		1
3M	rs142894171	2	150581757		G	T	421	0.003563	3	1.287888043	0.266960796	4.82425907	1.41E-06	18.07103941	3.74586836	intergenic	LINC01920;LINC02612	dist=9536;dist=47140		1
3M	rs17746486	2	95056864	0.71280998	C	T	421	0.032067	29	3.355977065	0.696974974	4.815061071	1.47E-06	6.908513578	1.434771746	intergenic	MAL;MRPS5	dist=2872;dist=28507		1
3M	rs180764936	12	101104843		T	C	421	0.003563	3	1.197190428	0.248754746	4.812734014	1.49E-06	19.34730528	4.020023799	intronic	ANO4	.		1
3M	rs190806532	12	48468364	0.728540003	G	T	421	0.002375	3	1.082541559	0.225298273	4.804926128	1.55E-06	21.32695498	4.438560431	intergenic	ZNF641;ANP32D	dist=117118;dist=4195		1
3M	rs147393020	10	123019758		A	G	421	0.005938	5	1.619245544	0.337859644	4.792657467	1.65E-06	14.1853505	2.959808958	intronic	ACADSB	.		1
3M	rs79539453	11	125396457		C	T	421	0.002375	3	1.102705134	0.230127137	4.791721423	1.65E-06	20.82206158	4.345424064	intronic	PKNOX2	.		1
3M	rs371245624	5	163453950		T	C	421	0.003563	3	1.17843308	0.245958889	4.791179069	1.66E-06	19.47959308	4.065720108	UTR3	NUDCD2	NM_001329991:c.*170>0;NM_145266:c.*170>0		1
3M	rs536803366	8	121989172	0.708639979	T	C	421	0.002375	3	1.194921552	0.249406377	4.791062547	1.66E-06	19.20986385	4.009520573	intergenic	HAS2-AS1;SMILR	dist=343847;dist=425155		1
3M	rs150077525	13	57405415	0.75563997	A	G	421	0.007126	7	1.765848692	0.368578785	4.790966716	1.66E-06	12.99848746	2.713124141	intergenic	PRR20E;PCDH17	dist=235197;dist=226329		1
3M	rs371879555	12	22863028	0.786769986	T	C	421	0.003563	3	1.139634583	0.238093921	4.786491727	1.70E-06	20.10337646	4.200023233	intergenic	ETNK1;LOC101928441	dist=172363;dist=312608		1
3M	rs571986619	6	84969114	0.767470002	A	G	421	0.002375	3	1.102004226	0.230254532	4.786026206	1.70E-06	20.78580673	4.343019831	intergenic	TBX18;LINC02535	dist=204516;dist=418105		1
3M	rs183586634	21	37390730		G	A	421	0.005938	5	1.598766374	0.334213375	4.783669629	1.72E-06	14.31322017	2.992100476	intronic	DYRK1A	.		1
3M	rs143811231	1	79502446		T	C	421	0.008314	8	1.975347907	0.413110429	4.78164619	1.74E-06	11.57474094	2.420660266	intergenic	ADGRL4;LINC01781	dist=495716;dist=1033309		1
3M	rs2365739	1	62018790		G	A	421	0.021378	18	3.317349453	0.694985007	4.773267649	1.81E-06	6.868159166	1.43887996	intronic	PATJ	.		1
3M	rs146526206	4	90071867		T	C	421	0.017815	15	2.815290022	0.58984771	4.772909977	1.82E-06	8.091766558	1.695352855	intergenic	MMRN1;CCSER1	dist=117257;dist=55527		1
3M	rs567383525	8	114017265		C	T	421	0.003563	3	1.282541719	0.268838431	4.770678479	1.84E-06	17.74552267	3.719706274	intergenic	CSMD3;TRPS1	dist=580326;dist=1391230		1
3M	rs149425014	15	58659461	0.716620028	T	C	421	0.026128	22	3.013015463	0.631629727	4.770224288	1.84E-06	7.552247919	1.583206043	intronic	ADAM10	.		1
3M	rs559559983	1	246813830		C	A	421	0.007126	5	1.645339674	0.345154188	4.76697004	1.87E-06	13.81113197	2.897255878	intergenic	LINC01341;AHCTF1	dist=22344;dist=25268		1
3M	rs117166500	7	17013154		G	T	421	0.007126	7	1.813506071	0.380670761	4.763975219	1.90E-06	12.51468647	2.626941975	intergenic	AGR3;AHR	dist=131171;dist=285498		1
3M	rs145766563	1	185160370		G	A	421	0.003563	4	1.34508864	0.282384562	4.76332215	1.90E-06	16.86821019	3.541270076	intronic	SWT1	.		1
3M	rs540065886	9	2770228	0.797429979	T	C	421	0.003563	3	1.13736568	0.23877586	4.763319367	1.90E-06	19.94891509	4.188028045	intergenic	KCNV2;PUM3	dist=40191;dist=33927		1
3M	rs118084887	21	37491518		T	C	421	0.005938	5	1.518241868	0.31880157	4.762341255	1.91E-06	14.93826162	3.136747415	intronic	DYRK1A	.		1
3M	rs140420703	1	102337928	0.700510025	T	G	421	0.004751	5	1.375529427	0.288838786	1.375529427	1.91E-06	16.48765399	3.462138915	intergenic	OLFM3;COL11A1	dist=340694;dist=538539		1
3M	rs568321148	2	29647482		T	G	421	0.003563	3	1.279867135	0.268808164	4.761265858	1.92E-06	17.71250463	3.720125102	intronic	ALK	.		1

3M	rs55844051	7	23320744		T	C	421	0.003563	3	1.232383167	0.25888714	4.760310483	1.93E-06	18.3875896	3.862687038	intronic	IGF2BP3	.		1
3M	rs187236873	5	92234630		G	A	421	0.007126	6	1.81660653	0.381998785	4.755529603	1.98E-06	12.44906998	2.617809375	intergenic	ARRDC3-AS1;NR2F1-AS1	dist=813915;dist=1214615		1
3M	rs1812506	8	54763541		A	G	421	0.345606	291	10.64127813	2.239050947	4.752584188	2.01E-06	2.122588678	0.446617796	intronic	RP1	.		1
3M	rs539713344	7	100877165	0.772019982	G	A	421	0.002375	3	1.125805228	0.236990667	4.750420101	2.03E-06	20.04475604	4.219575451	intronic	SRRT	.		1
3M	rs536781978	2	29510815		A	G	421	0.003563	3	1.282866581	0.270080884	4.749934759	2.03E-06	17.58708236	3.702594509	intronic	ALK	.		1
3M	rs76327548	12	100789188		G	A	421	0.013064	11	2.458077916	0.517503975	4.749872531	2.04E-06	9.178427144	1.932352307	intergenic	GAS2L3;ANO4	dist=160900;dist=5588		1
3M	rs149421869	2	53256291		G	T	421	0.008314	8	1.92083422	0.404523583	4.748386252	2.05E-06	11.73821862	2.472043763	intergenic	MIR4431;ASB3	dist=553676;dist=413688		1
3M	rs75024143	21	21784226		G	T	421	0.014252	12	2.293452396	0.483452813	4.743901232	2.10E-06	9.812542411	2.068454196	ncRNA_intronic	LINC01425	.		1
3M	rs2375536	8	54728162		T	C	421	0.347981	292	10.56754692	2.229301935	4.740294148	2.13E-06	2.126358065	0.448570911	intronic	RP1	.		1
3M	rs141326851	6	134511989		A	C	421	0.016627	14	2.616968305	0.552323641	4.738106631	2.16E-06	8.578496884	1.810532677	intergenic	LINC01010;LOC101928304	dist=7969;dist=13329		1
3M	rs187518659	1	98990189		T	G	421	0.009501	8	1.921562765	0.405573328	4.737892339	2.16E-06	11.68196232	2.465645372	intronic	PLPPR5	.		1
3M	rs146479102	2	65598625	0.738789976	G	A	421	0.005938	5	1.448302352	0.305828916	4.735661917	2.18E-06	15.48467677	3.269801992	intergenic	SPRED2;MIR4778	dist=166026;dist=759622		1
3M	rs16850124	1	229695584		T	C	421	0.043943	37	4.326094288	0.91378287	4.73426941	2.20E-06	5.180956622	1.094351878	intergenic	URB2;LINC01682	dist=35384;dist=179966		1
3M	rs115348382	1	9595845		G	A	421	0.003563	3	1.230366386	0.259928152	4.733486451	2.21E-06	18.21074945	3.847216981	intronic	TMEM201	.		1
3M	rs184200893	2	69033781		C	T	421	0.003563	3	1.121211249	0.237019909	4.730451775	2.24E-06	19.95803558	4.219054866	intronic	ANTXR1	.		1
3M	rs148781275	11	103769875	0.774079978	A	G	421	0.004751	5	1.423809766	0.301038131	4.729665848	2.25E-06	15.71118528	3.321838325	intergenic	DYNC2H1;MIR4693	dist=290012;dist=80031		1
3M	rs139943877	3	155733500		G	A	421	0.007126	7	1.713092922	0.362264246	4.728849011	2.26E-06	13.0535902	2.760415942	intronic	PLCH1	.		1
3M	rs546409459	11	125885094		A	G	421	0.003563	3	1.180777655	0.249820856	4.726497517	2.28E-06	18.91954737	4.002868361	intronic	HYLS1	.		1
3M	rs186649043	3	175214503	0.769129992	C	T	421	0.003563	3	1.162234876	0.245948741	4.725516671	2.30E-06	19.21342086	4.065887859	intronic	NAALADL2	.		1
3M	rs80203220	3	123003484		C	T	421	0.005938	6	1.662045235	0.351841788	4.723842626	2.31E-06	13.42604201	2.842186558	intronic	SEMA5B	.		1
3M	rs541508507	2	169166755		G	A	421	0.004751	4	1.442660176	0.305446461	4.723119638	2.32E-06	15.46300334	3.273896179	intronic	LRP2	.		1
3M	rs529011661	10	20083387		G	A	421	0.004751	4	1.425272643	0.301883703	4.721263948	2.34E-06	15.63934688	3.312533901	intronic	PLXDC2	.		1
3M	rs182959028	22	45427152		T	C	421	0.008314	7	1.863907765	0.395028955	4.718407959	2.38E-06	11.94446103	2.531460004	intronic	RIBC2	.		1
3M	rs146207930	9	126280057		A	G	421	0.007126	6	1.87537986	0.397610416	4.716626584	2.40E-06	11.86243214	2.515024654	intergenic	LOC101929116;MVB12B	dist=4142;dist=46772		1
3M	rs290120	5	163841238		T	G	421	0.007126	6	1.664921355	0.353031552	4.716069562	2.40E-06	13.3587764	2.832608007	intergenic	MAT2B;LINC02143	dist=321884;dist=607184		1
3M	rs531769270	4	153489891	0.744459987	T	C	421	0.003563	3	1.339792194	0.284142497	4.715212285	2.41E-06	16.59453383	3.519360917	intronic	TMEM131L	.		1
3M	rs185620578	12	48175616	0.752129972	C	T	421	0.002375	3	1.072824946	0.227528468	4.715124023	2.42E-06	20.72322668	4.39505442	intergenic	ASB8;CCDC184	dist=18101;dist=8028		1
3M	rs62447184	7	36534898	0.730589986	G	A	421	0.042755	43	4.329544629	0.918817438	4.712083651	2.45E-06	5.128422094	1.088355486	intronic	AOAH	.		1
3M	rs118184666	12	20271815		G	A	421	0.007126	6	2.026245701	0.430149631	4.710560123	2.47E-06	10.95098026	2.324772421	intergenic	LINC02468;PDE3A	dist=143914;dist=96722		1
3M	rs10494861	1	205362746		G	A	421	0.003563	3	1.156337638	0.245524493	4.709663065	2.48E-06	19.18204982	4.072913404	intergenic	KLHDC8A;LEMD1-AS1	dist=5656;dist=10506		1
3M	rs183817723	16	59268871	0.79569	C	T	421	0.003563	4	1.261174136	0.267795557	4.709466243	2.48E-06	17.5860507	3.734191901	intergenic	GOT2;APOOP5	dist=534555;dist=485270		1
3M	rs12502861	4	2424578		T	C	421	0.010689	9	1.987294963	0.422008794	4.709131639	2.49E-06	11.15884718	2.369618867	intronic	CFAP99	.		1
3M	rs191792521	3	195919734	0.757189989	G	A	421	0.008314	6	1.587589638	0.337332296	4.706307863	2.52E-06	13.95154842	2.964435992	intergenic	TNK2-AS1;SDHAP1	dist=6470;dist=40187		1
3M	rs149949098	11	95366702		G	A	421	0.016627	13	2.587327209	0.549803064	4.705916312	2.53E-06	8.559276254	1.818833079	intergenic	LOC100129203;FAM76B	dist=132298;dist=402251		1
3M	rs74343174	5	162066176	0.766919971	C	A	421	0.005938	5	1.498573008	0.318559187	4.704221604	2.55E-06	14.76718237	3.139134082	intergenic	LINC01202;GABRG2	dist=64980;dist=1289		1
3M	rs189765693	4	4323066		T	C	421	0.004751	4	1.425732565	0.30309955	4.70384257	2.55E-06	15.51913413	3.299246074	intergenic	ZBTB49;NSG1	dist=1283;dist=63466		1
3M	rs189912648	5	135429749		C	T	421	0.003563	4	1.303529678	0.277126356	4.703737665	2.55E-06	16.97325991	3.608462274	intergenic	MACROH2A1;DCANP1	dist=29862;dist=14465		1
3M	rs151323346	12	20859090		T	C	421	0.005938	4	1.39062978	0.295827471	4.700813538	2.59E-06	15.89038883	3.380348678	intronic	LCO1B3;SLCO1B3-SLCO1B7	.		1
3M	rs536023430	7	147167980	0.788429976	T	C	421	0.003563	3	1.137129093	0.242081012	4.697308079	2.64E-06	19.40386834	4.130848564	intronic	CNTNAP2	.		1
3M	rs144026361	15	40956471	0.714779973	C	T	421	0.004751	5	1.365790883	0.290922074	4.694696631	2.67E-06	16.13729945	3.437346588	UTR3	CHAC1	NM_001142776:c.*6970>0;NM_024111:c.*6970>0		1
3M	rs186142189	2	67475575		G	A	421	0.007126	6	1.7290309	0.36845255	4.692682681	2.70E-06	12.73619271	2.714053683	intergenic	ETAA1;LINC01812	dist=63486;dist=320479		1
3M	rs142549310	2	169173996		C	T	421	0.004751	4	1.443948619	0.307707803	4.692596694	2.70E-06	15.25017126	3.249836338	exonic	LRP2	.		1
3M	rs72832764	5	170577669		G	A	421	0.003563	3	1.208783888	0.257601337	4.692459687	2.70E-06	18.21597569	3.881967434	intronic	KCNIP1	.		1
3M	rs183180157	1	181277985		A	C	421	0.009501	7	1.744207622	0.371856251	4.690542692	2.72E-06	12.61385999	2.689211211	intergenic	LINC01699;CACNA1E	dist=39381;dist=205532		1
3M	rs73227413	21	21764653		G	A	421	0.034442	28	3.687754962	0.78647158	4.688986931	2.75E-06	5.962055143	1.271501762	ncRNA_intronic	LINC01425	.		1
3M	rs185874707	8	18173165		C	T	421	0.011876	10	2.405517016	0.513034555	4.688801158	2.75E-06	9.139347655	1.949186444	intronic	NAT1	.		1
3M	rs423841	8	54643509		G	A	421	0.662708	291	-10.22664529	2.181175145	-4.688594272	2.75E-06	-2.149572575	0.458468456	intronic	RP1	.		1
3M	rs191271637	17	54045899	0.762179971	A	G	421	0.003563	3	1.195597009	0.255047807	4.687736869	2.76E-06	18.379836	3.920833552	intergenic	KIF2B;TOM1L1	dist=220706;dist=854792		1
3M	rs187978759	7	11672218		G	A	421	0.003563	3	1.07420489	0.229159549	4.687585114	2.76E-06	20.4555522	4.3637719	intronic	THSD7A	.		1
3M	rs193153124	3	148612923		A	G	421	0.005938	5	1.529063164	0.32624874	4.686801743	2.78E-06	14.36573132	3.065145937	intergenic	LINC02046;AGTR1	dist=212967;dist=84948		1
3M	rs1877768	6	16534692		C	T	421	0.017815	15	2.650393922	0.565512508	4.686711405	2.78E-06	8.287546847	1.768307483	intronic	ATXN1	.		1
3M	rs76617932	1	180961288		T	C	421	0.011876	9	2.08220138	0.444364012	4.685801107	2.79E-06	10.54496084	2.250407262	intergenic	KIAA1614-AS1;STX6	dist=6401;dist=11426		1
3M	rs188034471	9	83322799		G	A	421	0.004751	4	1.487832555	0.317535808	4.68555835	2.79E-06	14.75599992	3.149251127	intronic	FRMD3	.		1
3M	rs1595406	8	54718055		A	G	421	0.345606	291	10.45008066	2.230645943	4.684777831	2.80E-06	2.100188892	0.448300638	intronic	RP1	.		1
3M	rs147627638	6	98725240		A	G	421	0.007126	6	1.675781082	0.357955603	4.681533316	2.85E-06	13.07853062	2.793642539	intergenic	MIR2113;PNKY	dist=700621;dist=104901		1
3M	rs151272830	2	67455552		G	T	421	0.007126	6	1.643645432	0.351116105	4.681202055	2.85E-06	13.33234787	2.848060758	intergenic	ETAA1;LINC01812	dist=43503;dist=340462		1
3M	rs141169929	3	165090674	0.77117002	A	G	421	0.004751	5	1.473704308	0.315071386	4.677366376	2.91E-06	14.84541783	3.173883899	intergenic	SI;SLITRK3	dist=12178;dist=96046		1
3M	rs720372	8	54716077		G	A	421	0.346793	292	10.41667706	2.227853301	4.675656629	2.93E-06	2.098727338	0.448862589	intronic	RP1	.		1
3M	rs567080482	13	94423151		T	C	421	0.004751	4	1.409440819	0.301549206	4.67399944	2.95E-06	15.49995607	3.316208371	intergenic	GPC6;DCT	dist=15132;dist=13660		1
3M	rs546286713	13	90758825		G	A	421	0.004751	4	1.389978902	0.297519732	4.671888118	2.98E-06	15.70278409	3.361121605	intergenic	LINC01049;LINC00410	dist=223484;dist=132129		1
3M	rs142311947	5	177957468	0.768029988	G	A	421	0.007126	8	1.813233127	0.388171459	4.671217019	2.99E-06	12.03390127	2.576181159	ncRNA_intronic	LOC100128340	.		1
3M	rs185155853	15	40951902	0.723249972	C	T	421	0.004751	5	1.370089738	0.293393972	4.669795111	3.02E-06	15.91646574	3.408386314	intergenic	DLL4;CHAC1	dist=12829;dist=1569		1
3M	rs550763536	2	7312216		T	G	421	0.005938	5	1.504113282	0.322217093	4.66801208	3.04E-06	14.48716465	3.103497679	intergenic	OC101929452;LOC10050627	dist=234336;dist=109045		1
3M	rs555040883	22	40235472		G	A	421	0.004751	4	1.412000774	0.302532759	4.667265721	3.05E-06	15.42730692	3.305427169	intronic	TNRC6B	.		1
3M	rs2375219	8	54785735		C	T	421	0.393112	330	10.58980707	2.26895928	4.667253025	3.05E-06	2.057001668	0.440730695	intronic	RP1	.		1
3M	rs140062526	13	58711899		G	A	421	0.005938	5	1.544633202	0.331067832	4.665609433	3.08E-06	14.09260875	3.020529034	intergenic	LINC00374;DIAPH3	dist=478782;dist=953688		1
3M	rs572961122	13	107360637	0.793820024	C	T	421	0.005938	6	1.632302386	0.349861236	4.665570858	3.08E-06	13.33548956	2.858276075	intronic	FAM155A	.		1
3M	rs544042801	11	68692775		G	A	421	0.004751	4	1.370120555	0.293669353	4.665521075	3.08E-06	15.88698661	3.405190192	intergenic	GAL;TESMIN	dist=1600;dist=14665		1
3M	rs142993106	4	90036221		G	A	421	0.017815	15	2.691736025	0.577013528	4.664944401	3.09E-06	8.084636111	1.733061622	intergenic	MMRN1;CCSER1	dist=81611;dist=91173		1
3M	rs569916471	14	75424639	0.771009982	G	A	421	0.004751	5	1.390580812	0.298176949	4.663609368	3.11E-06	15.64040878	3.353713303	intergenic	LINC01220;JDP2	dist=128231;dist=3085		1
3M	rs7104959	11	129976231		C	T	421	0.003563	3	1.212990302	0.260168118	4.66233262	3.13E-06	17.92046105	3.843668505	intronic	PRDM10	.		1
3M	rs559228693	20	15982684	0.798879981	G	A	421	0.005938	5	1.512274875	0.324468321	4.660778189	3.15E-06	14.36435511	3.081964969	ncRNA_intronic	LOC613266	.		1
3M	rs187384541	8	1684075	0.760779977	A	G	421	0.035629	26	3.153891836	0.676696813	4.660716257	3.15E-06	6.887451174	1.477766677	intronic	DLGAP2	.		1
3M	rs141127122	22	40208435		G	A	421	0.004751	4	1.411818313	0.302953602	4.660179993	3.16E-06	15.38248751	3.30083549	intronic	TNRC6B	.		1
3M	rs193093906	10	125016920		G	A	421	0.009501	9	2.062968829	0.442790343	4.659019469	3.18E-06	10.52195366	2.25840517	intronic	CTBP2	.		1
3M	rs77871739	9	135660463		G	A	421	0.004751	4	1.439297375	0.309017583	4.657655271	3.20E-06	15.07246035	3.236061811	intergenic	GLT6D1;LCN9	dist=20923;dist=2859		1
3M	rs117816016	8	102739034		C	T	421	0.003563	3	1.090578449	0.234213862	4.656336053	3.22E-06	19.88070227	4.269602176	intergenic	LOC101927245;GASAL1	dist=52311;dist=67788		1
3M	rs775626702	5	163203061		A	C	421	0.003563	3	1.143758046	0.245686137	4.655362562	3.23E-06	18.94841366	4.070233717	intergenic	GABRG2;CCNG1	dist=1047522;dist=234510		1
3M	rs146048121	6	141877818		G	A	421	0.002375	3	1.082392071	0.232511623	4.655217038	3.24E-06	20.02143793	4.300860255	intergenic	MIR4465;NMBR	dist=1193935;dist=196666		1
3M	rs139493286	18	31236056		G	A	421	0.003563	3	1.241116041	0.266612329	4.655133716	3.24E-06	17.46030927	3.750764282	intergenic	DSC1;DSG1	dist=73200;dist=82104		1
3M	rs146442492	15	58689916	0.740549982	C	T	421	0.027316	24	3.06908876	0.65936392	4.654620409	3.25E-06	7.059258577	1.516613162	intronic	ADAM10	.		1
3M	rs181259864	7	97859511		C	A	421	0.003563	3	1.263697226	0.271559968	4.653473908	3.26E-06	17.13608209	3.682427888	ncRNA_intronic	CZ1P-ASNS	.		1
3M	rs150946694	22	46457283		T	C	421	0.004751	4	1.413199859	0.303835874	4.651194872	3.30E-06	15.30824787	3.291250592	intronic	CELSR1	.		1
3M	rs181933850	5	92169830		A	G	421	0.008314	7	1.957149723	0.420974257	4.64909597	3.33E-06	11.04365858	2.375442162	intergenic	ARRDC3-AS1;NR2F1-AS1	dist=749115;dist=1279415		1
3M	rs113006316	2	74575233	0.706809998	A	G	421	0.002375	3	0.943984037	0.203082246	4.648284397	3.35E-06	22.88867923	4.924113346	intronic	M1AP	.		1
3M	rs191006910	5	154836449		G	A	421	0.003563	3	1.226165352	0.263817655	4.647775955	3.36E-06	17.61738031	3.790496891	intronic	FAXDC2	.		1
3M	rs176786	14	45813767		T	C	421	0.32304	268	9.449422953	2.033680348	4.64646421	3.38E-06	2.284756409	0.49171936	intergenic	LINC02303;LINC00871	dist=98165;dist=250392		1
3M	rs187047882	3	164567833		G	A	421	0.003563	3	1.161853087	0.250171732	4.644222095	3.41E-06	18.56413613	3.997254169	intergenic	MIR1263;LINC01324	dist=396277;dist=146262		1
3M	rs73057656	3	33899079	0.799390018	A	G	421	0.036817	33	3.638911487	0.783762258	4.642876649	3.44E-06	5.923832897	1.27589711	intergenic	PDCD6IP;LOC101928135	dist=29372;dist=976718		1
3M	rs112557251	2	187510673	0.726930022	T	C	421	0.002375	3	1.043551353	0.224794821	4.642239298	3.45E-06	20.65100643	4.448501059	intronic	TFPI	.		1
3M	rs190190051	5	92179668		G	A	421	0.008314	7	1.947160785	0.419607459	4.640434158	3.48E-06	11.05898873	2.383179753	intergenic	ARRDC3-AS1;NR2F1-AS1	dist=758953;dist=1269577		1

3M	rs180989936	1	193075048		A	G	421	0.004751	4	1.253423246	0.270112952	4.640367065	3.48E-06	17.17935787	3.702154942	intronic	RO60	.		1
3M	rs188415494	8	25755782	0.735019982	C	T	421	0.003563	3	1.162251387	0.250533229	4.639110717	3.50E-06	18.51694779	3.991486498	intergenic	CDCA2;EBF2	dist=247865;dist=85943		1
3M	rs1686289	14	45791779		G	A	421	0.678147	277	-9.537609426	2.056514006	-4.637755637	3.52E-06	-2.255153927	0.486259757	intergenic	LINC02303;LINC00871	dist=76177;dist=272380		1
3M	rs76904423	12	100794966		G	A	421	0.010689	10	2.190673587	0.472659243	4.634784191	3.57E-06	9.805762313	2.115689082	UTR5	ANO4	NM_001286615:c.-1068200>0;NM_178826:c.-1068200>0		1
3M	rs183466664	12	26668754		A	G	421	0.004751	4	1.341044738	0.289344304	4.634771518	3.57E-06	16.01818822	3.456090156	intronic	ITPR2	.		1
3M	rs999769259	4	61646247	0.702899992	G	A	421	0.003563	3	0.969956079	0.209381164	4.632489677	3.61E-06	22.12467252	4.775978806	intronic	ADGRL3	.		1
3M	rs553840536	16	25686574		A	G	421	0.003563	3	1.093708141	0.236100775	4.632378455	3.61E-06	19.62034417	4.23547954	intergenic	ZKSCAN2;HS3ST4	dist=428729;dist=5385		1
3M	rs183962155	4	21258020		A	C	421	0.005938	6	1.557852883	0.336419694	4.630682783	3.64E-06	13.76460079	2.972477589	intronic	KCNIP4	.		1
3M	rs138480898	1	184986525		C	T	421	0.003563	3	1.261421145	0.272500077	4.629067106	3.67E-06	16.9873974	3.669723728	intergenic	NIBAN1;LINC01633	dist=12017;dist=15002		1
3M	rs185158855	2	222785307	0.73951	C	A	421	0.004751	4	1.284200145	0.277572279	4.626543222	3.72E-06	16.66788644	3.602665239	intergenic	MOGAT1;ACSL3	dist=75377;dist=75728		1
3M	rs180926150	1	102760770	0.792620003	C	T	421	0.002375	3	1.088498575	0.235408416	4.62387281	3.77E-06	19.64191801	4.247936485	intergenic	OLFM3;COL11A1	dist=763536;dist=115697		1
3M	rs146728064	17	19362127		G	A	421	0.007126	6	1.69020067	0.365674681	4.622143005	3.80E-06	12.64004111	2.734671147	intronic	B9D1	.		1
3M	rs111900874	7	89887355		G	A	421	0.014252	12	2.536039096	0.548671755	4.622142612	3.80E-06	8.424240128	1.822583342	ncRNA_intronic	STEAP2-AS1	.		1
3M	rs574076561	7	49505151		A	G	421	0.003563	3	1.086373837	0.235169216	4.619541003	3.85E-06	19.64347663	4.25225723	intergenic	CDC14C;VWC2	dist=577697;dist=268487		1
3M	rs76554191	2	95301880		G	A	421	0.04038	34	3.727552	0.807156314	4.618129021	3.87E-06	5.721480386	1.238917397	intronic	KCNIP3	.		1
3M	rs176783	14	45811710		A	G	421	0.321853	267	9.35224324	2.025260115	4.617798558	3.88E-06	2.280101466	0.493763736	intergenic	LINC02303;LINC00871	dist=96108;dist=252449		1
3M	rs140352232	2	107421656	0.797200024	G	A	421	0.002375	3	1.01264036	0.219291932	4.617772993	3.88E-06	21.05765113	4.560131294	intergenic	MIR548AU;LINC01886	dist=72131;dist=107763		1
3M	rs139062456	8	13394482		C	T	421	0.017815	15	2.721124769	0.589318868	4.617406495	3.89E-06	7.835158086	1.696874229	intronic	DLC1	.		1
3M	rs558614420	15	41518672	0.745689988	C	T	421	0.005938	6	1.524477016	0.330280733	4.615700711	3.92E-06	13.97508315	3.02772732	intronic	RPAP1	.		1
3M	rs12266995	10	24563854		T	C	421	0.030879	26	3.568935699	0.773323681	4.615060663	3.93E-06	5.967825345	1.293119589	intergenic	KIAA1217;ARHGAP21	dist=16006;dist=19760		1
3M	rs192750513	7	97948518		A	G	421	0.003563	3	1.238714279	0.26844613	4.614386809	3.94E-06	17.18924692	3.725142178	ncRNA_intronic	CZ1P-ASNS	.		1
3M	rs56302696	12	47899047	0.778989971	G	A	421	0.002375	3	1.068148373	0.231704225	4.60996503	4.03E-06	19.89590409	4.315847075	intronic	VDR	.		1
3M	rs151015676	5	177963936	0.785820007	T	G	421	0.002375	3	1.054373875	0.228793957	4.608399149	4.06E-06	20.14213671	4.370744819	intergenic	LOC100128340;PROP1	dist=4136;dist=28299		1
3M	rs12315614	12	64527177		C	A	421	0.07601	64	5.614667617	1.219220913	4.605127386	4.12E-06	3.777106623	0.820195905	intergenic	TBK1;RASSF3	dist=25064;dist=83318		1
3M	rs72837643	5	170761951	0.782980025	T	C	421	0.003563	4	1.251913633	0.271928683	4.60383075	4.15E-06	16.93028738	3.677434793	intergenic	KCNIP1;GABRP	dist=25319;dist=21768		1
3M	rs428110	14	45825457		A	C	421	0.317102	265	9.275749143	2.014875505	4.603633881	4.15E-06	2.284822992	0.49630858	intergenic	LINC02303;LINC00871	dist=109855;dist=238702		1
3M	rs111391231	7	89867731		T	C	421	0.014252	12	2.546596314	0.553340001	4.602227036	4.18E-06	8.317177547	1.807207138	intergenic	ZNF804B;STEAP2-AS1	dist=529203;dist=14622		1
3M	rs180765647	13	113724338		G	T	421	0.002375	3	1.05190176	0.228624832	4.600995217	4.20E-06	20.12465213	4.373978059	intronic	GRK1	.		1
3M	rs568658857	12	49460215		G	A	421	0.005938	5	1.486911933	0.32318174	4.600853791	4.21E-06	14.23611926	3.094234224	intronic	SPATS2	.		1
3M	rs182531466	5	92234256		C	A	421	0.007126	6	1.668434681	0.362732486	4.599628498	4.23E-06	12.68049781	2.756852606	intergenic	ARRDC3-AS1;NR2F1-AS1	dist=813541;dist=1214989		1
3M	rs181812512	11	66898258		C	T	421	0.003563	3	1.08407707	0.235726848	4.598869755	4.25E-06	19.50931683	4.242198164	intronic	PC	.		1
3M	rs556680896	13	100950161		C	T	421	0.003563	3	1.24171677	0.270060937	4.597913279	4.27E-06	17.0254659	3.702867989	ncRNA_intronic	NALCN-AS1	.		1
3M	rs142928734	13	100948828		G	A	421	0.003563	3	1.242180957	0.270237412	4.59662838	4.29E-06	17.00959296	3.700449886	ncRNA_intronic	NALCN-AS1	.		1
3M	rs143287889	4	35570658	0.79956001	C	T	421	0.002375	3	1.058108262	0.230199399	4.596485777	4.30E-06	19.96741	4.34406	intergenic	LINC02484;ARAP2	dist=1300911;dist=495346		1
3M	rs543844012	9	30107025		C	T	421	0.003563	3	1.164274175	0.253510467	4.592607905	4.38E-06	18.11604845	3.944610301	intergenic	LINGO2;LINC01242	dist=893424;dist=281910		1
3M	rs566018180	10	84995296	0.773450017	C	T	421	0.003563	3	1.136146605	0.247392568	4.592484792	4.38E-06	18.56355199	4.04215862	intergenic	CCSER2;LINC01519	dist=476775;dist=198125		1
3M	rs147171192	4	88214436		A	G	421	0.007126	5	1.584612591	0.345114993	4.591549547	4.40E-06	13.30440472	2.89758492	intronic	ABCG2	.		1
3M	rs2876414	20	15833059		G	T	421	0.022565	19	3.090964158	0.673260548	4.591037107	4.41E-06	6.819109065	1.485309072	intronic	MACROD2	.		1
3M	rs137873790	5	97751337		A	G	421	0.013064	11	2.280553097	0.497276469	4.586086892	4.52E-06	9.222408811	2.010953789	intergenic	LINC01340;LINC02234	dist=80286;dist=89421		1
3M	rs752259256	10	103165562	0.747950017	T	C	421	0.003563	3	1.029573618	0.224511445	4.58584023	4.52E-06	20.42586391	4.454115907	intronic	NT5C2	.		1
3M	rs185771987	5	73989664	0.738900006	T	C	421	0.003563	3	1.153019969	0.251461612	4.585272329	4.53E-06	18.23448239	3.976750144	intergenic	ARHGEF28;LINC01335	dist=47671;dist=316746		1
3M	rs557092705	20	35601989		C	T	421	0.003563	3	1.079320791	0.235404492	4.584962596	4.54E-06	19.47695457	4.248007298	ncRNA_exonic	FER1L4	.		1
3M	rs140642138	15	41832967		G	A	421	0.005938	6	1.547446255	0.337525343	4.584681679	4.55E-06	13.58322205	2.962740492	intronic	JMJD7;JMJD7-PLA2G4B	.		1
3M	rs139877408	2	128848699	0.79351002	A	G	421	0.002375	3	1.034497038	0.225706887	4.583364964	4.58E-06	20.30671294	4.430524974	intergenic	HS6ST1;LOC101927881	dist=529831;dist=15901		1
3M	rs2327968	20	15832846		C	T	421	0.024941	21	3.410130247	0.744098505	4.582901624	4.59E-06	6.158998564	1.343908089	intronic	MACROD2	.		1
3M	rs191986449	5	25745578		C	T	421	0.004751	3	1.135135845	0.247721071	4.582314459	4.60E-06	18.49787925	4.036798308	intergenic	LINC02211;CDH9	dist=443298;dist=1135019		1
3M	rs11690187	2	67338777		A	C	421	0.005938	5	1.539986371	0.336119518	4.581663041	4.61E-06	13.63105325	2.975132201	intergenic	LINC01828;ETAA1	dist=49533;dist=58556		1
3M	rs118040657	10	3430654	0.777450025	C	T	421	0.008314	8	1.732520372	0.378165204	4.581384943	4.62E-06	12.11477125	2.644346939	ncRNA_intronic	LOC105376360	.		1
3M	rs138109686	15	41759244		A	G	421	0.005938	6	1.543778108	0.336970687	4.581342434	4.62E-06	13.59567051	2.96761718	intronic	MGA	.		1
3M	rs563167766	1	102400809		G	A	421	0.002375	3	1.07877255	0.235715413	1.07877255	4.73E-06	19.41573898	4.242403955	intergenic	OLFM3;COL11A1	dist=403575;dist=475658		1
3M	rs545550279	8	114540200		G	T	421	0.003563	3	1.091458604	0.238542228	4.57553622	4.75E-06	19.18124208	4.192129875	intergenic	CSMD3;TRPS1	dist=1103261;dist=868295		1
3M	rs1498183	8	54804345		C	T	421	0.394299	332	10.33728121	2.259624491	4.574778355	4.77E-06	2.024574602	0.442551408	intronic	RP1	.		1
3M	rs375790303	1	184561348		G	A	421	0.008314	7	1.831959409	0.400449797	4.574754241	4.77E-06	11.42403934	2.497191924	intronic	C1orf21	.		1
3M	rs1396896	8	54782750		A	G	421	0.397862	334	10.46979793	2.288829589	4.574302074	4.78E-06	1.998533266	0.436904523	intronic	RP1	.		1
3M	rs7843693	8	54779552		G	A	421	0.397862	334	10.46760966	2.288570748	4.573863258	4.79E-06	1.998567561	0.436953938	intronic	RP1	.		1
3M	rs1391462	8	54787221		C	A	421	0.397862	334	10.46683006	2.288537601	4.573588854	4.79E-06	1.998476605	0.436960267	intronic	RP1	.		1
3M	rs150539922	21	41856807		T	C	421	0.003563	3	1.254834519	0.274365448	4.57358799	4.79E-06	16.66969372	3.64477381	intronic	PRDM15	.		1
3M	rs145896760	15	41827024		G	A	421	0.005938	6	1.564221999	0.342059488	4.572953105	4.81E-06	13.36888249	2.923468092	UTR3	MAPKBP1	4:c.*15880>0;NM_001128608:c.*15880>0;NM_001265611:c.*15880>0		1
3M	rs181415102	4	111768110	0.716539979	T	C	421	0.003563	4	1.144945045	0.250383121	4.572772469	4.81E-06	18.26310193	3.993879436	intergenic	MIR297;FAM241A	dist=907463;dist=377344		1
3M	rs6080	15	58545734	0.79065001	C	A	421	0.043943	36	3.688174426	0.80689065	4.570847892	4.86E-06	5.664767454	1.239325304	intronic	LIPC	.		1
3M	rs138249376	10	61756071		T	G	421	0.004751	4	1.271516185	0.278189815	4.57067843	4.86E-06	16.43007109	3.594667912	intronic	CABCOCO1	.		1
3M	rs529345909	11	67343381		A	G	421	0.003563	3	1.116845445	0.244375672	4.570198967	4.87E-06	18.70153001	4.092060355	ncRNA_intronic	LOC100130987	.		1
3M	rs191930622	12	47890872	0.790120006	G	A	421	0.002375	3	1.061255347	0.232228501	4.569875551	4.88E-06	19.67835791	4.306103677	intronic	VDR	.		1
3M	rs145116559	4	111756440	0.70095998	T	C	421	0.003563	4	1.144113716	0.250422362	4.568736218	4.91E-06	18.24412236	3.993253602	intergenic	MIR297;FAM241A	dist=895793;dist=389014		1
3M	rs12678939	8	54792461		A	G	421	0.394299	331	10.32905196	2.261417548	4.567512076	4.94E-06	2.019756183	0.442200513	intronic	RP1	.		1
3M	rs73586304	6	142518288		C	T	421	0.003563	3	1.14329832	0.250357885	4.566655922	4.96E-06	18.24051164	3.99428202	intergenic	ADGRG6;LOC153910	dist=72022;dist=8167		1
3M	rs113767990	14	81251219		G	A	421	0.004751	4	1.429622227	0.313123218	4.565685793	4.98E-06	14.58111547	3.193630952	intergenic	LOC101928504;STON2	dist=27839;dist=9431		1
																		Subtotals:	42	371
																		Total:		413

Supplemental Table S3. GWAS Results Risk Loci Indianapolis-1 Discovery Cohort
Merged quantitative traits

Notes
Top SNPs identified with the 12M and 3M QTs are merged, then clustered into risk loci by sorting by chromosomal position, then by chromosome
For risk loci clustering multiple SNPs, the Gene.refGene column entries are boxed
For risk loci clustering multiple SNPs, some of which are of genome-wide significance, the Gene.refGene column entries are bold boxed, the Score.pval column entries are bold boxed, and the bold boxes are shaded in gray
Risk loci clustering SNPs from both the 12 and 3 month QTs are bold boxed across the row and highlighted in blue
Standard Headers
rsID: reference SNP cluster ID, chr_38: chromosome number; pos_38, position of SNP on GRCh38 reference panel; Imputation_Rsq; estimate of the squared correlation between imputed and true genotypes; REF and ALT, reference allele and alternate allele; n.obs: number of observations;
caf: coding allele frequency; MAC: minor allele count; Score: p-values from Score test; Score.SE: standard error of the score statistic; Score.Stat: computed score statistic based on the derivative of the log-likelihood function; Score.pval: probability value associated with the Score.Stat;
EST: estimated effect size; EST.SE: standard error of the effect size; Func.refGene: SNP location with respect to nearest gene; Gene.refGene: closet gene(s) upstream and downstream; GeneDetail.refGene: distance of SNP to nearest gene(s)
Added Headers, Abbreviations and Acronyms
QT: quantitative trait; 3M: 3 month; 12M: 12 month
Count Risk Loci
columns adding up all risk loci broken down by p-value and QT, totals at bottom; 12+3 rep: risk loci clustering both 12M+3M QT SNPs that are duplicates; 12+3 diff: risk loci clustering both 12M+3M QT SNPs for which the 2 QTs identify different SNPs

																		Count Risk Loci					
QT	rsid	chr	pos_38	REF	ALT	n.obs	caf	MAC	Score	Score.SE	Score.Stat	Score.pval	Est	Est.SE	Func.refGene	Gene.refGene	GeneDetail.refGene	5E-08 12M	5E-08 3M	5E-06 12M	5E-06 3M	12+3 rep	12+3 diff
12M	rs115658028	1	7627617	G	A	439	0.00683371	7	2.313647214	0.504805248	4.583247148	4.58E-06	9.07923831	1.980961972	intronic	CAMTA1	.			1			
3M	rs115348382	1	9595845	G	A	421	0.00356295	3	1.230366386	0.259928152	4.733486451	2.21E-06	18.21074945	3.847216981	intronic	TMEM201	.				1		
3M	rs186532456	1	18621834	C	T	421	0.00356295	3	1.189953152	0.242873045	4.899486277	9.61E-07	20.17303433	4.117377453	intergenic	KLHDC7A;PAX7	dist=135848;dist=9012				1		
3M	rs562032622	1	18632839	A	C	421	0.00356295	3	1.126818759	0.231494862	4.867575676	1.13E-06	21.0267115	4.319750303	intronic	PAX7	.				1		
3M	rs2365739	1	62018790	G	A	421	0.02137767	18	3.317349453	0.694985007	4.773267649	1.81E-06	6.868159166	1.43887996	intronic	PATJ	.				1		
3M	rs149493615	1	79414404	G	A	421	0.00831354	8	1.98260972	0.410759865	4.826688019	1.39E-06	11.750632	2.434512435	intergenic	ADGRL4;LINC01781	dist=407674;dist=1121351				1		
3M	rs143811231	1	79502446	T	C	421	0.00831354	8	1.975347907	0.413110429	4.78164619	1.74E-06	11.57474094	2.420660266	intergenic	ADGRL4;LINC01781	dist=495716;dist=1033309						
3M	rs34270375	1	88905019	G	A	421	0.02612827	21	3.119890277	0.63606437	4.904991419	9.34E-07	7.711470175	1.572167924	intergenic	GTF2B;KYAT3	dist=13452;dist=30754				1		
3M	rs187518659	1	98990189	T	G	421	0.00950119	8	1.921562765	0.405573328	4.737892339	2.16E-06	11.68196232	2.465645372	intronic	PLPPR5	.				1		
3M	rs140420703	1	102337928	T	G	421	0.00475059	5	1.375529427	0.288838786	1.375529427	1.91E-06	16.48765399	3.462138915	intergenic	OLFM3;COL11A1	dist=340694;dist=538539		1				
3M	rs563167766	1	102400809	G	A	421	0.0023753	3	1.07877255	0.235715413	1.07877255	4.73E-06	19.41573898	4.242403955	intergenic	OLFM3;COL11A1	dist=403575;dist=475658						
3M	rs77180278	1	102496326	T	C	421	0.02850356	24	3.885721783	0.727780259	5.339141503	9.34E-08	7.336199959	1.374041118	intergenic	OLFM3;COL11A1	dist=499092;dist=380141						
3M	rs112351653	1	102754804	T	C	421	0.02850356	24	4.178844629	0.750261996	5.569847131	2.55E-08	7.423869471	1.332867724	intergenic	OLFM3;COL11A1	dist=757570;dist=121663						
3M	rs180926150	1	102760770	C	T	421	0.0023753	3	1.088498575	0.235408416	4.62387281	3.77E-06	19.64191801	4.247936485	intergenic	OLFM3;COL11A1	dist=763536;dist=115697						
3M	rs114413507	1	102953612	T	C	421	0.02850356	24	4.177369718	0.750215949	5.568223023	2.57E-08	7.42216029	1.332949535	intronic	COL11A1	.						
3M	rs116672066	1	103007360	G	A	421	0.02731591	23	4.276024986	0.728927557	5.866186486	4.46E-09	8.047694764	1.37187844	intronic	COL11A1	.						
3M	rs111928960	1	103168079	G	A	421	0.02850356	22	4.045287237	0.68574164	5.899141891	3.65E-09	8.602572083	1.45827516	intergenic	COL11A1;LOC101928436	dist=59557;dist=325967						
3M	rs113221952	1	103288418	A	G	421	0.02137767	19	3.040841012	0.619495467	4.908576695	9.17E-07	7.923507043	1.614216816	intergenic	COL11A1;LOC101928436	dist=179896;dist=205628						
3M	rs1856085	1	103571923	G	A	421	0.0023753	3	1.181208876	0.234330387	5.040784048	4.64E-07	21.51143997	4.267478979	intronic	AMY2B	.				1		
3M	rs143597860	1	103614521	A	G	421	0.0023753	3	1.179951068	0.233861351	5.045515482	4.52E-07	21.57481541	4.276037896	intergenic	AMY2B;AMY2A	dist=34987;dist=2811						
3M	rs144541665	1	103768107	G	A	421	0.0023753	3	1.181121027	0.232374488	5.082834329	3.72E-07	21.87346108	4.303398392	intergenic	AMY1C;LOC100129138	dist=9415;dist=304916						
3M	rs76098744	1	111806750	C	T	421	0.01662708	14	2.811615503	0.579024995	4.855775707	1.20E-06	8.386124522	1.727041163	intronic	KCND3	.				1		
3M	rs74683551	1	111811796	G	A	421	0.01662708	14	2.810351968	0.579019309	4.853641189	1.21E-06	8.38252043	1.727058121	intronic	KCND3	.						
12M	rs188069356	1	118327095	A	G	439	0.01025057	9	2.965705392	0.643538514	4.60843497	4.06E-06	7.16108651	1.553908552	intergenic	SPAG17;TBX15	dist=141867;dist=555952			1			
12M	rs181375873	1	118341865	G	T	439	0.01025057	9	2.965562087	0.643467132	4.608723494	4.05E-06	7.162329305	1.554080932	intergenic	SPAG17;TBX15	dist=156637;dist=541182						
12M	rs146600651	1	152295232	C	T	439	0.01480638	14	3.66583614	0.743346782	4.931528902	8.16E-07	6.634223785	1.345267141	intergenic	HRNR;FLG	dist=71039;dist=6933			1			
3M	rs76617932	1	180961288	T	C	421	0.01187648	9	2.08220138	0.444364012	4.685801107	2.79E-06	10.54496084	2.250407262	intergenic	KIAA1614-AS1;STX6	dist=6401;dist=11426				1		
3M	rs183180157	1	181277985	A	C	421	0.00950119	7	1.744207622	0.371856251	4.690542692	2.72E-06	12.61385999	2.689211211	intergenic	LINC01699;CACNA1E	dist=39381;dist=205532						
3M	rs375790303	1	184561348	G	A	421	0.00831354	7	1.831959409	0.400449797	4.574754241	4.77E-06	11.42403934	2.497191924	intronic	C1orf21	.				1		
3M	rs138480898	1	184986525	C	T	421	0.00356295	3	1.261421145	0.272500077	4.629067106	3.67E-06	16.9873974	3.669723728	intergenic	NIBAN1;LINC01633	dist=12017;dist=15002				1		
3M	rs145766563	1	185160370	G	A	421	0.00356295	4	1.34508864	0.282384562	4.76332215	1.90E-06	16.86821019	3.541270076	intronic	SWT1	.				1		
3M	rs147032554	1	186179732	T	G	421	0.00356295	3	1.360299196	0.273670575	4.970571635	6.68E-07	18.16260897	3.654028209	intronic	HMCN1	.				1		
3M	rs180989936	1	193075048	A	G	421	0.00475059	4	1.253423246	0.270112952	4.640367065	3.48E-06	17.17935787	3.702154942	intronic	RO60	.				1		
3M	rs10494861	1	205362746	G	A	421	0.00356295	3	1.156337638	0.245524493	4.709663065	2.48E-06	19.18204982	4.072913404	intergenic	KLHDC8A;LEMD1-AS1	dist=5656;dist=10506				1		
12M	rs185899532	1	227321571	T	C	439	0.00569476	4	1.908112393	0.410717289	4.645804902	3.39E-06	11.31144228	2.434764807	intergenic	CDC42BPA;ZNF678	dist=3079;dist=241985			1			
3M	rs2274996	1	229668791	C	T	421	0.0415677	35	4.420411647	0.88962935	4.968823976	6.74E-07	5.585274332	1.124063633	intergenic	URB2;LINC01682	dist=8591;dist=206759				1		
3M	rs2274997	1	229668899	A	G	421	0.0415677	35	4.42362483	0.889790308	4.971536315	6.64E-07	5.587312281	1.123860297	intergenic	URB2;LINC01682	dist=8699;dist=206651						
3M	rs2891865	1	229670621	A	G	421	0.0415677	35	4.419830983	0.88984663	4.966958165	6.80E-07	5.581813763	1.123789164	intergenic	URB2;LINC01682	dist=10421;dist=204929						
3M	rs2385790	1	229671745	C	T	421	0.0415677	35	4.418931852	0.88972149	4.966646197	6.81E-07	5.582248215	1.123947226	intergenic	URB2;LINC01682	dist=11545;dist=203805						
3M	rs12024557	1	229676610	A	C	421	0.04275534	35	4.42434228	0.891299524	4.963923079	6.91E-07	5.569309691	1.121957291	intergenic	URB2;LINC01682	dist=16410;dist=198940						
3M	rs4562666	1	229689023	T	C	421	0.04275534	36	4.44283225	0.897203314	4.951867853	7.35E-07	5.519225993	1.114574572	intergenic	URB2;LINC01682	dist=28823;dist=186527						
3M	rs12036586	1	229690631	G	A	421	0.04750594	40	4.551491816	0.933583971	4.875289164	1.09E-06	5.222121756	1.071140927	intergenic	URB2;LINC01682	dist=30431;dist=184919						
3M	rs16850124	1	229695584	T	C	421	0.04394299	37	4.326094288	0.91378287	4.73426941	2.20E-06	5.180956622	1.094351878	intergenic	URB2;LINC01682	dist=35384;dist=179966						
3M	rs12045643	1	229698303	C	T	421	0.04275534	36	4.472324416	0.903269355	4.951263308	7.37E-07	5.481491517	1.107089479	intergenic	URB2;LINC01682	dist=38103;dist=177247						
12M	rs75626507	1	241251961	G	A	439	0.00341686	4	1.774925973	0.38713626	4.584757758	4.55E-06	11.84274951	2.583069844	intronic	RGS7	.			1			
3M	rs559559983	1	246813830	C	A	421	0.00712589	5	1.645339674	0.345154188	4.76697004	1.87E-06	13.81113197	2.897255878	intergenic	LINC01341;AHCTF1	dist=22344;dist=25268				1		
3M	rs550763536	2	7312216	T	G	421	0.00593824	5	1.504113282	0.322217093	4.66801208	3.04E-06	14.48716465	3.103497679	intergenic	LOC101929452;LOC100506274	dist=234336;dist=109045						
3M	rs558553658	2	15542600	C	T	421	0.00475059	3	1.579685756	0.303851567	5.198873167	2.01E-07	17.10991069	3.291080613	intronic	NBAS	.				1		
3M	rs536781978	2	29510815	A	G	421	0.00356295	3	1.282866581	0.270080884	4.749934759	2.03E-06	17.58708236	3.702594509	intronic	ALK	.				1		
3M	rs568321148	2	29647482	T	G	421	0.00356295	3	1.279867135	0.268808164	4.761265858	1.92E-06	17.71250463	3.720125102	intronic	ALK	.						
12M	rs186792608	2	42869559	A	G	439	0.00341686	3	2.044330208	0.380373238	5.374537432	7.68E-08	14.12964133	2.62899673	intergenic	HAAO;LINC01819	dist=76976;dist=158293			1			
3M	rs76777840	2	48085811	G	A	421	0.00593824	5	1.714828291	0.345687108	4.960637102	7.03E-07	14.35007842	2.892789399	intergenic	FBXO11;FOXN2	dist=179313;dist=228488				1		
3M	rs145080832	2	48246004	G	A	421	0.00475059	4	1.617719031	0.30702202	5.269065159	1.37E-07	17.16184772	3.257095367	intergenic	FBXO11;FOXN2	dist=339506;dist=68295						
3M	rs184220112	2	48404604	C	A	421	0.00475059	4	1.587567329	0.310301123	5.116215225	3.12E-07	16.48790432	3.22267606	intergenic	FOXN2;PPP1R21	dist=25309;dist=36162				1		
3M	rs189890455	2	48428258	C	T	421	0.00593824	5	1.690230255	0.334051451	5.059790188	4.20E-07	15.14673914	2.993550834	intergenic	FOXN2;PPP1R21	dist=48963;dist=12508						
3M	rs181193202	2	52300216	T	C	421	0.00356295	3	1.397666205	0.26682604	5.238117712	1.62E-07	19.63120884	3.747760153	ncRNA_intronic	LOC730100	.				1		
3M	rs183378658	2	52336233	C	T	421	0.00356295	3	1.421941101	0.268359175	5.298649105	1.17E-07	19.74461693	3.726349214	ncRNA_intronic	LOC730100	.						
3M	rs190193113	2	52858640	G	A	421	0.00356295	3	1.409901074	0.265819171	5.303985667	1.13E-07	19.95336019	3.761955902	intergenic	MIR4431;ASB3	dist=156025;dist=811339		1				
3M	rs187520610	2	53132903	G	A	421	0.00475059	4	1.843352227	0.313121747	5.887014379	3.93E-09	18.80103964	3.193645952	intergenic	MIR4431;ASB3	dist=430288;dist=537076						
3M	rs149421869	2	53256291	G	T	421	0.00831354	8	1.92083422	0.404523583	4.748386252	2.05E-06	11.73821862	2.472043763	intergenic	MIR4431;ASB3	dist=553676;dist=413688						
3M	rs146479102	2	65598625	G	A	421	0.00593824	5	1.448302352	0.305828916	4.735661917	2.18E-06	15.48467677	3.269801992	intergenic	SPRED2;MIR4778	dist=166026;dist=759622				1		
3M	rs528288879	2	65648796	C	T	421	0.00593824	6	1.697856008	0.338843001	5.010745399	5.42E-07	14.78780847	2.951219288	intergenic	SPRED2;MIR4778	dist=216197;dist=709451						
3M	rs11690187	2	67338777	A	C	421	0.00593824	5	1.539986371	0.336119518	4.581663041	4.61E-06	13.63105325	2.975132201	intergenic	LINC01828;ETAA1	dist=49533;dist=58556				1		

3M	rs151272830	2	67455592	G	T	421	0.00712589	6	1.643645432	0.351116105	4.681202055	2.85E-06	13.33234787	2.848060758	intergenic	ETAA1;LINC01812	dist=43503;dist=340462						
3M	rs186142189	2	67475575	G	A	421	0.00712589	6	1.7290309	0.36845255	4.692682681	2.70E-06	12.73619271	2.714053683	intergenic	ETAA1;LINC01812	dist=63486;dist=320479						
12M	rs191298981	2	67882914	C	T	439	0.00455581	3	1.760281272	0.335644245	5.244485187	1.57E-07	15.62513066	2.979344989	intergenic	LINC01812;C1D	dist=57352;dist=158216	1					
12M	rs113537164	2	68007798	A	C	439	0.00455581	4	2.083286909	0.391161785	5.325895806	1.00E-07	13.61558315	2.556486955	intergenic	LINC01812;C1D	dist=182236;dist=33332						
12M	rs191823270	2	68025878	T	C	439	0.00341686	3	1.79080594	0.35569611	5.034651464	4.79E-07	14.1543619	2.811388633	intergenic	LINC01812;C1D	dist=200316;dist=15252						
12M	rs113154814	2	68062365	T	C	439	0.00569476	5	2.404201814	0.430061809	5.59036344	2.27E-08	12.99897672	2.32524716	intronic	C1D	.						
3M	rs184200893	2	69033781	C	T	421	0.00356295	3	1.121211249	0.237019909	4.730451775	2.24E-06	19.95803558	4.219054866	intronic	ANTXR1	.				1		
3M	rs111927235	2	74256827	A	G	421	0.00475059	4	1.423024126	0.288923909	4.925255689	8.43E-07	17.04689553	3.461118894	intronic	SLC4A5	.				1		
3M	rs111838310	2	74446364	C	A	421	0.00475059	5	1.465896314	0.287002463	5.107608836	3.26E-07	17.79639376	3.48429066	intergenic	RTKN;INO80B-WBP1	dist=4427;dist=8659				1		
3M	rs112983626	2	74470023	G	A	421	0.00475059	4	1.464072657	0.286917293	5.102768952	3.35E-07	17.78480792	3.485324945	intergenic	MOGS;MRPL53	dist=4641;dist=1959				1		
3M	rs113006316	2	74575233	A	G	421	0.0023753	3	0.943984037	0.203082246	4.648284397	3.35E-06	22.88867923	4.924113346	intronic	M1AP	.				1		
3M	rs17746486	2	95056864	C	T	421	0.03206651	29	3.355977065	0.696974974	4.815061071	1.47E-06	6.908513578	1.434771746	intergenic	MAL;MRPS5	dist=2872;dist=28507				1		
3M	rs76554191	2	95301880	G	A	421	0.04038005	34	3.727552	0.807156314	4.618129021	3.87E-06	5.721480386	1.238917397	intronic	KCNIP3	.				1		
3M	rs140352232	2	107421656	G	A	421	0.0023753	3	1.01264036	0.219291932	4.617772993	3.88E-06	21.05765113	4.560131294	intergenic	MIR548AU;LINC01886	dist=72131;dist=107763				1		
12M	rs182996422	2	111163073	G	A	439	0.00455581	4	1.96047942	0.426062862	4.601385365	4.20E-06	10.79978043	2.347071496	intronic	BCL2L11	.			1			
12M	rs146919974	2	114495286	T	C	439	0.00455581	5	1.870582973	0.407116317	4.594713833	4.33E-06	11.28599774	2.456300469	intronic	DPP10	.			1			
3M	rs116189766	2	125636287	T	C	421	0.0023753	3	1.114549479	0.229338184	4.85985133	1.17E-06	21.19076397	4.360372887	intergenic	CNTNAP5;LINC01941	dist=715069;dist=473813				1		
3M	rs139877408	2	128848699	A	G	421	0.0023753	3	1.034497038	0.225706887	4.583364964	4.58E-06	20.30671294	4.430524974	intergenic	HS6ST1;LOC101927881	dist=529831;dist=15901				1		
12M	rs144801217	2	129943819	T	C	439	0.01252847	11	3.020258487	0.622809182	4.849412265	1.24E-06	7.786353193	1.605628222	ncRNA_intronic	LINC01856	.			1			
12M	rs74492945	2	147426871	G	A	439	0.02961276	25	5.284785249	1.136173009	4.651391299	3.30E-06	4.093911105	0.880147647	intergenic	PABPC1P2;ACVR2A	dist=835881;dist=417647			1			
12M	rs75380640	2	147428229	A	G	439	0.02961276	25	5.284373278	1.136163974	4.65106569	3.30E-06	4.093657074	0.880154646	intergenic	PABPC1P2;ACVR2A	dist=837239;dist=416289						
12M	rs74517425	2	147435566	A	C	439	0.02961276	25	5.255817679	1.12800262	4.659402016	3.17E-06	4.130665953	0.886522764	intergenic	PABPC1P2;ACVR2A	dist=844576;dist=408952						
12M	rs75343152	2	147447179	T	C	439	0.02961276	25	5.373706425	1.145505076	4.691124061	2.72E-06	4.095245108	0.872977362	intergenic	PABPC1P2;ACVR2A	dist=856189;dist=397339						
12M	rs76487561	2	147457410	C	T	439	0.0284738	25	5.507313038	1.141042849	4.826561108	1.39E-06	4.229956054	0.876391277	intergenic	PABPC1P2;ACVR2A	dist=866420;dist=387108						
12M	rs10175536	2	147460050	A	G	439	0.02733485	24	5.059239074	1.084265764	4.666050745	3.07E-06	4.30341979	0.92228311	intergenic	PABPC1P2;ACVR2A	dist=869060;dist=384468						
12M	rs75304512	2	147460438	A	G	439	0.02733485	24	5.052915513	1.079656239	4.680115142	2.87E-06	4.334819707	0.926220739	intergenic	PABPC1P2;ACVR2A	dist=869448;dist=384080						
12M	rs74380227	2	147462714	C	A	439	0.02733485	24	5.051193478	1.079667701	4.678470491	2.89E-06	4.333250397	0.926210907	intergenic	PABPC1P2;ACVR2A	dist=871724;dist=381804						
12M	rs10210722	2	147465215	T	C	439	0.02733485	24	5.051665699	1.07945012	4.679850978	2.87E-06	4.335402712	0.9263976	intergenic	PABPC1P2;ACVR2A	dist=874225;dist=379303						
12M	rs1258074269	2	147466224	A	C	439	0.02733485	24	5.051665699	1.07945012	4.679850978	2.87E-06	4.335402712	0.9263976	intergenic	PABPC1P2;ACVR2A	dist=875234;dist=378294						
12M	rs77928469	2	147466550	C	T	439	0.02733485	24	5.051484579	1.079409821	4.679857902	2.87E-06	4.335570987	0.926432186	intergenic	PABPC1P2;ACVR2A	dist=875560;dist=377968						
12M	rs77592434	2	147469077	A	G	439	0.02733485	24	5.051588616	1.07940957	4.679955372	2.87E-06	4.335662295	0.926432401	intergenic	PABPC1P2;ACVR2A	dist=878087;dist=375441						
12M	rs6724770	2	147469854	A	G	439	0.02733485	24	5.05104599	1.078982106	4.68130654	2.85E-06	4.338632229	0.926799429	intergenic	PABPC1P2;ACVR2A	dist=878864;dist=374664						
12M	rs80339745	2	147470110	G	A	439	0.02733485	24	5.050651164	1.078960151	4.681035866	2.85E-06	4.338649649	0.926818288	intergenic	PABPC1P2;ACVR2A	dist=879120;dist=374408						
12M	rs78849093	2	147478023	C	A	439	0.02733485	24	5.057603105	1.078451085	4.689691702	2.74E-06	4.348543728	0.927255778	intergenic	PABPC1P2;ACVR2A	dist=887033;dist=366495						
12M	rs75330406	2	147481113	G	A	439	0.02733485	24	5.0599299	1.078749077	4.690553168	2.72E-06	4.348141072	0.926999635	intergenic	PABPC1P2;ACVR2A	dist=890123;dist=363405						
3M	rs142894171	2	150581757	G	T	421	0.00356295	3	1.287888043	0.266960796	4.82425907	1.41E-06	18.07103941	3.74586836	intergenic	LINC01920;LINC02612	dist=9536;dist=47140				1		
12M	rs138454387	2	163831400	G	A	439	0.00569476	5	2.115842897	0.458436819	4.615342424	3.92E-06	10.0675649	2.181325669	intergenic	FIGN;GRB14	dist=95392;dist=661017			1			
12M	rs146300183	2	164138767	T	C	439	0.00455581	4	1.984193903	0.407444048	4.869856149	1.12E-06	11.95220834	2.454324722	intergenic	FIGN;GRB14	dist=402759;dist=353650						
3M	rs541508507	2	169166755	G	A	421	0.00475059	4	1.442660176	0.305446461	4.723119638	2.32E-06	15.46300334	3.273896179	intronic	LRP2	.				1		
3M	rs142549310	2	169173996	C	T	421	0.00475059	4	1.443948619	0.307707803	4.692596694	2.70E-06	15.25017126	3.249836338	exonic	LRP2	.						
3M	rs556293455	2	176085692	G	A	421	0.00356295	3	1.630922146	0.336702149	4.843812709	1.27E-06	14.38604633	2.969984018	intergenic	EVX2;HOXD13	dist=1730;dist=7029				1		
3M	rs184098071	2	176251692	G	A	421	0.00356295	3	1.695892029	0.34869637	4.863520744	1.15E-06	13.94772404	2.86782452	intergenic	HOXD1;MTX2	dist=60785;dist=17750			1	1		1
12M	rs192500829	2	176385923	A	G	439	0.00455581	3	1.674098367	0.340713541	4.913506992	8.95E-07	14.42122604	2.935016895	intergenic	MTX2;MIR1246	dist=47898;dist=215057						
3M	rs532416695	2	176622062	G	A	421	0.00356295	3	1.400161077	0.265436681	5.274934396	1.33E-07	19.87266561	3.767376828	intergenic	MIR1246;LINC01116	dist=21010;dist=7519				1		
3M	rs112557251	2	187510673	T	C	421	0.0023753	3	1.043551353	0.224794821	4.642239298	3.45E-06	20.65100643	4.448501059	intronic	TFPI	.				1		
12M	rs140561530	2	205755707	G	T	439	0.0022779	3	1.412604553	0.289304563	4.882759325	1.05E-06	16.87757453	3.456564906	intronic	NRP2	.			1			
3M	rs185158855	2	222785307	C	A	421	0.00475059	4	1.284200145	0.277572279	4.626543222	3.72E-06	16.66788644	3.602665239	intergenic	MOGAT1;ACSL3	dist=75377;dist=75728				1		
3M	rs185510569	2	222950143	G	A	421	0.00356295	3	1.507358682	0.277399453	5.433892049	5.51E-08	19.58869056	3.604909775	intergenic	ACSL3;KCNE4	dist=5505;dist=102047				1		
3M	rs147559909	2	236142879	T	C	421	0.00593824	5	1.858263542	0.329805301	5.634425942	1.76E-08	17.08409752	3.032091952	intergenic	AGAP1;GBX2	dist=11086;dist=22356		1				
12M	rs146298016	2	237657156	G	A	439	0.00797267	7	2.369872335	0.517388552	4.580449887	4.64E-06	8.85301746	1.93278339	intronic	LRRFIP1	.			1			
3M	rs181217257	2	239067423	C	T	421	0.00475059	4	1.788144205	0.287937999	6.210170973	5.29E-10	21.56773677	3.472969885	intronic	HDAC4	.		1	1			1
3M	rs188076929	2	239072023	T	C	421	0.00475059	4	1.865485168	0.305579814	6.104739516	1.03E-09	19.97756143	3.272467463	intronic	HDAC4	.						
12M	rs115690445	2	239682619	A	C	439	0.00797267	7	2.51110667	0.515854898	4.867854661	1.13E-06	9.436480453	1.938529621	intergenic	HDAC4-AS1;LOC150935	dist=280255;dist=80241						
3M	rs112475378	3	1584977	T	C	421	0.01662708	14	2.445371748	0.506000821	4.832742644	1.35E-06	9.550859286	1.976281377	intergenic	CNTN6;CNTN4	dist=180760;dist=513826				1		
3M	rs145676540	3	2002951	C	T	421	0.00593824	5	1.68032939	0.346683687	4.846866048	1.25E-06	13.98065798	2.884473769	intergenic	CNTN6;CNTN4	dist=598734;dist=95852						
12M	rs55939894	3	7921601	A	G	439	0.18792711	165	-12.24219411	2.600577928	-4.707489817	2.51E-06	-1.810170642	0.384529911	intergenic	GRM7;LOC101927394	dist=180068;dist=31204			1			
12M	rs11920261	3	7935524	G	T	439	0.1856492	162	-12.09011362	2.598457274	-4.65280447	3.27E-06	-1.790602646	0.384843734	intergenic	GRM7;LOC101927394	dist=193991;dist=17281						
12M	rs2171551	3	7936391	C	A	439	0.1856492	162	-12.08866418	2.598460193	-4.652241434	3.28E-06	-1.790383954	0.384843302	intergenic	GRM7;LOC101927394	dist=194858;dist=16414						
3M	rs146007933	3	28185932	T	C	421	0.02137767	18	3.108745063	0.643323442	4.832320511	1.35E-06	7.511494525	1.554428045	intergenic	LINC01980;CMC1	dist=325607;dist=55687				1		
3M	rs73057656	3	33899079	A	G	421	0.0368171	33	3.638911487	0.783762258	4.642876649	3.44E-06	5.923832897	1.27589711	intergenic	PDCD6IP;LOC101928135	dist=29372;dist=976718				1		
3M	rs73085348	3	42669729	A	G	421	0.01187648	11	2.448275953	0.491389455	4.982353469	6.28E-07	10.13931704	2.035045707	intergenic	ZBTB47;KLHL40	dist=2149;dist=15808				1		
12M	rs2229593	3	42864724	C	T	439	0.02733485	24	4.870496863	1.032388754	4.717696551	2.39E-06	4.569689987	0.968627367	exonic	ACKR2	.			1			
12M	rs139029292	3	44072032	G	A	439	0.00569476	6	2.199467961	0.481405962	4.568842376	4.90E-06	9.490622744	2.077248888	intergenic	ABHD5;MIR138-1	dist=337661;dist=42180			1			
3M	rs142684595	3	55285273	T	C	421	0.00593824	5	1.730096674	0.321166729	5.386911268	7.17E-08	16.77294307	3.113647549	intergenic	LINC02017;WNT5A	dist=96059;dist=180442				1		
12M	rs148953849	3	56866690	G	A	439	0.00455581	4	1.732750622	0.35482831	4.883349426	1.04E-06	13.76256993	2.818264419	intronic	ARHGEF3	.			1			
3M	rs80203220	3	123003484	C	T	421	0.00593824	6	1.662045235	0.351841788	4.723842626	2.31E-06	13.42604201	2.842186558	intronic	SEMA5B	.				1		
12M	rs114280794	3	133123359	G	A	439	0.01594533	14	4.764666379	0.86324065	5.519511134	3.40E-08	6.393942562	1.158425521	intronic	TMEM108	.	1					
3M	rs192443987	3	135813503	G	A	421	0.00475059	4	1.31410558	0.27206124	4.830183004	1.36E-06	17.75402845	3.675643022	intergenic	EPHB1;PPP2R3A	dist=553038;dist=152225				1		
12M	rs148248743	3	136415753	C	T	439	0.0022779	3	1.586456973	0.298755282	5.310222348	1.09E-07	17.7744886	3.347221159	intronic	STAG1	.			1	1	1	
3M	rs148248743	3	136415753	C	T	421	0.0023753	3	1.069833829	0.216429281	4.943110391	7.69E-07	22.83937904	4.620446891	intronic	STAG1	.						
3M	rs576124203	3	142122354	T	G	421	0.00356295	3	1.313230925	0.260150702	5.047962263	4.47E-07	19.4039925	3.843925823	intronic	TFDP2	.				1		
3M	rs545552231	3	142145508	C	T	421	0.00356295	3	1.275833022	0.253432385	5.034214646	4.80E-07	19.8641332	3.945825635	intronic	TFDP2	.						
12M	rs138138661	3	147940027	C	T	439	0.00455581	4	1.755727133	0.380325922	4.616375137	3.91E-06	12.13794502	2.629323801	intergenic	LOC440982;LINC02032	dist=430117;dist=138132			1			
12M	rs148997617	3	148037789	C	T	439	0.00455581	4	1.904562472	0.412852965	4.613173773	3.97E-06	11.17389037	2.422169838	intergenic	LOC440982;LINC02032	dist=527879;dist=40370						
3M	rs193153124	3	148612923	A	G	421	0.00593824	5	1.529063164	0.32624874	4.686801743	2.78E-06	14.36573132	3.065145937	intergenic	LINC02046;AGTR1	dist=212967;dist=84948				1		
3M	rs188720948	3	150352093	T	C	421	0.00356295	3	1.368098189	0.277991774	4.921362129	8.59E-07	17.7032653	3.597228742	intergenic	LINC01214;TSC22D2	dist=28346;dist=56205				1		
3M	rs16823323	3	153939413	G	A	421	0.01662708	14	3.214602568	0.582174532	5.521716236	3.36E-08	9.484640653	1.717697949	intergenic	LINC02006;ARHGEF26-AS	dist=176887;dist=84988		1				
3M	rs139943877	3	155733500	G	A	421	0.00712589	7	1.713092922	0.362264246	4.728849011	2.26E-06	13.0535902	2.760415942	intronic	PLCH1	.				1		
3M	rs187047882	3	164567833	G	A	421	0.00356295	3	1.161853087	0.250171732	4.644222095	3.41E-06	18.56413613	3.997254169	intergenic	MIR1263;LINC01324	dist=396277;dist=146262			1	1		1
12M	rs139635002	3	164614597	T	C	439	0.00797267	8	2.540439901	0.548123379	4.63479574	3.57E-06	8.4557527	1.824406765	intergenic	MIR1263;LINC01324	dist=443041;dist=99498						
3M	rs141169929	3	165090674	A	G	421	0.00475059	5	1.473704308	0.315071386	4.677366376	2.91E-06	14.84541783	3.173883899	intergenic	SI;SLITRK3	dist=12178;dist=96046						
12M	rs754653672	3	165112751	A	G	439	0.00341686	3	1.695149727	0.37024662	4.578434038	4.68E-06	12.36590367	2.700902442	intergenic	SI;SLITRK3	dist=34255;dist=73969						
12M	rs994788245	3	165182662	G	T	439	0.00341686	3	1.457659335	0.317040616	4.597705341	4.27E-06	14.50194425	3.154169999	intergenic	SI;SLITRK3	dist=104166;dist=4058						

3M	rs186649043	3	175214503	C	T	421	0.00356295	3	1.162234876	0.245948741	4.725516671	2.30E-06	19.21342086	4.065887859	intronic	NAALADL2	.				1		
12M	rs189709453	3	177856520	G	A	439	0.00341686	3	1.623833795	0.337340968	4.813627607	1.48E-06	14.26932412	2.964359789	ncRNA_intronic	LINC02015	.			1	1	1	
3M	rs189709453	3	177856520	G	A	421	0.00356295	3	1.314463975	0.246356303	5.335621454	9.52E-08	21.65814876	4.05916142	ncRNA_intronic	LINC02015	.						
12M	rs186767531	3	177921989	T	C	439	0.00341686	3	1.610325603	0.338183106	4.761697361	1.92E-06	14.08023428	2.956977988	intergenic	LINC02015;LINC01014	dist=22765;dist=497212						
3M	rs186767531	3	177921989	T	C	421	0.00356295	3	1.293967205	0.247047316	5.237730262	1.63E-07	21.20132426	4.047807581	intergenic	LINC02015;LINC01014	dist=22765;dist=497212						
12M	rs182868205	3	177994660	C	T	439	0.00341686	3	1.649581368	0.344172256	4.792894657	1.64E-06	13.92586001	2.90552182	intergenic	LINC02015;LINC01014	dist=95436;dist=424541						
3M	rs182868205	3	177994660	C	T	421	0.00356295	3	1.323927501	0.251356167	5.267137522	1.39E-07	20.95487681	3.978418395	intergenic	LINC02015;LINC01014	dist=95436;dist=424541						
12M	rs76356799	3	179875980	G	A	439	0.00455581	4	2.106771109	0.432864845	4.867041374	1.13E-06	11.24378991	2.310189917	intronic	PEX5L	.			1			
12M	rs571346923	3	184247590	G	A	439	0.00341686	3	1.651446737	0.342609142	4.820206279	1.43E-06	14.06911168	2.918777925	intronic	ALG3	.			1			
3M	rs191792521	3	195919734	G	A	421	0.00831354	6	1.587589638	0.337332296	4.706307863	2.52E-06	13.95154842	2.964435992	intergenic	TNK2-AS1;SDHAP1	dist=6470;dist=40187				1		
3M	rs113651406	4	997179	C	T	421	0.00356295	3	1.369997433	0.265704278	5.156098506	2.52E-07	19.40540264	3.763582603	intronic	IDUA	.				1		
3M	rs12502861	4	2424578	T	C	421	0.01068884	9	1.987294963	0.422008794	4.709131639	2.49E-06	11.15884718	2.369618867	intronic	CFAP99	.				1		
3M	rs189765693	4	4323066	T	C	421	0.00475059	4	1.425732565	0.30309955	4.70384257	2.55E-06	15.51913413	3.299246074	intergenic	ZBTB49;NSG1	dist=1283;dist=63466				1		
12M	rs189094663	4	11621826	G	A	439	0.0022779	3	1.615404085	0.319409161	5.057475753	4.25E-07	15.83384692	3.13078059	intergenic	HS3ST1;LINC02360	dist=192932;dist=119125				1		
3M	rs183962155	4	21258020	A	C	421	0.00593824	6	1.557852883	0.336419694	4.630682783	3.64E-06	13.76460079	2.972477589	intronic	KCNIP4	.				1		
3M	rs113751774	4	23427231	C	T	421	0.00712589	7	1.694246649	0.337089114	5.026109061	5.01E-07	14.91032743	2.966574592	intergenic	GBA3;PPARGC1A	dist=607659;dist=364790		1				
3M	rs113063005	4	23507444	T	C	421	0.00593824	6	2.130262689	0.316937024	6.721406875	1.80E-11	21.2073894	3.155200957	intergenic	GBA3;PPARGC1A	dist=687872;dist=284577						
3M	rs145875128	4	32071514	G	A	421	0.00356295	3	1.153690598	0.217089638	5.314351295	1.07E-07	24.47998599	4.606392132	ncRNA_intronic	LINC02506	.	1		1	1		1
12M	rs146847867	4	32390284	G	T	439	0.00455581	4	2.185302341	0.426711618	5.121262811	3.03E-07	12.00169528	2.3435031	intergenic	LINC02353;LOC101928622	dist=37064;dist=1506055						
12M	rs183086778	4	32609199	A	G	439	0.00455581	5	2.346647932	0.430540407	5.45047084	5.02E-08	12.6596035	2.322662367	intergenic	LINC02353;LOC101928622	dist=255979;dist=1287140						
12M	rs144899985	4	32738728	A	G	439	0.00455581	5	2.245036023	0.430094163	5.219870935	1.79E-07	12.13657701	2.32507224	intergenic	LINC02353;LOC101928622	dist=385508;dist=1157611						
12M	rs192772770	4	32808172	T	G	439	0.00569476	5	2.334772732	0.461927473	5.054414098	4.32E-07	10.94200798	2.164842011	intergenic	LINC02353;LOC101928622	dist=454952;dist=1088167						
12M	rs76887296	4	32865383	C	T	439	0.00569476	5	2.374205059	0.477289182	4.974353383	6.55E-07	10.42209538	2.09516586	intergenic	LINC02353;LOC101928622	dist=512163;dist=1030956						
3M	rs143287889	4	35570658	C	T	421	0.0023753	3	1.058108262	0.230199399	4.596485777	4.30E-06	19.96741	4.34406	intergenic	LINC02484;ARAP2	dist=1300911;dist=495346						
3M	rs77141817	4	37052137	T	C	421	0.00356295	3	1.553761456	0.274563203	5.659030198	1.52E-08	20.61102923	3.642148656	intergenic	LINC02616;MIR4801	dist=31431;dist=189773		1				
3M	rs190822761	4	37097734	G	T	421	0.00356295	3	1.550650175	0.274463961	5.649740562	1.61E-08	20.58463536	3.643465595	intergenic	LINC02616;MIR4801	dist=77028;dist=144176						
12M	rs147267707	4	43024541	G	A	439	0.00455581	4	2.094031845	0.428388026	4.888166139	1.02E-06	11.41060403	2.334332284	intronic	GRXCR1	.			1			
12M	rs77618729	4	43048951	T	C	439	0.00455581	4	2.093237083	0.42828012	4.887542018	1.02E-06	11.41202168	2.334920424	intergenic	GRXCR1;LINC02383	dist=18293;dist=408583						
12M	rs556435089	4	43549016	G	A	439	0.00455581	4	2.093380466	0.427400072	4.897941303	9.68E-07	11.45985137	2.339728197	intergenic	LINC02383;LINC02475	dist=56473;dist=467845						
12M	rs532464521	4	43569022	T	C	439	0.00455581	4	2.094609056	0.427328656	4.901634909	9.50E-07	11.47041005	2.340119219	intergenic	LINC02383;LINC02475	dist=76479;dist=447839						
12M	rs191122698	4	60001148	T	C	439	0.00683371	6	2.484118908	0.503688379	4.931856703	8.15E-07	9.791483996	1.98535452	intergenic	LINC02429;MIR548AG1	dist=954189;dist=921471			1	1		1
12M	rs181164671	4	60202387	G	T	439	0.00683371	6	2.365357436	0.473829215	4.992004204	5.98E-07	10.53545041	2.110465052	intergenic	LINC02429;MIR548AG1	dist=1155428;dist=720232						
3M	rs999769259	4	61646247	G	A	421	0.00356295	3	0.969956079	0.209381164	4.632489677	3.61E-06	22.12467252	4.775978806	intronic	ADGRL3	.				1		
12M	rs183601329	4	79385832	C	T	439	0.00341686	3	1.528621191	0.307223916	4.97559308	6.50E-07	16.19533121	3.254954928	intergenic	NAA11;GK2	dist=59771;dist=20529			1			
12M	rs564991659	4	79495431	A	G	439	0.0022779	3	1.478600686	0.29470879	5.017158418	5.24E-07	17.02412208	3.393180096	ncRNA_intronic	LINC00989	.			1			
12M	rs115901263	4	86154667	A	G	439	0.00569476	6	2.238202026	0.474593389	4.716041306	2.40E-06	9.93701433	2.107066856	ncRNA_intronic	MAPK10-AS1	.			1	1		1
3M	rs147630370	4	86529522	T	C	421	0.00475059	4	1.530518883	0.309272178	4.948776482	7.47E-07	16.00136328	3.23339786	intergenic	MAPK10;MIR4452	dist=76327;dist=12960						
3M	rs147171192	4	88214436	A	G	421	0.00712589	5	1.584612591	0.345114993	4.591549547	4.40E-06	13.30440472	2.89758492	intronic	ABCG2	.				1		
3M	rs142993106	4	90036221	G	A	421	0.01781473	15	2.691736025	0.577013528	4.664944401	3.09E-06	8.084636111	1.733061622	intergenic	MMRN1;CCSER1	dist=81611;dist=91173				1		
3M	rs146526206	4	90071867	T	C	421	0.01781473	15	2.815290022	0.58984771	4.772909977	1.82E-06	8.091766558	1.695352855	intergenic	MMRN1;CCSER1	dist=117257;dist=55527						
3M	rs17017794	4	90904734	T	C	421	0.0391924	33	4.452559757	0.873737599	5.095991934	3.47E-07	5.83240545	1.144508375	intronic	CCSER1	.						
12M	rs979665	4	100960199	A	G	439	0.64464692	320	14.57615127	2.841511195	5.129718053	2.90E-07	1.805278143	0.351925413	intergenic	LINC01216;PPP3CA	dist=285086;dist=63219			1			
3M	rs145116559	4	111756440	T	C	421	0.00356295	4	1.144113716	0.250422362	4.568736218	4.91E-06	18.24412236	3.993253602	intergenic	MIR297;FAM241A	dist=895793;dist=389014				1		
3M	rs181415102	4	111768110	T	C	421	0.00356295	4	1.144945045	0.250383121	4.572772469	4.81E-06	18.26310193	3.993879436	intergenic	MIR297;FAM241A	dist=907463;dist=377344						
3M	rs191423619	4	125595472	G	T	421	0.0023753	3	1.225954306	0.23771753	5.157189324	2.51E-07	21.69461097	4.206673365	intergenic	MIR2054;INTU	dist=88165;dist=2037485				1		
3M	rs149298750	4	126237546	A	C	421	0.00356295	3	1.219140188	0.234426329	5.200525875	1.99E-07	22.18405204	4.265732461	intergenic	MIR2054;INTU	dist=730239;dist=1395411						
12M	rs181669665	4	128644045	G	A	439	0.00569476	4	1.881997947	0.380114274	4.951137266	7.38E-07	13.02539159	2.630787815	intergenic	LINC02615;JADE1	dist=124649;dist=165655			1			
12M	rs181132315	4	128726214	C	T	439	0.00341686	3	1.85813691	0.353270418	5.259814619	1.44E-07	14.88891893	2.830692717	intergenic	LINC02615;JADE1	dist=206818;dist=83486						
12M	rs13103178	4	130157785	T	C	439	0.01936219	21	4.095545102	0.855702041	4.786181292	1.70E-06	5.593280207	1.168631079	intergenic	LINC02465;LINC02479	dist=202417;dist=218576			1			
3M	rs112679237	4	138219706	T	C	421	0.01781473	18	3.098512369	0.596371661	5.195606321	2.04E-07	8.712027524	1.676806707	intronic	SLC7A11	.				1		
12M	rs184618624	4	150066449	T	C	439	0.0022779	3	1.489513245	0.308282145	4.831655903	1.35E-06	15.67283732	3.243781766	intergenic	IQCM;DCLK2	dist=250606;dist=11996			1			
12M	rs144967461	4	152847797	T	G	439	0.01822323	16	4.171868899	0.801202658	5.207008309	1.92E-07	6.498990306	1.248123667	intronic	ARFIP1	.			1			
3M	rs531769270	4	153489891	T	C	421	0.00356295	3	1.339792194	0.284142497	4.715212285	2.41E-06	16.59453383	3.519360917	intronic	TMEM131L	.				1		
3M	rs116651654	4	162317591	C	T	421	0.00712589	6	1.661626207	0.326131851	5.094952243	3.49E-07	15.62236937	3.066244515	intergenic	FSTL5;MIR4454	dist=153557;dist=775983			1	1		1
12M	rs114259958	4	162398591	A	G	439	0.00341686	3	1.650939137	0.357816014	4.613933065	3.95E-06	12.89470814	2.794732381	intergenic	FSTL5;MIR4454	dist=234557;dist=694983			1			
3M	rs567982164	5	25631188	G	A	421	0.0023753	3	1.14494858	0.228986639	5.000067194	5.73E-07	21.8356286	4.367067031	intergenic	LINC02211;CDH9	dist=328908;dist=1249409				1		
3M	rs191986449	5	25745578	C	T	421	0.00475059	3	1.135135845	0.247721071	4.582314459	4.60E-06	18.49787925	4.036798308	intergenic	LINC02211;CDH9	dist=443298;dist=1135019						
12M	rs185242027	5	29071862	G	A	439	0.00455581	3	1.595602789	0.344148816	4.636374483	3.55E-06	13.47200475	2.905719716	ncRNA_intronic	LOC101929645	.			1			
12M	rs10038482	5	37593566	C	T	439	0.00455581	4	1.945686385	0.408444718	4.763646826	1.90E-06	11.66289247	2.448311744	intronic	WDR70	.			1			
12M	rs142934021	5	53366842	G	A	439	0.00797267	7	2.548043395	0.487663617	5.225002041	1.74E-07	10.7143569	2.050593821	intergenic	LOC257396;FST	dist=251716;dist=113787			1			
3M	rs185771987	5	73989664	T	C	421	0.00356295	3	1.153019969	0.251461612	4.585272329	4.53E-06	18.23448239	3.976750144	intergenic	ARHGEF28;LINC01335	dist=47671;dist=316746				1		
3M	rs139360368	5	74076284	A	C	421	0.00356295	4	1.280137576	0.264060375	4.847897286	1.25E-06	18.35904869	3.78701272	intergenic	ARHGEF28;LINC01335	dist=134291;dist=230126						
3M	rs181933850	5	92169830	A	G	421	0.00831354	7	1.957149723	0.420974257	4.64909597	3.33E-06	11.04365858	2.375442162	intergenic	ARRDC3-AS1;NR2F1-AS1	dist=749115;dist=1279415				1		
3M	rs190190051	5	92179668	G	A	421	0.00831354	7	1.947160785	0.419607459	4.640434158	3.48E-06	11.05898873	2.383179753	intergenic	ARRDC3-AS1;NR2F1-AS1	dist=758953;dist=1269577						
3M	rs182531466	5	92234256	C	A	421	0.00712589	6	1.668434681	0.362732486	4.599628498	4.23E-06	12.68049781	2.756852606	intergenic	ARRDC3-AS1;NR2F1-AS1	dist=813541;dist=1214989						
3M	rs187236873	5	92234630	G	A	421	0.00712589	6	1.81660653	0.381998785	4.755529603	1.98E-06	12.44906998	2.617809375	intergenic	ARRDC3-AS1;NR2F1-AS1	dist=813915;dist=1214615						
3M	rs183816745	5	92336071	A	G	421	0.00593824	5	1.692149037	0.312309144	5.418186024	6.02E-08	17.34879088	3.201955563	intergenic	ARRDC3-AS1;NR2F1-AS1	dist=915356;dist=1113174						
3M	rs111407636	5	95744325	C	T	421	0.00356295	3	1.331610247	0.26725959	4.98246012	6.28E-07	18.64277396	3.741680518	intronic	RHOBTB3	.				1		
3M	rs111676272	5	95758594	C	A	421	0.00356295	3	1.327188035	0.267072797	4.969386812	6.72E-07	18.6068625	3.744297477	intronic	RHOBTB3	.						
3M	rs75848314	5	95762636	T	C	421	0.00356295	3	1.401525911	0.264060948	5.307584942	1.11E-07	20.0998481	3.787004507	intronic	RHOBTB3	.						
3M	rs111846247	5	95775939	T	C	421	0.00356295	3	1.3787786	0.259425442	5.314739335	1.07E-07	20.48657718	3.854672052	intronic	RHOBTB3	.						
3M	rs137873790	5	97751337	A	G	421	0.01306413	11	2.280553097	0.497276469	4.586086892	4.52E-06	9.222408811	2.010953789	intergenic	LINC01340;LINC02234	dist=80286;dist=89421				1		
12M	rs569429270	5	116060403	C	T	439	0.00341686	3	1.696972577	0.357132375	4.751662676	2.02E-06	13.30504599	2.800082182	downstream	ARL14EPL	dist=914			1			
12M	rs140512918	5	116111989	T	C	439	0.00341686	3	1.688393978	0.363434391	4.645663762	3.39E-06	12.78267517	2.75152827	intronic	COMMD10	.			1			
12M	rs185275344	5	120442008	C	T	439	0.00455581	5	1.963836945	0.429890979	4.56822088	4.92E-06	10.6264637	2.326171168	intergenic	FAM170A;PRR16	dist=806186;dist=22270			1			
3M	rs189912648	5	135429749	C	T	421	0.00356295	4	1.303529678	0.277126356	4.703737665	2.55E-06	16.97325991	3.608462274	intergenic	MACROH2A1;DCANP1	dist=29862;dist=14465				1		
3M	rs191006910	5	154836449	G	A	421	0.00356295	3	1.226165352	0.263817655	4.647775955	3.36E-06	17.61738031	3.790496891	intronic	FAXDC2	.				1		
3M	rs74343174	5	162066176	C	A	421	0.00593824	5	1.498573008	0.318559187	4.704221604	2.55E-06	14.76718237	3.139134082	intergenic	LINC01202;GABRG2	dist=64980;dist=1289				1		
3M	rs775626702	5	163203061	A	C	421	0.00356295	3	1.143758046	0.245686137	4.655362562	3.23E-06	18.94841366	4.070233717	intergenic	GABRG2;CCNG1	dist=1047522;dist=234510						
3M	rs371245624	5	163453950	T	C	421	0.00356295	3	1.17843308	0.245958889	4.791179069	1.66E-06	19.47959308	4.065720108	UTR3	NUDCD2	NM_001329991:c.*170>0;NM_14[illegible]				1		
3M	rs290120	5	163841238	T	G	421	0.00712589	6	1.664921355	0.353031552	4.716069562	2.40E-06	13.3587764	2.832608007	intergenic	MAT2B;LINC02143	dist=321884;dist=607184				1		
3M	rs545428520	5	168394261	T	C	421	0.00356295	4	1.606350972	0.272919814	5.885798274	3.96E-09	21.56603503	3.664079879	intronic	WWC1	.		1				
3M	rs528404963	5	168425020	T	C	421	0.00356295	4	1.58410842	0.273744969	5.786803767	7.17E-09	21.13939767	3.653035168	intronic	WWC1	.						

3M	rs72832764	5	170577669	G	A	421	0.00356295	3	1.208783888	0.257601337	4.692459687	2.70E-06	18.21597569	3.881967434	intronic	KCNIP1	.				1		
3M	rs72837643	5	170761951	T	C	421	0.00356295	4	1.251913633	0.271928683	4.60383075	4.15E-06	16.93028738	3.677434793	intergenic	KCNIP1;GABRP	dist=25319;dist=21768						
12M	rs141926026	5	175205538	C	T	439	0.0022779	3	1.568445555	0.318998882	4.916774461	8.80E-07	15.41314011	3.134807227	intergenic	LINC01951;DRD1	dist=209807;dist=234499			1			
3M	rs142311947	5	177957468	G	A	421	0.00712589	8	1.813233127	0.388171459	4.671217019	2.99E-06	12.03390127	2.576181159	ncRNA_intronic	LOC100128340	.				1		
3M	rs151015676	5	177963936	T	G	421	0.0023753	3	1.054373875	0.228793957	4.608399149	4.06E-06	20.14213671	4.370744819	intergenic	LOC100128340;PROP1	dist=4136;dist=28299						
3M	rs1877768	6	16534692	C	T	421	0.01781473	15	2.650393922	0.565512508	4.686711405	2.78E-06	8.287546847	1.768307483	intronic	ATXN1	.				1		
3M	rs150586237	6	24491120	C	T	421	0.00356295	3	1.542166452	0.264483938	5.830851057	5.51E-09	22.04614425	3.780947933	intergenic	GPLD1;ALDH5A1	dist=1542;dist=3849		1				
12M	rs17875371	6	30492455	C	T	439	0.01822323	16	3.620688814	0.776176074	4.664777664	3.09E-06	6.009947767	1.288367464	intronic	HLA-E	.			1			
12M	rs12212412	6	81267112	C	T	439	0.00911162	8	2.865042865	0.571860889	5.010034645	5.44E-07	8.760932498	1.748677029	intergenic	BCKDHB;TENT5A	dist=920842;dist=478618			1			
12M	rs71568803	6	81284001	A	G	439	0.00911162	8	2.871784743	0.573932376	5.003698802	5.62E-07	8.718272413	1.74236555	intergenic	BCKDHB;TENT5A	dist=937731;dist=461729						
12M	rs12193585	6	81292327	C	T	439	0.01025057	9	2.759336125	0.592983165	4.653312755	3.27E-06	7.847293194	1.686388517	intergenic	BCKDHB;TENT5A	dist=946057;dist=453403						
3M	rs571986619	6	84969114	A	G	421	0.0023753	3	1.102004226	0.230254532	4.786026206	1.70E-06	20.78580673	4.343019831	intergenic	TBX18;LINC02535	dist=204516;dist=418105				1		
3M	rs56224400	6	97644799	T	C	421	0.01662708	14	2.845683986	0.580546566	4.901732531	9.50E-07	8.443306398	1.722514712	ncRNA_intronic	LOC101927314	.				1		
3M	rs147627638	6	98725240	A	G	421	0.00712589	6	1.675781082	0.357955603	4.681533316	2.85E-06	13.07853062	2.793642539	intergenic	MIR2113;PNKY	dist=700621;dist=104901				1		
3M	rs141326851	6	134511989	A	C	421	0.01662708	14	2.616968305	0.552323641	4.738106631	2.16E-06	8.578496884	1.810532677	intergenic	LINC01010;LOC101928304	dist=7969;dist=13329				1		
3M	rs146048121	6	141877818	G	A	421	0.0023753	3	1.082392071	0.232511623	4.655217038	3.24E-06	20.02143793	4.300860255	intergenic	MIR4465;NMBR	dist=1193935;dist=196666		1				
3M	rs142106992	6	141948748	C	A	421	0.00356295	4	1.689946394	0.272044449	6.212023076	5.23E-10	22.83458862	3.675869896	intergenic	MIR4465;NMBR	dist=1264865;dist=125736						
3M	rs72983831	6	141985963	T	G	421	0.00593824	6	1.556807425	0.31516421	4.939670734	7.83E-07	15.67332386	3.17294911	intergenic	MIR4465;NMBR	dist=1302080;dist=88521						
3M	rs72986533	6	142290121	T	C	421	0.00831354	8	2.07386378	0.395157536	5.248194939	1.54E-07	13.28127256	2.530636288	intergenic	VTA1;ADGRG6	dist=65437;dist=11798						
3M	rs73586304	6	142518288	C	T	421	0.00356295	3	1.14329832	0.250357885	4.566655922	4.96E-06	18.24051164	3.99428202	intergenic	ADGRG6;LOC153910	dist=72022;dist=8167						
12M	rs118171627	6	143294753	C	A	439	0.00341686	3	1.522870764	0.325892127	4.672928974	2.97E-06	14.33888267	3.068500023	intronic	AIG1	.			1			
12M	rs4896997	6	148362788	C	T	439	0.00683371	6	2.511111457	0.527345105	4.761799124	1.92E-06	9.029758847	1.896291425	intronic	SASH1	.			1			
12M	rs4131286	6	148367827	G	T	439	0.00683371	6	2.510967274	0.527374461	4.761260659	1.92E-06	9.028235175	1.896185868	intronic	SASH1	.						
12M	rs17078283	6	148375784	C	T	439	0.00683371	6	2.511039365	0.527359738	4.761530287	1.92E-06	9.028998503	1.896238805	intronic	SASH1	.						
12M	rs56821264	6	148382765	C	T	439	0.00683371	6	2.51782977	0.523428582	4.810264197	1.51E-06	9.18991503	1.910480309	intronic	SASH1	.						
12M	rs181832855	6	151727964	C	T	439	0.00341686	3	1.6160007	0.338696272	4.771238528	1.83E-06	14.0870713	2.95249781	intronic	ESR1	.			1			
3M	rs148532212	6	165086368	T	C	421	0.00356295	3	1.406500453	0.26958124	5.217352852	1.81E-07	19.35354569	3.709456929	intergenic	MEAT6;C6orf118	dist=264305;dist=193296				1		
3M	rs117498042	6	165100792	C	T	421	0.00356295	3	1.405430664	0.269604623	5.212932361	1.86E-07	19.33547097	3.70913521	intergenic	MEAT6;C6orf118	dist=278729;dist=178872						
3M	rs148153037	6	167087898	G	A	421	0.00831354	8	2.445317826	0.413004518	5.920801636	3.20E-09	14.33592462	2.421281019	intergenic	CEP43;CCR6	dist=35180;dist=23909		1				
3M	rs184487573	6	167099983	A	G	421	0.00712589	7	2.008477086	0.385701261	5.207338654	1.92E-07	13.50096351	2.592680141	intergenic	CEP43;CCR6	dist=47265;dist=11824						
3M	rs17776100	7	6386848	G	A	421	0.02969121	25	3.798893519	0.756560221	5.021270496	5.13E-07	6.63697397	1.321771845	intronic	RAC1	.				1		
3M	rs187978759	7	11672218	G	A	421	0.00356295	3	1.07420489	0.229159549	4.687585114	2.76E-06	20.4555522	4.3637719	intronic	THSD7A	.				1		
3M	rs117166500	7	17013154	G	T	421	0.00712589	7	1.813506071	0.380670761	4.763975219	1.90E-06	12.51468647	2.626941975	intergenic	AGR3;AHR	dist=131171;dist=285498				1		
3M	rs75689761	7	18366950	C	T	421	0.00831354	7	2.0345491	0.414387092	4.909779146	9.12E-07	11.84829172	2.413202584	intronic	HDAC9	.		1				
3M	rs77346868	7	18366976	A	G	421	0.00950119	8	2.148608551	0.441537806	4.866193838	1.14E-06	11.02101286	2.264811725	intronic	HDAC9	.						
3M	rs78225611	7	18367841	A	C	421	0.00950119	8	2.147570798	0.441262518	4.866877908	1.13E-06	11.02943875	2.266224663	intronic	HDAC9	.						
3M	rs77300464	7	18369138	A	G	421	0.00712589	6	2.141507953	0.382123995	5.604222657	2.09E-08	14.66597943	2.616951597	intronic	HDAC9	.						
3M	rs79602997	7	18370627	G	A	421	0.00831354	7	2.026922666	0.413391185	4.903158896	9.43E-07	11.86082112	2.419016265	intronic	HDAC9	.						
3M	rs75773869	7	18371222	G	T	421	0.00831354	7	2.02750066	0.413389996	4.904571175	9.36E-07	11.86427156	2.41902322	intronic	HDAC9	.						
3M	rs75606013	7	18374990	G	A	421	0.00712589	6	2.140929626	0.382127995	5.602650559	2.11E-08	14.66171186	2.616924205	intronic	HDAC9	.						
3M	rs61434999	7	18378728	A	G	421	0.00831354	7	2.033933624	0.414449316	4.907556958	9.22E-07	11.84115106	2.412840272	intronic	HDAC9	.						
3M	rs78907958	7	18385394	T	G	421	0.00831354	7	2.090002735	0.415774551	5.026769271	4.99E-07	12.09013217	2.405149614	intronic	HDAC9	.						
3M	rs76526501	7	18391487	G	A	421	0.00712589	6	2.233180137	0.387925975	5.75671721	8.58E-09	14.83973124	2.577811398	intronic	HDAC9	.						
3M	rs74455595	7	18392161	A	G	421	0.00712589	6	2.233180137	0.387925975	5.75671721	8.58E-09	14.83973124	2.577811398	intronic	HDAC9	.						
3M	rs79182806	7	18394204	T	C	421	0.00831354	7	2.11859485	0.418485528	5.062528352	4.14E-07	12.09726027	2.389568893	intronic	HDAC9	.						
3M	rs10279777	7	18401966	G	A	421	0.00950119	8	2.476234721	0.438670193	5.644866606	1.65E-08	12.86813351	2.279616935	intronic	HDAC9	.						
3M	rs77867199	7	18402652	G	T	421	0.01068884	9	2.377239679	0.468684022	5.072158572	3.93E-07	10.82212821	2.13363365	intronic	HDAC9	.						
3M	rs80156375	7	18403592	A	C	421	0.00950119	8	2.260074467	0.442534058	5.107119835	3.27E-07	11.5406255	2.25971308	intronic	HDAC9	.						
3M	rs17169602	7	18407118	G	A	421	0.00950119	8	2.49198241	0.442489656	5.631730316	1.78E-08	12.72737168	2.259939835	intronic	HDAC9	.						
3M	rs10486295	7	18407184	G	A	421	0.00950119	8	2.488096085	0.441539202	5.635051363	1.75E-08	12.76229003	2.264804562	intronic	HDAC9	.						
3M	rs75090694	7	18407813	A	G	421	0.00831354	7	2.37298061	0.41420374	5.729017827	1.01E-08	13.83140053	2.414270813	intronic	HDAC9	.						
3M	rs55844051	7	23320744	T	C	421	0.00356295	3	1.232383167	0.25888714	4.760310483	1.93E-06	18.3875896	3.862687038	intronic	IGF2BP3	.				1		
3M	rs62447184	7	36534898	G	A	421	0.04275534	43	4.329544629	0.918817438	4.712083651	2.45E-06	5.128422094	1.088355486	intronic	AOAH	.				1		
3M	rs574076561	7	49505151	A	G	421	0.00356295	3	1.086373837	0.235169216	4.619541003	3.85E-06	19.64347663	4.25225723	intergenic	CDC14C;VWC2	dist=577697;dist=268487				1		
12M	rs564332768	7	64988831	A	C	439	0.00797267	6	2.356215761	0.507434013	4.643393431	3.43E-06	9.150733523	1.970699589	intronic	ERV3-1-ZNF117;ZNF117	.			1			
12M	rs533568586	7	66745708	G	A	439	0.00797267	7	2.458442137	0.536776292	4.580012523	4.65E-06	8.532441903	1.862973488	intronic	RABGEF1	.			1			
12M	rs189433222	7	67186631	C	T	439	0.00683371	7	2.388728197	0.523042224	4.566989217	4.95E-06	8.73158802	1.911891534	intronic	TYW1	.			1			
12M	rs566394355	7	67352900	T	G	439	0.00683371	6	2.423461328	0.526255379	4.605105094	4.12E-06	8.750704076	1.900218105	intergenic	LINC01372;LOC102723427	dist=12875;dist=667353						
12M	rs577438374	7	67418626	C	T	439	0.00683371	6	2.39527184	0.515335444	4.647985827	3.35E-06	9.019340475	1.940483644	intergenic	LINC01372;LOC102723427	dist=78601;dist=601627						
12M	rs548247109	7	67561468	G	A	439	0.00683371	6	2.378475952	0.500107071	4.755933457	1.98E-06	9.509830457	1.999571807	intergenic	LINC01372;LOC102723427	dist=221443;dist=458785						
3M	rs111391231	7	89867731	T	C	421	0.01425178	12	2.546596314	0.553340001	4.602227036	4.18E-06	8.317177547	1.807207138	intergenic	ZNF804B;STEAP2-AS1	dist=529203;dist=14622				1		
3M	rs111900874	7	89887355	G	A	421	0.01425178	12	2.536039096	0.548671755	4.622142612	3.80E-06	8.424240128	1.822583342	ncRNA_intronic	STEAP2-AS1	.						
3M	rs181259864	7	97859511	C	A	421	0.00356295	3	1.263697226	0.271559968	4.653473908	3.26E-06	17.13608209	3.682427888	ncRNA_intronic	CZ1P-ASNS	.				1		
3M	rs192750513	7	97948518	A	G	421	0.00356295	3	1.238714279	0.26844613	4.614386809	3.94E-06	17.18924692	3.725142178	ncRNA_intronic	CZ1P-ASNS	.						
3M	rs539713344	7	100877165	G	A	421	0.0023753	3	1.125805228	0.236990667	4.750420101	2.03E-06	20.04475604	4.219575451	intronic	SRRT	.				1		
3M	rs188028357	7	101025144	C	T	421	0.0023753	3	1.150125852	0.229467369	5.012154263	5.38E-07	21.84255775	4.357918094	intronic	MUC17	.				1		
12M	rs181182636	7	116014504	A	G	439	0.00341686	3	1.592950272	0.347387686	4.585511625	4.53E-06	13.19998323	2.878628233	intronic	TFEC	.			1			
12M	rs182211730	7	116363561	G	A	439	0.00455581	4	1.988619676	0.418066319	4.756708646	1.97E-06	11.37788056	2.391964991	intergenic	LOC102724434;CAV2	dist=76834;dist=136177			1			
12M	rs143281973	7	116367942	C	T	439	0.00455581	4	2.006625392	0.427483854	4.694037855	2.68E-06	10.98062023	2.339269638	intergenic	LOC102724434;CAV2	dist=81215;dist=131796						
12M	rs138873576	7	116385305	G	T	439	0.00455581	4	1.991831401	0.430896074	4.622533181	3.79E-06	10.72772174	2.320745209	intergenic	LOC102724434;CAV2	dist=98578;dist=114433						
12M	rs190354334	7	116435792	G	A	439	0.00455581	5	2.00243762	0.432429385	4.630669627	3.64E-06	10.70849897	2.312516295	intergenic	LOC102724434;CAV2	dist=149065;dist=63946						
12M	rs186893139	7	116456916	C	T	439	0.00455581	4	1.880403456	0.406387212	4.627122608	3.71E-06	11.38599462	2.460707351	intergenic	LOC102724434;CAV2	dist=170189;dist=42822						
12M	rs191903483	7	116474429	A	G	439	0.00455581	5	1.937757245	0.423414829	4.576498295	4.73E-06	10.80854514	2.361750063	intergenic	LOC102724434;CAV2	dist=187702;dist=25309						
12M	rs558784715	7	117403240	A	G	439	0.00341686	3	1.805721393	0.377457708	4.783903883	1.72E-06	12.67401298	2.649303432	intronic	ASZ1	.			1			
12M	rs576047962	7	117468967	A	G	439	0.00341686	3	1.817321889	0.377176493	4.818226812	1.45E-06	12.7744621	2.651278697	intergenic	ASZ1;CFTR	dist=41474;dist=11058						
12M	rs529110230	7	117535853	T	C	439	0.00341686	3	1.806947008	0.378567349	4.773119008	1.81E-06	12.60837477	2.641537902	intronic	CFTR	.						
12M	rs78282451	7	117553939	C	A	439	0.00455581	4	2.053353534	0.436106063	4.708381076	2.50E-06	10.79641279	2.293020173	ncRNA_intronic	CFTR-AS1	.						
12M	rs142215699	7	117559820	T	C	439	0.00341686	3	1.830346943	0.377150347	4.853096273	1.22E-06	12.86780275	2.651462495	ncRNA_intronic	CFTR-AS1	.						
12M	rs75862363	7	117570346	G	A	439	0.00455581	4	2.053944777	0.436108133	4.709714457	2.48E-06	10.79941901	2.293009291	intronic	CFTR	.						
12M	rs77846488	7	117571897	T	C	439	0.00455581	4	2.054832011	0.436103169	4.71180252	2.46E-06	10.80432993	2.293035389	intronic	CFTR	.						
12M	rs142721557	7	117578274	A	C	439	0.00341686	3	1.830638975	0.377148582	4.853893297	1.21E-06	12.86997625	2.651474902	intronic	CFTR	.						
12M	rs201355675	7	117585727	G	A	439	0.00341686	3	1.830359186	0.377152899	4.853095897	1.22E-06	12.86771469	2.651444555	intronic	CFTR	.						
12M	rs188993522	7	117634677	T	C	439	0.00341686	3	1.746488875	0.364012714	4.797878776	1.60E-06	13.18052527	2.747156793	intronic	CFTR	.						
12M	rs190601781	7	132751214	G	A	439	0.00341686	3	1.55039666	0.338020811	4.586689956	4.50E-06	13.56925315	2.958397728	intergenic	FLJ40288;LOC100506937	dist=22446;dist=7756			1			

3M	rs536023430	7	147167980	T	C	421	0.00356295	3	1.137129093	0.242081012	4.697308079	2.64E-06	19.40386834	4.130848564	intronic	CNTNAP2	.				1		
3M	rs187384541	8	1684075	A	G	421	0.03562945	26	3.153891836	0.676696813	4.660716257	3.15E-06	6.887451174	1.477766677	intronic	DLGAP2	.				1		
3M	rs575473987	8	5719972	C	T	421	0.00356295	3	1.471553256	0.257316966	5.718834929	1.07E-08	22.22486534	3.886257536	intergenic	CSMD1;LOC100287015	dist=725058;dist=683583		1				
3M	rs139062456	8	13394482	C	T	421	0.01781473	15	2.721124769	0.589318868	4.617406495	3.89E-06	7.835158086	1.696874229	intronic	DLC1	.				1		
3M	rs185874707	8	18173165	C	T	421	0.01187648	10	2.405517016	0.513034555	4.688801158	2.75E-06	9.139347655	1.949186444	intronic	NAT1	.				1		
3M	rs140797780	8	22230279	C	T	421	0.00356295	3	1.415632732	0.270894217	5.225776871	1.73E-07	19.29083956	3.691477848	intronic	PHYHIP	.				1		
3M	rs147601511	8	22464806	A	G	421	0.00475059	4	1.494160188	0.301407236	4.957280417	7.15E-07	16.44711814	3.317770381	intronic	PPP3CC	.				1		
3M	rs188415494	8	25755782	C	T	421	0.00356295	3	1.162251387	0.250533229	4.639110717	3.50E-06	18.51694779	3.991486498	intergenic	CDCA2;EBF2	dist=247865;dist=85943				1		
12M	rs111896192	8	37846591	C	T	439	0.00341686	4	1.834661883	0.379554059	4.833730106	1.34E-06	12.7352876	2.634670807	intronic	BRF2	.			1			
3M	rs9643828	8	54616513	C	T	421	0.67695962	279	-11.04343772	2.134612444	-5.173509483	2.30E-07	-2.423629403	0.468469114	intronic	RP1	.				1		
3M	rs423841	8	54643509	G	A	421	0.66270784	291	-10.22664529	2.181175145	-4.688594272	2.75E-06	-2.149572575	0.458468456	intronic	RP1	.						
3M	rs433324	8	54652049	A	G	421	0.66627078	287	-10.71178821	2.182726874	-4.907525693	9.22E-07	-2.248346209	0.458142524	intronic	RP1	.						
3M	rs369623	8	54659380	A	C	421	0.66627078	287	-10.8407396	2.201888659	-4.923382276	8.51E-07	-2.235981486	0.45415557	intronic	RP1	.						
3M	rs446222	8	54662400	G	A	421	0.66627078	288	-10.90656484	2.202740432	-4.951361803	7.37E-07	-2.247819003	0.453979954	intronic	RP1	.						
3M	rs432393	8	54667738	C	T	421	0.66270784	291	-10.75654812	2.217474543	-4.850810194	1.23E-06	-2.187538166	0.450963464	intronic	RP1	.						
3M	rs3098298	8	54670278	C	T	421	0.66270784	291	-10.75741494	2.217551047	-4.851033737	1.23E-06	-2.187563503	0.450947906	intronic	RP1	.						
3M	rs367179	8	54675056	T	C	421	0.66270784	291	-10.75741494	2.217551047	-4.851033737	1.23E-06	-2.187563503	0.450947906	intronic	RP1	.						
3M	rs382476	8	54678415	G	A	421	0.66627078	288	-10.90475865	2.202374434	-4.951364528	7.37E-07	-2.24819379	0.454055398	intronic	RP1	.						
3M	rs384543	8	54679049	G	A	421	0.66627078	288	-10.90475865	2.202374434	-4.951364528	7.37E-07	-2.24819379	0.454055398	intronic	RP1	.						
3M	rs405226	8	54679776	A	G	421	0.66270784	291	-10.75682372	2.217202771	-4.851529081	1.23E-06	-2.188130533	0.45101874	intronic	RP1	.						
3M	rs384127	8	54684929	G	A	421	0.66627078	288	-10.90492469	2.202409773	-4.951360471	7.37E-07	-2.248155875	0.454048112	intronic	RP1	.						
3M	rs858397	8	54702130	A	G	421	0.33135392	278	11.01774054	2.2226993	4.956919066	7.16E-07	2.230134803	0.449903412	intronic	RP1	.						
3M	rs2375537	8	54706948	C	T	421	0.33254157	280	11.08575521	2.225246111	4.981810848	6.30E-07	2.238768477	0.449388495	intronic	RP1	.						
3M	rs720372	8	54716077	G	A	421	0.34679335	292	10.41667706	2.227853301	4.675656629	2.93E-06	2.098727338	0.448862589	intronic	RP1	.						
3M	rs1437781	8	54717292	T	C	421	0.33254157	280	11.08371192	2.225151228	4.981105007	6.32E-07	2.23854673	0.449407657	intronic	RP1	.						
3M	rs1595406	8	54718055	A	G	421	0.3456057	291	10.45008066	2.230645943	4.684777831	2.80E-06	2.100188892	0.448300638	intronic	RP1	.						
3M	rs1437782	8	54720202	C	T	421	0.33016627	278	11.10161994	2.217354849	5.006695229	5.54E-07	2.2579585	0.450987807	exonic	RP1	.						
3M	rs10105693	8	54727912	C	T	421	0.32897862	276	11.34015363	2.223938019	5.099132049	3.41E-07	2.292839101	0.449652819	intronic	RP1	.						
3M	rs2375536	8	54728162	T	C	421	0.347981	292	10.56754692	2.229301935	4.740294148	2.13E-06	2.126358065	0.448570911	intronic	RP1	.						
3M	rs4737674	8	54749094	C	A	421	0.33016627	277	11.42523599	2.226081333	5.132443196	2.86E-07	2.305595541	0.449219885	intronic	RP1	.						
3M	rs11987234	8	54757269	A	G	421	0.32897862	276	11.39664661	2.227939022	5.115331478	3.13E-07	2.295992587	0.448845319	intronic	RP1	.						
3M	rs13277510	8	54761589	G	A	421	0.33016627	277	11.42815338	2.22667847	5.132377007	2.86E-07	2.304947516	0.449099416	intronic	RP1	.						
3M	rs12548593	8	54762057	G	T	421	0.33254157	279	11.39766972	2.234516943	5.100730946	3.38E-07	2.282699606	0.447524018	intronic	RP1	.						
3M	rs1812506	8	54763541	A	G	421	0.3456057	291	10.64127813	2.239050947	4.752584188	2.01E-06	2.122588678	0.446617796	intronic	RP1	.						
3M	rs16920698	8	54765874	G	A	421	0.33016627	277	11.42894943	2.226765953	5.132532863	2.86E-07	2.304926953	0.449081772	intronic	RP1	.						
3M	rs1561297	8	54765978	A	C	421	0.33254157	279	11.40464743	2.234979191	5.102798037	3.35E-07	2.28315237	0.447431459	intronic	RP1	.						
3M	rs4737676	8	54766986	G	A	421	0.33016627	277	11.42891023	2.226765217	5.132516953	2.86E-07	2.304920569	0.44908192	intronic	RP1	.						
3M	rs2083123	8	54767758	C	T	421	0.33254157	279	11.40261082	2.236761629	5.097821184	3.44E-07	2.279107938	0.447074908	intronic	RP1	.						
3M	rs983248	8	54768232	C	T	421	0.33016627	277	11.43612477	2.228588003	5.131556283	2.87E-07	2.302604284	0.448714611	intronic	RP1	.						
3M	rs1391463	8	54769316	T	G	421	0.33016627	277	11.43612548	2.228595636	5.131539026	2.87E-07	2.302588654	0.448713075	intronic	RP1	.						
3M	rs10958428	8	54773081	A	G	421	0.33372922	280	11.27671394	2.243728439	5.025881807	5.01E-07	2.239968848	0.445686734	intronic	RP1	.						
3M	rs13278605	8	54775611	C	T	421	0.32897862	277	11.43432709	2.242496155	5.09892829	3.42E-07	2.273773481	0.445931645	intronic	RP1	.						
3M	rs13276543	8	54775614	G	T	421	0.32897862	276	11.41118163	2.230992831	5.114844597	3.14E-07	2.29263157	0.448230934	intronic	RP1	.						
3M	rs7822082	8	54777660	T	C	421	0.33016627	277	11.70263936	2.228199285	5.252061357	1.50E-07	2.357087802	0.448792891	intronic	RP1	.						
3M	rs4737201	8	54778898	C	T	421	0.33016627	277	11.43417402	2.228701352	5.130420012	2.89E-07	2.301977341	0.44869179	intronic	RP1	.						
3M	rs7843693	8	54779552	G	A	421	0.39786223	334	10.46760966	2.288570748	4.573863258	4.79E-06	1.998567561	0.436953938	intronic	RP1	.						
3M	rs1396896	8	54782750	A	G	421	0.39786223	334	10.46979793	2.288829589	4.574302074	4.78E-06	1.998533266	0.436904523	intronic	RP1	.						
3M	rs2375219	8	54785735	C	T	421	0.39311164	330	10.58980707	2.26895928	4.667253025	3.05E-06	2.057001668	0.440730695	intronic	RP1	.						
3M	rs1391462	8	54787221	C	A	421	0.39786223	334	10.46683006	2.288537601	4.573588854	4.79E-06	1.998476605	0.436960267	intronic	RP1	.						
3M	rs12678939	8	54792461	A	G	421	0.39429929	331	10.32905196	2.261417548	4.567512076	4.94E-06	2.019756183	0.442200513	intronic	RP1	.						
3M	rs1498183	8	54804345	C	T	421	0.39429929	332	10.33728121	2.259624491	4.574778355	4.77E-06	2.024574602	0.442551408	intronic	RP1	.						
3M	rs117816016	8	102739034	C	T	421	0.00356295	3	1.090578449	0.234213862	4.656336053	3.22E-06	19.88070227	4.269602116	intergenic	LOC101927245;GASAL1	dist=52311;dist=67788				1		
3M	rs567383525	8	114017265	C	T	421	0.00356295	3	1.282541719	0.268838431	4.770678479	1.84E-06	17.74552267	3.719706274	intergenic	CSMD3;TRPS1	dist=580326;dist=1391230				1		
3M	rs545550279	8	114540200	G	T	421	0.00356295	3	1.091458604	0.238542228	4.57553622	4.75E-06	19.18124208	4.192129875	intergenic	CSMD3;TRPS1	dist=1103261;dist=868295						
3M	rs536803366	8	121989172	T	C	421	0.0023753	3	1.194921552	0.249406377	4.791062547	1.66E-06	19.20986385	4.009520573	intergenic	HAS2-AS1;SMILR	dist=343847;dist=425155				1		
12M	rs139457011	8	124378581	C	T	439	0.00341686	3	1.698245565	0.326123918	5.207362812	1.92E-07	15.96743605	3.0663191	intergenic	TMEM65;TRMT12	dist=5882;dist=72239			1			
3M	rs532513136	8	134801505	C	A	421	0.00356295	3	1.227599638	0.248065954	4.948682463	7.47E-07	19.94905941	4.031185989	upstream	MIR30B	dist=898				1		
3M	rs532730683	9	1784492	G	T	421	0.00356295	3	1.275657724	0.260996603	4.887641107	1.02E-06	18.72683804	3.831467497	intergenic	DMRT2;SMARCA2	dist=726938;dist=230855				1		
3M	rs540065886	9	2770228	T	C	421	0.00356295	3	1.13736568	0.23877586	4.763319367	1.90E-06	19.94891509	4.188028045	intergenic	KCNV2;PUM3	dist=40191;dist=33927				1		
3M	rs543844012	9	30107025	C	T	421	0.00356295	3	1.164274175	0.253510467	4.592607905	4.38E-06	18.11604845	3.944610301	intergenic	LINGO2;LINC01242	dist=893424;dist=281910				1		
12M	rs79914278	9	37658199	C	T	439	0.05125285	45	7.463015515	1.54104852	4.842816705	1.28E-06	3.142546548	0.648908835	intronic	FRMPD1	.			1			
12M	rs10973466	9	37661113	G	A	439	0.05125285	45	7.461346315	1.537487847	4.852946531	1.22E-06	3.156412936	0.650411645	intronic	FRMPD1	.						
12M	rs1442156642	9	37664627	G	A	439	0.15261959	134	10.58008025	2.239682708	4.723919246	2.31E-06	2.109191284	0.446491816	intronic	FRMPD1	.						
12M	rs2057644	9	37665983	G	A	439	0.15261959	134	10.57853162	2.239752645	4.723080313	2.32E-06	2.108750858	0.446477874	intronic	FRMPD1	.						
12M	rs2057643	9	37666013	T	C	439	0.15261959	134	10.57853162	2.239752645	4.723080313	2.32E-06	2.108750858	0.446477874	intronic	FRMPD1	.						
12M	rs10973475	9	37666364	C	T	439	0.05125285	45	7.456175961	1.536478706	4.852768822	1.22E-06	3.158370371	0.650838828	intronic	FRMPD1	.						
12M	rs10814594	9	37666670	T	C	439	0.15261959	134	10.57879091	2.239737923	4.723227126	2.32E-06	2.108830269	0.446480809	intronic	FRMPD1	.						
12M	rs1125576	9	37667822	T	C	439	0.15261959	134	10.57668904	2.239823833	4.722107554	2.33E-06	2.108249535	0.446463684	intronic	FRMPD1	.						
12M	rs60006744	9	37672694	G	T	439	0.05125285	45	7.444177374	1.53385479	4.853247792	1.21E-06	3.164085559	0.651952197	intronic	FRMPD1	.						
12M	rs2148140	9	37673842	C	T	439	0.05125285	45	7.444177374	1.53385479	4.853247792	1.21E-06	3.164085559	0.651952197	intronic	FRMPD1	.						
12M	rs113399724	9	37674360	C	T	439	0.05125285	45	7.444177374	1.53385479	4.853247792	1.21E-06	3.164085559	0.651952197	intronic	FRMPD1	.						
12M	rs12350891	9	79989706	G	A	439	0.00455581	4	1.919901525	0.416077273	4.614290779	3.94E-06	11.08998515	2.403399716	ncRNA_intronic	LINC01507	.			1	1		1
3M	rs148556485	9	81408911	A	C	421	0.0023753	3	1.17470554	0.234401563	5.011508991	5.40E-07	21.38001525	4.266183157	intergenic	LINC01507;TLE1	dist=1374356;dist=174772						
3M	rs140782222	9	81413979	T	C	421	0.0023753	3	1.1680569	0.231519821	5.045170192	4.53E-07	21.79152597	4.31928461	intergenic	LINC01507;TLE1	dist=1379424;dist=169704						
3M	rs188034471	9	83322799	G	A	421	0.00475059	4	1.487832555	0.317535808	4.68555835	2.79E-06	14.75599992	3.149251127	intronic	FRMD3	.				1		
3M	rs190294315	9	83330550	C	T	421	0.00593824	5	1.754632626	0.347069598	5.055564181	4.29E-07	14.56642764	2.881266486	intronic	FRMD3	.						
3M	rs545690161	9	90268417	G	A	421	0.00593824	6	1.900812423	0.332979511	5.708496652	1.14E-08	17.14368742	3.003187786	intergenic	MIR4290HG;LINC01508	dist=226918;dist=32479		1				
3M	rs565682685	9	90460046	T	C	421	0.00475059	4	1.368085345	0.268772597	5.090122138	3.58E-07	18.93839699	3.720617399	intergenic	LINC01508;LINC01501	dist=26557;dist=2386						
3M	rs183737367	9	90567765	T	C	421	0.00356295	3	1.295690386	0.235587115	5.499835526	3.80E-08	23.34523057	4.244714311	ncRNA_intronic	LINC01501	.						
3M	rs187213609	9	90653183	C	T	421	0.00356295	3	1.269834136	0.233965931	5.427431802	5.72E-08	23.19753039	4.274126556	intergenic	DIRAS2;SYK	dist=10359;dist=148417						
3M	rs150027952	9	100623134	A	G	421	0.0023753	3	1.072594942	0.221501288	4.842386925	1.28E-06	21.86166485	4.514646431	intergenic	CAVIN4;PLPPR1	dist=34747;dist=405593				1		
3M	rs146207930	9	126280057	A	G	421	0.00712589	6	1.87537986	0.397610416	4.716626584	2.40E-06	11.86243214	2.515024654	intergenic	LOC101929116;MVB12B	dist=4142;dist=46772				1		
3M	rs78296164	9	132391328	C	T	421	0.00831354	7	2.121725249	0.427501046	4.963087859	6.94E-07	11.60953385	2.339175566	intronic	TTF1	.				1		
3M	rs77871739	9	135660463	G	A	421	0.00475059	4	1.439297375	0.309017583	4.657655271	3.20E-06	15.07246035	3.236061811	intergenic	GLT6D1;LCN9	dist=20923;dist=2859			1	1		1

12M	rs76294395	9	135780517	G	A	439	0.00569476	5	2.250462353	0.487505863	4.616277515	3.91E-06	9.469173331	2.051257382	intronic	KCNT1	.						
12M	rs62582460	9	138095976	T	C	439	0.05808656	51	6.819364418	1.458397024	4.675931387	2.93E-06	3.206212925	0.68568434	intronic	CACNA1B	.			1			
3M	rs118040657	10	3430654	C	T	421	0.00831354	8	1.732520372	0.378165204	4.581384943	4.62E-06	12.11477125	2.644346939	ncRNA_intronic	LOC105376360	.				1		
12M	rs187982129	10	10793335	G	T	439	0.00455581	4	2.104360473	0.43943772	4.788757028	1.68E-06	10.89746466	2.275635325	ncRNA_intronic	SFTA1P	.			1			
12M	rs117285875	10	10833324	G	T	439	0.00455581	4	2.102691811	0.434748428	4.836571393	1.32E-06	11.1249888	2.30018083	intronic	CELF2	.			1			
3M	rs184425183	10	13415520	A	G	421	0.00356295	3	1.590271029	0.264865828	6.004062666	1.92E-09	22.66831744	3.775496477	intergenic	SEPHS1;BEND7	dist=67222;dist=22961		1				
3M	rs184458518	10	13429195	T	G	421	0.00356295	3	1.603233248	0.265164814	6.046176429	1.48E-09	22.80157891	3.771239424	intergenic	SEPHS1;BEND7	dist=80897;dist=9286						
3M	rs117998251	10	13455976	C	T	421	0.00356295	3	1.610926128	0.264914355	6.080931816	1.19E-09	22.95433112	3.774804885	intronic	BEND7	.						
3M	rs117025967	10	19918123	C	A	421	0.01306413	11	2.602875018	0.518823163	5.016882829	5.25E-07	9.669735635	1.927439002	intronic	PLXDC2	.				1		
3M	rs529011661	10	20083387	G	A	421	0.00475059	4	1.425272643	0.301883703	4.721263948	2.34E-06	15.63934688	3.312533901	intronic	PLXDC2	.						
12M	rs142956968	10	23201236	C	T	439	0.01594533	14	3.953608313	0.770445563	5.131586839	2.87E-07	6.660544343	1.297950235	downstream	C10orf67	dist=680			1			
12M	rs145764464	10	23312918	G	A	439	0.01708428	15	4.204896484	0.829087356	5.071717058	3.94E-07	6.11722881	1.20614552	intronic	C10orf67	.						
3M	rs12266995	10	24563854	T	C	421	0.03087886	26	3.568935699	0.773323681	4.615060663	3.93E-06	5.967825345	1.293119589	intergenic	KIAA1217;ARHGAP21	dist=16006;dist=19760				1		
12M	rs74384278	10	44647124	T	C	439	0.00911162	8	2.588932233	0.559803403	4.624716857	3.75E-06	8.261323236	1.786341411	intergenic	CXCL12;TMEM72-AS1	dist=262027;dist=163900			1			
3M	rs138249376	10	61756071	T	G	421	0.00475059	4	1.271516185	0.278189815	4.57067843	4.86E-06	16.43007109	3.594667912	intronic	CABCOCO1	.				1		
3M	rs140277951	10	80599344	G	A	421	0.00831354	6	1.967344821	0.378309358	5.200359916	1.99E-07	13.74631584	2.643339318	intronic	SH2D4B	.				1		
3M	rs566018180	10	84995296	C	T	421	0.00356295	3	1.136146605	0.247392568	4.592484792	4.38E-06	18.56355199	4.04215862	intergenic	CCSER2;LINC01519	dist=476775;dist=198125				1		
3M	rs140706881	10	94457778	G	A	421	0.00475059	4	1.226743807	0.254265992	4.824647601	1.40E-06	18.97480496	3.932889306	intronic	TBC1D12	.				1		
3M	rs117913371	10	101158729	G	A	421	0.02494062	21	3.968654489	0.710300363	5.587290525	2.31E-08	7.866095548	1.407845116	intergenic	TLX1NB;LINC01514	dist=17463;dist=17593		1				
3M	rs75334617	10	101196395	G	A	421	0.03800475	32	4.196585386	0.847261284	4.953118316	7.30E-07	5.846034048	1.180273451	intergenic	LINC01514;LBX1	dist=2248;dist=30581						
3M	rs752259256	10	103165562	T	C	421	0.00356295	3	1.029573618	0.224511445	4.58584023	4.52E-06	20.42586391	4.454115907	intronic	NT5C2	.				1		
12M	rs147944608	10	108663951	C	T	439	0.01252847	11	3.51284077	0.711541711	4.936942858	7.94E-07	6.938374489	1.405398986	intergenic	LINC01435;XPNPEP1	dist=594658;dist=1200815			1			
3M	rs180828621	10	122773893	G	A	421	0.00712589	6	1.931459198	0.357196912	5.407267342	6.40E-08	15.13805735	2.799576273	ncRNA_intronic	DMBT1L1	.				1		
3M	rs147393020	10	123019758	A	G	421	0.00593824	5	1.619245544	0.337859644	4.792657467	1.65E-06	14.1853505	2.959808958	intronic	ACADSB	.				1		
3M	rs193093906	10	125016920	G	A	421	0.00950119	9	2.062968829	0.442790343	4.659019469	3.18E-06	10.52195366	2.25840517	intronic	CTBP2	.				1		
12M	rs148815783	11	1013992	C	T	439	0.00797267	8	2.639026369	0.568648462	4.640874893	3.47E-06	8.161237049	1.758555711	exonic	MUC6	.			1			
12M	rs150788978	11	18175633	G	A	439	0.00797267	8	2.532677712	0.547949348	4.622101879	3.80E-06	8.435272154	1.824986202	intergenic	MRGPRX4;SLC25A51P4	dist=1353;dist=33505			1			
3M	rs151115079	11	18634194	T	C	421	0.00475059	5	1.669234911	0.305995633	5.455093905	4.89E-08	17.8273587	3.268020498	intronic	SPTY2D1	.		1				
3M	rs138414342	11	18657851	G	A	421	0.00475059	5	1.685610798	0.304678389	5.532426519	3.16E-08	18.15825054	3.282149429	intergenic	SPTY2D1;TMEM86A	dist=23509;dist=40928						
3M	rs541653703	11	18680239	G	A	421	0.00475059	4	1.710453669	0.305582922	5.597347058	2.18E-08	18.31694986	3.272434185	intergenic	SPTY2D1;TMEM86A	dist=45897;dist=18540						
3M	rs118093638	11	18696777	C	T	421	0.00593824	5	1.681215257	0.343292823	4.897321308	9.72E-07	14.26572587	2.912965064	intergenic	SPTY2D1;TMEM86A	dist=62435;dist=2002						
12M	rs192072893	11	24171605	G	A	439	0.01138952	10	2.98365585	0.63649116	4.687662667	2.76E-06	7.364851171	1.571113729	intergenic	MIR8054;LUZP2	dist=752415;dist=325448			1			
12M	rs183894319	11	24243274	T	C	439	0.01252847	11	3.441856532	0.682038209	5.046427729	4.50E-07	7.399039613	1.466193516	intergenic	MIR8054;LUZP2	dist=824084;dist=253779						
12M	rs146347965	11	24443383	C	A	439	0.01366743	12	3.980373837	0.744795958	5.344247369	9.08E-08	7.175451632	1.342649607	intergenic	MIR8054;LUZP2	dist=1024193;dist=53670						
12M	rs186629933	11	24499372	A	G	439	0.01366743	12	3.971746912	0.745783477	5.325603253	1.01E-07	7.14095098	1.340871755	intronic	LUZP2	.						
12M	rs72874841	11	24549456	C	T	439	0.02277904	20	4.439777519	0.9297034	4.775477341	1.79E-06	5.136560051	1.075611857	intronic	LUZP2	.						
12M	rs141411318	11	24584003	C	T	439	0.02277904	20	4.434973388	0.926703454	4.78575252	1.70E-06	5.164276125	1.079093853	intronic	LUZP2	.						
12M	rs72878847	11	24599192	A	G	439	0.023918	20	4.373449314	0.932123939	4.691918242	2.71E-06	5.033577665	1.072818708	intronic	LUZP2	.						
3M	rs181812512	11	66898258	C	T	421	0.00356295	3	1.08407707	0.235726848	4.598869755	4.25E-06	19.50931683	4.242198164	intronic	PC	.				1		
3M	rs529345909	11	67343381	A	G	421	0.00356295	3	1.116845445	0.244375672	4.570198967	4.87E-06	18.70153001	4.092060355	ncRNA_intronic	LOC100130987	.				1		
3M	rs544042801	11	68692775	G	A	421	0.00475059	4	1.370120555	0.293669353	4.665521075	3.08E-06	15.88698661	3.405190192	intergenic	GAL;TESMIN	dist=1600;dist=14665				1		
12M	rs139548692	11	82893220	C	T	439	0.00455581	6	1.861738012	0.402460972	4.625884601	3.73E-06	11.49399551	2.484712979	intronic	PRCP	.			1			
3M	rs149949098	11	95366702	G	A	421	0.01662708	13	2.587327209	0.549803064	4.705916312	2.53E-06	8.559276254	1.818833079	intergenic	LOC100129203;FAM76B	dist=132298;dist=402251				1		
3M	rs74521112	11	99218416	G	T	421	0.03206651	27	4.149638813	0.814414575	5.095241344	3.48E-07	6.256323868	1.227875864	intronic	CNTN5	.				1		
3M	rs79213709	11	99222724	G	A	421	0.03206651	26	4.048879944	0.810020577	4.998490236	5.78E-07	6.17081884	1.23453654	intronic	CNTN5	.						
3M	rs112007361	11	99317649	A	C	421	0.03087886	26	4.213518738	0.814161472	5.175286336	2.28E-07	6.356584678	1.228257582	intronic	CNTN5	.						
12M	rs183749982	11	102098824	G	A	439	0.00455581	4	1.971855551	0.409991583	4.80950252	1.51E-06	11.73073478	2.439074464	intergenic	CFAP300;YAP1	dist=14264;dist=11623			1			
3M	rs148781275	11	103769875	A	G	421	0.00475059	5	1.423809766	0.301038131	4.729665848	2.25E-06	15.71118528	3.321838325	intergenic	DYNC2H1;MIR4693	dist=290012;dist=80031				1		
3M	rs141281289	11	123822316	A	G	421	0.00593824	6	1.657109443	0.323734372	5.118731856	3.08E-07	15.81151802	3.088952198	intergenic	OR6M1;TMEM225	dist=15967;dist=60603				1		
3M	rs79539453	11	125396457	C	T	421	0.0023753	3	1.102705134	0.230127137	4.791721423	1.65E-06	20.82206158	4.345424064	intronic	PKNOX2	.				1		
3M	rs528140343	11	125849332	A	C	421	0.00356295	3	1.315991741	0.268284941	4.905201681	9.33E-07	18.28355207	3.727380292	intergenic	PATE4;HYLS1	dist=9260;dist=34282				1		
3M	rs546409459	11	125885094	A	G	421	0.00356295	3	1.180777655	0.249820856	4.726497517	2.28E-06	18.91954737	4.002868361	intronic	HYLS1	.						
3M	rs528609331	11	125972300	C	T	421	0.00356295	3	1.256070429	0.253271825	4.959376858	7.07E-07	19.58124182	3.948327054	intronic	CDON	.				1		
3M	rs7104959	11	129976231	C	T	421	0.00356295	3	1.212990302	0.260168118	4.66233262	3.13E-06	17.92046105	3.843668505	intronic	PRDM10	.				1		
12M	rs117699122	12	451096	T	C	439	0.00341686	4	1.919399067	0.369742041	5.191184271	2.09E-07	14.04001627	2.704588306	intergenic	CCDC77;B4GALNT3	dist=8456;dist=8843			1			
3M	rs189360484	12	1761344	A	G	421	0.00475059	4	1.530180886	0.301727521	5.071399785	3.95E-07	16.8078794	3.314248553	intronic	ADIPOR2	.				1		
3M	rs141754456	12	19998198	T	C	421	0.00712589	7	2.240311418	0.429474084	5.21640654	1.82E-07	12.14603335	2.328429207	intergenic	AEBP2;LINC02398	dist=475971;dist=16487				1		
3M	rs118184666	12	20271815	G	A	421	0.00712589	6	2.026245701	0.430149631	4.710560123	2.47E-06	10.95098026	2.324772421	intergenic	LINC02468;PDE3A	dist=143914;dist=96722				1		
3M	rs549931083	12	20363352	A	C	421	0.00356295	3	1.829003959	0.357281438	5.119224688	3.07E-07	14.32826937	2.798913946	intergenic	LINC02468;PDE3A	dist=235451;dist=5185						
3M	rs151323346	12	20859090	T	C	421	0.00593824	4	1.39062978	0.295827471	4.700813538	2.59E-06	15.89038883	3.380348678	intronic	SLCO1B3;SLCO1B3-SLCO1B7	.						
3M	rs371879555	12	22863028	T	C	421	0.00356295	3	1.139634583	0.238093921	4.786491727	1.70E-06	20.10337646	4.200023233	intergenic	ETNK1;LOC101928441	dist=172363;dist=312608				1		
3M	rs183466664	12	26668754	A	G	421	0.00475059	4	1.341044738	0.289344304	4.634771518	3.57E-06	16.01818822	3.456090156	intronic	ITPR2	.				1		
3M	rs77353774	12	28095919	G	A	421	0.00712589	6	1.82528489	0.373198242	4.890925749	1.00E-06	13.1054362	2.679541026	intergenic	PTHLH;LOC729291	dist=123186;dist=89706				1		
3M	rs113167689	12	28283029	C	T	421	0.00712589	6	1.892111617	0.365850586	5.171815182	2.32E-07	14.13641354	2.733356286	intronic	CCDC91	.				1		
3M	rs17510814	12	28316036	A	C	421	0.00712589	6	1.905807603	0.368446684	5.17254649	2.31E-07	14.03879235	2.714096892	intronic	CCDC91	.						
3M	rs141756120	12	28358163	A	C	421	0.00831354	6	1.868183897	0.361717894	5.164753879	2.41E-07	14.27840304	2.764585375	intronic	CCDC91	.						
3M	rs117991215	12	28358540	T	C	421	0.00831354	6	1.855759228	0.359817411	5.15750259	2.50E-07	14.33366601	2.77918736	intronic	CCDC91	.						
3M	rs191930622	12	47890872	G	A	421	0.0023753	3	1.061255347	0.232228501	4.569875551	4.88E-06	19.67835791	4.306103677	intronic	VDR	.				1		
3M	rs56302696	12	47899047	G	A	421	0.0023753	3	1.068148373	0.231704225	4.60996503	4.03E-06	19.89590409	4.315847075	intronic	VDR	.						
3M	rs185620578	12	48175616	C	T	421	0.0023753	3	1.072824946	0.227528468	4.715124023	2.42E-06	20.72322668	4.39505442	intergenic	ASB8;CCDC184	dist=18101;dist=8028				1		
3M	rs190806532	12	48468364	G	T	421	0.0023753	3	1.082541559	0.225298273	4.804926128	1.55E-06	21.32695498	4.438560431	intergenic	ZNF641;ANP32D	dist=117118;dist=4195				1		
3M	rs568658857	12	49460215	G	A	421	0.00593824	5	1.486911933	0.32318174	4.600853791	4.21E-06	14.23611926	3.094234224	intronic	SPATS2	.				1		
3M	rs137880949	12	62912517	T	C	421	0.00475059	3	1.263235635	0.24178583	5.224605748	1.75E-07	21.60840343	4.135891677	intronic	PPM1H	.		1				
3M	rs191053292	12	63051500	T	C	421	0.00356295	3	1.354341299	0.243048963	5.572298207	2.51E-08	22.9266488	4.114397317	intergenic	PPM1H;AVPR1A	dist=116350;dist=91259						
3M	rs182437250	12	63214686	T	C	421	0.00475059	4	1.307028829	0.263613284	4.958129612	7.12E-07	18.80834508	3.793435539	intergenic	AVPR1A;DPY19L2	dist=63485;dist=344227						
3M	rs12315614	12	64527177	C	A	421	0.0760095	64	5.614667617	1.219220913	4.605127386	4.12E-06	3.777106623	0.820195905	intergenic	TBK1;RASSF3	dist=25064;dist=83318				1		
12M	rs185948380	12	70219358	T	C	439	0.00569476	5	2.022172064	0.437534471	4.621743424	3.81E-06	10.56315269	2.285534207	intergenic	MYRFL;PRANCR	dist=260261;dist=2832			1			
12M	rs111582203	12	72290549	A	G	439	0.00797267	8	3.07529407	0.552250588	5.568656944	2.57E-08	10.08356907	1.810772179	intronic	TRHDE	.	1					
12M	rs112507626	12	72316321	G	A	439	0.02277904	19	4.543772122	0.930816356	4.881491491	1.05E-06	5.244312112	1.074325771	intronic	TRHDE	.						
12M	rs112796175	12	72396569	C	T	439	0.02505695	21	4.562372955	0.978318805	4.663482835	3.11E-06	4.766833481	1.022161687	intronic	TRHDE	.						
12M	rs112856203	12	72465084	T	C	439	0.02505695	21	4.413662148	0.961866897	4.588641281	4.46E-06	4.770557441	1.039644886	intronic	TRHDE	.						
3M	rs76327548	12	100789188	G	A	421	0.01306413	11	2.458077916	0.517503975	4.749872531	2.04E-06	9.178427144	1.932352307	intergenic	GAS2L3;ANO4	dist=160900;dist=5588				1		
3M	rs76904423	12	100794966	G	A	421	0.01068884	10	2.190673587	0.472659243	4.634784191	3.57E-06	9.805762313	2.115689082	UTR5	ANO4	NM_001286615:c.-1068200>0;NM_178826:c.-1068200>0						
3M	rs180764936	12	101104843	T	C	421	0.00356295	3	1.197190428	0.248754746	4.812734014	1.49E-06	19.34730528	4.020023799	intronic	ANO4	.						

3M	rs185855183	12	101111348	C	T	421	0.00356295	3	1.256842073	0.258975211	4.853136594	1.22E-06	18.73977272	3.861373435	intronic	ANO4	.						
12M	rs139496704	12	124154097	A	G	439	0.00797267	7	2.256578283	0.493630452	4.571391967	4.84E-06	9.260757615	2.025806949	intronic	ZNF664-RFLNA	.			1			
12M	rs566724618	13	22889700	C	T	439	0.00341686	3	1.92789865	0.356546436	5.407146043	6.40E-08	15.16533472	2.804683764	ncRNA_intronic	LINC00621	.		1				1
12M	rs147221953	13	22911583	G	A	439	0.00341686	3	1.853658494	0.344939547	5.373864819	7.71E-08	15.57914966	2.899058719	ncRNA_intronic	LINC00621	.						
3M	rs139598422	13	23312875	A	G	421	0.00356295	4	1.544940466	0.274599144	5.626166351	1.84E-08	20.48865215	3.641671944	intronic	SGCG	.						
12M	rs567095960	13	39877533	T	G	439	0.00341686	3	1.525495529	0.320695601	4.75683334	1.97E-06	14.83286119	3.118221752	intergenic	COG6;LINC00332	dist=85867;dist=304276			1			
3M	rs143371352	13	46809699	C	T	421	0.00356295	3	1.364496992	0.257779154	5.29327904	1.20E-07	20.53416252	3.879289636	intergenic	ESD;HTR2A	dist=12538;dist=21847				1		
3M	rs75186966	13	46821623	A	C	421	0.00356295	3	1.377358459	0.260942954	5.278389159	1.30E-07	20.22813447	3.832255231	intergenic	ESD;HTR2A	dist=24462;dist=9923						
3M	rs150077525	13	57405415	A	G	421	0.00712589	7	1.765848692	0.368578785	4.790966716	1.66E-06	12.99848746	2.713124141	intergenic	PRR20E;PCDH17	dist=235197;dist=226329				1		
3M	rs534845494	13	57639730	A	G	421	0.00475059	5	1.571808362	0.313987509	5.005958262	5.56E-07	15.94317648	3.184840074	intronic	PCDH17	.						
3M	rs140062526	13	58711899	G	A	421	0.00593824	5	1.544633202	0.331067832	4.665609433	3.08E-06	14.09260875	3.020529034	intergenic	LINC00374;DIAPH3	dist=478782;dist=953688				1		
12M	rs113791989	13	61344755	G	A	439	0.0022779	3	1.593884468	0.320567115	4.972077284	6.62E-07	15.51025373	3.119471569	intergenic	MIR3169;PCDH20	dist=144874;dist=64931			1			
12M	rs117475675	13	73424715	T	C	439	0.00455581	4	2.083781896	0.384121138	5.424804025	5.80E-08	14.12263864	2.603345406	intergenic	KLF5;LINC00392	dist=347172;dist=139529			1			
12M	rs142497891	13	73462846	G	A	439	0.00455581	4	2.241850629	0.429935434	5.214389078	1.84E-07	12.12830735	2.325930644	intergenic	KLF5;LINC00392	dist=385303;dist=101398						
12M	rs368187808	13	73783371	A	C	439	0.00341686	3	1.97018281	0.370345591	5.319849503	1.04E-07	14.3645547	2.700180652	intronic	KLF12	.			1			
12M	rs372194899	13	73783373	A	G	439	0.00341686	3	1.969004602	0.370330521	5.31688449	1.06E-07	14.35713287	2.700290535	intronic	KLF12	.						
3M	rs546286713	13	90758825	G	A	421	0.00475059	4	1.389978902	0.297519732	4.671888118	2.98E-06	15.70278409	3.361121605	intergenic	LINC01049;LINC00410	dist=223484;dist=132129				1		
3M	rs567080482	13	94423151	T	C	421	0.00475059	4	1.409440819	0.301549206	4.67399944	2.95E-06	15.49995607	3.316208371	intergenic	GPC6;DCT	dist=15132;dist=13660				1		
3M	rs142928734	13	100948828	G	A	421	0.00356295	3	1.242180957	0.270237412	4.59662838	4.29E-06	17.00959296	3.700449886	ncRNA_intronic	NALCN-AS1	.				1		
3M	rs556680896	13	100950161	C	T	421	0.00356295	3	1.24171677	0.270060937	4.597913279	4.27E-06	17.0254659	3.702867989	ncRNA_intronic	NALCN-AS1	.						
12M	rs117245766	13	105804682	A	G	439	0.00341686	3	1.590303394	0.330622791	4.8100235	1.51E-06	14.54837244	3.024594879	intergenic	LINC00343;LINC00460	dist=42886;dist=571881			1			
12M	rs139206944	13	105814677	C	T	439	0.00341686	3	1.855091687	0.369681378	5.018082589	5.22E-07	13.57407456	2.705032115	intergenic	LINC00343;LINC00460	dist=52881;dist=561886						
12M	rs561254473	13	105826900	A	G	439	0.00341686	3	1.848475898	0.368215591	5.020091341	5.16E-07	13.63356541	2.715800268	intergenic	LINC00343;LINC00460	dist=65104;dist=549663						
3M	rs184265355	13	107359004	A	C	421	0.00593824	6	1.694033349	0.342665495	4.94369399	7.67E-07	14.42717187	2.918297916	intronic	FAM155A	.				1		
3M	rs572961122	13	107360637	C	T	421	0.00593824	6	1.632302386	0.349861236	4.665570858	3.08E-06	13.33548956	2.858276075	intronic	FAM155A	.						
3M	rs528809914	13	112386942	G	A	421	0.00356295	3	1.373302637	0.265345521	5.17552597	2.27E-07	19.50485519	3.768671108	intronic	SPACA7	.				1		
3M	rs180765647	13	113724338	G	T	421	0.0023753	3	1.05190176	0.228624832	4.600995217	4.20E-06	20.12465213	4.373978059	intronic	GRK1	.				1		
3M	rs138215817	14	22173619	A	G	421	0.00475059	4	1.521988034	0.305467544	4.982486896	6.28E-07	16.31101896	3.273670217	intergenic	OR4E1;LOC105370401	dist=502284;dist=206292				1		
3M	rs74704551	14	29692681	C	T	421	0.00356295	3	1.302308212	0.231983741	5.613790894	1.98E-08	24.19907048	4.31064693	intronic	PRKD1	.		1				
3M	rs1686289	14	45791779	G	A	421	0.67814727	277	-9.537609426	2.056514006	-4.637755637	3.52E-06	-2.255153927	0.486259757	intergenic	LINC02303;LINC00871	dist=76177;dist=272380				1		
3M	rs176783	14	45811710	A	G	421	0.32185273	267	9.35224324	2.025260115	4.617798558	3.88E-06	2.280101466	0.493763736	intergenic	LINC02303;LINC00871	dist=96108;dist=252449						
3M	rs176786	14	45813767	T	C	421	0.32304038	268	9.449422953	2.033680348	4.64646421	3.38E-06	2.284756409	0.49171936	intergenic	LINC02303;LINC00871	dist=98165;dist=250392						
3M	rs428110	14	45825457	A	C	421	0.31710214	265	9.275749143	2.014875505	4.603633881	4.15E-06	2.284822992	0.49630858	intergenic	LINC02303;LINC00871	dist=109855;dist=238702						
3M	rs116862847	14	63674959	C	T	421	0.00712589	7	1.757642639	0.338759972	5.188460225	2.12E-07	15.3160369	2.951942625	intergenic	WDR89;SGPP1	dist=33088;dist=9258				1		
3M	rs569916471	14	75424639	G	A	421	0.00475059	5	1.390580812	0.298176949	4.663609368	3.11E-06	15.64040878	3.353713303	intergenic	LINC01220;JDP2	dist=128231;dist=3085				1		
3M	rs113767990	14	81251219	G	A	421	0.00475059	4	1.429622227	0.313123218	4.565685793	4.98E-06	14.58111547	3.193630952	intergenic	LOC101928504;STON2	dist=27839;dist=9431				1		
12M	rs192078346	14	103732352	T	C	439	0.00455581	4	1.644493869	0.360149192	4.566146218	4.97E-06	12.67848527	2.776627086	intronic	ZFYVE21	.			1			
3M	rs190251199	14	105124240	T	C	421	0.00356295	3	1.240668633	0.248987137	4.982862353	6.27E-07	20.01252919	4.016271726	intergenic	LINC02298;JAG2	dist=24744;dist=16758				1		
3M	rs185155853	15	40951902	C	T	421	0.00475059	5	1.370089738	0.293393972	4.669795111	3.02E-06	15.91646574	3.408386314	intergenic	DLL4;CHAC1	dist=12829;dist=1569				1		
3M	rs144026361	15	40956471	C	T	421	0.00475059	5	1.365790883	0.290922074	4.694696631	2.67E-06	16.13729945	3.437346588	UTR3	CHAC1	NM_001142776:c.*6970>0;NM_0[illegible].				1		
3M	rs558614420	15	41518672	C	T	421	0.00593824	6	1.524477016	0.330280733	4.615700711	3.92E-06	13.97508315	3.02772732	intronic	RPAP1	.				1		
3M	rs138109686	15	41759244	A	G	421	0.00593824	6	1.543778108	0.336970687	4.581342434	4.62E-06	13.59567051	2.96761718	intronic	MGA	.				1		
3M	rs145896760	15	41827024	G	A	421	0.00593824	6	1.564221999	0.342059488	4.572953105	4.81E-06	13.36888249	2.923468092	UTR3	MAPKBP1	NM_014994:c.*15880>0;NM_001[illegible].				1		
3M	rs140642138	15	41832967	G	A	421	0.00593824	6	1.547446255	0.337525343	4.584681679	4.55E-06	13.58322205	2.962740492	intronic	JMJD7;JMJD7-PLA2G4B	.				1		
3M	rs6080	15	58545734	C	A	421	0.04394299	36	3.688174426	0.80689065	4.570847892	4.86E-06	5.664767454	1.239325304	intronic	LIPC	.				1		
3M	rs145439370	15	58587566	T	C	421	0.03087886	26	3.500007735	0.712559708	4.911879937	9.02E-07	6.893288912	1.403391166	intergenic	LIPC;ADAM10	dist=17722;dist=1243						
3M	rs149425014	15	58659461	T	C	421	0.02612827	22	3.013015463	0.631629727	4.770224288	1.84E-06	7.552247919	1.583206043	intronic	ADAM10	.						
3M	rs146442492	15	58689916	C	T	421	0.02731591	24	3.06908876	0.65936392	4.654620409	3.25E-06	7.059258577	1.516613162	intronic	ADAM10	.						
3M	rs193253461	15	58937154	A	G	421	0.01306413	12	2.449615619	0.482149005	5.080619466	3.76E-07	10.53744676	2.074047629	intergenic	SLTM;RNF111	dist=3475;dist=50509				1		
3M	rs184117160	15	59112107	C	T	421	0.01425178	11	2.271648443	0.468677492	4.846933088	1.25E-06	10.34172362	2.133663377	intronic	CCNB2	.				1		
3M	rs80292573	15	59142887	T	G	421	0.03444181	30	3.721656224	0.753598905	4.938510655	7.87E-07	6.553234912	1.326965834	intronic	MYO1E	.				1		
3M	rs182303755	15	59342593	A	C	421	0.01306413	12	2.406496876	0.477647778	5.038224785	4.70E-07	10.54799166	2.093592905	intronic	MYO1E	.						
3M	rs138217865	15	93849653	C	T	421	0.00475059	4	1.402299902	0.28969226	4.840653664	1.29E-06	16.70964097	3.451938959	intergenic	LOC105370980;LINC02207	dist=641605;dist=6907				1		
12M	rs573087160	16	5796693	G	T	439	0.0022779	3	1.508610705	0.330012697	4.571371704	4.85E-06	13.85210855	3.030186441	intergenic	MIR8065;RBFOX1	dist=164127;dist=222331	1					
12M	rs80212581	16	6362966	C	T	439	0.00341686	3	1.753654591	0.363868409	4.819474705	1.44E-06	13.24510343	2.74824628	intronic	RBFOX1	.						
12M	rs140276610	16	6383734	C	T	439	0.00341686	4	1.668191344	0.35107035	4.751729512	2.02E-06	13.53497813	2.848431943	intronic	RBFOX1	.						
12M	rs113109648	16	6574271	T	C	439	0.0022779	3	1.641370009	0.319940791	5.130230515	2.89E-07	16.03493727	3.125578319	intronic	RBFOX1	.						
12M	rs140545577	16	6805089	C	T	439	0.00341686	3	1.756164801	0.376771481	4.6610874	3.15E-06	12.37112584	2.6541287	intronic	RBFOX1	.						
12M	rs138164904	16	6808238	C	T	439	0.00341686	3	2.188468482	0.375620207	5.826279955	5.67E-09	15.51109298	2.662263589	intronic	RBFOX1	.						
12M	rs533356672	16	7052822	A	G	439	0.00341686	3	1.552617484	0.337713192	4.597443984	4.28E-06	13.61345689	2.961092499	intronic	RBFOX1	.						
12M	rs72779724	16	10323552	C	T	439	0.08542141	74	8.555089753	1.872733081	4.568237642	4.92E-06	2.439342632	0.533978927	intergenic	GRIN2A;ATF7IP2	dist=140644;dist=62509			1			
3M	rs553840536	16	25686574	A	G	421	0.00356295	3	1.093708141	0.236100775	4.632378455	3.61E-06	19.62034417	4.23547954	intergenic	ZKSCAN2;HS3ST4	dist=428729;dist=5385				1		
12M	rs180797135	16	58970512	G	A	439	0.00341686	3	1.571216829	0.339826153	4.623590074	3.77E-06	13.60575114	2.942681104	intergenic	GOT2;APOOP5	dist=236196;dist=783629			1	1		1
12M	rs139174841	16	59022371	G	A	439	0.00911162	8	2.600047823	0.565206697	4.600171647	4.22E-06	8.138919215	1.769264245	intergenic	GOT2;APOOP5	dist=288055;dist=731770						
3M	rs183817723	16	59268871	C	T	421	0.00356295	4	1.261174136	0.267795557	4.709466243	2.48E-06	17.5860507	3.734191901	intergenic	GOT2;APOOP5	dist=534555;dist=485270						
12M	rs572469018	16	72297050	C	T	439	0.00341686	3	1.768848944	0.374808516	4.719340325	2.37E-06	12.59133697	2.668029026	ncRNA_intronic	LINC01572	.			1			
12M	rs537465910	16	72326220	G	A	439	0.00341686	3	1.764616188	0.374136714	4.716501002	2.40E-06	12.60635704	2.672819752	ncRNA_intronic	LINC01572	.						
12M	rs543741501	16	72351658	T	C	439	0.00341686	3	1.762934021	0.374136356	4.712009391	2.45E-06	12.59436385	2.672822315	ncRNA_intronic	LINC01572	.						
12M	rs149214138	16	72417776	T	C	439	0.00341686	3	1.753884158	0.372559459	4.707662398	2.51E-06	12.63600287	2.68413531	ncRNA_intronic	LINC01572	.						
12M	rs550597094	16	72420594	G	A	439	0.00341686	3	1.75871144	0.372991675	4.715149313	2.42E-06	12.64143312	2.681024986	ncRNA_intronic	LINC01572	.						
12M	rs537719803	16	72564328	C	A	439	0.00341686	3	1.724217925	0.368081111	4.68434232	2.81E-06	12.72638607	2.716792496	ncRNA_intronic	LINC01572	.						
12M	rs148996503	16	72564549	G	A	439	0.00341686	3	1.724389568	0.368079708	4.684826489	2.80E-06	12.72774994	2.716802848	ncRNA_intronic	LINC01572	.						
12M	rs530059247	16	72820010	C	T	439	0.00341686	3	1.656813634	0.357508509	4.634333424	3.58E-06	12.96286187	2.797136219	intronic	ZFHX3	.						
3M	rs144954214	16	76145464	A	G	421	0.0023753	3	1.106696587	0.225254382	4.913096823	8.96E-07	21.81132631	4.439425294	intergenic	CPHXL;CNTNAP4	dist=418974;dist=131937				1		
3M	rs529523094	16	77681654	A	G	421	0.00356295	3	1.307832421	0.26837178	4.873211417	1.10E-06	18.15843462	3.726174193	intergenic	ADAMTS18;NUDT7	dist=246620;dist=40838				1		
3M	rs146728064	17	19362127	G	A	421	0.00712589	6	1.69020067	0.365674681	4.622143005	3.80E-06	12.64004111	2.734671147	intronic	B9D1	.				1		
12M	rs72825069	17	34273909	C	T	439	0.02164009	20	4.470064227	0.904834992	4.940198229	7.80E-07	5.459778049	1.105173881	intergenic	CCL7;CCL11	dist=1667;dist=11833			1			
12M	rs77158902	17	34365017	C	T	439	0.02277904	20	4.491884806	0.953623181	4.710335166	2.47E-06	4.939409255	1.048632227	intergenic	CCL1;C17orf102	dist=1784;dist=209106						
12M	rs188353596	17	48001236	C	T	439	0.00341686	3	1.927521209	0.371158042	5.193262681	2.07E-07	13.99205215	2.694270059	intergenic	CDK5RAP3;COPZ2	dist=19450;dist=24931			1			
3M	rs184613584	17	50430860	A	C	421	0.00712589	6	1.77626107	0.354456155	5.011229298	5.41E-07	14.13779737	2.821223402	intronic	ACSF2	.				1		
3M	rs191271637	17	54045899	A	G	421	0.00356295	3	1.195597009	0.255047807	4.687736869	2.76E-06	18.379836	3.920833552	intergenic	KIF2B;TOM1L1	dist=220706;dist=854792				1		
12M	rs147669485	18	29023686	G	T	439	0.00341686	4	1.924640141	0.391979738	4.910050065	9.11E-07	12.52628537	2.551152271	intergenic	CDH2;MIR302F	dist=846556;dist=1275226			1	1		1
3M	rs185819304	18	29421615	G	A	421	0.00356295	3	1.281236388	0.25348417	5.05450257	4.32E-07	19.94011134	3.945019527	intergenic	CDH2;MIR302F	dist=1244486;dist=877296						

3M	rs187942235	18	29450465	C	T	421	0.00356295	3	1.294384565	0.251912631	5.138228126	2.77E-07	20.39686581	3.969630251	intergenic	CDH2;MIR302F	dist=1273336;dist=848446						
3M	rs139493286	18	31236056	G	A	421	0.00356295	3	1.241116041	0.266612329	4.655133716	3.24E-06	17.46030927	3.750764282	intergenic	DSC1;DSG1	dist=73200;dist=82104				1		
3M	rs143538552	18	31470299	A	G	421	0.00356295	3	1.44755014	0.274967228	5.264446053	1.41E-07	19.14572179	3.636797033	intronic	DSG3	.						
3M	rs373746073	18	31478421	C	A	421	0.00356295	3	1.441999195	0.274877768	5.245965155	1.55E-07	19.08471968	3.637980642	UTR3	DSG3	NM_001944:c.*21610>0						
3M	rs146333745	18	57830225	C	T	421	0.00356295	4	1.368613476	0.280282935	4.882971117	1.04E-06	17.42157837	3.567823349	intergenic	ATP8B1;NEDD4L	dist=26910;dist=214001				1		
12M	rs192064655	18	63290467	C	T	439	0.0022779	3	1.627195325	0.305228607	5.331070832	9.76E-08	17.46582956	3.276232883	intronic	BCL2	.			1			
12M	rs559152067	18	68362375	G	A	439	0.0022779	3	1.720252761	0.315267464	5.456486816	4.86E-08	17.30748472	3.17190993	intergenic	LOC643542;TMX3	dist=462756;dist=311313	1					
3M	rs185464792	19	18686561	C	T	421	0.0023753	3	1.114587174	0.222127708	5.017776414	5.23E-07	22.58960154	4.501914727	intronic	CRTC1	.				1		
3M	rs186768950	19	18695314	C	A	421	0.0023753	3	1.117877431	0.221839436	5.039128522	4.68E-07	22.71520611	4.507764788	intronic	CRTC1	.						
3M	rs541288561	19	18758635	T	G	421	0.00475059	5	1.591688559	0.31930164	4.984905675	6.20E-07	15.61190124	3.131834835	intronic	CRTC1	.						
3M	rs559008174	19	18765249	C	T	421	0.00475059	5	1.594517191	0.319680825	4.987841211	6.11E-07	15.60256615	3.12812006	intronic	CRTC1	.						
3M	rs570407448	19	18769220	G	A	421	0.00475059	5	1.587863075	0.320089569	4.960683605	7.02E-07	15.4977984	3.124125552	intronic	CRTC1	.						
3M	rs546144116	19	19452530	C	T	421	0.0023753	3	1.157037115	0.22363222	5.173839072	2.29E-07	23.13548147	4.471627577	intronic	GATAD2A	.				1		
3M	rs560206697	19	20546292	C	T	421	0.0023753	3	1.205675879	0.219610964	5.490053226	4.02E-08	24.99899431	4.553506728	intronic	ZNF737	.		1				
3M	rs111285015	19	22940396	G	A	421	0.00356295	3	1.280187265	0.216410877	5.915540303	3.31E-09	27.33476425	4.62083983	intergenic	ZNF723;ZNF728	dist=81729;dist=34487		1				
3M	rs1008091735	19	30599192	T	C	421	0.00356295	3	1.355816116	0.270944036	5.004044884	5.61E-07	18.46892428	3.690799086	intronic	ZNF536	.				1		
3M	rs148433854	19	30605571	G	A	421	0.00356295	3	1.369429098	0.271144709	5.050547011	4.41E-07	18.62675851	3.688067545	intronic	ZNF536	.						
12M	rs181104894	19	34621605	A	C	439	0.00341686	3	1.675710965	0.35551557	4.713467166	2.44E-06	13.25811742	2.81281633	ncRNA_intronic	SCGB1B2P	.			1			
12M	rs367732718	19	35427102	G	A	439	0.0022779	3	1.635791474	0.317523551	5.151717003	2.58E-07	16.22467686	3.149372695	intergenic	LINC01531;FFAR2	dist=10262;dist=21155			1			
12M	rs547186621	20	6126094	A	G	439	0.00341686	4	1.874227998	0.3576548	5.240326692	1.60E-07	14.65191208	2.795992109	intergenic	FERMT1;CASC20	dist=3064;dist=300638			1			
3M	rs2327968	20	15832846	C	T	421	0.02494062	21	3.410130247	0.744098505	4.582901624	4.59E-06	6.158998564	1.343908089	intronic	MACROD2	.				1		
3M	rs2876414	20	15833059	G	T	421	0.02256532	19	3.090964158	0.673260548	4.591037107	4.41E-06	6.819109065	1.485309072	intronic	MACROD2	.						
3M	rs140788628	20	15877856	C	A	421	0.01068884	8	2.265436379	0.438089306	5.171174799	2.33E-07	11.80392839	2.282639604	intronic	MACROD2	.						
3M	rs559228693	20	15982684	G	A	421	0.00593824	5	1.512274875	0.324468321	4.660778189	3.15E-06	14.36435511	3.081964969	ncRNA_intronic	LOC613266	.						
12M	rs138733283	20	32087884	C	T	439	0.00455581	5	2.081660055	0.444061489	4.687774347	2.76E-06	10.55658837	2.251940385	intronic	HCK	.			1			
12M	rs149859280	20	32090367	C	T	439	0.00455581	5	2.082920752	0.443760413	4.693795782	2.68E-06	10.57731976	2.25346825	intronic	HCK	.						
12M	rs146249289	20	32094428	C	T	439	0.00455581	4	2.061071174	0.430206938	4.790883158	1.66E-06	11.13622941	2.324462744	intronic	HCK	.						
12M	rs145791959	20	32134626	G	A	439	0.00569476	5	2.057551242	0.446297665	4.61026665	4.02E-06	10.33002637	2.240657027	intronic	TM9SF4	.			1			
12M	rs193041547	20	32184077	T	C	439	0.00455581	4	2.07477076	0.430413643	4.820411237	1.43E-06	11.19948524	2.32334643	intergenic	TM9SF4;TSPY26P	dist=16819;dist=5069						
12M	rs138055631	20	32192841	G	A	439	0.00569476	5	2.092550815	0.449042548	4.660027927	3.16E-06	10.37769794	2.226960461	UTR3	PLAGL2	NM_002657:c.*36110>0						
12M	rs145421321	20	32274323	C	T	439	0.00569476	6	2.10438963	0.457025953	4.604529826	4.13E-06	10.07498546	2.18805955	intergenic	POFUT1;KIF3B	dist=35665;dist=3328						
12M	rs143432612	20	32304972	C	T	439	0.00569476	6	2.095354506	0.458885267	4.566183876	4.97E-06	9.950600304	2.179193956	intronic	KIF3B	.						
12M	rs139816293	20	32333540	C	T	439	0.00569476	6	2.161099014	0.461179259	4.6860282	2.79E-06	10.16096909	2.168354235	UTR3	KIF3B	NM_004798:c.*22210>0						
12M	rs200198574	20	32358851	G	A	439	0.00569476	6	2.117861385	0.463869302	4.565642465	4.98E-06	9.842519093	2.155779645	intronic	ASXL1	.			1			
12M	rs148157126	20	32361036	C	T	439	0.00455581	5	2.092134134	0.43147068	4.848844269	1.24E-06	11.23794616	2.317654586	intronic	ASXL1	.						
3M	rs557092705	20	35601989	C	T	421	0.00356295	3	1.079320791	0.235404492	4.584962596	4.54E-06	19.47695457	4.248007298	ncRNA_exonic	FER1L4	.				1		
12M	rs3795102	20	57683075	C	T	439	0.00455581	4	2.19105865	0.424843705	5.157328741	2.50E-07	12.13935544	2.353806795	intronic	PMEPA1	.			1			
3M	rs184785969	21	15799112	C	A	421	0.00356295	3	1.446338922	0.269771603	5.361346058	8.26E-08	19.87364864	3.706839369	intronic	USP25	.		1				
3M	rs117280553	21	15834844	T	C	421	0.00356295	3	1.465128514	0.27006967	5.425002059	5.80E-08	20.08741695	3.702748263	intronic	USP25	.						
3M	rs79486609	21	15872687	G	A	421	0.00356295	3	1.447442576	0.264672828	5.468799292	4.53E-08	20.66248857	3.77824957	intronic	USP25	.						
3M	rs73227413	21	21764653	G	A	421	0.03444181	28	3.687754962	0.78647158	4.688986931	2.75E-06	5.962055143	1.271501762	ncRNA_intronic	LINC01425	.		1				
3M	rs75024143	21	21784226	G	T	421	0.01425178	12	2.293452396	0.483452813	4.743901232	2.10E-06	9.812542411	2.068454196	ncRNA_intronic	LINC01425	.						
3M	rs192134381	21	22078395	T	C	421	0.00356295	3	1.565034889	0.268006444	5.83954202	5.23E-09	21.78881202	3.731253572	ncRNA_intronic	LINC01687	.						
3M	rs118183140	21	34105187	C	T	421	0.02019002	17	3.113677552	0.640523765	4.861142898	1.17E-06	7.589324801	1.561222322	UTR3	SLC5A3	NM_006933:c.*78320>0				1		
3M	rs183586634	21	37390730	G	A	421	0.00593824	5	1.598766374	0.334213375	4.783669629	1.72E-06	14.31322017	2.992100476	intronic	DYRK1A	.				1		
3M	rs117185941	21	37394182	G	A	421	0.00593824	5	1.529223098	0.31531516	4.849824212	1.24E-06	15.38087864	3.171430132	intronic	DYRK1A	.						
3M	rs118084887	21	37491518	T	C	421	0.00593824	5	1.518241868	0.31880157	4.762341255	1.91E-06	14.93826162	3.136747415	intronic	DYRK1A	.						
12M	rs117262205	21	37733520	C	T	439	0.00683371	6	2.582956261	0.529108171	4.881716823	1.05E-06	9.226311531	1.889972701	intronic	KCNJ6	.			1			
3M	rs150539922	21	41856807	T	C	421	0.00356295	3	1.254834519	0.274365448	4.57358799	4.79E-06	16.66969372	3.64477381	intronic	PRDM15	.				1		
12M	rs5994128	22	17010042	A	G	439	0.77448747	220	-11.14584694	2.280785591	-4.886845561	1.02E-06	-2.142615062	0.438445422	intergenic	GAB4;CECR7	dist=1820;dist=26528			1			
3M	rs113625788	22	19981659	C	T	421	0.00831354	7	2.000739147	0.411621625	4.860626906	1.17E-06	11.80848286	2.429415606	exonic	ARVCF	.				1		
3M	rs78547898	22	32428291	G	A	421	0.00356295	3	1.210956763	0.240651719	5.03198883	4.85E-07	20.90983951	4.155382737	intronic	BPIFC	.				1		
12M	rs117360642	22	33435071	G	A	439	0.01366743	13	3.472105912	0.689882552	5.032894224	4.83E-07	7.295291363	1.449522092	intronic	LARGE1	.			1			
12M	rs61546457	22	34073343	T	C	439	0.0501139	44	6.361384106	1.334216593	4.767879623	1.86E-06	3.573542443	0.749503495	intergenic	LARGE1;LINC02885	dist=150520;dist=683324						
12M	rs35373244	22	34073359	G	A	439	0.0501139	44	6.361383523	1.334255598	4.767739805	1.86E-06	3.573333185	0.749481585	intergenic	LARGE1;LINC02885	dist=150536;dist=683308						
12M	rs35482368	22	34073458	T	C	439	0.0501139	44	6.407425495	1.347517024	4.754986676	1.98E-06	3.528702488	0.742105652	intergenic	LARGE1;LINC02885	dist=150635;dist=683209						
12M	rs5994839	22	34088192	C	A	439	0.03986333	35	5.549459281	1.192274826	4.654513506	3.25E-06	3.903893133	0.838732797	intergenic	LARGE1;LINC02885	dist=165369;dist=668475						
3M	rs541680196	22	40132086	G	A	421	0.00593824	5	1.654258232	0.339093792	4.878468054	1.07E-06	14.38678079	2.949036589	intronic	TNRC6B	.				1		
3M	rs185139807	22	40198777	G	A	421	0.00593824	5	1.65498472	0.338624126	4.887379825	1.02E-06	14.43305263	2.953126858	intronic	TNRC6B	.						
3M	rs141127122	22	40208435	G	A	421	0.00475059	4	1.411818313	0.302953602	4.660179993	3.16E-06	15.38248751	3.30083549	intronic	TNRC6B	.						
3M	rs148998974	22	40224526	A	G	421	0.00593824	5	1.667947442	0.339010604	4.920045046	8.65E-07	14.51295325	2.949760239	intronic	TNRC6B	.						
3M	rs555040883	22	40235472	G	A	421	0.00475059	4	1.412000774	0.302532759	4.667265721	3.05E-06	15.42730692	3.305427169	intronic	TNRC6B	.						
3M	rs182959028	22	45427152	T	C	421	0.00831354	7	1.863907765	0.395028955	4.718407959	2.38E-06	11.94446103	2.531460004	intronic	RIBC2	.				1		
3M	rs150946694	22	46457283	T	C	421	0.00475059	4	1.413199859	0.303835874	4.651194872	3.30E-06	15.30824787	3.291250592	intronic	CELSR1	.				1		
																		6	24	99	208	2	12

total 12 month loci: 105
total 3 month loci: 232

Supplemental Table S4. GWAS Results Risk Loci Indianapolis-1 Discovery Cohort
Overlap of 12 month (12M) and 3 month (3M) quantitative trait risk loci

Notes
Top SNPs identified with the 12M and 3M QTs are merged, then clustered into risk loci by sorting by chromosomal position, then by chromosome
Each risk locus clustering SNPs from both the 12 and 3 month QTs is boxed across the row and the Gene.refGene column entries are boxed
For risk loci clustering multiple SNPs, some of which are of genome-wide significance, the Gene.refGene column entries are boxed, the Score.pval column entries are boxed, and the boxes are shaded in gray
Standard Headers
rsID: reference SNP cluster ID, chr_38: chromosome number; pos_38, position of SNP on GRCh38 reference panel; Imputation_Rsq; estimate of the squared correlation between imputed and true genotypes; REF and ALT, reference allele and alternate allele; n.obs: number of observations;
caf: coding allele frequency; MAC: minor allele count; Score: p-values from Score test; Score.SE: standard error of the score statistic; Score.Stat: computed score statistic based on the derivative of the log-likelihood function; Score.pval: probability value associated with the Score.Stat;
EST: estimated effect size; EST.SE: standard error of the effect size; Func.refGene: SNP location with respect to nearest gene; Gene.refGene: closet gene(s) upstream and downstream; GeneDetail.refGene: distance of SNP to nearest gene(s)
Added Headers, Abbreviations and Acronyms
QT: quantitative trait; 3M: 3 month; 12M: 12 month
Count
5E-08 rep loci: includes SNP(s) of genome-wide significance repeated with the 2 QTs; 5E-08 diff loci: includes SNP(s) of genome-wide significance different with the 2 QTs; 5E-08 mixed: includes SNP(s) of genome-wide significance repeated and different with the 2 QTs
5E-06 rep loci: includes SNP(s) of suggestive significance repeated with the 2 QTs; 5E-06 diff loci: includes SNP(s) of suggestive significance different with the 2 QTs; 5E-08 mixed: includes SNP(s) of suggestive significance repeated and different with the 2 QTs
5E-08 rep SNPs: repeated genome-wide signficance SNP count; 5E-06 rep SNPs: repeated suggestive significance SNP count

																Count							
QT	rsid	chr	pos_38	REF	ALT	n.obs	caf	MAC	Score	Score.SE	Score.Stat	Score.pval	Est	Est.SE	Func.refGene	Gene.refGene	GeneDetail.refGene	5E-08 rep loci	5E-08 diff loci	5E-08 mixed loci	5E-06 rep loc	5E-06 diff loci	5E-06 mixed loci	5E-08 rep SNPs	5E-06 rep SNPs
3M	rs184098071	2	176251692	G	A	421	0.003562945	3	1.695892029	0.34869637	4.86352074	1.15E-06	13.94772404	2.86782452	intergenic	HOXD1;MTX2	dist=60785;dist=17750					1			
12M	rs192500829	2	176385923	A	G	439	0.004555809	3	1.674098367	0.340713541	4.91350699	8.95E-07	14.42122604	2.935016895	intergenic	MTX2;MIR1246	dist=47898;dist=215057								
3M	rs532416695	2	176622062	G	A	421	0.003562945	3	1.400161077	0.265436681	5.2749344	1.33E-07	19.87266561	3.767376828	intergenic	MIR1246;LINC01116	dist=21010;dist=7519								
3M	rs181217257	2	239067423	C	T	421	0.004750594	4	1.788144205	0.287937999	6.21017097	5.29E-10	21.56773677	3.472969885	intronic	HDAC4	.		1						
3M	rs188076929	2	239072023	T	C	421	0.004750594	4	1.865485168	0.305579814	6.10473952	1.03E-09	19.97756143	3.272467463	intronic	HDAC4	.								
12M	rs115690445	2	239682619	A	C	439	0.007972665	7	2.51110667	0.515854898	4.86785466	1.13E-06	9.436480453	1.938529621	intergenic	HDAC4-AS1;LOC150935	dist=280255;dist=80241								
12M	rs148248743	3	136415753	C	T	439	0.002277904	3	1.586456973	0.298755282	5.31022235	1.09E-07	17.7744886	3.347221159	intronic	STAG1	.				1				1
3M	rs148248743	3	136415753	C	T	421	0.002375297	3	1.069833829	0.216429281	4.94311039	7.69E-07	22.83937904	4.620446891	intronic	STAG1	.								
3M	rs187047882	3	164567833	G	A	421	0.003562945	3	1.161853087	0.250171732	4.6442221	3.41E-06	18.56413613	3.997254169	intergenic	MIR1263;LINC01324	dist=396277;dist=146262					1			
12M	rs139635002	3	164614597	T	C	439	0.007972665	8	2.540439901	0.548123379	4.63479574	3.57E-06	8.4557527	1.824406765	intergenic	MIR1263;LINC01324	dist=443041;dist=99498								
3M	rs141169929	3	165090674	A	G	421	0.004750594	5	1.473704308	0.315071386	4.67736638	2.91E-06	14.84541783	3.173883899	intergenic	SI;SLITRK3	dist=12178;dist=96046								
12M	rs754653672	3	165112751	A	G	439	0.003416856	3	1.695149727	0.37024662	4.57843404	4.68E-06	12.36590367	2.700902442	intergenic	SI;SLITRK3	dist=34255;dist=73969								
12M	rs994788245	3	165182662	G	T	439	0.003416856	3	1.457659335	0.317040616	4.59770534	4.27E-06	14.50194425	3.154169999	intergenic	SI;SLITRK3	dist=104166;dist=4058								
12M	rs189709453	3	177856520	G	A	439	0.003416856	3	1.623833795	0.337340968	4.81362761	1.48E-06	14.26932412	2.964359789	ncRNA_intronic	LINC02015	.				1				1
3M	rs189709453	3	177856520	G	A	421	0.003562945	3	1.314463975	0.246356303	5.33562145	9.52E-08	21.65814876	4.05916142	ncRNA_intronic	LINC02015	.								
12M	rs186767531	3	177921989	T	C	439	0.003416856	3	1.610325603	0.338183106	4.76169736	1.92E-06	14.08023428	2.956977988	intergenic	LINC02015;LINC01014	dist=22765;dist=497212								1
3M	rs186767531	3	177921989	T	C	421	0.003562945	3	1.293967205	0.247047316	5.23773026	1.63E-07	21.20132426	4.047807581	intergenic	LINC02015;LINC01014	dist=22765;dist=497212								
12M	rs182868205	3	177994660	C	T	439	0.003416856	3	1.649581368	0.344172256	4.79289466	1.64E-06	13.92586001	2.90552182	intergenic	LINC02015;LINC01014	dist=95436;dist=424541								1
3M	rs182868205	3	177994660	C	T	421	0.003562945	3	1.323927501	0.251356167	5.26713752	1.39E-07	20.95487681	3.978418395	intergenic	LINC02015;LINC01014	dist=95436;dist=424541								
3M	rs145875128	4	32071514	G	A	421	0.003562945	3	1.153690598	0.217089638	5.3143513	1.07E-07	24.47998599	4.606392132	ncRNA_intronic	LINC02506	.					1			
12M	rs146847867	4	32390284	G	T	439	0.004555809	4	2.185302341	0.426711618	5.12126281	3.03E-07	12.00169528	2.3435031	intergenic	LINC02353;LOC101928622	dist=37064;dist=1506055								
12M	rs183086778	4	32609199	A	G	439	0.004555809	5	2.346647932	0.430540407	5.45047084	5.02E-08	12.6596035	2.322662367	intergenic	LINC02353;LOC101928622	dist=255979;dist=1287140								
12M	rs144899985	4	32738728	A	G	439	0.004555809	5	2.245036023	0.430094163	5.21987094	1.79E-07	12.13657701	2.32507224	intergenic	LINC02353;LOC101928622	dist=385508;dist=1157611								
12M	rs192772770	4	32808172	T	G	439	0.005694761	5	2.334772732	0.461927473	5.0544141	4.32E-07	10.94200798	2.164842011	intergenic	LINC02353;LOC101928622	dist=454952;dist=1088167								
12M	rs76887296	4	32865383	C	T	439	0.005694761	5	2.374205059	0.477289182	4.97435338	6.55E-07	10.42209538	2.09516586	intergenic	LINC02353;LOC101928622	dist=512163;dist=1030956								
3M	rs143287889	4	35570658	C	T	421	0.002375297	3	1.058108262	0.230199399	4.59648578	4.30E-06	19.96741	4.34406	intergenic	LINC02484;ARAP2	dist=1300911;dist=495346								
12M	rs191122698	4	60001148	T	C	439	0.006833713	6	2.484118908	0.503688379	4.9318567	8.15E-07	9.791483996	1.98535452	intergenic	LINC02429;MIR548AG1	dist=954189;dist=921471					1			
12M	rs181164671	4	60202387	G	T	439	0.006833713	6	2.365357436	0.473829215	4.9920042	5.98E-07	10.53545041	2.110465052	intergenic	LINC02429;MIR548AG1	dist=1155428;dist=720232								
3M	rs999769259	4	61646247	G	A	421	0.003562945	3	0.969956079	0.209381164	4.63248968	3.61E-06	22.12467252	4.775978806	intronic	ADGRL3	.								
12M	rs115901263	4	86154667	A	G	439	0.005694761	6	2.238202026	0.474593389	4.71604131	2.40E-06	9.93701433	2.107066856	ncRNA_intronic	MAPK10-AS1	.					1			
3M	rs147630370	4	86529522	T	C	421	0.004750594	4	1.530518883	0.309272178	4.94877648	7.47E-07	16.00136328	3.23339786	intergenic	MAPK10;MIR4452	dist=76327;dist=12960								
3M	rs116651654	4	162317591	C	T	421	0.007125891	6	1.661626207	0.326131851	5.09495224	3.49E-07	15.62236937	3.066244515	intergenic	FSTL5;MIR4454	dist=153557;dist=775983					1			
12M	rs114259958	4	162398591	A	G	439	0.003416856	3	1.650939137	0.357816014	4.61393307	3.95E-06	12.89470814	2.794732381	intergenic	FSTL5;MIR4454	dist=234557;dist=694983								
12M	rs12350891	9	79989706	G	A	439	0.004555809	4	1.919901525	0.416077273	4.61429078	3.94E-06	11.08998515	2.403399716	ncRNA_intronic	LINC01507	.					1			
3M	rs148556485	9	81408911	A	C	421	0.002375297	3	1.17470554	0.234401563	5.01150899	5.40E-07	21.38001525	4.266183157	intergenic	LINC01507;TLE1	dist=1374356;dist=174772								
3M	rs140782222	9	81413979	T	C	421	0.002375297	3	1.1680569	0.231519821	5.04517019	4.53E-07	21.79152597	4.31928461	intergenic	LINC01507;TLE1	dist=1379424;dist=169704								
3M	rs77871739	9	135660463	G	A	421	0.004750594	4	1.439297375	0.309017583	4.65765527	3.20E-06	15.07246035	3.236061811	intergenic	GLT6D1;LCN9	dist=20923;dist=2859					1			
12M	rs76294395	9	135780517	G	A	439	0.005694761	5	2.250462353	0.487505863	4.61627752	3.91E-06	9.469173331	2.051257382	intronic	KCNT1	.								
12M	rs566724618	13	22889700	C	T	439	0.003416856	3	1.92789865	0.356546436	5.40714604	6.40E-08	15.16533472	2.804683764	ncRNA_intronic	LINC00621	.		1			1			
12M	rs147221953	13	22911583	G	A	439	0.003416856	3	1.853658494	0.344939547	5.37386482	7.71E-08	15.57914966	2.899058719	ncRNA_intronic	LINC00621	.								
3M	rs139598422	13	23312875	A	G	421	0.003562945	4	1.544940466	0.274599144	5.62616635	1.84E-08	20.48865215	3.641671944	intronic	SGCG	.								
12M	rs180797135	16	58970512	G	A	439	0.003416856	3	1.571216829	0.339826153	4.62359007	3.77E-06	13.60575114	2.942681104	intergenic	GOT2;APOOP5	dist=236196;dist=783629					1			
12M	rs139174841	16	59022371	G	A	439	0.009111617	8	2.600047823	0.565206697	4.60017165	4.22E-06	8.138919215	1.769264245	intergenic	GOT2;APOOP5	dist=288055;dist=731770								
3M	rs183817723	16	59268871	C	T	421	0.003562945	4	1.261174136	0.267795557	4.70946624	2.48E-06	17.5860507	3.734191901	intergenic	GOT2;APOOP5	dist=534555;dist=485270								
12M	rs147669485	18	29023686	G	T	439	0.003416856	4	1.924640141	0.391979738	4.91005007	9.11E-07	12.52628537	2.551152271	intergenic	CDH2;MIR302F	dist=846556;dist=1275226					1			
3M	rs185819304	18	29421615	G	A	421	0.003562945	3	1.281236388	0.25348417	5.05450257	4.32E-07	19.94011134	3.945019527	intergenic	CDH2;MIR302F	dist=1244486;dist=877296								
3M	rs187942235	18	29450465	C	T	421	0.003562945	3	1.294384565	0.251912631	5.13822813	2.77E-07	20.39686581	3.969630251	intergenic	CDH2;MIR302F	dist=1273336;dist=848446								
																Totals:		0	2	0	2	11	0	0	4

Supplemental Table S5. Target Gene Prioritization
SNP-associated genes that are aqueous outflow pathway (AOP)-expressed

Notes
Top SNPs identified with the 12M and 3M QTs are merged, then clustered into risk loci by sorting by chromosomal position, then by chromosome
For risk loci clustering multiple SNPs, the Gene.refGene column entries are boxed
For risk loci clustering multiple SNPs, some of which are of genome-wide significance, the Gene.refGene column entries are boxed, the Score.pval column entries are boxed, and the boxes are shaded in gray
Risk loci clustering SNPs from both the 12 and 3 month QTs are boxed across the row and highlighted in blue
Standard Headers
rsID: reference SNP cluster ID, chr_38: chromosome number; pos_38, position of SNP on GRCh38 reference panel; Imputation_Rsq; estimate of the squared correlation between imputed and true genotypes; REF and ALT, reference allele and alternate allele; n.obs: number of observations;
caf: coding allele frequency; MAC: minor allele count; Score: p-values from Score test; Score.SE: standard error of the score statistic; Score.Stat: computed score statistic based on the derivative of the log-likelihood function; Score.pval: probability value associated with the Score.Stat;
EST: estimated effect size; EST.SE: standard error of the effect size; Func.refGene: SNP location with respect to nearest gene; Gene.refGene: closet gene(s) upstream and downstream; GeneDetail.refGene: distance of SNP to nearest gene(s)
Special Headers
QT: quantitative trait; Gene.refGene AOP-expressed: of the Gene.refGene genes listed, these are the ones that are AOP-expressed
up;downstream AOP-expressed: if none of the Gene.refGene genes were AOP expressed, looked further upstream and downstream of the identified SNP for the next closest gene(s) that are AOP-expressed
Abbreviations and Acronyms
3 month; 12M: 12 month

QT	rsid	chr	pos_38	REF	ALT	n.obs	caf	MAC	Score	Score.SE	Score.Stat	Score.pval	Est	Est.SE	Func.refGene	Gene.refGene	GeneDetail.refGene	Gene.refGene AOP-expressed	up;downstream AOP-expressed
12M	rs115658028	1	7627617	G	A	439	0.00683371	7	2.313647214	0.504805248	4.583247148	4.58E-06	9.07923831	1.980961972	intronic	CAMTA1	.	CAMTA1	
3M	rs115348382	1	9595845	G	A	421	0.00356295	3	1.230366386	0.259928152	4.733486451	2.21E-06	18.21074945	3.847216981	intronic	TMEM201	.	TMEM201	
3M	rs186532456	1	18621834	C	T	421	0.00356295	3	1.189953152	0.242873045	4.899486277	9.61E-07	20.17303433	4.117377453	intergenic	KLHDC7A;PAX7	dist=135848;dist=9012	KLHDC7A	
3M	rs562032622	1	18632839	A	C	421	0.00356295	3	1.126818759	0.231494862	4.867575676	1.13E-06	21.0267115	4.319750303	intronic	PAX7	.		TM2D1;L1TD1
3M	rs2365739	1	62018790	G	A	421	0.02137767	18	3.317349453	0.694985007	4.773267649	1.81E-06	6.868159166	1.43887996	intronic	PATJ	.	LEPR	
3M	rs149493615	1	79414404	G	A	421	0.00831354	8	1.98260972	0.410759865	4.826688019	1.39E-06	11.750632	2.434512435	intergenic	ADGRL4;LINC01781	dist=407674;dist=1121351	ADGRL4	
3M	rs143811231	1	79502446	T	C	421	0.00831354	8	1.975347907	0.413110429	4.78164619	1.74E-06	11.57474094	2.420660266	intergenic	ADGRL4;LINC01781	dist=495716;dist=1033309		
3M	rs34270375	1	88905019	G	A	421	0.02612827	21	3.119890277	0.63606437	4.904991419	9.34E-07	7.711470175	1.572167924	intergenic	GTF2B;KYAT3	dist=13452;dist=30754	GTF2B	
3M	rs187518659	1	98990189	T	G	421	0.00950119	8	1.921562765	0.405573328	4.737892339	2.16E-06	11.68196232	2.465645372	intronic	PLPPR5	.	PLPPR5	
3M	rs140420703	1	102337928	T	G	421	0.00475059	5	1.375529427	0.288838786	1.375529427	1.91E-06	16.48765399	3.462138915	intergenic	OLFM3;COL11A1	dist=340694;dist=538539	OLFM3;COL11A1	
3M	rs563167766	1	102400809	G	A	421	0.0023753	3	1.07877255	0.235715413	1.07877255	4.73E-06	19.41573898	4.242403955	intergenic	OLFM3;COL11A1	dist=403575;dist=475658		
3M	rs77180278	1	102496326	T	C	421	0.02850356	24	3.885721783	0.727780259	5.339141503	9.34E-08	7.336199959	1.374041118	intergenic	OLFM3;COL11A1	dist=499092;dist=380141		
3M	rs112351653	1	102754804	T	C	421	0.02850356	24	4.178844629	0.750261996	5.569847131	2.55E-08	7.423869471	1.332867724	intergenic	OLFM3;COL11A1	dist=757570;dist=121663		
3M	rs180926150	1	102760770	C	T	421	0.0023753	3	1.088498575	0.235408416	4.62387281	3.77E-06	19.64191801	4.247936485	intergenic	OLFM3;COL11A1	dist=763536;dist=115697		
3M	rs114413507	1	102953612	T	C	421	0.02850356	24	4.177369718	0.750215949	5.568223023	2.57E-08	7.42216029	1.332949535	intronic	COL11A1	.		
3M	rs116672066	1	103007360	G	A	421	0.02731591	23	4.276024986	0.728927557	5.866186486	4.46E-09	8.047694764	1.37187844	intronic	COL11A1	.		
3M	rs111928960	1	103168079	G	A	421	0.02850356	22	4.045287237	0.68574164	5.899141891	3.65E-09	8.602572083	1.45827516	intergenic	COL11A1;LOC101928436	dist=59557;dist=325967		
3M	rs113221952	1	103288418	A	G	421	0.02137767	19	3.040841012	0.619495467	4.908576695	9.17E-07	7.923507043	1.614216816	intergenic	COL11A1;LOC101928436	dist=179896;dist=205628		
3M	rs1856085	1	103571923	G	A	421	0.0023753	3	1.181208876	0.234330387	5.040784048	4.64E-07	21.51143997	4.267478979	intronic	AMY2B	.	AMY2B;AMY2A	
3M	rs143597860	1	103614521	A	G	421	0.0023753	3	1.179951068	0.233861351	5.045515482	4.52E-07	21.57481541	4.276037896	intergenic	AMY2B;AMY2A	dist=34987;dist=2811		
3M	rs144541665	1	103768107	G	A	421	0.0023753	3	1.181121027	0.232374488	5.082834329	3.72E-07	21.87346108	4.303398392	intergenic	AMY1C;LOC100129138	dist=9415;dist=304916		
3M	rs76098744	1	111806750	C	T	421	0.01662708	14	2.811615503	0.579024995	4.855775707	1.20E-06	8.386124522	1.727041163	intronic	KCND3	.	KCND3	
3M	rs74683551	1	111811796	G	A	421	0.01662708	14	2.810351968	0.579019309	4.853641189	1.21E-06	8.38252043	1.727058121	intronic	KCND3	.		
12M	rs188069356	1	118327095	A	G	439	0.01025057	9	2.965705392	0.643538514	4.60843497	4.06E-06	7.16108651	1.553908552	intergenic	SPAG17;TBX15	dist=141867;dist=555952	SPAG17;TBX15	
12M	rs181375873	1	118341865	G	T	439	0.01025057	9	2.965562087	0.643467132	4.608723494	4.05E-06	7.162329305	1.554080932	intergenic	SPAG17;TBX15	dist=156637;dist=541182		
12M	rs146600651	1	152295232	C	T	439	0.01480638	14	3.66583614	0.743346782	4.931528902	8.16E-07	6.634223785	1.345267141	intergenic	HRNR;FLG	dist=71039;dist=6933	HRNR	
3M	rs76617932	1	180961288	T	C	421	0.01187648	9	2.08220138	0.444364012	4.685801107	2.79E-06	10.54496084	2.250407262	intergenic	KIAA1614-AS1;STX6	dist=6401;dist=11426	KIAA1614;STX6	
3M	rs183180157	1	181277985	A	C	421	0.00950119	7	1.744207622	0.371856251	4.690542692	2.72E-06	12.61385999	2.689211211	intergenic	LINC01699;CACNA1E	dist=39381;dist=205532		
3M	rs375790303	1	184561348	G	A	421	0.00831354	7	1.831959409	0.400449797	4.574754241	4.77E-06	11.42403934	2.497191924	intronic	C1orf21	.	C1orf21	
3M	rs138480898	1	184986525	C	T	421	0.00356295	3	1.261421145	0.272500077	4.629067106	3.67E-06	16.9873974	3.669723728	intergenic	NIBAN1;LINC01633	dist=12017;dist=15002		EDEM3;RNF2
3M	rs145766563	1	185160370	G	A	421	0.00356295	4	1.34508864	0.282384562	4.76332215	1.90E-06	16.86821019	3.541270076	intronic	SWT1	.	SWT1	
3M	rs147032554	1	186179732	T	G	421	0.00356295	3	1.360299196	0.273670575	4.970571635	6.68E-07	18.16260897	3.654028209	intronic	HMCN1	.	HMCN1	
3M	rs180989936	1	193075048	A	G	421	0.00475059	4	1.253423246	0.270112952	4.640367065	3.48E-06	17.17935787	3.702154942	intronic	RO60	.		GLRX2
3M	rs10494861	1	205362746	G	A	421	0.00356295	3	1.156337638	0.245524493	4.709663065	2.48E-06	19.18204982	4.072913404	intergenic	KLHDC8A;LEMD1-AS1	dist=5656;dist=10506	KLHDC8A;LEMD1	
12M	rs185899532	1	227321571	T	C	439	0.00569476	4	1.908112393	0.410717289	4.645804902	3.39E-06	11.31144228	2.434764807	intergenic	CDC42BPA;ZNF678	dist=3079;dist=241985	CDC42BPA	
3M	rs2274996	1	229668791	C	T	421	0.0415677	35	4.420411647	0.88962935	4.968823976	6.74E-07	5.585274332	1.124063633	intergenic	URB2;LINC01682	dist=8591;dist=206759	URB2	
3M	rs2274997	1	229668899	A	G	421	0.0415677	35	4.42362483	0.889790308	4.971536315	6.64E-07	5.587312281	1.123860297	intergenic	URB2;LINC01682	dist=8699;dist=206651		
3M	rs2891865	1	229670621	A	G	421	0.0415677	35	4.419830983	0.88984663	4.966958165	6.80E-07	5.581813763	1.123789164	intergenic	URB2;LINC01682	dist=10421;dist=204929		
3M	rs2385790	1	229671745	C	T	421	0.0415677	35	4.418931852	0.88972149	4.966646197	6.81E-07	5.582248215	1.123947226	intergenic	URB2;LINC01682	dist=11545;dist=203805		
3M	rs12024557	1	229676610	A	G	421	0.04275534	35	4.42434228	0.891299524	4.963923079	6.91E-07	5.569309691	1.121957291	intergenic	URB2;LINC01682	dist=16410;dist=198940		
3M	rs4562666	1	229689023	T	C	421	0.04275534	36	4.44283225	0.897203314	4.951867853	7.35E-07	5.519225993	1.114574572	intergenic	URB2;LINC01682	dist=28823;dist=186527		
3M	rs12036586	1	229690631	G	A	421	0.04750594	40	4.551491816	0.933583971	4.875289164	1.09E-06	5.222121756	1.071140927	intergenic	URB2;LINC01682	dist=30431;dist=184919		
3M	rs16850124	1	229695584	T	C	421	0.04394299	37	4.326094288	0.91378287	4.73426941	2.20E-06	5.180956622	1.094351878	intergenic	URB2;LINC01682	dist=35384;dist=179966		
3M	rs12045643	1	229698303	C	T	421	0.04275534	36	4.472324416	0.903269355	4.951263308	7.37E-07	5.481491517	1.107089479	intergenic	URB2;LINC01682	dist=38103;dist=177247		
12M	rs75626507	1	241251961	G	A	439	0.00341686	4	1.774925973	0.38713626	4.584757758	4.55E-06	11.84274951	2.583069844	intronic	RGS7	.	RGS7	
3M	rs559559983	1	246813830	C	A	421	0.00712589	5	1.645339674	0.345154188	4.76697004	1.87E-06	13.81113197	2.897255878	intergenic	LINC01341;AHCTF1	dist=22344;dist=25268	AHCTF1	
3M	rs550763536	2	7312216	T	G	421	0.00593824	5	1.504113282	0.322217093	4.66801208	3.04E-06	14.48716465	3.103497679	intergenic	LOC101929452;LOC100506274	dist=234336;dist=109045		RNF144A;ID2
3M	rs558553658	2	15542600	C	T	421	0.00475059	3	1.579685756	0.303851567	5.198873167	2.01E-07	17.10991069	3.291080613	intronic	NBAS	.	NBAS	
3M	rs536781978	2	29510815	A	G	421	0.00356295	3	1.282866581	0.270080884	4.749934759	2.03E-06	17.58708236	3.702594509	intronic	ALK	.	ALK	
3M	rs568321148	2	29647482	T	G	421	0.00356295	3	1.279867135	0.268808164	4.761265858	1.92E-06	17.71250463	3.720125102	intronic	ALK	.		
12M	rs186792608	2	42869559	A	G	439	0.00341686	3	2.044330208	0.380373238	5.374537432	7.68E-08	14.12964133	2.62899673	intergenic	HAAO;LINC01819	dist=76976;dist=158293	HAAO	
3M	rs76777840	2	48085811	G	A	421	0.00593824	5	1.714828291	0.345687108	4.960637102	7.03E-07	14.35007842	2.892789399	intergenic	FBXO11;FOXN2	dist=179313;dist=228488	FBXO11;FOXN2	
3M	rs145080832	2	48246004	G	A	421	0.00475059	4	1.617719031	0.30702202	5.269065159	1.37E-07	17.16184772	3.257095367	intergenic	FBXO11;FOXN2	dist=339506;dist=68295		
3M	rs184220112	2	48404604	C	A	421	0.00475059	4	1.587567329	0.310301123	5.116215225	3.12E-07	16.48790432	3.22267606	intergenic	FOXN2;PPP1R21	dist=25309;dist=36162	FOXN2;PPP1R21	
3M	rs189890455	2	48428258	C	T	421	0.00593824	5	1.690230255	0.334051451	5.059790188	4.20E-07	15.14673914	2.993550834	intergenic	FOXN2;PPP1R21	dist=48963;dist=12508		
3M	rs181193202	2	52300216	T	C	421	0.00356295	3	1.397666205	0.26682604	5.238117712	1.62E-07	19.63120884	3.747760153	ncRNA_intronic	LOC730100	.		NRXN1;ASB3
3M	rs183378658	2	52336233	C	T	421	0.00356295	3	1.421941101	0.268359175	5.298649105	1.17E-07	19.74461693	3.726349214	ncRNA_intronic	LOC730100	.		
3M	rs190193113	2	52858640	G	A	421	0.00356295	3	1.409901074	0.265819171	5.303985667	1.13E-07	19.95336019	3.761955902	intergenic	MIR4431;ASB3	dist=156025;dist=811339	ASB3	
3M	rs187520610	2	53132903	G	A	421	0.00475059	4	1.843352227	0.313121747	5.887014379	3.93E-09	18.80103964	3.193645952	intergenic	MIR4431;ASB3	dist=430288;dist=537076		
3M	rs149421869	2	53256291	G	T	421	0.00831354	8	1.92083422	0.404523183	4.748386252	2.05E-06	11.73821862	2.472043763	intergenic	MIR4431;ASB3	dist=553676;dist=413688		
3M	rs146479102	2	65598625	G	A	421	0.00593824	5	1.448302352	0.305828916	4.735661917	2.18E-06	15.48467677	3.269801992	intergenic	SPRED2;MIR4778	dist=166026;dist=759622	SPRED2	
3M	rs528288879	2	65648796	C	T	421	0.00593824	6	1.697856008	0.338843001	5.010745399	5.42E-07	14.78780847	2.951219288	intergenic	SPRED2;MIR4778	dist=216197;dist=709451		
3M	rs11690187	2	67338777	A	C	421	0.00593824	5	1.539986371	0.336119518	4.581663041	4.61E-06	13.63105325	2.975132201	intergenic	LINC01828;ETAA1	dist=49533;dist=58556	ETAA1	
3M	rs151272830	2	67455592	G	T	421	0.00712589	6	1.643645432	0.351116105	4.681202055	2.85E-06	13.33234787	2.848060758	intergenic	ETAA1;LINC01812	dist=43503;dist=340462		
3M	rs186142189	2	67475575	G	A	421	0.00712589	6	1.7290309	0.36845255	4.692682681	2.70E-06	12.73619271	2.714053683	intergenic	ETAA1;LINC01812	dist=63486;dist=320479		
12M	rs191298981	2	67882914	C	T	439	0.00455581	3	1.760281272	0.335644245	5.244485187	1.57E-07	15.62513066	2.979344989	intergenic	LINC01812;C1D	dist=57352;dist=158216	CD1	
12M	rs113537164	2	68007798	A	C	439	0.00455581	4	2.083286909	0.391161785	5.325895806	1.00E-07	13.61558315	2.556486955	intergenic	LINC01812;C1D	dist=182236;dist=33332		

12M	rs191823270	2	68025878	T	C	439	0.00341686	3	1.79080594	0.35569611	5.034651464	4.79E-07	14.1543619	2.811388633	intergenic	LINC01812;C1D	dist=200316;dist=15252		
12M	rs113154814	2	68062365	T	C	439	0.00569476	5	2.404201814	0.430061809	5.59036344	2.27E-08	12.99897672	2.32524716	intronic	C1D	.		
3M	rs184200893	2	69033781	C	T	421	0.00356295	3	1.121211249	0.237019909	4.730451775	2.24E-06	19.95803558	4.219054866	intronic	ANTXR1	.	ANTXR1	
3M	rs111927235	2	74256827	A	G	421	0.00475059	4	1.423024126	0.288923909	4.925255689	8.43E-07	17.04689553	3.461118894	intronic	SLC4A5	.	SLC4A5	
3M	rs111838310	2	74446364	C	A	421	0.00475059	5	1.465896314	0.287002463	5.107608836	3.26E-07	17.79639376	3.48429066	intergenic	RTKN;INO80B-WBP1	dist=4427;dist=8659	RTKN	
3M	rs112983626	2	74470023	G	A	421	0.00475059	4	1.464072657	0.286917293	5.102768952	3.35E-07	17.78480792	3.485324945	intergenic	MOGS;MRPL53	dist=4641;dist=1959	MOGS	
3M	rs113006316	2	74575233	A	G	421	0.0023753	3	0.943984037	0.203082246	4.648284397	3.35E-06	22.88867923	4.924113346	intronic	M1AP	.	M1AP	
3M	rs17746486	2	95056864	C	T	421	0.03206651	29	3.355977065	0.696974974	4.815061071	1.47E-06	6.908513578	1.434771746	intergenic	MAL;MRPS5	dist=2872;dist=28507	MAL;MRPS5	
3M	rs76554191	2	95301880	G	A	421	0.04038005	34	3.727552	0.807156314	4.618129021	3.87E-06	5.721480386	1.238917397	intronic	KCNIP3	.	KCNIP3	
3M	rs140352232	2	107421656	G	A	421	0.0023753	3	1.01264036	0.219291932	4.617772993	3.88E-06	21.05765113	4.560131294	intergenic	MIR548AU;LINC01886	dist=72131;dist=107763		ST6GAL2;RGPD4
12M	rs182996422	2	111163073	G	A	439	0.00455581	4	1.96047942	0.426062862	4.601385365	4.20E-06	10.79978043	2.347071496	intronic	BCL2L11	.	BCL2L11	
12M	rs146919974	2	114495286	T	C	439	0.00455581	5	1.870582973	0.407116317	4.594713833	4.33E-06	11.28599774	2.456300469	intronic	DPP10	.	DPP10	
3M	rs116189766	2	125636287	T	C	421	0.0023753	3	1.114549479	0.229338184	4.85985133	1.17E-06	21.19076397	4.360372887	intergenic	CNTNAP5;LINC01941	dist=715069;dist=473813		TSN;GYPC
3M	rs139877408	2	128848699	A	G	421	0.0023753	3	1.034497038	0.225706887	4.583364964	4.58E-06	20.30671294	4.430524974	intergenic	HS6ST1;LOC101927881	dist=529831;dist=15901	HS6ST1	
12M	rs144801217	2	129943819	T	C	439	0.01252847	11	3.020258487	0.622809182	4.849412265	1.24E-06	7.786353193	1.605628222	ncRNA_intronic	LINC01856	.		;RAB6C
12M	rs74492945	2	147426871	G	A	439	0.02961276	25	5.284785249	1.136173009	4.651391299	3.30E-06	4.093911105	0.880147647	intergenic	PABPC1P2;ACVR2A	dist=835881;dist=417647	ACVR2A	
12M	rs75380640	2	147428229	A	G	439	0.02961276	25	5.284373278	1.136163974	4.65106569	3.30E-06	4.093657074	0.880154646	intergenic	PABPC1P2;ACVR2A	dist=837239;dist=416289		
12M	rs74517425	2	147435566	A	C	439	0.02961276	25	5.255817679	1.12800262	4.659402016	3.17E-06	4.130665953	0.886522764	intergenic	PABPC1P2;ACVR2A	dist=844576;dist=408952		
12M	rs75343152	2	147447179	T	C	439	0.02961276	25	5.373706425	1.145505076	4.691124061	2.72E-06	4.095245108	0.872977362	intergenic	PABPC1P2;ACVR2A	dist=856189;dist=397339		
12M	rs76487561	2	147457410	C	T	439	0.0284738	25	5.507313038	1.141042849	4.826561108	1.39E-06	4.229956054	0.876391277	intergenic	PABPC1P2;ACVR2A	dist=866420;dist=387108		
12M	rs10175536	2	147460050	A	G	439	0.02733485	24	5.059239074	1.084265764	4.666050745	3.07E-06	4.30341979	0.92228311	intergenic	PABPC1P2;ACVR2A	dist=869060;dist=384468		
12M	rs75304512	2	147460438	A	G	439	0.02733485	24	5.052915513	1.079656239	4.680115142	2.87E-06	4.334819707	0.926220739	intergenic	PABPC1P2;ACVR2A	dist=869448;dist=384080		
12M	rs74380227	2	147462714	C	A	439	0.02733485	24	5.051193478	1.079667701	4.678470491	2.89E-06	4.333250397	0.926210907	intergenic	PABPC1P2;ACVR2A	dist=871724;dist=381804		
12M	rs10210722	2	147465215	T	C	439	0.02733485	24	5.051665699	1.07945012	4.679850978	2.87E-06	4.335402712	0.9263976	intergenic	PABPC1P2;ACVR2A	dist=874225;dist=379303		
12M	rs1258074269	2	147466224	A	C	439	0.02733485	24	5.051665699	1.07945012	4.679850978	2.87E-06	4.335402712	0.9263976	intergenic	PABPC1P2;ACVR2A	dist=875234;dist=378294		
12M	rs6708887	2	147466224	A	C	439	0.02733485	24	5.051665699	1.07945012	4.679850978	2.87E-06	4.335402712	0.9263976	intergenic	PABPC1P2;ACVR2A	dist=875234;dist=378294		
12M	rs77928469	2	147466550	C	T	439	0.02733485	24	5.051484579	1.079409821	4.679857902	2.87E-06	4.335570987	0.926432186	intergenic	PABPC1P2;ACVR2A	dist=875560;dist=377968		
12M	rs77592434	2	147469077	A	G	439	0.02733485	24	5.051588616	1.07940957	4.679955372	2.87E-06	4.335662295	0.926432401	intergenic	PABPC1P2;ACVR2A	dist=878087;dist=375441		
12M	rs6724770	2	147469854	A	G	439	0.02733485	24	5.05104599	1.078982106	4.68130654	2.85E-06	4.338632229	0.926799429	intergenic	PABPC1P2;ACVR2A	dist=878864;dist=374664		
12M	rs80339745	2	147470110	G	A	439	0.02733485	24	5.050651164	1.078960151	4.681035866	2.85E-06	4.338469649	0.926818288	intergenic	PABPC1P2;ACVR2A	dist=879120;dist=374408		
12M	rs78849093	2	147478023	C	A	439	0.02733485	24	5.057603105	1.078451085	4.689691702	2.74E-06	4.348543728	0.927255778	intergenic	PABPC1P2;ACVR2A	dist=887033;dist=366495		
12M	rs75330406	2	147481113	G	A	439	0.02733485	24	5.0599299	1.078749077	4.690553168	2.72E-06	4.348141072	0.926999635	intergenic	PABPC1P2;ACVR2A	dist=890123;dist=363405		
3M	rs142894171	2	150581757	G	T	421	0.00356295	3	1.287888043	0.266960796	4.82425907	1.41E-06	18.07103941	3.74586836	intergenic	LINC01920;LINC02612	dist=9536;dist=47140		RND3;RBM43
12M	rs138454387	2	163831400	G	A	439	0.00569476	5	2.115842897	0.458436819	4.615342424	3.92E-06	10.0675649	2.181325669	intergenic	FIGN;GRB14	dist=95392;dist=661017	FIGN;GRB14	
12M	rs146300183	2	164138767	T	C	439	0.00455581	4	1.984193903	0.407444048	4.869856149	1.12E-06	11.95220834	2.454324722	intergenic	FIGN;GRB14	dist=402759;dist=353650		
3M	rs541508507	2	169166755	G	A	421	0.00475059	4	1.442660176	0.305446461	4.723119638	2.32E-06	15.46300334	3.273896179	intronic	LRP2	.	LRP2	
3M	rs142549310	2	169173996	C	T	421	0.00475059	4	1.443948619	0.307707803	4.692596694	2.70E-06	15.25017126	3.249836338	exonic	LRP2	.		
3M	rs556293455	2	176085692	G	A	421	0.00356295	3	1.630922146	0.336702149	4.843812709	1.27E-06	14.38604633	2.969984018	intergenic	EVX2;HOXD13	dist=1730;dist=7029	EVX2;HOXD13	
3M	rs184098071	2	176251692	G	A	421	0.00356295	3	1.695892029	0.34869637	4.863520744	1.15E-06	13.94772404	2.86782452	intergenic	HOXD1;MTX2	dist=60785;dist=17750	MTX2	
12M	rs192500829	2	176385923	A	G	439	0.00455581	3	1.674098367	0.340713541	4.913506992	8.95E-07	14.42122604	2.935016895	intergenic	MTX2;MIR1246	dist=47898;dist=215057		
3M	rs532416695	2	176622062	G	A	421	0.00356295	3	1.400161077	0.265436681	5.274934396	1.33E-07	19.87266561	3.767376828	intergenic	MIR1246;LINC01116	dist=21010;dist=7519		
3M	rs112557251	2	187510673	T	C	421	0.0023753	3	1.043551353	0.224794821	4.642239298	3.45E-06	20.65100643	4.448501059	intronic	TFPI	.	TFPI	
12M	rs140561530	2	205755707	G	T	439	0.0022779	3	1.412604553	0.289304563	4.882759325	1.05E-06	16.87757453	3.456564906	intronic	NRP2	.	NRP2	
3M	rs185158855	2	222785307	C	A	421	0.00475059	4	1.284200145	0.277572279	4.626543222	3.72E-06	16.66788644	3.602665239	intergenic	MOGAT1;ACSL3	dist=75377;dist=75728	MOGAT1;ACSL3	
3M	rs185510569	2	222950143	G	A	421	0.00356295	3	1.507358682	0.277399453	5.433892049	5.51E-08	19.58869056	3.604909775	intergenic	ACSL3;KCNE4	dist=5505;dist=102047	ACSL3;KCNE4	
3M	rs147559909	2	236142879	T	C	421	0.00593824	5	1.858263542	0.329805301	5.634425932	1.76E-08	17.08409752	3.032091952	intergenic	AGAP1;GBX2	dist=11086;dist=22356	AGAP1	
12M	rs146298016	2	237657156	G	A	439	0.00797267	7	2.369872335	0.517388552	4.580449887	4.64E-06	8.85301746	1.93278339	intronic	LRRFIP1	.	LRRFIP1	
3M	rs181217257	2	239067423	C	T	421	0.00475059	4	1.788144205	0.287937999	6.210170973	5.29E-10	21.56773677	3.472969885	intronic	HDAC4	.	HDAC4	
3M	rs188076929	2	239072023	T	C	421	0.00475059	4	1.865485168	0.305579814	6.104739516	1.03E-09	19.97756143	3.272467463	intronic	HDAC4	.		
12M	rs115690445	2	239682619	A	C	439	0.00797267	7	2.51110667	0.515854898	4.867854661	1.13E-06	9.436480453	1.938529621	intergenic	HDAC4-AS1;LOC150935	dist=280255;dist=80241		
3M	rs112475378	3	1584977	T	C	421	0.01662708	14	2.445371748	0.506000821	4.832742644	1.35E-06	9.550859286	1.976281377	intergenic	CNTN6;CNTN4	dist=180760;dist=513826	CNTN6;CNTN4	
3M	rs145676540	3	2002951	C	T	421	0.00593824	5	1.68032939	0.346683687	4.846866048	1.25E-06	13.98065798	2.884473769	intergenic	CNTN6;CNTN4	dist=598734;dist=95852		
12M	rs55939894	3	7921601	A	G	439	0.18792711	165	-12.24219411	2.600577928	-4.707489817	2.51E-06	-1.810170642	0.384529911	intergenic	GRM7;LOC101927394	dist=180068;dist=31204	GRM7	
12M	rs11920261	3	7935524	G	T	439	0.1856492	162	-12.09011362	2.598457274	-4.65280447	3.27E-06	-1.790602646	0.384843734	intergenic	GRM7;LOC101927394	dist=193991;dist=17281		
12M	rs2171551	3	7936391	C	A	439	0.1856492	162	-12.08866418	2.598460193	-4.652241434	3.28E-06	-1.790383954	0.384843302	intergenic	GRM7;LOC101927394	dist=194858;dist=16414		
3M	rs146007933	3	28185932	T	C	421	0.02137767	18	3.108745063	0.643323442	4.832320511	1.35E-06	7.511494525	1.554428045	intergenic	LINC01980;CMC1	dist=325607;dist=55687	CMC1	
3M	rs73057656	3	33899079	A	G	421	0.0368171	33	3.638911487	0.783762258	4.642876649	3.44E-06	5.923832897	1.27589711	intergenic	PDCD6IP;LOC101928135	dist=29372;dist=976718	PDCD6IP	
3M	rs73085348	3	42669729	A	G	421	0.01187648	11	2.448275953	0.491389455	4.982353469	6.28E-07	10.13931704	2.035045707	intergenic	ZBTB47;KLHL40	dist=2149;dist=15808	ZBTB47	
12M	rs2229593	3	42864724	C	T	439	0.02733485	24	4.870496863	1.032388754	4.717696551	2.39E-06	4.569689987	0.968627367	exonic	ACKR2	.	ACKR2	
12M	rs139029292	3	44072032	G	A	439	0.00569476	6	2.199467961	0.481405962	4.568842376	4.90E-06	9.490622744	2.077248888	intergenic	ABHD5;MIR138-1	dist=337661;dist=42180	ABHD5	
3M	rs142684595	3	55285273	T	C	421	0.00593824	5	1.730096674	0.321166729	5.386911268	7.17E-08	16.77294307	3.113647549	intergenic	LINC02017;WNT5A	dist=96059;dist=180442	WNT5A	
12M	rs148953849	3	56866690	G	A	439	0.00455581	4	1.732750622	0.35482831	4.883349426	1.04E-06	13.76256993	2.818264419	intronic	ARHGEF3	.	ARHGEF3	
3M	rs80203220	3	123003484	C	T	421	0.00593824	6	1.662045235	0.351841788	4.723842626	2.31E-06	13.42604201	2.842186558	intronic	SEMA5B	.	SEMA5B	
12M	rs114280794	3	133123359	G	A	439	0.01594533	14	4.764666379	0.86324065	5.519511134	3.40E-08	6.393942562	1.158425521	intronic	TMEM108	.	TMEM108	
3M	rs192443987	3	135813503	G	A	421	0.00475059	4	1.31410558	0.27206124	4.830183004	1.36E-06	17.75402845	3.675643022	intergenic	EPHB1;PPP2R3A	dist=553038;dist=152225	EPHB1;PPP2R3A	
3M	rs148248743	3	136415753	C	T	421	0.0023753	3	1.069833829	0.216429281	4.943110391	7.69E-07	22.83937904	4.620446891	intronic	STAG1	.		
12M	rs148248743	3	136415753	C	T	439	0.0022779	3	1.586456973	0.298755282	5.310222348	1.09E-07	17.7744886	3.347221159	intronic	STAG1	.	STAG1	
3M	rs576124203	3	142122354	T	G	421	0.00356295	3	1.313230925	0.260150702	5.047962263	4.47E-07	19.4039925	3.843925823	intronic	TFDP2	.	TFDP2	
3M	rs545552231	3	142145508	C	T	421	0.00356295	3	1.275833022	0.253432385	5.034214646	4.80E-07	19.8641332	3.945825635	intronic	TFDP2	.		
12M	rs138138661	3	147940027	C	T	439	0.00455581	4	1.755727133	0.380325922	4.616375137	3.91E-06	12.13794502	2.629323801	intergenic	LOC440982;LINC02032	dist=430117;dist=138132		
12M	rs148997617	3	148037789	C	T	439	0.00455581	4	1.904562472	0.412852965	4.613173773	3.97E-06	11.17389037	2.422169838	intergenic	LOC440982;LINC02032	dist=527879;dist=40370		
3M	rs193153124	3	148612923	A	G	421	0.00593824	5	1.529063164	0.32624874	4.686801743	2.78E-06	14.36573132	3.065145937	intergenic	LINC02046;AGTR1	dist=212967;dist=84948	AGTR1	
3M	rs188720948	3	150352093	T	C	421	0.00356295	3	1.368098189	0.277991774	4.921362129	8.59E-07	17.7032653	3.597228742	intergenic	LINC01214;TSC22D2	dist=28346;dist=56205	TSC22D2	
3M	rs16823323	3	153939413	G	A	421	0.01662708	14	3.214602568	0.582174532	5.521716236	3.36E-08	9.484640653	1.717697949	intergenic	LINC02006;ARHGEF26-AS	dist=176887;dist=84988	ARHGEF26	
3M	rs139943877	3	155733500	G	A	421	0.00712589	7	1.713092922	0.362264246	4.728849011	2.26E-06	13.0535902	2.760415942	intronic	PLCH1	.	PLCH1	
3M	rs187047882	3	164567833	G	A	421	0.00356295	3	1.161853087	0.250171732	4.644222095	3.41E-06	18.56413613	3.997254169	intergenic	MIR1263;LINC01324	dist=396277;dist=146262		
12M	rs139635002	3	164614597	T	C	439	0.00797267	8	2.540439901	0.548123379	4.63479574	3.57E-06	8.4557527	1.824406765	intergenic	MIR1263;LINC01324	dist=443041;dist=99498		
3M	rs141169929	3	165090674	A	G	421	0.00475059	5	1.473704308	0.315071386	4.677366376	2.91E-06	14.84541783	3.173883899	intergenic	SI;SLITRK3	dist=12178;dist=96046	SLITRK3	
12M	rs754653672	3	165112751	A	G	439	0.00341686	3	1.695149727	0.37024662	4.578434038	4.68E-06	12.36590367	2.700902442	intergenic	SI;SLITRK3	dist=34255;dist=73969		
12M	rs994788245	3	165182662	G	T	439	0.00341686	3	1.457659335	0.317040616	4.597705341	4.27E-06	14.50194425	3.154169999	intergenic	SI;SLITRK3	dist=104166;dist=4058		
3M	rs186649043	3	175214503	C	T	421	0.00356295	3	1.162234876	0.245948741	4.725516671	2.30E-06	19.21342086	4.065887859	intronic	NAALADL2	.	NAALADL2	
3M	rs189709453	3	177856520	G	A	421	0.00356295	3	1.314463975	0.246356303	5.335621454	9.52E-08	21.65814876	4.05916142	ncRNA_intronic	LINC02015	.		
12M	rs189709453	3	177856520	G	A	439	0.00341686	3	1.623833795	0.337340968	4.813627607	1.48E-06	14.26932412	2.964359789	ncRNA_intronic	LINC02015	.		TBL1XR1;ZMAT3
3M	rs186767531	3	177921989	T	C	421	0.00356295	3	1.293967205	0.247047316	5.237730262	1.63E-07	21.20132426	4.047807581	intergenic	LINC02015;LINC01014	dist=22765;dist=497212		
12M	rs186767531	3	177921989	T	C	439	0.00341686	3	1.610325603	0.338183106	4.761697361	1.92E-06	14.08023428	2.956977988	intergenic	LINC02015;LINC01014	dist=22765;dist=497212		
3M	rs182868205	3	177994660	C	T	421	0.00356295	3	1.323927501	0.251356167	5.267137522	1.39E-07	20.95487681	3.978418395	intergenic	LINC02015;LINC01014	dist=95436;dist=424541		
12M	rs182868205	3	177994660	C	T	439	0.00341686	3	1.649581368	0.344172256	4.792894657	1.64E-06	13.92586001	2.90552182	intergenic	LINC02015;LINC01014	dist=95436;dist=424541		
12M	rs76356799	3	179875980	G	A	439	0.00455581	4	2.106771109	0.432864845	4.867041374	1.13E-06	11.24378991	2.310189917	intronic	PEX5L	.	PEX5L	

12M	rs571346923	3	184247590	G	A	439	0.00341686	3	1.651446737	0.342609142	4.820206279	1.43E-06	14.06911168	2.918777925	intronic	ALG3	.	GMNC	
3M	rs191792521	3	195919734	G	A	421	0.00831354	6	1.587589638	0.337332296	4.706307863	2.52E-06	13.95154842	2.964435992	intergenic	TNK2-AS1;SDHAP1	dist=6470;dist=40187	TNK2	
3M	rs113651406	4	997179	C	T	421	0.00356295	3	1.369997433	0.265704278	5.156098506	2.52E-07	19.40540264	3.763582603	intronic	IDUA	.	IDUA	
3M	rs12502861	4	2424578	T	C	421	0.01068884	9	1.987294963	0.422008794	4.709131639	2.49E-06	11.15884718	2.369618867	intronic	CFAP99	.	CFAP99	
3M	rs189765693	4	4323066	T	C	421	0.00475059	4	1.425732565	0.30309955	4.70384257	2.55E-06	15.51913413	3.299246074	intergenic	ZBTB49;NSG1	dist=1283;dist=63466	ZBTB49;NSG1	
12M	rs189094663	4	11621826	G	A	439	0.0022779	3	1.615404085	0.319409161	5.057475753	4.25E-07	15.83384692	3.13078059	intergenic	HS3ST1;LINC02360	dist=192932;dist=119125	HS3ST1;LINC02360	
3M	rs183962155	4	21258020	A	C	421	0.00593824	6	1.557852883	0.336419694	4.630682783	3.64E-06	13.76460079	2.972477589	intronic	KCNIP4	.	KCNIP4	
3M	rs113751774	4	23427231	C	T	421	0.00712589	7	1.694246649	0.337089114	5.026109061	5.01E-07	14.91032743	2.966574592	intergenic	GBA3;PPARGC1A	dist=607659;dist=364790	PPARGC1A	
3M	rs113063005	4	23507444	T	C	421	0.00593824	6	2.130262689	0.316937024	6.721406875	1.80E-11	21.2073894	3.155200957	intergenic	GBA3;PPARGC1A	dist=687872;dist=284577		
3M	rs145875128	4	32071514	G	A	421	0.00356295	3	1.153690598	0.217089638	5.314351295	1.07E-07	24.47998599	4.606392132	ncRNA_intronic	LINC02506	.		
12M	rs146847867	4	32390284	G	T	439	0.00455581	4	2.185302341	0.426711618	5.121262811	3.03E-07	12.00169528	2.3435031	intergenic	LINC02353;LOC101928622	dist=37064;dist=1506055		
12M	rs183086778	4	32609199	A	G	439	0.00455581	5	2.346647932	0.430540407	5.45047084	5.02E-08	12.6596035	2.322662367	intergenic	LINC02353;LOC101928622	dist=255979;dist=1287140	PCDH7;ARAP2	
12M	rs144899985	4	32738728	A	G	439	0.00455581	5	2.245036023	0.430094163	5.219870935	1.79E-07	12.13657701	2.32507224	intergenic	LINC02353;LOC101928622	dist=385508;dist=1157611		
12M	rs192772770	4	32808172	T	G	439	0.00569476	5	2.334772732	0.461927473	5.054414098	4.32E-07	10.94200798	2.164842011	intergenic	LINC02353;LOC101928622	dist=454952;dist=1088167		
12M	rs76887296	4	32865383	C	T	439	0.00569476	5	2.374205059	0.477289182	4.974353383	6.55E-07	10.42209538	2.09516586	intergenic	LINC02353;LOC101928622	dist=512163;dist=1030956		
3M	rs143287889	4	35570658	C	T	421	0.0023753	3	1.058108262	0.230199399	4.596485777	4.30E-06	19.96741	4.34406	intergenic	LINC02484;ARAP2	dist=1300911;dist=495346		
3M	rs77141817	4	37052137	T	C	421	0.00356295	3	1.553761456	0.274563203	5.659030198	1.52E-08	20.61102923	3.642148656	intergenic	LINC02616;MIR4801	dist=31431;dist=189773		;PCGKA1,RELL1
3M	rs190822761	4	37097734	G	T	421	0.00356295	3	1.550650175	0.274463961	5.649740562	1.61E-08	20.58463536	3.643465595	intergenic	LINC02616;MIR4801	dist=77028;dist=144176		
12M	rs147267707	4	43024541	G	A	439	0.00455581	4	2.094031845	0.428388026	4.888166139	1.02E-06	11.41060403	2.334332284	intronic	GRXCR1	.		
12M	rs77618729	4	43048951	T	C	439	0.00455581	4	2.093237083	0.42828012	4.887542018	1.02E-06	11.41202168	2.334920424	intergenic	GRXCR1;LINC02383	dist=18293;dist=408583		ATP8A1;KCTD8
12M	rs556435089	4	43549016	G	A	439	0.00455581	4	2.093380466	0.427400072	4.897941303	9.68E-07	11.45985137	2.339728197	intergenic	LINC02383;LINC02475	dist=56473;dist=467845		
12M	rs532464521	4	43569022	T	C	439	0.00455581	4	2.094609056	0.427328656	4.901634909	9.50E-07	11.47041005	2.340119219	intergenic	LINC02383;LINC02475	dist=76479;dist=447839		
12M	rs191122698	4	60001148	T	C	439	0.00683371	6	2.484118908	0.503688379	4.931856703	8.15E-07	9.791483996	1.98535452	intergenic	LINC02429;MIR548AG1	dist=954189;dist=921471		IGFBP7;ADGRL3
12M	rs181164671	4	60202387	G	T	439	0.00683371	6	2.365357436	0.473829215	4.992004204	5.98E-07	10.53545041	2.110465052	intergenic	LINC02429;MIR548AG1	dist=1155428;dist=720232		
3 month	rs999769259	4	61646247	G	A	421	0.00356295	3	0.969956079	0.209381164	4.632489677	3.61E-06	22.12467252	4.775978806	intronic	ADGRL3	.	ADGRL3	
12M	rs183601329	4	79385832	C	T	439	0.00341686	3	1.528621191	0.307223916	4.97559308	6.50E-07	16.19533121	3.254954928	intergenic	NAA11;GK2	dist=59771;dist=20529		BMP2K,PAQR3;ANTXR2
12M	rs564991659	4	79495431	A	G	439	0.0022779	3	1.478600686	0.29470879	5.017158418	5.24E-07	17.02412208	3.393180096	ncRNA_intronic	LINC00989	.		
12M	rs115901263	4	86154667	A	G	439	0.00569476	6	2.238202026	0.474593389	4.716041306	2.40E-06	9.93701433	2.107066856	ncRNA_intronic	MAPK10-AS1	.	MAPK10	
3M	rs147630370	4	86529522	T	C	421	0.00475059	4	1.530518883	0.309272178	4.948776482	7.47E-07	16.00136328	3.23339786	intergenic	MAPK10;MIR4452	dist=76327;dist=12960		
3M	rs147171192	4	88214436	A	G	421	0.00712589	5	1.584612591	0.345114993	4.591549547	4.40E-06	13.30440472	2.89758492	intronic	ABCG2	.	ABCG2	
3M	rs142993106	4	90036221	G	A	421	0.01781473	15	2.691736025	0.577013528	4.664944401	3.09E-06	8.084636111	1.733061622	intergenic	MMRN1;CCSER1	dist=81611;dist=91173	MMRN1;CCSER1	
3M	rs146526206	4	90071867	T	C	421	0.01781473	15	2.815290022	0.58984771	4.772909977	1.82E-06	8.091766558	1.695352855	intergenic	MMRN1;CCSER1	dist=117257;dist=55527		
3M	rs17017794	4	90904734	T	C	421	0.0391924	33	4.452559757	0.873737599	5.095991934	3.47E-07	5.83240545	1.144508375	intronic	CCSER1	.		
12M	rs979665	4	100960199	A	G	439	0.64464692	320	14.57615127	2.841511195	5.129718053	2.90E-07	1.805278143	0.351925413	intergenic	LINC01216;PPP3CA	dist=285086;dist=63219	PPP3CA	
3M	rs145116559	4	111756440	T	C	421	0.00356295	4	1.144113716	0.250422362	4.568736218	4.91E-06	18.24412236	3.993253602	intergenic	MIR297;FAM241A	dist=895793;dist=389014	PITX2;AP1AR	
3M	rs181415102	4	111768110	T	C	421	0.00356295	4	1.144945045	0.250383121	4.572772469	4.81E-06	18.26310193	3.993879436	intergenic	MIR297;FAM241A	dist=907463;dist=377344		
3M	rs191423619	4	125595472	G	T	421	0.0023753	3	1.225954306	0.23771753	5.157189324	2.51E-07	21.69461097	4.206673365	intergenic	MIR2054;INTU	dist=88165;dist=2037485	INTU	
3M	rs149298750	4	126237546	A	C	421	0.00356295	3	1.219140188	0.234426329	5.200525875	1.99E-07	22.18405204	4.265732461	intergenic	MIR2054;INTU	dist=730239;dist=1395411		
12M	rs181669665	4	128644045	G	A	439	0.00569476	4	1.881997947	0.380114274	4.951137266	7.38E-07	13.02539159	2.630787815	intergenic	LINC02615;JADE1	dist=124649;dist=165655	JADE1	
12M	rs181132315	4	128726214	C	T	439	0.00341686	3	1.85813691	0.353270418	5.259814619	1.44E-07	14.88891893	2.830692717	intergenic	LINC02615;JADE1	dist=206818;dist=83486		
12M	rs13103178	4	130157785	T	C	439	0.01936219	21	4.095545102	0.855702041	4.786181292	1.70E-06	5.593280207	1.168631079	intergenic	LINC02465;LINC02479	dist=202417;dist=218576		JADE1,SCLT1,C4orf33;
12M	rs1369229648	4	130157785	T	C	439	0.01936219	21	4.095545102	0.855702041	4.786181292	1.70E-06	5.593280207	1.168631079	intergenic	LINC02465;LINC02479	dist=202417;dist=218576		
3M	rs112679237	4	138219706	T	C	421	0.01781473	18	3.098512369	0.596371661	5.195606321	2.04E-07	8.712027524	1.676806707	intronic	SLC7A11	.	SLC7A11	
12M	rs184618624	4	150066449	T	C	439	0.0022779	3	1.489513245	0.308282145	4.831655903	1.35E-06	15.67283732	3.243781766	intergenic	IQCM;DCLK2	dist=250606;dist=11996	DCLK2	
12M	rs144967461	4	152847797	T	G	439	0.01822323	16	4.171868899	0.801202658	5.207008309	1.92E-07	6.498990306	1.248123667	intronic	ARFIP1	.	ARFIP1	
3M	rs531769270	4	153489891	T	C	421	0.00356295	3	1.339792194	0.284142497	4.715212285	2.41E-06	16.59453383	3.519360917	intronic	TMEM131L	.		MND1;TLR2
3M	rs116651654	4	162317591	C	T	421	0.00712589	6	1.661626207	0.326131851	5.094952243	3.49E-07	15.62236937	3.066244515	intergenic	FSTL5;MIR4454	dist=153557;dist=775983	FSTL5	
12M	rs114259958	4	162398591	A	G	439	0.00341686	3	1.650939137	0.357816014	4.613933065	3.95E-06	12.89470814	2.794732381	intergenic	FSTL5;MIR4454	dist=234557;dist=694983		
3M	rs567982164	5	25631188	G	A	421	0.0023753	3	1.14494858	0.228986639	5.000067194	5.73E-07	21.8356286	4.367067031	intergenic	LINC02211;CDH9	dist=328908;dist=1249409	CDH9	
3M	rs191986449	5	25745578	C	T	421	0.00475059	3	1.135135845	0.247721071	4.582314459	4.60E-06	18.49787925	4.036798308	intergenic	LINC02211;CDH9	dist=443298;dist=1135019		
12M	rs185242027	5	29071862	G	A	439	0.00455581	3	1.595602789	0.344148816	4.636374483	3.55E-06	13.47200475	2.905719716	ncRNA_intronic	LOC101929645	.		No genes
12M	rs10038482	5	37593566	C	T	439	0.00455581	4	1.945686385	0.408444718	4.763646826	1.90E-06	11.66289247	2.448311744	intronic	WDR70	.	WDR70	
12M	rs142934021	5	53366842	G	A	439	0.00797267	7	2.548043395	0.487663617	5.225002041	1.74E-07	10.7143569	2.050593821	intergenic	LOC257396;FST	dist=251716;dist=113787	FST	
3M	rs185771987	5	73989664	T	C	421	0.00356295	3	1.153019969	0.251461612	4.585272329	4.53E-06	18.23448239	3.976750144	intergenic	ARHGEF28;LINC01335	dist=47671;dist=316746	ARHGEF28	
3M	rs139360368	5	74076284	A	C	421	0.00356295	4	1.280137576	0.264060375	4.847897286	1.25E-06	18.35904869	3.78701272	intergenic	ARHGEF28;LINC01335	dist=134291;dist=230126		
3M	rs181933850	5	92169830	A	G	421	0.00831354	7	1.957149723	0.420974257	4.64909597	3.33E-06	11.04365858	2.375442162	intergenic	ARRDC3-AS1;NR2F1-AS1	dist=749115;dist=1279415	ARRDC3;NR2F1	
3M	rs190190051	5	92179668	G	A	421	0.00831354	7	1.947160785	0.419607459	4.640434158	3.48E-06	11.05898873	2.383179753	intergenic	ARRDC3-AS1;NR2F1-AS1	dist=758953;dist=1269577		
3M	rs182531466	5	92234256	C	A	421	0.00712589	6	1.668434681	0.362732486	4.599628498	4.23E-06	12.68049781	2.756852606	intergenic	ARRDC3-AS1;NR2F1-AS1	dist=813541;dist=1214989		
3M	rs187236873	5	92234630	G	A	421	0.00712589	6	1.81660653	0.381998785	4.755529603	1.98E-06	12.44906998	2.617809375	intergenic	ARRDC3-AS1;NR2F1-AS1	dist=813915;dist=1214615		
3M	rs183816745	5	92336071	A	G	421	0.00593824	5	1.692149037	0.312309144	5.418186024	6.02E-08	17.34879088	3.201955563	intergenic	ARRDC3-AS1;NR2F1-AS1	dist=915356;dist=1113174		
3M	rs111407636	5	95744325	C	T	421	0.00356295	3	1.331610247	0.26725959	4.98246012	6.28E-07	18.64277396	3.741680518	intronic	RHOBTB3	.	RHOBTB3	
3M	rs111676272	5	95758594	C	A	421	0.00356295	3	1.327188035	0.267072797	4.969386812	6.72E-07	18.6068625	3.744297477	intronic	RHOBTB3	.		
3M	rs75848314	5	95762636	T	C	421	0.00356295	3	1.401525911	0.264060948	5.307584942	1.11E-07	20.0998481	3.787004507	intronic	RHOBTB3	.		
3M	rs111846247	5	95775939	T	C	421	0.00356295	3	1.3787786	0.259425442	5.314739335	1.07E-07	20.48657718	3.854672052	intronic	RHOBTB3	.		
3M	rs137873790	5	97751337	A	G	421	0.01306413	11	2.280553097	0.497276469	4.586086892	4.52E-06	9.222408811	2.010953789	intergenic	LINC01340;LINC02234	dist=80286;dist=89421		RIOK2;RGMB
12M	rs569429270	5	116060403	C	T	439	0.00341686	3	1.696972577	0.357132375	4.751662676	2.02E-06	13.30504599	2.800082182	downstream	ARL14EPL	dist=914		LVRN;COMMD10
12M	rs140512918	5	116111989	T	C	439	0.00341686	3	1.688393978	0.363434391	4.645663762	3.39E-06	12.78267517	2.75152827	intronic	COMMD10	.	COMMD10	
12M	rs185275344	5	120442008	C	T	439	0.00455581	5	1.963836945	0.429890979	4.56822088	4.92E-06	10.6264637	2.326171168	intergenic	FAM170A;PRR16	dist=806186;dist=22270	FAM170A;PRR16	
3M	rs189912648	5	135429749	C	T	421	0.00356295	4	1.303529678	0.277126356	4.703737665	2.55E-06	16.97325991	3.608462274	intergenic	MACROH2A1;DCANP1	dist=29862;dist=14465	MACROH2A1;DCANP1	
3M	rs191006910	5	154836449	G	A	421	0.00356295	3	1.226165352	0.263817655	4.647775955	3.36E-06	17.61738031	3.790496891	intronic	FAXDC2	.	FAXDC2	
3M	rs74343174	5	162066176	C	A	421	0.00593824	5	1.498573008	0.318559187	4.704221604	2.55E-06	14.76718237	3.139134082	intergenic	LINC01202;GABRG2	dist=64980;dist=1289		
3M	rs775626702	5	163203061	A	C	421	0.00356295	3	1.143758046	0.245686137	4.655362562	3.23E-06	18.94841366	4.070233717	intergenic	GABRG2;CCNG1	dist=1047522;dist=234510	GABRG2;CCNG1	
3M	rs371245624	5	163453950	T	C	421	0.00356295	3	1.17843308	0.245958889	4.791179069	1.66E-06	19.47959308	4.065720108	UTR3	NUDCD2	NM_001329991:c.*170>0;NM_14	NUDCD2	
3M	rs290120	5	163841238	T	G	421	0.00712589	6	1.664921355	0.353031552	4.716069562	2.40E-06	13.3587764	2.832608007	intergenic	MAT2B;LINC02143	dist=321884;dist=607184	MAT2B	
3M	rs545428520	5	168394261	T	C	421	0.00356295	4	1.606350972	0.272919814	5.885798274	3.96E-09	21.56603503	3.664079879	intronic	WWC1	.	WWC1	
3M	rs528404963	5	168425020	T	C	421	0.00356295	4	1.58410842	0.273744969	5.786803767	7.17E-09	21.13939767	3.653035168	intronic	WWC1	.		
3M	rs72832764	5	170577669	G	A	421	0.00356295	3	1.208783888	0.257601337	4.692459687	2.70E-06	18.21597569	3.881967434	intronic	KCNIP1	.		
3M	rs72837643	5	170761951	T	C	421	0.00356295	4	1.251913633	0.271928683	4.60383075	4.15E-06	16.93028738	3.677434793	intergenic	KCNIP1;GABRP	dist=25319;dist=21768	KCNIP1;GABRP	
12M	rs141926026	5	175205538	C	T	439	0.0022779	3	1.568445555	0.318998882	4.916774461	8.80E-07	15.41314011	3.134807227	intergenic	LINC01951;DRD1	dist=209807;dist=234499	DRD1	
3M	rs142311947	5	177957468	G	A	421	0.00712589	8	1.813233127	0.388171459	4.671217019	2.99E-06	12.03390127	2.576181159	ncRNA_intronic	LOC100128340	.		FAM153A;FAM153C
3M	rs151015676	5	177963936	T	G	421	0.0023753	3	1.054373875	0.228793957	4.608399149	4.06E-06	20.14213671	4.370744819	intergenic	LOC100128340;PROP1	dist=4136;dist=28299		
3M	rs1877768	6	16534692	C	T	421	0.01781473	15	2.650393922	0.565512508	4.686711405	2.78E-06	8.287546847	1.768307483	intronic	ATXN1	.	ATXN1	
3M	rs150586237	6	24491120	C	T	421	0.00356295	3	1.542166452	0.264483938	5.830851057	5.51E-09	22.04614425	3.780947933	intergenic	GPLD1;ALDH5A1	dist=1542;dist=3849	GPLD1;ALDH5A1	
12M	rs17875371	6	30492455	C	T	439	0.01822323	16	3.620688814	0.776176074	4.664777664	3.09E-06	6.009947767	1.288367464	intronic	HLA-E	.	HLA-E	
12M	rs12212412	6	81267112	C	T	439	0.00911162	8	2.865042865	0.571860889	5.010034645	5.44E-07	8.760932498	1.748677029	intergenic	BCKDHB;TENT5A	dist=920842;dist=478618	BCKDHB	
12M	rs71568803	6	81284001	A	G	439	0.00911162	8	2.871784743	0.573932376	5.003698802	5.62E-07	8.718272413	1.74236555	intergenic	BCKDHB;TENT5A	dist=937731;dist=461729		
12M	rs12193585	6	81292327	C	T	439	0.01025057	9	2.759336125	0.592983165	4.653312755	3.27E-06	7.847293194	1.686388517	intergenic	BCKDHB;TENT5A	dist=946057;dist=453403		

3M	rs571986619	6	84969114	A	G	421	0.0023753	3	1.102004226	0.230254532	4.786026206	1.70E-06	20.78580673	4.343019831	intergenic	TBX18;LINC02535	dist=204516;dist=418105	TBX18	
3M	rs56224400	6	97644799	T	C	421	0.01662708	14	2.845683986	0.580546566	4.901732531	9.50E-07	8.443306398	1.722514712	ncRNA_intronic	LOC101927314	.		MMS22L;POU3F2
3M	rs147627638	6	98725240	A	G	421	0.00712589	6	1.675781082	0.357955603	4.681533316	2.85E-06	13.07853062	2.793642539	intergenic	MIR2113;PNKY	dist=700621;dist=104901		POU3F2
3M	rs141326851	6	134511989	A	C	421	0.01662708	14	2.616968305	0.552323641	4.738106631	2.16E-06	8.578496884	1.810532677	intergenic	LINC01010;LOC101928304	dist=7969;dist=13329		SGK1;ALDH8A1
3M	rs146048121	6	141877818	G	A	421	0.0023753	3	1.082392071	0.232511623	4.655217038	3.24E-06	20.02143793	4.300860255	intergenic	MIR4465;NMBR	dist=1193935;dist=196666		
3M	rs142106992	6	141948748	C	A	421	0.00356295	4	1.689946394	0.272044449	6.212023076	5.23E-10	22.83458862	3.675869896	intergenic	MIR4465;NMBR	dist=1264865;dist=125736		
3M	rs72983831	6	141985963	T	G	421	0.00593824	6	1.556807425	0.31516421	4.939670734	7.83E-07	15.67332386	3.17294911	intergenic	MIR4465;NMBR	dist=1302080;dist=88521		
3M	rs72986533	6	142290121	T	C	421	0.00831354	8	2.07386378	0.395157536	5.248194939	1.54E-07	13.28127256	2.530636288	intergenic	VTA1;ADGRG6	dist=65437;dist=11798	VTA1;ADGRG6	
3M	rs73586304	6	142518288	C	T	421	0.00356295	3	1.14329832	0.250357885	4.566655922	4.96E-06	18.24051164	3.99428202	intergenic	ADGRG6;LOC153910	dist=72022;dist=8167		
12M	rs118171627	6	143294753	C	A	439	0.00341686	3	1.522870764	0.325892127	4.672928974	2.97E-06	14.33888267	3.068500023	intronic	AIG1	.	AIG1	
12M	rs118171627	6	143294753	C	A	439	0.00341686	3	1.522870764	0.325892127	4.672928974	2.97E-06	14.33888267	3.068500023	intronic	AIG1	.		
12M	rs118171627	6	143294753	C	A	439	0.00341686	3	1.522870764	0.325892127	4.672928974	2.97E-06	14.33888267	3.068500023	intronic	AIG1	.		
12M	rs118171627	6	143294753	C	A	439	0.00341686	3	1.522870764	0.325892127	4.672928974	2.97E-06	14.33888267	3.068500023	intronic	AIG1	.		
12M	rs4896997	6	148362788	C	T	439	0.00683371	6	2.511111457	0.527345105	4.761799124	1.92E-06	9.029758847	1.896291425	intronic	SASH1	.	SASH1	
12M	rs4131286	6	148367827	G	T	439	0.00683371	6	2.510967274	0.527374461	4.761260659	1.92E-06	9.028235175	1.896185868	intronic	SASH1	.		
12M	rs17078283	6	148375784	C	T	439	0.00683371	6	2.511039365	0.527359738	4.761530287	1.92E-06	9.028998503	1.896238805	intronic	SASH1	.		
12M	rs56821264	6	148382765	C	T	439	0.00683371	6	2.51782977	0.523428582	4.810264197	1.51E-06	9.18991503	1.910480309	intronic	SASH1	.		
12M	rs917335559	6	148382765	C	T	439	0.00683371	6	2.51782977	0.523428582	4.810264197	1.51E-06	9.18991503	1.910480309	intronic	SASH1	.		
12M	rs181832855	6	151727964	C	T	439	0.00341686	3	1.6160007	0.338696272	4.771238528	1.83E-06	14.0870713	2.95249781	intronic	ESR1	.	ESR1	
3M	rs148532212	6	165086368	T	C	421	0.00356295	3	1.406500453	0.26958124	5.217352852	1.81E-07	19.35354569	3.709456929	intergenic	MEAT6;C6orf118	dist=264305;dist=193296	C6orf118	
3M	rs117498042	6	165100792	C	T	421	0.00356295	3	1.405430664	0.269604623	5.212932361	1.86E-07	19.33547097	3.70913521	intergenic	MEAT6;C6orf118	dist=278729;dist=178872		
3M	rs148153037	6	167087898	G	A	421	0.00831354	8	2.445317826	0.413004518	5.920801636	3.20E-09	14.33592462	2.421281019	intergenic	CEP43;CCR6	dist=35180;dist=23909	CCR6	
3M	rs184487573	6	167099983	A	G	421	0.00712589	7	2.008477086	0.385701261	5.207338654	1.92E-07	13.50096351	2.592680141	intergenic	CEP43;CCR6	dist=47265;dist=11824		
3M	rs17776100	7	6386848	G	A	421	0.02969121	25	3.798893519	0.756560221	5.021270496	5.13E-07	6.63697397	1.321771845	intronic	RAC1	.	RAC1	
3M	rs187978759	7	11672218	G	A	421	0.00356295	3	1.07420489	0.229159549	4.687585114	2.76E-06	20.4555522	4.3637719	intronic	THSD7A	.	THSD7A	
3M	rs117166500	7	17013154	G	T	421	0.00712589	7	1.813506071	0.380670761	4.763975219	1.90E-06	12.51468647	2.626941975	intergenic	AGR3;AHR	dist=131171;dist=285498	AGR3;AHR	
3M	rs75689761	7	18366950	C	T	421	0.00831354	7	2.0345491	0.414387092	4.909779146	9.12E-07	11.84829172	2.413202584	intronic	HDAC9	.	HDAC9	
3M	rs77346868	7	18366976	A	G	421	0.00950119	8	2.148608551	0.441537806	4.866193838	1.14E-06	11.02101286	2.264811725	intronic	HDAC9	.		
3M	rs78225611	7	18367841	A	C	421	0.00950119	8	2.147570798	0.441262518	4.866877908	1.13E-06	11.02943875	2.266224663	intronic	HDAC9	.		
3M	rs77300464	7	18369138	A	G	421	0.00712589	6	2.141507953	0.382123995	5.604222657	2.09E-08	14.66597943	2.616951597	intronic	HDAC9	.		
3M	rs79602997	7	18370627	G	A	421	0.00831354	7	2.026922666	0.413391185	4.903158896	9.43E-07	11.86082112	2.419016265	intronic	HDAC9	.		
3M	rs75773869	7	18371222	G	T	421	0.00831354	7	2.02750066	0.413389996	4.904571175	9.36E-07	11.86427156	2.41902322	intronic	HDAC9	.		
3M	rs75606013	7	18374990	G	A	421	0.00712589	6	2.140929626	0.382127995	5.602650559	2.11E-08	14.66171186	2.616924205	intronic	HDAC9	.		
3M	rs61434999	7	18378728	A	G	421	0.00831354	7	2.033933624	0.414449316	4.907556958	9.22E-07	11.84115106	2.412840272	intronic	HDAC9	.		
3M	rs78907958	7	18385394	T	G	421	0.00831354	7	2.090002735	0.415774551	5.026769271	4.99E-07	12.09013217	2.405149614	intronic	HDAC9	.		
3M	rs76526501	7	18391487	G	A	421	0.00712589	6	2.233180137	0.387925975	5.75671721	8.58E-09	14.83973124	2.577811398	intronic	HDAC9	.		
3M	rs74455595	7	18392161	A	G	421	0.00712589	6	2.233180137	0.387925975	5.75671721	8.58E-09	14.83973124	2.577811398	intronic	HDAC9	.		
3M	rs79182806	7	18394204	T	C	421	0.00831354	7	2.11859485	0.418485528	5.062528352	4.14E-07	12.09726027	2.389568893	intronic	HDAC9	.		
3M	rs10279777	7	18401966	G	A	421	0.00950119	8	2.476234721	0.438670193	5.644866606	1.65E-08	12.86813351	2.279616935	intronic	HDAC9	.		
3M	rs77867199	7	18402652	G	T	421	0.01068884	9	2.377239679	0.468684022	5.072158572	3.93E-07	10.82212821	2.13363365	intronic	HDAC9	.		
3M	rs80156375	7	18403592	A	C	421	0.00950119	8	2.260074467	0.442534058	5.107119835	3.27E-07	11.5406255	2.25971308	intronic	HDAC9	.		
3M	rs17169602	7	18407118	G	A	421	0.00950119	8	2.49198241	0.442489656	5.631730316	1.78E-08	12.72737168	2.259939835	intronic	HDAC9	.		
3M	rs10486295	7	18407184	G	A	421	0.00950119	8	2.488096085	0.441539202	5.635051363	1.75E-08	12.76229003	2.264804562	intronic	HDAC9	.		
3M	rs75090694	7	18407813	A	G	421	0.00831354	7	2.37298061	0.41420374	5.729017827	1.01E-08	13.83140053	2.414270813	intronic	HDAC9	.		
3M	rs55844051	7	23320744	T	C	421	0.00356295	3	1.232383167	0.25888714	4.760310483	1.93E-06	18.3875896	3.862687038	intronic	IGF2BP3	.	IGF2BP3	
3M	rs62447184	7	36534898	G	A	421	0.04275534	43	4.329544629	0.918817438	4.712083651	2.45E-06	5.128422094	1.088355486	intronic	AOAH	.	AOAH	
3M	rs574076561	7	49505151	A	G	421	0.00356295	3	1.086373837	0.235169216	4.619541003	3.85E-06	19.64347663	4.25225723	intergenic	CDC14C;VWC2	dist=577697;dist=268487	VWC2	
12M	rs564332768	7	64988831	A	C	439	0.00797267	6	2.356215761	0.507434013	4.643393431	3.43E-06	9.150733523	1.970699589	intronic	ERV3-1-ZNF117;ZNF117	.	ERV3-1;ZNF117	
12M	rs533568586	7	66745708	G	A	439	0.00797267	7	2.458442137	0.536776292	4.580012523	4.65E-06	8.532441903	1.862973488	intronic	RABGEF1	.	RABGEF1	
12M	rs189433222	7	67166631	C	T	439	0.00683371	7	2.388728197	0.523042224	4.566989217	4.95E-06	8.73158802	1.911891534	intronic	TYW1	.	TYW1	
12M	rs566394355	7	67352900	T	G	439	0.00683371	6	2.423461328	0.526255379	4.605105094	4.12E-06	8.750704076	1.900218105	intergenic	LINC01372;LOC102723427	dist=12875;dist=667353		
12M	rs577438374	7	67418626	C	T	439	0.00683371	6	2.39527184	0.515335444	4.647985827	3.35E-06	9.019340475	1.940483644	intergenic	LINC01372;LOC102723427	dist=78601;dist=601627		
12M	rs548247109	7	67561468	G	A	439	0.00683371	6	2.378475952	0.500107071	4.755933457	1.98E-06	9.509830457	1.999571807	intergenic	LINC01372;LOC102723427	dist=221443;dist=458785		
3M	rs111391231	7	89867731	T	C	421	0.01425178	12	2.546596314	0.553340001	4.602227036	4.18E-06	8.317177547	1.807207138	intergenic	ZNF804B;STEAP2-AS1	dist=529203;dist=14622	ZNF804B;STEAP2	
3M	rs111900874	7	89887355	G	A	421	0.01425178	12	2.536039096	0.548671755	4.622142612	3.80E-06	8.424240128	1.822583342	ncRNA_intronic	STEAP2-AS1	.		
3M	rs181259864	7	97859511	C	A	421	0.00356295	3	1.263697226	0.271559968	4.653473908	3.26E-06	17.13608209	3.682427888	ncRNA_intronic	CZ1P-ASNS	.	CZ1P-ASNS	ASNS;TAC1
3M	rs192750513	7	97948518	A	G	421	0.00356295	3	1.238714279	0.26844613	4.614386809	3.94E-06	17.18924692	3.725142178	ncRNA_intronic	CZ1P-ASNS	.		
3M	rs539713344	7	100877165	G	A	421	0.0023753	3	1.125805228	0.236990667	4.750420101	2.03E-06	20.04475604	4.219575451	intronic	SRRT	.	SRRT	
3M	rs188028357	7	101025144	C	T	421	0.0023753	3	1.150125852	0.229467369	5.012154263	5.38E-07	21.84255775	4.357918094	intronic	MUC17	.		MUC12;TRIM56
12M	rs181182636	7	116014504	A	G	439	0.00341686	3	1.592950272	0.347387686	4.585511625	4.53E-06	13.19998323	2.878628233	intronic	TFEC	.	TFEC	
12M	rs182211730	7	116363561	G	A	439	0.00455581	4	1.988619676	0.418066319	4.756708646	1.97E-06	11.37788056	2.391964991	intergenic	LOC102724434;CAV2	dist=76834;dist=136177	CAV2	
12M	rs143281973	7	116367942	C	T	439	0.00455581	4	2.006625392	0.427483854	4.694037855	2.68E-06	10.98062023	2.339269638	intergenic	LOC102724434;CAV2	dist=81215;dist=131796		
12M	rs138873576	7	116385305	G	T	439	0.00455581	4	1.991831401	0.430896074	4.622533181	3.79E-06	10.72772174	2.320745209	intergenic	LOC102724434;CAV2	dist=98578;dist=114433		
12M	rs190354334	7	116435792	G	A	439	0.00455581	5	2.00243762	0.432429385	4.630669627	3.64E-06	10.70849897	2.312516295	intergenic	LOC102724434;CAV2	dist=149065;dist=63946		
12M	rs186893139	7	116456916	C	T	439	0.00455581	4	1.880403456	0.406387212	4.627122608	3.71E-06	11.38599462	2.460707351	intergenic	LOC102724434;CAV2	dist=170189;dist=42822		
12M	rs191903483	7	116474429	A	G	439	0.00455581	5	1.937757245	0.423414829	4.576498295	4.73E-06	10.80854514	2.361750063	intergenic	LOC102724434;CAV2	dist=187702;dist=25309		
12M	rs558784715	7	117403240	A	G	439	0.00341686	3	1.805721393	0.377457708	4.783903883	1.72E-06	12.67401298	2.649303432	intronic	ASZ1			
12M	rs576047962	7	117468967	A	G	439	0.00341686	3	1.817321889	0.377176493	4.818226812	1.45E-06	12.7744621	2.651278697	intergenic	ASZ1;CFTR	dist=41474;dist=11058	CFTR	
12M	rs529110230	7	117535853	T	C	439	0.00341686	3	1.806947008	0.378567349	4.773119008	1.81E-06	12.60837477	2.641537902	intronic	CFTR	.		
12M	rs78282451	7	117553939	C	A	439	0.00455581	4	2.053353534	0.436106063	4.708381076	2.50E-06	10.79641279	2.293020173	ncRNA_intronic	CFTR-AS1	.		
12M	rs142215699	7	117559820	T	C	439	0.00341686	3	1.830346943	0.377150347	4.853096273	1.22E-06	12.86780275	2.651462495	ncRNA_intronic	CFTR-AS1	.		
12M	rs75862363	7	117570346	G	A	439	0.00455581	4	2.053944777	0.436108133	4.709714457	2.48E-06	10.79941901	2.293009291	intronic	CFTR	.		
12M	rs77846488	7	117571897	T	C	439	0.00455581	4	2.054832011	0.436103169	4.71180252	2.46E-06	10.80432993	2.293035389	intronic	CFTR	.		
12M	rs142721557	7	117578274	A	C	439	0.00341686	3	1.830638975	0.377148582	4.853893297	1.21E-06	12.86997625	2.651474902	intronic	CFTR	.		
12M	rs201355675	7	117585727	G	A	439	0.00341686	3	1.830359186	0.377152899	4.853095897	1.22E-06	12.86771469	2.651444555	intronic	CFTR	.		
12M	rs188993522	7	117634677	T	C	439	0.00341686	3	1.746488875	0.364012714	4.797878776	1.60E-06	13.18052527	2.747156793	intronic	CFTR	.		
12M	rs190601781	7	132751214	G	A	439	0.00341686	3	1.55039666	0.338020811	4.586689956	4.50E-06	13.56925315	2.958397728	intergenic	FLJ40288;LOC100506937	dist=22446;dist=7756		PLXNA4;CHCHD3
3M	rs536023430	7	147167980	T	C	421	0.00356295	3	1.137129093	0.242081012	4.697308079	2.64E-06	19.40386834	4.130848564	intronic	CNTNAP2	.	CNTNAP2	
3M	rs187384541	8	1684075	A	G	421	0.03562945	26	3.153891836	0.676696813	4.660716257	3.15E-06	6.887451174	1.477766677	intronic	DLGAP2	.	DLGAP2	
3M	rs575473987	8	5719972	C	T	421	0.00356295	3	1.471553256	0.257316966	5.718834929	1.07E-08	22.22486534	3.886257536	intergenic	CSMD1;LOC100287015	dist=725058;dist=683583	CSMD1	
3M	rs139062456	8	13394482	C	T	421	0.01781473	15	2.721124769	0.589318868	4.617406495	3.89E-06	7.835158086	1.696874229	intronic	DLC1	.	DLC1	
3M	rs185874707	8	18173165	C	T	421	0.01187648	10	2.405517016	0.513034555	4.688801158	2.75E-06	9.139347655	1.949186444	intronic	NAT1	.	NAT1	
3M	rs140797780	8	22230279	C	T	421	0.00356295	3	1.415632732	0.270894217	5.225776871	1.73E-07	19.29083956	3.691477848	intronic	PHYHIP	.	PHYHIP	
3M	rs147601511	8	22464806	A	G	421	0.00475059	4	1.494160188	0.301407236	4.957280417	7.15E-07	16.44711814	3.317770381	intronic	PPP3CC	.	PPP3CC	
3M	rs188415494	8	25755782	C	T	421	0.00356295	3	1.162251387	0.250533229	4.639110717	3.50E-06	18.51694779	3.991486498	intergenic	CDCA2;EBF2	dist=247865;dist=85943	CDCA2;EBF2	
12M	rs111896192	8	37846591	C	T	439	0.00341686	4	1.834661883	0.379554059	4.833730106	1.34E-06	12.7352876	2.634670807	intronic	BRF2	.	BRF2	
3M	rs9643828	8	54616513	C	T	421	0.67695962	279	-11.04343772	2.134612444	-5.173509483	2.30E-07	-2.423629403	0.468469114	intronic	RP1	.		SOX17;XKR4
3M	rs423841	8	54643509	G	A	421	0.66270784	291	-10.22664529	2.181175145	-4.688594272	2.75E-06	-2.149572575	0.458468456	intronic	RP1	.		

3M	rs433324	8	54652049	A	G	421	0.66627078	287	-10.71178821	2.182726874	-4.907525693	9.22E-07	-2.248346209	0.458142524	intronic	RP1	.		
3M	rs369623	8	54659380	A	C	421	0.66627078	287	-10.8407396	2.201888659	-4.923382276	8.51E-07	-2.235981486	0.45415557	intronic	RP1	.		
3M	rs446222	8	54662400	G	A	421	0.66627078	288	-10.90656484	2.202740432	-4.951361803	7.37E-07	-2.247819003	0.453979954	intronic	RP1	.		
3M	rs432393	8	54667738	C	T	421	0.66270784	291	-10.75654812	2.217474543	-4.850810194	1.23E-06	-2.187538166	0.450963464	intronic	RP1	.		
3M	rs3098298	8	54670278	C	T	421	0.66270784	291	-10.75741494	2.217551047	-4.851033737	1.23E-06	-2.187563503	0.450947906	intronic	RP1	.		
3M	rs367179	8	54675056	T	C	421	0.66270784	291	-10.75741494	2.217551047	-4.851033737	1.23E-06	-2.187563503	0.450947906	intronic	RP1	.		
3M	rs382476	8	54678415	G	A	421	0.66627078	288	-10.90475865	2.202374434	-4.951364528	7.37E-07	-2.24819379	0.454055398	intronic	RP1	.		
3M	rs384543	8	54679049	G	A	421	0.66627078	288	-10.90475865	2.202374434	-4.951364528	7.37E-07	-2.24819379	0.454055398	intronic	RP1	.		
3M	rs405226	8	54679776	A	G	421	0.66270784	291	-10.75682372	2.217202771	-4.851529081	1.23E-06	-2.188130533	0.45101874	intronic	RP1	.		
3M	rs384127	8	54684929	G	A	421	0.66627078	288	-10.90492469	2.202409773	-4.951360471	7.37E-07	-2.248155875	0.454048112	intronic	RP1	.		
3M	rs858397	8	54702130	A	G	421	0.33135392	278	11.01774054	2.2226993	4.956919066	7.16E-07	2.230134803	0.449903412	intronic	RP1	.		
3M	rs2375537	8	54706948	C	T	421	0.33254157	280	11.08575521	2.225246111	4.981810848	6.30E-07	2.238768477	0.449388495	intronic	RP1	.		
3M	rs720372	8	54716077	G	A	421	0.34679335	292	10.41667706	2.227853301	4.675656629	2.93E-06	2.098727338	0.448862589	intronic	RP1	.		
3M	rs1437781	8	54717292	T	C	421	0.33254157	280	11.08371192	2.225151228	4.981105007	6.32E-07	2.23854673	0.449407657	intronic	RP1	.		
3M	rs1595406	8	54718055	A	G	421	0.3456057	291	10.45008066	2.230645943	4.684777831	2.80E-06	2.100188892	0.448300638	intronic	RP1	.		
3M	rs1437782	8	54720202	C	T	421	0.33016627	278	11.10161994	2.217354849	5.006695229	5.54E-07	2.2579585	0.450987807	exonic	RP1	.		
3M	rs10105693	8	54727912	C	T	421	0.32897862	276	11.34015363	2.223938019	5.099132049	3.41E-07	2.292839101	0.449652819	intronic	RP1	.		
3M	rs2375536	8	54728162	T	C	421	0.347981	292	10.56754692	2.229301935	4.740294148	2.13E-06	2.126358065	0.448570911	intronic	RP1	.		
3M	rs4737674	8	54749094	C	A	421	0.33016627	277	11.42523599	2.226081333	5.132443196	2.86E-07	2.305595541	0.449219885	intronic	RP1	.		
3M	rs11987234	8	54757269	A	G	421	0.32897862	276	11.39664661	2.227939022	5.115331478	3.13E-07	2.295992587	0.448845319	intronic	RP1	.		
3M	rs13277510	8	54761589	G	A	421	0.33016627	277	11.42815338	2.22667847	5.132377007	2.86E-07	2.304947516	0.449099416	intronic	RP1	.		
3M	rs12548593	8	54762057	G	T	421	0.33254157	279	11.39766972	2.234516943	5.100730946	3.38E-07	2.282699606	0.447524018	intronic	RP1	.		
3M	rs1812506	8	54763541	A	G	421	0.3456057	291	10.64127813	2.239050947	4.752584188	2.01E-06	2.122588678	0.446617796	intronic	RP1	.		
3M	rs16920698	8	54765874	G	A	421	0.33016627	277	11.42894943	2.226765953	5.132532863	2.86E-07	2.304926953	0.449081772	intronic	RP1	.		
3M	rs1561297	8	54765978	A	C	421	0.33254157	279	11.40464743	2.234979191	5.102798037	3.35E-07	2.28315237	0.447431459	intronic	RP1	.		
3M	rs4737676	8	54766986	G	A	421	0.33016627	277	11.42891023	2.226765217	5.132516953	2.86E-07	2.304920569	0.44908192	intronic	RP1	.		
3M	rs2083123	8	54767758	C	T	421	0.33254157	279	11.40261082	2.236761629	5.097821184	3.44E-07	2.279107938	0.447074908	intronic	RP1	.		
3M	rs983248	8	54768232	C	T	421	0.33016627	277	11.43612477	2.228588003	5.131556283	2.87E-07	2.302604284	0.448714611	intronic	RP1	.		
3M	rs1391463	8	54769316	T	G	421	0.33016627	277	11.43612548	2.228595636	5.131539026	2.87E-07	2.302588654	0.448713075	intronic	RP1	.		
3M	rs10958428	8	54773081	A	G	421	0.33372922	280	11.27671394	2.243728439	5.025881807	5.01E-07	2.239968848	0.445686734	intronic	RP1	.		
3M	rs13278605	8	54775611	C	T	421	0.32897862	277	11.43432709	2.242496155	5.09892829	3.42E-07	2.273773481	0.445931645	intronic	RP1	.		
3M	rs13276543	8	54775614	G	T	421	0.32897862	276	11.41118163	2.230992831	5.114844597	3.14E-07	2.29263157	0.448230934	intronic	RP1	.		
3M	rs7822082	8	54777660	T	C	421	0.33016627	277	11.70263936	2.228199285	5.252061357	1.50E-07	2.357087802	0.448792891	intronic	RP1	.		
3M	rs4737201	8	54778898	C	T	421	0.33016627	277	11.43417402	2.228701352	5.130420012	2.89E-07	2.301977341	0.44869179	intronic	RP1	.		
3M	rs7843693	8	54779552	G	A	421	0.39786223	334	10.46760966	2.288570748	4.573863258	4.79E-06	1.998567561	0.436953938	intronic	RP1	.		
3M	rs1396896	8	54782750	A	G	421	0.39786223	334	10.46979793	2.288829589	4.574302074	4.78E-06	1.998533266	0.436904523	intronic	RP1	.		
3M	rs2375219	8	54785735	C	T	421	0.39311164	330	10.58980707	2.26895928	4.667253025	3.05E-06	2.057001668	0.440730695	intronic	RP1	.		
3M	rs1391462	8	54787221	C	A	421	0.39786223	334	10.46683006	2.288537601	4.573588854	4.79E-06	1.998476605	0.436960267	intronic	RP1	.		
3M	rs12678939	8	54792461	A	G	421	0.39429929	331	10.32905196	2.261417548	4.567512076	4.94E-06	2.019756183	0.442200513	intronic	RP1	.		
3M	rs1498183	8	54804345	C	T	421	0.39429929	332	10.33728121	2.259624491	4.574778355	4.77E-06	2.024574602	0.442551408	intronic	RP1	.		
3M	rs117816016	8	102739034	C	T	421	0.00356295	3	1.090578449	0.234213862	4.656336053	3.22E-06	19.88070227	4.269602116	intergenic	LOC101927245;GASAL1	dist=52311;dist=67788		KLF10;AZIN1
3M	rs567383525	8	114017265	C	T	421	0.00356295	3	1.282541719	0.268838431	4.770678479	1.84E-06	17.74552267	3.719706274	intergenic	CSMD3;TRPS1	dist=580326;dist=1391230	CSMD3;TRPS1	
3M	rs545550279	8	114540200	G	T	421	0.00356295	3	1.091458604	0.238542228	4.57553622	4.75E-06	19.18124208	4.192129875	intergenic	CSMD3;TRPS1	dist=1103261;dist=868295		
3M	rs536803366	8	121989172	T	C	421	0.0023753	3	1.194921552	0.249406377	4.791062547	1.66E-06	19.20986385	4.009520573	intergenic	HAS2-AS1;SMILR	dist=343847;dist=425155	HAS2	
12M	rs139457011	8	124378581	C	T	439	0.00341686	3	1.698245565	0.326123918	5.207362812	1.92E-07	15.96743605	3.0663191	intergenic	TMEM65;TRMT12	dist=5882;dist=72239	TMEM65;TRMT12	
3M	rs532513136	8	134801505	C	A	421	0.00356295	3	1.227599638	0.248065954	4.948682463	7.47E-07	19.94905941	4.031185989	upstream	MIR30B	dist=898		ZFAT
3M	rs532730683	9	1784492	G	T	421	0.00356295	3	1.275657724	0.260996603	4.887641107	1.02E-06	18.72683804	3.831467497	intergenic	DMRT2;SMARCA2	dist=726938;dist=230855	DMRT2;SMARCA2	
3M	rs540065886	9	2770228	T	C	421	0.00356295	3	1.13736568	0.23877586	4.763319367	1.90E-06	19.94891509	4.188028045	intergenic	KCNV2;PUM3	dist=40191;dist=33927	PUM3	
3M	rs543844012	9	30107025	C	T	421	0.00356295	3	1.164274175	0.253510467	4.592607905	4.38E-06	18.11604845	3.944610301	intergenic	LINGO2;LINC01242	dist=893424;dist=281910	LINGO2	
12M	rs79914278	9	37658199	C	T	439	0.05125285	45	7.463015515	1.54104852	4.842816705	1.28E-06	3.142546548	0.648908835	intronic	FRMPD1	.	FRMPD1	
12M	rs10973466	9	37661113	G	A	439	0.05125285	45	7.461346315	1.537487847	4.852946531	1.22E-06	3.156412936	0.650411645	intronic	FRMPD1	.		
12M	rs1442156642	9	37664627	G	A	439	0.15261959	134	10.58008025	2.239682708	4.723919246	2.31E-06	2.109191284	0.446491816	intronic	FRMPD1	.		
12M	rs6476657	9	37664627	G	A	439	0.15261959	134	10.58008025	2.239682708	4.723919246	2.31E-06	2.109191284	0.446491816	intronic	FRMPD1	.		
12M	rs2057644	9	37665983	G	A	439	0.15261959	134	10.57853162	2.239752645	4.723080313	2.32E-06	2.108750858	0.446477874	intronic	FRMPD1	.		
12M	rs2057643	9	37666013	T	C	439	0.15261959	134	10.57853162	2.239752645	4.723080313	2.32E-06	2.108750858	0.446477874	intronic	FRMPD1	.		
12M	rs10973475	9	37666364	C	T	439	0.05125285	45	7.456175961	1.536478706	4.852768822	1.22E-06	3.158370371	0.650838828	intronic	FRMPD1	.		
12M	rs10814594	9	37666670	T	C	439	0.15261959	134	10.57879091	2.239737923	4.723227126	2.32E-06	2.108830269	0.446480809	intronic	FRMPD1	.		
12M	rs1125576	9	37667822	T	C	439	0.15261959	134	10.57668904	2.239823833	4.722107554	2.33E-06	2.108249535	0.446463684	intronic	FRMPD1	.		
12M	rs60006744	9	37672694	G	T	439	0.05125285	45	7.444177374	1.53385479	4.853247792	1.21E-06	3.164085559	0.651952197	intronic	FRMPD1	.		
12M	rs2148140	9	37673842	C	T	439	0.05125285	45	7.444177374	1.53385479	4.853247792	1.21E-06	3.164085559	0.651952197	intronic	FRMPD1	.		
12M	rs113399724	9	37674360	C	T	439	0.05125285	45	7.444177374	1.53385479	4.853247792	1.21E-06	3.164085559	0.651952197	intronic	FRMPD1	.		
12M	rs12350891	9	79989706	G	A	439	0.00455581	4	1.919901525	0.416077273	4.614290779	3.94E-06	11.08998515	2.403399716	ncRNA_intronic	LINC01507			
3M	rs148556485	9	81408911	A	C	421	0.0023753	3	1.17470554	0.234401563	5.011508991	5.40E-07	21.38001525	4.266183157	intergenic	LINC01507;TLE1	dist=1374356;dist=174772	TLE1	
3M	rs140782222	9	81413979	T	C	421	0.0023753	3	1.1680569	0.231519821	5.045170192	4.53E-07	21.79152597	4.31928461	intergenic	LINC01507;TLE1	dist=1379424;dist=169704		
3M	rs188034471	9	83322799	G	A	421	0.00475059	4	1.487832555	0.317535808	4.68555835	2.79E-06	14.75599992	3.149251127	intronic	FRMD3	.	FRMD3	
3M	rs190294315	9	83330550	C	T	421	0.00593824	5	1.754632626	0.347069598	5.055564181	4.29E-07	14.56642764	2.881266486	intronic	FRMD3			
3M	rs545690161	9	90268417	G	A	421	0.00593824	6	1.900812423	0.332979511	5.708496652	1.14E-08	17.14368742	3.003187786	intergenic	MIR4290HG;LINC01508	dist=226918;dist=32479		
3M	rs565682685	9	90460046	T	C	421	0.00475059	4	1.368085345	0.268772597	5.090122138	3.58E-07	18.93839699	3.720617399	intergenic	LINC01508;LINC01501	dist=26557;dist=2386		
3M	rs183737367	9	90567765	T	C	421	0.00356295	3	1.295690386	0.235587115	5.499835526	3.80E-08	23.34523057	4.244714311	ncRNA_intronic	LINC01501	.	DIRAS2;SYK	
3M	rs187213609	9	90653183	C	T	421	0.00356295	3	1.269834136	0.233965931	5.427431802	5.72E-08	23.19753039	4.274126556	intergenic	DIRAS2;SYK	dist=10359;dist=148417		
3M	rs150027952	9	100623134	A	G	421	0.0023753	3	1.072594942	0.221501288	4.842386925	1.28E-06	21.86166485	4.514646431	intergenic	CAVIN4;PLPPR1	dist=34747;dist=405593	PLPPR1	
3M	rs146207930	9	126280057	A	G	421	0.00712589	6	1.87537986	0.397610416	4.716626584	2.40E-06	11.86243214	2.515024654	intergenic	LOC101929116;MVB12B	dist=4142;dist=46772	MVB12B	
3M	rs78296164	9	132391328	C	T	421	0.00831354	7	2.121725249	0.427501046	4.963087859	6.94E-07	11.60953385	2.339175566	intronic	TTF1	.	TTF1	
3M	rs77871739	9	135660463	G	A	421	0.00475059	4	1.439297375	0.309017583	4.657655271	3.20E-06	15.07246035	3.236061811	intergenic	GLT6D1;LCN9	dist=20923;dist=2859		MRPS2;CAMSAP1
12M	rs76294395	9	135780517	G	A	439	0.00569476	5	2.250462353	0.487505863	4.616277515	3.91E-06	9.469173331	2.051257382	intronic	KCNT1	.		
12M	rs62582460	9	138095976	T	C	439	0.05808656	51	6.819364418	1.458397024	4.675931387	2.93E-06	3.206212925	0.68568434	intronic	CACNA1B	.	CACNA1B	
3M	rs118040657	10	3430654	C	T	421	0.00831354	8	1.732520372	0.378165204	4.581384943	4.62E-06	12.11477125	2.644346939	ncRNA_intronic	LOC105376360	.		PITRM1;KLF6
12M	rs187982129	10	10793335	G	T	439	0.00455581	4	2.104360473	0.43943772	4.788757028	1.68E-06	10.89746466	2.275635325	ncRNA_intronic	SFTA1P	.	SFTA1P	
12M	rs117285875	10	10833324	G	T	439	0.00455581	4	2.102691811	0.434748428	4.836571393	1.32E-06	11.1249888	2.30018083	intronic	CELF2	.	CELF2	
3M	rs184425183	10	13415520	A	G	421	0.00356295	3	1.590271029	0.264865828	6.004062666	1.92E-09	22.66831744	3.775496477	intergenic	SEPHS1;BEND7	dist=67222;dist=22961	SEPHS1;BEND7	
3M	rs184458518	10	13429195	T	G	421	0.00356295	3	1.603233248	0.265164814	6.046176429	1.48E-09	22.80157891	3.771239424	intergenic	SEPHS1;BEND7	dist=80897;dist=9286		
3M	rs117998251	10	13455976	C	T	421	0.00356295	3	1.610926128	0.264914355	6.080931816	1.19E-09	22.95433112	3.774804885	intronic	BEND7	.		
3M	rs117025967	10	19918123	C	A	421	0.01306413	11	2.602875018	0.518823163	5.016882829	5.25E-07	9.669735635	1.927439002	intronic	PLXDC2	.		MALRD1;NEBL
3M	rs529011661	10	20083387	G	A	421	0.00475059	4	1.425272643	0.301883703	4.721263948	2.34E-06	15.63934688	3.312533901	intronic	PLXDC2	.		
12M	rs142956968	10	23201236	C	T	439	0.01594533	14	3.953608313	0.770445563	5.131586839	2.87E-07	6.660544343	1.297950235	downstream	C10orf67	dist=680		MSRB2;OTUD1
12M	rs145764464	10	23312918	G	A	439	0.01708428	15	4.204896484	0.829087356	5.071717058	3.94E-07	6.11722881	1.20614552	intronic	C10orf67	.		
3M	rs12266995	10	24563854	T	C	421	0.03087886	26	3.568935699	0.773323681	4.615060663	3.93E-06	5.967825345	1.293119589	intergenic	KIAA1217;ARHGAP21	dist=16006;dist=19760	ARHGAP21	
12M	rs74384278	10	44647124	T	C	439	0.00911162	8	2.588932233	0.559803403	4.624716857	3.75E-06	8.261323236	1.786341411	intergenic	CXCL12;TMEM72-AS1	dist=262027;dist=163900	CXCL12	
3M	rs138249376	10	61756071	T	G	421	0.00475059	4	1.271516185	0.278189815	4.57067843	4.86E-06	16.43007109	3.594667912	intronic	CABCOCO1	.	TMEM26;ARID5B	

3M	rs140277951	10	80599344	G	A	421	0.00831354	6	1.967344821	0.378309358	5.200359916	1.99E-07	13.74631584	2.643339318	intronic	SH2D4B	.	TSPAN14;NRG3	
3M	rs566018180	10	84995296	C	T	421	0.00356295	3	1.136146605	0.247392568	4.592484792	4.38E-06	18.56355199	4.04215862	intergenic	CCSER2;LINC01519	dist=476775;dist=198125	CCSER2	
3M	rs140706881	10	94457778	G	A	421	0.00475059	4	1.226743807	0.254265992	4.824647601	1.40E-06	18.97480496	3.932889306	intronic	TBC1D12	.	TBC1D12	
3M	rs117913371	10	101158729	G	A	421	0.02494062	21	3.968654489	0.710300363	5.587290525	2.31E-08	7.866095548	1.407855116	intergenic	TLX1NB;LINC01514	dist=17463;dist=17593		KAZALD1;BTRC
3M	rs75334617	10	101196395	G	A	421	0.03800475	32	4.196585386	0.847261284	4.953118316	7.30E-07	5.846034048	1.180273451	intergenic	LINC01514;LBX1	dist=2248;dist=30581		
3M	rs752259256	10	103165562	T	C	421	0.00356295	3	1.029573618	0.224511445	4.58584023	4.52E-06	20.42586391	4.454115907	intronic	NT5C2	.	NT5C2	
12M	rs147944608	10	108663951	C	T	439	0.01252847	11	3.51284077	0.711541711	4.936942858	7.94E-07	6.938374489	1.405398986	intergenic	LINC01435;XPNPEP1	dist=594658;dist=1200815	XPNPEP1	
3M	rs180828621	10	122773893	G	A	421	0.00712589	6	1.931459198	0.357196912	5.407267342	6.40E-08	15.13805735	2.799576273	ncRNA_intronic	DMBT1L1	.		CUZD1;FAM24B
3M	rs147393020	10	123019758	A	G	421	0.00593824	5	1.619245544	0.337859644	4.792657467	1.65E-06	14.1853505	2.959808958	intronic	ACADSB	.	ACADSB	
3M	rs193093906	10	125016920	G	A	421	0.00950119	9	2.062968829	0.442790343	4.659019469	3.18E-06	10.52195366	2.25840517	intronic	CTBP2	.	CTBP2	
12M	rs148815783	11	1013992	C	T	439	0.00797267	8	2.639026369	0.568648462	4.640874893	3.47E-06	8.161237049	1.758555711	exonic	MUC6	.		AP2A2;
12M	rs150788978	11	18175633	G	A	439	0.00797267	8	2.532677712	0.547949348	4.622101879	3.80E-06	8.435272154	1.824986202	intergenic	MRGPRX4;SLC25A51P4	dist=1353;dist=33505		MRGPRX3;SAA4
3M	rs151115079	11	18634194	T	C	421	0.00475059	5	1.669234911	0.305995633	5.455093905	4.89E-08	17.8273587	3.268020498	intronic	SPTY2D1	.		
3M	rs138414342	11	18657851	G	A	421	0.00475059	5	1.685610798	0.304678389	5.532426519	3.16E-08	18.15825054	3.282149429	intergenic	SPTY2D1;TMEM86A	dist=23509;dist=40928	SPTY2D1;TMEM86A	
3M	rs541653703	11	18680239	G	A	421	0.00475059	4	1.710453669	0.305582922	5.597347058	2.18E-08	18.31694986	3.272434185	intergenic	SPTY2D1;TMEM86A	dist=45897;dist=18540		
3M	rs118093638	11	18696777	C	T	421	0.00593824	5	1.681215257	0.343292823	4.897321308	9.72E-07	14.26572587	2.912965064	intergenic	SPTY2D1;TMEM86A	dist=62435;dist=2002		
12M	rs192072893	11	24171605	G	A	439	0.01138952	10	2.98365585	0.63649116	4.687662667	2.76E-06	7.364851171	1.571113729	intergenic	MIR8054;LUZP2	dist=752415;dist=325448		
12M	rs183894319	11	24243274	T	C	439	0.01252847	11	3.441856532	0.682038209	5.046427729	4.50E-07	7.399039613	1.466193516	intergenic	MIR8054;LUZP2	dist=824084;dist=253779	LUZP2	
12M	rs146347965	11	24443383	C	A	439	0.01366743	12	3.980373837	0.744795958	5.344247369	9.08E-08	7.175451632	1.342649607	intergenic	MIR8054;LUZP2	dist=1024193;dist=53670		
12M	rs186629933	11	24499372	A	G	439	0.01366743	12	3.971746912	0.745783477	5.325603253	1.01E-07	7.14095098	1.340871755	intronic	LUZP2	.		
12M	rs72874841	11	24549456	C	T	439	0.02277904	20	4.439777519	0.9297034	4.775477341	1.79E-06	5.136560051	1.075611857	intronic	LUZP2	.		
12M	rs141411318	11	24584003	C	T	439	0.02277904	20	4.434973388	0.926703454	4.78575252	1.70E-06	5.164276125	1.079093853	intronic	LUZP2	.		
12M	rs72878847	11	24599192	A	G	439	0.023918	20	4.373449314	0.932123939	4.691918242	2.71E-06	5.033577665	1.072818708	intronic	LUZP2	.		
3M	rs181812512	11	66898258	C	T	421	0.00356295	3	1.08407707	0.235726848	4.598869755	4.25E-06	19.50931683	4.242198164	intronic	PC	.	PC	
3M	rs529345909	11	67343381	A	G	421	0.00356295	3	1.116845445	0.244375672	4.570198967	4.87E-06	18.70153001	4.092060355	ncRNA_intronic	LOC100130987	.	LOC100130987	SSH3;POLD4
3M	rs544042801	11	68692775	G	A	421	0.00475059	4	1.370120555	0.293669353	4.665521075	3.08E-06	15.88698661	3.405190192	intergenic	GAL;TESMIN	dist=1600;dist=14665	GAL	
12M	rs139548692	11	82893220	C	T	439	0.00455581	6	1.861738012	0.402460972	4.625884601	3.73E-06	11.49399551	2.484712979	intronic	PRCP	.	PRCP	
3M	rs149949098	11	95366702	G	A	421	0.01662708	13	2.587327209	0.549803064	4.705916312	2.53E-06	8.559276254	1.818833079	intergenic	LOC100129203;FAM76B	dist=132298;dist=402251	FAM76B	
3M	rs74521112	11	99218416	G	T	421	0.03206651	27	4.149638813	0.814414575	5.095241344	3.48E-07	6.256323868	1.227875864	intronic	CNTN5	.	CNTN5	
3M	rs79213709	11	99222724	G	A	421	0.03206651	26	4.048879944	0.810020577	4.998490236	5.78E-07	6.17081884	1.23453654	intronic	CNTN5	.		
3M	rs112007361	11	99317649	A	C	421	0.03087886	26	4.213518738	0.814161472	5.175286336	2.28E-07	6.356584678	1.228257582	intronic	CNTN5	.		
12M	rs183749982	11	102098824	G	A	439	0.00455581	4	1.971855551	0.409991583	4.80950252	1.51E-06	11.73073478	2.439074464	intergenic	CFAP300;YAP1	dist=14264;dist=11623	YAP1	
3M	rs148781275	11	103769875	A	G	421	0.00475059	5	1.423809766	0.301038131	4.729665848	2.25E-06	15.71118528	3.321838325	intergenic	DYNC2H1;MIR4693	dist=290012;dist=80031	DYNC2H1	
3M	rs141281289	11	123822316	A	G	421	0.00593824	6	1.657109443	0.323734372	5.118731856	3.08E-07	15.81151802	3.088952198	intergenic	OR6M1;TMEM225	dist=15967;dist=60603		No gene nearby
3M	rs79539453	11	125396457	C	T	421	0.0023753	3	1.102705134	0.230127137	4.791721423	1.65E-06	20.82206158	4.345424064	intronic	PKNOX2	.	PKNOX2	
3M	rs528140343	11	125849332	A	C	421	0.00356295	3	1.315991741	0.268284941	4.905201681	9.33E-07	18.28355207	3.727380292	intergenic	PATE4;HYLS1	dist=9260;dist=34282		
3M	rs546409459	11	125885094	A	G	421	0.00356295	3	1.180777655	0.249820856	4.726497517	2.28E-06	18.91954737	4.002868361	intronic	HYLS1	.	HYLS1	
3M	rs528609331	11	125972300	C	T	421	0.00356295	3	1.256070429	0.253271825	4.959376858	7.07E-07	19.58124182	3.948327054	intronic	CDON	.	CDON	
3M	rs7104959	11	129976231	C	T	421	0.00356295	3	1.212990302	0.260168118	4.66233262	3.13E-06	17.92046105	3.843668505	intronic	PRDM10	.	PRDM10	
12M	rs117699122	12	451096	T	C	439	0.00341686	4	1.919399067	0.369742041	5.191184271	2.09E-07	14.04001627	2.704588306	intergenic	CCDC77;B4GALNT3	dist=8456;dist=8843	CCDC77;B4GALNT3	
3M	rs189360484	12	1761344	A	G	421	0.00475059	4	1.530180886	0.301727521	5.071399785	3.95E-07	16.8078794	3.314248553	intronic	ADIPOR2	.	ADIPOR2	
3M	rs141754456	12	19998198	T	C	421	0.00712589	7	2.240311418	0.429474084	5.21640654	1.82E-07	12.14603335	2.328429207	intergenic	AEBP2;LINC02398	dist=475971;dist=16487	AEBP2	
3M	rs118184666	12	20271815	G	A	421	0.00712589	6	2.026245701	0.430149631	4.710560123	2.47E-06	10.95098026	2.324772421	intergenic	LINC02468;PDE3A	dist=143914;dist=96722		
3M	rs549931083	12	20363352	A	C	421	0.00356295	3	1.829003959	0.357281438	5.119224688	3.07E-07	14.32826937	2.798913946	intergenic	LINC02468;PDE3A	dist=235451;dist=5185	PDE3A	
3M	rs151323346	12	20859090	T	C	421	0.00593824	4	1.39062978	0.295827471	4.700813538	2.59E-06	15.89038883	3.380348678	intronic	SLCO1B3;SLCO1B3-SLCO1B7	.		
3M	rs371879555	12	22863028	T	C	421	0.00356295	3	1.139634583	0.238093921	4.786491727	1.70E-06	20.10337646	4.200023233	intergenic	ETNK1;LOC101928441	dist=172363;dist=312608	ETNK1	
3M	rs183466664	12	26668754	A	G	421	0.00475059	4	1.341044738	0.289344304	4.634771518	3.57E-06	16.01818822	3.456090156	intronic	ITPR2	.	ITPR2	
3M	rs77353774	12	28095919	G	A	421	0.00712589	6	1.82528489	0.373198242	4.890925749	1.00E-06	13.1054362	2.679541026	intergenic	PTHLH;LOC729291	dist=123186;dist=89706	PTHLH	
3M	rs113167689	12	28283029	C	T	421	0.00712589	6	1.892111617	0.365850586	5.171815182	2.32E-07	14.13641354	2.733356286	intronic	CCDC91	.		
3M	rs17510814	12	28316036	A	C	421	0.00712589	6	1.905807603	0.368446684	5.17254649	2.31E-07	14.03879235	2.714096892	intronic	CCDC91	.	CCDC91	
3M	rs141756120	12	28358163	A	C	421	0.00831354	6	1.868183897	0.361717894	5.164753879	2.41E-07	14.27840304	2.764585375	intronic	CCDC91	.		
3M	rs117991215	12	28358540	T	C	421	0.00831354	6	1.855759228	0.359817411	5.15750259	2.50E-07	14.33366601	2.77918736	intronic	CCDC91	.		
3M	rs191930622	12	47890872	G	A	421	0.0023753	3	1.061255347	0.232228501	4.569875551	4.88E-06	19.67835791	4.306103677	intronic	VDR	.	VDR	
3M	rs56302696	12	47899047	G	A	421	0.0023753	3	1.068148373	0.231704225	4.60996503	4.03E-06	19.89590409	4.315847075	intronic	VDR	.		
3M	rs185620578	12	48175616	C	T	421	0.0023753	3	1.072824946	0.227528468	4.715124023	2.42E-06	20.72322668	4.39505442	intergenic	ASB8;CCDC184	dist=18101;dist=8028	ASB8;CCDC184	
3M	rs190806532	12	48468364	G	T	421	0.0023753	3	1.082541559	0.225298273	4.804926128	1.55E-06	21.32695498	4.438560431	intergenic	ZNF641;ANP32D	dist=117118;dist=4195	ZNF641;ANP32D	
3M	rs568658857	12	49460215	G	A	421	0.00593824	5	1.486911933	0.32318174	4.600853791	4.21E-06	14.23611926	3.094234224	intronic	SPATS2	.	SPATS2	
3M	rs137880949	12	62912517	T	C	421	0.00475059	3	1.263235635	0.24178583	5.224605748	1.75E-07	21.60840343	4.135891677	intronic	PPM1H	.		
3M	rs191053292	12	63051500	T	C	421	0.00356295	3	1.354341299	0.243048963	5.572298207	2.51E-08	22.9266488	4.114397317	intergenic	PPM1H;AVPR1A	dist=116350;dist=91259	PPM1H;AVPR1A	
3M	rs182437250	12	63214686	T	C	421	0.00475059	4	1.307028829	0.263613284	4.958129612	7.12E-07	18.80834508	3.793435539	intergenic	AVPR1A;DPY19L2	dist=63485;dist=344227		
3M	rs12315614	12	64527177	C	A	421	0.0760095	64	5.614667617	1.219220913	4.605127386	4.12E-06	3.777106623	0.820195905	intergenic	TBK1;RASSF3	dist=25064;dist=83318	TBK1,RASSF3	
12M	rs185948380	12	70219358	T	C	439	0.00569476	5	2.022172064	0.437534471	4.621743424	3.81E-06	10.56315269	2.285534207	intergenic	MYRFL;PRANCR	dist=260261;dist=2832	MYRFL	
12M	rs111582203	12	72290549	A	G	439	0.00797267	8	3.07529407	0.552250588	5.568656944	2.57E-08	10.08356907	1.810772179	intronic	TRHDE	.	TRHDE	
12M	rs112507626	12	72316321	G	A	439	0.02277904	19	4.543772122	0.930816356	4.881491491	1.05E-06	5.244312112	1.074325771	intronic	TRHDE	.		
12M	rs112796175	12	72396569	C	T	439	0.02505695	21	4.562372955	0.978318805	4.663482835	3.11E-06	4.766833481	1.022161687	intronic	TRHDE	.		
12M	rs112856203	12	72465084	T	C	439	0.02505695	21	4.413662148	0.961866897	4.588641281	4.46E-06	4.770557441	1.039644886	intronic	TRHDE	.		
3M	rs76327548	12	100789188	G	A	421	0.01306413	11	2.458077916	0.517503975	4.749872531	2.04E-06	9.178427144	1.932352307	intergenic	GAS2L3;ANO4	dist=160900;dist=5588	GAS2L3;ANO4	
3M	rs76904423	12	100794966	G	A	421	0.01068884	10	2.190673587	0.472659243	4.634784191	3.57E-06	9.805762313	2.115689082	UTR5	ANO4	NM_001286615:c.-1068200>0;NM	.	
3M	rs180764936	12	101104843	T	C	421	0.00356295	3	1.197190428	0.248754746	4.812734014	1.49E-06	19.34730528	4.020023799	intronic	ANO4	.		
3M	rs185855183	12	101111348	C	T	421	0.00356295	3	1.256842073	0.258975211	4.853136594	1.22E-06	18.73977272	3.861373435	intronic	ANO4	.		
12M	rs139496704	12	124154097	A	G	439	0.00797267	7	2.256578283	0.493630452	4.571391967	4.84E-06	9.260757615	2.025806949	intronic	ZNF664-RFLNA	.	ZNF664, RFLNA	
12M	rs566724618	13	22889700	C	T	439	0.00341686	3	1.92789865	0.356546436	5.407146043	6.40E-08	15.16533472	2.804683764	ncRNA_intronic	LINC00621	.		
12M	rs147221953	13	22911583	G	A	439	0.00341686	3	1.853658494	0.344939547	5.373864819	7.71E-08	15.57914966	2.899058719	ncRNA_intronic	LINC00621	.	SGCG	
3M	rs139598422	13	23312875	A	G	421	0.00356295	4	1.544940466	0.274599144	5.626166351	1.84E-08	20.48865215	3.641671944	intronic	SGCG	.		
12M	rs567095960	13	39877533	T	G	439	0.00341686	3	1.525495529	0.320695601	4.75683334	1.97E-06	14.83286119	3.118221752	intergenic	COG6;LINC00332	dist=85867;dist=304276	COG6	
3M	rs143371352	13	46809699	C	T	421	0.00356295	3	1.364496992	0.257779154	5.29327904	1.20E-07	20.53416252	3.879289636	intergenic	ESD;HTR2A	dist=12538;dist=21847	ESD;HTR2A	
3M	rs75186966	13	46821623	A	C	421	0.00356295	3	1.377358459	0.260942954	5.278389159	1.30E-07	20.22813447	3.832255231	intergenic	ESD;HTR2A	dist=24462;dist=9923		
3M	rs150077525	13	57405415	A	G	421	0.00712589	7	1.765848692	0.368578785	4.790966716	1.66E-06	12.99848746	2.713124141	intergenic	PRR20E;PCDH17	dist=235197;dist=226329	PCDH17	
3M	rs534845494	13	57639730	A	G	421	0.00475059	5	1.571808362	0.313987509	5.005958262	5.56E-07	15.94317648	3.184840074	intronic	PCDH17	.		
3M	rs140062526	13	58711899	G	A	421	0.00593824	5	1.544633202	0.331067832	4.665609433	3.08E-06	14.09260875	3.020529034	intergenic	LINC00374;DIAPH3	dist=478782;dist=953688	DIAPH3	
12M	rs113791989	13	61344755	G	A	439	0.0022779	3	1.593884468	0.320567115	4.972077284	6.62E-07	15.51025373	3.119471569	intergenic	MIR3169;PCDH20	dist=144874;dist=64931	PCDH20	
12M	rs117475675	13	73424715	T	C	439	0.00455581	4	2.083781896	0.384121138	5.424804025	5.80E-08	14.12263864	2.603345406	intergenic	KLF5;LINC00392	dist=347172;dist=139529	KLF5	
12M	rs142497891	13	73462846	G	A	439	0.00455581	4	2.241850629	0.429935434	5.214389078	1.84E-07	12.12830735	2.325930644	intergenic	KLF5;LINC00392	dist=385303;dist=101398		
12M	rs368187808	13	73783371	A	C	439	0.00341686	3	1.97018281	0.370345591	5.319849503	1.04E-07	14.3645547	2.700180652	intronic	KLF12	.	KLF12	
12M	rs372194899	13	73783373	A	G	439	0.00341686	3	1.969004602	0.370330521	5.31688449	1.06E-07	14.35713287	2.700290535	intronic	KLF12	.		
3M	rs546286713	13	90758825	G	A	421	0.00475059	4	1.389978902	0.297519732	4.671888158	2.98E-06	15.70278409	3.361121605	intergenic	LINC01049;LINC00410	dist=223484;dist=132129		SLITRK5;GPC5
3M	rs567080482	13	94423151	T	C	421	0.00475059	4	1.409440819	0.301549206	4.67399944	2.95E-06	15.49995607	3.316208371	intergenic	GPC6;DCT	dist=15132;dist=13660	GPC6;DCT	
3M	rs142928734	13	100948828	G	A	421	0.00356295	3	1.242180957	0.270237412	4.59662838	4.29E-06	17.00959296	3.700449886	ncRNA_intronic	NALCN-AS1	.	NALCN	

3M	rs556680896	13	100950161	C	T	421	0.00356295	3	1.24171677	0.270060937	4.597913279	4.27E-06	17.0254659	3.702867989	ncRNA_intronic	NALCN-AS1	.		
12M	rs117245766	13	105804682	A	G	439	0.00341686	3	1.590303394	0.330622791	4.8100235	1.51E-06	14.54837244	3.024594879	intergenic	LINC00343;LINC00460	dist=42886;dist=571881		;EFNB2
12M	rs139206944	13	105814677	C	T	439	0.00341686	3	1.855091687	0.369681378	5.018082589	5.22E-07	13.57407456	2.705032115	intergenic	LINC00343;LINC00460	dist=52881;dist=561886		
12M	rs561254473	13	105826900	A	G	439	0.00341686	3	1.848475898	0.368215591	5.020091341	5.16E-07	13.63356541	2.715800268	intergenic	LINC00343;LINC00460	dist=65104;dist=549663		
3M	rs184265355	13	107359004	A	C	421	0.00593824	6	1.694033349	0.342665495	4.94369399	7.67E-07	14.42717187	2.918297916	intronic	FAM155A	.	FAM155A	
3M	rs572961122	13	107360637	C	T	421	0.00593824	6	1.632302386	0.349861236	4.665570858	3.08E-06	13.33548956	2.858276075	intronic	FAM155A	.		
3M	rs528809914	13	112386942	G	A	421	0.00356295	3	1.373302637	0.265345521	5.17552597	2.27E-07	19.50485519	3.768671108	intronic	SPACA7	.		TUBGCP3
3M	rs180765647	13	113724338	G	T	421	0.0023753	3	1.05190176	0.228624832	4.600995217	4.20E-06	20.12465213	4.373978059	intronic	GRK1	.		ATP4B;TMEM255B
3M	rs138215817	14	22173619	A	G	421	0.00475059	4	1.521988034	0.305467544	4.982486896	6.28E-07	16.31101896	3.273670217	intergenic	OR4E1;LOC105370401	dist=502284;dist=206292		SALL2;
3M	rs74704551	14	29692681	C	T	421	0.00356295	3	1.302308212	0.231983741	5.613790894	1.98E-08	24.19907048	4.31064693	intronic	PRKD1	.	PRKD1	
3M	rs1686289	14	45791779	G	A	421	0.67814727	277	-9.537609426	2.056514006	-4.637755637	3.52E-06	-2.255153927	0.486259757	intergenic	LINC02303;LINC00871	dist=76177;dist=272380		MIS18BP1;RPL10L
3M	rs176783	14	45811710	A	G	421	0.32185273	267	9.35224324	2.025260115	4.617798558	3.88E-06	2.280101466	0.493763736	intergenic	LINC02303;LINC00871	dist=96108;dist=252449		
3M	rs176786	14	45813767	T	C	421	0.32304038	268	9.449422953	2.033680348	4.64646421	3.38E-06	2.284756409	0.49171936	intergenic	LINC02303;LINC00871	dist=98165;dist=250392		
3M	rs428110	14	45825457	A	C	421	0.31710214	265	9.275749143	2.014875505	4.603633881	4.15E-06	2.284822992	0.49630858	intergenic	LINC02303;LINC00871	dist=109855;dist=238702		
3M	rs116862847	14	63674959	C	T	421	0.00712589	7	1.757642639	0.338759972	5.188460225	2.12E-07	15.3160369	2.951942625	intergenic	WDR89;SGPP1	dist=33088;dist=9258	WDR89;SGPP1	
3M	rs569916471	14	75424639	G	A	421	0.00475059	5	1.390580812	0.298176949	4.663609368	3.11E-06	15.64040878	3.353713303	intergenic	LINC01220;JDP2	dist=128231;dist=3085	JDP2	
3M	rs113767990	14	81251219	G	A	421	0.00475059	4	1.429622227	0.313123218	4.565685793	4.98E-06	14.58111547	3.193630952	intergenic	LOC101928504;STON2	dist=27839;dist=9431	STON2	
12M	rs192078346	14	103732352	T	C	439	0.00455581	4	1.644493869	0.360149192	4.566146218	4.97E-06	12.67848527	2.776627086	intronic	ZFYVE21	.	ZFYVE21	
3M	rs190251199	14	105124240	T	C	421	0.00356295	3	1.240668633	0.248987137	4.982862353	6.27E-07	20.01252919	4.016271726	intergenic	LINC02298;JAG2	dist=24744;dist=16758	JAG2	
3M	rs185155853	15	40951902	C	T	421	0.00475059	5	1.370089738	0.293393972	4.669795111	3.02E-06	15.91646574	3.408386314	intergenic	DLL4;CHAC1	dist=12829;dist=1569	DLL4;CHAC1	
3M	rs144026361	15	40956471	C	T	421	0.00475059	5	1.365790883	0.290922074	4.694696631	2.67E-06	16.13729945	3.437346588	UTR3	CHAC1	NM_001142776:c.*6970>0;NM_024111:c.*6970>0		
3M	rs558614420	15	41518672	C	T	421	0.00593824	6	1.524477016	0.330280733	4.615700711	3.92E-06	13.97508315	3.02772732	intronic	RPAP1	.	LTK;TYRO3	
3M	rs138109686	15	41759244	A	G	421	0.00593824	6	1.543778108	0.336970687	4.581342434	4.62E-06	13.59567051	2.96761718	intronic	MGA	.	MGA	
3M	rs145896760	15	41827024	G	A	421	0.00593824	6	1.564221999	0.342059488	4.572953105	4.81E-06	13.36888249	2.923468092	UTR3	MAPKBP1	NM_014994:c.*15880>0;NM_001	MAPKBP1	
3M	rs140642138	15	41832967	G	A	421	0.00593824	6	1.547446255	0.337525343	4.584681679	4.55E-06	13.58322205	2.962740492	intronic	JMJD7;JMJD7-PLA2G4B	.	JMJD7;JMJD7-PLA2G4B	
3M	rs6080	15	58545734	C	A	421	0.04394299	36	3.688174426	0.80689065	4.570847892	4.86E-06	5.664767454	1.239325304	intronic	LIPC	.	LIPC;ADAM10	
3M	rs145439370	15	58587566	T	C	421	0.03087886	26	3.500007735	0.712559708	4.911879937	9.02E-07	6.893288912	1.403391166	intergenic	LIPC;ADAM10	dist=17722;dist=1243	LIPC;ADAM10	
3M	rs149425014	15	58659461	T	C	421	0.02612827	22	3.013015463	0.631629727	4.770224288	1.84E-06	7.552247919	1.583206043	intronic	ADAM10	.		
3M	rs146442492	15	58689916	C	T	421	0.02731591	24	3.06908876	0.65936392	4.654620409	3.25E-06	7.059258577	1.516613162	intronic	ADAM10	.		
3M	rs193253461	15	58937154	A	G	421	0.01306413	12	2.449615619	0.482149005	5.080619466	3.76E-07	10.53744676	2.074047629	intergenic	SLTM;RNF111	dist=3475;dist=50509	SLTM;RNF111	
3M	rs184117160	15	59112107	C	T	421	0.01425178	11	2.271648443	0.468677492	4.846933088	1.25E-06	10.34172362	2.133663377	intronic	CCNB2	.	CCNB2	
3M	rs80292573	15	59142887	T	G	421	0.03444181	30	3.721656224	0.753598905	4.938510655	7.87E-07	6.553234912	1.326965834	intronic	MYO1E	.	MYO1E	
3M	rs182303755	15	59342593	A	C	421	0.01306413	12	2.406496876	0.477647778	5.038224785	4.70E-07	10.54799166	2.093592905	intronic	MYO1E	.		
3M	rs138217865	15	93849653	C	T	421	0.00475059	4	1.402299902	0.28969226	4.840653664	1.29E-06	16.70964097	3.451938959	intergenic	LOC105370980;LINC02207	dist=641605;dist=6907		RGMA;MCTP2
12M	rs573087160	16	5796693	G	T	439	0.0022779	3	1.508610705	0.330012697	4.571371704	4.85E-06	13.85210855	3.030186441	intergenic	MIR8065;RBFOX1	dist=164127;dist=222331		
12M	rs80212581	16	6362966	C	T	439	0.00341686	3	1.753654591	0.363868409	4.819474705	1.44E-06	13.24510343	2.74824628	intronic	RBFOX1	.	RBFOX1	
12M	rs140276610	16	6383734	C	T	439	0.00341686	4	1.668191344	0.35107035	4.751729512	2.02E-06	13.53497813	2.848431943	intronic	RBFOX1	.		
12M	rs113109648	16	6574271	T	C	439	0.0022779	3	1.641370009	0.319940791	5.130230515	2.89E-07	16.03493727	3.125578319	intronic	RBFOX1	.		
12M	rs140545577	16	6805089	C	T	439	0.00341686	3	1.756164801	0.376771481	4.6610874	3.15E-06	12.37112584	2.6541287	intronic	RBFOX1	.		
12M	rs138164904	16	6808238	C	T	439	0.00341686	3	2.188468482	0.375620207	5.826279955	5.67E-09	15.51109298	2.662263589	intronic	RBFOX1	.		
12M	rs533356672	16	7052822	A	G	439	0.00341686	3	1.552617484	0.337713192	4.597443984	4.28E-06	13.61345689	2.961092499	intronic	RBFOX1	.		
12M	rs72779724	16	10323552	C	T	439	0.08542141	74	8.555089753	1.872733081	4.568237642	4.92E-06	2.439342632	0.533978927	intergenic	GRIN2A;ATF7IP2	dist=140644;dist=62509	GRIN2A;ATF7IP2	
3M	rs553840536	16	25686574	A	G	421	0.00356295	3	1.093708141	0.236100775	4.632378455	3.61E-06	19.62034417	4.23547954	intergenic	ZKSCAN2;HS3ST4	dist=428729;dist=5385	ZKSCAN2;HS3ST4	
12M	rs180797135	16	58970512	G	A	439	0.00341686	3	1.571216829	0.339826153	4.623590074	3.77E-06	13.60575114	2.942681104	intergenic	GOT2;APOOP5	dist=236196;dist=783629	GOT2	
12M	rs139174841	16	59022371	G	A	439	0.00911162	8	2.600047823	0.565206697	4.600171647	4.22E-06	8.138919215	1.769264245	intergenic	GOT2;APOOP5	dist=288055;dist=731770		
3M	rs183817723	16	59268871	C	T	421	0.00356295	4	1.261174136	0.267795557	4.709466243	2.48E-06	17.5860507	3.734191901	intergenic	GOT2;APOOP5	dist=534555;dist=485270		
12M	rs572469018	16	72297050	C	T	439	0.00341686	3	1.768848944	0.374808516	4.719340325	2.37E-06	12.59133697	2.668029026	ncRNA_intronic	LINC01572	.		
12M	rs537465910	16	72326220	G	A	439	0.00341686	3	1.764616188	0.374136714	4.716501002	2.40E-06	12.60635704	2.672819752	ncRNA_intronic	LINC01572	.	PMFBP1;ZFHX3	
12M	rs543741501	16	72351658	T	C	439	0.00341686	3	1.762934021	0.374136356	4.712009391	2.45E-06	12.59436385	2.672822315	ncRNA_intronic	LINC01572	.		
12M	rs149214138	16	72417776	T	C	439	0.00341686	3	1.753884158	0.372559459	4.707662398	2.51E-06	12.63600287	2.68413531	ncRNA_intronic	LINC01572	.		
12M	rs550597094	16	72420594	G	A	439	0.00341686	3	1.75871144	0.372991675	4.715149313	2.42E-06	12.64143312	2.681024986	ncRNA_intronic	LINC01572	.		
12M	rs537719803	16	72564328	C	A	439	0.00341686	3	1.724217925	0.368081111	4.68434232	2.81E-06	12.72638607	2.716792496	ncRNA_intronic	LINC01572	.		
12M	rs148996503	16	72564549	G	A	439	0.00341686	3	1.724389568	0.368079708	4.684826489	2.80E-06	12.72774994	2.716802848	ncRNA_intronic	LINC01572	.		
12M	rs530059247	16	72820010	C	T	439	0.00341686	3	1.656813634	0.357508509	4.634333424	3.58E-06	12.96286187	2.797136219	intronic	ZFHX3	.		
3M	rs144954214	16	76145464	A	G	421	0.0023753	3	1.106696587	0.225254382	4.913096823	8.96E-07	21.81132631	4.439425294	intergenic	CPHXL;CNTNAP4	dist=418974;dist=131937	CNTNAP4	
3M	rs529523094	16	77681654	A	G	421	0.00356295	3	1.307832421	0.26837178	4.873211417	1.10E-06	18.15843462	3.726174193	intergenic	ADAMTS18;NUDT7	dist=246620;dist=40838	NUDT7	
3M	rs146728064	17	19362127	G	A	421	0.00712589	6	1.69020067	0.365674681	4.622143005	3.80E-06	12.64004111	2.734671147	intronic	B9D1	.	B9D1	
12M	rs72825069	17	34273909	C	T	439	0.02164009	20	4.470064227	0.904834992	4.940198229	7.80E-07	5.459778049	1.105173881	intergenic	CCL7;CCL11	dist=1667;dist=11833	CCL7;CCL8;CCL2	
12M	rs77158902	17	34365017	C	T	439	0.02277904	20	4.491884806	0.953623181	4.710335166	2.47E-06	4.939409255	1.048632227	intergenic	CCL1;C17orf102	dist=1784;dist=209106		
12M	rs188353596	17	48001236	C	T	439	0.00341686	3	1.927521209	0.371158042	5.193262681	2.07E-07	13.99205215	2.694270059	intergenic	CDK5RAP3;COPZ2	dist=19450;dist=24931	CDK5RAP3;COPZ2	
3M	rs184613584	17	50430860	A	C	421	0.00712589	6	1.77626107	0.354456155	5.011229298	5.41E-07	14.13779737	2.821223402	intronic	ACSF2	.	ACSF2	
3M	rs191271637	17	54045899	A	G	421	0.00356295	3	1.195597009	0.255047807	4.687736869	2.76E-06	18.379836	3.920833552	intergenic	KIF2B;TOM1L1	dist=220706;dist=854792	KIF2B;TOM1L1	
12M	rs147669485	18	29023686	G	T	439	0.00341686	4	1.924640141	0.391979738	4.910050065	9.11E-07	12.52628537	2.551152271	intergenic	CDH2;MIR302F	dist=846556;dist=1275226		
3M	rs185819304	18	29421615	G	A	421	0.00356295	3	1.281236388	0.25348417	5.05450257	4.32E-07	19.94011134	3.945019527	intergenic	CDH2;MIR302F	dist=1244486;dist=877296	CDH2	
3M	rs187942235	18	29450465	C	T	421	0.00356295	3	1.294384565	0.251912631	5.138228126	2.77E-07	20.39686581	3.969630251	intergenic	CDH2;MIR302F	dist=1273336;dist=848446		
3M	rs139493286	18	31236056	G	A	421	0.00356295	3	1.241116041	0.266612329	4.655133716	3.24E-06	17.46030927	3.750764282	intergenic	DSC1;DSG1	dist=73200;dist=82104	DSG1	
3M	rs143538552	18	31470299	A	G	421	0.00356295	3	1.44755014	0.274967228	5.264446053	1.41E-07	19.14572179	3.636797033	intronic	DSG3	.	DSG3	
3M	rs373746073	18	31478421	C	A	421	0.00356295	3	1.441999195	0.274877768	5.245965155	1.55E-07	19.08471968	3.637980642	UTR3	DSG3	NM_001944:c.*21610>0		
3M	rs146333745	18	57830225	C	T	421	0.00356295	4	1.368613476	0.280282935	4.882971117	1.04E-06	17.42157837	3.567823349	intergenic	ATP8B1;NEDD4L	dist=26910;dist=214001	ATP8B1;NEDD4L	
12M	rs192064655	18	63290467	C	T	439	0.0022779	3	1.627195325	0.305228607	5.331070832	9.76E-08	17.46582956	3.276232883	intronic	BCL2	.	BCL2	
12M	rs559152067	18	68362375	G	A	439	0.0022779	3	1.720252761	0.315267464	5.456486816	4.86E-08	17.30748472	3.17190993	intergenic	LOC643542;TMX3	dist=462756;dist=311313	TMX3	
3M	rs185464792	19	18686561	C	T	421	0.0023753	3	1.114587174	0.222127708	5.017776414	5.23E-07	22.58960154	4.501914727	intronic	CRTC1	.	CRTC1	
3M	rs186768950	19	18695314	C	A	421	0.0023753	3	1.117877431	0.221839436	5.039128522	4.68E-07	22.71520611	4.507764788	intronic	CRTC1	.		
3M	rs541288561	19	18758635	T	G	421	0.00475059	5	1.591688559	0.31930164	4.984905675	6.20E-07	15.61190124	3.131834835	intronic	CRTC1	.		
3M	rs559008174	19	18765249	C	T	421	0.00475059	5	1.594517191	0.319680825	4.987841211	6.11E-07	15.60256615	3.12812006	intronic	CRTC1	.		
3M	rs570407448	19	18769220	G	A	421	0.00475059	5	1.587863075	0.320089569	4.960683605	7.02E-07	15.4977984	3.124125552	intronic	CRTC1	.		
3M	rs546144116	19	19452530	C	T	421	0.0023753	3	1.157037115	0.22363222	5.173839072	2.29E-07	23.13548147	4.471627577	intronic	GATAD2A	.	GATAD2A	
3M	rs560206697	19	20546292	C	T	421	0.0023753	3	1.205675879	0.219610964	5.490053226	4.02E-08	24.99899431	4.553506728	intronic	ZNF737	.	ZNF737	
3M	rs111285015	19	22940396	G	A	421	0.00356295	3	1.280187265	0.216410877	5.915540303	3.31E-09	27.33476425	4.62083983	intergenic	ZNF723;ZNF728	dist=81729;dist=34487	ZNF728	
3M	rs1008091735	19	30599192	T	C	421	0.00356295	3	1.355816116	0.270944036	5.004044884	5.61E-07	18.46892428	3.690799086	intronic	ZNF536	.	ZNF536	
3M	rs148433854	19	30605571	G	A	421	0.00356295	3	1.369429098	0.271144709	5.050547011	4.41E-07	18.62675851	3.688067545	intronic	ZNF536	.		
12M	rs181104894	19	34621605	A	C	439	0.00341686	3	1.675710965	0.35551557	4.713467166	2.44E-06	13.25811742	2.81281633	ncRNA_intronic	SCGB1B2P	.	SCGB1B2P	
12M	rs367732718	19	35427102	G	A	439	0.0022779	3	1.635791474	0.317523551	5.151717003	2.58E-07	16.22467686	3.149372695	intergenic	LINC01531;FFAR2	dist=10262;dist=21155	FFAR2	
12M	rs547186621	20	6126094	A	G	439	0.00341686	4	1.874227998	0.3576548	5.240326692	1.60E-07	14.65191208	2.795992109	intergenic	FERMT1;CASC20	dist=3064;dist=300638	FERMT1	
3M	rs2327968	20	15832846	C	T	421	0.02494062	21	3.410130247	0.744098505	4.582901624	4.59E-06	6.158998564	1.343908089	intronic	MACROD2	.		
3M	rs2876414	20	15833059	G	T	421	0.02256532	19	3.090964158	0.673260548	4.591037107	4.41E-06	6.819109065	1.485309072	intronic	MACROD2	.	MACROD2	
3M	rs140788628	20	15877856	C	A	421	0.01068884	8	2.265436379	0.438089306	5.171174799	2.33E-07	11.80392839	2.282639604	intronic	MACROD2	.		
3M	rs559228693	20	15982684	G	A	421	0.00593824	5	1.512274875	0.324468321	4.660778189	3.15E-06	14.36435511	3.081964969	ncRNA_intronic	LOC613266	.		

12M	rs138733283	20	32087884	C	T	439	0.00455581	5	2.081660055	0.444061489	4.687774347	2.76E-06	10.55658837	2.251940385	intronic	HCK	.	HCK	
12M	rs149859280	20	32090367	C	T	439	0.00455581	5	2.082920752	0.443760413	4.693795782	2.68E-06	10.57731976	2.25346825	intronic	HCK	.		
12M	rs146249289	20	32094428	C	T	439	0.00455581	4	2.061071174	0.430206938	4.790883158	1.66E-06	11.13622941	2.324462744	intronic	HCK	.		
12M	rs145791959	20	32134626	G	A	439	0.00569476	5	2.057551242	0.446297665	4.61026665	4.02E-06	10.33002637	2.240657027	intronic	TM9SF4	.	TM9SF4	
12M	rs193041547	20	32184077	T	C	439	0.00455581	4	2.07477076	0.430413643	4.820411237	1.43E-06	11.19948524	2.32334643	intergenic	TM9SF4;TSPY26P	dist=16819;dist=5069		
12M	rs138055631	20	32192841	G	A	439	0.00569476	5	2.092550815	0.449042548	4.660027927	3.16E-06	10.37769794	2.226960461	UTR3	PLAGL2	NM_002657:c.*36110>0	PLAGL2	
12M	rs145421321	20	32274323	C	T	439	0.00569476	6	2.10438963	0.457025953	4.604529826	4.13E-06	10.07498546	2.18805955	intergenic	POFUT1;KIF3B	dist=35665;dist=3328	POFUT1	
12M	rs143432612	20	32304972	C	T	439	0.00569476	6	2.095354506	0.458885267	4.566183876	4.97E-06	9.950600304	2.179193956	intronic	KIF3B	.	KIF3B	
12M	rs139816293	20	32333540	C	T	439	0.00569476	6	2.161099014	0.461179259	4.6860282	2.79E-06	10.16096909	2.168354235	UTR3	KIF3B	NM_004798:c.*22210>0		
12M	rs200198574	20	32358851	G	A	439	0.00569476	6	2.117861385	0.463869302	4.565642465	4.98E-06	9.842519093	2.155779645	intronic	ASXL1	.	ASXL1	
12M	rs148157126	20	32361036	C	T	439	0.00455581	5	2.092134134	0.43147068	4.848844269	1.24E-06	11.23794616	2.317654586	intronic	ASXL1	.		
3M	rs557092705	20	35601989	C	T	421	0.00356295	3	1.079320791	0.235404492	4.584962596	4.54E-06	19.47695457	4.248007298	ncRNA_exonic	FER1L4	.		ERGIC3;SPAG4
12M	rs3795102	20	57683075	C	T	439	0.00455581	4	2.19105865	0.424843705	5.157328741	2.50E-07	12.13935544	2.353806795	intronic	PMEPA1	.	PMEPA1	
3M	rs184785969	21	15799112	C	A	421	0.00356295	3	1.446338922	0.269771603	5.361346058	8.26E-08	19.87364864	3.706839369	intronic	USP25	.	USP25	
3M	rs117280553	21	15834844	T	C	421	0.00356295	3	1.465128514	0.27006967	5.425002059	5.80E-08	20.08741695	3.702748263	intronic	USP25	.		
3M	rs79486609	21	15872687	G	A	421	0.00356295	3	1.447442576	0.264672828	5.468799292	4.53E-08	20.66248857	3.77824957	intronic	USP25	.		
3M	rs73227413	21	21764653	G	A	421	0.03444181	28	3.687754962	0.78647158	4.688986931	2.75E-06	5.962055143	1.271501762	ncRNA_intronic	LINC01425	.		NCAM2;MRPL39
3M	rs75024143	21	21784226	G	T	421	0.01425178	12	2.293452396	0.483452813	4.743901232	2.10E-06	9.812542411	2.068454196	ncRNA_intronic	LINC01425	.		
3M	rs192134381	21	22078395	T	C	421	0.00356295	3	1.565034889	0.268006444	5.83954202	5.23E-09	21.78881202	3.731253572	ncRNA_intronic	LINC01687	.		
3M	rs118183140	21	34105187	C	T	421	0.02019002	17	3.113677552	0.640523765	4.861142898	1.17E-06	7.589324801	1.561222322	UTR3	SLC5A3	NM_006933:c.*78320>0	SLC5A3	
3M	rs183586634	21	37390730	G	A	421	0.00593824	5	1.598766374	0.334213375	4.783669629	1.72E-06	14.31322017	2.992100476	intronic	DYRK1A	.	DYRK1A	
3M	rs117185941	21	37394182	G	A	421	0.00593824	5	1.529223098	0.31531516	4.849824212	1.24E-06	15.38087864	3.171430132	intronic	DYRK1A	.		
3M	rs118084887	21	37491518	T	C	421	0.00593824	5	1.518241868	0.31880157	4.762341255	1.91E-06	14.93826162	3.136747415	intronic	DYRK1A	.		
12M	rs117262205	21	37733520	C	T	439	0.00683371	6	2.582956261	0.529108171	4.881716823	1.05E-06	9.226311531	1.889972701	intronic	KCNJ6	.	KCNJ6	
3M	rs150539922	21	41856807	T	C	421	0.00356295	3	1.254834519	0.274365448	4.57358799	4.79E-06	16.66969372	3.64477381	intronic	PRDM15	.	PRDM15	
12M	rs5994128	22	17010042	A	G	439	0.77448747	220	-11.14584694	2.280785591	-4.886845561	1.02E-06	-2.142615062	0.438445422	intergenic	GAB4;CECR7	dist=1820;dist=26528	CECR7	
3M	rs113625788	22	19981659	C	T	421	0.00831354	7	2.000739147	0.411621625	4.860626906	1.17E-06	11.80848286	2.429415606	exonic	ARVCF	.	ARVCF	
3M	rs78547898	22	32428291	G	A	421	0.00356295	3	1.210956763	0.240651719	5.03198883	4.85E-07	20.90983951	4.155382737	intronic	BPIFC	.		RTCB;FBXO7
12M	rs117360642	22	33435071	G	A	439	0.01366743	13	3.472105912	0.689882552	5.032894224	4.83E-07	7.295291363	1.449522092	intronic	LARGE1	.	LARGE1	
12M	rs61546457	22	34073343	T	C	439	0.0501139	44	6.361384106	1.334216593	4.767879623	1.86E-06	3.573542443	0.749503495	intergenic	LARGE1;LINC02885	dist=150520;dist=683324		
12M	rs35373244	22	34073359	G	A	439	0.0501139	44	6.361383523	1.334255598	4.767739805	1.86E-06	3.573333185	0.749481585	intergenic	LARGE1;LINC02885	dist=150536;dist=683308		
12M	rs35482368	22	34073458	T	C	439	0.0501139	44	6.407425495	1.347517024	4.754986676	1.98E-06	3.528702488	0.742105652	intergenic	LARGE1;LINC02885	dist=150635;dist=683209		
12M	rs5994839	22	34088192	C	A	439	0.03986333	35	5.549459281	1.192274826	4.654513506	3.25E-06	3.903893133	0.838732797	intergenic	LARGE1;LINC02885	dist=165369;dist=668475		
3M	rs541680196	22	40132086	G	A	421	0.00593824	5	1.654258232	0.339093792	4.878468054	1.07E-06	14.38678079	2.949036589	intronic	TNRC6B	.	TNRC6B	
3M	rs185139807	22	40198777	G	A	421	0.00593824	5	1.65498472	0.338624126	4.887379825	1.02E-06	14.43305263	2.953126858	intronic	TNRC6B	.		
3M	rs141127122	22	40208435	G	A	421	0.00475059	4	1.411818313	0.302953602	4.660179993	3.16E-06	15.38248751	3.30083549	intronic	TNRC6B	.		
3M	rs148998974	22	40224526	A	G	421	0.00593824	5	1.667947442	0.339010604	4.920045046	8.65E-07	14.51295325	2.949760239	intronic	TNRC6B	.		
3M	rs555040883	22	40235472	G	A	421	0.00475059	4	1.412000774	0.302532759	4.667265721	3.05E-06	15.42730692	3.305427169	intronic	TNRC6B	.		
3M	rs182959028	22	45427152	T	C	421	0.00831354	7	1.863907765	0.395028955	4.718407959	2.38E-06	11.94446103	2.531460004	intronic	RIBC2	.	RIBC2	
3M	rs150946694	22	46457283	T	C	421	0.00475059	4	1.413199859	0.303835874	4.651194872	3.30E-06	15.30824787	3.291250592	intronic	CELSR1	.	CELSR1	

Supplemental Table S6. Target Gene Prioritization
Co-localization of associated SNPs with eQTLs or sQTLs and their target genes

Notes
SNPs clustered into risk loci, sorted by chromosomal position, then by chromosome
Multiple results for a single SNP are boxed in bold
Standard Headers
rsID: reference SNP cluster ID, chr_38: chromosome number; pos_38, position of SNP on GRCh38 reference panel; REF and ALT, reference allele and alternate allele;
Func.refGene: SNP location with respect to nearest gene; Gene.refGene: closet gene(s) upstream and downstream; GeneDetail.refGene: distance of SNP to nearest gene(s)
Special Headers
QT: quantitative trait
Abbreviations and Acronyms
3M: 3 month; 12M: 12 month; AOP: aqueous outflow pathway; QTL: quantitative trait locus; eQTL: expression quantitative trait locus; sQTL: slicing quantitative trait locus
Special Headers
Closest (gene) AOP expressed: closest gene that is aqueous outflow pathway-expressed as determined by searching on Spectacle; QTL driven gene, if AOP-expressed: gene identified as the target of the QTL, listed here only if aqueous outflow pathway-expressed
P-value of genome-wide significance?: the SNP p-value; QTL type: eQTL or sQTL; Effect: effect of the QTL on gene expression; Tissue: tissue(s) from which the QTL was identified

QT	rsID	chr	pos_38	REF	ALT	P-value if genome-wide significance	Func.refGene	Gene.refGene	Closest gene(s) AOP-expressed	QTL driven gene, if AOP-expressed	QTL type	P-value	Normalized effect size	Tissue
3 mo	rs77180278	1	102496326	T	C		intergenic	OLFM3;COL11A1	COL11A1	COL11A1	sQTL	8.10E-07	-0.96	Testis
3 mo	rs112351653	1	102754804	T	C	2.55E-08	intergenic	OLFM3;COL11A1	COL11A1	COL11A1	sQTL	8.20E-06	-0.91	Testis
3 mo	rs114413507	1	102953612	T	C	2.57E-08	intronic	COL11A1	COL11A1	COL11A1	sQTL	4.20E-07	-1	Testis
3 mo	rs116672066	1	103007360	G	A	4.46E-09	intronic	COL11A1	COL11A1	COL11A1	sQTL	4.20E-07	-1	Testis
3 mo	rs111928960	1	103168079	G	A	3.65E-09	intergenic	COL11A1;LOC101928436	COL11A1	COL11A1	sQTL	4.20E-07	-1	Testis
3 mo	rs113221952	1	103288418	A	G		intergenic	COL11A1;LOC101928436	COL11A1	COL11A1	sQTL	3.10E-08	-1.1	Testis
3 mo	rs74683551	1	111811796	G	A		intronic	KCND3	KCND3	WNT2B	eQTL	5.50E-06	1.9	Brain - Anterior cingulate cortex (BA24)
12 mo	rs146600651	1	152295232	C	T		intergenic	HRNR;FLG	HRNR	HRNR	eQTL	1.00E-05	-1.5	Brain - Hypothalamus
12 mo	rs146600651	1	152295232	C	T		intergenic	HRNR;FLG	HRNR	FLG2	eQTL	5.60E-05	-1	Heart - Atrial Appendage
3 mo	rs2274996	1	229668791	C	T		intergenic	URB2;LINC01682	URB2	TAF5L	sQTL	1.00E-05	-0.44	Cells - Cultured fibroblasts
3 mo	rs2274997	1	229668899	A	G		intergenic	URB2;LINC01682	URB2	TAF5L	sQTL	1.00E-05	-0.44	Cells - Cultured fibroblasts
3 mo	rs2385790	1	229671745	C	T		intergenic	URB2;LINC01682	URB2	TAF5L	sQTL	1.00E-05	-0.47	Adipose - Subcutaneous
3 mo	rs16850124	1	229695584	T	C		intergenic	URB2;LINC01682	URB2	TAF5L	sQTL	6.00E-06	-0.41	Cells - Cultured fibroblasts
3 mo	rs17746486	2	95056864	C	T		intergenic	MAL;MRPS5	MAL;MRPS5	GPAT2	eQTL	1.70E-05	0.5	Muscle - Skeletal
3 mo	rs17746486	2	95056864	C	T		intergenic	MAL;MRPS5	MAL;MRPS5	TEKT4	eQTL	3.40E-04	0.61	Cells - EBV-transformed lymphocytes
3 mo	rs17746486	2	95056864	C	T		intergenic	MAL;MRPS5	MAL;MRPS5	ZNF514	eQTL	7.70E-06	0.74	Prostate
3 mo	rs17746486	2	95056864	C	T		intergenic	MAL;MRPS5	MAL;MRPS5	ZNF514	eQTL	9.20E-06	0.44	Whole Blood
3 mo	rs17746486	2	95056864	C	T		intergenic	MAL;MRPS5	MAL;MRPS5	ZNF514	eQTL	5.40E-04	0.3	Esophagus - Mucosa
3 mo	rs17746486	2	95056864	C	T		intergenic	MAL;MRPS5	MAL;MRPS5	ZNF514	eQTL	7.70E-04	0.29	Cells - Cultured fibroblasts
3 mo	rs17746486	2	95056864	C	T		intergenic	MAL;MRPS5	MAL;MRPS5	ZNF514	sQTL	7.20E-22	1.4	Nerve - Tibial
3 mo	rs17746486	2	95056864	C	T		intergenic	MAL;MRPS5	MAL;MRPS5	ZNF514	sQTL	1.00E-21	1.5	Adipose - Subcutaneous
3 mo	rs17746486	2	95056864	C	T		intergenic	MAL;MRPS5	MAL;MRPS5	ZNF514	sQTL	1.00E-20	1.5	Esophagus - Muscularis
3 mo	rs17746486	2	95056864	C	T		intergenic	MAL;MRPS5	MAL;MRPS5	ZNF514	sQTL	2.40E-20	1.4	Thyroid
3 mo	rs17746486	2	95056864	C	T		intergenic	MAL;MRPS5	MAL;MRPS5	ZNF514	sQTL	2.40E-13	1.2	Colon - Sigmoid
3 mo	rs17746486	2	95056864	C	T		intergenic	MAL;MRPS5	MAL;MRPS5	ZNF514	sQTL	4.50E-13	-1.1	Thyroid
3 mo	rs17746486	2	95056864	C	T		intergenic	MAL;MRPS5	MAL;MRPS5	ZNF514	sQTL	6.00E-13	1.3	Breast - Mammary Tissue
3 mo	rs17746486	2	95056864	C	T		intergenic	MAL;MRPS5	MAL;MRPS5	ZNF514	sQTL	1.60E-12	1.7	Esophagus - Gastroesophageal Junction
3 mo	rs17746486	2	95056864	C	T		intergenic	MAL;MRPS5	MAL;MRPS5	ZNF514	sQTL	4.70E-11	-1	Skin - Sun Exposed (Lower leg)
3 mo	rs17746486	2	95056864	C	T		intergenic	MAL;MRPS5	MAL;MRPS5	ZNF514	sQTL	3.50E-08	-1	Esophagus - Muscularis
3 mo	rs17746486	2	95056864	C	T		intergenic	MAL;MRPS5	MAL;MRPS5	ZNF514	sQTL	4.10E-07	-0.77	Nerve - Tibial
3 mo	rs73085348	3	42669729	A	G		intergenic	ZBTB47;KLHL40	ZBTB47	KRBOX1	eQTL	2.80E-05	-0.35	Testis
3 mo	rs73085348	3	42669729	A	G		intergenic	ZBTB47;KLHL40	ZBTB47	NKTR	eQTL	1.10E-04	-0.37	Breast - Mammary Tissue
3 mo	rs73085348	3	42669729	A	G		intergenic	ZBTB47;KLHL40	ZBTB47	NKTR	eQTL	1.20E-04	-0.32	Esophagus - Muscularis
3 mo	rs73085348	3	42669729	A	G		intergenic	ZBTB47;KLHL40	ZBTB47	NKTR	eQTL	1.20E-04	-0.37	Colon - Transverse
3 mo	rs73085348	3	42669729	A	G		intergenic	ZBTB47;KLHL40	ZBTB47	NKTR	eQTL	1.40E-04	-0.28	Cells - Cultured fibroblasts
3 mo	rs73085348	3	42669729	A	G		intergenic	ZBTB47;KLHL40	ZBTB47	NKTR	eQTL	2.00E-04	-0.26	Nerve - Tibial
12 mo	rs2229593	3	42864724	C	T		exonic	ACKR2	ACKR2	HIGD1A	eQTL	1.20E-04	-0.64	Brain - Frontal Cortex (BA9)
3 mo	rs139943877	3	155733500	G	A		intronic	PLCH1	PLCH1	PLCH1	eQTL	7.00E-06	-0.88	Artery - Tibial
3 mo	rs139943877	3	155733500	G	A		intronic	PLCH1	PLCH1	GMPS	eQTL	1.90E-04	0.39	Thyroid
12 mo	rs76356799	3	179875980	G	A		intronic	PEX5L	PEX5L	USP13	eQTL	2.60E-05	0.48	Lung
12 mo	rs76356799	3	179875980	G	A		intronic	PEX5L	PEX5L	USP13	eQTL	1.70E-04	0.37	Thyroid
3 mo	rs75848314	5	95762636	T	C		intronic	RHOBTB3	RHOBTB3	PCSK1	eQTL	1.00E-04	-0.95	Brain - Nucleus accumbens (basal ganglia)
3 mo	rs111846247	5	95775939	T	C		intronic	RHOBTB3	RHOBTB3	GLRX	sQTL	4.60E-08	1.5	Brain - Anterior qcingulate cortex (BA24)
12 mo	rs17875371	6	30492455	C	T		intronic	HLA-E	HLA-E	HLA-A	sQTL	5.20E-06	1.2	Cells - EBV-transformed lymphocytes
12 mo	rs17875371	6	30492455	C	T		intronic	HLA-E	HLA-E	HLA-F	sQTL	2.10E-06	1.3	Cells - EBV-transformed lymphocytes
12 mo	rs17875371	6	30492455	C	T		intronic	HLA-E	HLA-E	ZFP57	eQTL	3.10E-06	0.69	Whole Blood

12 mo	rs17875371	6	30492455	C	T		intronic	HLA-E	HLA-E	ZFP57	eQTL	3.30E-06	0.76	Skin - Not Sun Exposed (Suprapubic)
12 mo	rs17875371	6	30492455	C	T		intronic	HLA-E	HLA-E	ZFP57	eQTL	4.80E-05	0.6	Adipose - Subcutaneous
12 mo	rs17875371	6	30492455	C	T		intronic	HLA-E	HLA-E	ZFP57	eQTL	7.70E-05	0.65	Nerve - Tibial
3 mo	rs17776100	7	6386848	G	A		intronic	RAC1	RAC1	CCZ1	eQTL	1.20E-06	-0.66	Skin - Not Sun Exposed (Suprapubic)
3 mo	rs17776100	7	6386848	G	A		intronic	RAC1	RAC1	CCZ1	eQTL	2.80E-06	-0.7	Thyroid
3 mo	rs17776100	7	6386848	G	A		intronic	RAC1	RAC1	CCZ1	eQTL	1.10E-05	-0.67	Lung
3 mo	rs17776100	7	6386848	G	A		intronic	RAC1	RAC1	CCZ1	eQTL	1.50E-05	-0.67	Testis
3 mo	rs17776100	7	6386848	G	A		intronic	RAC1	RAC1	CCZ1	eQTL	2.50E-05	-0.64	Nerve - Tibial
3 mo	rs17776100	7	6386848	G	A		intronic	RAC1	RAC1	CCZ1	sQTL	1.30E-09	-0.72	Adipose - Subcutaneous
3 mo	rs17776100	7	6386848	G	A		intronic	RAC1	RAC1	CCZ1	sQTL	2.80E-09	0.67	Adipose - Subcutaneous
3 mo	rs17776100	7	6386848	G	A		intronic	RAC1	RAC1	CCZ1	sQTL	4.00E-08	-0.63	Muscle - Skeletal
3 mo	rs17776100	7	6386848	G	A		intronic	RAC1	RAC1	CCZ1	sQTL	4.10E-08	0.6	Muscle - Skeletal
3 mo	rs17776100	7	6386848	G	A		intronic	RAC1	RAC1	CCZ1	sQTL	2.70E-06	0.58	Nerve - Tibial
3 mo	rs17776100	7	6386848	G	A		intronic	RAC1	RAC1	CCZ1	sQTL	2.70E-06	-0.61	Nerve - Tibial
3 mo	rs17776100	7	6386848	G	A		intronic	RAC1	RAC1	CCZ1B	eQTL	1.60E-06	0.69	Nerve - Tibial
3 mo	rs17776100	7	6386848	G	A		intronic	RAC1	RAC1	CCZ1B	eQTL	1.50E-05	0.9	Pituitary
3 mo	rs17776100	7	6386848	G	A		intronic	RAC1	RAC1	CCZ1B	eQTL	2.20E-05	0.4	Whole Blood
3 mo	rs17776100	7	6386848	G	A		intronic	RAC1	RAC1	CCZ1B	eQTL	4.00E-05	0.57	Thyroid
3 mo	rs17776100	7	6386848	G	A		intronic	RAC1	RAC1	CCZ1B	eQTL	9.60E-05	0.49	Muscle - Skeletal
12 mo	rs75862363	7	117570346	G	A		intronic	CFTR	CFTR	ANKRD7	eQTL	3.80E-04	-0.71	Skin - Not Sun Exposed (Suprapubic)
3 mo	rs423841	8	54643509	G	A		intronic	RP1	SOX17;XKR4	XKR4	eQTL	3.70E-05	0.093	Nerve - Tibial
3 mo	rs433324	8	54652049	A	G		intronic	RP1	SOX17;XKR4	XKR4	eQTL	1.60E-05	0.097	Nerve - Tibial
3 mo	rs369623	8	54659380	A	C		intronic	RP1	SOX17;XKR4	XKR4	eQTL	1.90E-04	0.083	Nerve - Tibial
3 mo	rs432393	8	54667738	C	T		intronic	RP1	SOX17;XKR4	XKR4	eQTL	4.60E-06	0.1	Nerve - Tibial
3 mo	rs3098298	8	54670278	C	T		intronic	RP1	SOX17;XKR4	XKR4	eQTL	4.60E-06	0.1	Nerve - Tibial
3 mo	rs367179	8	54675056	T	C		intronic	RP1	SOX17;XKR4	XKR4	eQTL	3.70E-06	0.1	Nerve - Tibial
3 mo	rs382476	8	54678415	G	A		intronic	RP1	SOX17;XKR4	XKR4	eQTL	1.90E-04	0.083	Nerve - Tibial
3 mo	rs384543	8	54679049	G	A		intronic	RP1	SOX17;XKR4	XKR4	eQTL	1.90E-04	0.083	Nerve - Tibial
3 mo	rs405226	8	54679776	A	G		intronic	RP1	SOX17;XKR4	XKR4	eQTL	4.60E-06	0.1	Nerve - Tibial
3 mo	rs384127	8	54684929	G	A		intronic	RP1	SOX17;XKR4	XKR4	eQTL	1.90E-04	0.083	Nerve - Tibial
3 mo	rs858397	8	54702130	A	G		intronic	RP1	SOX17;XKR4	XKR4	eQTL	2.30E-04	-0.082	Nerve - Tibial
3 mo	rs2375537	8	54706948	C	T		intronic	RP1	SOX17;XKR4	XKR4	eQTL	4.60E-06	-0.1	Nerve - Tibial
3 mo	rs720372	8	54716077	G	A		intronic	RP1	SOX17;XKR4	XKR4	eQTL	2.80E-06	-0.1	Nerve - Tibial
3 mo	rs1437781	8	54717292	T	C		intronic	RP1	SOX17;XKR4	XKR4	eQTL	4.60E-06	-0.1	Nerve - Tibial
3 mo	rs1595406	8	54718055	A	G		intronic	RP1	SOX17;XKR4	XKR4	eQTL	2.80E-06	-0.1	Nerve - Tibial
3 mo	rs1437782	8	54720202	C	T		exonic	RP1	SOX17;XKR4	XKR4	eQTL	1.90E-04	-0.083	Nerve - Tibial
3 mo	rs10105693	8	54727912	C	T		intronic	RP1	SOX17;XKR4	XKR4	eQTL	5.40E-05	-0.089	Nerve - Tibial
3 mo	rs2375536	8	54728162	T	C		intronic	RP1	SOX17;XKR4	XKR4	eQTL	1.90E-06	-0.11	Nerve - Tibial
3 mo	rs4737674	8	54749094	C	A		intronic	RP1	SOX17;XKR4	XKR4	eQTL	5.50E-05	-0.089	Nerve - Tibial
3 mo	rs11987234	8	54757269	A	G		intronic	RP1	SOX17;XKR4	XKR4	eQTL	1.00E-04	-0.086	Nerve - Tibial
3 mo	rs13277510	8	54761589	G	A		intronic	RP1	SOX17;XKR4	XKR4	eQTL	1.00E-04	-0.086	Nerve - Tibial
3 mo	rs12548593	8	54762057	G	T		intronic	RP1	SOX17;XKR4	XKR4	eQTL	5.60E-06	-0.099	Nerve - Tibial
3 mo	rs1812506	8	54763541	A	G		intronic	RP1	SOX17;XKR4	XKR4	eQTL	4.80E-06	-0.099	Nerve - Tibial
3 mo	rs16920698	8	54765874	G	A		intronic	RP1	SOX17;XKR4	XKR4	eQTL	1.00E-04	-0.086	Nerve - Tibial
3 mo	rs1561297	8	54765978	A	C		intronic	RP1	SOX17;XKR4	XKR4	eQTL	4.70E-06	-0.1	Nerve - Tibial
3 mo	rs4737676	8	54766986	G	A		intronic	RP1	SOX17;XKR4	XKR4	eQTL	1.00E-04	-0.086	Nerve - Tibial
3 mo	rs2083123	8	54767758	C	T		intronic	RP1	SOX17;XKR4	XKR4	eQTL	3.20E-05	-0.091	Nerve - Tibial
3 mo	rs983248	8	54768232	C	T		intronic	RP1	SOX17;XKR4	XKR4	eQTL	8.70E-05	-0.087	Nerve - Tibial
3 mo	rs1391463	8	54769316	T	G		intronic	RP1	SOX17;XKR4	XKR4	eQTL	2.70E-04	-0.081	Nerve - Tibial
3 mo	rs10958428	8	54773081	A	G		intronic	RP1	SOX17;XKR4	XKR4	eQTL	5.60E-06	-0.099	Nerve - Tibial
3 mo	rs13278605	8	54775611	C	T		intronic	RP1	SOX17;XKR4	XKR4	eQTL	1.60E-04	-0.084	Nerve - Tibial
3 mo	rs13276543	8	54775614	G	T		intronic	RP1	SOX17;XKR4	XKR4	eQTL	1.60E-04	-0.084	Nerve - Tibial
3 mo	rs7822082	8	54777660	T	C		intronic	RP1	SOX17;XKR4	XKR4	eQTL	4.80E-05	-0.09	Nerve - Tibial
3 mo	rs4737201	8	54778898	C	T		intronic	RP1	SOX17;XKR4	XKR4	eQTL	1.00E-04	-0.086	Nerve - Tibial
3 mo	rs7843693	8	54779552	G	A		intronic	RP1	SOX17;XKR4	XKR4	eQTL	1.20E-04	-0.081	Nerve - Tibial
3 mo	rs1396896	8	54782750	A	G		intronic	RP1	SOX17;XKR4	XKR4	eQTL	1.10E-04	-0.082	Nerve - Tibial
3 mo	rs1391462	8	54787221	C	A		intronic	RP1	SOX17;XKR4	XKR4	eQTL	1.00E-04	-0.082	Nerve - Tibial
12 mo	rs79914278	9	37658199	C	T		intronic	FRMPD1	FRMPD1	FRMPD1	eQTL	4.30E-09	0.47	Heart - Atrial Appendage
12 mo	rs79914278	9	37658199	C	T		intronic	FRMPD1	FRMPD1	FRMPD1	eQTL	4.90E-06	0.35	Heart - Left Ventricle
12 mo	rs79914278	9	37658199	C	T		intronic	FRMPD1	FRMPD1	FBXO10	sQTL	4.00E-07	-0.5	Muscle - Skeletal
12 mo	rs79914278	9	37658199	C	T		intronic	FRMPD1	FRMPD1	FBXO10	sQTL	1.60E-06	-0.5	Cells - Cultured fibroblasts
12 mo	rs10973466	9	37661113	G	A		intronic	FRMPD1	FRMPD1	FRMPD1	eQTL	1.70E-12	0.59	Heart - Atrial Appendage
12 mo	rs10973466	9	37661113	G	A		intronic	FRMPD1	FRMPD1	FRMPD1	eQTL	3.50E-05	0.35	Heart - Left Ventricle
12 mo	rs10973466	9	37661113	G	A		intronic	FRMPD1	FRMPD1	FBXO10	sQTL	3.00E-09	-0.64	Muscle - Skeletal
12 mo	rs10973466	9	37661113	G	A		intronic	FRMPD1	FRMPD1	FBXO10	sQTL	1.50E-06	0.48	Muscle - Skeletal
12 mo	rs10973466	9	37661113	G	A		intronic	FRMPD1	FRMPD1	FBXO10	sQTL	1.90E-06	-0.55	Cells - Cultured fibroblasts
12 mo	rs10973466	9	37661113	G	A		intronic	FRMPD1	FRMPD1	FBXO10	sQTL	5.00E-06	-0.76	Testis

12 mo	rs6476657	9	37664627	G	A		intronic	FRMPD1	FRMPD1	FRMPD1	eQTL	3.00E-16	0.37	Heart - Atrial Appendage
12 mo	rs6476657	9	37664627	G	A		intronic	FRMPD1	FRMPD1	FRMPD1	eQTL	4.20E-12	0.29	Heart - Left Ventricle
12 mo	rs6476657	9	37664627	G	A		intronic	FRMPD1	FRMPD1	FRMPD1	eQTL	3.90E-07	0.33	Pancreas
12 mo	rs6476657	9	37664627	G	A		intronic	FRMPD1	FRMPD1	FRMPD1	eQTL	5.80E-05	0.23	Pituitary
12 mo	rs6476657	9	37664627	G	A		intronic	FRMPD1	FRMPD1	GRHPR	eQTL	1.60E-05	-0.17	Esophagus - Mucosa
12 mo	rs6476657	9	37664627	G	A		intronic	FRMPD1	FRMPD1	GRHPR	eQTL	2.30E-05	-0.16	Thyroid
12 mo	rs2057644	9	37665983	G	A		intronic	FRMPD1	FRMPD1	FRMPD1	eQTL	3.00E-16	0.37	Heart - Atrial Appendage
12 mo	rs2057644	9	37665983	G	A		intronic	FRMPD1	FRMPD1	FRMPD1	eQTL	4.20E-12	0.29	Heart - Left Ventricle
12 mo	rs2057644	9	37665983	G	A		intronic	FRMPD1	FRMPD1	FRMPD1	eQTL	3.90E-07	0.33	Pancreas
12 mo	rs2057644	9	37665983	G	A		intronic	FRMPD1	FRMPD1	FRMPD1	eQTL	5.80E-05	0.23	Pituitary
12 mo	rs2057644	9	37665983	G	A		intronic	FRMPD1	FRMPD1	GRHPR	eQTL	1.60E-05	-0.17	Esophagus - Mucosa
12 mo	rs2057644	9	37665983	G	A		intronic	FRMPD1	FRMPD1	GRHPR	eQTL	2.30E-05	-0.16	Thyroid
12 mo	rs2057643	9	37666013	T	C		intronic	FRMPD1	FRMPD1	FRMPD1	eQTL	3.00E-16	0.37	Heart - Atrial Appendage
12 mo	rs2057643	9	37666013	T	C		intronic	FRMPD1	FRMPD1	FRMPD1	eQTL	4.20E-12	0.29	Heart - Left Ventricle
12 mo	rs2057643	9	37666013	T	C		intronic	FRMPD1	FRMPD1	FRMPD1	eQTL	3.90E-07	0.33	Pancreas
12 mo	rs2057643	9	37666013	T	C		intronic	FRMPD1	FRMPD1	FRMPD1	eQTL	5.80E-05	0.23	Pituitary
12 mo	rs2057643	9	37666013	T	C		intronic	FRMPD1	FRMPD1	GRHPR	eQTL	1.60E-05	-0.17	Esophagus - Mucosa
12 mo	rs2057643	9	37666013	T	C		intronic	FRMPD1	FRMPD1	GRHPR	eQTL	2.30E-05	-0.16	Thyroid
12 mo	rs10973475	9	37666364	C	T		intronic	FRMPD1	FRMPD1	FRMPD1	eQTL	1.70E-12	0.59	Heart - Atrial Appendage
12 mo	rs10973475	9	37666364	C	T		intronic	FRMPD1	FRMPD1	FRMPD1	eQTL	3.50E-05	0.35	Heart - Left Ventricle
12 mo	rs10973475	9	37666364	C	T		intronic	FRMPD1	FRMPD1	FBXO10	sQTL	3.00E-09	-0.64	Muscle - Skeletal
12 mo	rs10973475	9	37666364	C	T		intronic	FRMPD1	FRMPD1	FBXO10	sQTL	1.50E-06	0.48	Muscle - Skeletal
12 mo	rs10973475	9	37666364	C	T		intronic	FRMPD1	FRMPD1	FBXO10	sQTL	1.90E-06	-0.55	Cells - Cultured fibroblasts
12 mo	rs10973475	9	37666364	C	T		intronic	FRMPD1	FRMPD1	FBXO10	sQTL	5.00E-06	-0.76	Testis
12 mo	rs10814594	9	37666670	T	C		intronic	FRMPD1	FRMPD1	FRMPD1	eQTL	3.70E-16	0.37	Heart - Atrial Appendage
12 mo	rs10814594	9	37666670	T	C		intronic	FRMPD1	FRMPD1	FRMPD1	eQTL	1.60E-11	0.28	Heart - Left Ventricle
12 mo	rs10814594	9	37666670	T	C		intronic	FRMPD1	FRMPD1	FRMPD1	eQTL	3.70E-07	0.33	Pancreas
12 mo	rs10814594	9	37666670	T	C		intronic	FRMPD1	FRMPD1	FRMPD1	eQTL	5.80E-05	0.23	Pituitary
12 mo	rs10814594	9	37666670	T	C		intronic	FRMPD1	FRMPD1	GRHPR	eQTL	1.60E-05	-0.17	Esophagus - Mucosa
12 mo	rs10814594	9	37666670	T	C		intronic	FRMPD1	FRMPD1	GRHPR	eQTL	2.80E-05	-0.16	Thyroid
12 mo	rs10814594	9	37666670	T	C		intronic	FRMPD1	FRMPD1	SHB	eQTL	1.20E-04	-0.1	Whole Blood
12 mo	rs1125576	9	37667822	T	C		intronic	FRMPD1	FRMPD1	FRMPD1	eQTL	8.00E-17	0.38	Heart - Atrial Appendage
12 mo	rs1125576	9	37667822	T	C		intronic	FRMPD1	FRMPD1	FRMPD1	eQTL	8.40E-12	0.29	Heart - Left Ventricle
12 mo	rs1125576	9	37667822	T	C		intronic	FRMPD1	FRMPD1	FRMPD1	eQTL	2.10E-07	0.34	Pancreas
12 mo	rs1125576	9	37667822	T	C		intronic	FRMPD1	FRMPD1	GRHPR	eQTL	4.30E-06	-0.18	Esophagus - Mucosa
12 mo	rs1125576	9	37667822	T	C		intronic	FRMPD1	FRMPD1	GRHPR	eQTL	1.70E-05	-0.17	Thyroid
12 mo	rs60006744	9	37672694	G	T		intronic	FRMPD1	FRMPD1	FRMPD1	eQTL	1.70E-12	0.59	Heart - Atrial Appendage
12 mo	rs60006744	9	37672694	G	T		intronic	FRMPD1	FRMPD1	FRMPD1	eQTL	3.50E-05	0.35	Heart - Left Ventricle
12 mo	rs60006744	9	37672694	G	T		intronic	FRMPD1	FRMPD1	FBXO10	sQTL	3.00E-09	-0.64	Muscle - Skeletal
12 mo	rs60006744	9	37672694	G	T		intronic	FRMPD1	FRMPD1	FBXO10	sQTL	1.50E-06	0.48	Muscle - Skeletal
12 mo	rs60006744	9	37672694	G	T		intronic	FRMPD1	FRMPD1	FBXO10	sQTL	1.90E-06	-0.55	Cells - Cultured fibroblasts
12 mo	rs60006744	9	37672694	G	T		intronic	FRMPD1	FRMPD1	FBXO10	sQTL	5.00E-06	-0.76	Testis
12 mo	rs2148140	9	37673842	C	T		intronic	FRMPD1	FRMPD1	FRMPD1	eQTL	1.70E-12	0.59	Heart - Atrial Appendage
12 mo	rs2148140	9	37673842	C	T		intronic	FRMPD1	FRMPD1	FRMPD1	eQTL	3.50E-05	0.35	Heart - Left Ventricle
12 mo	rs2148140	9	37673842	C	T		intronic	FRMPD1	FRMPD1	FBXO10	sQTL	3.00E-09	-0.64	Muscle - Skeletal
12 mo	rs2148140	9	37673842	C	T		intronic	FRMPD1	FRMPD1	FBXO10	sQTL	1.50E-06	0.48	Muscle - Skeletal
12 mo	rs2148140	9	37673842	C	T		intronic	FRMPD1	FRMPD1	FBXO10	sQTL	1.90E-06	-0.55	Cells - Cultured fibroblasts
12 mo	rs2148140	9	37673842	C	T		intronic	FRMPD1	FRMPD1	FBXO10	sQTL	5.00E-06	-0.76	Testis
12 mo	rs113399724	9	37674360	C	T		intronic	FRMPD1	FRMPD1	FRMPD1	eQTL	1.70E-12	0.59	Heart - Atrial Appendage
12 mo	rs113399724	9	37674360	C	T		intronic	FRMPD1	FRMPD1	FRMPD1	eQTL	3.50E-05	0.35	Heart - Left Ventricle
12 mo	rs113399724	9	37674360	C	T		intronic	FRMPD1	FRMPD1	FBXO10	sQTL	3.00E-09	-0.64	Muscle - Skeletal
12 mo	rs113399724	9	37674360	C	T		intronic	FRMPD1	FRMPD1	FBXO10	sQTL	1.50E-06	0.48	Muscle - Skeletal
12 mo	rs113399724	9	37674360	C	T		intronic	FRMPD1	FRMPD1	FBXO10	sQTL	1.90E-06	-0.55	Cells - Cultured fibroblasts
12 mo	rs113399724	9	37674360	C	T		intronic	FRMPD1	FRMPD1	FBXO10	sQTL	5.00E-06	-0.76	Testis
12 mo	rs62582460	9	138095976	T	C		intronic	CACNA1B	CACNA1B	FAM157B	eQTL	1.10E-06	0.52	Skin - Sun Exposed (Lower leg)
12 mo	rs62582460	9	138095976	T	C		intronic	CACNA1B	CACNA1B	FAM157B	eQTL	1.80E-06	0.25	Whole Blood
3 mo	rs149949098	11	95366702	G	A		intergenic	LOC100129203;FAM76B	FAM76B	ENDOD1	eQTL	8.60E-06	0.44	Adipose - Subcutaneous
12 mo	rs112507626	12	72316321	G	A		intronic	TRHDE	TRHDE	TRHDE	eQTL	5.10E-05	0.39	Colon - Transverse
12 mo	rs112796175	12	72396569	C	T		intronic	TRHDE	TRHDE	TRHDE	eQTL	1.30E-04	0.38	Colon - Transverse
12 mo	rs145791959	20	32134626	G	A		intronic	TM9SF4	TM9SF4	LINC00028	eQTL	1.30E-04	-1	Spleen
12 mo	rs138055631	20	32192841	G	A		UTR3	PLAGL2	PLAGL2	LINC00028	eQTL	1.30E-04	-1	Spleen
12 mo	rs145421321	20	32274323	C	T		intergenic	POFUT1;KIF3B	POFUT1;KIF3B	LINC00028	eQTL	1.30E-04	-1	Spleen
12 mo	rs143432612	20	32304972	C	T		intronic	KIF3B	KIF3B	LINC00028	eQTL	1.30E-04	-1	Spleen
12 mo	rs139816293	20	32333540	C	T		UTR3	KIF3B	KIF3B	LINC00028	eQTL	2.10E-04	-0.96	Spleen
12 mo	rs5994128	22	17010042	A	G		intergenic	GAB4;CECR7	CECR7	CECR7	eQTL	3.80E-18	-0.51	Cells - Cultured fibroblasts
12 mo	rs5994128	22	17010042	A	G		intergenic	GAB4;CECR7	CECR7	CECR7	eQTL	7.30E-17	-0.44	Skin - Sun Exposed (Lower leg)

12 mo	rs5994128	22	17010042	A	G		intergenic	GAB4;CECR7	CECR7	CECR7	eQTL	4.20E-16	-0.42	Adipose - Subcutaneous
12 mo	rs5994128	22	17010042	A	G		intergenic	GAB4;CECR7	CECR7	CECR7	eQTL	2.70E-15	-0.38	Muscle - Skeletal
12 mo	rs5994128	22	17010042	A	G		intergenic	GAB4;CECR7	CECR7	CECR7	eQTL	3.60E-15	-0.41	Thyroid
12 mo	rs5994128	22	17010042	A	G		intergenic	GAB4;CECR7	CECR7	CECR7	sQTL	1.50E-07	-0.6	Ovary
12 mo	rs5994128	22	17010042	A	G		intergenic	GAB4;CECR7	CECR7	IL17RA	sQTL	1.50E-07	-0.6	Ovary

Supplemental Table S7. Target Gene Prioritization
Prioritized target gene lists

Notes
441 genes 45 paralagous groups
Abbreviations and Acronyms
AOP: aqueous outflow pathway; QTL: quantitative trait locus; eQTL: expression quantitative trait locus; sQTL: slicing quantitative trait locus

AOP-Expressed	AOP-expressed up, downstream	AOP-expressed e/sQTLs	Combined	Paralogous groups	Gene product function
ABCG2	ADGRL3	ANKRD7	ABCG2		Superfamily of ATP-binding cassette (ABC) transporters, white subfamily
ABHD5	ALDH8A1	CCZ1	ABHD5		Coenzyme A-dependent lysophosphatidic acid acyltransferase
ACADSB	ANTXR2	CCZ1B	ACADSB		Acyl-CoA dehydrogenase family of enzymes involved in the metabolism of fatty acids or branch chained amino acids
ACKR2	AP2A2	CECR7	ACKR2		One of a family of beta chemokine receptors critical for the recruitment of effector immune cells to the inflammation site
ACSF2	ASB3	COL11A1	ACSF2	ACSF2	Acyl-CoA synthase family, catalyzes the initial reaction in fatty acid metabolism, by forming a thioester with CoA
ACSL3	ASNS	ENDOD1	ACSL3	ACSL3	"
ACVR2A	ATP4B	FAM157B	ACVR2A		Activin A Receptor Type 2A, binds activins, members of TGFB superfamily
ADAM10	ATP8A1	FBXO10	ADAM10		ADAM family transmembrane metalloprotease which mediates the ectodomain shedding of a myriad of transmembrane proteins
ADGRG6	AZIN1	FLG2	ADGRG6		G-protein coupled receptor activated by type IV collagen
ADGRL3	BMP2K	FRMPD1	ADGRL3	ADGRL3	Latrophilin subfamily of G-protein coupled receptors
ADGRL4	BTRC	GLRX	ADGRL4	ADGRL4	"
ADIPOR2	C4orf33	GMPS	ADIPOR2		Adiponectin receptor for ADIPOQ, an essential hormone secreted by adipocytes that regulates glucose and lipid metabolism
AEBP2	CAMSAP1	GPAT2	AEBP2		Accessory subunit for the core Polycomb repressive complex 2 (PRC2) involved in transcriptional repression
AGAP1	CHCHD3	GRHPR	AGAP1		GTPase-activating protein for ARF1 and ARF5
AGR3	COMMD10	HIGD1A	AGR3		Disulfide isomerase (PDI) family of endoplasmic reticulum (ER) proteins that catalyze protein folding and thiol-disulfide interchange reactions
AGTR1	CUZD1	HLA-A	AGTR1		Receptor for angiotensin II, a potent vasopressor hormone and a primary regulator of aldosterone secretion
AHCTF1	EDEM3	HLA-F	AHCTF1		Involved in nuclear pore complex assembly and regulation of cytokinesis.
AHR	EFNB2	HRNR	AHR		ligand-activated helix-loop-helix transcription factor involved in the regulation of biological responses to planar aromatic hydrocarbons.
AIG1	ERGIC3	IL17RA	AIG1		Hydrolyzes bioactive fatty-acid esters of hydroxy-fatty acids
ALDH5A1	FAM153A	KRBOX1	ALDH5A1	ALDH5A1	Aldehyde dehydrogenase family
ALK	FAM153C	LINC00028	ALDH8A1	ALDH8A1	"
AMY2A	FAM24B	NKTR	ALK		receptor tyrosine kinase of the insulin receptor superfamily
AMY2B	FBXO7	PCSK1	AMY2A	AMY2A	Alpha-amylase family, catalyze starch catabolism
ANO4	GLRX2	PLCH1	AMY2B	AMY2B	"
ANP32D	GPC5	SHB	ANKRD7		Unknown
ANTXR1	GYPC	TAF5L	ANO4		Anotamin family (Ca2+-activated Cl− channel)
AOAH	ID2	TEKT4	ANP32D		Phosphoprotein 32 (PP32) is a tumor suppressor
AP1AR	IGFBP7	TRHDE	ANTXR1	ANTXR1	type I transmembrane protein and endothelial marker
ARAP2	JADE1	USP13	ANTXR2	ANTXR2	"
ARFIP1	KAZALD1	WNT2B	AOAH		Catalyzes the hydrolysis of acyloxylacyl-linked fatty acyl chains from bacterial lipopolysaccharides, effectively detoxifying these molecules
ARHGAP21	KCTD8	XKR4	AP1AR		Enables AP-1 adaptor complex binding activity and kinesin binding activity
ARHGEF26	KLF10	ZFP57	AP2A2		Subunit of the AP-2 adaptor protein complex, which is involved in linking lipid and protein membrane components with the clathrin lattice
ARHGEF28	KLF6	ZNF514	ARAP2		PIP3 dependent GTPase-activating protein; modulates actin cytoskeleton remodeling by regulating ARF and RHO family members
ARHGEF3	L1TD1		ARFIP1		Involved in negative regulation of retrograde transport, endosome to Golgi
ARID5B	LVRN		ARHGAP21		Functions as a GTPase-activating protein (GAP) for RHOA and CDC42.
ARRDC3	MALRD1		ARHGEF26	ARHGEF26	Rho-guanine nucleotide exchange factor (Rho-GEF) family
ARVCF	MCTP2		ARHGEF28	ARHGEF28	"
ASB3	MIS18BP1		ARHGEF3	ARHGEF3	"
ASB8	MMS22L		ARID5B		forms a histone H3K9Me2 demethylase complex; regulates the transcription of target genes involved in adipogenesis
ASXL1	MND1		ARRDC3		arrestin family of proteins, which regulate G protein-mediated signaling
ATF7IP2	MRGPRX3		ARVCF		Catenin family member that plays an important role in the formation of adherens junction complexes

ATP8B1	MRPL39		ASB3	ASB3	Family of substrate-recognition components of a SCF-like ECS (Elongin-Cullin-SOCS-box protein) E3 ubiquitin-protein ligase complex
ATXN1	MRPS2		ASB8	ASB8	"
AVPR1A	MSRB2		ASNS		Involved in the synthesis of asparagine
B4GALNT3	MUC12		ASXL1		member of the Polycomb group of proteins, which are necessary for the maintenance of stable repression of homeotic and other loci
B9D1	NCAM2		ATF7IP2		Probable Polycomb group (PcG) protein involved in transcriptional regulation mediated by ligand-bound nuclear hormone receptors
BCKDHB	NEBL		ATP4B		P-type cation-transporting ATPase
BCL2	NRXN1		ATP8A1	ATP8A1	Catalytic component of a P4-ATPase flippase complex
BCL2L11	OTUD1		ATP8B1	ATP8B1	"
BEND7	PAQR3		ATXN1		Chromatin-binding factor that repress Notch signaling in the absence of Notch intracellular domain by acting as a CBF1 corepressor.
BRF2	PGCKA1		AVPR1A		Receptor for arginine vasopressin
C1orf21	PITRM1		AZIN1		Antizyme inhibitor family
C6orf118	PLXNA4		B4GALNT3		Catalyzes transfer of N-acetylgalactosamine (GalNAc) in N-linked glycans and probably O-linked glycans
CACNA1B	POLD4		B9D1		Component of the tectonic-like complex in primary cilia, required for ciliogenesis and sonic hedgehog/SHH signaling
CAMTA1	POU3F2		BCKDHB		subunit of branched-chain keto acid dehydrogenase, a multienzyme complex associated with the inner membrane of mitochondria
CAV2	RAB6C		BCL2		integral outer mitochondrial membrane protein that blocks the apoptotic death of some cells such as lymphocytes
CCDC184	RBM43		BCL2L11		BCL-2 protein family
CCDC77	RELL1		BEND7		Transcription factor, unknown role
CCDC91	RGMA		BMP2K		BMP-2-inducible kinase
CCL2	RGMB		BRF2		General activator of RNA polymerase III transcription
CCL7	RGPD4		BTRC		F-box protein, one of the four subunits of ubiquitin protein ligase complex SCF (SKP1-cullin-F-box)
CCL8	RIOK2		C1orf21		Unknown
CCNB2	RND3		C4orf33		"
CCNG1	RNF144A		C6orf118		"
CCR6	RNF2		CACNA1B		Calcium Voltage-Gated Channel Subunit involved in muscle contraction
CCSER1	RPL10L		CAMSAP1		Key microtubule-organizing protein that specifically binds the minus-end of non-centrosomal microtubules
CCSER2	RTCB		CAMTA1		Calmodulin-Binding Transcription Activator 1
CD1	SAA4		CAV2		Caveolin, a scaffolding protein within caveolar membranes
CDC42BPA	SALL2		CCDC184		Coiled-Coil Domain-Containing Protein, unknown function
CDCA2	SCLT1		CCDC77		Coiled-Coil Domain-Containing Protein, unknown function
CDH2	SGK1		CCDC91		Coiled-Coil Domain-Containing Protein, involved in the regulation of membrane traffic through the trans-Golgi network
CDH9	SLITRK5		CCL2	CCL2	C-C Motif Chemokine Ligand
CDK5RAP3	SOX17		CCL7	CCL7	"
CDON	SPAG4		CCL8	CCL8	"
CECR7	SSH3		CCNB2	CCNB2	Cyclin (cell cycle regulator)
CELF2	ST6GAL2		CCNG1	CCNG1	"
CELSR1	TAC1		CCR6		Beta chemokine receptor family, ligand is CCL20
CFAP99	TBL1XR1		CCSER1	CCSER1	Microtubule binding protein family, mediates bundle formation
CFTR	TLR2		CCSER2	CCSER2	"
CHAC1	TM2D1		CCZ1	CCZ1	Enables guanyl-nucleotide exchange factor activity, predicted to be involved in vesicle-mediated transport.
CMC1	TMEM255B		CCZ1B	CCZ1B	"
CNTN4	TRIM56		CD1		Member of CD1 gene family that encode surface glycoproteins (CD1a-e) that present lipid/glycolipid antigens to T cells
CNTN5	TSN		CDC42BPA		Serine/threonine-protein kinase plays a role in the regulation of cytoskeleton reorganization and cell migration
CNTN6	TUBGCP3		CDCA2		Targeting subunit of protein phosphatase 1, with a role in targeting this protein to chromatin during anaphase
CNTNAP2	XKR4		CDH2	CDH2	Cadherin family (calcium-dependent cell adhesion protein)
CNTNAP4	ZFAT		CDH9	CDH9	"
COG6	ZMAT3		CDK5RAP3		Substrate adapter of E3 ligase complexes mediating ufmylation
COL11A1			CDON		Component of a cell-surface receptor complex that mediates cell-cell interactions between muscle precursor cells.
COMMD10			CECR7		GPCR receptor for the MIP-3-beta chemokine. Probable mediator of EBV effects on B-lymphocytes or of normal lymphocyte functions
COPZ2			CELF2		RNA-binding protein implicated in the regulation of several post-transcriptional events
CRTC1			CELSR1		Cadherin superfamily, flamingo subfamily
CSMD1			CFAP99		Cilia And Flagella Associated Protein
CSMD3			CFTR		Superfamily of ATP-binding cassette (ABC) transporters, functions as a chloride channel; mutations in this gene cause cystic fibrosis
CTBP2			CHAC1		Gamma-glutamylcyclotransferase family; deglycinates the Notch receptor, which prevents receptor maturation and inhibits Notch signaling

CXCL12			CHCHD3		Encodes an inner mitochondrial membrane scaffold protein
CZ1P-ASNS			CMC1		Component of the MITRAC (mitochondrial translation regulation assembly intermediate of cytochrome c oxidase complex) complex
DCANP1			CNTN4	CNTN4	Contactin (axon-associated cell adhesion molecule)
DCLK2			CNTN5	CNTN5	"
DCT			CNTN6	CNTN6	"
DIAPH3			CNTNAP2	CNTNAP2	Contactin-associated protein family
DIRAS2			CNTNAP4	CNTNAP4	"
DLC1			COG6		Subunit of the conserved oligomeric Golgi complex that is required for maintaining normal structure and activity of the Golgi apparatus
DLGAP2			COL11A1		One of the three collagen chains that form type XI collagen
DLL4			COMMD10		Scaffold protein in the commander complex that is essential for endosomal recycling of transmembrane cargos
DMRT2			COPZ2		subunit of the coatomer protein complex. COPI vesicles function in the retrograde Golgi-to-ER transport of dilysine-tagged proteins
DPP10			CRTC1		CREB-regulated transcription coactivator protein; involved in energy metabolism
DSG1			CSMD1	CSMD1	CUB And Sushi Multiple Domains 1 family, complement cascade inhibitor
DSG3			CSMD3	CSMD3	"
DYNC2H1			CTBP2		Corepressor targeting diverse transcription regulators. Functions in brown adipose tissue (BAT) differentiation
DYRK1A			CUZD1		Localized to zymogen granules, where it functions in trypsinogen activation
EBF2			CXCL12		Activates chemokine receptor CXCR4 to induce a rapid and transient rise in the level of intracellular calcium ions and chemotaxis
EPHB1			CZ1P-ASNS		naturally occurring readthrough transcript; a candidate for nonsense-mediated mRNA decay (NMD)
ERV3-1			DCANP1		Binds with and transactivates the corticotropin-releasing hormone (CRH) promoter
ESD			DCLK2		Member of the protein kinase superfamily and the doublecortin family
ESR1			DCT		Dopachrome Tautomerase, role in melanin biosynthesis, also involved in energy metabolism
ETAA1			DIAPH3		Diaphanous subfamily of the formin family; actin nucleation and elongation factor required for the assembly of F-actin structures
ETNK1			DIRAS2		DIRAS subfamily of small GTPases
EVX2			DLC1		Rho GTPase Activating Protein
FAM155A			DLGAP2		May play a role in the molecular organization of synapses and neuronal cell signaling.
FAM170A			DLL4		Ligand in Notch signaling, Delta subfamily, characterized by a DSL domain, EGF repeats, and a transmembrane domain
FAM76B			DMRT2		DMRT family transcriptional activator that directly regulates early activation of the myogenic determination
FAXDC2			DPP10		S9B family in clan SC of the serine proteases, promotes cell surface expression of the potassium channel KCND2
FBXO11			DRD1		Encodes the D1 subtype of the dopamine receptor
FERMT1			DSG1	DSG1	Desmoglein protein subfamily, components of desmosome
FFAR2			DSG3	DSG3	"
FIGN			DYNC2H1		Dynein, may function as a motor for intraflagellar retrograde transport. Functions in cilia biogenesis.
FOXN2			DYRK1A		Dual-specificity tyrosine phosphorylation-regulated kinase (DYRK) family
FOXN2			EBF2		Transcription factor that acts in synergy with the Wnt-responsive LEF1/CTNNB1 pathway
FRMD3			EDEM3		Involved in endoplasmic reticulum-associated degradation (ERAD), a quality control mechanism
FRMPD1			EFNB2		Encodes a member of the ephrin (EPH) family
FST			ENDOD1		Plays a role in the modulation of innate immune signaling through the cGAS-STING pathway by interacting with RNF26
FSTL5			EPHB1		Receptor tyrosine kinase which binds ephrin-B family ligands residing on adjacent cells
GABRG2			ERGIC3		Involved in endoplasmic reticulum to Golgi vesicle-mediated transport and positive regulation of intracellular protein transport.
GABRP			ERV3-1		This gene contains sequence derived from endogenous retrovirus
GAL			ESD		Serine hydrolase that belongs to the esterase D family, may be involved in the recycling of sialic acids
GAS2L3			ESR1		Estrogen receptor 1
GATAD2A			ETAA1		Replication stress response protein that accumulates at DNA damage sites and promotes replication fork progression and integrity
GMNC			ETNK1		An ethanolamine kinase, may be a rate-controlling step in phosphatidylethanolamine biosynthesis
HLA-E			EVX2		Homeobox transcription factor
GOT2			FAM153A	FAM153A	Unknown
GPC6			FAM153C	FAM153C	Unknown
GPLD1			FAM155A		Also known as NALF1; auxillary component of the NALCN sodium channel complex
GRB14			FAM157B		Unknown
GRIN2A			FAM170A		Acts as a nuclear transcription factor that positively regulates the expression of heat shock genes
GRM7			FAM24B		Unknown
GTF2B			FAM76B		Gene Ontology (GO) annotations related to this gene include deNEDDylase activity
HAAO			FAXDC2		Promotes megakaryocyte differentiation

HAS2			FBXO10	FBXO10	Substrate recognition component of a SCF (SKP1-CUL1-F-box protein) E3 ubiquitin-protein ligase complex
HCK			FBXO11	FBXO11	"
HDAC4			FBXO7	FBXO7	"
HDAC9			FERMT1		Fermitin family member, involved in integrin signaling and linkage of the actin cytoskeleton to the extracellular matrix
HMCN1			FFAR2		GP40 family of G protein-coupled receptors, regulates whole-body energy homeostasis, adipogenesis
HOXD13			FIGN		ATP-dependent microtubule severing protein
HRNR			FLG2		Filaggrin-like protein upregulated by calcium, proteolyzed by calpain 1, involved in epithelial homeostasis
HS3ST1			FOXN2		Transcription factor
HS3ST4			FRMD3		Unknown
HS6ST1			FRMPD1		Involved in establishment of protein localization to membrane and regulation of G protein-coupled receptor signaling pathway
HTR2A			FST	FST	TGFB superfamily member follistatin (activin antagonist)
HYLS1			FSTL5	FSTL5	"
IDUA			GABRG2	GABRG2	Subunit of the GABA-A receptor, a major inhibitory neurotransmitter in the brain
IGF2BP3			GABRP	GABRP	"
INTU			GAL		Neuroendocrine hormone of the central and peripheral nervous systems, role in contraction of smooth muscle
ITPR2			GAS2L3		Cytoskeletal linker protein, may promote and stabilize the formation of the actin and microtubule network
JADE1			GATAD2A		Transcriptional repressor, acts as a component of the histone deacetylase NuRD complex which participates in the remodeling of chromatin
JAG2			GLRX	GLRX	Glutaredoxin family, antioxidant defense system
JDP2			GLRX2	GLRX2	"
JMJD7			GMNC		Regulator of DNA replication
JMJD7-PLA2G4B			GMPS		Catalyzes the conversion of xanthine monophosphate (XMP) to GMP
KCND3			GOT2		Glutamic-oxaloacetic transaminase, role in the intracellular NAD(H) redox balance, facilitates cellular uptake of long-chain free fatty acids
KCNE4			GPAT2		Glycerol-3-phosphate O-acyltransferase
KCNIP1			GPC5	GPC5	Glypican family, act as co-receptors on the cell surface, playing crucial roles in regulating various signaling pathways
KCNIP3			GPC6	GPC6	"
KCNIP4			GPLD1		Glycosylphosphatidylinositol (GPI) anchor-degrading enzyme
KCNJ6			GRB14		Encodes a growth factor receptor-binding protein that interacts with insulin receptors and insulin-like growth-factor receptors
KIAA1614			GRHPR		Unknown
KIF2B			GRIN2A		Encodes a member of the glutamate-gated ion channel protein family
KIF3B			GRM7		G-protein coupled receptor activated by glutamate that regulates axon outgrowth
KLF12			GTF2B		General transcription factor IIB, one of the ubiquitous factors required for transcription initiation by RNA polymerase II
KLF5			GYPC		Integral membrane glycoprotein, regulates the mechanical stability of red cells
KLHDC7A			HAAO		Catalyzes the synthesis of quinolinic acid (QUIN) from 3-hydroxyanthranilic acid
KLHDC8A			HAS2		Hyaluronic acid synthetase
LARGE1			HCK		Src family of tyrosine kinases, regulates innate immune responses
LEMD1			HDAC4	HDAC4	Class IIa histone deacetylase
LEPR			HDAC9	HDAC9	"
LINC02360			HIGD1A		Acts upstream of or within negative regulation of apoptotic process. Located in mitochondrion and nucleoplasm
LINGO2			HLA-A		HLA class I heavy chain paralogues
LIPC			HLA-E		"
LOC100130987			HLA-F		"
LRP2			HMCN1		Hemicentin, multifunctional
LRRFIP1			HOXD13		Homeobox family transcription factors
LTK			HRNR		Involved in cell envelope organization and establishment of skin barrier
LUZP2			HS3ST1	HS3ST1	Heparan sulfate biosynthetic enzyme family
M1AP			HS3ST4	HS3ST4	"
MACROD2			HS6ST1	HS6ST1	"
MACROH2A1			HTR2A		Seratonin receptor; stimulating these receptors increases IOP
MAL			HYLS1		Centriolar and ciliogenesis associated protein
MAPK10			ID2		Transcriptional regulator, regulates the circadian clock
MAPKBP1			IDUA		Enzyme that catalyzes hydrolysis of the terminal alpha-L-iduronic acid residues of two glycosaminoglycans
MAT2B			IGF2BP3		Binds to the 5' UTR of the insulin-like growth factor II leader 3 mRNA and may repress translation of insulin-like growth factor II
MGA			IGFBP7		Encodes a member of the insulin-like growth factor (IGF)-binding protein (IGFBP) family

MMRN1			IL17RA		Encodes a receptor for IL17A and IL17F, major effector cytokines of innate and adaptive immune system
MOGAT1			INTU		Plays a key role in ciliogenesis
MOGS			ITPR2		Inositol 1,4,5-triphosphate receptor family, whose members are second messenger intracellular calcium release channels
MRPS5			JADE1		Involved in several processes, including negative regulation of canonical Wnt signaling pathway
MTX2			JAG2		Notch ligand of the serrate family
MVB12B			JDP2		Component of the AP-1 transcription factor that represses transactivation mediated by the Jun family of protein
MYO1E			JMJD7		Endopeptidase that cleaves histone N-terminal tails, to generate 'tailless nucleosomes'
MYRFL			JMJD7-PLA2G4B		Read-through protein; unknown function
NAALADL2			KAZALD1		Secreted member of the insulin growth factor-binding protein (IGFBP) superfamily
NALCN			KCND3	KCND3	Voltage-gated potassium (Kv) channel
NAT1			KCNE4	KCNE4	"
NBAS			KCNIP1	KCNIP1	Voltage-gated potassium (Kv) channel interacting protein
NEDD4L			KCNIP3	KCNIP3	"
NR2F1			KCNIP4	KCNIP4	"
NRG3			KCNJ6	KCNJ6	Voltage-gated potassium (Kv) channel
NRP2			KCTD8		Auxiliary subunit of GABA-B receptors that determine the pharmacology and kinetics of the receptor response
NSG1			KIAA1614		Predicted to be involved in centrosome cycle; establishment or maintenance of cell polarity; and regulation of cellular localization
NT5C2			KIF2B	KIF2B	Kinesin family (plus end microtubule-dependent motor)
NUDCD2			KIF3B	KIF3B	"
NUDT7			KLF10	KLF10	Kruppel-like factor subfamily of zinc finger proteins (transcriptional regulators)
OLFM3			KLF12	KLF12	"
PC			KLF5	KLF5	"
PCDH17			KLF6	KLF6	"
PCDH20			KLHDC7A	KLHDC7A	Kelch domain-containing family, unknown function
PCDH7			KLHDC8A	KLHDC8A	"
PDCD6IP			KRBOX1		Predicted to be involved in regulation of DNA-templated transcription
PDE3A			L1TD1		Unknown
PEX5L			LARGE1		N-acetylglucosaminyltransferase gene family, participates in glycosylation of alpha-dystroglycan
PHYHIP			LEMD1		Unknown
PITX2			LEPR		Receptor for leptin, an adipocyte-specific hormone that regulates body weight, and is involved in the regulation of fat metabolism
PKNOX2			LINGO2		Predicted to act upstream of or within positive regulation of synapse assembly.
PLAGL2			LIPC		Catalyzes the hydrolysis of triglycerides and phospholipids
PLCH1			LRP2		Megalin, a multi-ligand endocytic receptor
PLPPR1			LRRFIP1		Transcriptional repressor
PLPPR5			LTK		Ros/insulin receptor family of tyrosine kinases
PMEPA1			LUZP2		Unknown
PMFBP1			LVRN		Metalloprotease
POFUT1			M1AP		Required for meiosis I progression during spermatogenesis
PPARGC1A			MACROD2		Chromatin modifier; deacetylates a signaling molecule generated by the deacetylation of acetylated lysine residues
PPM1H			MACROH2A1		Chromatin modifier; variant histone H2A which replaces conventional H2A in a subset of nucleosomes where it represses transcription
PPP1R21			MAL		Integral membrane protein, involved in vesicular trafficking cycling between the Golgi complex and the apical plasma membrane
PPP2R3A			MALRD1		Enhances production and/or transport of FGF19 and thus has a role in regulation of bile acid synthesis
PPP3CA			MAPK10		MAP kinase family of signal transduction proteins
PPP3CC			MAPKBP1		MAP kinase signaling, scaffold protein
PRCP			MAT2B		Regulatory subunit of S-adenosylmethionine synthetase
PRDM10			MCTP2		Predicted to be involved in regulation of neurotransmitter secretion
PRDM15			MGA		Functions as a dual-specificity transcription factor, regulating the expression of both MAX-network and T-box family target genes
PRKD1			MIS18BP1		Required for recruitment of CENPA to centromeres and normal chromosome segregation during mitosis
PRR16			MMRN1		Multimerin, a massive, soluble protein found in platelets and in the endothelium of blood vessels
PTHLH			MMS22L		Forms a complex with tonsoku-like, DNA repair protein (TONSL)
PUM3			MND1		Required for proper homologous chromosome pairing and efficient cross-over and intragenic recombination during meiosis
RABGEF1			MOGAT1		Unknown
RAC1			MOGS		encodes the first enzyme in the N-linked oligosaccharide processing pathway

RASSF3			MRGPRX3		Encodes a member of the mas-related/sensory neuron specific subfamily of G protein coupled receptors
RBFOX1			MRPL39	MRPL39	Mitochondrial ribosomal protein
RFLNA			MRPS2	MRPS2	"
RGS7			MRPS5	MRPS5	"
RHOBTB3			MSRB2		Methionine-sulfoxide reductase; upon oxidative stress, may play a role in the preservation of mitochondrial integrity
RIBC2			MTX2		Involved in transport of proteins into the mitochondrion
RNF111			MUC12		Membrane-associated mucin
DRD1			MVB12B		Component of the ESCRT-I complex, a regulator of vesicular trafficking process.
RTKN			MYO1E		Nonmuscle class I myosin of the unconventional myosin protein family
SASH1			MYRFL		Unknown
SCGB1B2P			NAALADL2		N-acetylated alpha-linked acidic dipeptidase (NAALADase) gene family
SEMA5B			NALCN		Voltage-independent, nonselective cation channel
SEPHS1			NAT1		One of two arylamine N-acetyltransferase (NAT) genes in the human genome, participates in detoxification
SFTA1P			NBAS		Involved in Golgi-to-endoplasmic reticulum (ER) retrograde transport
SGCG			NCAM2		Neural cell adhesion molecule
SGPP1			NEBL		Binds to actin and plays an important role in the assembly of the Z-disk in cardiac muscle
SLC4A5			NEDD4L		Member of Nedd4 family of HECT domain E3 ubiquitin ligases
SLC5A3			NKTR		PPIase that catalyzes the cis-trans isomerization of proline imidic peptide bonds in oligopeptides and may therefore assist protein folding
SLC7A11			NR2F1	NR2F1	Nuclear hormone receptor and transcriptional regulator (Coup transcription factor)
SLITRK3			NRG3	NRG3	Nuclear hormone receptor and transcriptional regulator (mineralocorticoid receptor)
SLTM			NRP2		Neuregulin gene family, direct ligand for the ERBB4 tyrosine kinase receptor
SMARCA2			NRXN1		Neurexin-1, binds neuroligins
SPAG17			NSG1		Role in the recycling mechanism in neurons of multiple receptors, acts at the level of early endosomes
SPATS2			NT5C2		Hydrolase that serves as an important role in cellular purine metabolism
SPRED2			NUDCD2		May regulate the LIS1/dynein pathway by stabilizing LIS1 with Hsp90 chaperone
SPTY2D1			NUDT7		Nudix hydrolase involved in eliminating potentially toxic nucleotide metabolites from the cell
SRRT			OLFM3		Olfactomedin Related ER Localized Protein
STAG1			OTUD1		Deubiquitinating enzyme
STEAP2			PAQR3		Encodes a seven-transmembrane protein localized in the Golgi apparatus
STON2			PC		Pyruvate carboxylase
STX6			PCDH17	PCDH17	Protocadherin gene family, a subfamily of the cadherin superfamily
SWT1			PCDH20	PCDH20	"
SYK			PCDH7	PCDH7	"
TBC1D12			PGCKA1		acts as a tumor supressor targeting KEAP1/USP17/ELK1/CDK6 axis
TBK1			PCSK1		Subtilisin-like proprotein convertase family, process protein and peptide precursors trafficking
TBX15			PDCD6IP		Multifunctional protein involved in endocytosis, multivesicular body biogenesis
TBX18			PDE3A		Member of the cGMP-inhibited cyclic nucleotide phosphodiesterase (cGI-PDE) family
TFDP2			PEX5L		Accessory subunit of hyperpolarization-activated cyclic nucleotide-gated (HCN) channels
TFEC			PHYHIP		Interacts with PHYH, a peroxisomal protein involved in the alpha-oxidation of 3-methyl branched fatty acids
TFPI			PITRM1		ATP-dependent metalloprotease that degrades post-cleavage mitochondrial transit peptides
THSD7A			PITX2		RIEG/PITX homeobox family of transcription factors
TLE1			PKNOX2		Homeodomain transcription factor
TM9SF4			PLAGL2		Transcription factor
TMEM108			PLCH1		PLC-eta family of the phosphoinositide-specific phospholipase C
TMEM201			PLPPR1	PLPPR1	Plasticity-related gene (PRG) family, mediates lipid phosphate phosphatase activity in neurons
TMEM26			PLPPR5	PLPPR5	"
TMEM65			PLXNA4		Coreceptor for SEMA3A; function to inhibit axonal outgrowth, or to stimulate the growth of apical dendrites
TMEM86A			PMEPA1		Encodes a transmembrane protein that contains a Smad interacting motif involved in TGFB signaling
TMX3			PMFBP1		Involved in the general organization of cellular cytoskeleton
TNK2			POFUT1		Enzyme that O-fucosylates EGF-like repeats, including in the Notch protein; essential for Notch activity
TNRC6B			POLD4		Component of the tetrameric DNA polymerase delta complex (Pol-delta4), plays a role in high fidelity genome replication and repair
TOM1L1			POU3F2		POU-III class of neural transcription factors, enhances the activation of corticotropin-releasing hormone regulated genes
TRHDE			PPARGC1A		Transcriptional coactivator that regulates the genes involved in energy metabolism

TRMT12			PPM1H		Phosphoprotein phosphatase involve in multilamellar body biogenesis; deficient in Parkinson's disease
TRPS1			PPP1R21		Component of the FERRY complex (Five-subunit Endosomal Rab5 and RNA/ribosome intermediary)
TSC22D2			PPP2R3A		Regulatory subunits of the protein phosphatase 2
TSPAN14			PPP3CA	PPP3CA	Calcium-dependent, calmodulin-stimulated protein phosphatase
TTF1			PPP3CC	PPP3CC	" Calcineurin
TYRO3			PRCP		Encodes a member of the peptidase S28 family of serine exopeptidases
TYW1			PRDM10	PRDM10	Transcription factor that contains C2H2-type zinc-finger, unknown role
URB2			PRDM15	PRDM15	"
USP25			PRKD1		Serine/threonine-protein kinase that converts transient diacylglycerol (DAG) signals into prolonged physiological effects downstream of PKC
VDR			PRR16		Regulator of cell size that promotes cell size increase independently of mTOR and Hippo signaling pathways
VTA1			PTHLH		Parathyroid hormone-like hormone, a neuroendocrine peptide
VWC2			PUM3		Protein tyrosine phosphatase (PTP) family. PTPs are known to be signaling molecules
WDR70			RAB6C		Inhibits the poly(ADP-ribosyl)ation activity of PARP1 and the degradation of PARP1 by CASP3 following genotoxic stress
WDR89			RABGEF1		Involved in endocytic membrane fusion and membrane trafficking of recycling endosomes
WNT5A			RAC1		Rac family GTPase
WWC1			RASSF3		A member of a subfamily of the RAS superfamily, plasma membrane GTP-binding proteins
XPNPEP1			RBFOX1		Fox-1 family of RNA-binding proteins, regulate tissue-specific alternative splicing
YAP1			RBM43		Predicted to enable RNA binding activity
ZBTB47			RELL1		Involved in positive regulation of p38MAPK cascade
ZBTB49			RFLNA		Involved in the regulation of the perinuclear actin network and nuclear shape through interaction with filamins
ZFHX3			RGMA	RGMA	Member of the repulsive guidance molecule (RGM) family
ZFYVE21			RGMB	RGMB	"
ZKSCAN2			RGPD4		Predicted to contribute to GTPase activator activity
ZNF117			RGS7		GTPase activator component of the RGS7-GNB5 complex that regulates G protein-coupled receptor signaling cascades
ZNF536			RHOBTB3		Rab9-regulated ATPase required for endosome to Golgi transport.
ZNF641			RIBC2		Predicted to be involved in flagellated sperm motility. Located in axonemal microtubule.
ZNF664			RIOK2		Serine/threonine-protein kinase involved in the final steps of cytoplasmic maturation of the 40S ribosomal subunit.
ZNF728			RND3		Rho Family GTPase
ZNF737			RNF111	RNF111	RING finger domain-containing E3 ubiquitin ligase family, modifies transcription
ZNF804B			RNF144A	RNF144A	"
			RNF2	RNF2	"
			RPL10L		Ribosome protein L10
			RTCB		Catalytic subunit of the tRNA-splicing ligase complex
			RTKN		Scaffold protein that interacts with GTP-bound Rho proteins to inhibit their GTPase activity
			SAA4		Major acute phase reactant
			SALL2		Transcription factor that plays a role in eye development
			SASH1		Scaffold protein involved in the TLR4 signaling pathway
			SCGB1B2P		Secretoglobin Family 1B Member 2, Pseudogene
			SCLT1		Encodes a major glucose transporter in the mammalian blood-brain barrier
			SEMA5B		Semaphorin protein family which regulates axon growth during development of the nervous system
			SEPHS1		Enzyme that synthesizes selenophosphate from selenide and ATP
			SFTA1P		SFTA1P (Surfactant Associated 1, LncRNA) is a Pseudogene
			SGCG		Component of the sarcoglycan complex, forms a link between the F-actin cytoskeleton and the extracellular matrix of muscle
			SGK1		Serum/Glucocorticoid Regulated Kinase, plays an important role in cellular stress response
			SGPP1		Sphingosine-1-phosphate (S1P) is a bioactive sphingolipid metabolite that regulates diverse biologic processes
			SHB		Adapter protein which regulates several signal transduction cascades by linking activated receptors to downstream signaling components
			SLC4A5		Mediates sodium- and bicarbonate-dependent electrogenic sodium bicarbonate cotransport
			SLC5A3		Electrogenic Na(+)-coupled sugar symporter
			SLC7A11		Heterodimer with SLC3A2, that functions as an antiporter
			SLITRK3	SLITRK3	SLITRK family of structurally related transmembrane proteins that are involved in controlling neurite outgrowth
			SLITRK5	SLITRK5	"
			SLTM		When overexpressed, acts as a general inhibitor of transcription that eventually leads to apoptosis
			SMARCA2		SWI/SNF family of proteins with helicase and ATPase activities altering the chromatin structure

			SOX17		Proangiogenic transcription factor, Inhibits Wnt signaling to activate Notch, thus reduces Sox17 to restrict angiogenesis
			SPAG17	SPAG17	Plays a critical role in the function and structure of motile cilia
			SPAG4	SPAG4	Involved in spermatogenesis
			SPATS2		Unknown
			SPRED2		Negatively regulates Ras signaling pathways and downstream activation of MAP kinases; essential regulator of lymphangiogenesis
			SPTY2D1		Histone chaperone that stabilizes pre-existing histone tetramers and regulates replication-independent histone exchange on chromatin
			SRRT		Acts as a mediator between the cap-binding complex (CBC) and the primary microRNAs (miRNAs) processing machinery
			SSH3		Protein phosphatase which may play a role in the regulation of actin filament dynamics
			ST6GAL2		Sialyltransferase, transfers sialic acid from the donor of substrate CMP-sialic acid to galactose containing acceptor substrate
			STAG1		Component of cohesin complex, a complex required for the cohesion of sister chromatids after DNA replication
			STEAP2		Integral membrane protein that functions as a NADPH-dependent ferric-chelate reductase
			STON2		Encodes a protein which is a membrane protein involved in regulating endocytotic complexes
			STX6		Targets endosomes to the trans-Golgi network, and may therefore function in retrograde trafficking
			SWT1		SWT1 RNA Endoribonuclease Homolog
			SYK		Non-receptor type Tyr protein kinase; involved in regulating angiogenesis
			TAC1		Encodes 4 tachykinin peptide hormone family products, substance P, neurokinin A, neuropeptide K, neuropeptide gamma
			TAF5L		Functions as a component of the PCAF complex, capable of efficiently acetylating histones in a nucleosomal context
			TBC1D12		RAB11A-binding protein that plays a role in neurite outgrowth; enables GTPase activity
			TBK1		Serine/threonine kinase; induces angiogenesis
			TBL1XR1		F-box-like protein, plays an essential role in transcription activation mediated by nuclear receptors
			TBX15	TBX15	T-box family transcription factor
			TBX18	TBX18	"
			TEKT4		Microtubule inner protein (MIP) part of the dynein-decorated doublet microtubules (DMTs) in cilia and flagellar axoneme
			TFDP2		Binds DNA cooperatively with E2F family members to stimulate transcription; locks adipocyte differentiation
			TFEC		Microphthalmia (MiT) family of basic helix-loop-helix leucine zipper transcription factors
			TFPI		Kunitz-type serine protease inhibitor that regulates the tissue factor (TF)-dependent pathway of blood coagulation
			THSD7A		Role in actin cytoskeleton rearrangement; soluble form promotes sprouting angiogenesis
			TLE1		Transcriptional corepressor involved in WNT and Notch signaling
			TLR2		Toll-like receptor (TLR) family, role in pathogen recognition and activation of innate immunity
			TM2D1		May participate in amyloid-beta-induced apoptosis via its interaction with beta-APP42
			TM9SF4		Multifunctional protein located in Golgi apparatus and early endosome
			TMEM108		Multifunctional protein located in endosomes
			TMEM201		Proposed to be involved in actin-dependent nuclear movement
			TMEM255B		Unknown
			TMEM26		Selective surface protein marker of beige adipocytes, may coexist with classical brown adipocytes in brown adipose tissue
			TMEM65		Enzyme involved in lysoplasmalogen metabolism in the adipocyte tissue and macrophages
			TMEM86A		Disulfide isomerase (PDI) family of ER proteins that catalyze protein folding and thiol-disulfide interchange
			TMX3		TNF receptor superfamily; mediates calcineurin-dependent activation of NF-AT, as well as NF-kappa-B and AP-1
			TNK2		Non-receptor tyrosine-protein and serine/threonine-protein kinase promoting angiogenesis
			TNRC6B		Plays a role in RNA-mediated gene silencing by both micro-RNAs (miRNAs) and short interfering RNAs (siRNAs)
			TOM1L1		Probable adapter protein involved in signaling pathways
			TRHDE		Extracellular peptidase that specifically cleaves and inactivates the neuropeptide thyrotropin-releasing hormone
			TRIM56		Tripartite motif (TRIM) family, E3 ubiquitin-protein ligase
			TRMT12		tRNA wybutosine-synthesizing protein 2 homolog
			TRPS1		Transcriptional repressor. Binds specifically to GATA sequences and represses expression of GATA-regulated genes
			TSC22D2		Involved in negative regulation of cell cycle
			TSN		DNA-binding protein that recognizes consensus sequences at the breakpoint junctions in chromosomal translocation
			TSPAN14		Required for ADAM10 exit from the ER and for enzymatic maturation and trafficking to the cell surface
			TTF1		Transcription termination factor that is localized to the nucleolus and plays a critical role in ribosomal gene transcription
			TUBGCP3		Gamma-tubulin complex necessary for microtubule nucleation at the centrosome
			TYRO3		Receptor tyrosine kinase that transduces signals from the ECM
			TYW1		Probable component of the wybutosine biosynthesis pathway.
			URB2		Involved in regulation of signal transduction by p53 class mediator and ribosome biogenesis

			USP13	USP13	De-ubiquitinase
			USP25	USP25	"
			VDR		Vitamin D3 receptor, a member of the nuclear hormone receptor superfamily
			VTA1		Involved in trafficking of the multivesicular body
			VWC2		BMP antagonist
			WDR70		Unknown
			WDR89		Unknown
			WNT2B	WNT2B	Wingless-type MMTV integration site (WNT) family of highly conserved, secreted signaling factors, function in the Wnt signaling pathway
			WNT5A	WNT5A	"
			WWC1		Acts as a scaffolding protein, facilitating protein-protein interactions within the Hippo pathway, regulated by Notch
			XKR4		Phospholipid scramblase that promotes phosphatidylserine exposure on apoptotic cell surface
			XPNPEP1		Metalloaminopeptidase that plays a role in degradation and maturation of tachykinins, neuropeptides, and peptide hormones
			YAP1		Downstream nuclear effector of the Hippo signaling pathway
			ZBTB47	ZBTB47	Zinc Finger And BTB Domain Containing transcription factor
			ZBTB49	ZBTB49	"
			ZFAT		Transcriptional regulator involved in apoptosis and cell survival; downregulates immune response genes
			ZFHX3		Transcriptional regulator
			ZFP57		Transcription regulator and master maintainer of DNA methylation imprints
			ZFYVE21		role in cell adhesion, and thereby in cell motility which requires repeated formation and disassembly of focal adhesions
			ZKSCAN2		Transcriptional regulator
			ZMAT3		RNA-binding protein, target gene of p53, essential for p53-mediated growth inhibition
			ZNF117	ZNF117	Zinc finger family of transcriptional regulators
			ZNF514	ZNF514	Zinc finger family of transcriptional regulators
			ZNF536	ZNF536	Zinc finger family of transcriptional regulators
			ZNF641	ZNF641	Zinc finger family of transcriptional regulators
			ZNF664	ZNF664	Zinc finger family of transcriptional regulators
			ZNF728	ZNF728	Zinc finger family of transcriptional regulators
			ZNF737	ZNF737	Zinc finger family of transcriptional regulators
			ZNF804B	ZNF804B	Zinc finger family of transcriptional regulators

441 genes 45 paralogous groups

Supplemental Table S8. Prioritized Target Gene Validation
GC-regulation of prioritized target genes

Notes

The list of GWAS prioritized target genes compiled in this study was compared to lists from a study of GC-regulated DEGs in human TM cell strains (reference below), GC-regulated DEGs from 2 paired eye studies (Table S9 and S10), and genes identified as GC-regulated DEGs by a glucocorticoid receptor in silico (Table S11)

Abbreviations and Acronyms

DEG: differentially-expressed gene; GC: glucocorticoid

Reference

(1) Nehme A, Lobenhofer EK, Stamer WD, Edelman JL. Glucocorticoids with different chemical structures but similar glucocorticoid receptor potency regulate subsets of common and unique genes in human trabecular meshwork cells. BMC Med Genomics. 2009;2:58.

			GC-regulated count		
GWAS prioritized target genes	Paired eye matches	In silico matches	DEGs in TM cells	New DEGs paired eye	New DEGs in silico
ABCG2			1		
ABHD5			1		
ACADSB		ACADSB	1		
ACKR2					
ACSF2		ACSF2			
ACSL3		ACSL3			1
ACVR2A		ACVR2A			1
ADAM10		ADAM10	1		
ADGRG6		ADGRG6			1
ADGRL3		ADGRL3			1
ADGRL4					
ADIPOR2		ADIPOR2			1
AEBP2		AEBP2			1
AGAP1		AGAP1			1
AGR3					
AGTR1					
AHCTF1		AHCTF1	1		
AHR					
AIG1		AIG1			1
ALDH5A1		ALDH5A1	1		
ALDH8A1					
ALK		ALK			1
AMY2A					
AMY2B					
ANKRD7					

ANO4		ANO4			1
ANP32D			1		
ANTXR1	ANTXR1	ANTXR1	1		
ANTXR2	ANTXR2	ANTXR2	1		
AOAH					
AP1AR					
AP2A2		AP2A2			1
ARAP2					
ARFIP1		ARFIP1			1
ARHGAP21			1		
ARHGEF26	ARHGEF26	ARHGEF26	1		
ARHGEF28			1		
ARHGEF3		ARHGEF3	1		
ARID5B		ARID5B			1
ARRDC3		ARRDC3	1		
ARVCF		ARVCF	1		
ASB3					
ASB8		ASB8	1		
ASNS		ASNS	1		
ASXL1		ASXL1			1
ATF7IP2					
ATP4B		ATP4B			1
ATP8A1					
ATP8B1		ATP8B1	1		
ATXN1		ATXN1	1		
AVPR1A					
AZIN1		AZIN1			1
B4GALNT3					
B9D1			1		
BCKDHB					
BCL2		BCL2			1
BCL2L11	BCL2L11			1	
BEND7	BEND7	BEND7	1		
BMP2K		BMP2K	1		
BRF2		BRF2			1
BTRC		BTRC	1		
C1D					
C1orf21		C1orf21	1		
C4orf19					
C4orf33					
C6orf118					
CACNA1B		CACNA1B			1
CAMSAP1					
CAMTA1		CAMTA1			1
CAV2		CAV2	1		
CCDC184					
CCDC77		CCDC77			1

CCDC91		CCDC91	1		
CCL2	CCL2		1		
CCL7					
CCL8					
CCNB2			1		
CCNG1			1		
CCR6					
CCSER1		CCSER1			1
CCSER2		CCSER2			1
CCZ1					
CCZ1B					
CDC42BPA		CDC42BPA	1		
CDCA2		CDCA2	1		
CDH2	CDH2	CDH2	1		
CDH9		CDH9			1
CDK5RAP3					
CDON		CDON			1
CECR7					
CELF2		CELF2			1
CELSR1		CELSR1			1
CFAP99					
CFTR				1	
CHAC1	CHAC1		1		
CHCHD3		CHCHD3			1
CMC1					
CNTN4		CNTN4			1
CNTN5		CNTN5			1
CNTN6			1		
CNTNAP2		CNTNAP2	1		
CNTNAP4			1		
COG6			1		
COL11A1	COL11A1	COL11A1	1		
COMMD10		COMMD10			
COPZ2	COPZ2	COPZ2	1		
CRTC1		CRTC1			1
CSMD1		CSMD1			1
CSMD3		CSMD3			1
CTBP2		CTBP2			1
CUZD1					
CXCL12			1		
CZ1P-ASNS					
DCANP1					
DCLK2					
DCT		DCT			1
DIAPH3	DIAPH3	DIAPH3	1		
DIRAS2					
DLC1		DLC1	1		

DLGAP2					
DLL4					
DMRT2					
DPP10		DPP10			1
DRD1					
DSG1					
DSG3					
DYNC2H1		DYNC2H1	1		
DYRK1A		DYRK1A			1
EBF2		EBF2			1
EDEM3			1		
EFNB2		EFNB2			1
ENDOD1					
EPHB1		EPHB1			1
ERGIC3	ERGIC1	ERGIC3	1		
ERV3-1					
ESD					
ESR1		ESR1			1
ETAA1					
ETNK1			1		
EVX2		EVX2			1
FAM153A					
FAM153C					
FAM155A			1		
FAM157B					
FAM170A					
FAM24B					
FAM76B					
FAXDC2		FAXDC2			1
FBXO10		FBXO11			1
FBXO11					
FBXO7			1		
FERMT1					
FFAR2					
FIGN					
FLG2					
FOXN2			1		
FRMD3	FRMD3			1	
FRMPD1		FRMPD1			1
FST	FST	FST	1		
FSTL5		FSTL5	1		
GABRG2					
GABRP					
GAL	GAL			1	
GAS2L3		GAS2L3	1		
GATAD2A					
GLRX					

GLRX2			1		
GMNC		GMNC	1		
GMPS		GOT2			1
GOT2		GPAT2	1		
GPAT2					
GPC5		GPC5	1		1
GPC6		GPC6			1
GPLD1		GPLD1			1
GRB14		GRB14			1
GRHPR		GRHPR			1
GRIN2A		GRIN2A			1
GRM7		GRM7			1
GTF2B					
GYPC					
HAAO		HAAO			1
HAS2					
HCK		HCK	1		
HDAC4		HDAC4	1		
HDAC9		HDAC9			1
HIGD1A		HIGD1A			1
HLA-A					
HLA-E		HLA-E			1
HLA-F		HLA-F			1
HMCN1	HMCN1	HMCN1		1	
HOXD13		HOXD13			1
HRNR					
HS3ST1			1		
HS3ST4		HS3ST4			1
HS6ST1	HS6ST1		1		
HTR2A	HTR2A			1	
HYLS1		HYLS1	1		
ID2		ID2	1		
IDUA			1		
IGF2BP3		IGF2BP3			1
IGFBP7		IGFBP7			1
IL17RA		IL17RA	1		1
INTU		INTU	1		1
ITPR2		ITPR2			1
JADE1					
JAG2					
JDP2		JDP2			1
JMJD7		JMJD7	1		
JMJD7-PLA2G4B		JMJD7-PLA2G4I	1		
KAZALD1		KAZALD1			1
KCND3		KCND3			1
KCNE4	KCNE4	KCNE4	1		
KCNIP1		KCNIP1			1

KCNIP3		KCNIP3			1
KCNIP4		KCNIP4			1
KCNJ6		KCNJ6			1
KCTD8					
KIAA1614		KIAA1614			1
KIF2B		KIF2B			1
KIF3B			1		
KLF10					
KLF12		KLF12	1		
KLF5		KLF5	1		
KLF6		KLF6			1
KLHDC7A	KLHDC7A		1		
KLHDC8A			1		
KRBOX1			1		
L1TD1					
LARGE1		LARGE1			1
LEMD1					1
LEPR		LEPR			1
LINGO2		LINGO2			1
LIPC		LIPC	1		
LOC100130987		LOC100130987			1
LRP2		LRP2			1
LRRFIP1					
LTK					
LUZP2		LUZP2			1
LVRN		LVRN			1
M1AP		M1AP			1
MACROD2		MACROD2			1
MACROH2A1		MACROH2A1			1
MAL		MAL			1
MALRD1					
MAPK10	MAPK10	MAPK10		1	
MAPKBP1		MAPKBP1			1
MAT2B		MAT2B	1		
MCTP2		MCTP2	1		
MGA		MGA	1		
MIS18BP1			1		
MMRN1			1		
MMS22L		MMS22L			1
MND1					
MOGAT1					
MOGS			1		
MRGPRX3					
MRPL39					
MRPS2					
MRPS5					
MSRB2					

MTX2		MTX2			1
MUC12				1	
MVB12B	MVB12B	MVB12B		1	
MYO1E		MYO1E			1
MYRFL					
NAALADL2					
NALCN			1		
NAT1			1		
NBAS		NBAS			1
NCAM2		NCAM2			1
NEBL		NEBL			1
NEDD4L		NEDD4L			1
NKTR		NKTR	1		
NR2F1		NR2F1	1		
NRG3		NRG3			1
NRP2		NRP2	1		
NRXN1		NRXN1			1
NSG1		NSG1			1
NT5C2		NT5C2			1
NUDCD2					
NUDT7					
OLFM3					
OTUD1			1		
PAQR3					
PC	PC	PC		1	
PCDH17		PCDH17			1
PCDH20		PCDH20	1		
PCDH7		PCDH7			1
PCSK1					
PDCD6IP			1		
PDE3A		PDE3A			1
PEX5L		PEX5L	1		
PGCKA1		PGCKA1			1
PHYHIP		PHYHIP			1
PITRM1		PITRM1			1
PITX2		PITX2	1		
PKNOX2		PKNOX2			1
PLAGL2			1		
PLCH1			1		
PLPPR1		PLPPR1			1
PLPPR5		PLXNA4			1
PLXNA4					
PMEPA1		PMEPA1			1
PMFBP1					
POFUT1		POFUT1			1
POLD4		POLD4			1
POU3F2		POU3F2			1

PPARGC1A		PPARGC1A	1		
PPM1H		PPM1H			1
PPP1R21		PPP1R21	1		
PPP2R3A		PPP2R3A	1		
PPP3CA		PPP3CA	1		
PPP3CC		PPP3CC	1		
PRCP		PRCP			
PRDM10		PRDM10			1
PRDM15		PRDM15			1
PRKD1		PRKD1			1
PRR16		PRR16			1
PTHLH	PTHLH	PTHLH			1
PUM3			1		
RAB6C			1		
RABGEF1			1		
RAC1		RAC1	1		
RASSF3					
RBFOX1	RBFOX1	RBFOX1	1		
RBM43	RBM43			1	
RELL1			1		
RFLNA		RFLNA			1
RGMA		RGMA			1
RGMB		RGPD4			1
RGPD4		RGS7	1		
RGS7					
RHOBTB3		RHOBTB3	1		
RIBC2		RIBC2			1
RIOK2			1		
RND3		RND3			1
RNF111					
RNF144A		RNF144A	1		
RNF2		RNF2			
RPL10L		RPL10L			1
RTCB					1
RTKN		RTKN			1
SAA4	SAA4	SAA4		1	
SALL2					
SASH1		SASH1			1
SCGB1B2P					
SCLT1					
SEMA5B		SEMA5B	1		
SEPHS1					
SFTA1P					
SGCG		SGCG			1
SGK1		SGK1			1
SGPP1			1		
SHB		SHB	1		

SLC4A5		SLC4A5			1
SLC5A3		SLC5A3			1
SLC7A11					1
SLITRK3					
SLITRK5			1		
SLTM					
SMARCA2		SMARCA2			1
SOX17			1		
SPAG4		SPAG4			1
SPATS2		SPATS2	1		
SPRED2					
SPTY2D1					
SRRT					
SSH3			1		
ST6GAL2					
STAG1		STAG1	1		1
STEAP2	STEAP2	STEAP2		1	
STON2	STON2	STON2	1		
STX6		STX6			1
SWT1			1		
SYK			1		
TAC1					
TAF5L					
TBC1D12					
TBK1					
TBL1XR1			1		
TBX15		TBX15			1
TBX18	TBX18			1	
TEKT4		TEKT4	1		
TFDP2		TFDP2	1		
TFEC			1		
TFPI					
THSD7A		THSD7A			1
TLE1		TLE1	1		
TLR2					1
TM2D1			1		
TM9SF4			1		
TMEM108		TMEM108			1
TMEM201			1		
TMEM255B					
TMEM26			1		
TMEM65					
TMEM86A		TMEM86A			1
TMX3		TMX3			1
TNK2	TNK2	TNK2		1	
TNRC6B		TNRC6B			1
TOM1L1					
TRHDE		TRHDE			1

TRIM56			1		
TRMT12					
TRPS1		TRPS1	1		
TSC22D2		TSC22D2	1		
TSN			1		
TSPAN14					
TTF1					
TUBGCP3					
TYRO3		TYRO3			1
TYW1			1		
URB2					
USP13		USP13			1
USP25		USP25	1		
VDR		VDR			1
VTA1			1		
VWC2					
WDR70		WDR70			1
WDR89			1		
WNT2B		WNT2B			1
WNT5A		WNT5A			1
WWC1		WWC1			1
XKR4		XKR4	1		
XPNPEP1		XPNPEP1	1		
YAP1			1		
ZBTB47		ZBTB47	1		
ZBTB49					
ZFAT			1		
ZFHX3		ZFHX3			1
ZFP57					
ZFYVE21			1		
ZKSCAN2					
ZMAT3		ZMAT3			1
ZNF117					
ZNF514					
ZNF536		ZNF536			1
ZNF641		ZNF641	1		
ZNF664					
ZNF728			1		
ZNF737					
ZNF804B					

GC-regulated count:	144	31	239		
Percent total:	33%	7%	54%		
GC-regulated new:	144	15	154	313	Total count
GC-regulated percent:	33%	3%	35%	71%	Total percent

441	Total prioritized target genes

Supplemental Table S9. Prioritized Target Gene Validation
GC-regulated genes from 2 different paired eye studies

Abbreviations and Acronyms
DEG: differentially-expressed gene

Human study: Kathirvel K, Lester K, Haribalaganesh R, Muthukkaruppan V, Lane B, Simpson DA, Goljanek-Whysall K, Sheridan C, Bharanidharan D, Willoughby CE, Senthilkumari S (2022). Short and long-term effect of dexamethasone on the transcriptome profile of primary human trabecular meshwork cells in vitro. Scientific Reports 12: 8299.

Bovine Study: Bermudez JY, Webber HC, Brown B, Braun TA, Clark AF, Mao W (2017). A Comparison of Gene Expression Profiles between Glucocorticoid Responder and Non-Responder Bovine Trabecular Meshwork Cells Using RNA Sequencing. PLoS ONE 12(1): e0169671.

Human Responders All DEGs	Human Responders Down-Regulated DEGs	Human Responders Up-Regulated DEGs	Human Non-Responders All DEGs	Human Non-Responders Down-Regulated DEGs	Human Non-Responders Up-Regulated DEGs	Bovine Responders All DEGs	Bovine Non-Responders All DEGs	All DEGs That Match GWAS Prioritized Genes	Responder DEGs That Match GWAS Prioritized Genes	Non-Responder DEGs That Match GWAS Prioritized Genes	
AATK	AATK	ABCA6	ABCA6	ADM2	ABCA6	AARS	AANAT	ANTXR1	ANTXR1		31 total matches
ABCA6	ABCB1	ABCA9	ACA59	ADRA2A	ACA59	ABL1	ABCA10	ANTXR2	ANTXR2		24 responders
ABCA9	ABI3	ABRA	ADH1A	AQP1	ADH1A	ACAD10	ABCA6	ARHGEF26		ARHGEF26	8 non-responders
ABCB1	ACP5	ACA59	ADH1B	ARHGAP9	ADH1B	ACOT2	ADA	BCL2L11		BCL2L11	(1 overlap)
ABI3	ACPP	ACTG1P3	ADH4	ARSI	ADH4	ACSL5	ADAMTS15	BEND7	BEND7		
ABRA	ADAMTS9-AS1	ADH1A	ADM2	ASPN	ADRA1B	ADAM33	AMIGO1	CCL2	CCL2		
ACA59	ADRA2A	ADH1B	ADRA1B	ATP1A3	AFAP1L1	ADAMTS10	APLN	CDH2		CDH2	
ACP5	ADRB1	ADH4	ADRA2A	BEX2	AIM1	ADAMTS2	APOLD1	CHAC1		CHAC1	
ACPP	AGR2	ADRA1B	AFAP1L1	BST2	AKR1B15	ADAMTSL2	AQPEP	COL11A1	COL11A1		
ACTG1P3	ANO7	AFF2	AIM1	C1orf87	ALDH1L1-AS2	ADCY6	C10H5orf13	COPZ2	COPZ2		
ADAMTS9-AS1	AOC1	ANGPTL1	AKR1B15	C2orf40	ALOX15B	ADRB1	C2	DIAPH3	DIAPH3		
ADH1A	AOC3	ANGPTL5	ALDH1L1-AS2	CA3	ANGPTL1	AFF2	CAPN6	ERGIC1	ERGIC1		
ADH1B	AOC4P	ANGPTL7	ALOX15B	CADM1	ANGPTL5	AGRN	CBS	FRMD3	FRMD3		
ADH4	AP1M2	ANO3	ANGPTL1	CCND2	ANGPTL7	AHCY	CCDC3	FST	FST		
ADRA1B	AQP1	AOX1	ANGPTL5	CCND2-AS1	ANKRD2	AHNAK2	CDH2	GAL	KLHDC7A		
ADRA2A	AQP3	APOD	ANGPTL7	CCND2-AS2	AOX1	AKR1A1	COL14A1	HMCN1	HMCN1		
ADRB1	AQP5	B3GALT2	ANKRD2	CERS1	APCDD1	ALAS1	COL27A1	HS6ST1		HS6ST1	
AFF2	ARSI	BCRP1	AOX1	CHAC1	APOD	ALDH1A3	CPM	HTR2A	HTR2A		
AGR2	ASCL2	BHLHE22	APCDD1	CILP2	ARHGEF26	ALOX12	CRABP1	KCNE4	KCNE4		
ANGPTL1	ATAD3C	C1QTNF7	APOD	CLDN1	B3GNT7	ANGPT1	CSF1	KLHDC7A		KLHDC7A	
ANGPTL5	ATP1A2	C5AR2	AQP1	CNGA1	C3	ANKH	CXCL14	MAPK10	MAPK10		
ANGPTL7	ATP8B4	CCDC54	ARHGAP9	CNTN6	CHI3L2	ANKRD10	CYP4V2	MVB12B	MVB12B		
ANO3	AZGP1	CHRM2	ARHGEF26	COL19A1	CHRNA5	ANP32A	ECE1	PC	PC		
ANO7	B3GAT1	CPM	ARSI	CPA4	CLSTN2	ANPEP	EDN1	PTHLH	PTHLH	PTHLH	
AOC1	B4GALNT3	CYP51A1P2	ASPN	CTNNA3	CNTN1	ANTXR1	EEPD1	RBFOX1	RBFOX1		
AOC3	BCAS1	CYP7B1	ATP1A3	ELOVL2-AS1	CPM	ANTXR2	FAM65C	RBM43	RBM43		
AOC4P	BCL11B	DKK2	B3GNT7	EPHA3	CRISPLD2	ANXA1	FLRT3	SAA4	SAA4		
AOX1	BCL6B	FAM46B	BEX2	ERICH2	DGKG	APEX1	FOXO4	STEAP2	STEAP2		
AP1M2	BMP7	FAM65C	BST2	FNDC1	DKK2	APLP1	FREM2	STON2	STON2		
APOD	BST2	FGD4	C1orf87	FST	DUSP5	APOA1	GABRE	TBX18		TBX18	
AQP1	BTC	FGF14	C2orf40	GRIA2	EDNRB	APOD	GAL	TNK2	TNK2		
AQP3	C10orf128	FGFR4	C3	GRID2	FAM107A	APRT	GCH1				
AQP5	C1orf115	FHL5	CA3	GRM5	FAM150B	AQP1	GPR1				
ARSI	C1orf116	FKBP5	CADM1	IGFL2	FAM46B	ARHGAP42	GREM1				
ASCL2	C1QC	FMO2	CCND2	IL32	FGFR4	ARHGEF25	HMGCS1				
ATAD3C	C1QTNF2	FPR1	CCND2-AS1	INA	FHL5	ARL15	HMOX1				
ATP1A2	C2orf40	FRG2C	CCND2-AS2	IPCEF1	FKBP5	ARL4C	HS6ST1				
ATP8B4	C2orf54	FRMD3	CERS1	KAL1	FMO2	ARSA	HSD11B1				
AZGP1	C3orf80	GALNT15	CHAC1	KCNMB2	FPR1	ARSJ	HTR2A				
B3GALT2	C5orf38	GIP	CHI3L2	KCNS1	GALNT15	ASAP3	IL6				
B3GAT1	C7	GJA5	CHRNA5	KCTD16	GGT5	ATF3	INSIG1				
B4GALNT3	CACNG4	GPX3	CILP2	KIAA1211	GPM6B	ATF5	KCNMA1				
BCAS1	CALML3	H19	CLDN1	KLHDC7B	GPRC5B	ATP10A	KLF15				
BCL11B	CAMK2A	HIF3A	CLSTN2	KRT17	GRK5-IT1	ATP9A	KLF9				
BCL6B	CAMK2B	HNRNPA3P11	CNGA1	KRT17P1	H19	B2M	LNX1				
BCRP1	CAMSAP3	HSPD1P11	CNTN1	KRT23	HEYL	BACE1	LOC100297713				
BHLHE22	CAPN11	IGF2	CNTN6	LAMP3	HIF3A	BACE2	LOC100847212				
BMP7	CASKIN1	ITGA10	COL19A1	LFNG	HLX	BAHCC1	LOC101906324				
BST2	CBFA2T3	KCNE1	CPA4	LINC01133	HMGN2P15	BAI1	LOC507432				
BTC	CBLC	KIAA1456	CPM	LPHN3	IGF2	BAZ2B	LOC518495				
C10orf128	CCDC64B	KLF15	CRISPLD2	LRRC15	IGF2-AS	BBS7	LOC781282				
C1orf115	CCDC88C	KRT18P62	CTNNA3	LRRN4CL	INHBB	BCL2L1	LOC786352				
C1orf116	CCL5	LDHAL6B	DGKG	LTK	ISM1	BCL2L11	LRRN3				
C1QC	CCM2L	LEP	DKK2	MXRA5Y	KIF5C	BDKRB1	LSS				
C1QTNF2	CCR1	LINC00547	DUSP5	MYHAS	LGI3	BDKRB2	M-SAA3.2				
C1QTNF7	CD177	LINC00664	EDNRB	NGEF	LINC00525	BEND6	MGST1				
C2orf40	CD34	LINC00702	ELOVL2-AS1	NGFR	LINC00702	BEND7	MSMO1				
C2orf54	CD38	LINC01088	EPHA3	NMNAT2	LINC00704	BGN	PDE7A				
C3orf80	CD7	LOC100421166	ERICH2	NPY6R	LINC00968	BIRC3	PGM5				
C5AR2	CD74	LSP1	FAM107A	NTM	LINC01088	BMP4	PLSCR4				
C5orf38	CD79B	MAOA	FAM150B	PADI2	LSP1	BOLA-A	PRUNE2				
C7	CDH1	MAP1LC3C	FAM46B	PI16	MAOA	BST2	PTGS1				
CACNG4	CDH22	MARCH10	FGFR4	PLXNC1	METTL7A	BZW2	PTH2R				
CALML3	CDH3	MIR5690	FHL5	PRSS35	MIR5685	C14H8orf47	PTHLH				
CAMK2A	CDH5	MOB3B	FKBP5	RAB39B	MIRLET7D	C19H17orf67	PTX3				
CAMK2B	CEBPA	MRO	FMO2	RARRES2	MOB3B	C1QBP	R3HDML				
CAMSAP3	CEMIP	MYOC	FNDC1	RBFOX1	MREG	C1QTNF3	RAPGEF4				
CAPN11	CERS1	NEDD9	FPR1	RDH12	MRO	C1R	RDH11				
CASKIN1	CFD	NKAIN2	FST	RGS16	MTFP1	C1S	RSPO1				
CBFA2T3	CHGB	NPSR1-AS1	GALNT15	RGS7BP	MTSS1	C27H8orf4	S100A12				
CBLC	CHI3L1	NTRK2	GGT5	RIMS2	MYBPHL	C5H12orf75	S100A9				
CCDC54	CHIT1	OCA2	GPM6B	RIMS3	MYOC	C7H5orf30	S1PR3				
CCDC64B	CHODL	OLAH	GPRC5B	SEMA3D	NCAM1-AS1	C8G	SFRP1				
CCDC88C	CHRDL2	P2RY14	GRIA2	SEMA6B	NEDD9	CA12	SFRP2				
CCL5	CHRM1	PDK4	GRID2	SLC14A1	NR0B1	CALCRL	SLC1A5				
CCM2L	CHRNA2	PDLIM1P4	GRK5-IT1	SLC24A2	NRCAM	CAMK1D	SLC26A2				
CCR1	CHRNA4	PER1	GRM5	SLC7A5	OCA2	CASK	SLC2A3				

CD177	CILP2	PLCE1-AS1		H19	TAC3	OLAH		CASP1	SLC2A5				
CD34	CITED1	PPP1R14A		HEYL	TMEM63C	P2RY14		CAV1	SLC43A2				
CD38	CLCA2	PRODH		HIF3A	TNFSF15	PDK4		CBFA2T3	SPON1				
CD7	CLDN3	PRR33		HLX	TNFSF18	PER1		CCDC102B	SPP1				
CD74	CLDN4	PTK2B		HMGN2P15	TNNT2	PLIN5		CCDC8	SQLE				
CD79B	CLEC14A	RAPGEF5		IGF2	UNC5B	PMEL		CCKAR	SRPX				
CDH1	CNFN	RN7SKP69		IGF2-AS	VCAN	PNMT		CCL2	SULT1B1				
CDH22	COL14A1	RN7SL608P		IGFL2	VCAN-AS1	POM121L9P		CCL5	TBX18				
CDH3	COL15A1	RNA5SP111		IL32	VNN1	PPP1R14A		CCT3	TCP11L2				
CDH5	COL17A1	RPL7P57		INA	WNT2	PRODH		CCT5	TGFBI				
CEBPA	COL9A1	SAA1		INHBB		PRR33		CD274	TRAF3IP2				
CEMIP	COL9A3	SAA2		IPCEF1		PTGDR2		CDH11	TUBB3				
CERS1	CPA3	SAA4		ISM1		PTHLH		CDR2	VCAN				
CFD	CPA4	SAMHD1		KAL1		RAMP2		CGN	WFDC1				
CHGB	CPAMD8	SCN3A		KCNMB2		RAMP2-AS1		CHCHD4	WFDC18				
CHI3L1	CPLX1	SEMG1		KCNS1		RGCC		CHRD	XPNPEP2				
CHIT1	CPNE4	SLC16A10		KCTD16		RN7SKP97		CHRNA7					
CHODL	CPXM1	SLC16A12		KIAA1211		RPA4		CHST2					
CHRDL2	CRABP2	SLC38A11		KIF5C		RPL23AP81		CILP					
CHRM1	CTSH	ST7-AS2		KLHDC7B		SAA1		CMPK2					
CHRM2	CUX2	SYNDIG1		KRT17		SAA2		CNGA3					
CHRNA2	CWH43	TIMP4		KRT17P1		SAMHD1		CNN1					
CHRNA4	CX3CL1	TLDC2		KRT23		SCARA5		COL11A1					
CILP2	CXADR	TNFAIP8L3		LAMP3		SCN3A		COL16A1					
CITED1	CXCL13	TSC22D3		LFNG		SFTPC		COL4A2					
CLCA2	CXCL14	TUSC5		LGI3		SIX2		COL4A3					
CLDN3	CXorf36	UBE2CP1		LINC00525		SLC16A10		COL4A5					
CLDN4	CYBB	USP2		LINC00702		SLC16A12		COL5A1					
CLEC14A	CYP24A1	XRCC6P2		LINC00704		SOAT2		COL5A3					
CNFN	CYP26A1	ZBTB16		LINC00968		SOX13		COL6A2					
COL14A1	CYTH4			LINC01088		SPP1		COL8A2					
COL15A1	DACT2			LINC01133		STAR		COPZ2					
COL17A1	DENND1C			LPHN3		STEAP4		CPE					
COL9A1	DES			LRRC15		STOX1		CPEB4					
COL9A3	DIO3			LRRN4CL		SYN2		CPED1					
CPA3	DIO3OS			LSP1		SYTL4		CPNE3					
CPA4	DIRAS3			LTK		TBXAS1		CPQ					
CPAMD8	DOC2B			MAOA		TIMP4		CRABP2					
CPLX1	DRAXIN			METTL7A		TLDC2		CREB3L1					
CPM	DSC2			MIR5685		TLE2		CREG2					
CPNE4	DTX1			MIRLET7D		TLE6		CRISPLD2					
CPXM1	ECSCR			MOB3B		TMOD1		CRLF3					
CRABP2	EDN3			MREG		TNNT3		CRNN					
CTSH	EFCC1			MRO		TRAV39		CRYAB					
CUX2	EFNA1			MTFP1		TRPC3		CSDA					
CWH43	EHF			MTSS1		TRPV6		CSRP2					
CX3CL1	ELF3			MXRA5Y		TXNRD1		CT					
CXADR	ELFN1			MYBPHL		USP2		CTSS					
CXCL13	ELFN2			MYHAS		VAV3		CXCL3					
CXCL14	ELOVL2			MYOC		WSCD1		CXXC5					
CXorf36	ELOVL7			NCAM1-AS1		XRCC6P2		CYP1B1					
CYBB	EMID1			NEDD9		ZBTB16		CYP27A1					
CYP24A1	ENPP6			NGEF				CYYR1					
CYP26A1	EPCAM			NGFR				DACT1					
CYP51A1P2	ESPN			NMNAT2				DAGLB					
CYP7B1	ESRP1			NPY6R				DBP					
CYTH4	EVPL			NR0B1				DCTPP1					
DACT2	EVX2			NRCAM				DDX27					
DENND1C	EXOC3L4			NTM				DDX58					
DES	FAM110D			OCA2				DEPDC1B					
DIO3	FAM19A3			OLAH				DIAPH3					
DIO3OS	FAM201A			P2RY14				DKC1					
DIRAS3	FAM3B			PADI2				DLG5					
DKK2	FAM3D			PDK4				DMD					
DOC2B	FAM46C			PER1				DNM1					
DRAXIN	FAR2P1			PI16				DSTN					
DSC2	FAR2P2			PLIN5				DTL					
DTX1	FBN3			PLXNC1				DTX3L					
ECSCR	FGR			PMEL				DUSP26					
EDN3	FLT4			PNMT				EDA					
EFCC1	FNDC1			POM121L9P				EFEMP2					
EFNA1	FOLH1			PPP1R14A				EFHD2					
EHF	FOXA1			PRODH				EHD3					
ELF3	FOXQ1			PRR33				EIF4EBP1					
ELFN1	FST			PRSS35				ELMOD1					
ELFN2	FXYD2			PTGDR2				ELTD1					
ELOVL2	FXYD3			PTHLH				EMC2					
ELOVL7	FXYD6			RAB39B				EPAS1					
EMID1	FZD10			RAMP2				EPB41L3					
ENPP6	FZD10-AS1			RAMP2-AS1				EPHB3					
EPCAM	G0S2			RARRES2				EPSTI1					
ESPN	GALNT16			RBFOX1				ERAP2					
ESRP1	GAP43			RDH12				ERGIC1					
EVPL	GATA5			RGCC				ERMP1					
EVX2	GGT6			RGS16				ESCO2					
EXOC3L4	GIMAP1			RGS7BP				ESM1					
FAM110D	GIMAP4			RIMS2				EXOSC5					
FAM19A3	GIMAP5			RIMS3				EXTL1					
FAM201A	GIMAP6			RN7SKP97				F2R					
FAM3B	GIMAP7			RPA4				FADS2					
FAM3D	GIMAP8			RPL23AP81				FAM101B					
FAM46B	GJB1			SAA1				FAM109B					
FAM46C	GJB2			SAA2				FAM114A1					
FAM65C	GLB1L2			SAMHD1				FAM126B					
FAR2P1	GNA15			SCARA5				FAM129A					

FAR2P2	GNG4			SCN3A				FAM155B					
FBN3	GOLT1A			SEMA3D				FAM162A					
FGD4	GP2			SEMA6B				FAM198A					
FGF14	GPR143			SFTPC				FAM20A					
FGFR4	GPR20			SIX2				FAP					
FGR	GPR56			SLC14A1				FBLIM1					
FHL5	GRAMD4P2			SLC16A10				FBXO32					
FKBP5	GREB1			SLC16A12				FERMT2					
FLT4	GREM2			SLC24A2				FGF1					
FMO2	GRHL2			SLC7A5				FGR					
FNDC1	GRIK4			SOAT2				FKBP1A					
FOLH1	GRIN1			SOX13				FKBP4					
FOXA1	GYLTL1B			SPP1				FKBP9					
FOXQ1	HES2			STAR				FMOD					
FPR1	HGD			STEAP4				FOS					
FRG2C	HID1			STOX1				FOSL1					
FRMD3	HLA-DMB			SYN2				FRS2					
FST	HLA-DOA			SYTL4				FTH1					
FXYD2	HLA-DPA1			TAC3				FXYD6					
FXYD3	HLA-DQA1			TBXAS1				GADD45G					
FXYD6	HLA-DQA2			TIMP4				GALNTL4					
FZD10	HLA-DQB1			TLDC2				GAPDH					
FZD10-AS1	HLA-DRA			TLE2				GBP5					
G0S2	HLA-DRB1			TLE6				GDAP1					
GALNT15	HMCN2			TMEM63C				GFRA2					
GALNT16	HMGCS2			TMOD1				GHR					
GAP43	HMGN2P46			TNFSF15				GIPC2					
GATA5	HOTTIP			TNFSF18				GLDC					
GGT6	HOXA10			TNNT2				GLDN					
GIMAP1	HOXA11			TNNT3				GNL3					
GIMAP4	HOXA11-AS			TRAV39				GNPTAB					
GIMAP5	HOXA13			TRPC3				GPCPD1					
GIMAP6	HOXA3			TRPV6				GPR83					
GIMAP7	HOXA5			TXNRD1				GPX3					
GIMAP8	HOXA7			UNC5B				GPX7					
GIP	HOXA9			USP2				GRIA3					
GJA5	HOXB13			VAV3				GRID1					
GJB1	HOXD10			VCAN				HAPLN4					
GJB2	HOXD13			VCAN-AS1				HEXIM2					
GLB1L2	HOXD3			VNN1				HHIPL1					
GNA15	HOXD8			WNT2				HMCN1					
GNG4	HOXD9			WSCD1				HMGA1					
GOLT1A	HPD			XRCC6P2				HPRT1					
GP2	HPN			ZBTB16				HSD17B7					
GPR143	HPSE2							HSD3B1					
GPR20	HSH2D							HSF4					
GPR56	HSPA12B							HSPA5					
GPX3	HSPA6							HSPB6					
GRAMD4P2	ICAM2							HTRA1					
GREB1	IGF1							IFI27					
GREM2	IGHG3							IFI44					
GRHL2	IGHM							IFI44L					
GRIK4	IGHV4-34							IFI6					
GRIN1	IGHV4-39							IFIH1					
GYLTL1B	IGJ							IFRD1					
H19	IGKV1-5							IFRD2					
HES2	IGKV3-11							IGF1					
HGD	IGLC3							IGF2					
HID1	IGLV1-40							IGFALS					
HIF3A	IGSF9							IGLON5					
HLA-DMB	IRF8							IGSF8					
HLA-DOA	IRX4							IGSF9B					
HLA-DPA1	ISL1							IL11RA					
HLA-DQA1	ITGAX							IL27RA					
HLA-DQA2	ITM2A							IMPDH2					
HLA-DQB1	JPH3							INHBA					
HLA-DRA	JPH4							INPP5B					
HLA-DRB1	KCNH2							IRF9					
HMCN2	KCNH6							ISG15					
HMGCS2	KCNK5							ISLR2					
HMGN2P46	KCNN3							ITGA3					
HNRNPA3P11	KCNQ1							ITGA6					
HOTTIP	KIAA1210							ITGBL1					
HOXA10	KIAA1211L							JAG1					
HOXA11	KIAA1324							JDP2					
HOXA11-AS	KIF12							JSP.1					
HOXA13	KIF1A							KANK1					
HOXA3	KL							KCNE4					
HOXA5	KLK11							KCTD15					
HOXA7	KLK2							KDM4A					
HOXA9	KLK3							KIAA0664					
HOXB13	KLK4							KIRREL					
HOXD10	KLK7							KLHDC7A					
HOXD13	KLKP1							KLHL5					
HOXD3	KREMEN2							KPNA2					
HOXD8	KRT13							KPNA3					
HOXD9	KRT14							LAMP5					
HPD	KRT15							LGALS3					
HPN	KRT18							LIMD1					
HPSE2	KRT23							LIMS2					
HSH2D	KRT5							LMNB1					
HSPA12B	KRT6A							LMO4					
HSPA6	KRT79							LMOD1					
HSPD1P11	KRT8							LOC100139000					
ICAM2	LAD1							LOC100298356					

IGF1	LAMP3							LOC100336592					
IGF2	LAMP5							LOC100336728					
IGHG3	LCK							LOC100337226					
IGHM	LCN2							LOC100847951					
IGHV4-34	LCN6							LOC100848012					
IGHV4-39	LCP1							LOC100848407					
IGJ	LGALS7B							LOC100848488					
IGKV1-5	LGR6							LOC100848491					
IGKV3-11	LINC00086							LOC100848673					
IGLC3	LINC00261							LOC100848808					
IGLV1-40	LINC00668							LOC100848869					
IGSF9	LINC00890							LOC100848883					
IRF8	LINC00964							LOC101902032					
IRX4	LINC01018							LOC101902176					
ISL1	LINC01297							LOC101902543					
ITGA10	LINC01315							LOC101904916					
ITGAX	LMAN1L							LOC101905266					
ITM2A	LMO2							LOC101905956					
JPH3	LRG1							LOC101906454					
JPH4	LRRC15							LOC101906941					
KCNE1	LRRC26							LOC101907106					
KCNH2	LYPD3							LOC101908206					
KCNH6	LYZ							LOC505383					
KCNK5	MAL2							LOC507055					
KCNN3	MALL							LOC510798					
KCNQ1	MAOB							LOC511937					
KIAA1210	MARVELD3							LOC512486					
KIAA1211L	MB							LOC515330					
KIAA1324	MCF2L							LOC532442					
KIAA1456	MCHR1							LOC616200					
KIF12	MEOX1							LOC616821					
KIF1A	MEST							LOC618696					
KL	MIR200A							LOC783612					
KLF15	MIR205HG							LOC783680					
KLK11	MIR3189							LOC788176					
KLK2	MIR429							LOC788696					
KLK3	MLC1							LRIG1					
KLK4	MMP7							LRIG3					
KLK7	MMP9							LRRC71					
KLKP1	MPZL2							LRRC8C					
KREMEN2	MS4A6A							LTBP3					
KRT13	MSI1							LURAP1L					
KRT14	MSMB							LY96					
KRT15	MT1G							LYST					
KRT18	MT1H							MADD					
KRT18P62	MXRA5							MANEA					
KRT23	MXRA5Y							MAPK10					
KRT5	MYBPC1							MB21D2					
KRT6A	MYCT1							MCC					
KRT79	MYH14							MCM10					
KRT8	MYL4							MCM7					
LAD1	MYOZ3							MDGA1					
LAMP3	MZB1							MDH2					
LAMP5	NAT8L							MDK					
LCK	NDP							MEF2A					
LCN2	NEFH							MFAP4					
LCN6	NELL2							MFGE8					
LCP1	NFAM1							MFNG					
LDHAL6B	NGEF							MFSD1					
LEP	NGFR							MFSD4					
LGALS7B	NKD2							MICAL3					
LGR6	NKX3-1							MIF					
LINC00086	NKX3-2							MKI67					
LINC00261	NOS3							MLXIPL					
LINC00547	NOSTRIN							MMP3					
LINC00664	NOVA2							MRC2					
LINC00668	NPR1							MROH1					
LINC00702	NPY							MRPL12					
LINC00890	NTF4							MRVI1					
LINC00964	NWD1							MT1A					
LINC01018	NYX							MT3					
LINC01088	OGDHL							MTSS1L					
LINC01297	OR2I1P							MTURN					
LINC01315	OVOL2							MVB12B					
LMAN1L	P2RX1							MX1					
LMO2	P2RX2							MX2					
LOC100421166	PAGE4							MXRA7					
LRG1	PALD1							MXRA8					
LRRC15	PARVG							MYL9					
LRRC26	PCAT18							MYLIP					
LSP1	PCAT4							MYO3A					
LYPD3	PCDH17							MYPN					
LYZ	PCGEM1							NCALD					
MAL2	PCP4							NDRG2					
MALL	PDE2A							NEBL					
MAOA	PDE3B							NEK7					
MAOB	PDE9A							NF2					
MAP1LC3C	PDZK1IP1							NHS					
MARCH10	PECAM1							NIFK					
MARVELD3	PGF							NIPAL3					
MB	PGM5-AS1							NIPAL4					
MCF2L	PHF21B							NME2					
MCHR1	PI15							NOLC1					
MEOX1	PI16							NOTCH3					
MEST	PIGR							NOX4					

MIR200A	PKP1							NOX5					
MIR205HG	PKP3							NPAS3					
MIR3189	PLA2G2A							NPR3					
MIR429	PLA2G4F							NPY1R					
MIR5690	PLA2G7							NQO1					
MLC1	PLCH2							NR4A2					
MMP7	PLVAP							NRIP1					
MMP9	PMCH							NRP1					
MOB3B	POTEH							NSUN2					
MPZL2	PRAC1							NT5C					
MRO	PROK1							NTN1					
MS4A6A	PRR15L							NTN4					
MSI1	PRSS16							NTNG2					
MSMB	PRSS22							NTRK2					
MT1G	PRSS35							NTS					
MT1H	PRSS8							NUAK2					
MXRA5	PTCH2							NUDT4					
MXRA5Y	PTGER1							NXT2					
MYBPC1	PTPN6							OAS1X					
MYCT1	RAB11FIP4							OAS1Y					
MYH14	RAB25							ODC1					
MYL4	RAI2							OLR1					
MYOC	RAMP3							OPLAH					
MYOZ3	RAP1GAP							OXR1					
MZB1	RARRES2							PA2G4					
NAT8L	RASAL3							PARD3B					
NDP	RBBP8NL							PARP10					
NEDD9	RBFOX3							PARP12					
NEFH	RBM47							PARP16					
NELL2	RELN							PARP9					
NFAM1	REM1							PC					
NGEF	RGS16							PCDH18					
NGFR	RGS7BP							PCDHGA2					
NKAIN2	RIC3							PCOLCE					
NKD2	RLN1							PCSK1					
NKX3-1	RNASE1							PCSK1N					
NKX3-2	RNF165							PDE1A					
NOS3	RNF43							PDGFA					
NOSTRIN	ROBO4							PDGFB					
NOVA2	RORC							PDGFD					
NPR1	RPLP0P2							PDGFRB					
NPSR1-AS1	RTN4RL1							PDLIM7					
NPY	RUFY4							PDPN					
NTF4	S100A14							PER2					
NTRK2	S1PR1							PFDN6					
NWD1	SALL3							PGAM1					
NYX	SCGB3A1							PGK1					
OCA2	SDK2							PGRMC2					
OGDHL	SELE							PHACTR2					
OLAH	SELP							PHB					
OR2I1P	SEMA6B							PHF16					
OVOL2	SERPINB11							PI15					
P2RX1	SFN							PIGT					
P2RX2	SFRP2							PIK3R1					
P2RY14	SFRP4							PITPNC1					
PAGE4	SH2D3C							PKD1					
PALD1	SHH							PLA2G12A					
PARVG	SHISA6							PLAC8					
PCAT18	SLC14A1							PLAC9					
PCAT4	SLC2A5							PLAU					
PCDH17	SLC44A4							PLCE1					
PCGEM1	SLC45A3							PLD3					
PCP4	SLC52A3							PLEKHG1					
PDE2A	SLC7A14							PLEKHG5					
PDE3B	SLCO2A1							PLS3					
PDE9A	SMOC1							PNMAL2					
PDK4	SMR3B							PNP					
PDLIM1P4	SORL1							PODNL1					
PDZK1IP1	SOX18							POP1					
PECAM1	SP5							PPAN					
PER1	SP8							PPID					
PGF	SPDEF							PPM1K					
PGM5-AS1	SPINK5							PRDX4					
PHF21B	SPINT1							PRELP					
PI15	SPNS2							PRKAR2B					
PI16	SPOCK3							PRKX					
PIGR	SPRR1B							PROCR					
PKP1	SRD5A2							PROS1					
PKP3	SSTR1							PRRX2					
PLA2G2A	SSTR2							PTGDS					
PLA2G4F	ST14							PTH1R					
PLA2G7	STAB1							PTPN21					
PLCE1-AS1	STAC2							PTPN5					
PLCH2	STC1							PTPRK					
PLVAP	SULT1C4							PTPRN					
PMCH	SULT2B1							PVR					
POTEH	SYNDIG1							PVRL3					
PPP1R14A	SYT13							PXDC1					
PRAC1	SYT17							RAB11FIP4					
PRODH	SYT7							RAB37					
PROK1	SYTL1							RARRES2					
PRR15L	TAC3							RASA3					
PRR33	TAL1							RASGEF1B					
PRSS16	TBX1							RASGRP1					
PRSS22	TBX4							RBM20					

PRSS35	TBX5-AS1							RBM43					
PRSS8	TCEAL2							RCN2					
PTCH2	TENM1							RELB					
PTGER1	TFCP2L1							RESP18					
PTK2B	TFF1							RHBDF2					
PTPN6	TIE1							RHOJ					
RAB11FIP4	TMC5							RIMKLB					
RAB25	TMC6							RMRP					
RAI2	TMC8							RNF213					
RAMP3	TMEFF2							RPL22L1					
RAP1GAP	TMEM125							RPS4Y1					
RAPGEF5	TMEM150C							RPS6KA2					
RARRES2	TMEM179							RRM2					
RASAL3	TMEM63C							RRS1					
RBBP8NL	TMPRSS2							RSAD2					
RBFOX3	TNFSF15							RSL1D1					
RBM47	TNFSF18							RUSC2					
RELN	TNNT2							RXFP4					
REM1	TNRC6C-AS1							S100A10					
RGS16	TNS4							S100A2					
RGS7BP	TP63							SACS					
RIC3	TPD52							SAV1					
RLN1	TPSAB1							SCARA3					
RN7SKP69	TPSB2							SCARF2					
RN7SL608P	TPSD1							SCUBE2					
RNA5SP111	TRGC1							SDC2					
RNASE1	TRIM29							SEC14L5					
RNF165	TRPM8							SEL1L					
RNF43	TRPV6							SELV					
ROBO4	TSPAN1							SEMA3D					
RORC	TSPAN7							SEMA7A					
RPL7P57	TTC22							SERTAD4					
RPLP0P2	TYROBP							SETBP1					
RTN4RL1	UPK3A							SFXN1					
RUFY4	VAMP8							SGCD					
S100A14	VENTX							SH3BGR					
S1PR1	VIPR1							SH3BP4					
SAA1	VSTM2A							SIAH1					
SAA2	VWA1							SIGMAR1					
SAA4	VWF							SIK1					
SALL3	WFDC2							SIPA1L2					
SAMHD1	WNK2							SLC1A3					
SCGB3A1	WNT10A							SLC22A15					
SCN3A	WNT10B							SLC25A5					
SDK2	WNT11							SLC29A1					
SELE	WNT2							SLC2A1					
SELP	WNT4							SLC2A4					
SEMA6B	WNT6							SLC38A4					
SEMG1	WNT7B							SLC38A5					
SERPINB11	WSCD2							SLC40A1					
SFN	ZDHHC8P1							SLC41A2					
SFRP2	ZMYND15							SLC4A3					
SFRP4	ZNF385C							SLC5A6					
SH2D3C								SLC9A9					
SHH								SLCO2A1					
SHISA6								SLCO4A1					
SLC14A1								SLFN11					
SLC16A10								SLIT3					
SLC16A12								SMAD7					
SLC2A5								SMOC1					
SLC38A11								SMPD1					
SLC44A4								SMS					
SLC45A3								SNAP91					
SLC52A3								SNRPB					
SLC7A14								SOCS1					
SLCO2A1								SORT1					
SMOC1								SPATS2L					
SMR3B								SPRYD7					
SORL1								SQRDL					
SOX18								SRD5A1					
SP5								SRGAP3					
SP8								SRPK1					
SPDEF								SRPK3					
SPINK5								ST5					
SPINT1								STEAP2					
SPNS2								STIM2					
SPOCK3								STK38L					
SPRR1B								STON2					
SRD5A2								SURF1					
SSTR1								SUSD5					
SSTR2								SWAP70					
ST14								SYNJ2					
ST7-AS2								SYTL2					
STAB1								SYTL4					
STAC2								TANC2					
STC1								TBC1D32					
SULT1C4								TBC1D9					
SULT2B1								TCEA3					
SYNDIG1								TCF7					
SYT13								TCN2					
SYT17								TFRC					
SYT7								TGFB1I1					
SYTL1								TGFB2					
TAC3								TGFB3					
TAL1								TGFBR2					

TBX1								TGFBR3					
TBX4								THOP1					
TBX5-AS1								THPO					
TCEAL2								THRB					
TENM1								THY1					
TFCP2L1								TIMP1					
TFF1								TIMP2					
TIE1								TIMP3					
TIMP4								TMCC3					
TLDC2								TMEM241					
TMC5								TMEM41B					
TMC6								TMEM45A					
TMC8								TMEM63A					
TMEFF2								TMEM8B					
TMEM125								TMTC1					
TMEM150C								TMTC2					
TMEM179								TNC					
TMEM63C								TNFAIP8					
TMPRSS2								TNFRSF25					
TNFAIP8L3								TNK2					
TNFSF15								TNXB					
TNFSF18								TPI1					
TNNT2								TRANK1					
TNRC6C-AS1								TRPA1					
TNS4								TSPAN9					
TP63								TUBA1C					
TPD52								TUBA4A					
TPSAB1								TULP4					
TPSB2								TXNDC17					
TPSD1								TXNRD1					
TRGC1								UACA					
TRIM29								UBA7					
TRPM8								UCN2					
TRPV6								USP31					
TSC22D3								VASN					
TSPAN1								VAV3					
TSPAN7								VLDLR					
TTC22								VOPP1					
TUSC5								WBP1					
TYROBP								WDR35					
UBE2CP1								WTIP					
UPK3A								WWP2					
USP2								XAF1					
VAMP8								YBX2					
VENTX								YOD1					
VIPR1								YPEL2					
VSTM2A								ZBP1					
VWA1								ZFP36L1					
VWF								ZNF271					
WFDC2								ZNF385B					
WNK2								ZNF423					
WNT10A								ZNF488					
WNT10B								ZNF521					
WNT11								ZNHIT1					
WNT2													
WNT4													
WNT6													
WNT7B													
WSCD2													
XRCC6P2													
ZBTB16													
ZDHHC8P1													
ZMYND15													
ZNF385C													

Supplemental Table S10. Prioritized Target Gene Validation
Paired eye study matches with prioritized target genes

Notes
These studies used paired human donor eyes, or paired eyes from bovine donors as described in the text.
Gene functions are from GeneCards.
* indicates genes associated with a SNP of genome-wide significance identified in this study

Species	Gene	Log2 Fold change	Function
**Genes differentially regulated in TM cells of steroid responders**			
Bovine	ANTXR1	-1.06	Cell surface receptor with role in pathological angiogenesis
Bovine	ANTXR2	-1.07	Cell surface receptor with role in vascular development
Bovine	*BEND7	-1.11	Transcription factor with unknown role
Bovine	CCL2	-1.00	Cytokine ligand for C-C chemokine receptor CCR2; inflammation
Bovine	*COL11A1	-1.63	Collagen XI subunit involved in regulation of fibrillogenesis
Bovine	COPZ2	-0.83	Adaptor for COPI-1 mediated Golgi-ER transport
Bovine	DIAPH3	1.34	Assembly of F-actin structures (Formin family)
Bovine	ERGIC1	1.00	Endoplasmic reticulum-golgi intermediate compartment (ERGIC) protein
Human	FRMD3	2.21	Unknown
Human	FST	-2.08	Activin antagonist (TGFB superfamily member)
Bovine	HMCN1	-1.54	Multifunctional (Hemicentin)
Bovine	KCNE4	-1.83	Voltage-gated potassium channel, delayed rectifier (regulates KCNQ1)
Bovine	KLHDC7A	2.16	Unknown
Bovine	MAPK10	-0.84	Member of the MAP kinase family; signal transduction
Bovine	MFSD1	-0.72	Recycles lysosomal proteolysis products
Bovine	MVB12B	-0.82	Component of ESCRT-I complex that regulates vesicular trafficking
Bovine	PC	-0.77	Pyruvate carboxylase
Bovine	RBM43	-0.97	Predicted to enable RNA binding activity
Human	SAA4	-2.34	Serum amyloid A; redicted to be involved in acute-phase response
Bovine	STEAP2	0.77	Metalloreductase
Bovine	STON2	-1.55	Regulates vesicle-mediated transport
Bovine	TNK2	-0.83	Non-receptor protein kinase, downstream effector of CDC42
**Genes differentially regulated in TM cells of steroid non-responders**			
Human	*ARHGEF26	2.08	Rho-guanine nucleotide exchange factor (RhoG)
Bovine	BCL2L11	-1.46	Member of BCL-2 protein family of apoptosis regulators
Bovine	CDH2	-0.95	Cell-cell adhesion (N-cadherin)
Human	CHAC1	-2.43	Enzymatic inhibition of Notch (g-glutamylcyclotransferase family)
Human	FST	-2.08	TGFB superfamily inhibitor (follistatin)
Bovine	GAL	-2.26	Neuroendocrine peptide that controls smooth muscle contraction
Bovine	HS6ST1	-0.92	Enzyme that modifies heparan sulfate
Bovine	HTR2A	1.93	Serotonin receptor that reduces IOP when activated
Bovine	PTHLH	-2.50	Parathyroid hormone-like hormone
Human	PTHLH	2.42	Parathyroid hormone-like hormone
Human	*RBFOX1	-3.80	Regulates alternative RNA splicing
Bovine	TBX18	1.12	Transcriptional repressor

4 A. Celada *et al.* , The transcription factor PU.1 is involved in macrophage proliferation. *The Journal of experimental medicine* **184**, 61-69 (1996).
5 D. Beck *et al.* , PU.1 eviction at lymphocyte-specific chromatin domains mediates glucocorticoid response in acute lymphoblastic leukemia. Nature communications 15, 9697 (2024).
6 K. M. Harlen, L. S. Churchman, The code and beyond: transcription regulation by the RNA polymerase II carboxy-terminal domain. Nature reviews. Molecular cell biology 18, 263-273 (2017).
7 S. Aittomaki *et al.* , Cooperation among Stat1, glucocorticoid receptor, and PU.1 in transcriptional activation of the high-affinity Fc gamma receptor I in monocytes. Journal of immunology 164, 5689-5697 (2000).
8 D. Langlais, C. Couture, A. Balsalobre, J. Drouin, The Stat3/GR interaction code: predictive value of direct/indirect DNA recruitment for transcription outcome. Molecular cell 47, 38-49 (2012).

Supplemental Table S11. Prioritized Target Gene Validation
GC-regulation in silico and other upstream regulators

Notes
As analyzed using NIH Database for Annotation, Visualization and Integrated Discovery (DAVID) Bioinformatics functional annotation clustering tool paired with the UCSF_TFBS database
Abbreviations and Acronyms
DEG: differentially-expressed gene; GC: glucocorticoid; GR: glucocorticoid receptor; TF: transcription factor

GWAS: 441 genes submitted **436 DAVID IDs** **Prioritized Target Genes** UCSF_TFBS

Classification stringency: Low
Annotation Cluster 1 (top score) Enrichment score: 9.43

Transcription Factor	Gene #	% of DAVID IDs	P value	Benjamini-corrected	Notes
GR	239	54.69	7.9E-08	1.5E-07	Glucocorticoid receptor

Classification stringency: Medium
Annotation Cluster 1 (top score) Enrichment score: 22.32 there were a total of 2 annotation clusters

Transcription Factor	Gene #	% DAVID IDs	P value	Benjamini-corrected	Notes
LHX3	236	54	3.84E-28	3.40E-26	LIM domain TF
HLF	239	54.69	7.68E-23	9.07E-22	PAR bZIP TF
CHX10	231	52.86	3.77E-18	2.15E-17	Homeobox TF

Paired Eye: 618 genes submitted **590 DAVID IDs** **All Responder DEGs** UCSF_TFBS
Human Paired Eye Study

Classification stringency: Low
Annotation Cluster 1 (top score) Enrichment score: 3.42

TF	Gene #	% DAVID IDs	P value	Benjamini-corrected	Notes
GR	264	0.45	8.12E-03	2.77E-02	Glucocorticoid receptor

Classification stringency: Medium
Single Annotation Cluster Enrichment score: 1.46

Transcription Factor	Gene #	% DAVID IDs	P value	Benjamini-corrected	Notes
S8	244	0.41	1.95E-04	1.91E-03	Paired domain TF, also known as Prx2 or Prrxl1
LHX3	182	0.31	4.68E-02	1.05E-01	LIM domain TF
CHX10	196	0.33	1.75E-01	2.68E-01	Homeobox TF
CART1	200	0.34	8.96E-01	9.67E-01	Paired-like homeodomain TF, Encoded by ALX4

Supplementary Table S12. Top Prioritized Target Genes Additional Validation
Summary of chromatin analysis for SNPs that cluster in the top 29 risk loci

Notes
The analysis was performed on RegulomeDB and HaploReg v4.2
Abreviations and Acronyms
AOP: aqueous outflow pathways; caQTL: chromatin accessibility quantitative trait locus; GR: glucocorticoid receptor; LD: linkage disequilibrium; SiPhy cons: Site Phylogenetic Conservation

Prioritized Gene	Hit SNP	SNP Significance	Genome-wide significance hit SNP in LD at R^2=0.4	SNP Significance	Motifs Changed	Co-localizes with	Organ or cell type used for chIP-seq when information is available	AOP cell types where transcription factors listed are primarily expressed (as assessed on Spectacle)
**Features of active chromatin**								
HDAC9	rs74455595	Genome-level				caQTL	lymphoblast cell line	
NCAM2	rs75024143	Suggestive				SiPhy cons		
SPTY2D1	rs151115079	Genome-wide				SiPhy cons		
**Chip-identified binding proteins indicating active transcription**								
BEND7	rs184425183	Genome-wide				CCNT2	bodily fluid, blood, K562	All cell types
GPLD1	rs150586237	Genome-wide				CTCF	bodily fluid, blood, endocrine gland, epithelium, liver, exocrine gland, uterus, mammary gland, exocrine gland, esophagus, skin of body, K562, HepG2, HeLa-S3, MCF-7, GM12873, epithelial cell of esophagus, keratinocyte, SU-DHL-6, Loucy, right lobe of liver, GM23338	All cell types
SPTY2D1	rs151115079	Genome-wide				SiPhy cons		All cell types
SPTY2D1	rs151115079	Genome-wide				POL2		All cell types
SPTY2D1	rs151115079	Genome-wide				TAF1		All cell types
**TF binding motif**								
AGAP1	rs188848340	Suggestive	rs147559909	Genome-wide	GR binding motif			All cell types
COL11A1	rs140420703	Suggestive			GR binding motif			All cell types
CCR6	rs184487573	Suggestive	rs148153037	Genome-wide	GR binding motif			All cell types
GPLD1	Multiple non-hits		rs150586237	Genome-wide	GR binding motif			All cell types
HDAC4	rs188076929				GR binding motif			All cell types
PPM1H	rs182437250	Suggestive			GR binding motif			All cell types
TRHDE	rs112796175	Suggestive			GR binding motif			All cell types
**Chip-identified binding proteins known to interact with the GR**								
HDAC4	rs188076929	Genome-wide				JUND	endocrine gland, epithelium, liver, exocrine gland, HepG2	All cell types
HDAC9	rs10279777	Genome-wide				Pu-1, encoded by *SPI1*	bodily fluid, blood, GM12891, HL-60	Macrophages, ciliary muscle cells, neurons
SPTY2D1	rs138414342	Genome-wide				STAT1, STAT4, STAT5A, STAT5B	esophagus squamous epithelium, inflammatory macrophage, motor neuron suppressor macrophage	All cell types
SPTY2D1	rs151115079	Genome-wide				Oct-2, encoded by *POU2F2*		macrophages, ciliary muscle cells, neurons
SPTY2D1	rs151115079	Genome-wide				HEY1		TM, vascular endothelium, Schwann cells

KEY

caQTL:	[illegible] by the Assay for Transposase-Accessible Chromatin (ATAC-seq). Chromatin accessibility is a reliable indicator of local cis-regulatory activity (1).
CCNT2:	A transcription factor that is a regulatory subunit of the positive transcription elongation factor B (P-TEFb) complex. P-TEFb is essential for RNA polymerase II's elongation of transcription and co-transcriptional processing.
CTCF:	An eleven zinc finger (ZF), multivalent transcriptional regulator that organizes chromatin.
JUND:	A subunit of AP1, which can act as a pioneer factor to prime GR binding.
HEY1:	A TF target of the NOTCH signaling pathway. GCs inhibit HEY1 expression (2).
Oct-2:	Encoded by POU2F2. The GR interacts physically with POU2F2 to modulate transcription (3).
Pu-1:	Also known as Pu.1, a tissue-specific TF of the hematopoietic lineage (4) encoded by SPI1. GR binding elements in DNA are primed by Pu-1, which interacts with the GR (5).
RNA pol II:	RNA polymerase II, an enzyme responsible for transcribing genes that encode proteins, as well as some non-coding RNA genes (6).
SiPhy Cons:	A measure of evolutionary conservation based on alignment of 17 vertebrate species used by HaploregV.2. Conservation suggests function importance of these loci.
STAT1:	A transcription factor. the GR does not physically bind STAT1, however an indirect mechanism of cross-modulation has been demonstrated that integrates STAT1 and the GR with PU.1 (7).
STAT3:	A transcription factor. STAT3 interacts with the GR reciprocally by tethering, i.e., GR tethering to DNA-bound STAT3 results in transcriptional repression, whereas STAT3 tethering to GR results in synergism (8).
TBP:	Also called TATA binding protein, a component of the transcription initiation machinery.
TAF1:	A TBP-associated factor.

REFERENCES

1 J. D. Buenrostro, B. Wu, H. Y. Chang, W. J. Greenleaf, ATAC-seq: A Method for Assaying Chromatin Accessibility Genome-Wide. Curr Protoc Mol Biol 109, 21 29 21-21 29 29 (2015).
2 S. Zanotti, J. Yu, S. Adhikari, E. Canalis, Glucocorticoids inhibit notch target gene expression in osteoblasts. *Journal of cellular biochemistry* **119**, 6016-6023 (2018).
3 L. D. Ward, M. Kellis, HaploReg: a resource for exploring chromatin states, conservation, and regulatory motif alterations within sets of genetically linked variants. Nucleic acids research 40, D930-934 (2012).

4 A. Celada *et al.* , The transcription factor PU.1 is involved in macrophage proliferation. *The Journal of experimental medicine* **184**, 61-69 (1996).
5 D. Beck *et al.* , PU.1 eviction at lymphocyte-specific chromatin domains mediates glucocorticoid response in acute lymphoblastic leukemia. Nature communications 15, 9697 (2024).
6 K. M. Harlen, L. S. Churchman, The code and beyond: transcription regulation by the RNA polymerase II carboxy-terminal domain. Nature reviews. Molecular cell biology 18, 263-273 (2017).
7 S. Aittomaki *et al.* , Cooperation among Stat1, glucocorticoid receptor, and PU.1 in transcriptional activation of the high-affinity Fc gamma receptor I in monocytes. Journal of immunology 164, 5689-5697 (2000).
8 D. Langlais, C. Couture, A. Balsalobre, J. Drouin, The Stat3/GR interaction code: predictive value of direct/indirect DNA recruitment for transcription outcome. Molecular cell 47, 38-49 (2012).

Supplementary Table S13. GWAS Results Indianapolis-2 Replication Cohort
12 month quantitative trait (QT), P-value ordered

Notes
60 SNPs Total
Standard Headers
rsID: reference SNP cluster ID, chr_38: chromosome number; pos_38, position of SNP on GRCh38 reference panel; Imputation_Rsq; estimate of the squared correlation between imputed and true genotypes;
REF and ALT, reference allele and alternate allele; n.obs: number of observations; caf: coding allele frequency; MAC: minor allele count; Score: p-values from Score test; Score.SE: standard error of the score statistic;
Score.Stat: computed score statistic based on the derivative of the log-likelihood function; Score.pval: probability value associated with the Score.Stat; EST: estimated effect size; EST.SE: standard error of the effect size;
Func.refGene: SNP location with respect to nearest gene; Gene.refGene: closet gene(s) upstream and downstream; GeneDetail.refGene: distance of SNP to nearest gene(s)
Added Headers
QT: quantitative trait
Abbreviations and Acronyms
3M: 3 month; 12M: 12 month; AOP:aqueous out flow pathway

QT	rsID	chr_38	POS_38	freq	MAC	Rsq	Score	Score.SE	Score.Stat	Score.pval	Func.refGene	Gene.refGene	GeneDetail.refGene
12M	rs74649788	chr18	6045906	0.049893204	10	0.923243	2.439599988	0.449001371	5.43339095	5.52931E-08	intronic	L3MBTL4	.
12M	rs74833295	chr13	30194287	0.029165049	6	0.9983	1.909228275	0.360021719	5.303091934	1.13858E-07	intergenic	LINC00365;KATNAL1	dist=85412;dist=8343
12M	rs73150883	chr3	146330831	0.023815534	5	0.848249	1.481987222	0.287749854	5.150262284	2.60122E-07	intergenic	PLSCR4;PLSCR2	dist=79652;dist=102457
12M	rs189490695	chr8	23685209	0.016383495	3	0.874937	1.2982161	0.255687499	5.077354608	3.82726E-07	intergenic	NKX3-1;NKX2-6	dist=2272;dist=17242
12M	rs539324287	chr8	23605195	0.016941748	3	0.907302	1.341800652	0.264601135	5.071031363	3.95666E-07	intergenic	SLC25A37;NKX3-1	dist=32645;dist=73498
12M	rs113199364	chr17	77161818	0.033300971	7	0.893794	1.815437814	0.358406655	5.065301631	4.07754E-07	intronic	SEC14L1	.
12M	rs185293133	chr3	75433381	0.011626214	2	0.492536	0.80016771	0.159019081	5.031897471	4.85649E-07	ncRNA_intronic	FAM86DP	.
12M	rs58503971	chr18	6049552	0.044349515	9	0.959099	2.087223432	0.417798884	4.995761149	5.86042E-07	intronic	L3MBTL4	.
12M	rs79381542	chr18	6049027	0.044359223	9	0.959092	2.087445553	0.417849208	4.995691057	5.86254E-07	intronic	L3MBTL4	.
12M	rs114241096	chr5	116644156	0.029092233	6	0.998458	1.782650366	0.361233296	4.934900476	8.01916E-07	intergenic	SEMA6A;LOC102467223	dist=69230;dist=99146
12M	rs114587121	chr5	116644342	0.029092233	6	0.998458	1.782650366	0.361233296	4.934900476	8.01916E-07	intergenic	SEMA6A;LOC102467223	dist=69416;dist=98960
12M	rs79516960	chr5	116645028	0.029097087	6	0.998286	1.782403345	0.361231089	4.934246796	8.04606E-07	intergenic	SEMA6A;LOC102467223	dist=70102;dist=98274
12M	rs78085382	chr5	116645205	0.029097087	6	0.998286	1.782403345	0.361231089	4.934246796	8.04606E-07	intergenic	SEMA6A;LOC102467223	dist=70279;dist=98097
12M	rs79794218	chr5	116645540	0.029097087	6	0.998286	1.782403345	0.361231089	4.934246796	8.04606E-07	intergenic	SEMA6A;LOC102467223	dist=70614;dist=97762
12M	rs114108437	chr5	116646294	0.029097087	6	0.998286	1.782403345	0.361231089	4.934246796	8.04606E-07	intergenic	SEMA6A;LOC102467223	dist=71368;dist=97008
12M	rs76006987	chr5	116656217	0.029087379	6	0.998629	1.782276135	0.361233864	4.933856736	8.06216E-07	intergenic	SEMA6A;LOC102467223	dist=81291;dist=87085
12M	rs77857559	chr5	116652484	0.029126214	6	1	1.784300998	0.361724673	4.932759998	8.10758E-07	intergenic	SEMA6A;LOC102467223	dist=77558;dist=90818
12M	rs115917626	chr5	116653147	0.029126214	6	1	1.784300998	0.361724673	4.932759998	8.10758E-07	intergenic	SEMA6A;LOC102467223	dist=78221;dist=90155
12M	rs148654242	chr13	52129747	0.036063107	7	0.890541	1.896523445	0.386307062	4.90936778	9.13705E-07	ncRNA_exonic	LOC101929657	.
12M	rs76433992	chr5	116641213	0.029834951	6	0.974211	1.764770451	0.360470372	4.895743415	9.79348E-07	intergenic	SEMA6A;LOC102467223	dist=66287;dist=102089
12M	rs138665118	chr2	49835636	0.030864078	6	0.892953	1.721021507	0.356977463	4.821092882	1.42774E-06	intergenic	FSHR;NRXN1	dist=681108;dist=82869
12M	rs116626718	chr5	163226090	0.02438835	5	0.995221	1.593867941	0.333478221	4.779526333	1.75709E-06	intergenic	GABRG2;CCNG1	dist=1070551;dist=211481
12M	rs28798228	chr2	126155850	0.033174757	7	0.961063	1.808450116	0.378512536	4.777781303	1.7724E-06	intergenic	CNTNAP5;GYPC	dist=1240473;dist=500084
12M	rs12621729	chr2	74255421	0.032208738	7	0.942493	1.780987998	0.373137772	4.77300378	1.81498E-06	intronic	SLC4A5	.
12M	rs115331238	chr5	163219712	0.024461165	5	0.983743	1.583713858	0.331858007	4.772263512	1.82167E-06	intergenic	GABRG2;CCNG1	dist=1064173;dist=217859
12M	rs72705714	chr15	27391807	0.067961165	14	1	2.448622014	0.514411414	4.760046044	1.93549E-06	intronic	GABRG3	.
12M	rs72705712	chr15	27391241	0.067961166	14	0.998164	2.444243265	0.513892292	4.756333777	1.9714E-06	intronic	GABRG3	.
12M	rs72705715	chr15	27392048	0.067961166	14	0.998164	2.444243265	0.513892292	4.756333777	1.9714E-06	intronic	GABRG3	.
12M	rs72705711	chr15	27390210	0.067970874	14	0.997858	2.443670474	0.513826908	4.75582426	1.97638E-06	intronic	GABRG3	.
12M	rs28796094	chr2	126197637	0.032441747	7	0.939443	1.7618909	0.370731447	4.752472211	2.00944E-06	intergenic	CNTNAP5;GYPC	dist=1282260;dist=458297
12M	rs17206867	chr7	9449981	0.019781553	4	0.886061	1.317750936	0.277733732	4.744655705	2.08861E-06	intergenic	NXPH1;PER4	dist=697018;dist=184289
12M	rs75968509	chr15	31090915	0.019359223	4	0.979554	1.431828899	0.302121941	4.739241691	2.1452E-06	intronic	TRPM1	.
12M	rs114008181	chr5	163234729	0.025213592	5	0.958521	1.5722456	0.331875986	4.73744913	2.16425E-06	intergenic	GABRG2;CCNG1	dist=1079190;dist=202842
12M	rs72705708	chr15	27388838	0.068470875	14	0.990527	2.432058671	0.513440775	4.736785214	2.17135E-06	intronic	GABRG3	.
12M	rs116189826	chr5	163202772	0.024538835	5	0.937899	1.5321512	0.32364261	4.734083693	2.20047E-06	intergenic	GABRG2;CCNG1	dist=1047233;dist=234799

12M	rs72705709	chr15	27389069	0.068349515	14	0.992399	2.429618929	0.513388602	4.732514355	2.21756E-06	intronic	GABRG3	.
12M	rs72705710	chr15	27389259	0.068320389	14	0.992701	2.428373305	0.513251522	4.731351398	2.2303E-06	intronic	GABRG3	.
12M	rs74591804	chr8	132987195	0.014558252	3	0.999662	1.200642195	0.254591569	4.715954269	2.4058E-06	intronic	TG	.
12M	rs111621289	chr12	10893543	0.03042233	6	0.810976	1.581110295	0.336660743	4.696449854	2.64722E-06	ncRNA_intronic	PRH1-PRR4	.
12M	rs148294287	chr6	126169178	0.016917476	3	0.861892	1.203746268	0.259271271	4.642806213	3.43709E-06	intergenic	MIR5695;CENPW	dist=46562;dist=170611
12M	rs9380234	chr6	31280791	0.024305825	5	0.995714	1.53280058	0.332764823	4.606257864	4.0998E-06	intergenic	HLA-C;HLA-B	dist=8655;dist=73081
12M	rs41544614	chr6	31271024	0.024349515	5	0.994693	1.533218592	0.332869958	4.606058777	4.10372E-06	intronic	HLA-C	.
12M	rs142006494	chr6	158568942	0.015582524	3	0.909469	1.102833648	0.239463028	4.60544434	4.11586E-06	intronic	TMEM181	.
12M	rs9366775	chr6	31272319	0.024271845	5	1	1.534745688	0.333295989	4.604752947	4.12956E-06	upstream	HLA-C	dist=183
12M	rs9357121	chr6	31272702	0.024271845	5	1	1.534745688	0.333295989	4.604752947	4.12956E-06	upstream	HLA-C	dist=566
12M	rs9391714	chr6	31277303	0.024271845	5	1	1.534745688	0.333295989	4.604752947	4.12956E-06	intergenic	HLA-C;HLA-B	dist=5167;dist=76569
12M	rs56356836	chr6	31279490	0.024237864	5	0.998566	1.532381559	0.332833918	4.60404267	4.14368E-06	intergenic	HLA-C;HLA-B	dist=7354;dist=74382
12M	rs9405016	chr6	31280221	0.024237864	5	0.998566	1.532381559	0.332833918	4.60404267	4.14368E-06	intergenic	HLA-C;HLA-B	dist=8085;dist=73651
12M	rs12529015	chr6	31280485	0.024237864	5	0.998566	1.532381559	0.332833918	4.60404267	4.14368E-06	intergenic	HLA-C;HLA-B	dist=8349;dist=73387
12M	rs9368669	chr6	31280716	0.024237864	5	0.998566	1.532381559	0.332833918	4.60404267	4.14368E-06	intergenic	HLA-C;HLA-B	dist=8580;dist=73156
12M	rs17198734	chr6	31288249	0.024237864	5	0.998566	1.532381559	0.332833918	4.60404267	4.14368E-06	intergenic	HLA-C;HLA-B	dist=16113;dist=65623
12M	rs17192386	chr6	31288281	0.024237864	5	0.998566	1.532381559	0.332833918	4.60404267	4.14368E-06	intergenic	HLA-C;HLA-B	dist=16145;dist=65591
12M	rs1050276	chr6	31270291	0.024432039	5	0.991508	1.532787887	0.332980302	4.603238922	4.15971E-06	exonic	HLA-C	.
12M	rs29029490	chr6	31271950	0.024334952	5	0.991645	1.529466801	0.332272783	4.603045682	4.16357E-06	intronic	HLA-C	.
12M	rs4298345	chr6	31273041	0.024203884	5	0.997144	1.529478709	0.332384994	4.601527555	4.19404E-06	upstream	HLA-C	dist=905
12M	rs6583185	chr3	196898971	0.97179126	6	0.853106	-1.52139796	0.331189308	-4.593741177	4.35369E-06	intronic	SENP5	.
12M	rs114500719	chr5	163069412	0.028330097	6	0.909911	1.557183414	0.34021336	4.577078964	4.71514E-06	intergenic	GABRG2;CCNG1	dist=913873;dist=368159
12M	rs189440995	chr2	133887028	0.014786408	3	0.899627	1.137571373	0.248782585	4.572552272	4.81819E-06	intergenic	NCKAP5;MIR3679	dist=318568;dist=240097
12M	rs77364739	chr10	71030533	0.038834951	8	1	1.881493073	0.411573362	4.571464641	4.84327E-06	intergenic	PCBD1;UNC5B	dist=141747;dist=182002
12M	rs72678487	chr4	124461029	0.019456311	4	0.987952	1.343334873	0.29403913	4.568558189	4.91091E-06	intergenic	LINC01091;LOC101927087	dist=530666;dist=38913

Supplementary Table S14. GWAS Results Indianapolis-2 Replication Cohort
3 month quantitative trait (QT), p-value ordered

Notes

102 SNPs Total

Standard Headers

rsID: reference SNP cluster ID, chr_38: chromosome number; pos_38, position of SNP on GRCh38 reference panel; Imputation_Rsq; estimate of the squared correlation between imputed and true genotypes; REF and ALT, reference allele and alternate allele; n.obs: number of observations; caf: coding allele frequency; MAC: minor allele count; Score: p-values from Score test; Score.SE: standard error of the score statistic; Score.Stat: computed score statistic based on the derivative of the log-likelihood function; Score.pval: probability value associated with the Score.Stat; EST: estimated effect size; EST.SE: standard error of the effect size; Func.refGene: SNP location with respect to nearest gene; Gene.refGene: closet gene(s) upstream and downstream; GeneDetail.refGene: distance of SNP to nearest gene(s)

Added Headers

QT: quantitiative trait

Abbreviations and Acronyms

3M: 3 month; 12M: 12 month; AOP:aqueous out flow pathway

QT	rsID	Chr_38	chr_382	freq	MAC	Rsq	Score	Score.SE	Score.Stat	Score.pval	Func.refGene	Gene.refGene	GeneDetail.refGene
3M	rs9592740	13	chr13	0.066820388	14	0.9795	3.693411	0.700908722	5.269460143	1.36826E-07	intergenic	ATXN8OS;LINC00348	dist=514581;dist=360807
3M	rs150977401	10	chr10	0.01631068	3	0.8708	1.860675	0.358119043	5.195689556	2.03962E-07	intronic	CASP7	.
3M	rs9599709	13	chr13	0.065	13	0.9585	3.560256	0.685315257	5.195063146	2.0465E-07	intergenic	ATXN8OS;LINC00348	dist=528543;dist=346845
3M	rs4337430	2	chr2	0.248378641	51	0.973	6.84431	1.325444312	5.163785781	2.42005E-07	intergenic	OC151121;LOC3890	dist=532066;dist=116905
3M	rs145531574	2	chr2	0.013723301	3	0.8698	1.731905	0.335798226	5.157575737	2.50168E-07	intergenic	RND3;LOC10192926	dist=3372;dist=61465
3M	rs143862437	15	chr15	0.014752427	3	0.9521	1.816785	0.35534236	5.112774488	3.17461E-07	intergenic	RORA;VPS13C	dist=10193;dist=612895
3M	rs182462684	15	chr15	0.014762136	3	0.9515	1.816595	0.35534099	5.112258325	3.1833E-07	intergenic	RORA;VPS13C	dist=13323;dist=609765
3M	rs61754236	12	chr12	0.028985437	6	0.9951	2.517384	0.494187455	5.093985122	3.50614E-07	exonic	NAV3	.
3M	rs2014160	12	chr12	0.01511165	3	0.8537	1.729592	0.339602326	5.092992566	3.52456E-07	intronic	WNK1	.
3M	rs140686116	12	chr12	0.02904369	6	0.9971	2.519911	0.495194249	5.088732794	3.60464E-07	intronic	NAV3	.
3M	rs138697513	8	chr8	0.030781554	6	0.8843	2.577315	0.506628696	5.087187008	3.63413E-07	intergenic	IDO2;C8orf4	dist=71007;dist=66070
3M	rs145892996	8	chr8	0.030776699	6	0.8841	2.576606	0.50654249	5.086652731	3.64438E-07	intergenic	IDO2;C8orf4	dist=70873;dist=66204
3M	rs34017523	2	chr2	0.253538835	52	0.9789	6.708705	1.334874447	5.025719568	5.01548E-07	intergenic	OC151121;LOC3890	dist=533874;dist=115097
3M	rs6720381	2	chr2	0.253538835	52	0.9789	6.708705	1.334874447	5.025719568	5.01548E-07	intergenic	OC151121;LOC3890	dist=535961;dist=113010
3M	rs34895266	2	chr2	0.254174757	52	0.981	6.714996	1.337157087	5.021845077	5.11775E-07	intergenic	OC151121;LOC3890	dist=541788;dist=107183
3M	rs4239537	19	chr19	0.019417476	4	1	2.066074	0.41173362	5.017986311	5.22159E-07	intergenic	NOVA2;CCDC61	dist=3750;dist=18312
3M	rs150428353	22	chr22	0.024252427	5	0.7834	2.149293	0.429334633	5.006102254	5.55433E-07	intronic	DGCR2	.
3M	rs144327698	15	chr15	0.01557767	3	0.9166	1.784212	0.357897115	4.985266493	6.18765E-07	intergenic	RORA;VPS13C	dist=46045;dist=577043
3M	rs143096638	15	chr15	0.015490291	3	0.9134	1.774737	0.356244746	4.98179214	6.29981E-07	intergenic	RORA;VPS13C	dist=33553;dist=589535
3M	rs146268777	3	chr3	0.014699029	3	0.9443	1.721264	0.347407762	4.954593705	7.24816E-07	ncRNA_intronic	ADAMTS9-AS1	.
3M	rs35748618	2	chr2	0.222898058	46	0.9623	6.12404	1.238717369	4.943856031	7.65923E-07	intergenic	OC151121;LOC3890	dist=552678;dist=96293
3M	rs190652742	5	chr5	0.024257281	5	0.9994	2.247608	0.454880737	4.941093741	7.76855E-07	intergenic	RHGEF28;LINC0133	dist=25561;dist=338856
3M	rs6742395	2	chr2	0.258529127	53	0.9762	6.558142	1.329599407	4.932419546	8.12172E-07	intergenic	OC151121;LOC3890	dist=561121;dist=87850
3M	rs186620466	X	chrX	0.013970874	3	0.873	1.634757	0.331433352	4.932386049	8.12312E-07	intergenic	DACH2;KLHL4	dist=94083;dist=591027
3M	rs191906004	10	chr10	0.015543689	3	0.8436	1.645849	0.333793955	4.93073396	8.19212E-07	intronic	MLLT10	.
3M	rs188790792	10	chr10	0.014849515	3	0.8239	1.594035	0.323294895	4.930592272	8.19807E-07	intronic	MLLT10	.
3M	rs4277471	2	chr2	0.257281553	53	1	6.683699	1.358232147	4.920881476	8.61553E-07	intergenic	OC151121;LOC3890	dist=555178;dist=93793
3M	rs12116501	1	chr1	0.040257281	8	0.8071	2.387314	0.485212014	4.920145248	8.648E-07	intronic	LRP8	.
3M	rs72754890	1	chr1	0.019203883	4	0.8064	1.815512	0.369826911	4.909085989	9.15019E-07	intronic	RGS7	.
3M	rs149916697	7	chr7	0.018815534	4	0.9284	1.879423	0.384569904	4.887077696	1.02344E-06	intronic	STEAP2	.
3M	rs79847565	7	chr7	0.018800971	4	0.9455	1.894046	0.387759417	4.8845918	1.03643E-06	intronic	GTPBP10	.
3M	rs187685448	7	chr7	0.018776699	4	0.9679	1.910374	0.392033885	4.872982793	1.09926E-06	intronic	GTPBP10	.
3M	rs1882610	2	chr2	0.263592233	54	0.9774	6.506401	1.335621499	4.871440856	1.10787E-06	intergenic	OC151121;LOC3890	dist=557944;dist=91027

3M	rs143425398	7	chr7	0.018825242	4	0.9704	1.913164	0.392804365	4.870526408	1.11301E-06	intergenic	CLDN12;CDK14	dist=1847;dist=178561
3M	rs12621133	2	chr2	0.264009709	54	0.98	6.522687	1.33928955	4.87025933	1.11452E-06	intergenic	OC151121;LOC38903	dist=550944;dist=98027
3M	rs150148213	7	chr7	0.01892233	4	0.9738	1.921234	0.394605718	4.868742276	1.12311E-06	intronic	CDK14	.
3M	rs147985761	1	chr1	0.013373786	3	0.894	1.580946	0.324859599	4.866551587	1.13562E-06	intergenic	TNFRSF8;MIR7846	dist=11019;dist=11717
3M	rs4879986	9	chr9	0.023582524	5	0.9707	2.18417	0.448849347	4.866154904	1.1379E-06	intronic	RNF38	.
3M	rs2248297	9	chr9	0.97573301	5	0.9998	-2.242693	0.461738278	-4.85706436	1.19139E-06	intronic	RNF38	.
3M	rs146154846	4	chr4	0.028898058	6	0.712	2.023347	0.417548067	4.845782525	1.26114E-06	intergenic	FSTL5;MIR4454	dist=891178;dist=38362
3M	rs75685045	2	chr2	0.020179612	4	0.945	2.00561	0.414444146	4.839276486	1.30313E-06	intergenic	DPP10;DDX18	dist=746343;dist=1223586
3M	rs79319828	9	chr9	0.024014563	5	0.9887	2.210438	0.456810098	4.838856266	1.30588E-06	intronic	RNF38	.
3M	rs76774745	9	chr9	0.024029126	5	0.9881	2.210045	0.456790677	4.838201677	1.31019E-06	intronic	RNF38	.
3M	rs79985283	17	chr17	0.0145	3	0.9949	1.703939	0.352883611	4.828613304	1.37487E-06	intronic	MGC57346-CRHR1	.
3M	rs77364739	10	chr10	0.038834951	8	1	2.690625	0.557664123	4.824813526	1.40134E-06	intergenic	PCBD1;UNC5B	dist=141747;dist=182002
3M	rs79934683	1	chr1	0.038961165	8	0.9931	2.731689	0.567885471	4.81028073	1.50718E-06	intronic	CTH	.
3M	rs146812516	20	chr20	0.017223301	4	0.863	1.826409	0.380591181	4.798874146	1.5956E-06	intergenic	BMP2;LINC01428	dist=194893;dist=171296
3M	rs41307102	14	chr14	0.024456311	5	0.6367	1.768121	0.369052815	4.790970568	1.65976E-06	intronic	ZFHX2	.
3M	rs150470417	8	chr8	0.044912622	9	0.9604	2.78514	0.582245322	4.783447747	1.72314E-06	intergenic	C8orf4;ZMAT4	dist=36865;dist=338419
3M	rs116259399	8	chr8	0.04368932	9	1	2.810808	0.588520937	4.776053945	1.78768E-06	intergenic	C8orf4;ZMAT4	dist=14274;dist=361010
3M	rs115149137	5	chr5	0.014830097	3	0.9674	1.706395	0.358423256	4.760836832	1.92792E-06	intronic	ADAMTS16	.
3M	rs7800716	7	chr7	0.017533981	4	0.8306	1.680077	0.353443095	4.753459521	1.99965E-06	ncRNA_intronic	STEAP2-AS1	.
3M	rs113189296	4	chr4	0.037621359	8	0.9705	2.919015	0.615000485	4.746362423	2.07107E-06	intronic	APBB2	.
3M	rs72754888	1	chr1	0.020087379	4	0.8364	1.824933	0.38452179	4.74597992	2.07499E-06	intronic	RGS7	.
3M	rs145155685	5	chr5	0.014868932	3	0.9443	1.680708	0.35442199	4.742109459	2.11504E-06	intronic	ADAMTS16	.
3M	rs189164061	7	chr7	0.014519418	3	0.9963	1.695949	0.358675926	4.728360877	2.2634E-06	intronic	ABCA13	.
3M	rs76143420	11	chr11	0.014893204	3	0.9713	1.69951	0.360057984	4.720101852	2.35727E-06	intergenic	ACCSL;ACCS	dist=4448;dist=1754
3M	rs75197268	13	chr13	0.00926699	2	0.5394	1.077369	0.228342774	4.718211147	2.37927E-06	intronic	FGF14	.
3M	rs113484345	18	chr18	0.023024272	5	0.8907	1.975849	0.419524828	4.709731495	2.48043E-06	intergenic	KC6;PIK3C3	dist=293911;dist=140691
3M	rs187567621	4	chr4	0.014470874	3	0.948	1.644356	0.349394212	4.706306808	2.52245E-06	intronic	GALNTL6	.
3M	rs61856855	10	chr10	0.013485437	3	0.6384	1.297898	0.276292266	4.697556103	2.63293E-06	intergenic	PIK3AP1;MIR607	dist=78261;dist=29886
3M	rs147220861	13	chr13	0.027004854	6	0.826	2.031117	0.432525467	4.695948131	2.65373E-06	intergenic	LINC00378;MIR3169	dist=402574;dist=101424
3M	rs145442123	13	chr13	0.022786408	5	0.9305	2.002149	0.426532153	4.694017139	2.67892E-06	intronic	MYO16	.
3M	rs76854078	12	chr12	0.01723301	4	0.9045	1.74408	0.37159489	4.693499608	2.68571E-06	intronic	FAM19A2	.
3M	rs149030097	11	chr11	0.01565534	3	0.9315	1.691379	0.360755196	4.688439897	2.75296E-06	intronic	ACCSL	.
3M	rs145034465	4	chr4	0.02361165	5	0.7328	1.814612	0.387671579	4.680797751	2.85761E-06	intergenic	FSTL5;MIR4454	dist=915043;dist=14497
3M	rs75505956	10	chr10	0.036461165	8	0.9532	2.445967	0.523449162	4.672787474	2.97139E-06	intergenic	PCBD1;UNC5B	dist=158277;dist=165472
3M	rs62072692	17	chr17	0.023776699	5	0.8199	1.836346	0.394018804	4.660554743	3.15358E-06	intergenic	KIF2B;TOM1L1	dist=463573;dist=611906
3M	rs4355062	2	chr2	0.297684466	61	0.982	6.436745	1.381972449	4.657650781	3.19838E-06	intergenic	OC151121;LOC38903	dist=537092;dist=111879
3M	rs185879164	2	chr2	0.018174757	4	0.9295	1.804243	0.387623961	4.654621515	3.24576E-06	intronic	EXOC6B	.
3M	rs183818481	10	chr10	0.011912621	2	0.6742	1.227912	0.263818829	4.654374424	3.24966E-06	intergenic	NEBL-AS1;CASC10	dist=285177;dist=34392
3M	rs28631454	15	chr15	0.048796117	10	0.8906	2.632235	0.565845842	4.651858229	3.28957E-06	intergenic	TRIM69;C15orf43	dist=137980;dist=50893
3M	rs146684353	20	chr20	0.032533981	7	0.9186	2.316388	0.498848234	4.643473279	3.426E-06	intronic	EYA2	.
3M	rs55850402	6	chr6	0.019490291	4	0.9883	1.930704	0.415892439	4.642316741	3.44524E-06	intergenic	IMPG1;HTR1B	dist=689793;dist=698377
3M	rs191563430	6	chr6	0.019490291	4	0.9883	1.930447	0.415888081	4.641745998	3.45477E-06	intergenic	IMPG1;HTR1B	dist=677994;dist=710176
3M	rs115540332	2	chr2	0.018898058	4	0.9147	1.818658	0.391871559	4.640953721	3.46805E-06	intronic	EXOC6B	.
3M	rs42872	5	chr5	0.306907767	63	0.9807	6.43628	1.389496475	4.632095094	3.61984E-06	intronic	ARL15	.
3M	rs144178739	X	chrX	0.015194175	3	0.8145	1.517882	0.327834067	4.630030494	3.65612E-06	intergenic	RPS6KA3;CNKSR2	dist=650277;dist=457509
3M	rs13164825	5	chr5	0.155339806	32	1	5.112932	1.104845474	4.627734581	3.69687E-06	intronic	ADAMTS12	.
3M	rs79231845	4	chr4	0.053990291	11	0.9643	3.258068	0.70424346	4.626337545	3.72189E-06	intergenic	02723778;LOC10272	dist=569458;dist=216247
3M	rs77029592	6	chr6	0.019640777	4	0.9808	1.923001	0.415738055	4.625511258	3.73676E-06	intergenic	IMPG1;HTR1B	dist=647417;dist=740753
3M	rs181139900	10	chr10	0.011519417	2	0.7758	1.283846	0.277566907	4.625356308	3.73955E-06	intronic	DNAJC1	.
3M	rs10057508	5	chr5	0.164631068	34	0.9919	4.973633	1.076119596	4.621821771	3.80385E-06	intronic	ADAMTS12	.
3M	rs78549750	6	chr6	0.019699029	4	0.9783	1.920386	0.415757928	4.618999777	3.85594E-06	intergenic	IMPG1;HTR1B	dist=637633;dist=750537
3M	rs56232065	6	chr6	0.019820388	4	0.98	1.925532	0.417298507	4.614280674	3.94459E-06	intergenic	IMPG1;HTR1B	dist=632275;dist=755895
3M	rs13176485	5	chr5	0.159796116	33	0.9916	4.948363	1.073508457	4.609524006	4.03592E-06	intronic	ADAMTS12	.
3M	rs35388476	5	chr5	0.15973301	33	0.9921	4.946113	1.073435357	4.60774197	4.07065E-06	intronic	ADAMTS12	.

3M	rs144499025	21	chr21	0.007194175	1	0.5388	0.846201	0.183711704	4.606133495	4.10225E-06	intergenic	LINC00649;MRPS6	dist=38864;dist=70697
3M	rs147118669	11	chr11	0.012529126	3	0.7968	1.341789	0.291618921	4.601172279	4.2012E-06	intergenic	NELL1;ANO5	dist=177993;dist=439500
3M	rs184920540	10	chr10	0.011684466	2	0.7882	1.295186	0.281543412	4.600306895	4.21869E-06	intronic	DNAJC1	.
3M	rs67267661	5	chr5	0.160194175	33	1	4.958248	1.078142852	4.598878331	4.24772E-06	intronic	ADAMTS12	.
3M	rs16885796	4	chr4	0.053223301	11	0.9943	3.281609	0.713701131	4.598015718	4.26534E-06	intergenic	02723778;LOC1027	dist=552705;dist=233000
3M	rs11676376	2	chr2	0.024257282	5	0.9986	2.107857	0.458486846	4.597421782	4.27751E-06	intergenic	SPRED2;MIR4778	dist=641939;dist=283786
3M	rs45496395	10	chr10	0.013985437	3	0.8731	1.470045	0.319890854	4.595456497	4.31803E-06	intronic	DNAJC1	.
3M	rs115916744	4	chr4	0.053402913	11	0.9999	3.290833	0.717091233	4.589140975	4.45074E-06	intergenic	02723778;LOC1027	dist=566567;dist=219138
3M	rs116957173	4	chr4	0.053398058	11	1	3.290739	0.717096412	4.588976583	4.45424E-06	intergenic	02723778;LOC1027	dist=559588;dist=226117
3M	rs143503046	5	chr5	0.00734466	2	0.6866	0.983175	0.214354222	4.58668487	4.5034E-06	intergenic	LINC01019;IRX1	dist=25887;dist=34073
3M	rs116168704	4	chr4	0.053165049	11	0.9912	3.264992	0.71189775	4.586321223	4.51124E-06	intergenic	02723778;LOC1027	dist=564298;dist=221407
3M	rs115263712	1	chr1	0.014364078	3	0.8337	1.46895	0.320848564	4.578327141	4.68709E-06	intronic	IGSF21	.
3M	rs560030667	4	chr4	0.010296117	2	0.9445	1.359541	0.297050321	4.576803849	4.72134E-06	ncRNA_intronic	LINC01060	.
3M	rs544973300	4	chr4	0.010276699	2	0.9464	1.359542	0.297112351	4.575850811	4.74288E-06	ncRNA_intronic	LINC01060	.
3M	rs12153142	5	chr5	0.980427185	4	0.4992	-1.381863	0.302073383	-4.57459343	4.77146E-06	ncRNA_intronic	LOC102467216	.

Supplementary Table S15. GWAS Results Indianapolis-2 Replication Cohort
12 month quantitative trait (QT), risk locus ordered

Notes
Bold boxes in Gene.refGene column indicate individual risk loci; Shaded cells indicate risk loci that co-localize with risk loci identified in the Indianapolis-1 GWAS
25 risk loci total   one overlaps with 3 month: PCBD1;UNC5B   Thus 57+25-1 = 81 total risk loci   total risk loci overlapping Indianapolis-1; 13/81 = 16%
Standard Headers
rsID: reference SNP cluster ID, chr_38: chromosome number; pos_38, position of SNP on GRCh38 reference panel; Imputation_Rsq; estimate of the squared correlation between imputed and true genotypes; REF and ALT, reference allele and alternate allele;
n.obs: number of observations; caf: coding allele frequency; MAC: minor allele count; Score: p-values from Score test; Score.SE: standard error of the score statistic; Score.Stat: computed score statistic based on the derivative of the log-likelihood function;
Score.pval: probability value associated with the Score.Stat; EST: estimated effect size; EST.SE: standard error of the effect size; Func.refGene: SNP location with respect to nearest gene; Gene.refGene: closet gene(s) upstream and downstream;
GeneDetail.refGene: distance of SNP to nearest gene(s)
Added Headers
QT: quantitative trait
Abbreviations and Acronyms
3M: 3 month; 12M: 12 month; AOP:aqueous out flow pathway

QT	rsID	Chr_38	POS_38	freq	MAC	Rsq	Score	Score.SE	Score.Stat	Score.pval	Func.refGene	Gene.refGene	GeneDetail.refGene	AOP-expressed refGene (protein coding only)	Gene name if AOP-expressed
12M	rs138665118	chr2	49835636	0.0308641	6	0.892953	1.721021507	0.35697746	4.821092882	1.42774E-06	intergenic	FSHR;NRXN1	dist=681108;dist=82869	NRXN1	Neurexin-1
12M	rs12621729	chr2	74255421	0.0322087	7	0.942493	1.780987998	0.37313777	4.77300378	1.81498E-06	intronic	SLC4A5	.	SLC4A5	Solute Carrier Family 4 Member 5
12M	rs28798228	chr2	126155850	0.0331748	7	0.961063	1.808450116	0.37851254	4.777781303	1.7724E-06	intergenic	CNTNAP5;GYPC	dist=1240473;dist=500084	GYPC	Glycophorin C
12M	rs28796094	chr2	126197637	0.0324417	7	0.939443	1.7618909	0.37073145	4.752472211	2.00944E-06	intergenic	CNTNAP5;GYPC	dist=1282260;dist=458297		
12M	rs189440995	chr2	133887028	0.0147864	3	0.899627	1.137571373	0.24878258	4.572552272	4.81819E-06	intergenic	NCKAP5;MIR3679	dist=318568;dist=240097	NCKAP5	NCK Associated Protein 5
12M	rs185293133	chr3	75433381	0.0116262	2	0.492536	0.80016771	0.15901908	5.031897471	4.85649E-07	ncRNA_intronic	FAM86DP	.		
12M	rs73150883	chr3	146330831	0.0238155	5	0.848249	1.481987222	0.28774985	5.150262284	2.60122E-07	intergenic	PLSCR4;PLSCR2	dist=79652;dist=102457	PLSCR4;PLSCR2	Phospholipid scramblase 4; phospholipid scramblase 2
12M	rs6583185	chr3	196898971	0.9717913	6	0.853106	-1.52139796	0.33118931	-4.593741177	4.35369E-06	intronic	SENP5	.	SENP5	SUMO specific peptidase 5
12M	rs72678487	chr4	124461029	0.0194563	4	0.987952	1.343334873	0.29403913	4.568558189	4.91091E-06	intergenic	LINC01091;LOC101927087	dist=530666;dist=38913		
12M	rs76433992	chr5	116641213	0.029835	6	0.974211	1.764770451	0.36047037	4.895743415	9.79348E-07	intergenic	SEMA6A;LOC102467223	dist=66287;dist=102089	SEMA6A	Semaphorin 6A
12M	rs114241096	chr5	116644156	0.0290922	6	0.998458	1.782650366	0.3612333	4.934900476	8.01916E-07	intergenic	SEMA6A;LOC102467223	dist=69230;dist=99146		
12M	rs114587121	chr5	116644342	0.0290922	6	0.998458	1.782650366	0.3612333	4.934900476	8.01916E-07	intergenic	SEMA6A;LOC102467223	dist=69416;dist=98960		
12M	rs79516960	chr5	116645028	0.0290971	6	0.998286	1.782403345	0.36123109	4.934246796	8.04606E-07	intergenic	SEMA6A;LOC102467223	dist=70102;dist=98274		
12M	rs78085382	chr5	116645205	0.0290971	6	0.998286	1.782403345	0.36123109	4.934246796	8.04606E-07	intergenic	SEMA6A;LOC102467223	dist=70279;dist=98097		
12M	rs79794218	chr5	116645540	0.0290971	6	0.998286	1.782403345	0.36123109	4.934246796	8.04606E-07	intergenic	SEMA6A;LOC102467223	dist=70614;dist=97762		
12M	rs114108437	chr5	116646294	0.0290971	6	0.998286	1.782403345	0.36123109	4.934246796	8.04606E-07	intergenic	SEMA6A;LOC102467223	dist=71368;dist=97008		
12M	rs77857559	chr5	116652484	0.0291262	6	1	1.784300998	0.36172467	4.932759998	8.10758E-07	intergenic	SEMA6A;LOC102467223	dist=77558;dist=90818		
12M	rs115917626	chr5	116653147	0.0291262	6	1	1.784300998	0.36172467	4.932759998	8.10758E-07	intergenic	SEMA6A;LOC102467223	dist=78221;dist=90155		
12M	rs76006987	chr5	116656217	0.0290874	6	0.998629	1.782276135	0.36123386	4.933856736	8.06216E-07	intergenic	SEMA6A;LOC102467223	dist=81291;dist=87085		
12M	rs114500719	chr5	163069412	0.0283301	6	0.909911	1.557183414	0.34021336	4.577078964	4.71514E-06	intergenic	GABRG2;CCNG1	dist=913873;dist=368159	GABRG2	Gamma-Aminobutyric Acid Type A Receptor Subunit
12M	rs116189826	chr5	163202772	0.0245388	5	0.937899	1.5321512	0.32364261	4.734083693	2.20047E-06	intergenic	GABRG2;CCNG1	dist=1047233;dist=234799	CCNG1	Cyclin G1
12M	rs115331238	chr5	163219712	0.0244612	5	0.983743	1.583713858	0.33185801	4.772263512	1.82167E-06	intergenic	GABRG2;CCNG1	dist=1064173;dist=217859		
12M	rs116626718	chr5	163226090	0.0243883	5	0.995221	1.593867941	0.33347822	4.779526333	1.75709E-06	intergenic	GABRG2;CCNG1	dist=1070551;dist=211481		
12M	rs114008181	chr5	163234729	0.0252136	5	0.958521	1.5722456	0.33187599	4.73744913	2.16425E-06	intergenic	GABRG2;CCNG1	dist=1079190;dist=202842		
12M	rs1050276	chr6	31270291	0.024432	5	0.991508	1.532787887	0.3329803	4.603238922	4.15971E-06	exonic	HLA-C	.		
12M	rs41544614	chr6	31271024	0.0243495	5	0.994693	1.533218592	0.33286996	4.606058777	4.10372E-06	intronic	HLA-C	.		
12M	rs29029490	chr6	31271950	0.024335	5	0.991645	1.529466801	0.33227278	4.603045682	4.16357E-06	intronic	HLA-C	.		
12M	rs9366775	chr6	31272319	0.0242718	5	1	1.534745688	0.33329599	4.604752947	4.12956E-06	upstream	HLA-C	dist=183		
12M	rs9357121	chr6	31272702	0.0242718	5	1	1.534745688	0.33329599	4.604752947	4.12956E-06	upstream	HLA-C	dist=566		
12M	rs4298345	chr6	31273041	0.0242039	5	0.997144	1.529478709	0.33238499	4.601527555	4.19404E-06	upstream	HLA-C	dist=905		
12M	rs9391714	chr6	31277303	0.0242718	5	1	1.534745688	0.33329599	4.604752947	4.12956E-06	intergenic	HLA-C;HLA-B	dist=5167;dist=76569	HLA-C	Major Histocompatibility Complex, Class I, C
12M	rs56356836	chr6	31279490	0.0242379	5	0.998566	1.532381559	0.33283392	4.60404267	4.14368E-06	intergenic	HLA-C;HLA-B	dist=7354;dist=74382	HLA-B	Major Histocompatibility Complex, Class I, B
12M	rs9405016	chr6	31280221	0.0242379	5	0.998566	1.532381559	0.33283392	4.60404267	4.14368E-06	intergenic	HLA-C;HLA-B	dist=8085;dist=73651		
12M	rs12529015	chr6	31280485	0.0242379	5	0.998566	1.532381559	0.33283392	4.60404267	4.14368E-06	intergenic	HLA-C;HLA-B	dist=8349;dist=73387		
12M	rs9368669	chr6	31280716	0.0242379	5	0.998566	1.532381559	0.33283392	4.60404267	4.14368E-06	intergenic	HLA-C;HLA-B	dist=8580;dist=73156		
12M	rs9380234	chr6	31280791	0.0243058	5	0.995714	1.53280058	0.33276482	4.606257864	4.0998E-06	intergenic	HLA-C;HLA-B	dist=8655;dist=73081		
12M	rs17198734	chr6	31288249	0.0242379	5	0.998566	1.532381559	0.33283392	4.60404267	4.14368E-06	intergenic	HLA-C;HLA-B	dist=16113;dist=65623		
12M	rs17192386	chr6	31288281	0.0242379	5	0.998566	1.532381559	0.33283392	4.60404267	4.14368E-06	intergenic	HLA-C;HLA-B	dist=16145;dist=65591		
12M	rs148294287	chr6	126169178	0.0169175	3	0.861892	1.203746268	0.25927127	4.642806213	3.43709E-06	intergenic	MIR5695;CENPW	dist=46562;dist=170611	CENPW	Centromere protein W
12M	rs142006494	chr6	158568942	0.0155825	3	0.909469	1.102833648	0.23946303	4.60544434	4.11586E-06	intronic	TMEM181	.	TMEM181	Transmembrane protein 181
12M	rs17206867	chr7	9449981	0.0197816	4	0.886061	1.317750936	0.27773373	4.744655705	2.08861E-06	intergenic	NXPH1;PER4	dist=697018;dist=184289		
12M	rs539324287	chr8	23605195	0.0169417	3	0.907302	1.341800652	0.26460113	5.071031363	3.95666E-07	intergenic	SLC25A37;NKX3-1	dist=32645;dist=73498	SLC25A37	Solute Carrier Family 25 Member 37
12M	rs189490695	chr8	23685209	0.0163835	3	0.874937	1.2982161	0.2556875	5.077354608	3.82726E-07	intergenic	NKX3-1;NKX2-6	dist=2272;dist=17242	NKX3-1	NK3 Homeobox 1
12M	rs74591804	chr8	132987195	0.0145583	3	0.999662	1.200642195	0.25459157	4.715954269	2.4058E-06	intronic	TG	.	TG	Thyroglobulin
12M	rs77364739	chr10	71030533	0.038835	8	1	1.881493073	0.41157336	4.571464641	4.84327E-06	intergenic	PCBD1;UNC5B	dist=141747;dist=182002	PCBD1;UNC5B	Pterin-4 Alpha-Carbinolamine Dehydratase 1;Unc-5 Netrin Receptor B
12M	rs111621289	chr12	10893543	0.0304223	6	0.810976	1.581110295	0.33666074	4.696449854	2.64722E-06	ncRNA_intronic	PRH1-PRR4	.		
12M	rs74833295	chr13	30194287	0.029165	6	0.9983	1.909228275	0.36002172	5.303091934	1.13858E-07	intergenic	LINC00365;KATNAL1	dist=85412;dist=8343	KATNAL1	Katanin Catalytic Subunit A1 Like 1
12M	rs148654242	chr13	52129747	0.0360631	7	0.890541	1.896523445	0.38630706	4.90936778	9.13705E-07	ncRNA_exonic	LOC101929657	.		
12M	rs72705708	chr15	27388838	0.0684709	14	0.990527	2.432058671	0.51344077	4.736785214	2.17135E-06	intronic	GABRG3	.	GABRG3	Gamma-Aminobutyric Acid Type A Receptor Subunit Gamma3
12M	rs72705709	chr15	27389069	0.0683495	14	0.992399	2.429618929	0.5133886	4.732514355	2.21756E-06	intronic	GABRG3	.		
12M	rs72705710	chr15	27389259	0.0683204	14	0.992701	2.428373305	0.51325152	4.731351398	2.2303E-06	intronic	GABRG3	.		
12M	rs72705711	chr15	27390210	0.0679709	14	0.997858	2.443670474	0.51382691	4.75582426	1.97638E-06	intronic	GABRG3	.		
12M	rs72705712	chr15	27391241	0.0679612	14	0.998164	2.444243265	0.51389229	4.756333777	1.9714E-06	intronic	GABRG3	.		
12M	rs72705714	chr15	27391807	0.0679612	14	1	2.448622014	0.51441141	4.760046044	1.93549E-06	intronic	GABRG3	.		
12M	rs72705715	chr15	27392048	0.0679612	14	0.998164	2.444243265	0.51389229	4.756333777	1.9714E-06	intronic	GABRG3	.		
12M	rs75968509	chr15	31090915	0.0193592	4	0.979554	1.431828899	0.30212194	4.739241691	2.1452E-06	intronic	TRPM1	.	TRPM1	Transient Receptor Potential Cation Channel Subfamily M Member 1
12M	rs113199364	chr17	77161818	0.033301	7	0.893794	1.815437814	0.35840665	5.065301631	4.07754E-07	intronic	SEC14L1	.	SEC14L1	SEC14 Like Lipid Binding 1
12M	rs74649788	chr18	6045906	0.0498932	10	0.923243	2.439599988	0.44900137	5.43339095	5.52931E-08	intronic	L3MBTL4	.	L3MBTL4	L3MBTL Histone Methyl-Lysine Binding Protein 4

12M	rs79381542	chr18	6049027	0.0443592	9	0.959092	2.087445553	0.41784921	4.995691057	5.86254E-07	intronic	L3MBTL4	.		
12M	rs58503971	chr18	6049552	0.0443495	9	0.959099	2.087223432	0.41779888	4.995761149	5.86042E-07	intronic	L3MBTL4	.		

Supplementary Table S16. GWAS Results Indianapolis-2 Replication Cohort
3 month quantitative trait (QT), risk locus ordered

Notes
Bold boxes in Gene.refGene column indicate individual risk loci; Shaded cells indicate risk loci that co-localize with risk loci identified in the Indianapolis-1 GWAS
57 risk loci total one overlaps with 12 month: PCBD1;UNC5B Thus 57+25-1 = 81 total risk loci total risk loci overlapping Indianapolis-1; 13/81 = 16%

Standard Headers
rsID: reference SNP cluster ID, chr_38: chromosome number; pos_38, position of SNP on GRCh38 reference panel; Imputation_Rsq; estimate of the squared correlation between imputed and true genotypes; REF and ALT, reference allele and alternate allele; n.obs: number of observations; caf: coding allele frequency; MAC: minor allele count; Score: p-values from Score test; Score.SE: standard error of the score statistic; Score.Stat: computed score statistic based on the derivative of the log-likelihood function; Score.pval: probability value associated with the Score.Stat; EST: estimated effect size; EST.SE: standard error of the effect size; Func.refGene: SNP location with respect to nearest gene; Gene.refGene: closet gene(s) upstream and downstream; GeneDetail.refGene: distance of SNP to nearest gene(s)

Added Headers
QT: quantitative trait

Abbreviations and Acronyms
3M: 3 month; 12M: 12 month; AOP:aqueous out flow pathway

QT	rsID	Chr_38	POS_38	freq	MAC	Rsq	Score	Score.SE	Score.Stat	Score.pval	Func.refGene	Gene.refGene	GeneDetail.refGene	AOP-expressed refGene (protein coding only)
3M	rs147985761	chr1	12155226	0.013373786	3	0.894	1.580946	0.324859599	4.866551587	1.13562E-06	intergenic	TNFRSF8;MIR7846	dist=11019;dist=11717	TNFRSF8
3M	rs115263712	chr1	18287999	0.014364078	3	0.8337	1.46895	0.320848564	4.578327141	4.68709E-06	intronic	IGSF21	.	IGSF21
3M	rs12116501	chr1	53255051	0.040257281	8	0.8071	2.387314	0.485212014	4.920145248	8.648E-07	intronic	LRP8	.	LRP8
3M	rs79934683	chr1	70420646	0.038961165	8	0.9931	2.731689	0.567885471	4.81028073	1.50718E-06	intronic	CTH	.	CTH
3M	rs72754888	chr1	2.41E+08	0.020087379	4	0.8364	1.824933	0.38452179	4.74597992	2.07499E-06	intronic	RGS7	.	RGS7
3M	rs72754890	chr1	2.41E+08	0.019203883	4	0.8064	1.815512	0.369826911	4.909085989	9.15019E-07	intronic	RGS7	.	
3M	rs11676376	chr2	66074461	0.024257282	5	0.9986	2.107857	0.458486846	4.597421782	4.27751E-06	intergenic	SPRED2;MIR4778	dist=641939;dist=283786	SPRED2
3M	rs185879164	chr2	72402105	0.018174757	4	0.9295	1.804243	0.387623961	4.654621515	3.24576E-06	intronic	EXOC6B	.	EXOC6B
3M	rs115540332	chr2	72625653	0.018898058	4	0.9147	1.818658	0.391871559	4.640953721	3.46805E-06	intronic	EXOC6B	.	
3M	rs75685045	chr2	1.17E+08	0.020179612	4	0.945	2.00561	0.414444146	4.839276486	1.30313E-06	intergenic	DPP10;DDX18	dist=746343;dist=1223586	DPP10;DDX18
3M	rs4337430	chr2	1.3E+08	0.248378641	51	0.973	6.84431	1.325444312	5.163785781	2.42005E-07	intergenic	LOC151121;LOC389033	dist=532066;dist=116905	
3M	rs34017523	chr2	1.3E+08	0.253538835	52	0.9789	6.708705	1.334874447	5.025719568	5.01548E-07	intergenic	LOC151121;LOC389033	dist=533874;dist=115097	
3M	rs6720381	chr2	1.3E+08	0.253538835	52	0.9789	6.708705	1.334874447	5.025719568	5.01548E-07	intergenic	LOC151121;LOC389033	dist=535961;dist=113010	
3M	rs4355062	chr2	1.3E+08	0.297684466	61	0.982	6.436745	1.381972449	4.657650781	3.19838E-06	intergenic	LOC151121;LOC389033	dist=537092;dist=111879	
3M	rs34895266	chr2	1.3E+08	0.254174757	52	0.981	6.714996	1.337157087	5.021845077	5.11775E-07	intergenic	LOC151121;LOC389033	dist=541788;dist=107183	
3M	rs12621133	chr2	1.3E+08	0.264009709	54	0.98	6.522687	1.33928955	4.87025933	1.11452E-06	intergenic	LOC151121;LOC389033	dist=550944;dist=98027	
3M	rs35748618	chr2	1.3E+08	0.222898058	46	0.9623	6.12404	1.238717369	4.943856031	7.65923E-07	intergenic	LOC151121;LOC389033	dist=552678;dist=96293	
3M	rs4277471	chr2	1.3E+08	0.257281553	53	1	6.683699	1.358232147	4.920881476	8.61553E-07	intergenic	LOC151121;LOC389033	dist=555178;dist=93793	
3M	rs1882610	chr2	1.3E+08	0.263592233	54	0.9774	6.506401	1.335621499	4.871440856	1.10787E-06	intergenic	LOC151121;LOC389033	dist=557944;dist=91027	
3M	rs6742395	chr2	1.3E+08	0.258529127	53	0.9762	6.558142	1.329599407	4.932419546	8.12172E-07	intergenic	LOC151121;LOC389033	dist=561121;dist=87850	
3M	rs145531574	chr2	1.5E+08	0.013723301	3	0.8698	1.731905	0.335798226	5.157575737	2.50168E-07	intergenic	RND3;LOC101929260	dist=3372;dist=61465	RND3
3M	rs146268777	chr3	64574169	0.014699029	3	0.9443	1.721264	0.347407762	4.954593705	7.24816E-07	ncRNA_intronic	ADAMTS9-AS1	.	ADAMTS9
3M	rs16885796	chr4	31764379	0.053223301	11	0.9943	3.281609	0.713701131	4.598015718	4.26534E-06	intergenic	LOC102723778;LOC102723828	dist=552705;dist=233000	
3M	rs116957173	chr4	31771262	0.053398058	11	1	3.290739	0.717096412	4.588976583	4.45424E-06	intergenic	LOC102723778;LOC102723828	dist=559588;dist=226117	
3M	rs116168704	chr4	31775972	0.053165049	11	0.9912	3.264992	0.71189775	4.586321223	4.51124E-06	intergenic	LOC102723778;LOC102723828	dist=564298;dist=221407	
3M	rs115916744	chr4	31778241	0.053402913	11	0.9999	3.290833	0.717091233	4.589140975	4.45074E-06	intergenic	LOC102723778;LOC102723828	dist=566567;dist=219138	
3M	rs79231845	chr4	31781132	0.053990291	11	0.9643	3.258068	0.70424346	4.626337545	3.72189E-06	intergenic	LOC102723778;LOC102723828	dist=569458;dist=216247	
3M	rs113189296	chr4	40946147	0.037621359	8	0.9705	2.919015	0.615000485	4.746362423	2.07107E-06	intronic	APBB2	.	APBB2
3M	rs146154846	chr4	1.63E+08	0.028898058	6	0.712	2.023347	0.417548067	4.845782525	1.26114E-06	intergenic	FSTL5;MIR4454	dist=891178;dist=38362	FSTL5
3M	rs145034465	chr4	1.63E+08	0.02361165	5	0.7328	1.814612	0.387671579	4.680797751	2.85761E-06	intergenic	FSTL5;MIR4454	dist=915043;dist=14497	
3M	rs187567621	chr4	1.72E+08	0.014470874	3	0.948	1.644356	0.349394212	4.706306808	2.52245E-06	intronic	GALNTL6	.	GALNTL6
3M	rs544973300	chr4	1.88E+08	0.010276699	2	0.9464	1.359542	0.297112351	4.575850811	4.74288E-06	ncRNA_intronic	LINC01060	.	
3M	rs560030667	chr4	1.88E+08	0.010296117	2	0.9445	1.359541	0.297050321	4.576803849	4.72134E-06	ncRNA_intronic	LINC01060	.	
3M	rs143503046	chr5	3561981	0.00734466	2	0.6866	0.983175	0.214354222	4.58668487	4.5034E-06	intergenic	LINC01019;IRX1	dist=25887;dist=34073	IRX1
3M	rs115149137	chr5	5233611	0.014830097	3	0.9674	1.706395	0.358423256	4.760836832	1.92792E-06	intronic	ADAMTS16	.	
3M	rs145155685	chr5	5241090	0.014868932	3	0.9443	1.680708	0.35442199	4.742109459	2.11504E-06	intronic	ADAMTS16	.	
3M	rs13164825	chr5	33635930	0.155339806	32	1	5.112932	1.104845474	4.627734581	3.69687E-06	intronic	ADAMTS12	.	ADAMTS12
3M	rs35388476	chr5	33644018	0.15973301	33	0.9921	4.946113	1.073435357	4.60774197	4.07065E-06	intronic	ADAMTS12	.	
3M	rs67267661	chr5	33644509	0.160194175	33	1	4.958248	1.078142852	4.598878331	4.24772E-06	intronic	ADAMTS12	.	
3M	rs10057508	chr5	33646351	0.164631068	34	0.9919	4.973633	1.076119596	4.621821771	3.80385E-06	intronic	ADAMTS12	.	
3M	rs13176485	chr5	33647529	0.159796116	33	0.9916	4.948363	1.073508457	4.609524006	4.03592E-06	intronic	ADAMTS12	.	
3M	rs42872	chr5	54094210	0.306907767	63	0.9807	6.43628	1.389496475	4.632095094	3.61984E-06	intronic	ARL15	.	ARL15
3M	rs190652742	chr5	73967554	0.024257281	5	0.9994	2.247608	0.454880737	4.941093741	7.76855E-07	intergenic	ARHGEF28;LINC01335	dist=25561;dist=338856	ARHGEF28

3M	rs12153142	chr5	1.13E+08	0.980427185	4	0.4992	-1.381863	0.302073383	-4.57459343	4.77146E-06	ncRNA_intronic	LOC102467216	.	
3M	rs56232065	chr6	76704953	0.019820388	4	0.98	1.925532	0.417298507	4.614280674	3.94459E-06	intergenic	IMPG1;HTR1B	dist=632275;dist=755895	
3M	rs78549750	chr6	76710311	0.019699029	4	0.9783	1.920386	0.415757928	4.618999777	3.85594E-06	intergenic	IMPG1;HTR1B	dist=637633;dist=750537	
3M	rs77029592	chr6	76720095	0.019640777	4	0.9808	1.923001	0.415738055	4.625511258	3.73676E-06	intergenic	IMPG1;HTR1B	dist=647417;dist=740753	
3M	rs191563430	chr6	76750672	0.019490291	4	0.9883	1.930447	0.415888081	4.641745998	3.45477E-06	intergenic	IMPG1;HTR1B	dist=677994;dist=710176	
3M	rs55850402	chr6	76762471	0.019490291	4	0.9883	1.930704	0.415892439	4.642316741	3.44524E-06	intergenic	IMPG1;HTR1B	dist=689793;dist=698377	
3M	rs189164061	chr7	48356469	0.014519418	3	0.9963	1.695949	0.358675926	4.728360877	2.2634E-06	intronic	ABCA13	.	ABCA13
3M	rs7800716	chr7	90172547	0.017533981	4	0.8306	1.680077	0.353443095	4.753459521	1.99965E-06	ncRNA_intronic	STEAP2-AS1	.	STEAP2
3M	rs149916697	chr7	90224643	0.018815534	4	0.9284	1.879423	0.384569904	4.887077696	1.02344E-06	intronic	STEAP2	.	
3M	rs79847565	chr7	90359162	0.018800971	4	0.9455	1.894046	0.387759417	4.8845918	1.03643E-06	intronic	GTPBP10	.	GTPBP10
3M	rs187685448	chr7	90384215	0.018776699	4	0.9679	1.910374	0.392033885	4.872982793	1.09926E-06	intronic	GTPBP10	.	
3M	rs143425398	chr7	90417801	0.018825242	4	0.9704	1.913164	0.392804365	4.870526408	1.11301E-06	intergenic	CLDN12;CDK14	dist=1847;dist=178561	CDK14
3M	rs150148213	chr7	90623747	0.01892233	4	0.9738	1.921234	0.394605718	4.868742276	1.12311E-06	intronic	CDK14	.	
3M	rs145892996	chr8	40087264	0.030776699	6	0.8841	2.576606	0.50654249	5.086652731	3.64438E-07	intergenic	IDO2;C8orf4	dist=70873;dist=66204	C8orf4
3M	rs138697513	chr8	40087398	0.030781554	6	0.8843	2.577315	0.506628696	5.087187008	3.63413E-07	intergenic	IDO2;C8orf4	dist=71007;dist=66070	
3M	rs116259399	chr8	40169582	0.04368932	9	1	2.810808	0.588520937	4.776053945	1.78768E-06	intergenic	C8orf4;ZMAT4	dist=14274;dist=361010	ZMAT4
3M	rs150470417	chr8	40192173	0.044912622	9	0.9604	2.78514	0.582245322	4.783447747	1.72314E-06	intergenic	C8orf4;ZMAT4	dist=36865;dist=338419	
3M	rs4879986	chr9	36357609	0.023582524	5	0.9707	2.18417	0.448849347	4.866154904	1.1379E-06	intronic	RNF38	.	RNF38
3M	rs79319828	chr9	36360848	0.024014563	5	0.9887	2.210438	0.456810098	4.838856266	1.30588E-06	intronic	RNF38	.	
3M	rs2248297	chr9	36381905	0.97573301	5	0.9998	-2.242693	0.461738278	-4.85706436	1.19139E-06	intronic	RNF38	.	
3M	rs76774745	chr9	36386868	0.024029126	5	0.9881	2.210045	0.456790677	4.838201677	1.31019E-06	intronic	RNF38	.	
3M	rs183818481	chr10	21460100	0.011912621	2	0.6742	1.227912	0.263818829	4.654374424	3.24966E-06	intergenic	NEBL-AS1;CASC10	dist=285177;dist=34392	NEBL
3M	rs191906004	chr10	21594510	0.015543689	3	0.8436	1.645849	0.333793955	4.93073396	8.19212E-07	intronic	MLLT10	.	MLLT10
3M	rs188790792	chr10	21681453	0.014849515	3	0.8239	1.594035	0.323294895	4.930592272	8.19807E-07	intronic	MLLT10	.	
3M	rs184920540	chr10	21779539	0.011684466	2	0.7882	1.295186	0.281543412	4.600306895	4.21869E-06	intronic	DNAJC1	.	DNAJC1
3M	rs181139900	chr10	21794688	0.011519417	2	0.7758	1.283846	0.277566907	4.625356308	3.73955E-06	intronic	DNAJC1	.	
3M	rs45496395	chr10	21927128	0.013985437	3	0.8731	1.470045	0.319890854	4.595456497	4.31803E-06	intronic	DNAJC1	.	
3M	rs77364739	chr10	71030533	0.038834951	8	1	2.690625	0.557664123	4.824813526	1.40134E-06	intergenic	PCBD1;UNC5B	dist=141747;dist=182002	PCBD1;UNC5B
3M	rs75505956	chr10	71047063	0.036461165	8	0.9532	2.445967	0.523449162	4.672787474	2.97139E-06	intergenic	PCBD1;UNC5B	dist=158277;dist=165472	
3M	rs61856855	chr10	96798783	0.013485437	3	0.6384	1.297898	0.276292266	4.697556103	2.63293E-06	intergenic	PIK3AP1;MIR607	dist=78261;dist=29886	PIK3AP1
3M	rs150977401	chr10	1.14E+08	0.01631068	3	0.8708	1.860675	0.358119043	5.195689556	2.03962E-07	intronic	CASP7	.	CASP7
3M	rs147118669	chr11	21753676	0.012529126	3	0.7968	1.341789	0.291618921	4.601172279	4.2012E-06	intergenic	NELL1;ANO5	dist=177993;dist=439500	NELL1;ANO5
3M	rs149030097	chr11	44052203	0.01565534	3	0.9315	1.691379	0.360755196	4.688439897	2.75296E-06	intronic	ACCSL	.	
3M	rs76143420	chr11	44064425	0.014893204	3	0.9713	1.69951	0.360057984	4.720101852	2.35727E-06	intergenic	ACCSL;ACCS	dist=4448;dist=1754	ACCS
3M	rs2014160	chr12	788899	0.01511165	3	0.8537	1.729592	0.339602326	5.092992566	3.52456E-07	intronic	WNK1	.	WNK1
3M	rs76854078	chr12	61751217	0.01723301	4	0.9045	1.74408	0.37159489	4.693499608	2.68571E-06	intronic	FAM19A2	.	FAM19A2
3M	rs61754236	chr12	78137211	0.028985437	6	0.9951	2.517384	0.494187455	5.093985122	3.50614E-07	exonic	NAV3	.	NAV3
3M	rs140686116	chr12	78151620	0.02904369	6	0.9971	2.519911	0.495194249	5.088732794	3.60464E-07	intronic	NAV3	.	
3M	rs147220861	chr13	61098374	0.027004854	6	0.826	2.031117	0.432525467	4.695948131	2.65373E-06	intergenic	LINC00378;MIR3169	dist=402574;dist=101424	
3M	rs9592740	chr13	70654334	0.066820388	14	0.9795	3.693411	0.700908722	5.269460143	1.36826E-07	intergenic	ATXN8OS;LINC00348	dist=514581;dist=360807	
3M	rs9599709	chr13	70668296	0.065	13	0.9585	3.560256	0.685315257	5.195063146	2.0465E-07	intergenic	ATXN8OS;LINC00348	dist=528543;dist=346845	
3M	rs75197268	chr13	1.02E+08	0.00926699	2	0.5394	1.077369	0.228342774	4.718211147	2.37927E-06	intronic	FGF14	.	FGF14
3M	rs145442123	chr13	1.09E+08	0.022786408	5	0.9305	2.002149	0.426532153	4.694017139	2.67892E-06	intronic	MYO16	.	MYO16
3M	rs41307102	chr14	23532453	0.024456311	5	0.6367	1.768121	0.369052815	4.790970568	1.65976E-06	intronic	ZFHX2	.	ZFHX2
3M	rs28631454	chr15	44905809	0.048796117	10	0.8906	2.632235	0.565845842	4.651858229	3.28957E-06	intergenic	TRIM69;C15orf43	dist=137980;dist=50893	TRIM69
3M	rs143862437	chr15	61239496	0.014752427	3	0.9521	1.816785	0.35534236	5.112774488	3.17461E-07	intergenic	RORA;VPS13C	dist=10193;dist=612895	RORA;VPS13C
3M	rs182462684	chr15	61242626	0.014762136	3	0.9515	1.816595	0.35534099	5.112258325	3.1833E-07	intergenic	RORA;VPS13C	dist=13323;dist=609765	
3M	rs143096638	chr15	61262856	0.015490291	3	0.9134	1.774737	0.356244746	4.98179214	6.29981E-07	intergenic	RORA;VPS13C	dist=33553;dist=589535	
3M	rs144327698	chr15	61275348	0.01557767	3	0.9166	1.784212	0.357897115	4.985266493	6.18765E-07	intergenic	RORA;VPS13C	dist=46045;dist=577043	
3M	rs79985283	chr17	45703113	0.0145	3	0.9949	1.703939	0.352883611	4.828613304	1.37487E-06	intronic	MGC57346-CRHR1	.	
3M	rs62072692	chr17	54288785	0.023776699	5	0.8199	1.836346	0.394018804	4.660554743	3.15358E-06	intergenic	KIF2B;TOM1L1	dist=463573;dist=611906	TOM1L1
3M	rs113484345	chr18	41814507	0.023024272	5	0.8907	1.975849	0.419524828	4.709731495	2.48043E-06	intergenic	KC6;PIK3C3	dist=293911;dist=140691	PIK3C3
3M	rs4239537	chr19	45977149	0.019417476	4	1	2.066074	0.41173362	5.017986311	5.22159E-07	intergenic	NOVA2;CCDC61	dist=3750;dist=18312	NOVA2;CCDC61
3M	rs146812516	chr20	6975171	0.017223301	4	0.863	1.826409	0.380591181	4.798874146	1.5956E-06	intergenic	BMP2;LINC01428	dist=194893;dist=171296	BMP2
3M	rs146684353	chr20	47091670	0.032533981	7	0.9186	2.316388	0.498848234	4.643473279	3.426E-06	intronic	EYA2	.	EYA2
3M	rs144499025	chr21	34002825	0.007194175	1	0.5388	0.846201	0.183711704	4.606133495	4.10225E-06	intergenic	LINC00649;MRPS6	dist=38864;dist=70697	MRPS6
3M	rs150428353	chr22	19092873	0.024252427	5	0.7834	2.149293	0.429334633	5.006102254	5.55433E-07	intronic	DGCR2	.	DGCR2
3M	rs144178739	chrX	20916909	0.015194175	3	0.8145	1.517882	0.327834067	4.630030494	3.65612E-06	intergenic	RPS6KA3;CNKSR2	dist=650277;dist=457509	CNKSR2
3M	rs186620466	chrX	86926685	0.013970874	3	0.873	1.634757	0.331433352	4.932386049	8.12312E-07	intergenic	DACH2;KLHL4	dist=94083;dist=591027	DACH2;KLHL4

Supplemental Table S17. Independent Replication
Indianpolis-2 cohort, 12M quantitative trait (QT), comparing to discovery 12M QT

Notes
Threshold for replicative significance: 5.00E-02
Headers
rsID: reference SNP cluster ID, chr: chromosome number; pos_37, position of SNP on GRCh37 reference panel; REF and ALT, reference allele and alternate allele;
n.obs: number of observations; caf: coding allele frequency; MAC: minor allele count; EST: estimated effect size; EST.SE: standard error of the effect size;
Score.pval: probability value for the discovery cohort; pval.indianapolis-2: probability value for the replication cohort

rsID	chr	pos_37	REF	ALT	n.obs Indy-1	caf Indy-1	MAC Indy-1	Est Indy-1	Est.SE Indy-1	Score.pval Indy-1	pval.Indy-2	Effect direction Indy-1	Effect direction Indy-2	Direction concordant
rs17875371	6	30460232	C	T	439	0.0182	16	6.01	1.74236555	3.09E-06	7.41E-02	+	+	Yes
rs112856203	12	72858864	T	C	439	0.0251	21	4.771	1.345267141	4.46E-06	1.08E-01	+	+	Yes
rs112796175	12	72790349	C	T	439	0.0251	21	4.767	2.110465052	3.11E-06	1.09E-01	+	+	Yes
rs77158902	17	32692036	C	T	439	0.0228	20	4.939	2.353806795	2.47E-06	1.59E-01	+	-	No
rs147944608	10	110423709	C	T	439	0.0125	11	6.938	2.322662367	7.94E-07	2.12E-01	+	-	No
rs72825069	17	32600928	C	T	439	0.0216	20	5.46	3.17190993	7.80E-07	2.17E-01	+	-	No
rs75626507	1	241415261	G	A	439	0.0034	4	11.843	3.134807227	4.55E-06	2.64E-01	+	-	No
rs1442156642	9	37664624	G	A	439	0.1526	134	2.109	2.32507224	2.31E-06	3.00E-01	+	-	No
rs2057644	9	37665980	G	A	439	0.1526	134	2.109	3.0663191	2.32E-06	3.00E-01	+	-	No
rs2057643	9	37666010	T	C	439	0.1526	134	2.109	1.248123667	2.32E-06	3.00E-01	+	-	No
rs10814594	9	37666667	T	C	439	0.1526	134	2.109	2.325930644	2.32E-06	3.02E-01	+	-	No
rs1125576	9	37667819	T	C	439	0.1526	134	2.108	2.694270059	2.33E-06	3.06E-01	+	-	No
rs10973475	9	37666361	C	T	439	0.0513	45	3.158	1.340871755	1.22E-06	3.18E-01	+	-	No
rs10973466	9	37661110	G	A	439	0.0513	45	3.156	2.556486955	1.22E-06	3.19E-01	+	-	No
rs79914278	9	37658196	C	T	439	0.0513	45	3.143	2.700180652	1.28E-06	3.19E-01	+	-	No
rs60006744	13	37672691	G	T	439	0.0513	45	3.164	2.899058719	1.21E-06	3.30E-01	+	-	No
rs2148140	9	37673839	C	T	439	0.0513	45	3.164	1.342649607	1.21E-06	3.55E-01	+	-	No
rs113399724	9	37674357	C	T	439	0.0513	45	3.164	3.276232883	1.21E-06	3.55E-01	+	-	No
rs55939894	3	7963288	A	G	439	0.1879	165	-1.81	3.149372695	2.51E-06	3.75E-01	-	+	No
rs11920261	3	7977211	G	T	439	0.1856	162	-1.791	3.119471569	3.27E-06	3.76E-01	-	+	No
rs2171551	3	7978078	C	A	439	0.1856	162	-1.79	2.630787815	3.28E-06	3.76E-01	-	+	No
rs146600651	1	152267708	C	T	439	0.0148	14	6.634	2.603345406	8.16E-07	4.19E-01	+	-	No
rs140561530	2	206620431	G	T	439	0.0023	3	16.878	2.62899673	1.05E-06	4.23E-01	+	-	No
rs142497891	13	74036983	G	A	439	0.0046	4	12.128	2.32524716	1.84E-07	4.75E-01	+	+	Yes
rs74517425	2	148193134	A	C	439	0.0296	25	4.131	3.254954928	3.17E-06	5.22E-01	+	-	No
rs72878847	11	24620738	A	G	439	0.0239	20	5.034	1.297950235	2.71E-06	5.23E-01	+	+	Yes
rs74492945	2	148184439	G	A	439	0.0296	25	4.094	1.105173881	3.30E-06	5.23E-01	+	-	No
rs75380640	2	148185797	A	G	439	0.0296	25	4.094	1.405398986	3.30E-06	5.23E-01	+	-	No
rs139548692	11	82604262	C	T	439	0.0046	6	11.494	1.98535452	3.73E-06	5.30E-01	+	+	Yes
rs5994128	22	17490932	A	G	439	0.7745	220	-2.143	2.804683764	1.02E-06	5.55E-01	-	+	No
rs75343152	2	148204747	T	C	439	0.0296	25	4.095	3.125578319	2.72E-06	5.68E-01	+	-	No
rs2229593	3	42906216	C	T	439	0.0273	24	4.57	2.704588306	2.39E-06	5.78E-01	+	+	Yes
rs141411318	11	24605549	C	T	439	0.0228	20	5.164	3.347221159	1.70E-06	7.19E-01	+	+	Yes
rs144967461	4	153768949	T	G	439	0.0182	16	6.499	1.810772179	1.92E-07	7.22E-01	+	-	No
rs72874841	11	24571002	C	T	439	0.0228	20	5.137	2.830692717	1.79E-06	7.32E-01	+	+	Yes
rs6724770	2	148227422	A	G	439	0.0273	24	4.339	1.20614552	2.85E-06	7.38E-01	+	-	No

rs76487561	2	148214978	C	T	439	0.0285	25	4.23	2.700290535	1.39E-06	7.39E-01	+	-	No
rs75330406	2	148238681	G	A	439	0.0273	24	4.348	0.351925413	2.72E-06	7.39E-01	+	-	No
rs78849093	4	148235591	C	A	439	0.0273	24	4.349	2.3435031	2.74E-06	7.39E-01	+	-	No
rs75304512	2	148218006	A	G	439	0.0273	24	4.335	2.164842011	2.87E-06	7.39E-01	+	-	No
rs77592434	2	148226645	A	G	439	0.0273	24	4.336	1.466193516	2.87E-06	7.39E-01	+	-	No
rs77928469	2	148224118	C	T	439	0.0273	24	4.336	2.811388633	2.87E-06	7.39E-01	+	-	No
rs74380227	2	148220282	C	A	439	0.0273	24	4.333	2.705032115	2.89E-06	7.39E-01	+	-	No
rs80339745	2	148227678	G	A	439	0.0273	24	4.338	3.13078059	2.85E-06	7.40E-01	+	-	No
rs10210722	2	148222783	T	C	439	0.0273	24	4.335	1.449522092	2.87E-06	7.40E-01	+	-	No
rs1258074269	2	148223792	A	C	439	0.0273	24	4.335	2.715800268	2.87E-06	7.40E-01	+	-	No
rs10175536	2	148217618	A	G	439	0.0273	24	4.303	1.748677029	3.07E-06	7.40E-01	+	-	No
rs62582460	9	140990428	T	C	439	0.0581	51	3.206	3.393180096	2.93E-06	7.58E-01	+	-	No
rs115658028	1	7687677	G	A	439	0.0068	7	9.079	2.935016895	4.58E-06	7.70E-01	+	-	No
rs979665	4	101881356	A	G	439	0.6446	320	1.805	1.158425521	2.90E-07	8.09E-01	+	-	No
rs72779724	16	10417409	C	T	439	0.0854	74	2.439	2.551152271	4.92E-06	8.44E-01	+	-	No
rs61546457	22	34469332	T	C	439	0.0501	44	3.574	2.979344989	1.86E-06	9.26E-01	+	-	No
rs35373244	22	34469348	G	A	439	0.0501	44	3.573	2.795992109	1.86E-06	9.27E-01	+	-	No
rs35482368	22	34469447	T	C	439	0.0501	44	3.529	2.050593821	1.98E-06	9.27E-01	+	-	No
rs117475675	13	73998852	T	C	439	0.0046	4	14.123	2.662263589	5.80E-08	9.78E-01	+	+	Yes
rs5994839	22	34484181	C	A	439	0.0399	35	3.904	2.09516586	3.25E-06	9.95E-01	+	-	No

Supplemental Table S18. Independent Replication
Chennai-1 cohort, case-control, comparing to discovery 12 month (12M) quantitative trait (QT)

Notes
Threshold for replicative significance: 5.00E-02 Total SNPs found: 58
Headers
rsID: reference SNP cluster ID, chr: chromosome number; pos_37, position of SNP on GRCh37 reference panel; REF and ALT, reference allele and alternate allele; n.obs: number of observations; caf: coding allele frequency; MAC: minor allele count; EST: estimated effect size; EST.SE: standard error of the effect size; Score.pval: probability value for the discovery cohort; pval.indianapolis-2: probability value for the replication cohort

rsID	chr	pos_37	REF	ALT	n.obs Indy-1	caf Indy-1	MAC Indy-1	Est Indy-1	Est.SE Indy-1	Score.pval Indy-1	pval.chennai
rs75626507	1	241415261	G	A	439	0.0034	4	11.84	2.58	4.55E-06	1.46E-01
rs5994839	22	34484181	C	A	439	0.0399	35	3.90	0.84	3.25E-06	1.98E-01
rs117699122	12	560262	T	C	439	0.0034	4	14.04	2.70	2.09E-07	2.96E-01
rs142956968	10	23490165	C	T	439	0.0159	14	6.66	1.30	2.87E-07	2.96E-01
rs145764464	10	23601847	G	A	439	0.0171	15	6.12	1.21	3.94E-07	2.96E-01
rs72825069	17	32600928	C	T	439	0.0216	20	5.46	1.11	7.80E-07	2.96E-01
rs146600651	1	152267708	C	T	439	0.0148	14	6.63	1.35	8.16E-07	2.96E-01
rs112507626	12	72710101	G	A	439	0.0228	19	5.24	1.07	1.05E-06	2.96E-01
rs144801217	2	130701392	T	C	439	0.0125	11	7.79	1.61	1.24E-06	2.96E-01
rs141411318	11	24605549	C	T	439	0.0228	20	5.16	1.08	1.70E-06	2.96E-01
rs72874841	11	24571002	C	T	439	0.0228	20	5.14	1.08	1.79E-06	2.96E-01
rs2229593	3	42906216	C	T	439	0.0273	24	4.57	0.97	2.39E-06	2.96E-01
rs77158902	17	32692036	C	T	439	0.0228	20	4.94	1.05	2.47E-06	2.96E-01
rs62582460	9	140990428	T	C	439	0.0581	51	3.21	0.69	2.93E-06	2.96E-01
rs112856203	12	72858864	T	C	439	0.0251	21	4.77	1.04	4.46E-06	2.96E-01
rs146298016	2	238565799	G	A	439	0.0080	7	8.85	1.93	4.64E-06	2.96E-01
rs139496704	12	124638643	A	G	439	0.0080	7	9.26	2.03	4.84E-06	2.96E-01
rs183086778	4	32610821	A	G	439	0.0046	5	12.66	2.32	5.02E-08	3.34E-01
rs139816293	20	30921343	C	T	439	0.0057	6	10.16	2.17	2.79E-06	3.34E-01
rs17875371	6	30460232	C	T	439	0.0182	16	6.01	1.29	3.09E-06	3.34E-01
rs139174841	16	59056275	G	A	439	0.0091	8	8.14	1.77	4.22E-06	3.34E-01
rs77846488	7	117211951	T	C	439	0.0046	4	10.80	2.29	2.46E-06	3.48E-01
rs75862363	7	117210400	G	A	439	0.0046	4	10.80	2.29	2.48E-06	3.48E-01
rs78282451	7	117193993	C	A	439	0.0046	4	10.80	2.29	2.50E-06	3.48E-01
rs12350891	9	82604621	G	A	439	0.0046	4	11.09	2.40	3.94E-06	3.54E-01
rs55939894	3	7963288	A	G	439	0.1879	165	-1.81	0.38	2.51E-06	3.58E-01
rs12212412	6	81976829	C	T	439	0.0091	8	8.76	1.75	5.44E-07	5.13E-01

rs76356799	3	179593768	G	A	439	0.0046	4	11.24	2.31	1.13E-06	5.13E-01
rs3795102	20	56258131	C	T	439	0.0046	4	12.14	2.35	2.50E-07	5.33E-01
rs61546457	22	34469332	T	C	439	0.0501	44	3.57	0.75	1.86E-06	5.34E-01
rs35482368	22	34469447	T	C	439	0.0501	44	3.53	0.74	1.98E-06	5.34E-01
rs979665	4	101881356	A	G	439	0.6446	320	1.81	0.35	2.90E-07	5.40E-01
rs147944608	10	110423709	C	T	439	0.0125	11	6.94	1.41	7.94E-07	5.84E-01
rs6724770	2	148227422	A	G	439	0.0273	24	4.34	0.93	2.85E-06	5.84E-01
rs10210722	2	148222783	T	C	439	0.0273	24	4.34	0.93	2.87E-06	5.84E-01
rs10175536	2	148217618	A	G	439	0.0273	24	4.30	0.92	3.07E-06	5.84E-01
rs117475675	13	73998852	T	C	439	0.0046	4	14.12	2.60	5.80E-08	6.04E-01
rs142497891	13	74036983	G	A	439	0.0046	4	12.13	2.33	1.84E-07	6.04E-01
rs56821264	6	148703901	C	T	439	0.0068	6	9.19	1.91	1.51E-06	6.04E-01
rs4896997	6	148683924	C	T	439	0.0068	6	9.03	1.90	1.92E-06	6.04E-01
rs17078283	6	148696920	C	T	439	0.0068	6	9.03	1.90	1.92E-06	6.04E-01
rs12193585	6	82002044	C	T	439	0.0103	9	7.85	1.69	3.27E-06	6.04E-01
rs10814594	9	37666667	T	C	439	0.1526	134	2.11	0.45	2.32E-06	6.05E-01
rs2057644	9	37665980	G	A	439	0.1526	134	2.11	0.45	2.32E-06	6.05E-01
rs2057643	9	37666010	T	C	439	0.1526	134	2.11	0.45	2.32E-06	6.05E-01
rs1125576	9	37667819	T	C	439	0.1526	134	2.11	0.45	2.33E-06	6.05E-01
rs5994128	22	17490932	A	G	439	0.7745	220	-2.14	0.44	1.02E-06	6.12E-01
rs60006744	9	37672691	G	T	439	0.0513	45	3.16	0.65	1.21E-06	6.74E-01
rs10973475	9	37666361	C	T	439	0.0513	45	3.16	0.65	1.22E-06	6.74E-01
rs10973466	9	37661110	G	A	439	0.0513	45	3.16	0.65	1.22E-06	7.07E-01
rs79914278	9	37658196	C	T	439	0.0513	45	3.14	0.65	1.28E-06	7.07E-01
rs35373244	22	34469348	G	A	439	0.0501	44	3.57	0.75	1.86E-06	7.35E-01
rs72878847	11	24620738	A	G	439	0.0239	20	5.03	1.07	2.71E-06	8.77E-01
rs144899985	4	32740350	A	G	439	0.0046	5	12.14	2.33	1.79E-07	9.56E-01
rs192772770	4	32809794	T	G	439	0.0057	5	10.94	2.16	4.32E-07	9.56E-01
rs76887296	4	32867005	C	T	439	0.0057	5	10.42	2.10	6.55E-07	9.56E-01
rs76487561	2	148214978	C	T	439	0.0285	25	4.23	0.88	1.39E-06	9.56E-01
rs75343152	2	148204747	T	C	439	0.0296	25	4.10	0.87	2.72E-06	9.56E-01
rs75330406	2	148238681	G	A	439	0.0273	24	4.35	0.93	2.72E-06	9.56E-01
rs78849093	2	148235591	C	A	439	0.0273	24	4.35	0.93	2.74E-06	9.56E-01
rs80339745	2	148227678	G	A	439	0.0273	24	4.34	0.93	2.85E-06	9.56E-01
rs75304512	2	148218006	A	G	439	0.0273	24	4.33	0.93	2.87E-06	9.56E-01
rs77592434	2	148226645	A	G	439	0.0273	24	4.34	0.93	2.87E-06	9.56E-01
rs77928469	2	148224118	C	T	439	0.0273	24	4.34	0.93	2.87E-06	9.56E-01
rs74380227	2	148220282	C	A	439	0.0273	24	4.33	0.93	2.89E-06	9.56E-01
rs74492945	2	148184439	G	A	439	0.0296	25	4.09	0.88	3.30E-06	9.56E-01
rs75380640	2	148185797	A	G	439	0.0296	25	4.09	0.88	3.30E-06	9.56E-01

rs2171551	3	7978078	C	A	439	0.1856	162	-1.79	0.38	3.28E-06	9.67E-01

Supplemental Table S19. Independent Replication
Chennai-1 cohort, case-control, comparing to discovery 3 month (3M) quantitative trait (QT)

Notes
Threshold for replicative significance: 5.00E-02 Total SNPs found: 122
Headers
rsID: reference SNP cluster ID, chr: chromosome number; pos_37, position of SNP on GRCh37 reference panel; REF and ALT, reference allele and alternate allele; n.obs: number of observations; caf: coding allele frequency; MAC: minor allele count; EST: estimated effect size; EST.SE: standard error of the effect size; Score.pval: probability value for the discovery cohort; pval.chennai: probability value for the replication cohort

rsID	chr	pos_37	REF	ALT	n.obs Indy-1	caf Indy-1	MAC Indy-1	Est Indy-1	Est.SE Indy-1	Score.pval Indy-1	pval.chennai
rs17017794	4	91825885	T	C	421	0.039192399	33	5.83240545	1.144508375	3.47E-07	6.39E-02
rs75334617	10	102956152	G	A	421	0.038004751	32	5.846034048	1.180273451	7.30E-07	6.39E-02
rs76098744	1	112349372	C	T	421	0.016627078	14	8.386124522	1.727041163	1.20E-06	6.78E-02
rs74683551	1	112354418	G	A	421	0.016627078	14	8.38252043	1.727058121	1.21E-06	6.78E-02
rs17510814	12	28468969	A	C	421	0.007125891	6	14.03879235	2.714096892	2.31E-07	6.78E-02
rs141756120	12	28511096	A	C	421	0.008313539	6	14.27840304	2.764585375	2.41E-07	6.78E-02
rs143371352	13	47383834	C	T	421	0.003562945	3	20.53416252	3.879289636	1.20E-07	6.78E-02
rs9643828	8	55529073	C	T	421	0.67695962	279	-2.423629403	0.468469114	2.30E-07	8.50E-02
rs147601511	8	22322319	A	G	421	0.004750594	4	16.44711814	3.317770381	7.15E-07	9.08E-02
rs112007361	11	99188380	A	C	421	0.03087886	26	6.356584678	1.228257582	2.28E-07	9.08E-02
rs76327548	12	101182966	G	A	421	0.013064133	11	9.178427144	1.932352307	2.04E-06	1.13E-01
rs10105693	8	55640472	C	T	421	0.328978622	276	2.292839101	0.449652819	3.41E-07	1.28E-01
rs11987234	8	55669829	A	G	421	0.328978622	276	2.295992587	0.448845319	3.13E-07	1.28E-01
rs7822082	8	55690220	T	C	421	0.330166271	277	2.357087802	0.448792891	1.50E-07	1.28E-01
rs149421869	2	53483429	G	T	421	0.008313539	8	11.73821862	2.472043763	2.05E-06	1.38E-01
rs17776100	7	6426479	G	A	421	0.029691211	25	6.63697397	1.321771845	5.13E-07	1.38E-01
rs2274997	1	229804646	A	G	421	0.041567696	35	5.587312281	1.123860297	6.64E-07	1.44E-01
rs74521112	11	99089147	G	T	421	0.032066508	27	6.256323868	1.227875864	3.48E-07	1.69E-01
rs79213709	11	99093455	G	A	421	0.032066508	26	6.17081884	1.23453654	5.78E-07	1.69E-01
rs1498183	8	55716905	C	T	421	0.394299287	332	2.024574602	0.442551408	4.77E-06	1.83E-01
rs858397	8	55614690	A	G	421	0.331353919	278	2.230134803	0.449903412	7.16E-07	1.85E-01
rs13278605	8	55688171	C	T	421	0.328978622	277	2.273773481	0.445931645	3.42E-07	1.85E-01
rs13276543	8	55688174	G	T	421	0.328978622	276	2.29263157	0.448230934	3.14E-07	1.85E-01
rs2375219	8	55698295	C	T	421	0.393111639	330	2.057001668	0.440730695	3.05E-06	1.85E-01
rs12502861	4	2426305	T	C	421	0.010688836	9	11.15884718	2.369618867	2.49E-06	1.98E-01
rs2274996	1	229804538	C	T	421	0.041567696	35	5.585274332	1.124063633	6.74E-07	2.43E-01
rs2891865	1	229806368	A	G	421	0.041567696	35	5.581813763	1.123789164	6.80E-07	2.43E-01
rs2385790	1	229807492	C	T	421	0.041567696	35	5.582248215	1.123947226	6.81E-07	2.43E-01
rs12024557	1	229812357	A	C	421	0.042755344	35	5.569309691	1.121957291	6.91E-07	2.43E-01
rs4562666	1	229824770	T	C	421	0.042755344	36	5.519225993	1.114574572	7.35E-07	2.43E-01
rs12036586	1	229826378	G	A	421	0.047505938	40	5.222121756	1.071140927	1.09E-06	2.43E-01
rs16850124	1	229831331	T	C	421	0.043942993	37	5.180956622	1.094351878	2.20E-06	2.43E-01
rs12045643	1	229834050	C	T	421	0.042755344	36	5.481491517	1.107089479	7.37E-07	2.43E-01
rs2375536	8	55640722	T	C	421	0.347980998	292	2.126358065	0.448570911	2.13E-06	2.58E-01
rs4737674	8	55661654	C	A	421	0.330166271	277	2.305595541	0.449219885	2.86E-07	2.58E-01
rs13277510	8	55674149	G	A	421	0.330166271	277	2.304947516	0.449099416	2.86E-07	2.58E-01
rs12548593	8	55674617	G	T	421	0.332541568	279	2.282699606	0.447524018	3.38E-07	2.58E-01
rs1812506	8	55676101	A	G	421	0.345605701	291	2.122588678	0.446617796	2.01E-06	2.58E-01
rs16920698	8	55678434	G	A	421	0.330166271	277	2.304926953	0.449081772	2.86E-07	2.58E-01
rs1561297	8	55678538	A	C	421	0.332541568	279	2.28315237	0.447431459	3.35E-07	2.58E-01
rs4737676	8	55679546	G	A	421	0.330166271	277	2.304920569	0.44908192	2.86E-07	2.58E-01
rs2083123	8	55680318	C	T	421	0.332541568	279	2.279107938	0.447074908	3.44E-07	2.58E-01
rs983248	8	55680792	C	T	421	0.330166271	277	2.302604284	0.448714611	2.87E-07	2.58E-01
rs1391463	8	55681876	T	G	421	0.330166271	277	2.302588654	0.448713075	2.87E-07	2.58E-01
rs4737201	8	55691458	C	T	421	0.330166271	277	2.301977341	0.44869179	2.89E-07	2.58E-01
rs7843693	8	55692112	G	A	421	0.397862233	334	1.998567561	0.436953938	4.79E-06	2.58E-01
rs1396896	8	55695310	A	G	421	0.397862233	334	1.998533266	0.436904523	4.78E-06	2.58E-01
rs1391462	8	55699781	C	A	421	0.397862233	334	1.998476605	0.436960267	4.79E-06	2.58E-01
rs12678939	8	55705021	A	G	421	0.394299287	331	2.019756183	0.442200513	4.94E-06	2.58E-01
rs12266995	10	24852783	T	C	421	0.03087886	26	5.967825345	1.293119589	3.93E-06	2.85E-01
rs149421869	2	52563371	C	T	421	0.003562945	3	19.74461693	3.726349214	1.17E-07	2.96E-01
rs187520610	2	53360041	G	A	421	0.004750594	4	18.80103964	3.193645952	3.93E-09	2.96E-01
rs116651654	4	163238743	C	T	421	0.007125891	6	15.62236937	3.066244515	3.49E-07	2.96E-01
rs290120	5	163268244	T	G	421	0.007125891	6	13.3587764	2.832608007	2.40E-06	2.96E-01
rs73586304	6	142839425	C	T	421	0.003562945	3	18.24051164	3.99428202	4.96E-06	2.96E-01
rs181259864	7	97488823	C	A	421	0.003562945	3	17.13608209	3.682427888	3.26E-06	2.96E-01

rs192750513	7	97577830	A	G	421	0.003562945	3	17.18924692	3.725142178	3.94E-06	2.96E-01
rs151115079	11	18655741	T	C	421	0.004750594	5	17.8273587	3.268020498	4.89E-08	2.96E-01
rs138414342	11	18679398	G	A	421	0.004750594	5	18.15825054	3.282149429	3.16E-08	2.96E-01
rs151323346	12	21012024	T	C	421	0.005938242	4	15.89038883	3.380348678	2.59E-06	2.96E-01
rs75186966	13	47395758	A	C	421	0.003562945	3	20.22813447	3.832255231	1.30E-07	2.96E-01
rs146442492	15	58982115	C	T	421	0.027315914	24	7.059258577	1.516613162	3.25E-06	2.96E-01
rs2327968	20	15813491	C	T	421	0.024940618	21	6.158998564	1.343908089	4.59E-06	2.96E-01
rs2876414	20	15813704	G	T	421	0.022565321	19	6.819109065	1.485309072	4.41E-06	2.96E-01
rs140788628	20	15858501	C	A	421	0.010688836	8	11.80392839	2.282639604	2.33E-07	2.96E-01
rs2365739	1	62484462	G	A	421	0.021377672	18	6.868159166	1.43887996	1.81E-06	3.34E-01
rs1856085	1	104114545	G	A	421	0.002375297	3	21.51143997	4.267478979	4.64E-07	3.34E-01
rs143597860	1	104157143	A	G	421	0.002375297	3	21.57481541	4.276037896	4.52E-07	3.34E-01
rs144541665	1	104310729	G	A	421	0.002375297	3	21.87346108	4.303398392	3.72E-07	3.34E-01
rs113651406	4	990967	C	T	421	0.003562945	3	19.40540264	3.763582603	2.52E-07	3.34E-01
rs140797780	8	22087792	C	T	421	0.003562945	3	19.29083956	3.691477848	1.73E-07	3.34E-01
rs76904423	12	101188744	G	A	421	0.010688836	10	9.805762313	2.115689082	3.57E-06	3.34E-01
rs150586237	6	24491348	C	T	421	0.003562945	3	22.04614425	3.780947933	5.51E-09	3.46E-01
rs423841	8	55556069	G	A	421	0.662707838	291	-2.149572575	0.458468456	2.75E-06	3.49E-01
rs433324	8	55564609	A	G	421	0.666270784	287	-2.248346209	0.458142524	9.22E-07	3.49E-01
rs369623	8	55571940	A	C	421	0.666270784	287	-2.235981486	0.45415557	8.51E-07	3.49E-01
rs446222	8	55574960	G	A	421	0.666270784	288	-2.247819003	0.453979954	7.37E-07	3.49E-01
rs432393	8	55580298	C	T	421	0.662707838	291	-2.187538166	0.450963464	1.23E-06	3.49E-01
rs3098298	8	55582838	C	T	421	0.662707838	291	-2.187563503	0.450947906	1.23E-06	3.49E-01
rs367179	8	55587616	T	C	421	0.662707838	291	-2.187563503	0.450947906	1.23E-06	3.49E-01
rs382476	8	55590975	G	A	421	0.666270784	288	-2.24819379	0.454055398	7.37E-07	3.49E-01
rs384543	8	55591609	G	A	421	0.666270784	288	-2.24819379	0.454055398	7.37E-07	3.49E-01
rs405226	8	55592336	A	G	421	0.662707838	291	-2.188130533	0.45101874	1.23E-06	3.49E-01
rs384127	8	55597489	G	A	421	0.666270784	288	-2.248155875	0.454048112	7.37E-07	3.49E-01
rs2375537	8	55619508	C	T	421	0.332541568	280	2.238768477	0.449388495	6.30E-07	3.49E-01
rs720372	8	55628637	G	A	421	0.346793349	292	2.098727338	0.448862589	2.93E-06	3.49E-01
rs1437781	8	55629852	T	C	421	0.332541568	280	2.23854673	0.449407657	6.32E-07	3.49E-01
rs1595406	8	55630615	A	G	421	0.345605701	291	2.100188892	0.448300638	2.80E-06	3.49E-01
rs1437782	8	55632762	C	T	421	0.330166271	278	2.2579585	0.450987807	5.54E-07	3.49E-01
rs10958428	8	55685641	A	G	421	0.333729216	280	2.239968848	0.445686734	5.01E-07	3.49E-01
rs75689761	7	18406573	C	T	421	0.008313539	7	11.84829172	2.413202584	9.12E-07	4.27E-01
rs77346868	7	18406599	A	G	421	0.009501188	8	11.02101286	2.264811725	1.14E-06	4.27E-01
rs78225611	7	18407464	A	C	421	0.009501188	8	11.02943875	2.266224663	1.13E-06	4.27E-01
rs77300464	7	18408761	A	G	421	0.007125891	6	14.66597943	2.616951597	2.09E-08	4.27E-01
rs79602997	7	18410250	G	A	421	0.008313539	7	11.86082112	2.419016265	9.43E-07	4.27E-01
rs75773869	7	18410845	G	T	421	0.008313539	7	11.86427156	2.41902322	9.36E-07	4.27E-01
rs75606013	7	18414613	G	A	421	0.007125891	6	14.66171186	2.616924205	2.11E-08	4.27E-01
rs61434999	7	18418351	A	G	421	0.008313539	7	11.84115106	2.412840272	9.22E-07	4.27E-01
rs78907958	7	18425017	T	G	421	0.008313539	7	12.09013217	2.405149614	4.99E-07	4.27E-01
rs76526501	7	18431110	G	A	421	0.007125891	6	14.83973124	2.577811398	8.58E-09	4.27E-01
rs74455595	7	18431784	A	G	421	0.007125891	6	14.83973124	2.577811398	8.58E-09	4.27E-01
rs79182806	7	18433827	T	C	421	0.008313539	7	12.09726027	2.389568893	4.14E-07	4.27E-01
rs10279777	7	18441589	G	A	421	0.009501188	8	12.86813351	2.279616935	1.65E-08	4.27E-01
rs77867199	7	18442275	G	T	421	0.010688836	9	10.82212821	2.13363365	3.93E-07	4.27E-01
rs80156375	7	18443215	A	C	421	0.009501188	8	11.5406255	2.25971308	3.27E-07	4.27E-01
rs17169602	7	18446741	G	A	421	0.009501188	8	12.72737168	2.259939835	1.78E-08	4.27E-01
rs10486295	7	18446807	G	A	421	0.009501188	8	12.76229003	2.264804562	1.75E-08	4.27E-01
rs75090694	7	18447436	A	G	421	0.008313539	7	13.83140053	2.414270813	1.01E-08	4.27E-01
rs12315614	12	64920957	C	A	421	0.076009501	64	3.777106623	0.820195905	4.12E-06	5.64E-01
rs147630370	4	87450675	T	C	421	0.004750594	4	16.00136328	3.23339786	7.47E-07	6.04E-01
rs142993106	4	90957372	G	A	421	0.017814727	15	8.084636111	1.733061622	3.09E-06	6.04E-01
rs146526206	4	90993018	T	C	421	0.017814727	15	8.091766558	1.695352855	1.82E-06	6.04E-01
rs139493286	18	28816019	G	A	421	0.003562945	3	17.46030927	3.750764282	3.24E-06	7.11E-01
rs1686289	14	46260982	G	A	421	0.678147268	277	-2.255153927	0.486259757	3.52E-06	8.30E-01
rs176786	14	46282970	T	C	421	0.32304038	268	2.284756409	0.49171936	3.38E-06	8.30E-01
rs79539453	11	125266353	C	T	421	0.002375297	3	20.82206158	4.345424064	1.65E-06	9.23E-01
rs78547898	22	32824278	G	A	421	0.003562945	3	20.90983951	4.155382737	4.85E-07	9.27E-01
rs7104959	11	129846126	C	T	421	0.003562945	3	17.92046105	3.843668505	3.13E-06	9.37E-01
rs10494861	1	205331874	G	A	421	0.003562945	3	19.18204982	4.072913404	2.48E-06	9.56E-01
rs1877768	6	16534923	C	T	421	0.017814727	15	8.287546847	1.768307483	2.78E-06	9.56E-01
rs111391231	7	89497045	T	C	421	0.014251781	12	8.317177547	1.807207138	4.18E-06	9.56E-01
rs111900874	7	89516669	G	A	421	0.014251781	12	8.424240128	1.822583342	3.80E-06	9.56E-01
rs115348382	1	9655903	G	A	421	0.003562945	3	18.21074945	3.847216981	2.21E-06	NA
rs186532456	1	18948328	C	T	421	0.003562945	3	20.17303433	4.117377453	9.61E-07	NA
rs562032622	1	18959333	A	C	421	0.003562945	3	21.0267115	4.319750303	1.13E-06	NA

rs149493615	1	79880089	G	A	421	0.008313539	8	11.750632	2.434512435	1.39E-06	NA
rs143811231	1	79968131	T	C	421	0.008313539	8	11.57474094	2.420660266	1.74E-06	NA
rs34270375	1	89370702	G	A	421	0.026128266	21	7.711470175	1.572167924	9.34E-07	NA
rs187518659	1	99455745	T	G	421	0.009501188	8	11.68196232	2.465645372	2.16E-06	NA
rs140420703	1	102803484	T	G	421	0.004750594	5	16.48765399	3.462138915	1.91E-06	NA
rs563167766	1	102866365	G	A	421	0.002375297	3	19.41573898	4.242403955	4.73E-06	NA
rs77180278	1	102961882	T	C	421	0.028503563	24	7.336199959	1.374041118	9.34E-08	NA
rs112351653	1	103220360	T	C	421	0.028503563	24	7.423869471	1.332867724	2.55E-08	NA
rs180926150	1	103226326	C	T	421	0.002375297	3	19.64191801	4.247936485	3.77E-06	NA
rs114413507	1	103419168	T	C	421	0.028503563	24	7.42216029	1.332949535	2.57E-08	NA
rs116672066	1	103472916	G	A	421	0.027315914	23	8.047694764	1.37187844	4.46E-09	NA
rs111928960	1	103633635	G	A	421	0.028503563	22	8.602572083	1.45827516	3.65E-09	NA
rs113221952	1	103753974	A	G	421	0.021377672	19	7.923507043	1.614216816	9.17E-07	NA
rs76617932	1	180930424	T	C	421	0.011876485	9	10.54496084	2.250407262	2.79E-06	NA
rs183180157	1	181247121	A	C	421	0.009501188	7	12.61385999	2.689211211	2.72E-06	NA
rs375790303	1	184530482	G	A	421	0.008313539	7	11.42403934	2.497191924	4.77E-06	NA
rs138480898	1	184955657	C	T	421	0.003562945	3	16.9873974	3.669723728	3.67E-06	NA
rs145766563	1	185129502	G	A	421	0.003562945	4	16.86821019	3.541270076	1.90E-06	NA
rs147032554	1	186148864	T	G	421	0.003562945	3	18.16260897	3.654028209	6.68E-07	NA
rs180989936	1	193044178	A	G	421	0.004750594	4	17.17935787	3.702154942	3.48E-06	NA
rs559559983	1	246977132	C	A	421	0.007125891	5	13.81113197	2.897255878	1.87E-06	NA
rs550763536	2	7452347	T	G	421	0.005938242	5	14.48716465	3.103497679	3.04E-06	NA
rs558553658	2	15682724	C	T	421	0.004750594	3	17.10991069	3.291080613	2.01E-07	NA
rs536781978	2	29733681	A	G	421	0.003562945	3	17.58708236	3.702594509	2.03E-06	NA
rs568321148	2	29870348	T	G	421	0.003562945	3	17.71250463	3.720125102	1.92E-06	NA
rs76777840	2	48312950	G	A	421	0.005938242	5	14.35007842	2.892789399	7.03E-07	NA
rs145080832	2	48473143	G	A	421	0.004750594	4	17.16184772	3.257095367	1.37E-07	NA
rs184220112	2	48631743	C	A	421	0.004750594	4	16.48790432	3.22267606	3.12E-07	NA
rs189890455	2	48655397	C	T	421	0.005938242	5	15.14673914	2.993550834	4.20E-07	NA
rs181193202	2	52527354	T	C	421	0.003562945	3	19.63120884	3.747760153	1.62E-07	NA
rs190193113	2	53085778	G	A	421	0.003562945	3	19.95336019	3.761955902	1.13E-07	NA
rs146479102	2	65825759	G	A	421	0.005938242	5	15.48467677	3.269801992	2.18E-06	NA
rs528288879	2	65875930	C	T	421	0.005938242	6	14.78780847	2.951219288	5.42E-07	NA
rs11690187	2	67565909	A	C	421	0.005938242	5	13.63105325	2.975132201	4.61E-06	NA
rs151272830	2	67682724	G	T	421	0.007125891	6	13.33234787	2.848060758	2.85E-06	NA
rs186142189	2	67702707	G	A	421	0.007125891	6	12.73619271	2.714053683	2.70E-06	NA
rs184200893	2	69260913	C	T	421	0.003562945	3	19.95803558	4.219054866	2.24E-06	NA
rs111927235	2	74483954	A	G	421	0.004750594	4	17.04689553	3.461118894	8.43E-07	NA
rs111838310	2	74673491	C	A	421	0.004750594	5	17.79639376	3.48429066	3.26E-07	NA
rs112983626	2	74697150	G	A	421	0.004750594	4	17.78480792	3.485324945	3.35E-07	NA
rs113006316	2	74802360	A	G	421	0.002375297	3	22.88867923	4.924113346	3.35E-06	NA
rs17746486	2	95722609	C	T	421	0.032066508	29	6.908513578	1.434771746	1.47E-06	NA
rs76554191	2	95967628	G	A	421	0.040380048	34	5.721480386	1.238917397	3.87E-06	NA
rs140352232	2	108038112	G	A	421	0.002375297	3	21.05765113	4.560131294	3.88E-06	NA
rs116189766	2	126393864	T	C	421	0.002375297	3	21.19076397	4.360372887	1.17E-06	NA
rs139877408	2	129606273	A	G	421	0.002375297	3	20.30671294	4.430524974	4.58E-06	NA
rs142894171	2	151438271	G	T	421	0.003562945	3	18.07103941	3.74586836	1.41E-06	NA
rs541508507	2	170023265	G	A	421	0.004750594	4	15.46300334	3.273896179	2.32E-06	NA
rs142549310	2	170030506	C	T	421	0.004750594	4	15.25017126	3.249836338	2.70E-06	NA
rs556293455	2	176950420	G	A	421	0.003562945	3	14.38604633	2.969984018	1.27E-06	NA
rs184098071	2	177116420	G	A	421	0.003562945	3	13.94772404	2.86782452	1.15E-06	NA
rs532416695	2	177486790	G	A	421	0.003562945	3	19.87266561	3.767376828	1.33E-07	NA
rs112557251	2	188375400	T	C	421	0.002375297	3	20.65100643	4.448501059	3.45E-06	NA
rs185158855	2	223650026	C	A	421	0.004750594	4	16.66788644	3.602665239	3.72E-06	NA
rs185510569	2	223814861	G	A	421	0.003562945	3	19.58869056	3.604909775	5.51E-08	NA
rs147559909	2	237051523	T	C	421	0.005938242	5	17.08409752	3.032091952	1.76E-08	NA
rs181217257	2	239989119	C	T	421	0.004750594	4	21.56773677	3.472969885	5.29E-10	NA
rs188076929	2	239993719	T	C	421	0.004750594	4	19.97756143	3.272467463	1.03E-09	NA
rs112475378	3	1626661	T	C	421	0.016627078	14	9.550859286	1.976281377	1.35E-06	NA
rs145676540	3	2044635	C	T	421	0.005938242	5	13.98065798	2.884473769	1.25E-06	NA
rs146007933	3	28227423	T	C	421	0.021377672	18	7.511494525	1.554428045	1.35E-06	NA
rs73057656	3	33940571	A	G	421	0.036817102	33	5.923832897	1.27589711	3.44E-06	NA
rs73085348	3	42711221	A	G	421	0.011876485	11	10.13931704	2.035045707	6.28E-07	NA
rs142684595	3	55319301	T	C	421	0.005938242	5	16.77294307	3.113647549	7.17E-08	NA
rs80203220	3	122722331	C	T	421	0.005938242	6	13.42604201	2.842186558	2.31E-06	NA
rs192443987	3	135532345	G	A	421	0.004750594	4	17.75402845	3.675643022	1.36E-06	NA
rs148248743	3	136134595	C	T	421	0.002375297	3	22.83937904	4.620446891	7.69E-07	NA
rs576124203	3	141841196	T	G	421	0.003562945	3	19.4039925	3.843925823	4.47E-07	NA
rs545552231	3	141864350	C	T	421	0.003562945	3	19.8641332	3.945825635	4.80E-07	NA

rs193153124	3	148330710	A	G	421	0.005938242	5	14.36573132	3.065145937	2.78E-06	NA
rs188720948	3	150069880	T	C	421	0.003562945	3	17.7032653	3.597228742	8.59E-07	NA
rs16823323	3	153657202	G	A	421	0.016627078	14	9.484640653	1.717697949	3.36E-08	NA
rs139943877	3	155451289	G	A	421	0.007125891	7	13.0535902	2.760415942	2.26E-06	NA
rs187047882	3	164285621	G	A	421	0.003562945	3	18.56413613	3.997254169	3.41E-06	NA
rs141169929	3	164808462	A	G	421	0.004750594	5	14.84541783	3.173883899	2.91E-06	NA
rs186649043	3	174932293	C	T	421	0.003562945	3	19.21342086	4.065887859	2.30E-06	NA
rs189709453	3	177574308	G	A	421	0.003562945	3	21.65814876	4.05916142	9.52E-08	NA
rs186767531	3	177639777	T	C	421	0.003562945	3	21.20132426	4.047807581	1.63E-07	NA
rs182868205	3	177712448	C	T	421	0.003562945	3	20.95487681	3.978418395	1.39E-07	NA
rs191792521	3	195646605	G	A	421	0.008313539	6	13.95154842	2.964435992	2.52E-06	NA
rs189765693	4	4324793	T	C	421	0.004750594	4	15.51913413	3.299246074	2.55E-06	NA
rs183962155	4	21259643	A	C	421	0.005938242	6	13.76460079	2.972477589	3.64E-06	NA
rs113751774	4	23428854	C	T	421	0.007125891	7	14.91032743	2.966574592	5.01E-07	NA
rs113063005	4	23509067	T	C	421	0.005938242	6	21.2073894	3.155200957	1.80E-11	NA
rs145875128	4	32073136	G	A	421	0.003562945	3	24.47998599	4.606392132	1.07E-07	NA
rs143287889	4	35572280	C	T	421	0.002375297	3	19.96741	4.34406	4.30E-06	NA
rs77141817	4	37053759	T	C	421	0.003562945	3	20.61102923	3.642148656	1.52E-08	NA
rs190822761	4	37099356	G	T	421	0.003562945	3	20.58463536	3.643465595	1.61E-08	NA
rs999769259	4	62511965	G	A	421	0.003562945	3	22.12467252	4.775978806	3.61E-06	NA
rs147171192	4	89135588	A	G	421	0.007125891	5	13.30440472	2.89758492	4.40E-06	NA
rs145116559	4	112677596	T	C	421	0.003562945	4	18.24412236	3.993253602	4.91E-06	NA
rs181415102	4	112689266	T	C	421	0.003562945	4	18.26310193	3.993879436	4.81E-06	NA
rs191423619	4	126516627	G	T	421	0.002375297	3	21.69461097	4.206673365	2.51E-07	NA
rs149298750	4	127158701	A	C	421	0.003562945	3	22.18405204	4.265732461	1.99E-07	NA
rs112679237	4	139140860	T	C	421	0.017814727	18	8.712027524	1.676806707	2.04E-07	NA
rs531769270	4	154411043	T	C	421	0.003562945	3	16.59453383	3.519360917	2.41E-06	NA
rs567982164	5	25631297	G	A	421	0.002375297	3	21.8356286	4.367067031	5.73E-07	NA
rs191986449	5	25745687	C	T	421	0.004750594	3	18.49787925	4.036798308	4.60E-06	NA
rs185771987	5	73285489	T	C	421	0.003562945	3	18.23448239	3.976750144	4.53E-06	NA
rs139360368	5	73372109	A	C	421	0.003562945	4	18.35904869	3.78701272	1.25E-06	NA
rs181933850	5	91465647	A	G	421	0.008313539	7	11.04365858	2.375442162	3.33E-06	NA
rs190190051	5	91475485	G	A	421	0.008313539	7	11.05898873	2.383179753	3.48E-06	NA
rs182531466	5	91530073	C	A	421	0.007125891	6	12.68049781	2.756852606	4.23E-06	NA
rs187236873	5	91530447	G	A	421	0.007125891	6	12.44906998	2.617809375	1.98E-06	NA
rs183816745	5	91631888	A	G	421	0.005938242	5	17.34879088	3.201955563	6.02E-08	NA
rs111407636	5	95080029	C	T	421	0.003562945	3	18.64277396	3.741680518	6.28E-07	NA
rs111676272	5	95094298	C	A	421	0.003562945	3	18.6068625	3.744297477	6.72E-07	NA
rs75848314	5	95098340	T	C	421	0.003562945	3	20.0998481	3.787004507	1.11E-07	NA
rs111846247	5	95111643	T	C	421	0.003562945	3	20.48657718	3.854672052	1.07E-07	NA
rs137873790	5	97087041	A	G	421	0.013064133	11	9.222408811	2.010953789	4.52E-06	NA
rs189912648	5	134765439	C	T	421	0.003562945	4	16.97325991	3.608462274	2.55E-06	NA
rs191006910	5	154216009	G	A	421	0.003562945	3	17.61738031	3.790496891	3.36E-06	NA
rs74343174	5	161493182	C	A	421	0.005938242	5	14.76718237	3.139134082	2.55E-06	NA
rs775626702	5	162630067	A	C	421	0.003562945	3	18.94841366	4.070233717	3.23E-06	NA
rs371245624	5	162880956	T	C	421	0.003562945	3	19.47959308	4.065720108	1.66E-06	NA
rs545428520	5	167821266	T	C	421	0.003562945	4	21.56603503	3.664079879	3.96E-09	NA
rs528404963	5	167852025	T	C	421	0.003562945	4	21.13939767	3.653035168	7.17E-09	NA
rs72832764	5	170004673	G	A	421	0.003562945	3	18.21597569	3.881967434	2.70E-06	NA
rs72837643	5	170188955	T	C	421	0.003562945	4	16.93028738	3.677434793	4.15E-06	NA
rs142311947	5	177384469	G	A	421	0.007125891	8	12.03390127	2.576181159	2.99E-06	NA
rs151015676	5	177390937	T	G	421	0.002375297	3	20.14213671	4.370744819	4.06E-06	NA
rs571986619	6	85678832	A	G	421	0.002375297	3	20.78580673	4.343019831	1.70E-06	NA
rs56224400	6	98092675	T	C	421	0.016627078	14	8.443306398	1.722514712	9.50E-07	NA
rs147627638	6	99173116	A	G	421	0.007125891	6	13.07853062	2.793642539	2.85E-06	NA
rs141326851	6	134833127	A	C	421	0.016627078	14	8.578496884	1.810532677	2.16E-06	NA
rs146048121	6	142198955	G	A	421	0.002375297	3	20.02143793	4.300860255	3.24E-06	NA
rs142106992	6	142269885	C	A	421	0.003562945	4	22.83458862	3.675869896	5.23E-10	NA
rs72983831	6	142307100	T	G	421	0.005938242	6	15.67332386	3.17294911	7.83E-07	NA
rs72986533	6	142611258	T	C	421	0.008313539	8	13.28127256	2.530636288	1.54E-07	NA
rs148532212	6	165499857	T	C	421	0.003562945	3	19.35354569	3.709456929	1.81E-07	NA
rs117498042	6	165514281	C	T	421	0.003562945	3	19.33547097	3.70913521	1.86E-07	NA
rs148153037	6	167501386	G	A	421	0.008313539	8	14.33592462	2.421281019	3.20E-09	NA
rs184487573	6	167513471	A	G	421	0.007125891	7	13.50096351	2.592680141	1.92E-07	NA
rs187978759	7	11711845	G	A	421	0.003562945	3	20.4555522	4.3637719	2.76E-06	NA
rs117166500	7	17052778	G	T	421	0.007125891	7	12.51468647	2.626941975	1.90E-06	NA
rs55844051	7	23360363	T	C	421	0.003562945	3	18.3875896	3.862687038	1.93E-06	NA
rs1301444047	7	36574504	G	A	421	0.042755344	43	5.128422094	1.088355486	2.45E-06	NA
rs62447184	7	36574504	G	A	421	0.042755344	43	5.128422094	1.088355486	2.45E-06	NA

rs574076561	7	49544747	A	G	421	0.003562945	3	19.64347663	4.25225723	3.85E-06	NA
rs539713344	7	100474786	G	A	421	0.002375297	3	20.04475604	4.219575451	2.03E-06	NA
rs188028357	7	100668425	C	T	421	0.002375297	3	21.84255775	4.357918094	5.38E-07	NA
rs536023430	7	146865072	T	C	421	0.003562945	3	19.40386834	4.130848564	2.64E-06	NA
rs187384541	8	1632241	A	G	421	0.035629454	26	6.887451174	1.477766677	3.15E-06	NA
rs575473987	8	5577494	C	T	421	0.003562945	3	22.22486534	3.886257536	1.07E-08	NA
rs139062456	8	13251991	C	T	421	0.017814727	15	7.835158086	1.696874229	3.89E-06	NA
rs185874707	8	18030674	C	T	421	0.011876485	10	9.139347655	1.949186444	2.75E-06	NA
rs188415494	8	25613298	C	T	421	0.003562945	3	18.51694779	3.991486498	3.50E-06	NA
rs1221830047	8	55690220	T	C	421	0.330166271	277	2.357087802	0.448792891	1.50E-07	NA
rs117816016	8	103751262	C	T	421	0.003562945	3	19.88070227	4.269602116	3.22E-06	NA
rs567383525	8	115029494	C	T	421	0.003562945	3	17.74552267	3.719706274	1.84E-06	NA
rs545550279	8	115552429	G	T	421	0.003562945	3	19.18124208	4.192129875	4.75E-06	NA
rs536803366	8	123001411	T	C	421	0.002375297	3	19.20986385	4.009520573	1.66E-06	NA
rs555249476	8	123001411	T	C	421	0.002375297	3	19.20986385	4.009520573	1.66E-06	NA
rs532513136	8	135813748	C	A	421	0.003562945	3	19.94905941	4.031185989	7.47E-07	NA
rs532730683	9	1784492	G	T	421	0.003562945	3	18.72683804	3.831467497	1.02E-06	NA
rs540065886	9	2770228	T	C	421	0.003562945	3	19.94891509	4.188028045	1.90E-06	NA
rs543844012	9	30107023	C	T	421	0.003562945	3	18.11604845	3.944610301	4.38E-06	NA
rs148556485	9	84023826	A	C	421	0.002375297	3	21.38001525	4.266183157	5.40E-07	NA
rs140782222	9	84028894	T	C	421	0.002375297	3	21.79152597	4.31928461	4.53E-07	NA
rs188034471	9	85937714	G	A	421	0.004750594	4	14.75599992	3.149251127	2.79E-06	NA
rs190294315	9	85945465	C	T	421	0.005938242	5	14.56642764	2.881266486	4.29E-07	NA
rs545690161	9	93030699	G	A	421	0.005938242	6	17.14368742	3.003187786	1.14E-08	NA
rs565682685	9	93222328	T	C	421	0.004750594	4	18.93839699	3.720617399	3.58E-07	NA
rs183737367	9	93330047	T	C	421	0.003562945	3	23.34523057	4.244714311	3.80E-08	NA
rs187213609	9	93415465	C	T	421	0.003562945	3	23.19753039	4.274126556	5.72E-08	NA
rs150027952	9	103385416	A	G	421	0.002375297	3	21.86166485	4.514646431	1.28E-06	NA
rs146207930	9	129042336	A	G	421	0.007125891	6	11.86243214	2.515024654	2.40E-06	NA
rs78296164	9	135266715	C	T	421	0.008313539	7	11.60953385	2.339175566	6.94E-07	NA
rs77871739	9	138552309	G	A	421	0.004750594	4	15.07246035	3.236061811	3.20E-06	NA
rs118040657	10	3472846	C	T	421	0.008313539	8	12.11477125	2.644346939	4.62E-06	NA
rs184425183	10	13457520	A	G	421	0.003562945	3	22.66831744	3.775496477	1.92E-09	NA
rs184458518	10	13471195	T	G	421	0.003562945	3	22.80157891	3.771239424	1.48E-09	NA
rs117998251	10	13497976	C	T	421	0.003562945	3	22.95433112	3.774804885	1.19E-09	NA
rs117025967	10	20207052	C	A	421	0.013064133	11	9.669735635	1.927439002	5.25E-07	NA
rs529011661	10	20372316	G	A	421	0.004750594	4	15.63934688	3.312533901	2.34E-06	NA
rs138249376	10	63515829	T	G	421	0.004750594	4	16.43007109	3.594667912	4.86E-06	NA
rs140277951	10	82359100	G	A	421	0.008313539	6	13.74631584	2.643339318	1.99E-07	NA
rs566018180	10	86755052	C	T	421	0.003562945	3	18.56355199	4.04215862	4.38E-06	NA
rs140706881	10	96217535	G	A	421	0.004750594	4	18.97480496	3.932889306	1.40E-06	NA
rs117913371	10	102918486	G	A	421	0.024940618	21	7.866095548	1.407855116	2.31E-08	NA
rs752259256	10	104925319	T	C	421	0.003562945	3	20.42586391	4.454115907	4.52E-06	NA
rs180828621	10	124533409	G	A	421	0.007125891	6	15.13805735	2.799576273	6.40E-08	NA
rs147393020	10	124779274	A	G	421	0.005938242	5	14.1853505	2.959808958	1.65E-06	NA
rs193093906	10	126705489	G	A	421	0.009501188	9	10.52195366	2.25840517	3.18E-06	NA
rs541653703	11	18701786	G	A	421	0.004750594	4	18.31694986	3.272434185	2.18E-08	NA
rs118093638	11	18718324	C	T	421	0.005938242	5	14.26572587	2.912965064	9.72E-07	NA
rs181812512	11	66665729	C	T	421	0.003562945	3	19.50931683	4.242198164	4.25E-06	NA
rs529345909	11	67110852	A	G	421	0.003562945	3	18.70153001	4.092060355	4.87E-06	NA
rs544042801	11	68460243	G	A	421	0.004750594	4	15.88698661	3.405190192	3.08E-06	NA
rs149949098	11	95099866	G	A	421	0.016627078	13	8.559276254	1.818833079	2.53E-06	NA
rs148781275	11	103640603	A	G	421	0.004750594	5	15.71118528	3.321838325	2.25E-06	NA
rs141281289	11	123693024	A	G	421	0.005938242	6	15.81151802	3.088952198	3.08E-07	NA
rs528140343	11	125719227	A	C	421	0.003562945	3	18.28355207	3.727380292	9.33E-07	NA
rs546409459	11	125754989	A	G	421	0.003562945	3	18.91954737	4.002868361	2.28E-06	NA
rs528609331	11	125842195	C	T	421	0.003562945	3	19.58124182	3.948327054	7.07E-07	NA
rs189360484	12	1870510	A	G	421	0.004750594	4	16.8078794	3.314248553	3.95E-07	NA
rs141754456	12	20151132	T	C	421	0.007125891	7	12.14603335	2.328429207	1.82E-07	NA
rs118184666	12	20424749	G	A	421	0.007125891	6	10.95098026	2.324772421	2.47E-06	NA
rs549931083	12	20516286	A	C	421	0.003562945	3	14.32826937	2.798913946	3.07E-07	NA
rs371879555	12	23015962	T	C	421	0.003562945	3	20.10337646	4.200023233	1.70E-06	NA
rs183466664	12	26821687	A	G	421	0.004750594	4	16.01818822	3.456090156	3.57E-06	NA
rs77353774	12	28248852	G	A	421	0.007125891	6	13.1054362	2.679541026	1.00E-06	NA
rs113167689	12	28435962	C	T	421	0.007125891	6	14.13641354	2.733356286	2.32E-07	NA
rs117991215	12	28511473	T	C	421	0.008313539	6	14.33366601	2.77918736	2.50E-07	NA
rs191930622	12	48284655	G	A	421	0.002375297	3	19.67835791	4.306103677	4.88E-06	NA
rs56302696	12	48292830	G	A	421	0.002375297	3	19.89590409	4.315847075	4.03E-06	NA
rs185620578	12	48569399	C	T	421	0.002375297	3	20.72322668	4.39505442	2.42E-06	NA

rs190806532	12	48862147	G	T	421	0.002375297	3	21.32695498	4.438560431	1.55E-06	NA
rs568658857	12	49853998	G	A	421	0.005938242	5	14.23611926	3.094234224	4.21E-06	NA
rs137880949	12	63306297	T	C	421	0.004750594	3	21.60840343	4.135891677	1.75E-07	NA
rs191053292	12	63445280	T	C	421	0.003562945	3	22.9266488	4.114397317	2.51E-08	NA
rs182437250	12	63608466	T	C	421	0.004750594	4	18.80834508	3.793435539	7.12E-07	NA
rs180764936	12	101498621	T	C	421	0.003562945	3	19.34730528	4.020023799	1.49E-06	NA
rs185855183	12	101505126	C	T	421	0.003562945	3	18.73977272	3.861373435	1.22E-06	NA
rs139598422	13	23887014	A	G	421	0.003562945	4	20.48865215	3.641671944	1.84E-08	NA
rs150077525	13	57979549	A	G	421	0.007125891	7	12.99848746	2.713124141	1.66E-06	NA
rs534845494	13	58213864	A	G	421	0.004750594	5	15.94317648	3.184840074	5.56E-07	NA
rs140062526	13	59286033	G	A	421	0.005938242	5	14.09260875	3.020529034	3.08E-06	NA
rs546286713	13	91411079	G	A	421	0.004750594	4	15.70278409	3.361121605	2.98E-06	NA
rs567080482	13	95075405	T	C	421	0.004750594	4	15.49995607	3.316208371	2.95E-06	NA
rs142928734	13	101601082	G	A	421	0.003562945	3	17.00959296	3.700449886	4.29E-06	NA
rs556680896	13	101602415	C	T	421	0.003562945	3	17.0254659	3.702867989	4.27E-06	NA
rs184265355	13	108011352	A	C	421	0.005938242	6	14.42717187	2.918297916	7.67E-07	NA
rs572961122	13	108012985	C	T	421	0.005938242	6	13.33548956	2.858276075	3.08E-06	NA
rs528809914	13	113041256	G	A	421	0.003562945	3	19.50485519	3.768671108	2.27E-07	NA
rs180765647	13	114427311	G	T	421	0.002375297	3	20.12465213	4.373978059	4.20E-06	NA
rs138215817	14	22641516	A	G	421	0.004750594	4	16.31101896	3.273670217	6.28E-07	NA
rs74704551	14	30161887	C	T	421	0.003562945	3	24.19907048	4.31064693	1.98E-08	NA
rs176783	14	46280913	A	G	421	0.321852732	267	2.280101466	0.493763736	3.88E-06	NA
rs143048774	14	46294660	A	C	421	0.317102138	265	2.284822992	0.49630858	4.15E-06	NA
rs428110	14	46294660	A	C	421	0.317102138	265	2.284822992	0.49630858	4.15E-06	NA
rs116862847	14	64141677	C	T	421	0.007125891	7	15.3160369	2.951942625	2.12E-07	NA
rs569916471	14	75891342	G	A	421	0.004750594	5	15.64040878	3.353713303	3.11E-06	NA
rs113767990	14	81717563	G	A	421	0.004750594	4	14.58111547	3.193630952	4.98E-06	NA
rs190251199	14	105590577	T	C	421	0.003562945	3	20.01252919	4.016271726	6.27E-07	NA
rs185155853	15	41244100	C	T	421	0.004750594	5	15.91646574	3.408386314	3.02E-06	NA
rs144026361	15	41248669	C	T	421	0.004750594	5	16.13729945	3.437346588	2.67E-06	NA
rs558614420	15	41810870	C	T	421	0.005938242	6	13.97508315	3.02772732	3.92E-06	NA
rs138109686	15	42051442	A	G	421	0.005938242	6	13.59567051	2.96761718	4.62E-06	NA
rs145896760	15	42119222	G	A	421	0.005938242	6	13.36888249	2.923468092	4.81E-06	NA
rs140642138	15	42125165	G	A	421	0.005938242	6	13.58322205	2.962740492	4.55E-06	NA
rs6080	15	58837933	C	A	421	0.043942993	36	5.664767454	1.239325304	4.86E-06	NA
rs145439370	15	58879765	T	C	421	0.03087886	26	6.893288912	1.403391166	9.02E-07	NA
rs149425014	15	58951660	T	C	421	0.026128266	22	7.552247919	1.583206043	1.84E-06	NA
rs193253461	15	59229353	A	G	421	0.013064133	12	10.53744676	2.074047629	3.76E-07	NA
rs184117160	15	59404306	C	T	421	0.014251781	11	10.34172362	2.133663377	1.25E-06	NA
rs80292573	15	59435086	T	G	421	0.034441805	30	6.553234912	1.326965834	7.87E-07	NA
rs182303755	15	59634792	A	C	421	0.013064133	12	10.54799166	2.093592905	4.70E-07	NA
rs138217865	15	94392882	C	T	421	0.004750594	4	16.70964097	3.451938959	1.29E-06	NA
rs553840536	16	25697895	A	G	421	0.003562945	3	19.62034417	4.23547954	3.61E-06	NA
rs183817723	16	59302775	C	T	421	0.003562945	4	17.5860507	3.734191901	2.48E-06	NA
rs144954214	16	76179362	A	G	421	0.002375297	3	21.81132631	4.439425294	8.96E-07	NA
rs529523094	16	77715551	A	G	421	0.003562945	3	18.15843462	3.726174193	1.10E-06	NA
rs146728064	17	19265440	G	A	421	0.007125891	6	12.64004111	2.734671147	3.80E-06	NA
rs184613584	17	48508221	A	C	421	0.007125891	6	14.13779737	2.821223402	5.41E-07	NA
rs191271637	17	52123260	A	G	421	0.003562945	3	18.379836	3.920833552	2.76E-06	NA
rs185819304	18	27001580	G	A	421	0.003562945	3	19.94011134	3.945019527	4.32E-07	NA
rs187942235	18	27030430	C	T	421	0.003562945	3	20.39686581	3.969630251	2.77E-07	NA
rs143538552	18	29050262	A	G	421	0.003562945	3	19.14572179	3.636797033	1.41E-07	NA
rs373746073	18	29058384	C	A	421	0.003562945	3	19.08471968	3.637980642	1.55E-07	NA
rs146333745	18	55497457	C	T	421	0.003562945	4	17.42157837	3.567823349	1.04E-06	NA
rs185464792	19	18797371	C	T	421	0.002375297	3	22.58960154	4.501914727	5.23E-07	NA
rs186768950	19	18806124	C	A	421	0.002375297	3	22.71520611	4.507764788	4.68E-07	NA
rs541288561	19	18869445	T	G	421	0.004750594	5	15.61190124	3.131834835	6.20E-07	NA
rs559008174	19	18876059	C	T	421	0.004750594	5	15.60256615	3.12812006	6.11E-07	NA
rs570407448	19	18880030	G	A	421	0.004750594	5	15.4977984	3.124125552	7.02E-07	NA
rs546144116	19	19563339	C	T	421	0.002375297	3	23.13548147	4.471627577	2.29E-07	NA
rs560206697	19	20729098	C	T	421	0.002375297	3	24.99899431	4.553506728	4.02E-08	NA
rs111285015	19	23123198	G	A	421	0.003562945	3	27.33476425	4.62083983	3.31E-09	NA
rs1008091735	19	31090099	T	C	421	0.003562945	3	18.46892428	3.690799086	5.61E-07	NA
rs148433854	19	31096478	G	A	421	0.003562945	3	18.62675851	3.688067545	4.41E-07	NA
rs559228693	20	15963329	G	A	421	0.005938242	5	14.36435511	3.081964969	3.15E-06	NA
rs557092705	20	34189911	C	T	421	0.003562945	3	19.47695457	4.248007298	4.54E-06	NA
rs184785969	21	17171431	C	A	421	0.003562945	3	19.87364864	3.706839369	8.26E-08	NA
rs117280553	21	17207163	T	C	421	0.003562945	3	20.08741695	3.702748263	5.80E-08	NA
rs79486609	21	17245006	G	A	421	0.003562945	3	20.66248857	3.77824957	4.53E-08	NA

rs73227413	21	23136973	G	A	421	0.034441805	28	5.962055143	1.271501762	2.75E-06	NA
rs75024143	21	23156546	G	T	421	0.014251781	12	9.812542411	2.068454196	2.10E-06	NA
rs192134381	21	23450714	T	C	421	0.003562945	3	21.78881202	3.731253572	5.23E-09	NA
rs397836601	21	23450714	T	C	421	0.003562945	3	21.78881202	3.731253572	5.23E-09	NA
rs118183140	21	35477486	C	T	421	0.020190024	17	7.589324801	1.561222322	1.17E-06	NA
rs183586634	21	38763032	G	A	421	0.005938242	5	14.31322017	2.992100476	1.72E-06	NA
rs117185941	21	38766484	G	A	421	0.005938242	5	15.38087864	3.171430132	1.24E-06	NA
rs1329159859	21	38766484	G	A	421	0.005938242	5	15.38087864	3.171430132	1.24E-06	NA
rs118084887	21	38863820	T	C	421	0.005938242	5	14.93826162	3.136747415	1.91E-06	NA
rs150539922	21	43276916	T	C	421	0.003562945	3	16.66969372	3.64477381	4.79E-06	NA
rs113625788	22	19969182	C	T	421	0.008313539	7	11.80848286	2.429415606	1.17E-06	NA
rs796777817	22	32824278	G	A	421	0.003562945	3	20.90983951	4.155382737	4.85E-07	NA
rs541680196	22	40528090	G	A	421	0.005938242	5	14.38678079	2.949036589	1.07E-06	NA
rs185139807	22	40594781	G	A	421	0.005938242	5	14.43305263	2.953126858	1.02E-06	NA
rs141127122	22	40604439	G	A	421	0.004750594	4	15.38248751	3.30083549	3.16E-06	NA
rs148998974	22	40620530	A	G	421	0.005938242	5	14.51295325	2.949760239	8.65E-07	NA
rs555040883	22	40631476	G	A	421	0.004750594	4	15.42730692	3.305427169	3.05E-06	NA
rs182959028	22	45823032	T	C	421	0.008313539	7	11.94446103	2.531460004	2.38E-06	NA
rs150946694	22	46853180	T	C	421	0.004750594	4	15.30824787	3.291250592	3.30E-06	NA

Supplemental Table S20. Independent Replication
Chennai-2 cohort, case-control, comparing to discovery 12 month (12M) quantitative trait (QT)

Notes
Threshold for replicative significance: 5.00E-02 Total SNPs found: 58
Headers
rsID: reference SNP cluster ID, chr: chromosome number; pos_37, position of SNP on GRCh37 reference panel; REF and ALT, reference allele and alternate allele; n.obs: number of observations; caf: coding allele frequency; MAC: minor allele count; EST: estimated effect size; EST.SE: standard error of the effect size; Score.pval: probability value for the discovery cohort; pval.chennai: probability value for the replication cohort

rsID	chr	pos_37	REF	ALT	n.obs Indy-1	caf Indy-1	MAC Indy-1	Est Indy-1	Est.SE Indy-1	Score.pval Indy-1	pval.chennai
rs2057643	9	37666010	T	C	439	0.1526	134	2.109	0.446	2.32E-06	1.88E-02
rs2057644	9	37665980	G	A	439	0.1526	134	2.109	0.446	2.32E-06	2.44E-02
rs60006744	9	37672691	G	T	439	0.0513	45	3.164	0.652	1.21E-06	3.42E-02
rs61546457	22	34469332	T	C	439	0.0501	44	3.574	0.750	1.86E-06	3.58E-02
rs10973475	9	37666361	C	T	439	0.0513	45	3.158	0.651	1.22E-06	4.09E-02
rs979665	4	101881356	A	G	439	0.6446	320	1.805	0.352	2.90E-07	4.61E-02
rs147944608	10	110423709	C	T	439	0.0125	11	6.938	1.405	7.94E-07	5.91E-02
rs2229593	3	42906216	C	T	439	0.0273	24	4.570	0.969	2.39E-06	7.23E-02
rs10973466	9	37661110	G	A	439	0.0513	45	3.156	0.650	1.22E-06	9.31E-02
rs35373244	22	34469348	G	A	439	0.0501	44	3.573	0.749	1.86E-06	9.41E-02
rs1125576	9	37667819	T	C	439	0.1526	134	2.108	0.446	2.33E-06	1.19E-01
rs79914278	9	37658196	C	T	439	0.0513	45	3.143	0.649	1.28E-06	1.21E-01
rs75862363	7	117210400	G	A	439	0.0046	4	10.799	2.293	2.48E-06	1.71E-01
rs2148140	9	37673839	C	T	439	0.0513	45	3.164	0.652	1.21E-06	1.92E-01
rs12193585	6	82002044	C	T	439	0.0103	9	7.847	1.686	3.27E-06	1.93E-01
rs56821264	6	148703901	C	T	439	0.0068	6	9.190	1.910	1.51E-06	2.05E-01
rs17875371	6	30460232	C	T	439	0.0182	16	6.010	1.288	3.09E-06	2.12E-01
rs35482368	22	34469447	T	C	439	0.0501	44	3.529	0.742	1.98E-06	2.49E-01
rs78282451	7	117193993	C	A	439	0.0046	4	10.796	2.293	2.50E-06	2.54E-01
rs77846488	7	117211951	T	C	439	0.0046	4	10.804	2.293	2.46E-06	2.85E-01
rs5994128	22	17490932	A	G	439	0.7745	220	-2.143	0.438	1.02E-06	3.04E-01
rs72878847	11	24620738	A	G	439	0.0239	20	5.034	1.073	2.71E-06	3.07E-01
rs142497891	13	74036983	G	A	439	0.0046	4	12.128	2.326	1.84E-07	3.25E-01
rs10814594	9	37666667	T	C	439	0.1526	134	2.109	0.446	2.32E-06	3.27E-01
rs4896997	6	148683924	C	T	439	0.0068	6	9.030	1.896	1.92E-06	3.29E-01
rs17078283	6	148696920	C	T	439	0.0068	6	9.029	1.896	1.92E-06	3.42E-01
rs113399724	9	37674357	C	T	439	0.0513	45	3.164	0.652	1.21E-06	3.50E-01
rs77928469	2	148224118	C	T	439	0.0273	24	4.336	0.926	2.87E-06	3.56E-01
rs75626507	1	241415261	G	A	439	0.0034	4	11.843	2.583	4.55E-06	3.59E-01
rs78849093	2	148235591	C	A	439	0.0273	24	4.349	0.927	2.74E-06	3.80E-01
rs146600651	1	152267708	C	T	439	0.0148	14	6.634	1.345	8.16E-07	3.88E-01
rs117699122	12	560262	T	C	439	0.0034	4	14.040	2.705	2.09E-07	4.03E-01
rs74380227	2	148220282	C	A	439	0.0273	24	4.333	0.926	2.89E-06	4.03E-01
rs12212412	6	81976829	C	T	439	0.0091	8	8.761	1.749	5.44E-07	4.19E-01
rs71568803	6	81993718	A	G	439	0.0091	8	8.718	1.742	5.62E-07	4.19E-01
rs117475675	13	73998852	T	C	439	0.0046	4	14.123	2.603	5.80E-08	4.23E-01
rs139816293	20	30921343	C	T	439	0.0057	6	10.161	2.168	2.79E-06	4.23E-01
rs72779724	16	10417409	C	T	439	0.0854	74	2.439	0.534	4.92E-06	4.27E-01
rs112856203	12	72858864	T	C	439	0.0251	21	4.771	1.040	4.46E-06	4.36E-01
rs1442156642	9	37664624	G	A	439	0.1526	134	2.109	0.446	2.31E-06	4.40E-01
rs55939894	3	7963288	A	G	439	0.1879	165	-1.810	0.385	2.51E-06	4.83E-01
rs76356799	3	179593768	G	A	439	0.0046	4	11.244	2.310	1.13E-06	5.66E-01
rs75343152	2	148204747	T	C	439	0.0296	25	4.095	0.873	2.72E-06	5.73E-01
rs118171627	6	143615890	C	A	439	0.0034	3	14.339	3.069	2.97E-06	5.74E-01
rs139174841	16	59056275	G	A	439	0.0091	8	8.139	1.769	4.22E-06	5.76E-01
rs147669485	18	26603650	G	T	439	0.0034	4	12.526	2.551	9.11E-07	5.80E-01
rs112796175	12	72790349	C	T	439	0.0251	21	4.767	1.022	3.11E-06	5.83E-01
rs141411318	11	24605549	C	T	439	0.0228	20	5.164	1.079	1.70E-06	5.86E-01
rs5994839	22	34484181	C	A	439	0.0399	35	3.904	0.839	3.25E-06	5.88E-01
rs76487561	2	148214978	C	T	439	0.0285	25	4.230	0.876	1.39E-06	5.96E-01
rs2171551	3	7978078	C	A	439	0.1856	162	-1.790	0.385	3.28E-06	6.24E-01
rs11920261	3	7977211	G	T	439	0.1856	162	-1.791	0.385	3.27E-06	6.93E-01
rs10210722	2	148222783	T	C	439	0.0273	24	4.335	0.926	2.87E-06	6.94E-01
rs10175536	2	148217618	A	G	439	0.0273	24	4.303	0.922	3.07E-06	7.04E-01
rs1258074269	2	148223792	A	C	439	0.0273	24	4.335	0.926	2.87E-06	7.15E-01
rs3795102	20	56258131	C	T	439	0.0046	4	12.139	2.354	2.50E-07	8.15E-01
rs12350891	9	82604621	G	A	439	0.0046	4	11.090	2.403	3.94E-06	9.15E-01
rs4131286	6	148688963	G	T	439	0.0068	6	9.028	1.896	1.92E-06	9.26E-01

Supplemental Table S21. Independent Replication
Chennai-2 cohort, case-control, comparing to discovery 3 month 3M) quantitative trait (QT)

Notes
Threshold for replicative significance: 5.00E-02 Total SNPs found: 123 Total risk loci found: 41
Bold boxed rows: SNPs that attained adjusted p-values of replication significance
Headers
rsID: reference SNP cluster ID, chr: chromosome number; pos_37, position of SNP on GRCh37 reference panel; REF and ALT, reference allele and alternate allele; n.obs: number of observations; caf: coding allele frequency;
MAC: minor allele count; EST: estimated effect size; EST.SE: standard error of the effect size; Score.pval: probability value for the discovery cohort; pval.chennai: probability value for the replication cohort;
adj pval SNPs: p-value adjusted for multiple corrections based on number of SNPs; adj pval loci: p-value adjusted for multiple corrections based on risk loci

rsID	chr	pos_37	REF	ALT	n.obs Indy-1	caf Indy-1	MAC Indy-1	Est Indy-1	Est.SE Indy-1	Score.pval Indy-1	pval.chennai	adj pval SNPs	adj pval loci	Closest gene	Prioritized gene (AOP-expressed)
rs7822082	8	55690220	T	C	421	0.33016627	277	2.3570878	0.44879289	1.50E-07	8.66E-06	1.07E-03	3.55E-04	RP1	SOX17
rs1561297	8	55678538	A	C	421	0.33254157	279	2.28315237	0.44743146	3.35E-07	7.41E-05	9.11E-03	3.04E-03	RP1	SOX17
rs193153124	3	148330710	A	G	421	0.00593824	5	14.3657313	3.06514594	2.78E-06	3.56E-04	4.38E-02	1.46E-02	AGTR1	AGTR1
rs12678939	8	55705021	A	G	421	0.39429929	331	2.01975618	0.44220051	4.94E-06	1.66E-03	2.04E-01	6.81E-02	RP1	SOX17
rs446222	8	55574960	G	A	421	0.66627078	288	-2.247819	0.45397995	7.37E-07	1.95E-03	2.40E-01	8.00E-02	RP1	SOX17
rs2375537	8	55619508	C	T	421	0.33254157	280	2.23876848	0.44938849	6.30E-07	3.33E-03	4.10E-01	1.37E-01	RP1	SOX17
rs2375536	8	55640722	T	C	421	0.347981	292	2.12635806	0.44857091	2.13E-06	3.38E-03	4.16E-01	1.39E-01	RP1	SOX17
rs1812506	8	55676101	A	G	421	0.3456057	291	2.12258868	0.4466178	2.01E-06	5.06E-03	6.22E-01	2.07E-01	RP1	SOX17
rs858397	8	55614690	A	G	421	0.33135392	278	2.2301348	0.44990341	7.16E-07	1.03E-02	1.00E+00	4.22E-01	RP1	SOX17
rs382476	8	55590975	G	A	421	0.66627078	288	-2.2481938	0.4540554	7.37E-07	1.07E-02	1.00E+00	4.39E-01	RP1	SOX17
rs1595406	8	55630615	A	G	421	0.3456057	291	2.10018889	0.44830064	2.80E-06	1.27E-02	1.00E+00	5.21E-01	RP1	SOX17
rs13278605	8	55688171	C	T	421	0.32897862	277	2.27377348	0.44593165	3.42E-07	1.37E-02	1.00E+00	5.62E-01	RP1	SOX17
rs13276543	8	55688174	G	T	421	0.32897862	276	2.29263157	0.44823093	3.14E-07	1.37E-02	1.00E+00	5.62E-01	RP1	SOX17
rs405226	8	55592336	A	G	421	0.66270784	291	-2.1881305	0.45101874	1.23E-06	1.41E-02	1.00E+00	5.78E-01	RP1	SOX17
rs1391462	8	55699781	C	A	421	0.39786223	334	1.9984766	0.43696027	4.79E-06	1.84E-02	1.00E+00	7.54E-01	RP1	SOX17
rs1437782	8	55632762	C	T	421	0.33016627	278	2.2579585	0.45098781	5.54E-07	1.98E-02	1.00E+00	8.12E-01	RP1	SOX17
rs2375219	8	55698295	C	T	421	0.39311164	330	2.05700167	0.44073069	3.05E-06	2.18E-02	1.00E+00	8.94E-01	RP1	SOX17
rs16920698	8	55678434	G	A	421	0.33016627	277	2.30492695	0.44908177	2.86E-07	2.28E-02	1.00E+00	9.35E-01	RP1	SOX17
rs4737676	8	55679546	G	A	421	0.33016627	277	2.30492057	0.44908192	2.86E-07	2.53E-02	1.00E+00	1.00E+00	RP1	SOX17
rs1396896	8	55695310	A	G	421	0.39786223	334	1.99853327	0.43690452	4.78E-06	2.66E-02	1.00E+00	1.00E+00	RP1	SOX17
rs9643828	8	55529073	C	T	421	0.67695962	279	-2.4236294	0.46846911	2.30E-07	2.99E-02	1.00E+00	1.00E+00	RP1	SOX17
rs1437781	8	55629852	T	C	421	0.33254157	280	2.23854673	0.44940766	6.32E-07	3.11E-02	1.00E+00	1.00E+00	RP1	SOX17
rs12548593	8	55674617	G	T	421	0.33254157	279	2.28269961	0.44752402	3.38E-07	3.14E-02	1.00E+00	1.00E+00	RP1	SOX17
rs2083123	8	55680318	C	T	421	0.33254157	279	2.27910794	0.44707491	3.44E-07	3.14E-02	1.00E+00	1.00E+00	RP1	SOX17
rs720372	8	55628637	G	A	421	0.34679335	292	2.09872734	0.44886259	2.93E-06	3.58E-02	1.00E+00	1.00E+00	RP1	SOX17
rs7843693	8	55692112	G	A	421	0.39786223	334	1.99856756	0.43695394	4.79E-06	3.58E-02	1.00E+00	1.00E+00	RP1	SOX17
rs13277510	8	55674149	G	A	421	0.33016627	277	2.30494752	0.44909942	2.86E-07	3.62E-02	1.00E+00	1.00E+00	RP1	SOX17
rs983248	8	55680792	C	T	421	0.33016627	277	2.30260428	0.44871461	2.87E-07	3.75E-02	1.00E+00	1.00E+00	RP1	SOX17
rs432393	8	55580298	C	T	421	0.66270784	291	-2.1875382	0.45096346	1.23E-06	4.10E-02	1.00E+00	1.00E+00	RP1	SOX17
rs12024557	1	229812357	A	C	421	0.04275534	35	5.56930969	1.12195729	6.91E-07	4.44E-02	1.00E+00	1.00E+00	URB2	URB2
rs16823323	3	153657202	G	A	421	0.01662708	14	9.48464065	1.71769795	3.36E-08	5.30E-02	1.00E+00	1.00E+00	ARHGEF26	ARHGEF26
rs79182806	7	18433827	T	C	421	0.00831354	7	12.0972603	2.38956889	4.14E-07	5.30E-02	1.00E+00	1.00E+00	HDAC9	HDAC9
rs369623	8	55571940	A	C	421	0.66627078	287	-2.2359815	0.45415557	8.51E-07	6.33E-02	1.00E+00	1.00E+00	RP1	SOX17
rs384127	8	55597489	G	A	421	0.66627078	288	-2.2481559	0.45404811	7.37E-07	6.35E-02	1.00E+00	1.00E+00	RP1	SOX17
rs184613584	17	48508221	A	C	421	0.00712589	6	14.1377974	2.8212234	5.41E-07	6.48E-02	1.00E+00	1.00E+00	ACSF2	ACSF2
rs182959028	22	45823032	T	C	421	0.00831354	7	11.944461	2.53146	2.38E-06	6.48E-02	1.00E+00	1.00E+00	RIBC2	RIBC2
rs433324	8	55564609	A	G	421	0.66627078	287	-2.2483462	0.45814252	9.22E-07	6.83E-02	1.00E+00	1.00E+00	RP1	SOX17
rs1301444047	7	36574504	G	A	421	0.04275534	43	5.12842209	1.08835549	2.45E-06	7.23E-02	1.00E+00	1.00E+00	AOAH	AOAH
rs62447184	7	36574504	G	A	421	0.04275534	43	5.12842209	1.08835549	2.45E-06	7.23E-02	1.00E+00	1.00E+00	AOAH	AOAH
rs541288561	19	18869445	T	G	421	0.00475059	5	15.6119012	3.13183483	6.20E-07	7.23E-02	1.00E+00	1.00E+00	CRTC1	CRTC1
rs570407448	19	18880030	G	A	421	0.00475059	5	15.4977984	3.12412555	7.02E-07	7.23E-02	1.00E+00	1.00E+00	CRTC1	CRTC1

rs423841	8	55556069	G	A	421	0.66270784	291	-2.1495726	0.45846846	2.75E-06	7.39E-02	1.00E+00	1.00E+00	RP1	SOX17
rs117913371	10	102918486	G	A	421	0.02494062	21	7.86609555	1.40785512	2.31E-08	7.46E-02	1.00E+00	1.00E+00	LINC01514	KAZALD1
rs147601511	8	22322319	A	G	421	0.00475059	4	16.4471181	3.31777038	7.15E-07	7.96E-02	1.00E+00	1.00E+00	PPP3CC	PPP3CC
rs1391463	8	55681876	T	G	421	0.33016627	277	2.30258865	0.44871307	2.87E-07	9.01E-02	1.00E+00	1.00E+00	RP1	SOX17
rs78225611	7	18407464	A	C	421	0.00950119	8	11.0294387	2.26622466	1.13E-06	1.05E-01	1.00E+00	1.00E+00	HDAC9	HDAC9
rs77300464	7	18408761	A	G	421	0.00712589	6	14.6659794	2.6169516	2.09E-08	1.10E-01	1.00E+00	1.00E+00	HDAC9	HDAC9
rs4737674	8	55661654	C	A	421	0.33016627	277	2.30559554	0.44921988	2.86E-07	1.16E-01	1.00E+00	1.00E+00	RP1	SOX17
rs11987234	8	55669829	A	G	421	0.32897862	276	2.29599259	0.44884532	3.13E-07	1.20E-01	1.00E+00	1.00E+00	RP1	SOX17
rs17169602	7	18446741	G	A	421	0.00950119	8	12.7273717	2.25993984	1.78E-08	1.25E-01	1.00E+00	1.00E+00	HDAC9	HDAC9
rs3098298	8	55582838	C	T	421	0.66270784	291	-2.1875635	0.45094791	1.23E-06	1.25E-01	1.00E+00	1.00E+00	RP1	SOX17
rs61434999	7	18418351	A	G	421	0.00831354	7	11.8411511	2.41284027	9.22E-07	1.28E-01	1.00E+00	1.00E+00	HDAC9	HDAC9
rs77346868	7	18406599	A	G	421	0.00950119	8	11.0210129	2.26481172	1.14E-06	1.31E-01	1.00E+00	1.00E+00	HDAC9	HDAC9
rs139493286	18	28816019	G	A	421	0.00356295	3	17.4603093	3.75076428	3.24E-06	1.33E-01	1.00E+00	1.00E+00	DSG1	DSG1
rs1877768	6	16534923	C	T	421	0.01781473	15	8.28754685	1.76830748	2.78E-06	1.60E-01	1.00E+00	1.00E+00	ATXN1	ATXN1
rs1686289	14	46260982	G	A	421	0.67814727	277	-2.2551539	0.48625976	3.52E-06	1.63E-01	1.00E+00	1.00E+00	LINC02303	MIS18BP1
rs150586237	6	24491348	C	T	421	0.00356295	3	22.0461443	3.78094793	5.51E-09	1.64E-01	1.00E+00	1.00E+00	GPLD1	GPLD1
rs10105693	8	55640472	C	T	421	0.32897862	276	2.2928391	0.44965282	3.41E-07	1.68E-01	1.00E+00	1.00E+00	RP1	SOX17
rs79539453	11	125266353	C	T	421	0.0023753	3	20.8220616	4.34542406	1.65E-06	1.73E-01	1.00E+00	1.00E+00	PKNOX2	PKNOX2
rs10279777	7	18441589	G	A	421	0.00950119	8	12.8681335	2.27961694	1.65E-08	1.97E-01	1.00E+00	1.00E+00	HDAC9	HDAC9
rs367179	8	55587616	T	C	421	0.66270784	291	-2.1875635	0.45094791	1.23E-06	2.03E-01	1.00E+00	1.00E+00	RP1	SOX17
rs384543	8	55591609	G	A	421	0.66627078	288	-2.2481938	0.4540554	7.37E-07	2.18E-01	1.00E+00	1.00E+00	RP1	SOX17
rs2385790	1	229807492	C	T	421	0.0415677	35	5.58224822	1.12394723	6.81E-07	2.21E-01	1.00E+00	1.00E+00	URB2	URB2
rs78907958	7	18425017	T	G	421	0.00831354	7	12.0901322	2.40514961	4.99E-07	2.36E-01	1.00E+00	1.00E+00	HDAC9	HDAC9
rs2274997	1	229804646	A	G	421	0.0415677	35	5.58731228	1.1238603	6.64E-07	2.43E-01	1.00E+00	1.00E+00	URB2	URB2
rs1498183	8	55716905	C	T	421	0.39429929	332	2.0245746	0.44255141	4.77E-06	2.53E-01	1.00E+00	1.00E+00	RP1	SOX17
rs10958428	8	55685641	A	G	421	0.33372922	280	2.23996885	0.44568673	5.01E-07	2.78E-01	1.00E+00	1.00E+00	RP1	SOX17
rs80156375	7	18443215	A	C	421	0.00950119	8	11.5406255	2.25971308	3.27E-07	2.83E-01	1.00E+00	1.00E+00	HDAC9	HDAC9
rs79213709	11	99093455	G	A	421	0.03206651	26	6.17081884	1.23453654	5.78E-07	2.83E-01	1.00E+00	1.00E+00	CNTN5	CNTN5
rs12502861	4	2426305	T	C	421	0.01068884	9	11.1588472	2.36961887	2.49E-06	3.29E-01	1.00E+00	1.00E+00	CFAP99	CFAP99
rs557092705	20	34189911	C	T	421	0.00356295	3	19.4769546	4.2480073	4.54E-06	3.29E-01	1.00E+00	1.00E+00	FER1L4	ERGIC3
rs75773869	7	18410845	G	T	421	0.00831354	7	11.8642716	2.41902322	9.36E-07	3.31E-01	1.00E+00	1.00E+00	HDAC9	HDAC9
rs74455595	7	18431784	A	G	421	0.00712589	6	14.8397312	2.5778114	8.58E-09	3.40E-01	1.00E+00	1.00E+00	HDAC9	HDAC9
rs79602997	7	18410250	G	A	421	0.00831354	7	11.8608211	2.41901626	9.43E-07	3.50E-01	1.00E+00	1.00E+00	HDAC9	HDAC9
rs75090694	7	18447436	A	G	421	0.00831354	7	13.8314005	2.41427081	1.01E-08	3.59E-01	1.00E+00	1.00E+00	HDAC9	HDAC9
rs4737201	8	55691458	C	T	421	0.33016627	277	2.30197734	0.44869179	2.89E-07	3.65E-01	1.00E+00	1.00E+00	RP1	SOX17
rs17746486	2	95722609	C	T	421	0.03206651	29	6.90851358	1.43477175	1.47E-06	3.80E-01	1.00E+00	1.00E+00	MAL	MAL
rs118040657	10	3472846	C	T	421	0.00831354	8	12.1147712	2.64434694	4.62E-06	3.80E-01	1.00E+00	1.00E+00	LOC105376360	KLF6
rs113651406	4	990967	C	T	421	0.00356295	3	19.4054026	3.7635826	2.52E-07	3.88E-01	1.00E+00	1.00E+00	IDUA	IDUA
rs75689761	7	18406573	C	T	421	0.00831354	7	11.8482917	2.41320258	9.12E-07	3.91E-01	1.00E+00	1.00E+00	HDAC9	HDAC9
rs147171192	4	89135588	A	G	421	0.00712589	5	13.3044047	2.89758492	4.40E-06	4.15E-01	1.00E+00	1.00E+00	ABCG2	ABCG2
rs10486295	7	18446807	G	A	421	0.00950119	8	12.76229	2.26480456	1.75E-08	4.22E-01	1.00E+00	1.00E+00	HDAC9	HDAC9
rs74683551	1	112354418	G	A	421	0.01662708	14	8.38252043	1.72705812	1.21E-06	4.23E-01	1.00E+00	1.00E+00	KCND3	KCND3
rs147630370	4	87450675	T	C	421	0.00475059	4	16.0013633	3.23339786	7.47E-07	4.23E-01	1.00E+00	1.00E+00	MAPK10	MAPK10
rs111391231	7	89497045	T	C	421	0.01425178	12	8.31717755	1.80720714	4.18E-06	4.23E-01	1.00E+00	1.00E+00	STEAP2	STEAP2
rs112007361	11	99188380	A	C	421	0.03087886	26	6.35658468	1.22825758	2.28E-07	4.23E-01	1.00E+00	1.00E+00	CNTN5	CNTN5
rs10494861	1	205331874	G	A	421	0.00356295	3	19.1820498	4.0729134	2.48E-06	4.27E-01	1.00E+00	1.00E+00	KLHDC8A	KLHDC8A
rs142894171	2	151438271	G	T	421	0.00356295	3	18.0710394	3.74586836	1.41E-06	4.27E-01	1.00E+00	1.00E+00	LINC02612	RND3
rs176786	14	46282970	T	C	421	0.32304038	268	2.28475641	0.49171936	3.38E-06	4.32E-01	1.00E+00	1.00E+00	LINC02303	MIS18BP1
rs76327548	12	101182966	G	A	421	0.01306413	11	9.17842714	1.93235231	2.04E-06	4.39E-01	1.00E+00	1.00E+00	ANO4	ANO4
rs12045643	1	229834050	C	T	421	0.04275534	36	5.48149152	1.10708948	7.37E-07	4.45E-01	1.00E+00	1.00E+00	URB2	URB2
rs140782222	9	84028894	T	C	421	0.0023753	3	21.791526	4.31928461	4.53E-07	4.53E-01	1.00E+00	1.00E+00	TLE1	TLE1
rs12266995	10	24852783	T	C	421	0.03087886	26	5.96782535	1.29311959	3.93E-06	4.91E-01	1.00E+00	1.00E+00	ARHGAP21	ARHGAP21
rs176783	14	46280913	A	G	421	0.32185273	267	2.28010147	0.49376374	3.88E-06	5.10E-01	1.00E+00	1.00E+00	LINC02303	MIS18BP1
rs77867199	7	18442275	G	T	421	0.01068884	9	10.8221282	2.13363365	3.93E-07	5.14E-01	1.00E+00	1.00E+00	HDAC9	HDAC9
rs2891865	1	229806368	A	G	421	0.0415677	35	5.58181376	1.12378916	6.80E-07	5.18E-01	1.00E+00	1.00E+00	URB2	URB2
rs16850124	1	229831331	T	C	421	0.04394299	37	5.18095662	1.09435188	2.20E-06	5.34E-01	1.00E+00	1.00E+00	URB2	URB2

rs75606013	7	18414613	G	A	421	0.00712589	6	14.6617119	2.61692421	2.11E-08	5.47E-01	1.00E+00	1.00E+00	HDAC9	HDAC9
rs290120	5	163268244	T	G	421	0.00712589	6	13.3587764	2.83260801	2.40E-06	5.69E-01	1.00E+00	1.00E+00	MAT2B	MAT2B
rs73085348	3	42711221	A	G	421	0.01187648	11	10.139317	2.03504571	6.28E-07	5.76E-01	1.00E+00	1.00E+00	STEAP2	STEAP2
rs111900874	7	89516669	G	A	421	0.01425178	12	8.42424013	1.82258334	3.80E-06	5.76E-01	1.00E+00	1.00E+00	STEAP2	STEAP2
rs148556485	9	84023826	A	C	421	0.0023753	3	21.3800152	4.26618316	5.40E-07	5.76E-01	1.00E+00	1.00E+00	TLE1	TLE1
rs180765647	13	114427311	G	T	421	0.0023753	3	20.1246521	4.37397806	4.20E-06	5.76E-01	1.00E+00	1.00E+00	GRK1	ATP4B
rs2327968	20	15813491	C	T	421	0.02494062	21	6.15899856	1.34390809	4.59E-06	5.83E-01	1.00E+00	1.00E+00	MACROD2	MACROD2
rs2365739	1	62484462	G	A	421	0.02137767	18	6.86815917	1.43887996	1.81E-06	5.88E-01	1.00E+00	1.00E+00	PATJ	PATJ
rs142993106	4	90957372	G	A	421	0.01781473	15	8.08463611	1.73306162	3.09E-06	5.88E-01	1.00E+00	1.00E+00	CCSER1	CCSER1
rs180828621	10	124533409	G	A	421	0.00712589	6	15.1380574	2.79957627	6.40E-08	5.90E-01	1.00E+00	1.00E+00	DMBT1L1	CUZD1
rs12315614	12	64920957	C	A	421	0.0760095	64	3.77710662	0.8201959	4.12E-06	6.20E-01	1.00E+00	1.00E+00	TBK1	TBK1
rs4562666	1	229824770	T	C	421	0.04275534	36	5.51922599	1.11457457	7.35E-07	6.58E-01	1.00E+00	1.00E+00	URB2	URB2
rs2876414	20	15813704	G	T	421	0.02256532	19	6.81910907	1.48530907	4.41E-06	6.60E-01	1.00E+00	1.00E+00	MACROD2	MACROD2
rs12036586	1	229826378	G	A	421	0.04750594	40	5.22212176	1.07114093	1.09E-06	6.73E-01	1.00E+00	1.00E+00	URB2	URB2
rs75334617	10	102956152	G	A	421	0.03800475	32	5.84603405	1.18027345	7.30E-07	7.59E-01	1.00E+00	1.00E+00	LINC01514	KAZALD1
rs78547898	22	32824278	G	A	421	0.00356295	3	20.9098395	4.15538274	4.85E-07	7.68E-01	1.00E+00	1.00E+00	BPIFC	FOX07
rs796777817	22	32824278	G	A	421	0.00356295	3	20.9098395	4.15538274	4.85E-07	7.68E-01	1.00E+00	1.00E+00	BPIFC	FOX07
rs2274996	1	229804538	C	T	421	0.0415677	35	5.58527433	1.12406363	6.74E-07	7.87E-01	1.00E+00	1.00E+00	URB2	URB2
rs143048774	14	46294660	A	C	421	0.31710214	265	2.28482299	0.49630858	4.15E-06	8.12E-01	1.00E+00	1.00E+00	LINC02303	MIS18BP1
rs428110	14	46294660	A	C	421	0.31710214	265	2.28482299	0.49630858	4.15E-06	8.12E-01	1.00E+00	1.00E+00	LINC02303	MIS18BP1
rs76526501	7	18431110	G	A	421	0.00712589	6	14.8397312	2.5778114	8.58E-09	8.23E-01	1.00E+00	1.00E+00	HDAC9	HDAC9
rs146526206	4	90993018	T	C	421	0.01781473	15	8.09176656	1.69535286	1.82E-06	8.30E-01	1.00E+00	1.00E+00	CCSER1	CCSER1
rs17017794	4	91825885	T	C	421	0.0391924	33	5.83240545	1.14450838	3.47E-07	9.15E-01	1.00E+00	1.00E+00	CCSER1	CCSER1
rs147559909	2	237051523	T	C	421	0.00593824	5	17.0840975	3.03209195	1.76E-08	9.53E-01	1.00E+00	1.00E+00	AGAP1	AGAP1
rs74521112	11	99089147	G	T	421	0.03206651	27	6.25632387	1.22787586	3.48E-07	9.53E-01	1.00E+00	1.00E+00	CNTN5	CNTN5
rs115348382	1	9655903	G	A	421	0.00356295	3	18.2107495	3.84721698	2.21E-06	NA				
rs186532456	1	18948328	C	T	421	0.00356295	3	20.1730343	4.11737745	9.61E-07	NA				
rs562032622	1	18959333	A	C	421	0.00356295	3	21.0267115	4.3197503	1.13E-06	NA				
rs149493615	1	79880089	G	A	421	0.00831354	8	11.750632	2.43451244	1.39E-06	NA				
rs143811231	1	79968131	T	C	421	0.00831354	8	11.5747409	2.42066027	1.74E-06	NA				
rs34270375	1	89370702	G	A	421	0.02612827	21	7.71147017	1.57216792	9.34E-07	NA				
rs187518659	1	99455745	T	G	421	0.00950119	8	11.6819623	2.46564537	2.16E-06	NA				
rs140420703	1	102803484	T	G	421	0.00475059	5	16.487654	3.46213891	1.91E-06	NA				
rs563167766	1	102866365	G	A	421	0.0023753	3	19.415739	4.24240395	4.73E-06	NA				
rs77180278	1	102961882	T	C	421	0.02850356	24	7.33619996	1.37404112	9.34E-08	NA				
rs112351653	1	103220360	T	C	421	0.02850356	24	7.42386947	1.33286772	2.55E-08	NA				
rs180926150	1	103226326	C	T	421	0.0023753	3	19.641918	4.24793649	3.77E-06	NA				
rs114413507	1	103419168	T	C	421	0.02850356	24	7.42216029	1.33294954	2.57E-08	NA				
rs116672066	1	103472916	G	A	421	0.02731591	23	8.04769476	1.37187844	4.46E-09	NA				
rs111928960	1	103633635	G	A	421	0.02850356	22	8.60257208	1.45827516	3.65E-09	NA				
rs113221952	1	103753974	A	G	421	0.02137767	19	7.92350704	1.61421682	9.17E-07	NA				
rs1856085	1	104114545	G	A	421	0.0023753	3	21.51144	4.26747898	4.64E-07	NA				
rs143597860	1	104157143	A	G	421	0.0023753	3	21.5748154	4.2760379	4.52E-07	NA				
rs144541665	1	104310729	G	A	421	0.0023753	3	21.8734611	4.30339839	3.72E-07	NA				
rs76098744	1	112349372	C	T	421	0.01662708	14	8.38612452	1.72704116	1.20E-06	NA				
rs76617932	1	180930424	T	C	421	0.01187648	9	10.5449608	2.25040726	2.79E-06	NA				
rs183180157	1	181247121	A	C	421	0.00950119	7	12.61386	2.68921121	2.72E-06	NA				
rs375790303	1	184530482	G	A	421	0.00831354	7	11.4240393	2.49719192	4.77E-06	NA				
rs138480898	1	184955657	C	T	421	0.00356295	3	16.9873974	3.66972373	3.67E-06	NA				
rs145766563	1	185129502	G	A	421	0.00356295	4	16.8682102	3.54127008	1.90E-06	NA				
rs147032554	1	186148864	T	G	421	0.00356295	3	18.162609	3.65402821	6.68E-07	NA				
rs180989936	1	193044178	A	G	421	0.00475059	4	17.1793579	3.70215494	3.48E-06	NA				
rs559559983	1	246977132	C	A	421	0.00712589	5	13.811132	2.89725588	1.87E-06	NA				
rs550763536	2	7452347	T	G	421	0.00593824	5	14.4871647	3.10349768	3.04E-06	NA				
rs558553658	2	15682724	C	T	421	0.00475059	3	17.1099107	3.29108061	2.01E-07	NA				
rs536781978	2	29733681	A	G	421	0.00356295	3	17.5870824	3.70259451	2.03E-06	NA				

rs568321148	2	29870348	T	G	421	0.00356295	3	17.7125046	3.7201251	1.92E-06	NA				
rs76777840	2	48312950	G	A	421	0.00593824	5	14.3500784	2.8927894	7.03E-07	NA				
rs145080832	2	48473143	G	A	421	0.00475059	4	17.1618477	3.25709537	1.37E-07	NA				
rs184220112	2	48631743	C	A	421	0.00475059	4	16.4879043	3.22267606	3.12E-07	NA				
rs189890455	2	48655397	C	T	421	0.00593824	5	15.1467391	2.99355083	4.20E-07	NA				
rs181193202	2	52527354	T	C	421	0.00356295	3	19.6312088	3.74776015	1.62E-07	NA				
rs183378658	2	52563371	C	T	421	0.00356295	3	19.7446169	3.72634921	1.17E-07	NA				
rs190193113	2	53085778	G	A	421	0.00356295	3	19.9533602	3.7619559	1.13E-07	NA				
rs187520610	2	53360041	G	A	421	0.00475059	4	18.8010396	3.19364595	3.93E-09	NA				
rs149421869	2	53483429	G	T	421	0.00831354	8	11.7382186	2.47204376	2.05E-06	NA				
rs146479102	2	65825759	G	A	421	0.00593824	5	15.4846768	3.26980199	2.18E-06	NA				
rs528288879	2	65875930	C	T	421	0.00593824	6	14.7878085	2.95121929	5.42E-07	NA				
rs11690187	2	67565909	A	C	421	0.00593824	5	13.6310532	2.9751322	4.61E-06	NA				
rs151272830	2	67682724	G	T	421	0.00712589	6	13.3323479	2.84806076	2.85E-06	NA				
rs186142189	2	67702707	G	A	421	0.00712589	6	12.7361927	2.71405368	2.70E-06	NA				
rs184200893	2	69260913	C	T	421	0.00356295	3	19.9580356	4.21905487	2.24E-06	NA				
rs111927235	2	74483954	A	G	421	0.00475059	4	17.0468955	3.46111889	8.43E-07	NA				
rs111838310	2	74673491	C	A	421	0.00475059	5	17.7963938	3.48429066	3.26E-07	NA				
rs112983626	2	74697150	G	A	421	0.00475059	4	17.7848079	3.48532494	3.35E-07	NA				
rs113006316	2	74802360	A	G	421	0.0023753	3	22.8886792	4.92411335	3.35E-06	NA				
rs76554191	2	95967628	G	A	421	0.04038005	34	5.72148039	1.2389174	3.87E-06	NA				
rs140352232	2	108038112	G	A	421	0.0023753	3	21.0576511	4.56013129	3.88E-06	NA				
rs116189766	2	126393864	T	C	421	0.0023753	3	21.190764	4.36037289	1.17E-06	NA				
rs139877408	2	129606273	A	G	421	0.0023753	3	20.3067129	4.43052497	4.58E-06	NA				
rs541508507	2	170023265	G	A	421	0.00475059	4	15.4630033	3.27389618	2.32E-06	NA				
rs142549310	2	170030506	C	T	421	0.00475059	4	15.2501713	3.24983634	2.70E-06	NA				
rs556293455	2	176950420	G	A	421	0.00356295	3	14.3860463	2.96998402	1.27E-06	NA				
rs184098071	2	177116420	G	A	421	0.00356295	3	13.947724	2.86782452	1.15E-06	NA				
rs532416695	2	177486790	G	A	421	0.00356295	3	19.8726656	3.76737683	1.33E-07	NA				
rs112557251	2	188375400	T	C	421	0.0023753	3	20.6510064	4.44850106	3.45E-06	NA				
rs185158855	2	223650026	C	A	421	0.00475059	4	16.6678864	3.60266524	3.72E-06	NA				
rs185510569	2	223814861	G	A	421	0.00356295	3	19.5886906	3.60490977	5.51E-08	NA				
rs181217257	2	239989119	C	T	421	0.00475059	4	21.5677368	3.47296989	5.29E-10	NA				
rs188076929	2	239993719	T	C	421	0.00475059	4	19.9775614	3.27246746	1.03E-09	NA				
rs112475378	3	1626661	T	C	421	0.01662708	14	9.55085929	1.97628138	1.35E-06	NA				
rs145676540	3	2044635	C	T	421	0.00593824	5	13.980658	2.88447377	1.25E-06	NA				
rs146007933	3	28227423	T	C	421	0.02137767	18	7.51149453	1.55442805	1.35E-06	NA				
rs73057656	3	33940571	A	G	421	0.0368171	33	5.9238329	1.27589711	3.44E-06	NA				
rs142684595	3	55319301	T	C	421	0.00593824	5	16.7729431	3.11364755	7.17E-08	NA				
rs80203220	3	122722331	C	T	421	0.00593824	6	13.426042	2.84218656	2.31E-06	NA				
rs192443987	3	135532345	G	A	421	0.00475059	4	17.7540285	3.67564302	1.36E-06	NA				
rs148248743	3	136134595	C	T	421	0.0023753	3	22.839379	4.62044689	7.69E-07	NA				
rs576124203	3	141841196	T	G	421	0.00356295	3	19.4039925	3.84392582	4.47E-07	NA				
rs545552231	3	141864350	C	T	421	0.00356295	3	19.8641332	3.94582563	4.80E-07	NA				
rs188720948	3	150069880	T	C	421	0.00356295	3	17.7032653	3.59722874	8.59E-07	NA				
rs139943877	3	155451289	G	A	421	0.00712589	7	13.0535902	2.76041594	2.26E-06	NA				
rs187047882	3	164285621	G	A	421	0.00356295	3	18.5641361	3.99725417	3.41E-06	NA				
rs141169929	3	164808462	A	G	421	0.00475059	5	14.8454178	3.1738839	2.91E-06	NA				
rs186649043	3	174932293	C	T	421	0.00356295	3	19.2134209	4.06588786	2.30E-06	NA				
rs189709453	3	177574308	G	A	421	0.00356295	3	21.6581488	4.05916142	9.52E-08	NA				
rs186767531	3	177639777	T	C	421	0.00356295	3	21.2013243	4.04780758	1.63E-07	NA				
rs182868205	3	177712448	C	T	421	0.00356295	3	20.9548768	3.97841839	1.39E-07	NA				
rs191792521	3	195646605	G	A	421	0.00831354	6	13.9515484	2.96443599	2.52E-06	NA				
rs189765693	4	4324793	T	C	421	0.00475059	4	15.5191341	3.29924607	2.55E-06	NA				
rs183962155	4	21259643	A	C	421	0.00593824	6	13.7646008	2.97247759	3.64E-06	NA				
rs113751774	4	23428854	C	T	421	0.00712589	7	14.9103274	2.96657459	5.01E-07	NA				

rs113063005	4	23509067	T	C	421	0.00593824	6	21.2073894	3.15520096	1.80E-11	NA				
rs145875128	4	32073136	G	A	421	0.00356295	3	24.479986	4.60639213	1.07E-07	NA				
rs143287889	4	35572280	C	T	421	0.0023753	3	19.96741	4.34406	4.30E-06	NA				
rs77141817	4	37053759	T	C	421	0.00356295	3	20.6110292	3.64214866	1.52E-08	NA				
rs190822761	4	37099356	G	T	421	0.00356295	3	20.5846354	3.64346559	1.61E-08	NA				
rs999769259	4	62511965	G	A	421	0.00356295	3	22.1246725	4.77597881	3.61E-06	NA				
rs145116559	4	112677596	T	C	421	0.00356295	4	18.2441224	3.9932536	4.91E-06	NA				
rs181415102	4	112689266	T	C	421	0.00356295	4	18.2631019	3.99387944	4.81E-06	NA				
rs191423619	4	126516627	G	T	421	0.0023753	3	21.694611	4.20667337	2.51E-07	NA				
rs149298750	4	127158701	A	C	421	0.00356295	3	22.184052	4.26573246	1.99E-07	NA				
rs112679237	4	139140860	T	C	421	0.01781473	18	8.71202752	1.67680671	2.04E-07	NA				
rs531769270	4	154411043	T	C	421	0.00356295	3	16.5945338	3.51936092	2.41E-06	NA				
rs116651654	4	163238743	C	T	421	0.00712589	6	15.6223694	3.06624451	3.49E-07	NA				
rs567982164	5	25631297	G	A	421	0.0023753	3	21.8356286	4.36706703	5.73E-07	NA				
rs191986449	5	25745687	C	T	421	0.00475059	3	18.4978793	4.03679831	4.60E-06	NA				
rs185771987	5	73285489	T	C	421	0.00356295	3	18.2344824	3.97675014	4.53E-06	NA				
rs139360368	5	73372109	A	C	421	0.00356295	4	18.3590487	3.78701272	1.25E-06	NA				
rs181933850	5	91465647	A	G	421	0.00831354	7	11.0436586	2.37544216	3.33E-06	NA				
rs190190051	5	91475485	G	A	421	0.00831354	7	11.0589887	2.38317975	3.48E-06	NA				
rs182531466	5	91530073	C	A	421	0.00712589	6	12.6804978	2.75685261	4.23E-06	NA				
rs187236873	5	91530447	G	A	421	0.00712589	6	12.44907	2.61780937	1.98E-06	NA				
rs183816745	5	91631888	A	G	421	0.00593824	5	17.3487909	3.20195556	6.02E-08	NA				
rs111407636	5	95080029	C	T	421	0.00356295	3	18.642774	3.74168052	6.28E-07	NA				
rs111676272	5	95094298	C	A	421	0.00356295	3	18.6068625	3.74429748	6.72E-07	NA				
rs75848314	5	95098340	T	C	421	0.00356295	3	20.0998481	3.78700451	1.11E-07	NA				
rs111846247	5	95111643	T	C	421	0.00356295	3	20.4865772	3.85467205	1.07E-07	NA				
rs137873790	5	97087041	A	G	421	0.01306413	11	9.22240881	2.01095379	4.52E-06	NA				
rs189912648	5	134765439	C	T	421	0.00356295	4	16.9732599	3.60846227	2.55E-06	NA				
rs191006910	5	154216009	G	A	421	0.00356295	3	17.6173803	3.79049689	3.36E-06	NA				
rs74343174	5	161493182	C	A	421	0.00593824	5	14.7671824	3.13913408	2.55E-06	NA				
rs775626702	5	162630067	A	C	421	0.00356295	3	18.9484137	4.07023372	3.23E-06	NA				
rs371245624	5	162880956	T	C	421	0.00356295	3	19.4795931	4.06572011	1.66E-06	NA				
rs545428520	5	167821266	T	C	421	0.00356295	4	21.566035	3.66407988	3.96E-09	NA				
rs528404963	5	167852025	T	C	421	0.00356295	4	21.1393977	3.65303517	7.17E-09	NA				
rs72832764	5	170004673	G	A	421	0.00356295	3	18.2159757	3.88196743	2.70E-06	NA				
rs72837643	5	170188955	T	C	421	0.00356295	4	16.9302874	3.67743479	4.15E-06	NA				
rs142311947	5	177384469	G	A	421	0.00712589	8	12.0339013	2.57618116	2.99E-06	NA				
rs151015676	5	177390937	T	G	421	0.0023753	3	20.1421367	4.37074482	4.06E-06	NA				
rs571986619	6	85678832	A	G	421	0.0023753	3	20.7858067	4.34301983	1.70E-06	NA				
rs56224400	6	98092675	T	C	421	0.01662708	14	8.4433064	1.72251471	9.50E-07	NA				
rs147627638	6	99173116	A	G	421	0.00712589	6	13.0785306	2.79364254	2.85E-06	NA				
rs141326851	6	134833127	A	C	421	0.01662708	14	8.57849688	1.81053268	2.16E-06	NA				
rs146048121	6	142198955	G	A	421	0.0023753	3	20.0214379	4.30086025	3.24E-06	NA				
rs142106992	6	142269885	C	A	421	0.00356295	4	22.8345886	3.6758699	5.23E-10	NA				
rs72983831	6	142307100	T	G	421	0.00593824	6	15.6733239	3.17294911	7.83E-07	NA				
rs72986533	6	142611258	T	C	421	0.00831354	8	13.2812726	2.53063629	1.54E-07	NA				
rs73586304	6	142839425	C	T	421	0.00356295	3	18.2405116	3.99428202	4.96E-06	NA				
rs148532212	6	165499857	T	C	421	0.00356295	3	19.3535457	3.70945693	1.81E-07	NA				
rs117498042	6	165514281	C	T	421	0.00356295	3	19.335471	3.70913521	1.86E-07	NA				
rs148153037	6	167501386	G	A	421	0.00831354	8	14.3359246	2.42128102	3.20E-09	NA				
rs184487573	6	167513471	A	G	421	0.00712589	7	13.5009635	2.59268014	1.92E-07	NA				
rs17776100	7	6426479	G	A	421	0.02969121	25	6.63697397	1.32177185	5.13E-07	NA				
rs187978759	7	11711845	G	A	421	0.00356295	3	20.4555522	4.3637719	2.76E-06	NA				
rs117166500	7	17052778	G	T	421	0.00712589	7	12.5146865	2.62694198	1.90E-06	NA				
rs55844051	7	23360363	T	C	421	0.00356295	3	18.3875896	3.86268704	1.93E-06	NA				
rs574076561	7	49544747	A	G	421	0.00356295	3	19.6434766	4.25225723	3.85E-06	NA				

rs181259864	7	97488823	C	A	421	0.00356295	3	17.1360821	3.68242789	3.26E-06	NA				
rs192750513	7	97577830	A	G	421	0.00356295	3	17.1892469	3.72514218	3.94E-06	NA				
rs539713344	7	100474786	G	A	421	0.0023753	3	20.044756	4.21957545	2.03E-06	NA				
rs188028357	7	100668425	C	T	421	0.0023753	3	21.8425578	4.35791809	5.38E-07	NA				
rs536023430	7	146865072	T	C	421	0.00356295	3	19.4038683	4.13084856	2.64E-06	NA				
rs187384541	8	1632241	A	G	421	0.03562945	26	6.88745117	1.47776668	3.15E-06	NA				
rs575473987	8	5577494	C	T	421	0.00356295	3	22.2248653	3.88625754	1.07E-08	NA				
rs139062456	8	13251991	C	T	421	0.01781473	15	7.83515809	1.69687423	3.89E-06	NA				
rs185874707	8	18030674	C	T	421	0.01187648	10	9.13934765	1.94918644	2.75E-06	NA				
rs140797780	8	22087792	C	T	421	0.00356295	3	19.2908396	3.69147785	1.73E-07	NA				
rs188415494	8	25613298	C	T	421	0.00356295	3	18.5169478	3.9914865	3.50E-06	NA				
rs117816016	8	103751262	C	T	421	0.00356295	3	19.8807023	4.26960212	3.22E-06	NA				
rs567383525	8	115029494	C	T	421	0.00356295	3	17.7455227	3.71970627	1.84E-06	NA				
rs545550279	8	115552429	G	T	421	0.00356295	3	19.1812421	4.19212988	4.75E-06	NA				
rs536803366	8	123001411	T	C	421	0.0023753	3	19.2098639	4.00952057	1.66E-06	NA				
rs555249476	8	123001411	T	C	421	0.0023753	3	19.2098639	4.00952057	1.66E-06	NA				
rs532513136	8	135813748	C	A	421	0.00356295	3	19.9490594	4.03118599	7.47E-07	NA				
rs532730683	9	1784492	G	T	421	0.00356295	3	18.726838	3.8314675	1.02E-06	NA				
rs540065886	9	2770228	T	C	421	0.00356295	3	19.9489151	4.18802805	1.90E-06	NA				
rs543844012	9	30107023	C	T	421	0.00356295	3	18.1160485	3.9446103	4.38E-06	NA				
rs188034471	9	85937714	G	A	421	0.00475059	4	14.7559999	3.14925113	2.79E-06	NA				
rs190294315	9	85945465	C	T	421	0.00593824	5	14.5664276	2.88126649	4.29E-07	NA				
rs545690161	9	93030699	G	A	421	0.00593824	6	17.1436874	3.00318779	1.14E-08	NA				
rs565682685	9	93222328	T	C	421	0.00475059	4	18.938397	3.7206174	3.58E-07	NA				
rs183737367	9	93330047	T	C	421	0.00356295	3	23.3452306	4.24471431	3.80E-08	NA				
rs187213609	9	93415465	C	T	421	0.00356295	3	23.1975304	4.27412656	5.72E-08	NA				
rs150027952	9	103385416	A	G	421	0.0023753	3	21.8616648	4.51464643	1.28E-06	NA				
rs146207930	9	129042336	A	G	421	0.00712589	6	11.8624321	2.51502465	2.40E-06	NA				
rs78296164	9	135266715	C	T	421	0.00831354	7	11.6095338	2.33917557	6.94E-07	NA				
rs77871739	9	138552309	G	A	421	0.00475059	4	15.0724603	3.23606181	3.20E-06	NA				
rs184425183	10	13457520	A	G	421	0.00356295	3	22.6683174	3.77549648	1.92E-09	NA				
rs184458518	10	13471195	T	G	421	0.00356295	3	22.8015789	3.77123942	1.48E-09	NA				
rs117998251	10	13497976	C	T	421	0.00356295	3	22.9543311	3.77480488	1.19E-09	NA				
rs117025967	10	20207052	C	A	421	0.01306413	11	9.66973563	1.927439	5.25E-07	NA				
rs529011661	10	20372316	G	A	421	0.00475059	4	15.6393469	3.3125339	2.34E-06	NA				
rs138249376	10	63515829	T	G	421	0.00475059	4	16.4300711	3.59466791	4.86E-06	NA				
rs140277951	10	82359100	G	A	421	0.00831354	6	13.7463158	2.64333932	1.99E-07	NA				
rs566018180	10	86755052	C	T	421	0.00356295	3	18.563552	4.04215862	4.38E-06	NA				
rs140706881	10	96217535	G	A	421	0.00475059	4	18.974805	3.93288931	1.40E-06	NA				
rs752259256	10	104925319	T	C	421	0.00356295	3	20.4258639	4.45411591	4.52E-06	NA				
rs147393020	10	124779274	A	G	421	0.00593824	5	14.1853505	2.95980896	1.65E-06	NA				
rs193093906	10	126705489	G	A	421	0.00950119	9	10.5219537	2.25840517	3.18E-06	NA				
rs151115079	11	18655741	T	C	421	0.00475059	5	17.8273587	3.2680205	4.89E-08	NA				
rs138414342	11	18679398	G	A	421	0.00475059	5	18.1582505	3.28214943	3.16E-08	NA				
rs541653703	11	18701786	G	A	421	0.00475059	4	18.3169499	3.27243418	2.18E-08	NA				
rs118093638	11	18718324	C	T	421	0.00593824	5	14.2657259	2.91296506	9.72E-07	NA				
rs181812512	11	66665729	C	T	421	0.00356295	3	19.5093168	4.24219816	4.25E-06	NA				
rs529345909	11	67110852	A	G	421	0.00356295	3	18.70153	4.09206035	4.87E-06	NA				
rs544042801	11	68460243	G	A	421	0.00475059	4	15.8869866	3.40519019	3.08E-06	NA				
rs149949098	11	95099866	G	A	421	0.01662708	13	8.55927625	1.81883308	2.53E-06	NA				
rs148781275	11	103640603	A	G	421	0.00475059	5	15.7111853	3.32183832	2.25E-06	NA				
rs141281289	11	123693024	A	G	421	0.00593824	6	15.811518	3.0889522	3.08E-07	NA				
rs528140343	11	125719227	A	C	421	0.00356295	3	18.2835521	3.72738029	9.33E-07	NA				
rs546409459	11	125754989	A	G	421	0.00356295	3	18.9195474	4.00286836	2.28E-06	NA				
rs528609331	11	125842195	C	T	421	0.00356295	3	19.5812418	3.94832705	7.07E-07	NA				
rs7104959	11	129846126	C	T	421	0.00356295	3	17.9204611	3.84366851	3.13E-06	NA				

rs189360484	12	1870510	A	G	421	0.00475059	4	16.8078794	3.31424855	3.95E-07	NA				
rs141754456	12	20151132	T	C	421	0.00712589	7	12.1460333	2.32842921	1.82E-07	NA				
rs118184666	12	20424749	G	A	421	0.00712589	6	10.9509803	2.32477242	2.47E-06	NA				
rs549931083	12	20516286	A	C	421	0.00356295	3	14.3282694	2.79891395	3.07E-07	NA				
rs151323346	12	21012024	T	C	421	0.00593824	4	15.8903888	3.38034868	2.59E-06	NA				
rs371879555	12	23015962	T	C	421	0.00356295	3	20.1033765	4.20002323	1.70E-06	NA				
rs183466664	12	26821687	A	G	421	0.00475059	4	16.0181882	3.45609016	3.57E-06	NA				
rs77353774	12	28248852	G	A	421	0.00712589	6	13.1054362	2.67954103	1.00E-06	NA				
rs113167689	12	28435962	C	T	421	0.00712589	6	14.1364135	2.73335629	2.32E-07	NA				
rs17510814	12	28468969	A	C	421	0.00712589	6	14.0387924	2.71409689	2.31E-07	NA				
rs141756120	12	28511096	A	C	421	0.00831354	6	14.278403	2.76458538	2.41E-07	NA				
rs117991215	12	28511473	T	C	421	0.00831354	6	14.333666	2.77918736	2.50E-07	NA				
rs191930622	12	48284655	G	A	421	0.0023753	3	19.6783579	4.30610368	4.88E-06	NA				
rs56302696	12	48292830	G	A	421	0.0023753	3	19.8959041	4.31584707	4.03E-06	NA				
rs185620578	12	48569399	C	T	421	0.0023753	3	20.7232267	4.39505442	2.42E-06	NA				
rs190806532	12	48862147	G	T	421	0.0023753	3	21.326955	4.43856043	1.55E-06	NA				
rs568658857	12	49853998	G	A	421	0.00593824	5	14.2361193	3.09423422	4.21E-06	NA				
rs137880949	12	63306297	T	C	421	0.00475059	3	21.6084034	4.13589168	1.75E-07	NA				
rs191053292	12	63445280	T	C	421	0.00356295	3	22.9266488	4.11439732	2.51E-08	NA				
rs182437250	12	63608466	T	C	421	0.00475059	4	18.8083451	3.79343554	7.12E-07	NA				
rs76904423	12	101188744	G	A	421	0.01068884	10	9.80576231	2.11568908	3.57E-06	NA				
rs180764936	12	101498621	T	C	421	0.00356295	3	19.3473053	4.0200238	1.49E-06	NA				
rs185855183	12	101505126	C	T	421	0.00356295	3	18.7397727	3.86137343	1.22E-06	NA				
rs139598422	13	23887014	A	G	421	0.00356295	4	20.4886522	3.64167194	1.84E-08	NA				
rs143371352	13	47383834	C	T	421	0.00356295	3	20.5341625	3.87928964	1.20E-07	NA				
rs75186966	13	47395758	A	C	421	0.00356295	3	20.2281345	3.83225523	1.30E-07	NA				
rs150077525	13	57979549	A	G	421	0.00712589	7	12.9984875	2.71312414	1.66E-06	NA				
rs534845494	13	58213864	A	G	421	0.00475059	5	15.9431765	3.18484007	5.56E-07	NA				
rs140062526	13	59286033	G	A	421	0.00593824	5	14.0926088	3.02052903	3.08E-06	NA				
rs546286713	13	91411079	G	A	421	0.00475059	4	15.7027841	3.3611216	2.98E-06	NA				
rs567080482	13	95075405	T	C	421	0.00475059	4	15.4999561	3.31620837	2.95E-06	NA				
rs142928734	13	101601082	G	A	421	0.00356295	3	17.009593	3.70044989	4.29E-06	NA				
rs556680896	13	101602415	C	T	421	0.00356295	3	17.0254659	3.70286799	4.27E-06	NA				
rs184265355	13	108011352	A	C	421	0.00593824	6	14.4271719	2.91829792	7.67E-07	NA				
rs572961122	13	108012985	C	T	421	0.00593824	6	13.3354896	2.85827607	3.08E-06	NA				
rs528809914	13	113041256	G	A	421	0.00356295	3	19.5048552	3.76867111	2.27E-07	NA				
rs138215817	14	22641516	A	G	421	0.00475059	4	16.311019	3.27367022	6.28E-07	NA				
rs74704551	14	30161887	C	T	421	0.00356295	3	24.1990705	4.31064693	1.98E-08	NA				
rs116862847	14	64141677	C	T	421	0.00712589	7	15.3160369	2.95194262	2.12E-07	NA				
rs569916471	14	75891342	G	A	421	0.00475059	5	15.6404088	3.3537133	3.11E-06	NA				
rs113767990	14	81717563	G	A	421	0.00475059	4	14.5811155	3.19363095	4.98E-06	NA				
rs190251199	14	105590577	T	C	421	0.00356295	3	20.0125292	4.01627173	6.27E-07	NA				
rs185155853	15	41244100	C	T	421	0.00475059	5	15.9164657	3.40838631	3.02E-06	NA				
rs144026361	15	41248669	C	T	421	0.00475059	5	16.1372994	3.43734659	2.67E-06	NA				
rs558614420	15	41810870	C	T	421	0.00593824	6	13.9750831	3.02772732	3.92E-06	NA				
rs138109686	15	42051442	A	G	421	0.00593824	6	13.5956705	2.96761718	4.62E-06	NA				
rs145896760	15	42119222	G	A	421	0.00593824	6	13.3688825	2.92346809	4.81E-06	NA				
rs140642138	15	42125165	G	A	421	0.00593824	6	13.5832221	2.96274049	4.55E-06	NA				
rs6080	15	58837933	C	A	421	0.04394299	36	5.66476745	1.2393253	4.86E-06	NA				
rs145439370	15	58879765	T	C	421	0.03087886	26	6.89328891	1.40339117	9.02E-07	NA				
rs149425014	15	58951660	T	C	421	0.02612827	22	7.55224792	1.58320604	1.84E-06	NA				
rs146442492	15	58982115	C	T	421	0.02731591	24	7.05925858	1.51661316	3.25E-06	NA				
rs193253461	15	59229353	A	G	421	0.01306413	12	10.5374468	2.07404763	3.76E-07	NA				
rs184117160	15	59404306	C	T	421	0.01425178	11	10.3417236	2.13366338	1.25E-06	NA				
rs80292573	15	59435086	T	G	421	0.03444181	30	6.55323491	1.32696583	7.87E-07	NA				
rs182303755	15	59634792	A	C	421	0.01306413	12	10.5479917	2.09359291	4.70E-07	NA				

rs138217865	15	94392882	C	T	421	0.00475059	4	16.709641	3.45193896	1.29E-06	NA				
rs553840536	16	25697895	A	G	421	0.00356295	3	19.6203442	4.23547954	3.61E-06	NA				
rs183817723	16	59302775	C	T	421	0.00356295	4	17.5860507	3.7341919	2.48E-06	NA				
rs144954214	16	76179362	A	G	421	0.0023753	3	21.8113263	4.43942529	8.96E-07	NA				
rs529523094	16	77715551	A	G	421	0.00356295	3	18.1584346	3.72617419	1.10E-06	NA				
rs146728064	17	19265440	G	A	421	0.00712589	6	12.6400411	2.73467115	3.80E-06	NA				
rs191271637	17	52123260	A	G	421	0.00356295	3	18.379836	3.92083355	2.76E-06	NA				
rs185819304	18	27001580	G	A	421	0.00356295	3	19.9401113	3.94501953	4.32E-07	NA				
rs187942235	18	27030430	C	T	421	0.00356295	3	20.3968658	3.96963025	2.77E-07	NA				
rs143538552	18	29050262	A	G	421	0.00356295	3	19.1457218	3.63679703	1.41E-07	NA				
rs373746073	18	29058384	C	A	421	0.00356295	3	19.0847197	3.63798064	1.55E-07	NA				
rs146333745	18	55497457	C	T	421	0.00356295	4	17.4215784	3.56782335	1.04E-06	NA				
rs185464792	19	18797371	C	T	421	0.0023753	3	22.5896015	4.50191473	5.23E-07	NA				
rs186768950	19	18806124	C	A	421	0.0023753	3	22.7152061	4.50776479	4.68E-07	NA				
rs559008174	19	18876059	C	T	421	0.00475059	5	15.6025661	3.12812006	6.11E-07	NA				
rs546144116	19	19563339	C	T	421	0.0023753	3	23.1354815	4.47162758	2.29E-07	NA				
rs560206697	19	20729098	C	T	421	0.0023753	3	24.9989943	4.55350673	4.02E-08	NA				
rs111285015	19	23123198	G	A	421	0.00356295	3	27.3347642	4.62083983	3.31E-09	NA				
rs1008091735	19	31090099	T	C	421	0.00356295	3	18.4689243	3.69079909	5.61E-07	NA				
rs148433854	19	31096478	G	A	421	0.00356295	3	18.6267585	3.68806754	4.41E-07	NA				
rs140788628	20	15858501	C	A	421	0.01068884	8	11.8039284	2.2826396	2.33E-07	NA				
rs559228693	20	15963329	G	A	421	0.00593824	5	14.3643551	3.08196497	3.15E-06	NA				
rs184785969	21	17171431	C	A	421	0.00356295	3	19.8736486	3.70683937	8.26E-08	NA				
rs117280553	21	17207163	T	C	421	0.00356295	3	20.087417	3.70274826	5.80E-08	NA				
rs79486609	21	17245006	G	A	421	0.00356295	3	20.6624886	3.77824957	4.53E-08	NA				
rs73227413	21	23136973	G	A	421	0.03444181	28	5.96205514	1.27150176	2.75E-06	NA				
rs75024143	21	23156546	G	T	421	0.01425178	12	9.81254241	2.0684542	2.10E-06	NA				
rs192134381	21	23450714	T	C	421	0.00356295	3	21.788812	3.73125357	5.23E-09	NA				
rs397836601	21	23450714	T	C	421	0.00356295	3	21.788812	3.73125357	5.23E-09	NA				
rs118183140	21	35477486	C	T	421	0.02019002	17	7.5893248	1.56122232	1.17E-06	NA				
rs183586634	21	38763032	G	A	421	0.00593824	5	14.3132202	2.99210048	1.72E-06	NA				
rs117185941	21	38766484	G	A	421	0.00593824	5	15.3808786	3.17143013	1.24E-06	NA				
rs1329159859	21	38766484	G	A	421	0.00593824	5	15.3808786	3.17143013	1.24E-06	NA				
rs118084887	21	38863820	T	C	421	0.00593824	5	14.9382616	3.13674741	1.91E-06	NA				
rs150539922	21	43276916	T	C	421	0.00356295	3	16.6696937	3.64477381	4.79E-06	NA				
rs113625788	22	19969182	C	T	421	0.00831354	7	11.8084829	2.42941561	1.17E-06	NA				
rs541680196	22	40528090	G	A	421	0.00593824	5	14.3867808	2.94903659	1.07E-06	NA				
rs185139807	22	40594781	G	A	421	0.00593824	5	14.4330526	2.95312686	1.02E-06	NA				
rs141127122	22	40604439	G	A	421	0.00475059	4	15.3824875	3.30083549	3.16E-06	NA				
rs148998974	22	40620530	A	G	421	0.00593824	5	14.5129532	2.94976024	8.65E-07	NA				
rs555040883	22	40631476	G	A	421	0.00475059	4	15.4273069	3.30542717	3.05E-06	NA				
rs150946694	22	46853180	T	C	421	0.00475059	4	15.3082479	3.29125059	3.30E-06	NA				

Supplementary Table S22. Independent Replication Meta-Analysis
Indianpolis-2 cohort, 3M quantitative trait (QT) and Florida-1 cohort, comparing to discovery 3M QT

Notes
Grayed boxes = improved p-values
Standard Headers
rsID: reference SNP cluster ID; p.{cohort]: p-value for the SNP in the indcated cohort
Z_meta: Z-score met-analysis; p_meta: p-value meta-analysis

rsID	p.Indianapolis-1	p.Indianapolis-2	p.Florida-1	Z_meta	p_meta	Risk locus
rs16823323	3.35704E-08	2.11E-01		5.501743324	3.76E-08	
rs142106992	5.23067E-10	8.80E-01		5.495807745	3.89E-08	
rs111285015	3.30788E-09	7.98E-01		5.411442856	6.25E-08	
rs189890455	4.19718E-07	2.10E-01		5.088530698	3.61E-07	FOXN2;PPP1R21
rs117913371	2.3064E-08	9.79E-01		5.01492616	5.31E-07	
rs112007361	2.27562E-07	4.11E-01		5.000517933	5.72E-07	
rs184613584	5.40834E-07	2.52E-01		4.996865834	5.83E-07	
rs145439370	9.02073E-07	2.26E-01		4.936961035	7.93E-07	LIPC;ADAM10
rs74521112	3.48297E-07	4.30E-01		4.91427519	8.91E-07	
rs76777840	7.02623E-07	3.01E-01		4.902501754	9.46E-07	
rs1877768	2.7763E-06	1.13E-01		4.901999554	9.49E-07	ATXN1
rs139062456	3.88566E-06	9.02E-02		4.888890822	1.01E-06	DLC1
rs79213709	5.77809E-07	4.30E-01		4.827523118	1.38E-06	
rs187384541	3.15111E-06	1.56E-01		4.805302698	1.55E-06	DLGAP2
rs149425014	1.84021E-06	2.65E-01		4.768008343	1.86E-06	
rs141281289	3.07597E-07	8.12E-01		4.689389951	2.74E-06	
rs142993106	3.087E-06	2.84E-01		4.654040118	3.25E-06	
rs193253461	3.76206E-07	8.29E-01		4.645963357	3.38E-06	
rs80292573	7.87215E-07	6.19E-01		4.643780334	3.42E-06	
rs146442492	3.24578E-06	3.05E-01		4.625076996	3.74E-06	
rs72986533	1.53597E-07	8.65E-01		4.623896588	3.77E-06	
rs6080	4.85755E-06	2.85E-01		4.569176387	4.90E-06	
rs76098744	1.19916E-06	6.41E-01		4.555548554	5.22E-06	
rs74683551	1.21215E-06	6.39E-01		4.55503049	5.24E-06	
rs112679237	2.04054E-07	8.23E-01		4.55300091	5.29E-06	
rs17746486	1.47155E-06	6.45E-01		4.516724818	6.28E-06	
rs2274996	6.73602E-07	8.87E-01		4.512537344	6.41E-06	
rs2274997	6.64244E-07	8.95E-01		4.510967459	6.45E-06	
rs2891865	6.80113E-07	8.87E-01		4.510866526	6.46E-06	
rs2385790	6.81207E-07	8.93E-01		4.507233855	6.57E-06	
rs12024557	6.90833E-07	8.94E-01		4.504454103	6.65E-06	
rs4562666	7.35045E-07	8.87E-01		4.497353283	6.88E-06	
rs176786	3.37673E-06	4.74E-01		4.479329233	7.49E-06	
rs1686289	3.52213E-06	4.81E-01		-4.466730671	7.94E-06	
rs143048774	4.15182E-06	4.42E-01		4.464969284	8.01E-06	
rs176783	3.87832E-06	4.69E-01		4.457203452	8.30E-06	
rs76554191	3.87215E-06	4.75E-01		4.453460654	8.45E-06	
rs147393020	1.64586E-06	7.19E-01		4.452211025	8.50E-06	
rs546286713	2.98444E-06	5.53E-01		4.447971928	8.67E-06	
rs2365739	1.81261E-06	7.20E-01		4.434029133	9.25E-06	
rs118183140	1.1671E-06	8.59E-01		4.432070145	9.33E-06	
rs184117160	1.25385E-06	8.43E-01		4.428534495	9.49E-06	
rs12045643	7.37333E-07	9.88E-01		4.426848411	9.56E-06	

rs73085348	6.28156E-07	9.37E-01		4.426456492	9.58E-06
rs17776100	5.13308E-07	7.78E-01		4.371150819	1.24E-05
rs117025967	5.25166E-07	7.67E-01		4.360753866	1.30E-05
rs73057656	3.43591E-06	6.94E-01		4.332724562	1.47E-05
rs191792521	2.52243E-06	7.98E-01		4.328401698	1.50E-05
rs141754456	1.82428E-07	4.24E-01		4.315059844	1.60E-05
rs75024143	2.09641E-06	8.89E-01		4.310338793	1.63E-05
rs16850124	2.19846E-06	8.87E-01		4.302495759	1.69E-05
rs140788628	2.32627E-07	4.12E-01		4.265889529	1.99E-05
rs12036586	1.08649E-06	7.65E-01		4.23256277	2.31E-05
rs1221830047	1.50406E-07	2.69E-01		4.210761623	2.55E-05
rs75334617	7.30335E-07	5.28E-01		4.154513576	3.26E-05
rs4737674	2.86005E-07	2.76E-01		4.110719966	3.94E-05
rs16920698	2.85869E-07	2.72E-01		4.107717835	4.00E-05
rs13277510	2.86106E-07	2.72E-01		4.107563819	4.00E-05
rs4737676	2.85893E-07	2.72E-01		4.107432866	4.00E-05
rs983248	2.87356E-07	2.72E-01		4.106572595	4.02E-05
rs1391463	2.87383E-07	2.72E-01		4.106164828	4.02E-05
rs4737201	2.89096E-07	2.67E-01		4.100275223	4.13E-05
rs10105693	3.41214E-07	2.95E-01		4.100229538	4.13E-05
rs11987234	3.1319E-07	2.72E-01		4.09213909	4.27E-05
rs13276543	3.13999E-07	2.72E-01		4.091846031	4.28E-05
rs13278605	3.41582E-07	2.73E-01		4.078289694	4.54E-05
rs2375537	6.2992E-07	2.39E-01	0.304091648	4.064722311	4.81E-05
rs1561297	3.34668E-07	2.39E-01		4.045303127	5.23E-05
rs12548593	3.38344E-07	2.38E-01		4.042759768	5.28E-05
rs2083123	3.43585E-07	2.38E-01		4.040084054	5.34E-05
rs76327548	2.03545E-06	6.01E-01		4.021015713	5.79E-05
rs423841	2.75088E-06	1.76E-01	0.046987035	-4.020403529	5.81E-05
rs187518659	2.15952E-06	5.84E-01		3.999129111	6.36E-05
rs1437782	5.53725E-07	2.73E-01		3.995695786	6.45E-05
rs149421869	2.05046E-06	5.64E-01		3.995691624	6.45E-05
rs10958428	5.01124E-07	2.38E-01		3.97592508	7.01E-05
rs9643828	2.29737E-07	1.47E-01	0.590916281	-3.971106415	7.15E-05
rs147630370	7.46814E-07	2.88E-01		3.958557714	7.54E-05
rs858397	7.16198E-07	2.73E-01		3.950598461	7.80E-05
rs446222	7.36959E-07	2.73E-01		-3.946145181	7.94E-05
rs384127	7.36964E-07	2.72E-01		-3.945036897	7.98E-05
rs382476	7.36949E-07	2.71E-01		-3.943904631	8.02E-05
rs384543	7.36949E-07	2.71E-01		-3.943904631	8.02E-05
rs1437781	6.32222E-07	2.39E-01		3.936328071	8.27E-05
rs369623	8.50611E-07	2.71E-01		-3.919175565	8.89E-05
rs76904423	3.5731E-06	6.01E-01		3.917382807	8.95E-05
rs183180157	2.72481E-06	4.78E-01		3.884364848	1.03E-04
rs149949098	2.52728E-06	4.37E-01		3.868123089	1.10E-04
rs146007933	1.34951E-06	2.61E-01		3.826911762	1.30E-04
rs432393	1.22958E-06	2.37E-01		-3.817751616	1.35E-04
rs405226	1.22513E-06	2.36E-01		-3.817526823	1.35E-04
rs3098298	1.2282E-06	2.36E-01		-3.817083246	1.35E-04
rs367179	1.2282E-06	2.36E-01		-3.817083246	1.35E-04
rs433324	9.22326E-07	1.90E-01		-3.811576843	1.38E-04
rs111900874	3.79797E-06	3.99E-01		3.763512576	1.68E-04
rs111391231	4.17997E-06	3.96E-01		3.743817403	1.81E-04
rs116651654	3.48829E-07	6.24E-02		3.733193891	1.89E-04
rs17017794	3.4692E-07	5.69E-02		3.716092414	2.02E-04

rs147171192	4.39967E-06	3.47E-01		3.693130181	2.22E-04
rs2375536	2.13408E-06	2.04E-01		3.679597387	2.34E-04
rs1812506	2.00833E-06	1.86E-01		3.667511405	2.45E-04
rs73227413	2.74561E-06	2.27E-01		3.66172474	2.51E-04
rs2876414	4.41049E-06	4.80E-01	0.047073158	3.643568363	2.69E-04
rs1595406	2.80264E-06	1.87E-01		3.607602949	3.09E-04
rs139943877	2.25796E-06	1.58E-01		3.606687701	3.10E-04
rs720372	2.93015E-06	1.87E-01		3.599578979	3.19E-04
rs2327968	4.58568E-06	4.83E-01	0.033057708	3.591342782	3.29E-04
rs1301444047	2.45197E-06	1.57E-01		3.589412355	3.31E-04
rs2375219	3.05253E-06	1.46E-01		3.532031785	4.12E-04
rs12266995	3.92981E-06	1.27E-01		3.453254083	5.54E-04
rs1498183	4.76724E-06	1.43E-01		3.444243226	5.73E-04
rs12678939	4.93547E-06	1.42E-01		3.436648484	5.89E-04
rs1391462	4.7944E-06	1.30E-01		3.421102345	6.24E-04
rs1396896	4.7781E-06	1.25E-01		3.412678552	6.43E-04
rs7843693	4.78812E-06	1.24E-01		3.412132741	6.45E-04
rs12315614	4.12213E-06	1.27E-01	0.03306202	2.663219712	7.74E-03

Supplemental Table S23. Functional Annotation Prioritized Target Genes
Pathway Enrichment Analysis

Notes
Events identified by Reactome; enrichment scores determined using DAVID Bioinformatics functional annotation tool; analysis performed at medium stringency s
Events ordered by DAVID enrichment score for GWAS prioritized target genes
DEGs: genes differentially regulated by glucocorticoids (GCs)
GWAS prioritized target genes list: see Table S8; Paired eye study DEGs lists: see Table S10

Reactome Events	DAVID Enrichment Scores											
	GWAS prioritized target genes	P-Value	Benjamimi Adjusted P-Value	Human paired eye study all responder DEGs	P-Value	Benjamimi Adjusted P-Value	Human paired eye study down-regulated responder DEGs	P-Value	Benjamimi Adjusted P-Value	Bovine paired eye study all responder DEGs	P-Value	Benjamimi Adjusted P-Value
Muscle contraction	1.46	5.38E-01	1.00E+00				2.48	7.06E-04	2.83E-02			
Muscle contraction: cardiac conduction	1.46	1.98E-01	1.00E+00	2.48	1.65E-04	1.03E-02	2.48	1.89E-03	5.52E-02			
Muscle contraction: cardiac conduction: Phase 0 – rapid depolarisation				0.97	1.73E-02	2.87E-01	0.94	2.07E-01	1.00E+00			
Muscle contraction: cardiac conduction: Phase 1 - inactivation of fast Na+ channels	1.46	3.97E-04	2.12E-01									
Muscle contraction: cardiac conduction: Phase 2 - plateau phase												
Muscle contraction: cardiac conduction: Phase 3 - rapid repolarisation												
Signal Transduction: Signaling by NOTCH	1.42	4.18E-02	1.00E+00							0.36	8.84E-01	1.00E+00
Signal transduction: signaling by NOTCH 1	1.42	6.68E-03	8.16E-01									
Signal transduction: signaling by NOTCH 2	1.42	2.18E-01	1.00E+00									
Signal transduction: signaling by NOTCH 3	1.42	9.86E-02	1.00E+00							0.36	8.84E-01	1.00E+00
Signal Transduction: Signaling by NOTCH: NOTCH 3 Activation and Transmission of Signal to the Nucleus	1.42	1.13E-01	1.00E+00							0.36	1.99E-01	1.00E+00
Signal Transduction: Signaling by NOTCH: Pre-NOTCH Processing in Golgi										0.31	1.52E-01	1.00E+00
Disease: diseases of signal transduction by growth factor receptors and second messengers [cancer]	1.42	1.73E-01	1.00E+00	0.46	4.88E-01	1.00E+00	0.36	5.92E-01	1.00E+00	0.61	5.51E-01	1.00E+00
Metabolism: metabolism of carbohydrates	1.41	5.60E-01	1.00E+00							0.82	3.06E-01	1.00E+00
Metabolism: metabolism of carbohydrates: Heparan sulfate/heparin (HS-GAG) metabolism	1.41	2.67E-03	8.16E-01									
Metabolism: metabolism of carbohydrates: HS-GAG biosynthesis	1.41	7.46E-03	8.16E-01									
Metabolism: metabolism of carbohydrates: HS-GAG degradation	1.41	7.68E-02	1.00E+00									
Metabolism: metabolism of carbohydrates: glycosaminoglycan metabolism	1.41	1.00E-01	1.00E+00							0.81	4.81E-01	1.00E+00
Disease: Diseases associated with glycosaminoglycan metabolism										0.81	4.89E-02	8.17E-01
